# Supplementary material for: 'Targeting' the search: An upgraded structural and functional repository of antimicrobial peptides for biofilm studies (B-AMP v2.0) with a focus on biofilm protein targets
Source: Front Cell Infect Microbiol. 2022 Oct 18;12:1020391. doi: 10.3389/fcimb.2022.1020391 (PMC9623296; doi:10.3389/fcimb.2022.1020391)
Supplement: Supplementary File 1 — Non-redundant list of 2502 biofilm protein targets in B-AMP v2.0 [file Table_1.pdf]

Target ID	Bacterial Protein Name	Organism Name	Strain	UniProt ID	PDB ID	PubMed ID	Classification as per UniProt GO Molecular Function	Classification of Function as per PDB
1	Biofilm-associated surface protein	*Staphylococcus aureus*		Q79LN3	7C7U	11292810; 34046916	Not Available	Cell adhesion
2	Type 1 fimbrin D-mannose specific adhesin (Protein FimH)	*Escherichia coli*	Strain K12	P08191	6YHW	2890081; 7905476; 7610040; 9278503; 16738553; 2900235; 1971261; 12010488	Mannose-binding	Cell adhesion
3	Fimbrial adhesin	*Proteus mirabilis*	Strain HI4320	B4EUK6	6Y4E	18375554; 32780778	Not Available	Cell adhesion
4	Putative large adhesion protein (Lap) involved in biofilm formation	*Marinobacter nauticus*	Strain ATCC 49840 / DSM 8798 / CIP 103578 / SP17	H8W6K8	6XI3	22689231; 31335890; 32984811	Calcium ion binding	Metal binding protein
5	Minor fimbrium subunit Mfa5	*Porphyromonas gingivalis*	Strain ATCC 33277 / DSM 20709 / CIP 103683 / JCM 12257 / NCTC 11834 / 2561	B2RHG5	6TNJ	18524787; 19589838; 24118823; 26001707; 26437277	Not Available	Cell adhesion
6	Fimbrin-like protein FimI	*Escherichia coli*	Strain K12	P39264	6SWH	7610040; 9278503; 16738553; 6147250	Not Available	Structural protein
7	TasA anchoring / assembly protein (Biofilm assembly accessory protein TapA)	*Bacillus subtilis*	Strain 168	P40949	6QAY	8969508; 9384377; 2507524; 10464223; 10559173; 16430695; 16430696; 21477127; 23646920; 24488317	Not Available	Protein fibril
8	Sensor protein SrrB (EC 2.7.13.3) (Staphylococcal respiratory response protein B)	*Staphylococcus aureus*		Q9L523	6PAJ, 7JL6	11157922; 15060046, 32354997	ATP binding, phosphorelay sensor kinase activity	Membrane protein
9	Uncharacterized fimbrial-like protein YdeQ	*Escherichia coli*	Strain K12	P77588	6MAP	9097039; 9278503; 16738553	Not Available	Sugar binding protein
10	Transcriptional regulatory protein LytR	*Staphylococcus aureus*	Strain NCTC 8325 / PS 47	P60611	6M8O	8550490; 8824633; 19502411; 23733465; 25491472	DNA-binding Transcription activator activity, phosphorelay response regulator activity, Transcription cis-regulatory region binding	Signaling protein
11	Curli production assembly / transport component CsgG	*Escherichia coli*	Strain K12	P0AEA2	6L7C	8817489; 8905232; 9278503; 16738553	Identical protein binding	Protein transport
12	Major fimbrium subunit FimA type-1 (Fimbrillin) (Fimbrilin) (Major fimbrial subunit protein type I)	*Porphyromonas gingivalis*	Strain ATCC 33277 / DSM 20709 / CIP 103683 / JCM 12257 / NCTC 11834 / 2561	B2RH54	6JZK	7902712; 18524787; 8778568; 9786913; 10377095; 12593606; 15165251; 17675496; 17526848; 20530728; 23809984; 27062925	Structural molecule	Cell adhesion
13	RNA polymerase sigma-H factor (Sigma-30)	*Porphyromonas gingivalis*	Strain ATCC 15692 / DSM 22644 / CIP 104116 / JCM 14847 / LMG 12228 / 1C / PRS 101 / PAO1	Q06198	6IN7	8432708; 8378309; 7961421; 10984043; 7737518	DNA-binding Transcription activator activity, sigma factor activity, Transcription cis-regulatory region binding	Transcription
14	Major curlin subunit	*Escherichia coli*	Strain K12	P28307	6G8C	8459772; 8817489; 8905232; 9278503; 16738553; 1357528; 1677357; 17010156	Identical protein binding	Protein fibril
15	Methyl-accepting chemotaxis protein TlpQ (TlpQ chemoreceptor)	*Porphyromonas gingivalis*	Strain ATCC 15692 / DSM 22644 / CIP 104116 / JCM 14847 / LMG 12228 / 1C / PRS 101 / PAO1	Q9I0I4	6FU4	10984043; 33097715; 30425146	Not Available	Signaling protein
16	Streptococcal hemagglutinin (Hs antigen) (Sialic acid-binding adhesin)	*Streptococcus gordonii*	Strain ChalliS / ATCC 35105 / BCRC 15272 / CH1 / DL1 / V288	A8AWU7	6EFC, 6EFD, 6X3K	11854202; 17720781; 8366515; 15213130; 19884334; 21071690; 27616700	Not Available	Cell adhesion
17	Putative hemin storage protein	*Bordetella bronchiseptica*	Strain ATCC BAA-588 / NCTC 13252 / RB50	A0A0H3LKK6	6AU1	12910271; 29684093	Hydrolase activity, acting on carbon-nitrogen (but not peptide) bonds	Hydrolase
18	Autoinducer-2 kinase (AI-2 kinase) (EC 2.7.1.189)	*Escherichia coli*	Strain K12	P77432	5YA1	9097039; 9278503; 16738553; 8649811; 15601708; 17557827; 20025244	Autoinducer-2 kinase activity, phosphoTransferase activity, alcohol group as acceptor	Structural protein
19	Putative outer Membrane protein	*Salmonella typhimurium*	Strain LT2 / SGSC1412 / ATCC 700720	Q8ZRK4	5Y9H	11677609; 16793551; 18448124; 29125121	Not Available	Cell adhesion
20	Putative fimbriae subunit	*Salmonella typhimurium*	Strain LT2 / SGSC1412 / ATCC 700720	Q7CR56	5Y9H	11677609; 29125121	Not Available	Cell adhesion
21	Putative ABC transporter, ATP-binding protein ComA	*Streptococcus mutans serotype C*	Strain ATCC 700610 / UA159	Q8DW05	5XE9	12397186; 20100826; 23534432; 28642545	ABC-type bacteriocin transporter activity, ATP binding, cysteine-type peptidase activity	Hydrolase

Target ID	Bacterial Protein Name	Organism Name	Strain	UniProt ID	PDB ID	PubMed ID	Classification as per UniProt GO Molecular Function	Classification of Function as per PDB
22	HTH-type Transcriptional regulator RcdA (Regulator of csgD)	*Escherichia coli*	Strain K12	P75811	5X5I	8905232; 9278503; 16738553; 23233451	DNA binding transcription factor activity	Transcription
23	CRISPR-associated protein Csy1	*Pseudomonas aeruginosa*	Strain UCBPP-PA14	Q02ML9	5UZ9, 6B44, 6B45, 6B47, 6B48, 6NE0	17038190; 21398535; 21081758; 21536913; 22522703; 28340349; 28985564; 28985564; 28985564; 28985564; 30872121	Not Available	Immune system / RNA
24	CRISPR-associated protein Csy2	*Pseudomonas aeruginosa*	Strain UCBPP-PA14	Q02MM0	5UZ9, 6B44, 6B45, 6B47, 6B48, 6NE0	17038190; 21398535; 21081758; 21536913; 22522703; 28340349;28985564;28985564;28985564; 28985564; 30872121	Not Available	Immune system / RNA
25	CRISPR-associated protein Csy3	*Pseudomonas aeruginosa*	Strain UCBPP-PA14	Q02MM1	5UZ9, 5XLO, 5XLP, 6B44, 6B45, 6B46, 6B47, 6B48, 6NE0	17038190; 21398535; 21081758; 21536913; 22522703; 28340349; 28574055; 28574055; 28985564; 28985564; 28985564; 28985564; 28985564; 30872121	Not Available	Immune system / RNA
26	Major biofilm matrix component (Spore coat-associated protein N) (Translocation-dependent antimicrobial spore component)	*Bacillus subtilis*	Strain 168	P54507	5OF1	8969508; 9384377; 3096962; 10368135; 10658653; 10049401; 10464223; 12429099; 16430695; 16430696; 20080671; 23646920; 29531041	Identical protein binding	Protein fibril
27	Minor fimbrium anchoring subunit Mfa2 (Minor fimbrial antigen 2)	*Porphyromonas gingivalis*	Strain ATCC 33277 / DSM 20709 / CIP 103683 / JCM 12257 / NCTC 11834 / 2561	B2RHG2	5NFI	18524787; 19589838; 26437277; 27062925; 29379120	Not Available	Cell adhesion
28	Minor fimbrium tip subunit Mfa3	*Porphyromonas gingivalis*	Strain ATCC 33277 / DSM 20709 / CIP 103683 / JCM 12257 / NCTC 11834 / 2561	B2RHG3	5NF4	18524787; 19589838; 24118823; 26001707; 26437277; 29379120	Not Available	Cell adhesion
29	Minor fimbrium subunit Mfa1 (Pg-II fim a)	*Porphyromonas gingivalis*	Strain ATCC 33277 / DSM 20709 / CIP 103683 / JCM 12257 / NCTC 11834 / 2561	B2RHG1	5NF2, 5NF3	18524787; 9786913; 11083792; 12593606; 15972485; 19589838; 23809984; 24118823; 26001707; 26437277; 29379120; 29379120	Not Available	Cell adhesion
30	Methyl-accepting chemotaxis protein PctA	*Pseudomonas aeruginosa*	Strain ATCC 15692 / DSM 22644 / CIP 104116 / JCM 14847 / LMG 12228 / 1C / PRS 101 / PAO1	G3XD24	5LTX, 5T65, 5T7M	8522505; 9353923; 10984043; 16233808; 23650915; 27031335; 30425146; 33097715; 31964737	Amino acid binding, transmembrane signaling receptor activity	Signaling protein
31	Regulatory protein RsaL (RsaL protein) (Virulence gene repressor RsaL)	*Pseudomonas aeruginosa*		Q9X7H4	5J2Y	1902216; 10094696	DNA binding	Gene regulation
32	Sensor protein kinase WalK (EC 2.7.13.3)	*Staphylococcus aureus*	Strain NCTC 8325 / PS 47	Q2G2U4	5IS1	10368139; 14762013; 17827301; 22825451; 27389096	ATP binding, metal ion binding, phosphorelay response regulator activity, phosphorelay sensor kinase activity, protein kinase activator activity	Transferase
33	Protein FimG	*Escherichia coli*	Strain K12	P08190	5IQO	2890081; 7610040; 9278503; 16738553	Not Available	Cell adhesion
34	2-heptyl-4(1H)-quinolone synthase subunit PqsB	*Pseudomonas aeruginosa*	Strain ATCC 15692 / DSM 22644 / CIP 104116 / JCM 14847 / LMG 12228 / 1C / PRS 101 / PAO1	Q9I4X2	5DWZ	10984043; 24239007; 26811339	AcylTransferase activity	Transferase
35	2-heptyl-4(1H)-quinolone synthase subunit PqsC (EC 2.3.1.230)	*Pseudomonas aeruginosa*	Strain ATCC 15692 / DSM 22644 / CIP 104116 / JCM 14847 / LMG 12228 / 1C / PRS 101 / PAO1	Q9I4X1	5DWZ	10984043; 24239007; 26811339	3-oxoacyl-[acyl-carrier-protein] synthase activity, beta-ketoacyl-acyl-carrier-protein synthase III activity	Transferase

Target ID	Bacterial Protein Name	Organism Name	Strain	UniProt ID	PDB ID	PubMed ID	Classification as per UniProt GO Molecular Function	Classification of Function as per PDB
36	Minor fimbrium tip subunit MfA4 (Immunoreactive 32 kDa antigen)	*Porphyromonas gingivalis*	Strain ATCC 33277 / DSM 20709 / CIP 103683 / JCM 12257 / NCTC 11834 / 2561	B2RHG4	5DHM	18524787; 19589838; 24118823; 26001707; 26437277; 26972441	Not Available	Cell adhesion
37	Fimbrial subunit CupB6	*Pseudomonas aeruginosa*	Strain ATCC 15692 / DSM 22644 / CIP 104116 / JCM 14847 / LMG 12228 / 1C / PRS 101 / PAO1	Q9HWU7	5CYL	10984043; 27481165	Not Available	Cell adhesion
38	CRISPR-associated nuclease / helicase Cas3 subtype I-F / YPEST (EC 3.1.-.-) (EC 3.6.4.-)	*Pseudomonas aeruginosa*	Strain UCBPP-PA14	Q02ML8	5B7I, 5GQH	17038190; 18952788; 21398535; 21081758; 27455460; 27585537	ATP binding, helicase activity, Hydrolase activity, Metal ion binding	Hydrolase
39	Diguanylate cyclase DosC (DGC) (EC 2.7.7.65) (Direct oxygen-sensing cyclase)	*Escherichia coli*	Strain K12	P0AA89	4ZVA	9097039; 9278503; 16738553; 16418169; 17010156; 19764732; 19332833; 21067162; 20303158; 20553324; 20576684; 22005448; 26148715	Carbon monoxide binding, Diguanylate cyclase activity, GTP binding, heme binding, Metal ion binding, oxygen binding	Signaling Protein
40	PslG	*Pseudomonas aeruginosa*	Strain ATCC 15692 / DSM 22644 / CIP 104116 / JCM 14847 / LMG 12228 / 1C / PRS 101 / PAO1	Q9I1N2	4ZN2	10984043; 26611635; 26424791	Hydrolase activity, hydrolyzing O-glycosyl compounds	Hydrolase
41	GGDEF domain-containing protein	*Pseudomonas aeruginosa*	Strain ATCC 15692 / DSM 22644 / CIP 104116 / JCM 14847 / LMG 12228 / 1C / PRS 101 / PAO1	Q9HW69	4WXO	10984043	Diguanylate cyclase activity, Metal ion binding	Transferase
42	Transcriptional regulator FleQ	*Pseudomonas aeruginosa*	Strain ATCC 15692 / DSM 22644 / CIP 104116 / JCM 14847 / LMG 12228 / 1C / PRS 101 / PAO1	G3XCV0	4WXM	10984043; 9287015; 11673434; 22581773; 26362077; 26712005	ATP binding, cis-regulatory region Sequence-specific DNA binding, cyclic-di-GMP binding, DNA-binding Transcription activator activity, DNA-binding Transcription repressor activity, Transcription cis-regulatory region binding	Transcriptional regulator
43	Surface protein G	*Staphylococcus aureus*	Strain NCTC 8325 / PS 47	Q2G2B2	4WVE	11830639; 12634333; 14523109; 17660408	Not Available	Structural protein
44	Fimbrial protein (Pilin)	*Neisseria meningitidis serogroup B*	Strain MC58	P05431	4V1J	3141743; 10710307; 413571; 8645220; 7934852; 8993351; 9515697; 17121595; 27698424	Not Available	Cell adhesion
45	Transcriptional regulatory protein CpxR	*Escherichia coli*	Strain K12	P0AE88	4UHJ, 4UHK, 4UHS, 4UHT	8294007; 8346018; 9278503; 16738553; 3058985; 7883164; 9351822; 9401031; 9473036; 10972835; 11830644; 16166523; 17259177; 23416055; 25207645; 28552574; 28552574; 30086390; 30086390	DNA-binding Transcription activator activity, DNA-binding Transcription factor activity, phosphorelay response regulator activity, Transcription cis-regulatory region binding	Transcription
46	PgaB	*Aggregatibacter actinomycetemcomitans*	Actinobacillus actinomycetemcomitans, Haemophilus actinomycetemcomitans	A5HJW8	4U10	17851029	Hydrolase activity, acting on carbon-nitrogen (but not peptide) bonds, Metal ion binding	Hydrolase
47	Outer membrane porin F	*Pseudomonas aeruginosa*	Strain ATCC 15692 / DSM 22644 / CIP 104116 / JCM 14847 / LMG 12228 / 1C / PRS 101 / PAO1	P13794	4RLC, 5U1H	2447060; 10984043; 25846137; 25846137	Calcium ion binding, complement component C3b binding, porin activity	Peptide binding protein
48	Minor fimbrium tip subunit MfA4 (Immunoreactive 32 kDa antigen PG49)	*Porphyromonas gingivalis*	Strain ATCC BAA-308 / W83	Q7MXK0	4RDB	12949112; 27062925; 27062925	Not Available	Structural protein
49	Fimbrium subunit Fim1C	*Phocaeicola vulgatus*	Strain ATCC 8482 / DSM 1447 / JCM 5826 / CCUG 4940 / NBRC 14291 / NCTC 11154 BacteroideS vulgatuS	A6L3B5	4QB7	17579514; 27062925; 27062925	Not Available	Cell adhesion
50	Major fimbrium subunit FimA type-4 (Fimbrillin) (Fimbrilin) (Major fimbrial subunit protein type-4) (FimA4)	*Porphyromonas gingivalis*	Strain ATCC BAA-308 / W83	P59914	4Q98	12949112; 23809984; 27062925; 27062925	Structural molecule	Cell adhesion

Target ID	Bacterial Protein Name	Organism Name	Strain	UniProt ID	PDB ID	PubMed ID	Classification as per UniProt GO Molecular Function	Classification of Function as per PDB
51	Adhesin / invasin TibA autotransporter [Cleaved into: Adhesin / invasin TibA (Glycoprotein TibA); Adhesin / invasin TibA translocator]	*Escherichia coli O78:H11*	Strain H10407 / ETEC	Q9XD84	4Q1Q	10417177; 20802035; 8039917; 11119488; 15784535; 25211077	Not Available	Cell adhesion
52	Phosphate-binding protein PstS	*Porphyromonas gingivalis*	Strain ATCC 15692 / DSM 22644 / CIP 104116 / JCM 14847 / LMG 12228 / 1C / PRS 101 / PAO1	G3XDA8	4OMB	16232467; 10984043; 12596240; 18282104; 6436026; 16232935; 24023943; 25005086	Phosphate ion binding	Transport protein
53	AIDA-I autotransporter (AIDA) [Cleaved into: Adhesin AIDA-I; AIDA-I translocator (AIDAc)]	*Escherichia coli*		Q03155	4MEE	1625582; 15950405; 8899706; 10559167; 15547278	Not Available	Protein binding
54	Serine-rich adhesin for platelets (Adhesin SraP) (Staphylococcus aureus surface protein A) (SasA)	*Staphylococcus aureus*	Strain NCTC 8325 / PS 47	Q2FUW1	4M00, 4M01, 4M02, 4M03	12634333; 15784571; 11830639; 18800056; 18621893; 19202081; 20472795; 20714350; 24901708; 24901708; 24901708; 24901708; 24901708	Calcium ion binding	Calcium binding protein
55	Putative fimbrium subunit Fim1C	*Bacteroides uniformis*	Strain ATCC 8492 / DSM 6597 / CCUG 4942 / CIP 103695 / JCM 5828 / KCTC 5204 / NCTC 13054 / VPI 0061	A7UZ95	4K4K	2.71E+15	Not Available	Cell adhesion
56	CapA (Capsular polysaccharide biosynthesis protein CapA) (Capsular polysaccharide type 8 biosynthesis protein cap8A) (Capsule biosynthesis protein CapA) (Chain length regulator (Capsular polysaccharide biosynthesis)) (Membrane protein CapB2)	*Staphylococcus aureus*		Q7BSA3	4JMP	10496925; 16565080; 24146800; 26048971; 3305292524146800	Not Available	Transferase
57	Putative fimbrium tip subunit Fim1C	*Parabacteroides distasonis*	Strain ATCC 8503 / DSM 20701 / CIP 104284 / JCM 5825 / NCTC 11152	A6LHQ6	4JG5	17579514; 26972441; 27062925; 27062925	Not Available	Cell adhesion
58	Sensor protein VraS (EC 2.7.13.3)	*Staphylococcus aureus*	Strain Mu50 / ATCC 700699	Q7A2Q0	4GT8	11418146; 10708580	ATP binding, phosphorelay sensor kinase activity, protein dimerization activity	Transferase
59	Exodeoxyribonuclease III (EC 3.1.11.2)	*Pseudomonas aeruginosa*		Q51380	4F1R	8824606; 23281037; 23717639; 30758287	Exodeoxyribonuclease III activity	Transcriptional regulator
60	Putative fimbrium tip subunit Fim1C	*Bacteroides ovatus*	Strain ATCC 8483 / DSM 1896 / JCM 5824 / BCRC 10623 / CCUG 4943 / NCTC 11153	A7M4E1	4EPS	27062925;	Not Available	Structural protein
61	Sensor histidine kinase CpxA (EC 2.7.13.3)	*Escherichia coli*	Strain K12	P0AE82	4BIU	3007473; 3058985; 8346018; 9278503; 16738553; 2185221; 8432716; 7883164; 9351822; 9401031; 9473036; 10972835; 11830644; 15919996; 16166523; 17259177; 20005847; 21317318; 21239493; 25207645; 24492262	ATP binding, Identical protein binding, phosphoprotein phosphatase activity, phosphorelay sensor kinase activity	Transferase
62	Biofilm-surface layer protein A (ORF-1)	*Bacillus subtilis*	Strain 168	P71014	4BHU	9384377; 8752328; 18978066; 21742882; 22571672; 28701036; 23904481; 26324938	Not Available	Structural protein
63	signaltransduction protein TRAP (Target of RNAIII-activating protein)	*Staphylococcus aureus*	Strain NCTC 8325 / PS 47	Q2G2F3	4AE5	11160124; 14726534; 16177293	Not Available	Signaling protein
64	Pneumococcal serine-rich repeat protein (PsrP) (Adhesin PsrP) (Serine-rich repeat protein PsrP)	*Streptococcus pneumoniae serotype 4*	Strain ATCC BAA-334 / TIGR4	A0A0H2URK1	3ZGH	11463916; 16861665; 18507531; 19202081; 19627498; 20714350; 24936067; 28456649; 28246170; 24430336; 27582320	DNA binding	Structural protein

Target ID	Bacterial Protein Name	Organism Name	Strain	UniProt ID	PDB ID	PubMed ID	Classification as per UniProt GO Molecular Function	Classification of Function as per PDB
65	Trimeric autotransporter adhesin AtaA (TAA AtaA) (Type 5 secretion system autotransporter AtaA)	*Acinetobacter spp.*	Strain Tol 5	K7ZP88	3WPA	23155410; 17090933; 23893702; 27074146; 27305955; 28720107; 31653547; 31972092; 26698633	Not Available	Cell adhesion
66	Poly-beta-1, 6-N-acetyl-D-glucosamine N-deacetylase (PGA N-deacetylase) (Poly-beta-1, 6-GlcNAc N-deacetylase) (EC 3.5.1.-)	*Escherichia coli*	Strain K12	P75906	3VUS	8905232; 9278503; 16738553; 15090514; 18359807	Hydrolase activity, acting on carbon-nitrogen (but not peptide) bonds, Hydrolase activity, hydrolyzing O-glycosyl compounds	Hydrolase
67	Diguanylate cyclase DgcZ (DGC) (EC 2.7.7.65) (Zinc-sensory diguanylate cyclase)	*Escherichia coli*	Strain K12	P31129	3TVK	8383113; 9097039; 9278503; 16738553; 18713317; 19332833; 19460094; 20582742; 26148715; 23769666	Diguanylate cyclase activity, GTP binding, Identical protein binding, zinc ion binding	Transferase
68	Acyl-homoserine lactone acylase PvdQ (AHL acylase PvdQ) (Acyl-HSL acylase PvdQ) (EC 3.5.1.97) [Cleaved into: Acyl-homoserine lactone acylase PvdQ subunit alpha (Acyl-HSL acylase PvdQ subunit alpha); Acyl-homoserine lactone acylase PvdQ subunit beta (Acyl-HSL acylase PvdQ subunit beta)]	*Pseudomonas aeruginosa*	Strain ATCC 15692 / DSM 22644 / CIP 104116 / JCM 14847 / LMG 12228 / 1C / PRS 101 / PAO1	Q9I194	3SRA	10984043; 16495538; 12207696; 14532048; 12686626	ADP-dependent short-chain-acyl-CoA Hydrolase activity, Hydrolase activity, acting on carbon-nitrogen (but not peptide) bonds, in linear amides, Identical protein binding	Hydrolase
69	Fap1 adhesin (Adhesin Fap1) (Fimbriae-associated protein Fap1) (Serine-rich repeat protein Fap1)	*Streptococcus parasanguinis*		A1C3L3	3RGU	10594831; 16997950; 9632253; 20164186; 20584910	Not Available	Structural protein
70	Putative fimbrium tip subunit Fim1F	*Parabacteroides distasonis*	Strain ATCC 8503 / DSM 20701 / CIP 104284 / JCM 5825 / NCTC 11152	A6LHQ9	3R4R	17579514; 26972441; 27062925; 27062925	Not Available	Cell adhesion
71	Response regulator	*Xanthomonas campestris pv. campestris*	Strain ATCC 33913 / DSM 3586 / NCPPB 528 / LMG 568 / P 25	Q8P559	3QYY	12024217; 22120736	Diguanylate cyclase activity, nucleotide binding	Signaling protein
72	Platelet binding protein GspB (Adhesin GspB) (Serine-rich adhesin for platelets) (Serine-rich repeat protein GspB)	*Streptococcus gordonii*		Q939N5	3QC5, 3QC6, 5IUC, 6EF7, 6EF9, 6EFA	12010500; 8366515; 14729688; 15049820; 15489421; 19202081; 20714350; 21531800; 26884191; 21765814; 21765814; 21765814; 21765814	Not Available	Sugar binding protein
73	Diguanylate cyclase with PAS sensory domain (EC 2.7.7.65)	*Shewanella oneidensis*	Strain MR-1	Q8EGB0	3MFX	12368813	Diguanylate cyclase activity	Transcription
74	Probable two-component response regulator	*Pseudomonas aeruginosa*	Strain ATCC 15692 / DSM 22644 / CIP 104116 / JCM 14847 / LMG 12228 / 1C / PRS 101 / PAO1	Q9HXT9	3I5C	10984043; 18366254; 19695263	Diguanylate cyclase activity, Identical protein binding, nucleotide binding	Signaling protein
75	mRNA interferase toxin MqsR (EC 3.1.-.-) (Endoribonuclease MqsR) (Motility quorum-sensing regulator MqsR) (Toxin MqsR)	*Escherichia coli*	Strain K12	Q46865	3HI2	9278503; 16738553; 16768798; 16352847; 19690171; 19909729; 20105222; 19943910; 21516113; 21788497; 29531044; 23432955; 23289863; 23172222; 25534751; 20041169	Endoribonuclease activity	DNA binding protein / toxin
76	Antitoxin MqsA	*Escherichia coli*	Strain K12	Q46864	3HI2	9278503; 16738553; 16768798; 19690171; 20105222; 19943910; 21516113; 23432955; 23172222; 24212724; 25534751; 20823526; 20041169; 21068382; 22789559	Metal ion binding, Sequence-specific DNA binding	DNA binding protein / toxin
77	Anthraniloyl-CoA anthraniloylTransferase (EC 2.3.1.262) (2-heptyl-4(1H)-quinolone synthase PqsD) (PqsD)	*Pseudomonas aeruginosa*	Strain ATCC 15692 / DSM 22644 / CIP 104116 / JCM 14847 / LMG 12228 / 1C / PRS 101 / PAO1	P20582	3H76, 3H77, 3H78	10984043; 2153661; 18728009; 21425231; 22992202; 24239007; 19694421; 19694421; 19694421;19694421	3-oxoacyl-[acyl-carrier-protein] synthase activity, acylTransferase activity	Transferase
78	Diguanylate cyclase	*Geobacter sulfurreducens*	Strain ATCC 51573 / DSM 12127 / PCA	Q74FR4	3EZU	14671304	Diguanylate cyclase activity	Signaling protein

Target ID	Bacterial Protein Name	Organism Name	Strain	UniProt ID	PDB ID	PubMed ID	Classification as per UniProt GO Molecular Function	Classification of Function as per PDB
79	Fucose-binding lectin PA-IIL	*Pseudomonas aeruginosa*	Strain ATCC 15692 / DSM 22644 / CIP 104116 / JCM 14847 / LMG 12228 / 1C / PRS 101 / PAO1	Q9HYN5	3DCQ	10984043; 12415289; 12909014; 15790314; 15573375; 16438968; 17540045; 17623286; 19101469; 23719508; 27308201; 26898721; 29896342; 29147502; 29163899; 30830745; 31146126	Carbohydrate binding, Metal ion binding	Sugar binding protein
80	Pilin (Lancefield T antigen) (Pilus backbone Structural protein)	*Streptococcus pyogenes serotype M1*		Q9A1S2	3B2M, 3GLD, 3GLE	11296296; 16088826; 17501921; 18063798; 18063798	Not Available	Structural protein
81	Biofilm operon icaADBC HTH-type negative Transcriptional regulator IcaR (Intercellular adhesion protein R)	*Staphylococcus epidermidis*	Strain ATCC 35984 / RP62A	Q5HKQ1	2ZCM	8809760; 15774886; 12142410; 18208836	DNA binding	Transcription
82	Lipoprotein NlpE (Copper homeostasis protein CutF)	*Escherichia coli*	Strain K12	P40710	2Z4H, 2Z4I	7635808; 7635807; 9278503; 16738553; 11830644; 15252048; 17698001; 17698001; 17698001	Identical protein binding	Signaling protein activator
83	CRISPR-associated endonuclease Cas6 / Csy4 (EC 3.1.-.-)	*Pseudomonas aeruginosa*	Strain UCBPP-PA14	Q02MM2	2XLI, 2XLJ, 2XLK, 4AL5, 4AL6, 4AL7, 5UZ9, 6B44, 6B45, 6B46, 6B47, 6B48, 6NE0	17038190; 21398535; 21081758; 21536913; 20829488; 22522703; 20829488; 20829488; 20829488; 22522703; 22522703; 22522703; 28340349; 28985564; 28985564; 28985564; 28985564; 28985564; 30872121	Endonuclease activity, RNA binding	Immune system / RNA
84	Autotransporter adhesin SadA (Salmonella adhesin A) (Type 5 secretion system autotransporter SadA)	*Salmonella typhimurium*	Strain LT2 / SGSC1412 / ATCC 700720	Q8ZL64	2WPQ, 2WPR, 2WPS, 2YNY, 2YNZ, 2YO0, 2YO1, 2YO2, 2YO3, 3ZMF	11677609; 21859856; 24369174; 18093992; 19805097; 23213248; 19805097; 19805097;19805097; 23213248; 23213248; 23213248; 23213248; 23213248; 23213248;18093992	Not Available	Membrane protein
85	2-aminobenzoylacetyl-CoA thioesterase (EC 3.1.2.32)	*Pseudomonas aeruginosa*	Strain ATCC 15692 / DSM 22644 / CIP 104116 / JCM 14847 / LMG 12228 / 1C / PRS 101 / PAO1	P20581	2Q0I, 2Q0J, 2VW8, 3DH8, 5HIO, 5HIP, 5HIQ, 5HIS, 7KGW, 7KGX	2153661; 10984043; 12426334; 18776012; 25960261; 27851827; 19788310; 20419351; 27082157; 19788310; 19788310; 20419351; 19788310; 27082157; 27082157; 27082157; 27082157; 33793200; 33793200	Beta-lactamase activity, Metal ion binding	Hydrolase
86	Probable two-component-system connector protein AriR	*Escherichia coli*	Strain K12	P75993	2OXL	9278503; 16738553; 15470704; 17511876; 18174134; 19172264; 19240136; 17765265; 17765265	DNA binding	Gene regulation

Target ID	Bacterial Protein Name	Organism Name	Strain	UniProt ID	PDB ID	PubMed ID	Classification as per UniProt GO Molecular Function	Classification of Function as per PDB
87	Transcription antitermination protein RfaH	*Escherichia coli*	Strain K12	P0AFW0	2OUG, 5OND, 6C6S, 6C6T	1584020; 1379743; 9278503; 16738553; 8606157; 8951819; 9171395; 9426123; 10660066; 12007406; 11983161; 16452414; 17434131; 17434131; 29741479; 29887376; 29887376	Bacterial-type RNA polymerase core enzyme binding, DNA binding, Transcription antitermination factor activity, DNA binding, translation activator activity	Transcription
88	High-affinity zinc uptake system binding-protein ZnuA	*Bacillus subtilis*	Strain 168	O34966	2O1E	9274031; 9384377; 9811636; 12426338; 14730579; 21813502; 22882210	Metal ion binding	Structural protein
89	Curli production assembly / transport component CsgE	*Escherichia coli*	Strain K12	P0AE95	2NA4	8817489; 8905232; 9278503; 16738553	Identical protein binding	Chaperone / protein transport
90	Type-1 fimbrial protein, A chain (Type-1A pilin)	*Escherichia coli*	Strain K12	P04128	2M5G	2858471; 6147250; 7610040; 9278503; 16738553	Identical protein binding	Structural protein
91	Sensor protein QseC (EC 2.7.13.3)	*Escherichia coli*	Strain K12	P40719	2KSE, 3JZ3	9278503; 16738553; 11929534; 15919996; 16352847; 20498088; 20594156	ATP binding, phosphorelay sensor kinase activity	Transferase
92	Protein FimF	*Escherichia coli*	Strain K12	P08189	2JMR	2890081; 7610040; 9278503; 16738553; 1970114	Not Available	Cell adhesion
93	Competence stimulating peptide (Competence stimulating protein)	*Streptococcus mutans*		Q99QI5	2I2J	11208787; 16936029	Pheromone activity	Signaling protein
94	Toxin YoeB (EC 3.1.-.-) (Putative endoribonuclease YoeB) (Putative mRNA interferase Yoeb)	*Escherichia coli*	Strain K12	P69348	2A6Q, 2A6R, 2A6S, 4V8X, 6NY6	9278503; 16738553; 14672926; 15009896; 15980067; 16768798; 17170003; 18854355; 19028895; 19124462; 19400780; 21788497; 29531044; 16109374; 16109374; 16109374; 16109374; 23945936; 31501867	Endoribonuclease activity, endoribonuclease activity, producing 3'-phosphomonoesters, ribosomal small subunit binding, RNA binding	Ribosome
95	Antitoxin YefM	*Escherichia coli*	Strain K12	P69346	2A6Q	9097040; 9278503; 16738553; 6170941; 7567469; 14672926; 15009896; 15980067; 16768798; 17170003; 19028895; 19400780; 16109374; 16109374	Sequence-specific DNA binding, toxic substance binding	Toxin inhhibitor
96	Global transcriptional regulator Spx (Redox-responsive Transcription factor Spx) (Suppressor of clpP and clpX) (Spx)	*Bacillus subtilis*	Strain 168	O31602	1Z3E, 3GFK, 3IHQ, 6GHB, 6GHO	9384377; 11544224; 11703662; 12028382; 12057962; 12642660; 14597697; 15659166; 16885442; 17158660; 17434969; 17908206; 18662407; 18687074; 19074380; 22307755; 23894131; 24417481; 24942655; 29271514; 30001325; 30718304; 30830258; 31655740; 16249335; 19580872; 20084284; 30982633; 16249335; 19580872; 20084284; 30982633; 30982633	Not Available	Protein binding

Target ID	Bacterial Protein Name	Organism Name	Strain	UniProt ID	PDB ID	PubMed ID	Classification as per UniProt GO Molecular Function	Classification of Function as per PDB
97	Carbon storage regulator (Translational dual regulator CsrA)	*Escherichia coli*	Strain K12	P69913	1Y00, 5Z38	8393005; 8604133; 9205837; 9278503; 16738553; 7751274; 7493933; 9211896; 11298291; 11741870; 12193630; 12067347; 12694612; 16352847; 16923806; 18223068; 18713317; 19103924; 19460094; 21488981; 21696456; 28851853; 28924029; 25833324; 15866937; 15866937; 29567971	mRNA 5'-UTR binding	RNA binding protein
98	Translational regulator CsrA (Carbon storage regulator) (Global Translational regulatory protein RsmA)	*Pseudomonas aeruginosa*	Strain ATCC 15692 / DSM 22644 / CIP 104116 / JCM 14847 / LMG 12228 / 1C / PRS 101 / PAO1	O69078	1VPZ	11673439; 10984043; 15126453; 16359708; 16104018	mRNA 5'-UTR binding	RNA binding protein
99	Endoribonuclease toxin MazF (EC 3.1.27.-) (Toxin MazF) (mRNA interferase MazF)	*Escherichia coli*	Strain K12	P0AE70	1UB4, 3NFC, 5CK9, 5CKB, 5CKD, 5CKE, 5CKF, 5CKH, 5CO7, 5CQX, 5CQY, 5CR2	2844820; 8226627; 9278503; 16738553; 8650219; 11071896; 11222603; 12123459; 12972253; 15150257; 15576778; 15316771; 15537630; 15837428; 16390452; 17962566; 18310334; 18854355; 19251848; 19707553; 21944167; 21419338; 21788497; 29531044; 20005847; 22412352; 23432955; 23416055; 23280569; 24375411; 25564525; 19215780; 26395283; 12718874; 16413577; 12718874; 27026704; 27026704; 27026704; 27026704; 27026704; 27026704; 27026704; 27026704; 27026704; 27026704	DNA binding, endoribonuclease activity, protein homodimerization activity, protein-containing complex binding, RNA binding	Hydrolase
100	O-acetyl-ADP-ribose deacetylase (EC 3.1.1.106) (Regulator of RNAse III activity)	*Escherichia coli*	Strain K12	P0A8D6	1SPV	8905232; 9278503; 16738553; 19141481; 21257746; 24267348; 25546632; 28034758; 28582517; 26481419	Deacetylase activity, endoribonuclease inhibitor activity, enzyme binding, O-acetyl-ADP-ribose deacetylase activity, purine nucleoside binding	Structural protein
101	Surface composition regulator	*Escherichia coli*	Strain K12	P26649	1RRZ	1324388; 9205837; 9278503; 16738553; 23537328; 9208918; 15161493	Not Available	Structural protein
102	Type IV major pilin protein PilA (Pilin)	*Pseudomonas aeruginosa*	Strain ATCC 15692 / DSM 22644 / CIP 104116 / JCM 14847 / LMG 12228 / 1C / PRS 101 / PAO1	P04739	1PAO	2997119; 10984043; 8330261; 8845350; 1429457; 9282737; 23266901; 26041805; 27162347; 31431558	Not Available	Fimbrial protein
103	AI-2 transport protein TqsA (Transport of quorum-sensing signal protein)	*Escherichia coli*	Strain K12	P0AFS5	--	9097039; 9278503; 16738553; 15919996; 16385049	Efflux transmembrane transporter activity	Not Available
104	HTH-type quorum sensing-dependent Transcriptional regulator VjbR	*Brucella melitensis biotype 1*	Strain 16M / ATCC 23456 / NCTC 10094	Q8YAY5	--	11756688; 16008582; 17056750; 17557825; 20529360	DNA binding	Not Available
105	Sensory / regulatory protein RpfC (EC 2.7.13.3)	*Xanthomonas campestris pv. campestris*	Strain 8004	P0C0F7	--	11123673; 15899963; 1645442; 12960398; 16611728; 28784774; 16940295; 20231439; 28369120	ATP binding, phosphorelay sensor kinase activity	Not Available
106	Exopolyphosphatase (ExopolyPase) (EC 3.6.1.11) (Polyphosphate:ADP phosphoTransferase) (PolyP:ADP phosphoTransferase) (EC 2.7.4.1)	*Pseudomonas aeruginosa*	Strain ATCC 15692 / DSM 22644 / CIP 104116 / JCM 14847 / LMG 12228 / 1C / PRS 101 / PAO1	Q9ZN70	--	10382967; 10984043; 26576296	Exopolyphosphatase activity, polyphosphate kinase activity, pyrophosphatase activity	Not Available

Target ID	Bacterial Protein Name	Organism Name	Strain	UniProt ID	PDB ID	PubMed ID	Classification as per UniProt GO Molecular Function	Classification of Function as per PDB
107	Two-component response regulator PprB	*Pseudomonas aeruginosa*	Strain ATCC 15692 / DSM 22644 / CIP 104116 / JCM 14847 / LMG 12228 / 1C / PRS 101 / PAO1	Q9HWA4	--	10984043; 12499175; 15882421; 18684246; 19151143; 21091863; 23209420; 31492668	DNA binding, phosphorelay response regulator activity	Not Available
108	Platelet adherence protein A (Adhesin PadA)	*Streptococcus gordonii*	Strain Challis / ATCC 35105 / BCRC 15272 / CH1 / DL1 / V288	A8AZP4	--	17720781; 8366515; 19884334; 21071690; 24136582; 27616700	Not Available	Not Available
109	S-ribosylhomocysteine lyase	*Listeria monocytogenes serovar 1 / 2a*	Strain ATCC BAA-679 / EGD-e	Q8Y7I9	--	12039883; 11679669	Iron ion binding, S-ribosylhomocysteine lyase activity	Not Available
110	Adhesin Ata autotransporter (Acinetobacter trimeric autotransporter) (Type 5 secretion system autotransporter Ata)	*Acinetobacter baumannii*	Strain ATCC 17978 / CIP 53.77 / LMG 1025 / NCDC KC755 / 5377	A3M3H0	--	17344419; 22825448; 22609912	Not Available	Not Available
111	Uncharacterized protein	*Desulfitobacterium hafniense*	Strain Y51	Q24NU6	--	16513756	Not Available	Not Available
112	Poly-beta-1, 6-N-acetyl-D-glucosamine N-deacetylase PgaB	*Pseudomonas spp. GM25*		J2X9J6	--	23045501	Hydrolase activity, acting on carbon-nitrogen (but not peptide) bonds	Not Available
113	Uncharacterized protein	*Limosilactobacillus mucosae LM1*		A0A0D4CKI7	--	22887668	Not Available	Not Available
114	Poly-beta-1, 6-N-acetyl-D-glucosamine synthase (Poly-beta-1, 6-Glcc synthase) (EC 2.4.1.-)	*Bordetella pertussis*	Strain Tohama I / ATCC BAA-589 / NCTC 13251	Q7VX65	--	12910271	Acetyl glucosaminyl transferase activity	Not Available
115	Uncharacterized protein	*Bordetella pertussis*	Strain Tohama I / ATCC BAA-589 / NCTC 13251	Q7VY41	--	12910271	Not Available	Not Available
116	Motility quorum-sensing regulator MqsR	*Pseudomonas spp. S35*		A0A6I6VNC7	--	25398872	Not Available	Not Available
117	Type II toxin-antitoxin system MqsR family toxin	*Pseudomonas caspiana*		A0A502JPJ4	--	31206918	Not Available	Not Available
118	Putative hemin storage protein	*Bordetella pertussis*	Strain Tohama I / ATCC BAA-589 / NCTC 13251	Q7VX64	--	12910271	Hydrolase activity, acting on carbon-nitrogen (but not peptide) bonds	Not Available
119	Surface composition regulator	*Klebsiella oxytoca*	Strain ATCC 8724 / DSM 4798 / JCM 20051 / NBRC 3318 / NRRL B-199 / KCTC 1686	A0A0H3H1S4	--	22493189	Not Available	Not Available
120	Uncharacterized protein	*Serpentinomona mccroryi*		A0A060NNA1	--	24845058	Not Available	Not Available
121	Poly-beta-1, 6-N-acetyl-D-glucosamine synthase (Poly-beta-1, 6-GlcNAc synthase) (EC 2.4.1.-)	*Cupriavidus gilardii CR3*		A0A0M4KYJ3	--	26301592	Acetyl glucosaminyl transferase activity	Not Available
122	Poly-beta-1, 6-N-acetyl-D-glucosamine biosynthesis protein PgaD	*Pseudomonas viciae*		A0A4P7PA43	--	30941113	Not Available	Not Available
123	Poly-beta-1, 6-N-acetyl-D-glucosamine biosynthesis protein PgaD	*Sedimenticola spp.*		A0A7C1YJ10	--	31911466	Not Available	Not Available
124	Poly-beta-1, 6-N-acetyl-D-glucosamine synthase (Poly-beta-1, 6-GlcNAc synthase) (EC 2.4.1.-)	*Pseudomonas viciae*		A0A4P7PA38	--	30941113	Acetyl glucosaminyl transferase activity	Not Available
125	Type II toxin-antitoxin system MqsR family toxin	*Pseudomonas gessardii*		A0A7Y1QPP7	--	32587583	Not Available	Not Available
126	Poly-beta-1, 6-N-acetyl-D-glucosamine biosynthesis protein PgaD	*Pseudomonas gessardii*		A0A7Y1MQN0	--	32587583	Not Available	Not Available
127	Poly-beta-1, 6-N-acetyl-D-glucosamine synthase (Poly-beta-1, 6-GlcNAc synthase) (EC 2.4.1.-)	*Sedimenticola spp.*		A0A7C1YCS2	--	31911466	Acetyl glucosaminyl transferase activity	Not Available
128	Poly-beta-1, 6-N-acetyl-D-glucosamine N-deacetylase PgaB (EC 3.5.1.-)	*Pseudomonas gessardii*		A0A7Y1MR48	--	32587583	Hydrolase activity, acting on carbon-nitrogen (but not peptide) bonds	Not Available
129	Uncharacterized protein	*Cupriavidus gilardii CR3*		A0A0M3SW47	--	26301592	Not Available	Not Available
130	Poly-beta-1, 6-N-acetyl-D-glucosamine N-deacetylase PgaB	*Sedimenticola spp.*		A0A7C1YIT8	--	31911466	Hydrolase activity, acting on carbon-nitrogen (but not peptide) bonds	Not Available
131	Poly-beta-1, 6-N-acetyl-D-glucosamine biosynthesis protein PgaD	*Candidatus Desulfofervidus auxilii*		A0A7C0Y839	--	31911466	Not Available	Not Available
132	Putative membrane protein	*Bordetella bronchiseptica 253*		A0A0C6P5Z7	--	23051057	Not Available	Not Available
133	Surface composition regulator	*Klebsiella pneumoniae subspp. pneumoniae*	Strain HS11286	A0A0H3GYK7	--	22408243	Not Available	Not Available
134	Hemin storage system HmsS protein	*Klebsiella pneumoniae subspp. pneumoniae*	Strain HS11286	A0A0H3GQE1	--	22408243	Not Available	Not Available
135	Poly-beta-1, 6-N-acetyl-D-glucosamine synthase (Poly-beta-1, 6-GlcNAc synthase) (EC 2.4.1.-)	*Klebsiella pneumoniae subspp. pneumoniae*	Strain HS11286	A0A0H3GH44	--	22408243	Acetyl glucosaminyl transferase activity	Not Available
136	Uncharacterized protein	*Collinsella aerofaciens*		A0A6N9JJB5	--	31477907	Not Available	Not Available
137	Motility quorum-sensing regulator	*Escherichia coli O17:K52:H18*	Strain UMN026 / ExPEC	B7ND13	--	19165319	Not Available	Not Available
138	Poly-beta-1, 6-N-acetyl-D-glucosamine synthase (Poly-beta-1, 6-GlcNAc synthase) (EC 2.4.1.-)	*Pseudomonas spp. CFBP13528*		A0A4U3IFB2	--	31040301	Acetyl glucosaminyl transferase activity	Not Available
139	Outer membrane N-deacetylase	*Pseudomonas spp.*	Strain M1	W5IKV6	--	10388678; 23405299	Hydrolase activity, acting on carbon-nitrogen (but not peptide) bonds	Not Available
140	Polysaccharide deacetylase	*Burkholderia spp. TJI49*		F0FYV7	--	23653265	Hydrolase activity, acting on carbon-nitrogen (but not peptide) bonds	Not Available
141	mRNA interferase MqsR	*Pseudomonas spp.*	Strain M1	W5IYY9	--	10388678; 23405299	Not Available	Not Available
142	Poly-beta-1, 6-N-acetyl-D-glucosamine N-deacetylase PgaB	*Stenotrophomonas spp. ZAC14D1_nalMI4_6*		A0A3Q8FCT2	--	29765358	Hydrolase activity, acting on carbon-nitrogen (but not peptide) bonds	Not Available
143	Poly-beta-1, 6-N-acetyl-D-glucosamine synthase (Poly-beta-1, 6-GlcNAc synthase) (EC 2.4.1.-)	*Stenotrophomonas spp. ZAC14D1_nalMI4_6*		A0A3Q8F836	--	29765358	Acetyl glucosaminyl transferase activity	Not Available
144	Predicted inner membrane protein	*Escherichia coli*	Strain ATCC 9637 / CCM 2024 / DSM 1116 / LMG 11080 / NBRC 13500 / NCIMB 8666 / NRRL B-766 / W	E0IZ23	--	21208457	Not Available	Not Available
145	Poly-beta-1, 6-N-acetyl-D-glucosamine synthase (Poly-beta-1, 6-GlcNAc synthase) (EC 2.4.1.-)	*Burkholderia spp. TJI49*		F0FYV6	--	23653265	Acetyl glucosaminyl transferase activity	Not Available
146	Poly-beta-1, 6-N-acetyl-D-glucosamine biosynthesis protein PgaD	*Pseudomonas spp. CFBP13528*		A0A4U3IG94	--	31040301	Not Available	Not Available
147	Uncharacterized protein	*Burkholderia spp. TJI49*		F0FZG2	--	23653265	Not Available	Not Available
148	Uncharacterized protein	*Gordonibacter urolithinfaciens*		A0A7K0IDQ7	--	31477907	Not Available	Not Available
149	Poly-beta-1, 6-N-acetyl-D-glucosamine N-deacetylase PgaB	*Pseudomonas spp. CFBP13528*		A0A4U3IGQ5	--	31040301	Hydrolase activity, acting on carbon-nitrogen (but not peptide) bonds	Not Available

Target ID	Bacterial Protein Name	Organism Name	Strain	UniProt ID	PDB ID	PubMed ID	Classification as per UniProt GO Molecular Function	Classification of Function as per PDB
150	Uncharacterized protein	*Candidatus thiomargarita nelsonii*		A0A0A6P4A5	--	27199933	Not Available	Not Available
151	Poly-beta-1, 6-N-acetyl-D-glucosamine N-deacetylase	*Pseudomonas spp. S35*		A0A6I6VSZ2	--	25398872	Hydrolase activity, acting on carbon-nitrogen (but not peptide) bonds	Not Available
152	Lipoprotein for biofilm formation	*Bacillus pumilus*	Strain SAFR-032	A8FF48	--	17895969	Not Available	Not Available
153	Poly-beta-1, 6-N-acetyl-D-glucosamine biosynthesis protein PgaD	*Pseudomonas spp. S35*		A0A6I6VN30	--	25398872	Not Available	Not Available
154	Uncharacterized protein	*Candidatus thiomargarita nelsonii*		A0A0A6P6A5	--	27199933	Not Available	Not Available
155	Uncharacterized protein	*Candidatus thiomargarita nelsonii*		A0A0A6S368	--	27199933	Not Available	Not Available
156	Poly-beta-1, 6-N-acetyl-D-glucosamine synthase (Poly-beta-1, 6-GlcNAc synthase) (EC 2.4.1.-)	*Pseudomonas spp. S35*		A0A6I6VPE8	--	25398872	Acetyl glucosaminyl transferase activity	Not Available
157	Uncharacterized protein	*Gamma proteobacterium HTCC5015*		B5JTY1	--	20472792	Not Available	Not Available
158	Uncharacterized protein	*Bacteroidetes bacterium*		A0A7C5HKG5	--	31911466	Not Available	Not Available
159	YcdR	*Gamma proteobacterium HTCC5015*		B5JTX9	--	20472792	Hydrolase activity, acting on carbon-nitrogen (but not peptide) bonds	Not Available
160	Poly-beta-1, 6-N-acetyl-D-glucosamine synthase (Poly-beta-1, 6-GlcNAc synthase) (EC 2.4.1.-)	*Gamma proteobacterium HTCC5015*		B5JTY0	--	20472792	Acetyl glucosaminyl transferase activity	Not Available
161	Poly-beta-1, 6-N-acetyl-D-glucosamine synthase (Poly-beta-1, 6-GlcNAc synthase) (EC 2.4.1.-)	*Pseudomonas gessardii*		A0A7Y1MQR9	--	32587583	Acetyl glucosaminyl transferase activity	Not Available
162	Outer membrane N-deacetylase	*Klebsiella aerogenes*	Strain ATCC 13048 / DSM 30053 / JCM 1235 / KCTC 2190 / NBRC 13534 / NCIMB 10102 / NCTC 10006	A0A0H3FMM8	--	22493190	Hydrolase activity, acting on carbon-nitrogen (but not peptide) bonds	Not Available
163	Toxin-antitoxin system, toxin component	*Nitrosomonadales bacterium*		A0A658BNF9	--	29662147	Not Available	Not Available
164	Type II toxin-antitoxin system MqsR family toxin	*Pseudomonas viciae*		A0A4P7PLV8	--	30941113	Not Available	Not Available
165	Outer membrane N-deacetylase	*Cupriavidus gilardii CR3*		A0A0M5KLW7	--	26301592	Hydrolase activity, acting on carbon-nitrogen (but not peptide) bonds	Not Available
166	Poly-beta-1, 6-N-acetyl-D-glucosamine synthase (Poly-beta-1, 6-GlcNAc synthase) (EC 2.4.1.-)	*Rhodocyclaceae bacterium*		A0A7C6VQ62	--	32123542	Acetyl glucosaminyl transferase activity	Not Available
167	GGDEF family protein	*Vibrio cholerae serotype O1*	Strain ATCC 39315 / El Tor Inaba N16961	Q9KTK2	--	10952301	Diguanylate cyclase activity	Not Available
168	Poly-beta-1, 6-N-acetyl-D-glucosamine synthase (Poly-beta-1, 6-GlcNAc synthase) (EC 2.4.1.-)	*Xenorhabdus stockiae*		A0A2D0KWC7	--	28993611	Acetyl glucosaminyl transferase activity	Not Available
169	Uncharacterized protein	*Lachnospiraceae bacterium*		A0A3C1WTK7	--	30148503	Not Available	Not Available
170	Histidinekinase (EC 2.7.13.3)	*Vibrio cholerae serotype O1*	Strain ATCC 39315 / El Tor Inaba N16961	Q9KLP6	--	10952301	Diguanylate cyclase activity, protein histidine kinase activity	Not Available
171	GGDEF family protein	*Vibrio cholerae serotype O1*	Strain ATCC 39315 / El Tor Inaba N16961	Q9KM29	--	10952301	Diguanylate cyclase activity	Not Available
172	GGDEF family protein	*Vibrio cholerae serotype O1*	Strain ATCC 39315 / El Tor Inaba N16961	Q9KSA6	--	10952301	Diguanylate cyclase activity	Not Available
173	GGDEF family protein	*Vibrio cholerae serotype O1*	Strain ATCC 39315 / El Tor Inaba N16961	Q9KT76	--	10952301	Diguanylate cyclase activity	Not Available
174	GGDEF family protein	*Vibrio cholerae serotype O1*	Strain ATCC 39315 / El Tor Inaba N16961	Q9KMV1	--	10952301	Diguanylate cyclase activity	Not Available
175	GGDEF family protein	*Vibrio cholerae serotype O1*	Strain ATCC 39315 / El Tor Inaba N16961	Q9KRN7	--	10952301	Diguanylate cyclase activity	Not Available
176	GGDEF family protein	*Vibrio cholerae serotype O1*	Strain ATCC 39315 / El Tor Inaba N16961	Q9KSP0	--	10952301	Diguanylate cyclase activity, Metal ion binding	Not Available
177	GGDEF family protein	*Vibrio cholerae serotype O1*	Strain ATCC 39315 / El Tor Inaba N16961	Q9KS87	--	10952301	Diguanylate cyclase activity	Not Available
178	Poly-beta-1, 6-N-acetyl-D-glucosamine N-deacetylase PgaB	*Enterobacter kobei*		A0A2J0PG21	--	29088362	Hydrolase activity, acting on carbon-nitrogen (but not peptide) bonds	Not Available
179	Sensory box / GGDEF family protein	*Vibrio cholerae serotype O1*	Strain ATCC 39315 / El Tor Inaba N16961	Q9KL11	--	10952301	Diguanylate cyclase activity	Not Available
180	Sensory box / GGDEF family protein	*Vibrio cholerae serotype O1*	Strain ATCC 39315 / El Tor Inaba N16961	Q9KPJ7	--	10952301	Diguanylate cyclase activity	Not Available
181	Poly-beta-1, 6-N-acetyl-D-glucosamine biosynthesis protein PgaD	*Xenorhabdus stockiae*		A0A2D0KWD2	--	28993611	Not Available	Not Available
182	GGDEF family protein	*Vibrio cholerae serotype O1*	Strain ATCC 39315 / El Tor Inaba N16961	Q9KSS1	--	10952301	Diguanylate cyclase activity	Not Available
183	GGDEF family protein	*Vibrio cholerae serotype O1*	Strain ATCC 39315 / El Tor Inaba N16961	Q9KL98	--	10952301	Diguanylate cyclase activity	Not Available
184	GGDEF family protein	*Vibrio cholerae serotype O1*	Strain ATCC 39315 / El Tor Inaba N16961	Q9KT01	--	10952301	Diguanylate cyclase activity	Not Available
185	Poly-beta-1, 6-N-acetyl-D-glucosamine biosynthesis protein PgaD	*Lachnospiraceae bacterium*		A0A3D0DPQ1	--	30148503	Not Available	Not Available
186	GGDEF family protein	*Vibrio cholerae serotype O1*	Strain ATCC 39315 / El Tor Inaba N16961	Q9KNN4	--	10952301	Diguanylate cyclase activity	Not Available
187	GGDEF family protein	*Vibrio cholerae serotype O1*	Strain ATCC 39315 / El Tor Inaba N16961	Q9KM32	--	10952301	Diguanylate cyclase activity	Not Available
188	Poly-beta-1, 6-N-acetyl-D-glucosamine synthase (Poly-beta-1, 6-GlcNAc synthase) (EC 2.4.1.-)	*Pseudomonas spp.*		A0A3D4NT76	--	30148503	Acetyl glucosaminyl transferase activity	Not Available
189	mRNA interferase MqsR	*Pseudomonas spp.*		A0A3D0JRX8	--	30148503	Not Available	Not Available
190	Surface composition regulator	*Salmonella agona*	Strain SL483	B5F692	--	21602358	Not Available	Not Available
191	mRNA interferase MqsR	*Pseudomonas spp.*		A0A355RA73	--	30148503	Not Available	Not Available
192	Type II toxin-antitoxin system MqsR family toxin	*Pseudomonas spp.*		A0A3D1VUK6	--	30148503	Not Available	Not Available
193	Poly-beta-1, 6-N-acetyl-D-glucosamine biosynthesis protein PgaD	*Pseudomonas spp.*		A0A3D4NT61	--	30148503	Not Available	Not Available
194	Poly-beta-1, 6-N-acetyl-D-glucosamine synthase (Poly-beta-1, 6-GlcNAc synthase) (EC 2.4.1.-)	*Xenorhabdus budapestensis*		A0A2D0J1F4	--	28993611	Acetyl glucosaminyl transferase activity	Not Available
195	Poly-beta-1, 6-N-acetyl-D-glucosamine biosynthesis protein PgaD	*Priestia megaterium*	Bacillus megaterium	A0A2T7JNY8	--	31228823	Not Available	Not Available
196	Hypothetical lipoprotein ycdR	*Escherichia coli O6:H1*	Strain CFT073 / ATCC 700928 / UPEC	A0A0H2V5Y9	--	12471157	Hydrolase activity, acting on carbon-nitrogen (but not peptide) bonds	Not Available
197	Putative polysaccharide deacetylase associated with biofilm formation putative lipoprotein (PgaB)	*Ralstonia solanacearum CMR15*		D8NG92	--	20550686	Hydrolase activity, acting on carbon-nitrogen (but not peptide) bonds	Not Available
198	Type II toxin-antitoxin system MqsR family toxin	*Desulfovibrio spp.*		A0A3D4GJY3	--	30148503	Not Available	Not Available
199	Uncharacterized protein	*Serpentinomonas raichei*		A0A060NIR8	--	24845058	Not Available	Not Available
200	Putative transmembrane protein	*Ralstonia solanacearum CMR15*		D8NG94	--	20550686	Not Available	Not Available
201	Poly-beta-1, 6-N-acetyl-D-glucosamine synthase (Poly-beta-1, 6-GlcNAc synthase) (EC 2.4.1.-)	*Ralstonia solanacearum CMR15*		D8NG93	--	20550686	Acetyl glucosaminyl transferase activity	Not Available
202	Poly-beta-1, 6-N-acetyl-D-glucosamine synthase (Poly-beta-1, 6-GlcNAc synthase) (EC 2.4.1.-)	*Escherichia coli O6:H1*	Strain CFT073 / ATCC 700928 / UPEC	A0A0H2V816	--	12471157	Acetyl glucosaminyl transferase activity	Not Available

Target ID	Bacterial Protein Name	Organism Name	Strain	UniProt ID	PDB ID	PubMed ID	Classification as per UniProt GO Molecular Function	Classification of Function as per PDB
203	Putative polysaccharide deacetylase	*Klebsiella pneumoniae subspp. pneumoniae*	Strain ATCC 700721 / MGH 78578	A6TH34	--	11677609	Hydrolase activity, acting on carbon-nitrogen (but not peptide) bonds	Not Available
204	Poly-beta-1, 6-N-acetyl-D-glucosamine synthase (Poly-beta-1, 6-GlcNAc synthase) (EC 2.4.1.-)	*Klebsiella pneumoniae subspp. pneumoniae*	Strain ATCC 700721 / MGH 78578	A6TH33	--	11677609	Acetyl glucosaminyl transferase activity	Not Available
205	Poly-beta-1, 6-N-acetyl-D-glucosamine synthase (Poly-beta-1, 6-GlcNAc synthase) (EC 2.4.1.-)	*Tetragenococcus halophilus*	Pediococcus halophilus	A0A3G5FLQ7	--	21357458	Acetyl glucosaminyl transferase activity	Not Available
206	Uncharacterized protein	*Hydrogenophaga spp.*		A0A350SRA6	--	30148503	Not Available	Not Available
207	Uncharacterized protein	*Escherichia coli O6:H1*	Strain CFT073 / ATCC 700928 / UPEC	A0A0H2V5Z5	--	12471157	Not Available	Not Available
208	Surface composition regulator	*Edwardsiella ictaluri*	Strain 93-146	C5B9Z2	--	22247535	Not Available	Not Available
209	Poly-beta-1, 6-N-acetyl-D-glucosamine N-deacetylase	*Tatumella spp. OPLPL6*		A0A2G8BYL8	--	29067021	Hydrolase activity, acting on carbon-nitrogen (but not peptide) bonds	Not Available
210	GGDEF domain-containing protein	*Rhodopirellula baltica*	Strain DSM 10527 / NCIMB 13988 / SH1	Q7UJ25	--	12835416	Diguanylate cyclase activity	Not Available
211	Poly-beta-1, 6-N-acetyl-D-glucosamine synthase (Poly-beta-1, 6-GlcNAc synthase) (EC 2.4.1.-)	*Tatumella spp. OPLPL6*		A0A2G8BYM4	--	29067021	Acetyl glucosaminyl transferase activity	Not Available
212	Probable two-component response regulator	*Rhodopirellula baltica*	Strain DSM 10527 / NCIMB 13988 / SH1	Q7URT2	--	12835416	Diguanylate cyclase activity	Not Available
213	GGDEF family protein	*Rhodopirellula baltica*	Strain DSM 10527 / NCIMB 13988 / SH1	Q7UIF2	--	12835416	Diguanylate cyclase activity	Not Available
214	Uncharacterized protein	*Rhodopirellula baltica*	Strain DSM 10527 / NCIMB 13988 / SH1	Q7UWZ9	--	12835416	Diguanylate cyclase activity	Not Available
215	Poly-beta-1, 6-N-acetyl-D-glucosamine N-deacetylase PgaB	*Acinetobacter seifertii*		N8SCM4	--	25563912	Hydrolase activity, acting on carbon-nitrogen (but not peptide) bonds	Not Available
216	Surface composition regulator	*Salmonella enterica subspp. enterica serovar*	Urbana Str. R8-2977	G5S153	--	21859443	Not Available	Not Available
217	Poly-beta-1, 6-N-acetyl-D-glucosamine synthase (Poly-beta-1, 6-GlcNAc synthase) (EC 2.4.1.-)	*Mammaliicoccus fleurettii*		A0A3A0I0Q2	--	28066335	Acetyl glucosaminyl transferase activity	Not Available
218	Poly-beta-1, 6-N-acetyl-D-glucosamine synthase (Poly-beta-1, 6-GlcNAc synthase) (EC 2.4.1.-)	*Aerococcus urinaehominis*		A0A109RGK8	--	27103727	Acetyl glucosaminyl transferase activity	Not Available
219	Poly-beta-1, 6-N-acetyl-D-glucosamine synthase (Poly-beta-1, 6-GlcNAc synthase) (EC 2.4.1.-)	*Stenotrophomonas geniculata N1*		A0A0L8AEC2	--	22689240	Acetyl glucosaminyl transferase activity	Not Available
220	Uncharacterized protein	*Candidatus Fermentibacter daniensis*		A0A163WHS5	--	27058503	Not Available	Not Available
221	Hemin storage protein	*Stenotrophomonas geniculata N1*		A0A0L8AES1	--	22689240	Not Available	Not Available
222	Uncharacterized protein	*Novosphingobium nitrogenifigens DSM 19370*		F1ZCU9	--	22156397	Not Available	Not Available
223	Surface composition regulator	*Salmonella dublin*		A0A3T3IH62	--	30286803	Not Available	Not Available
224	Poly-beta-1, 6-N-acetyl-D-glucosamine N-deacetylase PgaB	*Stenotrophomonas spp. YAU14A_MKIMI4_1*		A0A7U5WKB3	--	29765358	Hydrolase activity, acting on carbon-nitrogen (but not peptide) bonds	Not Available
225	Poly-beta-1, 6-N-acetyl-D-glucosamine synthase (Poly-beta-1, 6-GlcNAc synthase) (EC 2.4.1.-)	*Pseudomonas proteolytica*		A0A7Y1GX08	--	32587583	Acetyl glucosaminyl transferase activity	Not Available
226	mRNA interferase MqsR	*Lautropia spp. SCN 69-89*		A0A1D2SFM5	--	26031303	Not Available	Not Available
227	Poly-beta-1, 6-N-acetyl-D-glucosamine biosynthesis protein PgaD	*Lautropia spp. SCN 69-89*		A0A1D2SJT2	--	26031303	Not Available	Not Available
228	Poly-beta-1, 6-N-acetyl-D-glucosamine synthase (Poly-beta-1, 6-GlcNAc synthase) (EC 2.4.1.-)	*Pseudomonas proteolytica*		A0A7Y1E338	--	32587583	Acetyl glucosaminyl transferase activity	Not Available
229	Poly-beta-1, 6-N-acetyl-D-glucosamine biosynthesis protein PgaD	*Stenotrophomonas spp. YAU14A_MKIMI4_1*		A0A7U6AE53	--	29765358	Not Available	Not Available
230	Poly-beta-1, 6-N-acetyl-D-glucosamine biosynthesis protein PgaD	*Candidatus Accumulibacter spp.*		A0A3D3NIE8	--	30148503	Not Available	Not Available
231	Surface composition regulator	*Pectobacterium peruviense*		A0A0J5XXP7	--	26251497	DNA binding, DNA-directed DNA polymerase activity	Not Available
232	CDA1 protein	*Mannheimia succiniciproducens*	Strain MBEL55E	Q65TJ0	--	15378067	Hydrolase activity, acting on carbon-nitrogen (but not peptide) bonds	Not Available
233	Poly-beta-1, 6-N-acetyl-D-glucosamine synthase (Poly-beta-1, 6-GlcNAc synthase) (EC 2.4.1.-)	*Pectobacterium peruviense*		A0A0J5XL48	--	26251497	Acetyl glucosaminyl transferase activity	Not Available
234	mRNA interferase MqsR	*Candidatus Accumulibacter spp.*		A0A3D6DTJ4	--	30148503	Not Available	Not Available
235	Poly-beta-1, 6-N-acetyl-D-glucosamine synthase (Poly-beta-1, 6-GlcNAc synthase) (EC 2.4.1.-)	*Burkholderia multivorans CGD2*		B9BJ73	--	23105085	Acetyl glucosaminyl transferase activity	Not Available
236	Hemin storage protein	*Pectobacterium peruviense*		A0A0J5XP71	--	26251497	Not Available	Not Available
237	Uncharacterized protein	*Lautropia spp. SCN 69-89*		A0A1D2SI36	--	26031303	Not Available	Not Available
238	Poly-beta-1, 6-N-acetyl-D-glucosamine N-deacetylase (EC 3.5.1.-)	*Aeromona salmonicida subspp. pectinolytica 34mel*		T0PMK3	--	29304740	Hydrolase activity, acting on carbon-nitrogen (but not peptide) bonds	Not Available
239	Type II toxin-antitoxin system MqsR family toxin	*Nitrospirae bacterium*		A0A7C0YTH1	--	31911466	Not Available	Not Available
240	Poly-beta-1, 6-N-acetyl-D-glucosamine synthase (Poly-beta-1, 6-GlcNAc synthase) (EC 2.4.1.-)	*Pseudomonas spp. GM30*		W6VHT4	--	23045501	Acetyl glucosaminyl transferase activity	Not Available
241	Poly-beta-1, 6-N-acetyl-D-glucosamine synthase (Poly-beta-1, 6-GlcNAc synthase) (EC 2.4.1.-)	*Aeromona salmonicida subspp. pectinolytica 34mel*		T0PR46	--	29304740	Acetyl glucosaminyl transferase activity	Not Available
242	Poly-beta-1, 6-N-acetyl-D-glucosamine biosynthesis protein PgaD	*Burkholderiales bacterium*		A0A3A0ELI9	--	30199629	Not Available	Not Available
243	Poly-beta-1, 6-N-acetyl-D-glucosamine N-deacetylase PgaB	*Acinetobacter baumannii*		A0A5P1UIB3	--	25136007	Hydrolase activity, acting on carbon-nitrogen (but not peptide) bonds	Not Available
244	Poly-beta-1, 6-N-acetyl-D-glucosamine synthase (Poly-beta-1, 6-GlcNAc synthase) (EC 2.4.1.-)	*Burkholderiales bacterium*		A0A3A0EKV6	--	30199629	Acetyl glucosaminyl transferase activity	Not Available
245	Poly-beta-1, 6-N-acetyl-D-glucosamine N-deacetylase PgaB	*Burkholderiales bacterium*		A0A3A0ES14	--	30199629	Hydrolase activity, acting on carbon-nitrogen (but not peptide) bonds	Not Available
246	Poly-beta-1, 6-N-acetyl-D-glucosamine N-deacetylase PgaB	*Acinetobacter baumannii*		A0A7Z2AB22	--	27917170	Hydrolase activity, acting on carbon-nitrogen (but not peptide) bonds	Not Available
247	Poly-beta-1, 6-N-acetyl-D-glucosamine synthase (Poly-beta-1, 6-GlcNAc synthase) (EC 2.4.1.-)	*Xanthomonadaceae bacterium SCN 69-320*		A0A1E4K406	--	26031303	Acetyl glucosaminyl transferase activity	Not Available
248	Poly-beta-1, 6-N-acetyl-D-glucosamine biosynthesis protein PgaD	*Acinetobacter baumannii*		A0A7H1PNT2	--	27917170	Not Available	Not Available
249	Poly-beta-1, 6-N-acetyl-D-glucosamine N-deacetylase PgaB	*Xanthomonadaceae bacterium SCN 69-320*		A0A1E4K488	--	26031303	Hydrolase activity, acting on carbon-nitrogen (but not peptide) bonds	Not Available
250	Poly-beta-1, 6-N-acetyl-D-glucosamine biosynthesis protein PgaD	*Xanthomonadaceae bacterium SCN 69-320*		A0A1E4K403	--	26031303	Not Available	Not Available
251	Surface composition regulator	*Salmonella virchow*		A0A3Y8PE59	--	30286803	Not Available	Not Available
252	S-ribosylhomocysteine lyase (EC 4.4.1.21) (AI-2 synthesis protein) (Autoinducer-2 production protein LuxS)	*Streptococcus anginosus*		Q4PLF8	--	16897563	Iron ion binding, S-ribosylhomocysteine lyase activity	Not Available
253	Surface composition regulator	*Salmonella enterica subspp. enterica serovar*	London	A0A5J1SKH1	--	30286803	Not Available	Not Available

Target ID	Bacterial Protein Name	Organism Name	Strain	UniProt ID	PDB ID	PubMed ID	Classification as per UniProt GO Molecular Function	Classification of Function as per PDB
254	DUF1190 domain-containing protein (Lipoprotein)	*Xanthomonas campestris*		A0A6N3X0V8	--	32966572	Not Available	Not Available
255	Surface composition regulator	*Salmonella enterica subspp. enterica serovar*	Stanley	A0A486WXB0	--	30286803; 31099590	Not Available	Not Available
256	Surface composition regulator	*Serratia marcescens*		A0A0G3STT8	--	28636609	Not Available	Not Available
257	Uncharacterized protein	*Pseudomonas spp.*	Strain GM41 2012	W6VMB4	--	23045501	Not Available	Not Available
258	Poly-beta-1, 6-N-acetyl-D-glucosamine N-deacetylase PgaB (EC 3.5.1.-)	*Pseudomonas proteolytica*		A0A7Y1H7P0	--	32587583	Hydrolase activity, acting on carbon-nitrogen (but not peptide) bonds	Not Available
259	Poly-beta-1, 6-N-acetyl-D-glucosamine N-deacetylase PgaB (EC 3.5.1.-)	*Pseudomonas proteolytica*		A0A7Y1DEZ8	--	32587583	Hydrolase activity, acting on carbon-nitrogen (but not peptide) bonds	Not Available
260	Poly-beta-1, 6-N-acetyl-D-glucosamine synthase (Poly-beta-1, 6-GlcNAc synthase) (EC 2.4.1.-)	*Shigella spp.*		A0A351N4J1	--	30148503	Acetyl glucosaminyl transferase activity	Not Available
261	Type II toxin-antitoxin system MqsR family toxin	*Pseudomonas spp. WS 5011*		A0A7Y1BLX1	--	32587583	Not Available	Not Available
262	Poly-beta-1, 6-N-acetyl-D-glucosamine synthase (Poly-beta-1, 6-GlcNAc synthase) (EC 2.4.1.-)	*Klebsiella pneumoniae*		A0A2L0KKC0	--	29437920	Acetyl glucosaminyl transferase activity	Not Available
263	mRNA interferase MqsR of the MqsA-MqsR antitoxin / toxin complex and DNA-binding Transcriptionalrepressor (EC 3.1.-.-)	*Pseudomonas putida*	Strain ATCC 47054 / DSM 6125 / CFBP 8728 / NCIMB 11950 / KT2440	Q88F93	--	12534463	DNA binding, Hydrolase activity	Not Available
264	Hemin storage protein	*Stenotrophomonas maltophilia MF89*		T5KKA0	--	24092793	Not Available	Not Available
265	Poly-beta-1, 6-N-acetyl-D-glucosamine biosynthesis protein PgaD	*Pseudomonas orientalis*		A0A2L0RPX3	--	29479340	Not Available	Not Available
266	Hemin storage system protein	*Yersinia entomophaga*		A0A3S6EX35	--	27187466	Not Available	Not Available
267	Uncharacterized protein	*Lentilactobacillus diolivorans DSM 14421*		A0A0R1SPG5	--	26415554	Not Available	Not Available
268	Uncharacterized protein	*Gammaproteobacteria bacterium RBG_16_51_14*		A0A1G0FW62	--	27774985	Not Available	Not Available
269	Poly-beta-1, 6-N-acetyl-D-glucosamine synthase (Poly-beta-1, 6-GlcNAc synthase) (EC 2.4.1.-)	*Burkholderia multivorans CGD1*		B9B6W1	--	23105085	Acetyl glucosaminyl transferase activity	Not Available
270	Type II toxin-antitoxin system MqsR family toxin	*Companilactobacillus salsicarnum*		A0A646JPD0	--	31668878	Not Available	Not Available
271	Outer membrane N-deacetylase	*Yersinia entomophaga*		A0A3S6EYN4	--	27187466	Hydrolase activity, acting on carbon-nitrogen (but not peptide) bonds	Not Available
272	Biofilm PGA synthesis lipoprotein PgaB	*Xanthomonas oryzae pv. oryzicola*	Strain BLS256	G7TD27	--	21784931	Hydrolase activity, acting on carbon-nitrogen (but not peptide) bonds	Not Available
273	Biofilm PGA synthesis lipoprotein PgaB (EC 3.-.-.-)	*Burkholderia multivorans CGD1*		B9B6W2	--	23105085	Hydrolase activity, acting on carbon-nitrogen (but not peptide) bonds	Not Available
274	Uncharacterized protein	*Pelagibacterium spp. SCN 64-44*		A0A1E4G1S5	--	26031303	Not Available	Not Available
275	Biofilm PGA synthesis protein PgaD	*Enterobacter cloacae subspp. cloacae*	Strain ATCC 13047 / DSM 30054 / NBRC 13535 / NCTC 10005 / WDCM 00083 / NCDC 279-56	A0A0H3CRL8	--	20207761	Not Available	Not Available
276	Putative membrane protein	*Streptomyces coelicolor*	Strain ATCC BAA-471 / A3 2 / M145	Q9AD78	--	12000953	Diguanylate cyclase activity	Not Available
277	Poly-beta-1, 6-N-acetyl-D-glucosamine N-deacetylase	*Pseudomonas orientalis*		A0A2L0RPV9	--	29479340	Hydrolase activity, acting on carbon-nitrogen (but not peptide) bonds	Not Available
278	Uncharacterized protein	*Burkholderia multivorans CGD1*		B9B078	--	23105085	Not Available	Not Available
279	Uncharacterized protein	*Levilactobacillus koreensis JCM 16448*		A0A0R1KV26	--	26415554	Not Available	Not Available
280	Poly-beta-1, 6-N-acetyl-D-glucosamine synthase (Poly-beta-1, 6-GlcNAc synthase) (EC 2.4.1.-)	*Stenotrophomonas maltophilia*	Pseudomonas maltophilia Xanthomonas maltophilia	A0A2J0SNY9	--	29170656	Acetyl glucosaminyl transferase activity	Not Available
281	Biofilm PGA synthesis lipoprotein pgaB	*Acinetobacter spp. HA*		I4ZWY1	--	22933775	Hydrolase activity, acting on carbon-nitrogen (but not peptide) bonds	Not Available
282	mRNA interferase MqsR	*Photorhabdus spp. S9-53*		A0A329WFL1	--	29877789	Not Available	Not Available
283	Poly-beta-1, 6-N-acetyl-D-glucosamine N-deacetylase PgaB	*Candidatus Manganitrophus noduliformans*		A0A7X6DPV0	--	32669693	Hydrolase activity, acting on carbon-nitrogen (but not peptide) bonds	Not Available
284	Poly-beta-1, 6-N-acetyl-D-glucosamine synthase (Poly-beta-1, 6-GlcNAc synthase) (EC 2.4.1.-)	*Schinkia azotoformans LMG 9581*		K6DNV1	--	23087684	Acetyl glucosaminyl transferase activity	Not Available
285	Poly-beta-1, 6-N-acetyl-D-glucosamine synthase (Poly-beta-1, 6-GlcNAc synthase) (EC 2.4.1.-)	*Oceanospirillales bacterium*		A0A7V2SRN9	--	31911466	Acetyl glucosaminyl transferase activity	Not Available
286	Poly-beta-1, 6-N-acetyl-D-glucosamine synthase (Poly-beta-1, 6-GlcNAc synthase) (EC 2.4.1.-)	*Candidatus Manganitrophus noduliformans*		A0A7X6DPT7	--	32669693	Acetyl glucosaminyl transferase activity	Not Available
287	Surface composition regulator	*Salmonella typhimurium*	Strain D23580	A0A6C7IEY4	--	19901036	Not Available	Not Available
288	Uncharacterized protein	*Acinetobacter baumannii TYTH-1*		A0A7U4BP68	--	23209228	Not Available	Not Available
289	Poly-beta-1, 6-N-acetyl-D-glucosamine N-deacetylase PgaB	*Enterobacter chengduensis*		A0A5C1C483	--	30302649	Hydrolase activity, acting on carbon-nitrogen (but not peptide) bonds	Not Available
290	Poly-beta-1, 6-N-acetyl-D-glucosamine synthase (Poly-beta-1, 6-GlcNAc synthase) (EC 2.4.1.-)	*Priestia megaterium*	Strain DSM 319 / IMG 1521 Bacillus megaterium	D5DKD8	--	21705586	Acetyl glucosaminyl transferase activity	Not Available
291	Poly-beta-1, 6-N-acetyl-D-glucosamine synthase (Poly-beta-1, 6-GlcNAc synthase) (EC 2.4.1.-)	*Acinetobacter baumannii TYTH-1*		A0A7U4BPK8	--	23209228	Acetyl glucosaminyl transferase activity	Not Available
292	Xylanase / chitin deacetylase	*Acinetobacter baumannii TYTH-1*		A0A7U4F894	--	23209228	Hydrolase activity, acting on carbon-nitrogen (but not peptide) bonds, Hydrolase activity, acting on glycosyl bonds	Not Available
293	Lipoprotein HmsF	*Acinetobacter baumannii TYTH-1*		A0A7U4F6K9	--	23209228	Hydrolase activity, acting on carbon-nitrogen (but not peptide) bonds	Not Available
294	Uncharacterized protein	*Priestia megaterium*	Strain DSM 319 / IMG 1521 Bacillus megaterium	D5DKD7	--	21705586	Not Available	Not Available
295	Poly-beta-1, 6-N-acetyl-D-glucosamine biosynthesis protein PgaD	*Acinetobacter baumannii TYTH-1*		A0A7U4BLE2	--	23209228	Not Available	Not Available
296	Poly-beta-1, 6-N-acetyl-D-glucosamine synthase (Poly-beta-1, 6-GlcNAc synthase) (EC 2.4.1.-)	*Acinetobacter baumannii TYTH-1*		A0A7U4BLC4	--	23209228	Acetyl glucosaminyl transferase activity	Not Available
297	Poly-beta-1, 6-N-acetyl-D-glucosamine biosynthesis protein PgaD (Fragment)	*Guyparkeria spp. SCN-R1*		A0A426QDS2	--	24673239	Not Available	Not Available
298	Poly-beta-1, 6-N-acetyl-D-glucosamine synthase (Poly-beta-1, 6-GlcNAc synthase) (EC 2.4.1.-)	*Lentisphaerae bacterium GWF2_52_8*		A0A1G0ZGS6	--	27774985	Acetyl glucosaminyl transferase activity	Not Available

Target ID	Bacterial Protein Name	Organism Name	Strain	UniProt ID	PDB ID	PubMed ID	Classification as per UniProt GO Molecular Function	Classification of Function as per PDB
299	Predicted enzyme associated with biofilm formation	*Escherichia coli*	Strain ATCC 9637 / CCM 2024 / DSM 1116 / LMG 11080 / NBRC 13500 / NCIMB 8666 / NRRL B-766 / W	E0IZ21	--	21208457	Hydrolase activity, acting on carbon-nitrogen (but not peptide) bonds	Not Available
300	Surface composition regulator	*Escherichia coli*	Strain ATCC 9637 / CCM 2024 / DSM 1116 / LMG 11080 / NBRC 13500 / NCIMB 8666 / NRRL B-766 / W	E0IVV8	--	21208457	Not Available	Not Available
301	Uncharacterized protein	*Collinsella aerofaciens*		A0A6N9JPD9	--	31477907	Not Available	Not Available
302	Poly-beta-1, 6-N-acetyl-D-glucosamine synthase (Poly-beta-1, 6-GlcNAc synthase) (EC 2.4.1.-)	*Pseudomonas spp.*	Strain M1	W5IMC6	--	10388678; 23405299	Acetyl glucosaminyl transferase activity	Not Available
303	Poly-beta-1, 6-N-acetyl-D-glucosamine synthase (Poly-beta-1, 6-GlcNAc synthase) (EC 2.4.1.-)	*Escherichia coli*	Strain ATCC 9637 / CCM 2024 / DSM 1116 / LMG 11080 / NBRC 13500 / NCIMB 8666 / NRRL B-766 / W	E0IZ22	--	21208457	Acetyl glucosaminyl transferase activity	Not Available
304	Poly-beta-1, 6-N-acetyl-D-glucosamine biosynthesis protein PgaD	*Stenotrophomonas spp. ZAC14D1_naIMI4_6*		A0A3Q8FAH5	--	29765358	Not Available	Not Available
305	Poly-beta-1, 6-N-acetyl-D-glucosamine biosynthesis protein PgaD	*Stenotrophomonas spp. ZAC14D1_naIMI4_6*		A0A3Q8F8L9	--	29765358	Not Available	Not Available
306	Poly-beta-1, 6-N-acetyl-D-glucosamine synthase (Poly-beta-1, 6-GlcNAc synthase) (EC 2.4.1.-)	*Stenotrophomonas spp. ZAC14D1_naIMI4_6*		A0A3Q8FBD5	--	29765358	Acetyl glucosaminyl transferase activity	Not Available
307	Type II toxin-antitoxin system MqsR family toxin	*Pseudomonas spp. WS 5414*		A0A7Y1CDF1	--	32587583	Not Available	Not Available
308	Uncharacterized protein	*Zetaproteobacteria bacterium CG2_30_46_52*		A0A1J5IY78	--	27112493	Not Available	Not Available
309	Poly-beta-1, 6-N-acetyl-D-glucosamine N-deacetylase	*Leclercia spp. LSNIH1*		A0A2I8NTT5	--	29437920	Hydrolase activity, acting on carbon-nitrogen (but not peptide) bonds	Not Available
310	Biofilm PGA synthesis lipoprotein PgaB (EC 3.-.-.-)	*Pseudomonas fluorescens*	Strain ATCC BAA-477 / NRRL B-23932 / Pf-5	Q4KKC4	--	15980861	Hydrolase activity, acting on carbon-nitrogen (but not peptide) bonds	Not Available
311	Poly-beta-1, 6-N-acetyl-D-glucosamine synthase (Poly-beta-1, 6-GlcNAc synthase) (EC 2.4.1.-)	*Pseudomonas fluorescens*	Strain ATCC BAA-477 / NRRL B-23932 / Pf-5	Q4KKC3	--	15980861	Acetyl glucosaminyl transferase activity	Not Available
312	Uncharacterized protein	*Yersinia pseudotuberculosis serotype I*	Strain IP32953	Q666X0	--	15358858	Not Available	Not Available
313	Uncharacterized protein	*Thiotrichales bacterium SG8_50*		A0A0S8EKY3	--	25922666	Not Available	Not Available
314	Motility quorum-sensing regulator MqsR	*Pseudomonas fluorescens*	Strain ATCC BAA-477 / NRRL B-23932 / Pf-5	Q4KHU9	--	15980861	Not Available	Not Available
315	Type II toxin-antitoxin system MqsR family toxin	*Methylosinus sporium*		A0A2U1STG6	--	29725720	Not Available	Not Available
316	Poly-beta-1, 6-N-acetyl-D-glucosamine N-deacetylase PgaB (Fragment)	*Planctomycetaceae bacterium*		A0A3B8ZFN5	--	30148503	Hydrolase activity, acting on carbon-nitrogen (but not peptide) bonds	Not Available
317	Poly-beta-1, 6-N-acetyl-D-glucosamine synthase (Poly-beta-1, 6-GlcNAc synthase) (EC 2.4.1.-)	*Yersinia pseudotuberculosis serotype I*	Strain IP32953	Q66B29	--	15358858	Acetyl glucosaminyl transferase activity	Not Available
318	Poly-beta-1, 6-N-acetyl-D-glucosamine synthase (Poly-beta-1, 6-GlcNAc synthase) (EC 2.4.1.-) (Fragment)	*Planctomycetaceae bacterium*		A0A3B8ZEJ0	--	30148503	Acetyl glucosaminyl transferase activity	Not Available
319	Poly-beta-1, 6-N-acetyl-D-glucosamine synthase (Poly-beta-1, 6-GlcNAc synthase) (EC 2.4.1.-)	*Acinetobacter baumannii*		A0A2I8CSE1	--	29437920	Acetyl glucosaminyl transferase activity	Not Available
320	Uncharacterized protein	*Xanthomonas oryzae pv. oryzae*	Strain KACC10331 / KXO85	Q5GVV4	--	15673718	Not Available	Not Available
321	Poly-beta-1, 6-N-acetyl-D-glucosamine biosynthesis protein PgaD	*Acinetobacter baumannii*		A0A7Z1WPV2	--	27917170	Not Available	Not Available
322	Poly-beta-1, 6-N-acetyl-D-glucosamine synthase (Poly-beta-1, 6-GlcNAc synthase) (EC 2.4.1.-)	*Acinetobacter baumannii*		A0A1E3M1V1	--	25136007	Acetyl glucosaminyl transferase activity	Not Available
323	Uncharacterized protein	*Novosphingobium nitrogenifigens DSM 19370*		F1ZCX2	--	22156397	Not Available	Not Available
324	HmsF protein	*Xanthomonas oryzae pv. oryzae*	Strain KACC10331 / KXO85	Q5GVV6	--	15673718	Hydrolase activity, acting on carbon-nitrogen (but not peptide) bonds	Not Available
325	Poly-beta-1, 6-N-acetyl-D-glucosamine synthase (Poly-beta-1, 6-GlcNAc synthase) (EC 2.4.1.-)	*Xanthomonas oryzae pv. oryzae*	Strain KACC10331 / KXO85	Q5GVV5	--	15673718	Acetyl glucosaminyl transferase activity	Not Available
326	Poly-beta-1, 6-N-acetyl-D-glucosamine N-deacetylase PgaB	*Acinetobacter baumannii*		A0A2I8CS57	--	29437920	Hydrolase activity, acting on carbon-nitrogen (but not peptide) bonds	Not Available
327	Poly-beta-1, 6-N-acetyl-D-glucosamine synthase (Poly-beta-1, 6-GlcNAc synthase) (EC 2.4.1.-)	*Acinetobacter baumannii*		A0A0D5YG83	--	25845845	Acetyl glucosaminyl transferase activity	Not Available
328	Putative inner Membrane protein associated with biofilm formation	*Escherichia coli O45:K1*	Strain S88 / ExPEC	B7MIG7	--	19165319	Not Available	Not Available
329	Putative polysaccharide deacetylase associated with biofilm formation putative lipoprotein	*Escherichia coli O45:K1*	Strain S88 / ExPEC	B7MIG9	--	19165319	Hydrolase activity, acting on carbon-nitrogen (but not peptide) bonds	Not Available
330	Uncharacterized protein	*Deltaproteobacteria bacterium RIFOXYD12_FULL_57_12*		A0A1F9LU99	--	27774985	Not Available	Not Available
331	Uncharacterized protein	*Serratia spp. M24T3*		I0QPB7	--	22740681	Not Available	Not Available
332	Biofilm PGA synthesis lipoprotein PgaB	*Actinobacillus pleuropneumoniae serotype 5b*	Strain L20	A3N3L6	--	18065534	Hydrolase activity, acting on carbon-nitrogen (but not peptide) bonds	Not Available
333	Uncharacterized protein	*Halomonas spp. TD01*		F7SRR8	--	22040376	Not Available	Not Available
334	GGDEF family protein	*Deinococcus radiodurans*	Strain ATCC 13939 / DSM 20539 / JCM 16871 / LMG 4051 / NBRC 15346 / NCIMB 9279 / R1 / VKM B-1422	Q9RZN0	--	10567266	Diguanylate cyclase activity	Not Available
335	GGDEF family protein	*Deinococcus radiodurans*	Strain ATCC 13939 / DSM 20539 / JCM 16871 / LMG 4051 / NBRC 15346 / NCIMB 9279 / R1 / VKM B-1422	Q9RVP4	--	10567266	Diguanylate cyclase activity	Not Available
336	GGDEF family protein	*Deinococcus radiodurans*	Strain ATCC 13939 / DSM 20539 / JCM 16871 / LMG 4051 / NBRC 15346 / NCIMB 9279 / R1 / VKM B-1422	Q9RTW9	--	10567266	Diguanylate cyclase activity	Not Available

Target ID	Bacterial Protein Name	Organism Name	Strain	UniProt ID	PDB ID	PubMed ID	Classification as per UniProt GO Molecular Function	Classification of Function as per PDB
337	GGDEF family protein	*Deinococcus radiodurans*	Strain ATCC 13939 / DSM 20539 / JCM 16871 / LMG 4051 / NBRC 15346 / NCIMB 9279 / R1 / VKM B-1422	Q9RRJ2	--	10567266	Diguanylate cyclase activity	Not Available
338	GGDEF family protein	*Deinococcus radiodurans*	Strain ATCC 13939 / DSM 20539 / JCM 16871 / LMG 4051 / NBRC 15346 / NCIMB 9279 / R1 / VKM B-1422	Q9RZS9	--	10567266	Diguanylate cyclase activity	Not Available
339	Poly-beta-1, 6-N-acetyl-D-glucosamine synthase (Poly-beta-1, 6-GlcNAc synthase) (EC 2.4.1.-)	*Rubrivivax benzoatilyticus JA2 = ATCC BAA-35*		F3LKS7	--	21478355	Acetyl glucosaminyl transferase activity	Not Available
340	Poly-beta-1, 6-N-acetyl-D-glucosamine synthase (Poly-beta-1, 6-GlcNAc synthase) (EC 2.4.1.-)	*Paraburkholderia azotifigens*		A0A5C6VQD9	--	29185955	Acetyl glucosaminyl transferase activity	Not Available
341	Poly-beta-1, 6-N-acetyl-D-glucosamine synthase (Poly-beta-1, 6-GlcNAc synthase) (EC 2.4.1.-)	*Acidiferrobacteraceae bacterium*		A0A7V2NLQ8	--	31911466	Acetyl glucosaminyl transferase activity	Not Available
342	Poly-beta-1, 6-N-acetyl-D-glucosamine N-deacetylase PgaB (EC 3.5.1.-)	*Pseudomonas spp. WS 5010*		A0A7Y0ZL61	--	32587583	Hydrolase activity, acting on carbon-nitrogen (but not peptide) bonds	Not Available
343	Uncharacterized protein	*Lacticaseibacillus fabifermentans T30PCM01*		W6T8S0	--	24558238	Not Available	Not Available
344	Uncharacterized protein	*Xanthomonas oryzae pv. oryzicola*	Strain BLS256	G7TD25	--	21784931	Not Available	Not Available
345	NodB homology domain-containing protein	*Chromobacterium violaceum*	Strain ATCC 12472 / DSM 30191 / JCM 1249 / NBRC 12614 / NCIMB 9131 / NCTC 9757	Q7NQ63	--	14500782	Hydrolase activity, acting on carbon-nitrogen (but not peptide) bonds	Not Available
346	Type II toxin-antitoxin system MqsR family toxin	*Gemmataceae bacterium*		A0A7V3VXR8	--	31911466	Not Available	Not Available
347	Uncharacterized protein	*Lacticaseibacillus fabifermentans T30PCM01*		W6T5A4	--	24558238	Not Available	Not Available
348	Poly-beta-1, 6-N-acetyl-D-glucosamine synthase (Poly-beta-1, 6-GlcNAc synthase) (EC 2.4.1.-)	*Chromobacterium violaceum*	Strain ATCC 12472 / DSM 30191 / JCM 1249 / NBRC 12614 / NCIMB 9131 / NCTC 9757	Q7NTW2	--	14500782	Acetyl glucosaminyl transferase activity	Not Available
349	Hemin storage signal peptide protein	*Chromobacterium violaceum*	Strain ATCC 12472 / DSM 30191 / JCM 1249 / NBRC 12614 / NCIMB 9131 / NCTC 9757	Q7NTW1	--	14500782	Hydrolase activity, acting on carbon-nitrogen (but not peptide) bonds	Not Available
350	Uncharacterized protein	*Methylomicrobium album BG8*		H8GLP1	--	23580712	Not Available	Not Available
351	Uncharacterized protein	*Chromobacterium violaceum*	Strain ATCC 12472 / DSM 30191 / JCM 1249 / NBRC 12614 / NCIMB 9131 / NCTC 9757	Q7NTW3	--	14500782	Not Available	Not Available
352	Poly-beta-1, 6-N-acetyl-D-glucosamine synthase (Poly-beta-1, 6-GlcNAc synthase) (EC 2.4.1.-)	*Pseudomonas orientalis*		A0A2L0RQV7	--	29479340	Acetyl glucosaminyl transferase activity	Not Available
353	Poly-beta-1, 6-N-acetyl-D-glucosamine synthase (Poly-beta-1, 6-GlcNAc synthase) (EC 2.4.1.-)	*Xanthomonas oryzae pv. oryzicola*	Strain BLS256	G7TD26	--	21784931	Acetyl glucosaminyl transferase activity	Not Available
354	Poly-beta-1, 6-N-acetyl-D-glucosamine N-deacetylase PgaB	*Acinetobacter spp. ACNIH4*		A0A2S4TAE7	--	29437920	Hydrolase activity, acting on carbon-nitrogen (but not peptide) bonds	Not Available
355	Poly-beta-1, 6-N-acetyl-D-glucosamine N-deacetylase PgaB	*Acinetobacter spp. ACNIH4*		A0A2S4TAJ4	--	29437920	Hydrolase activity, acting on carbon-nitrogen (but not peptide) bonds	Not Available
356	GGDEF domain-containing protein	*Thermotoga maritima*	Strain ATCC 43589 / DSM 3109 / JCM 10099 / NBRC 100826 / MSB8	Q9X067	--	10360571	Diguanylate cyclase activity	Not Available
357	Response regulator	*Thermotoga maritima*	Strain ATCC 43589 / DSM 3109 / JCM 10099 / NBRC 100826 / MSB8	Q9WXZ0	--	10360571	Diguanylate cyclase activity	Not Available
358	GGDEF domain-containing protein	*Thermotoga maritima*	Strain ATCC 43589 / DSM 3109 / JCM 10099 / NBRC 100826 / MSB8	Q9X1T0	--	10360571	Diguanylate cyclase activity	Not Available
359	Poly-beta-1, 6-N-acetyl-D-glucosamine synthase (Poly-beta-1, 6-GlcNAc synthase) (EC 2.4.1.-)	*Staphylococcus simiae CCM 7213 = CCUG 51256*		G5JK07	--	22272658	Acetyl glucosaminyl transferase activity	Not Available
360	GGDEF domain-containing protein	*Thermotoga maritima*	Strain ATCC 43589 / DSM 3109 / JCM 10099 / NBRC 100826 / MSB8	Q9X1S4	--	10360571	Diguanylate cyclase activity	Not Available
361	Poly-beta-1, 6-N-acetyl-D-glucosamine synthase (Poly-beta-1, 6-GlcNAc synthase) (EC 2.4.1.-)	*Escherichia coli O45:K1*	Strain S88 / ExPEC	B7MIG8	--	19165319	Acetyl glucosaminyl transferase activity	Not Available
362	Poly-beta-1, 6-N-acetyl-D-glucosamine synthase (Poly-beta-1, 6-GlcNAc synthase) (EC 2.4.1.-)	*Pseudomonas chlororaphis*		A0A0D5Y7R2	--	25679537	Acetyl glucosaminyl transferase activity	Not Available
363	Hemin storage protein	*Stenotrophomonas geniculata N1*		A0A0L8AEA0	--	22689240	Hydrolase activity, acting on carbon-nitrogen (but not peptide) bonds	Not Available
364	Uncharacterized protein	*Actinobacteria bacterium*		A0A7V4MZE2	--	31911466	Not Available	Not Available
365	Poly-beta-1, 6-N-acetyl-D-glucosamine synthase (Poly-beta-1, 6-GlcNAc synthase) (EC 2.4.1.-)	*Pseudomonas spp. WS 5413*		A0A7Y0Z1V7	--	32587583	Acetyl glucosaminyl transferase activity	Not Available
366	Uncharacterized protein	*Hyphomonaceae bacterium*		A0A3D2W3N1	--	30148503	Not Available	Not Available
367	Putative PGA biosynthesis protein	*Escherichia coli O83:H1*	Strain NRG 857C / AIEC	A0A0H3EHC0	--	21108814	Not Available	Not Available
368	Poly-beta-1, 6-N-acetyl-D-glucosamine biosynthesis protein PgaD	*Pseudomonas spp. WS 5413*		A0A7Y0Z1X4	--	32587583	Not Available	Not Available
369	Poly-beta-1, 6-N-acetyl-D-glucosamine N-deacetylase PgaB (EC 3.5.1.-)	*Pseudomonas spp. WS 5413*		A0A7Y0Z1Z3	--	32587583	Hydrolase activity, acting on carbon-nitrogen (but not peptide) bonds	Not Available
370	Poly-beta-1, 6-N-acetyl-D-glucosamine N-deacetylase PgaB	*Methylophaga spp.*		A0A7C5BNS2	--	31911466	Hydrolase activity, acting on carbon-nitrogen (but not peptide) bonds	Not Available
371	Uncharacterized protein	*Stenotrophomonas spp.*		A0A3D0GBU3	--	30148503	Not Available	Not Available
372	mRNA interferase MqsR	*Hyphomonaceae bacterium*		A0A352CC32	--	30148503	Not Available	Not Available
373	Poly-beta-1, 6-N-acetyl-D-glucosamine synthase (Poly-beta-1, 6-GlcNAc synthase) (EC 2.4.1.-)	*Stenotrophomonas spp.*		A0A3C0X5Z0	--	30148503	Acetyl glucosaminyl transferase activity	Not Available
374	Poly-beta-1, 6-N-acetyl-D-glucosamine biosynthesis protein PgaD	*Stenotrophomonas spp.*		A0A357N0H1	--	30148503	Not Available	Not Available
375	Poly-beta-1, 6-N-acetyl-D-glucosamine synthase (Poly-beta-1, 6-GlcNAc synthase) (EC 2.4.1.-)	*Stenotrophomonas spp.*		A0A349Y4K4	--	30148503	Acetyl glucosaminyl transferase activity	Not Available

Target ID	Bacterial Protein Name	Organism Name	Strain	UniProt ID	PDB ID	PubMed ID	Classification as per UniProt GO Molecular Function	Classification of Function as per PDB
376	Poly-beta-1, 6-N-acetyl-D-glucosamine synthase (Poly-beta-1, 6-GlcNAc synthase) (EC 2.4.1.-)	*Escherichia coli O83:H1*	Strain NRG 857C / AIEC	A0A0H3EI51	--	21108814	Acetyl glucosaminyl transferase activity	Not Available
377	Surface composition regulator	*Escherichia coli O83:H1*	Strain NRG 857C / AIEC	A0A0H3EM10	--	21108814	Not Available	Not Available
378	Poly-beta-1, 6-N-acetyl-D-glucosamine N-deacetylase PgaB	*Deltaproteobacteria bacterium RBG_13_60_28*		A0A1F9BIL4	--	27774985	Hydrolase activity, acting on carbon-nitrogen (but not peptide) bonds	Not Available
379	Poly-beta-1, 6-N-acetyl-D-glucosamine synthase (Poly-beta-1, 6-GlcNAc synthase) (EC 2.4.1.-)	*Deltaproteobacteria bacterium RBG_13_60_28*		A0A1F9BKU9	--	27774985	Acetyl glucosaminyl transferase activity	Not Available
380	Poly-beta-1, 6-N-acetyl-D-glucosamine biosynthesis protein PgaD	*Stenotrophomonas spp.*		A0A3D0GC17	--	30148503	Not Available	Not Available
381	Poly-beta-1, 6-N-acetyl-D-glucosamine N-deacetylase PgaB	*Stenotrophomonas spp.*		A0A3C0X5X6	--	30148503	Hydrolase activity, acting on carbon-nitrogen (but not peptide) bonds	Not Available
382	Poly-beta-1, 6-N-acetyl-D-glucosamine synthase (Poly-beta-1, 6-GlcNAc synthase) (EC 2.4.1.-)	*Stenotrophomonas spp.*		A0A356JD40	--	30148503	Acetyl glucosaminyl transferase activity	Not Available
383	Poly-beta-1, 6-N-acetyl-D-glucosamine biosynthesis protein PgaD (Fragment)	*Stenotrophomonas spp.*		A0A3C0X5Y8	--	30148503	Not Available	Not Available
384	Poly-beta-1, 6-N-acetyl-D-glucosamine synthase (Poly-beta-1, 6-GlcNAc synthase) (EC 2.4.1.-)	*Stenotrophomonas spp.*		A0A3D4THW1	--	30148503	Acetyl glucosaminyl transferase activity	Not Available
385	Poly-beta-1, 6-N-acetyl-D-glucosamine N-deacetylase PgaB	*Stenotrophomonas spp.*		A0A349Y4K5	--	30148503	Hydrolase activity, acting on carbon-nitrogen (but not peptide) bonds	Not Available
386	Poly-beta-1, 6-N-acetyl-D-glucosamine synthase (Poly-beta-1, 6-GlcNAc synthase) (EC 2.4.1.-)	*Methylococcaceae bacterium*		A0A351V597	--	30148503	Acetyl glucosaminyl transferase activity	Not Available
387	Poly-beta-1, 6-N-acetyl-D-glucosamine synthase (Poly-beta-1, 6-GlcNAc synthase) (EC 2.4.1.-)	*Paraburkholderia hospita BS001*		I5CU28	--	22843604	Acetyl glucosaminyl transferase activity	Not Available
388	Type IV pilin PilA	*Vibrio vulnificus*		Q8VVV3	--	15731039	Not Available	Not Available
389	Uncharacterized protein	*Lactococcus lactis subspp. cremoris IBB477*		A0A1E7G187	--	27687992	Not Available	Not Available
390	Putative transmembrane protein	*blood disease bacterium R229*		G2ZXS3	--	21931687	Not Available	Not Available
391	Surface composition regulator	*Salmonella enterica subspp. enterica serovar*	Hvittingfoss	A0A5I0UXC3	--	30286803	Not Available	Not Available
392	IcaA (Fragment)	*Staphylococcus simulans*		Q83ZH8	--	12957956	Not Available	Not Available
393	Surface composition regulator	*Salmonella enterica subspp. enterica serovar*	Java	A0A741RTZ8	--	30286803	Not Available	Not Available
394	PsoA	*Pseudomonas putida*	Arthrobacter Siderocapsulatus	A8MN36	--	16585751; 18599835	Catalytic activity, phosphopantetheine binding	Not Available
395	Putative transmembrane protein	*Ralstonia syzygii R24*		G3A8J7	--	21931687	Not Available	Not Available
396	Poly-beta-1, 6-N-acetyl-D-glucosamine synthase (Poly-beta-1, 6-GlcNAc synthase) (EC 2.4.1.-)	*Staphylococcus aureus*		A0A1D0C161	--	20876371	Acetyl glucosaminyl transferase activity	Not Available
397	Hemin storage signal peptide protein	*Paraburkholderia hospita BS001*		I5CU27	--	22843604	Hydrolase activity, acting on carbon-nitrogen (but not peptide) bonds	Not Available
398	Surface composition regulator	*Salmonella enterica subspp. indica serovar*	45:a:e, n, x	A0A737BKL7	--	30286803	Not Available	Not Available
399	Poly-beta-1, 6-N-acetyl-D-glucosamine synthase (Poly-beta-1, 6-GlcNAc synthase) (EC 2.4.1.-)	*blood disease bacterium R229*		G2ZXS2	--	21931687	Acetyl glucosaminyl transferase activity	Not Available
400	Putative polysaccharide deacetylase associated with biofilm formation putative lipoprotein (PgaB)	*blood disease bacterium R229*		G2ZXS1	--	21931687	Hydrolase activity, acting on carbon-nitrogen (but not peptide) bonds	Not Available
401	Surface composition regulator	*Salmonella enterica subspp. houtenae serovar*	16:z4, z32:-	A0A735L3N9	--	30286803	Not Available	Not Available
402	IcaA (Fragment)	*Staphylococcus aureus*		Q83ZH9	--	12957956	Not Available	Not Available
403	Poly-beta-1, 6-N-acetyl-D-glucosamine synthase (Poly-beta-1, 6-GlcNAc synthase) (EC 2.4.1.-)	*Staphylococcus aureus*		A0A6A9GS78	--	31682959	Acetyl glucosaminyl transferase activity	Not Available
404	Glycerol esther hydrolase (Fragment)	*Staphylococcus aureus*		Q9RQP5	--	10496925	Hydrolase activity	Not Available
405	Surface composition regulator	*Salmonella enterica subspp. indica serovar*	45:a:e, n, x	A0A737BK14	--	30286803	Not Available	Not Available
406	Conjugative transfer protein Pilx7 (PilX7) (Pilx7 protein) (Putative PilX7)	*Escherichia coli*		B0ZDY1	--	15328093; 18440636; 19717626; 21831988; 24862095	Not Available	Not Available
407	Poly-beta-1, 6-N-acetyl-D-glucosamine N-deacetylase PgaB (Polysaccharide deacetylase)	*Burkholderia ambifaria*	Strain ATCC BAA-244 / AMMD Burkholderia cepacia Strain AMMD	Q0B9V5	--	25931592	Hydrolase activity, acting on carbon-nitrogen (but not peptide) bonds	Not Available
408	Poly-beta-1, 6-N-acetyl-D-glucosamine synthase (Poly-beta-1, 6-GlcNAc synthase) (EC 2.4.1.-)	*Stenotrophomonas maltophilia*	Pseudomonas maltophilia Xanthomonas maltophilia	A0A0K2IYC8	--	26324280; 29170656	Acetyl glucosaminyl transferase activity	Not Available
409	Poly-beta-1, 6-N-acetyl-D-glucosamine synthase (Poly-beta-1, 6-GlcNAc synthase) (EC 2.4.1.-)	*Enterobacter kobei*		A0A2J0PJF5	--	29088362	Acetyl glucosaminyl transferase activity	Not Available
410	Type II toxin-antitoxin system MqsR family toxin	*Deltaproteobacteria bacterium*		A0A7C8AFX8	--	32737307	Not Available	Not Available
411	Poly-beta-1, 6-N-acetyl-D-glucosamine biosynthesis protein PgaD	*Escherichia coli*		A0A0A1A417	--	30286803; 30947268	Not Available	Not Available
412	Poly-beta-1, 6-N-acetyl-D-glucosamine N-deacetylase PgaB (EC 3.5.1.-)	*Escherichia coli*		A0A795PNG2	--	30286803	Hydrolase activity, acting on carbon-nitrogen (but not peptide) bonds	Not Available
413	GCU-specific mRNA interferase toxin of the MqsR-MqsA toxin-antitoxin system (Type II toxin-antitoxin system toxin MqsR) (EC 3.1.26.-) (mRNA interferase MqsR) (EC 3.1.-.-)	*Escherichia coli*		A0A0H0HXD8	--	30286803; 31477907	Hydrolase activity	Not Available
414	Lipoprotein YcdR (EC 3.5.1.-) (Poly-beta-1, 6-N-acetyl-D-glucosamine N-deacetylase PgaB)	*Escherichia coli*		A0A2T3TFG9	--	31477907	Hydrolase activity, acting on carbon-nitrogen (but not peptide) bonds	Not Available
415	PGA biosynthesis protein (Poly-beta-1, 6-N-acetyl-D-glucosamine biosynthesis protein PgaD)	*Escherichia coli*		A0A0D8VWU3	--	30286803; 31477907; 32021174	Not Available	Not Available
416	Poly-beta-1, 6-N-acetyl-D-glucosamine synthase (Poly-beta-1, 6-GlcNAc synthase) (EC 2.4.1.-)	*Escherichia coli*		A0A1U9U1S6	--	30286803; 31477907	Acetyl glucosaminyl transferase activity	Not Available
417	Poly-beta-1, 6-N-acetyl-D-glucosamine synthase (Poly-beta-1, 6-GlcNAc synthase) (EC 2.4.1.-)	*Escherichia coli*		A0A3P1J9P9	--	30286803	Acetyl glucosaminyl transferase activity	Not Available
418	Poly-beta-1, 6-N-acetyl-D-glucosamine biosynthesis protein PgaD	*Enterobacter kobei*		A0A2J0PIC7	--	29088362	Not Available	Not Available
419	Surface composition regulator	*Salmonella enterica subspp. enterica serovar*	Give	A0A5C5HDB9	--	26139723; 30286803; 30902850	Not Available	Not Available
420	Poly-beta-1, 6-N-acetyl-D-glucosamine synthase (Poly-beta-1, 6-GlcNAc synthase) (EC 2.4.1.-)	*Escherichia coli*		C3TEV3	--	19439656	Acetyl glucosaminyl transferase activity	Not Available
421	Surface composition regulator	*Salmonella typhimurium*	Strain SL1344	A0A0H3NI14	--	22538806; 30286803	Not Available	Not Available
422	Poly-beta-1, 6-N-acetyl-D-glucosamine N-deacetylase PgaB	*Escherichia coli*		A0A3K0QE29	--	30286803	Hydrolase activity, acting on carbon-nitrogen (but not peptide) bonds	Not Available

Target ID	Bacterial Protein Name	Organism Name	Strain	UniProt ID	PDB ID	PubMed ID	Classification as per UniProt GO Molecular Function	Classification of Function as per PDB
423	Uncharacterized protein	*Deltaproteobacteria bacterium*		A0A661K4W2	--	31911466	Not Available	Not Available
424	LuxR regulator (SmcR-like protein VvpR)	*Vibrio scophthalmi LMG 19158*		F9RJF4	--	23198796; 21930677	DNA binding	Not Available
425	Poly-beta-1, 6-N-acetyl-D-glucosamine biosynthesis protein PgaD	*Escherichia coli*		A0A2A6Q924	--	28221970; 30924410	Not Available	Not Available
426	Poly-beta-1, 6-N-acetyl-D-glucosamine biosynthesis protein PgaD (Transporter)	*Acinetobacter pittii*	Acinetobacter genomospp. 3	A0A242STX0	--	30036696	Not Available	Not Available
427	Poly-beta-1, 6-N-acetyl-D-glucosamine N-deacetylase PgaB	*Escherichia coli*		A0A2A6Q8V5	--	28221970; 30924410	Hydrolase activity, acting on carbon-nitrogen (but not peptide) bonds	Not Available
428	Poly-beta-1, 6-N-acetyl-D-glucosamine N-deacetylase PgaB (EC 3.5.1.-)	*Escherichia coli*		A0A233ZEF3	--	30286803; 32021174	Hydrolase activity, acting on carbon-nitrogen (but not peptide) bonds	Not Available
429	Surface composition regulator	*Escherichia coli*		A0A1M0PIU6	--	30286803; 31477907	Not Available	Not Available
430	Poly-beta-1, 6-N-acetyl-D-glucosamine synthase (Poly-beta-1, 6-GlcNAc synthase) (EC 2.4.1.-)	*Escherichia coli*		A0A2A6Q915	--	28221970; 30924410	Acetyl glucosaminyl transferase activity	Not Available
431	Molecular chaperone (MrkB) (Putative bacterial pili assembly chaperone) (Putative fimbrial chaperone protein) (Type 3 fimbria chaperone MrkB)	*Escherichia coli*		B0LHG6	--	18055599; 19717626; 22479389; 26441869; 30286803	Not Available	Not Available
432	Plasmid stability protein StbD (Putative antitoxin) (Plasmid stability protein, antitoxin StbD) (Plasmid stabilization protein) (Putative antitoxin of toxin-antitoxin system) (RelB / StbD replicon stabilization protein (Antitoxin to RelE / StbE)) (Stability protein) (Stability protein StbD) (StbD) (StbD stability protein) (Toxin-antitoxin system, antitoxin component)	*Escherichia coli*		B0EYQ2	--	15328093; 17936903; 18440636; 19717626; 21831988; 22470007; 22479389; 25403661; 25421479; 26048440; 30286803; 30924410; 31642700; 31652858	Not Available	Not Available
433	Poly-beta-1, 6-N-acetyl-D-glucosamine synthase (Poly-beta-1, 6-GlcNAc synthase) (EC 2.4.1.-)	*Escherichia coli*		A0A1Y5BGC5	--	30286803	Acetyl glucosaminyl transferase activity	Not Available
434	Biofilm PGA synthesis protein PgaD (PGA biosynthesis protein) (Poly-beta-1, 6-N-acetyl-D-glucosamine biosynthesis) (Poly-beta-1, 6-N-acetyl-D-glucosamine biosynthesis protein PgaD)	*Escherichia coli*		C3TEV8	--	19439656; 23275093; 26441869; 30286803; 29437920; 30924410; 31642700; 31652858; 31477907; 30948755; 32739475; 28953937	Not Available	Not Available
435	Poly-beta-1, 6-N-acetyl-D-glucosamine synthase (Poly-beta-1, 6-GlcNAc synthase) (EC 2.4.1.-)	*Escherichia coli*		J7QDP1	--	23275093; 30286803; 29437920; 30924410; 31642700; 31652858; 31477907; 30948755; 28953937	Acetyl glucosaminyl transferase activity	Not Available
436	Surface composition regulator	*Escherichia coli*		J7QHM3	--	23275093; 26441869; 26227606; 28686503; 30286803; 29437920; 30924410; 31642700; 31652858; 31477907; 30947268; 30948755; 32739475; 32599678; 28953937	Not Available	Not Available
437	Poly-beta-1, 6-N-acetyl-D-glucosamine N-deacetylase PgaB (EC 3.5.1.-)	*Escherichia coli*		A0A192EMZ6	--	30286803	Hydrolase activity, acting on carbon-nitrogen (but not peptide) bonds	Not Available
438	Poly-beta-1, 6-N-acetyl-D-glucosamine synthase (Poly-beta-1, 6-GlcNAc synthase) (EC 2.4.1.-)	*Escherichia coli*		W8TQ48	--	26441869	Acetyl glucosaminyl transferase activity	Not Available
439	Surface composition regulator	*Salmonella newport*		A0A0R9NRZ2	--	30286803	Not Available	Not Available
440	Poly-beta-1, 6-N-acetyl-D-glucosamine synthase (Poly-beta-1, 6-GlcNAc synthase) (EC 2.4.1.-)	*Escherichia coli*		A0A1D7PYQ0	--	30286803; 30947268	Acetyl glucosaminyl transferase activity	Not Available
441	Surface composition regulator	*Escherichia coli*		E2QE75	--	28221970; 28686503; 30286803; 30924410; 31477907	Not Available	Not Available
442	Surface composition regulator	*Salmonella enterica subspp. enterica serovar*	Weltevreden	A0A3V7I4R8	--	31099590	Not Available	Not Available
443	Poly-beta-1, 6-N-acetyl-D-glucosamine N-deacetylase PgaB (EC 3.5.1.-)	*Escherichia coli*		A0A4Y8EDQ8	--	32739475	Hydrolase activity, acting on carbon-nitrogen (but not peptide) bonds	Not Available
444	Poly-beta-1, 6-N-acetyl-D-glucosamine synthase (Poly-beta-1, 6-GlcNAc synthase) (EC 2.4.1.-)	*Acinetobacter pittii*	Acinetobacter genomospp. 3	A0A242SD62	--	30036696	Acetyl glucosaminyl transferase activity	Not Available
445	Poly-beta-1, 6-N-acetyl-D-glucosamine synthase (Poly-beta-1, 6-GlcNAc synthase) (EC 2.4.1.-)	*Acinetobacter pittii*	Acinetobacter genomospp. 3	A0A0N9ALG4	--	30036696	Acetyl glucosaminyl transferase activity	Not Available
446	ActX (ActX protein) (Transcription termiNot Availabletion factor NusG) (Transcriptionalactivator)	*Escherichia coli*		B0ZDY9	--	15328093; 18440636; 19717626; 21831988; 30286803; 31652858	Not Available	Not Available
447	Surface composition regulator	*Salmonella enterica subspp. enterica serovar*	Sandiego	A0A5J1IID0	--	30286803	Not Available	Not Available

Target ID	Bacterial Protein Name	Organism Name	Strain	UniProt ID	PDB ID	PubMed ID	Classification as per UniProt GO Molecular Function	Classification of Function as per PDB
448	Chromosome partitioning protein ParA (ParA family protein) (ParF) (Partitioning protein ParA) (Plasmid partition protein) (Plasmid partition protein ParF) (Plasmid partitioning protein ParF) (Putative crown gall tumor protein VirC1)	*Escherichia coli*		B0EYM3	--	17936903; 19717626; 22470007; 22479389; 31652858	Not Available	Not Available
449	Surface composition regulator	*Pluralibacter gergoviae*	Enterobacter gergoviae	A0A1S2BI09	--	25502672	Not Available	Not Available
450	Poly-beta-1, 6-N-acetyl-D-glucosamine biosynthesis protein PgaD	*Escherichia coli*		A0A4Y8EDW4	--	32739475	Not Available	Not Available
451	Poly-beta-1, 6-N-acetyl-D-glucosamine N-deacetylase (EC 3.5.1.-) (Poly-beta-1, 6-N-acetyl-D-glucosamine N-deacetylase PgaB)	*Escherichia coli*		A0A2X6F457	--	30286803	Hydrolase activity, acting on carbon-nitrogen (but not peptide) bonds	Not Available
452	Poly-beta-1, 6-N-acetyl-D-glucosamine synthase (Poly-beta-1, 6-GlcNAc synthase) (EC 2.4.1.-)	*Escherichia coli*		A0A1Y2ZN44	--	30924410	Acetyl glucosaminyl transferase activity	Not Available
453	Lipoprotein YcdR (Poly-beta-1, 6-N-acetyl-D-glucosamine N-deacetylase) (Poly-beta-1, 6-N-acetyl-D-glucosamine N-deacetylase PgaB) (EC 3.5.1.-)	*Escherichia coli*		A0A0L6Y2B4	--	30286803	Hydrolase activity, acting on carbon-nitrogen (but not peptide) bonds	Not Available
454	PGA biosynthesis protein (Poly-beta-1, 6-N-acetyl-D-glucosamine biosynthesis protein PgaD)	*Escherichia coli*		A0A178T084	--	30286803	Not Available	Not Available
455	Poly-beta-1, 6-N-acetyl-D-glucosamine N-deacetylase PgaB (EC 3.5.1.-)	*Escherichia coli*		A0A0J2BYQ8	--	28686503; 30286803	Hydrolase activity, acting on carbon-nitrogen (but not peptide) bonds	Not Available
456	Poly-beta-1, 6-N-acetyl-D-glucosamine N-deacetylase (Poly-beta-1, 6-N-acetyl-D-glucosamine N-deacetylase PgaB) (EC 3.5.1.-)	*Escherichia coli*		A0A178T0C2	--	30286803	Hydrolase activity, acting on carbon-nitrogen (but not peptide) bonds	Not Available
457	Surface composition regulator	*Salmonella enterica subspp. enterica serovar*	Bredeney	A0A3V9PUT8	--	30286803	Not Available	Not Available
458	S-ribosylhomocysteine lyase (EC 4.4.1.21) (AI-2 synthesis protein) (Autoinducer-2 production protein LuxS)	*Streptococcus salivarius K12*		J7TQ69	--	23045482	Iron ion binding, S-ribosylhomocysteine lyase activity	Not Available
459	Poly-beta-1, 6-N-acetyl-D-glucosamine N-deacetylase PgaB (EC 3.5.1.-)	*Escherichia coli*		A0A0A1A2S7	--	30286803; 30947268	Hydrolase activity, acting on carbon-nitrogen (but not peptide) bonds	Not Available
460	MrkA fimbrial protein (MrkA fimbrial protein major fimbrial subunit) (Type 1 fimbrial protein) (Type 3 fimbria major subunit MrkA)	*Escherichia coli*		B0ZDW4	--	15328093; 18440636; 30286803; 31652858	Not Available	Not Available
461	Conjugal transfer protein (Conjugal transfer protein Pilx9) (PilX9) (Pilx9 protein) (Pilx9 protein, putative conjugal transfer protein, putative exported protein) (TrbG / VirB9 family P-type conjugative transfer protein)	*Escherichia coli*		B0ZDX9	--	15328093; 18440636; 19717626; 22479389; 31652858	Not Available	Not Available
462	Poly-beta-1, 6-N-acetyl-D-glucosamine synthase (Poly-beta-1, 6-GlcNAc synthase) (EC 2.4.1.-)	*Staphylococcus aureus*	Strain Newman	A0A0H3KAW6	--	17951380	Acetyl glucosaminyl transferase activity	Not Available
463	Surface composition regulator	*Salmonella enterica subspp. enterica serovar*	Schwarzengrund	A0A3T2ZVE9	--	30286803	Not Available	Not Available
464	Poly-beta-1, 6-N-acetyl-D-glucosamine biosynthesis protein PgaD	*Acinetobacter cumulans*		A0A498CYW7	--	30808586	Not Available	Not Available
465	Motility quorum-sensing regulator / GCU-specific mRNA interferase toxin (Type II toxin-antitoxin system MqsR family toxin)	*Pseudomonas chengduensis*		A0A1G6UUI8	--	32587583	Not Available	Not Available
466	Bacteriophage like protein (Putative toxin-antitoxin system, toxin component)	*Lactobacillus johnsonii ATCC 33200*		C2E3I3	--	26415554	Not Available	Not Available
467	Major facilitator superfamily permease	*Acinetobacter oleivorans*	Strain JCM 16667 / KCTC 23045 / DR1	D3JTG4	--	20629796; 20639327	Transmembrane transporter activity	Not Available
468	Acyl-CoA dehydrogenase	*Acinetobacter oleivorans*	Strain JCM 16667 / KCTC 23045 / DR1	D3JTG9	--	20629796; 20639327	Flavin adenine dinucleotide binding, oxidoreductase activity, acting on the CH-CH group of donors	Not Available
469	AqsR (Transcriptionalactivator protein)	*Acinetobacter oleivorans*	Strain JCM 16667 / KCTC 23045 / DR1	D3JTG7	--	20629796; 20639327	DNA binding	Not Available
470	Poly-beta-1, 6-N-acetyl-D-glucosamine synthase (Poly-beta-1, 6-GlcNAc synthase) (EC 2.4.1.-)	*Acinetobacter schindleri*		A0A2S1FF20	--	29954893	Acetyl glucosaminyl transferase activity	Not Available
471	Surface composition regulator	*Salmonella diarizonae*		A0A2I5HLE4	--	30286803	Not Available	Not Available
472	Poly-beta-1, 6-N-acetyl-D-glucosamine N-deacetylase PgaB	*Acinetobacter schindleri*		A0A2S1FF79	--	29954893	Hydrolase activity, acting on carbon-nitrogen (but not peptide) bonds	Not Available
473	Poly-beta-1, 6-N-acetyl-D-glucosamine N-deacetylase PgaB	*Acinetobacter schindleri*		A0A2S1FF30	--	29954893	Hydrolase activity, acting on carbon-nitrogen (but not peptide) bonds	Not Available
474	Acyl-homoserine-lactone synthase (EC 2.3.1.184) (Autoinducer synthesis protein)	*Acinetobacter oleivorans*	Strain JCM 16667 / KCTC 23045 / DR1	D3JTG5	--	20629796; 20639327	N-acyl homoserine lactone synthase activity	Not Available
475	Poly-beta-1, 6-N-acetyl-D-glucosamine biosynthesis protein PgaD	*Piscirickettsiaceae bacterium*		A0A2A4PM66	--	29099490	Not Available	Not Available
476	Acyl-CoA synthetase (AMP-forming) / AMP-acid ligase II (Long chain fatty acid-CoA ligase)	*Acinetobacter oleivorans*	Strain JCM 16667 / KCTC 23045 / DR1	D3JTG8	--	20629796; 20639327	Ligase activity	Not Available
477	Poly-beta-1, 6-N-acetyl-D-glucosamine N-deacetylase PgaB (Polysaccharide deacetylase) (Xylanae / chitin deacetylase) (EC 3.5.1.-)	*Acinetobacter baumannii*		A0A0D5YHR1	--	25845845	Hydrolase activity, acting on carbon-nitrogen (but not peptide) bonds, Hydrolase activity, acting on glycosyl bonds	Not Available
478	Putative inner Membrane protein (Uncharacterized protein yigG)	*Salmonella typhimurium*	Strain LT2 / SGSC1412 / ATCC 700720	Q7CPD6	--	11677609	Not Available	Not Available
479	Surface composition regulator	*Salmonella enterica subspp. enterica serovar*	Java	A0A426WQB0	--	30286803; 30902850; 31099590	Not Available	Not Available
480	Surface composition regulator	*Citrobacter pasteurii*		A0A7X5FWW8	--	30087402	Not Available	Not Available
481	Surface composition regulator	*Salmonella enterica subspp. enterica serovar*	Tennessee	A0A3G3DVF2	--	30286803; 30902850	Not Available	Not Available
482	Biofilm PGA synthesis protein pgaD (Biofilm synthesis auxiliary protein) (Poly-beta-1, 6-N-acetyl-D-glucosamine biosynthesis protein PgaD) (Transporter)	*Acinetobacter baumannii*		A0A059ZP60	--	25136007; 26170289; 29137647; 30124845	Not Available	Not Available
483	Biofilm PGA synthesis lipoprotein pgaB (Lipoprotein HmsF) (EC 3.5.1.-) (Poly-beta-1, 6-N-acetyl-D-glucosamine N-deacetylase PgaB) (Polysaccharide deacetylase)	*Acinetobacter baumannii*		A0A090BDC4	--	25845845; 27917170; 29137647	Hydrolase activity, acting on carbon-nitrogen (but not peptide) bonds	Not Available
484	Csu fimbrial tip adhesin CsuE (CsuE)	*Acinetobacter baumannii*		Q6XBY2	--	14663080	Not Available	Not Available
485	Poly-beta-1, 6-N-acetyl-D-glucosamine biosynthesis protein PgaD	*Citrobacter pasteurii*		A0A7X5JM17	--	30087402	Not Available	Not Available
486	Poly-beta-1, 6-N-acetyl-D-glucosamine biosynthesis protein PgaD	*Acinetobacter baumannii*		A0A090B5L8	--	27917170; 29137647; 30124845; 29437920	Not Available	Not Available

Target ID	Bacterial Protein Name	Organism Name	Strain	UniProt ID	PDB ID	PubMed ID	Classification as per UniProt GO Molecular Function	Classification of Function as per PDB
487	Poly-beta-1, 6-N-acetyl-D-glucosamine N-deacetylase PgaB (Polysaccharide deacetylase)	*Acinetobacter baumannii*		A0A6I1NXE1	--	26170289	Hydrolase activity, acting on carbon-nitrogen (but not peptide) bonds	Not Available
488	Hemin storage protein (Poly-beta-1, 6-N-acetyl-D-glucosamine N-deacetylase PgaB)	*Acinetobacter baumannii*		A0A090C165	--	25136007; 26170289	Hydrolase activity, acting on carbon-nitrogen (but not peptide) bonds	Not Available
489	Surface composition regulator	*Salmonella enterica I*		A0A3U0IR89	--	30286803	Not Available	Not Available
490	Poly-beta-1, 6-N-acetyl-D-glucosamine synthase (Poly-beta-1, 6-GlcNAc synthase) (EC 2.4.1.-)	*Pseudomonas baetica*		A0A2N0DQC6	--	29579234	Acetyl glucosaminyl transferase activity	Not Available
491	Biofilm PGA synthesis protein PgaD (Poly-beta-1, 6-N-acetyl-D-glucosamine biosynthesis protein PgaD)	*Pseudomonas baetica*		A0A2N0DQB9	--	29579234	Not Available	Not Available
492	Poly-beta-1, 6-N-acetyl-D-glucosamine biosynthesis protein PgaD	*Xanthomonas cucurbitae*		A0A2S7DKA1	--	33734871	Not Available	Not Available
493	Poly-beta-1, 6-N-acetyl-D-glucosamine biosynthesis protein PgaD	*Pseudomonas proteolytica*		A0A7Y1H7A3	--	32587583	Not Available	Not Available
494	Motility quorum-sensing regulator / GCU-specific mRNA interferase toxin (Type II toxin-antitoxin system MqsR family toxin)	*Pseudomonas baetica*		A0A2N0DMT7	--	29579234	Not Available	Not Available
495	Surface composition regulator	*Salmonella enterica subspp. enterica serovar*	Mikawasima	A0A5J0RCF5	--	30286803	Not Available	Not Available
496	Surface composition regulator	*Salmonella enterica subspp. diarizonae serovar*	48:i:z	A0A735VU22	--	30286803	Not Available	Not Available
497	Probable regulator protein RsaL (RsaL) (RsaL protein)	*Pseudomonas putida*	Arthrobacter Siderocapsulatus	Q8GEL9	--	12450862; 15345437; 16585751	Not Available	Not Available
498	Uncharacterized protein	*Desulfuromona spp.*		A0A357YHN4	--	30148503	Not Available	Not Available
499	Hemin storage protein (Poly-beta-1, 6-N-acetyl-D-glucosamine biosynthesis protein PgaD)	*Stenotrophomonas maltophilia*	Pseudomonas maltophilia Xanthomonas maltophilia	A0A0K2IM64	--	26324280	Not Available	Not Available
500	Surface composition regulator	*Salmonella enterica subspp. enterica serovar*	4, [5], 12:b:-	A0A741FNZ1	--	30286803	Not Available	Not Available
501	Surface composition regulator	*Salmonella enterica subspp. houtenae serovar*	45:g, z51:-	A0A701UF81	--	30286803	Not Available	Not Available
502	Putative cytoplasmic protein (Transposase)	*Escherichia coli*		B0LHH0	--	18055599; 19717626	Sequence-specific DNA binding	Not Available
503	Poly-beta-1, 6-N-acetyl-D-glucosamine N-deacetylase PgaB	*Pseudomonas spp. GM79*		J2X5H7	--	23045501	Hydrolase activity, acting on carbon-nitrogen (but not peptide) bonds	Not Available
504	mRNA interferase MqsR	*Photorhabdus spp. S10-54*		A0A329WE01	--	29877789	Not Available	Not Available
505	Poly-beta-1, 6-N-acetyl-D-glucosamine synthase (Poly-beta-1, 6-GlcNAc synthase) (EC 2.4.1.-)	*Pseudomonas spp. GM79*		J3IHV5	--	23045501	Acetyl glucosaminyl transferase activity	Not Available
506	Uncharacterized protein	*Gammaproteobacteria bacterium*		A0A3C0I1D7	--	30148503	Not Available	Not Available
507	Poly-beta-1, 6-N-acetyl-D-glucosamine synthase (Poly-beta-1, 6-GlcNAc synthase) (EC 2.4.1.-)	*Gammaproteobacteria bacterium*		A0A7C5JBS2	--	31911466	Acetyl glucosaminyl transferase activity	Not Available
508	Uncharacterized protein	*Gammaproteobacteria bacterium*		A0A7C1MU14	--	31911466	Not Available	Not Available
509	Poly-beta-1, 6-N-acetyl-D-glucosamine N-deacetylase PgaB (Fragment)	*Gammaproteobacteria bacterium*		A0A3D1S1W2	--	30148503	Hydrolase activity, acting on carbon-nitrogen (but not peptide) bonds	Not Available
510	Poly-beta-1, 6-N-acetyl-D-glucosamine N-deacetylase PgaB	*Gammaproteobacteria bacterium*		A0A7C1XX91	--	31911466	Hydrolase activity, acting on carbon-nitrogen (but not peptide) bonds	Not Available
511	Poly-beta-1, 6-N-acetyl-D-glucosamine synthase (Poly-beta-1, 6-GlcNAc synthase) (EC 2.4.1.-)	*Gammaproteobacteria bacterium*		A0A7C1TYP8	--	31911466	Acetyl glucosaminyl transferase activity	Not Available
512	Uncharacterized protein	*Treponema denticola*	Strain ATCC 35405 / DSM 14222 / CIP 103919 / JCM 8153 / KCTC 15104	Q73JV3	--	15064399	Not Available	Not Available
513	Poly-beta-1, 6-N-acetyl-D-glucosamine biosynthesis protein PgaD	*Gammaproteobacteria bacterium*		A0A7C5JCE7	--	31911466	Not Available	Not Available
514	Poly-beta-1, 6-N-acetyl-D-glucosamine synthase (Poly-beta-1, 6-GlcNAc synthase) (EC 2.4.1.-)	*Metakosakonia spp. MRY16-398*		A0A348DF59	--	30524415	Acetyl glucosaminyl transferase activity	Not Available
515	Poly-beta-1, 6-N-acetyl-D-glucosamine N-deacetylase PgaB (Fragment)	*Gammaproteobacteria bacterium*		A0A7C6AWG6	--	31911466	Hydrolase activity, acting on carbon-nitrogen (but not peptide) bonds	Not Available
516	Poly-beta-1, 6-N-acetyl-D-glucosamine N-deacetylase PgaB	*Gammaproteobacteria bacterium*		A0A7C3CT95	--	31911466	Hydrolase activity, acting on carbon-nitrogen (but not peptide) bonds	Not Available
517	Poly-beta-1, 6-N-acetyl-D-glucosamine synthase (Poly-beta-1, 6-GlcNAc synthase) (EC 2.4.1.-)	*Gammaproteobacteria bacterium*		A0A3D5AXG0	--	30148503	Acetyl glucosaminyl transferase activity	Not Available
518	Poly-beta-1, 6-N-acetyl-D-glucosamine biosynthesis protein PgaD	*Gammaproteobacteria bacterium*		A0A3D5AWF3	--	30148503	Not Available	Not Available
519	Poly-beta-1, 6-N-acetyl-D-glucosamine biosynthesis protein PgaD	*Gammaproteobacteria bacterium*		A0A7C1XYQ8	--	31911466	Not Available	Not Available
520	Fimbrial subunit CupA1	*Pseudomonas aeruginosa*	Strain ATCC 15692 / DSM 22644 / CIP 104116 / JCM 14847 / LMG 12228 / 1C / PRS 101 / PAO1	Q9I1Y7	--	10984043	Not Available	Not Available
521	Poly-beta-1, 6-N-acetyl-D-glucosamine biosynthesis protein PgaD	*Stenotrophomonas spp.*		A0A349Y4K3	--	30148503	Not Available	Not Available
522	Poly-beta-1, 6-N-acetyl-D-glucosamine biosynthesis protein PgaD	*Stenotrophomonas spp.*		A0A356JDL1	--	30148503	Not Available	Not Available
523	Poly-beta-1, 6-N-acetyl-D-glucosamine biosynthesis protein PgaD	*Deltaproteobacteria bacterium RBG_13_60_28*		A0A1F9BIK0	--	27774985	Not Available	Not Available
524	Surface composition regulator	*Shigella flexneri serotype X*	Strain 2002017	D2A7N5	--	19955273	Not Available	Not Available
525	GGDEF domain-containing protein	*Pseudomonas aeruginosa*	Strain ATCC 15692 / DSM 22644 / CIP 104116 / JCM 14847 / LMG 12228 / 1C / PRS 101 / PAO1	Q9I6W3	--	10984043	Diguanylate cyclase activity	Not Available
526	GGDEF domain-containing protein	*Pseudomonas aeruginosa*	Strain ATCC 15692 / DSM 22644 / CIP 104116 / JCM 14847 / LMG 12228 / 1C / PRS 101 / PAO1	Q9HYQ2	--	10984043	Diguanylate cyclase activity	Not Available
527	GGDEF domain-containing protein	*Pseudomonas aeruginosa*	Strain ATCC 15692 / DSM 22644 / CIP 104116 / JCM 14847 / LMG 12228 / 1C / PRS 101 / PAO1	Q9I4M8	--	10984043	Diguanylate cyclase activity	Not Available

Target ID	Bacterial Protein Name	Organism Name	Strain	UniProt ID	PDB ID	PubMed ID	Classification as per UniProt GO Molecular Function	Classification of Function as per PDB
528	Exodeoxyribonuclease III (EC 3.1.11.2)	*Pseudomonas aeruginosa*	Strain ATCC 15692 / DSM 22644 / CIP 104116 / JCM 14847 / LMG 12228 / 1C / PRS 101 / PAO1	G3XDB0	--	10984043	DNA-(apurinic or apyrimidinic site) endonuclease activity, double-stranded DNA 3'-5' exodeoxyribonuclease activity, exodeoxyribonuclease III activity, phosphoric diester Hydrolase activity	Not Available
529	Fimbrial domain-containing protein	*Pseudomonas aeruginosa*	Strain ATCC 15692 / DSM 22644 / CIP 104116 / JCM 14847 / LMG 12228 / 1C / PRS 101 / PAO1	Q9HTS0	--	10984043	Not Available	Not Available
530	Probable fimbrial subunit CupB1	*Pseudomonas aeruginosa*	Strain ATCC 15692 / DSM 22644 / CIP 104116 / JCM 14847 / LMG 12228 / 1C / PRS 101 / PAO1	Q9HWU2	--	10984043	Not Available	Not Available
531	Fimbrial subunit CupC1	*Pseudomonas aeruginosa*	Strain ATCC 15692 / DSM 22644 / CIP 104116 / JCM 14847 / LMG 12228 / 1C / PRS 101 / PAO1	Q9I4X7	--	10984043	Not Available	Not Available
532	GGDEF domain-containing protein	*Pseudomonas aeruginosa*	Strain ATCC 15692 / DSM 22644 / CIP 104116 / JCM 14847 / LMG 12228 / 1C / PRS 101 / PAO1	Q9HZ57	--	10984043	Diguanylate cyclase activity	Not Available
533	50S ribosomal subunit assembly factor BipA (EC 3.6.5.-) (GTP-binding protein BipA)	*Pseudomonas aeruginosa*	Strain ATCC 15692 / DSM 22644 / CIP 104116 / JCM 14847 / LMG 12228 / 1C / PRS 101 / PAO1	Q9HU67	--	10984043	GTP binding, GTPase activity, ribosome binding, rRNA binding, tRNA binding	Not Available
534	Uncharacterized protein	*Pseudomonas aeruginosa*	Strain ATCC 15692 / DSM 22644 / CIP 104116 / JCM 14847 / LMG 12228 / 1C / PRS 101 / PAO1	Q9I6K0	--	10984043	Diguanylate cyclase activity	Not Available
535	Regulatory protein RsaL	*Pseudomonas aeruginosa*	Strain ATCC 15692 / DSM 22644 / CIP 104116 / JCM 14847 / LMG 12228 / 1C / PRS 101 / PAO1	G3XD78	--	10984043	DNA binding, DNA-binding Transcription repressor activity, Transcription cis-regulatory region binding	Not Available
536	Uncharacterized protein	*Xylella fastidiosa*	Strain M23	B2I812	--	20601474	Not Available	Not Available
537	Uncharacterized protein	*Pseudomonas spp. GM79*		J2WV44	--	23045501	Not Available	Not Available
538	Poly-beta-1, 6-N-acetyl-D-glucosamine biosynthesis protein PgaD	*Pseudomonas spp. GM79*		J3DHL5	--	23045501	Not Available	Not Available
539	Biofilm PGA synthesis deacetylase PgaB	*Acinetobacter baumannii NCGM 237*		A0A0E1PPJ3	--	24550340	Hydrolase activity, acting on carbon-nitrogen (but not peptide) bonds	Not Available
540	Poly-beta-1, 6-N-acetyl-D-glucosamine synthase (Poly-beta-1, 6-GlcNAc synthase) (EC 2.4.1.-)	*Acinetobacter baumannii NCGM 237*		A0A0E1PT59	--	24550340	Acetyl glucosaminyl transferase activity	Not Available
541	Poly-beta-1, 6-N-acetyl-D-glucosamine N-deacetylase PgaB	*Acinetobacter baumannii NCGM 237*		A0A0E1PVR4	--	24550340	Hydrolase activity, acting on carbon-nitrogen (but not peptide) bonds	Not Available
542	Poly-beta-1, 6-N-acetyl-D-glucosamine auxiliary protein PgaD	*Acinetobacter baumannii NCGM 237*		A0A0E1PU81	--	24550340	Not Available	Not Available
543	PgaB	*Pseudomonas ogarae*	Strain DSM 112162 / CECT 30235 / F113	G8PY99	--	22328765	Hydrolase activity, acting on carbon-nitrogen (but not peptide) bonds	Not Available
544	PgaD	*Pseudomonas ogarae*	Strain DSM 112162 / CECT 30235 / F113	G8PYA1	--	22328765	Not Available	Not Available
545	Poly-beta-1, 6-N-acetyl-D-glucosamine synthase (Poly-beta-1, 6-GlcNAc synthase) (EC 2.4.1.-)	*Pseudomonas ogarae*	Strain DSM 112162 / CECT 30235 / F113	G8PYA0	--	22328765	Acetyl glucosaminyl transferase activity	Not Available
546	Poly-beta-1, 6-N-acetyl-D-glucosamine synthase (Poly-beta-1, 6-GlcNAc synthase) (EC 2.4.1.-)	*Acinetobacter baumannii NCGM 237*		A0A0E1PS38	--	24550340	Acetyl glucosaminyl transferase activity	Not Available
547	Toxin-antitoxin system	*Pseudomonas ogarae*	Strain DSM 112162 / CECT 30235 / F113	G8Q9Z1	--	22328765	Not Available	Not Available
548	Poly-beta-1, 6-N-acetyl-D-glucosamine synthase (Poly-beta-1, 6-GlcNAc synthase) (EC 2.4.1.-)	*Candidatus Competibacteraceae bacterium*		A0A433I7B0	--	30578268	Acetyl glucosaminyl transferase activity	Not Available
549	Poly-beta-1, 6-N-acetyl-D-glucosamine biosynthesis protein PgaD	*Candidatus Competibacteraceae bacterium*		A0A3S0WCV8	--	30578268	Not Available	Not Available
550	Poly-beta-1, 6-N-acetyl-D-glucosamine biosynthesis protein PgaD	*Pseudomonas spp. GM24*		J2QNC2	--	23045501	Not Available	Not Available
551	Poly-beta-1, 6-N-acetyl-D-glucosamine N-deacetylase PgaB	*Candidatus Competibacteraceae bacterium*		A0A3S0W208	--	30578268	Hydrolase activity, acting on carbon-nitrogen (but not peptide) bonds	Not Available
552	Poly-beta-1, 6-N-acetyl-D-glucosamine synthase (Poly-beta-1, 6-GlcNAc synthase) (EC 2.4.1.-)	*Pseudomonas spp. GM24*		J3FT73	--	23045501	Acetyl glucosaminyl transferase activity	Not Available
553	Biofilm PGA synthesis auxiliary protein PgaD	*Acinetobacter baumannii NCGM 237*		A0A0E1PVN7	--	24550340	Not Available	Not Available
554	Poly-beta-1, 6-N-acetyl-D-glucosamine N-deacetylase PgaB	*Pseudomonas spp. GM24*		J2R4Q4	--	23045501	Hydrolase activity, acting on carbon-nitrogen (but not peptide) bonds	Not Available
555	Uncharacterized protein	*Kangiella koreensis*	Strain DSM 16069 / KCTC 12182 / SW-125	C7RBF2	--	21304661	Not Available	Not Available
556	Uncharacterized protein	*Pseudomonas spp. TCU-HL1*		A0A1B3E8H3	--	27542933	Not Available	Not Available
557	Uncharacterized protein	*Candidatus Lambdaproteobacteria bacterium RIFOXYD2_FULL_56_26*		A0A1F6H0I8	--	27774985	Not Available	Not Available
558	Poly-beta-1, 6-N-acetyl-D-glucosamine synthase (Poly-beta-1, 6-GlcNAc synthase) (EC 2.4.1.-)	*Acinetobacter spp. ACNIH2*		A0A2L0HWN6	--	29437920	Acetyl glucosaminyl transferase activity	Not Available
559	Poly-beta-1, 6-N-acetyl-D-glucosamine synthase (Poly-beta-1, 6-GlcNAc synthase) (EC 2.4.1.-)	*Carnobacterium divergens*	Lactobacillus divergens	A0A7I0FF87	--	30081251	Acetyl glucosaminyl transferase activity	Not Available
560	Poly-beta-1, 6-N-acetyl-D-glucosamine synthase (Poly-beta-1, 6-GlcNAc synthase) (EC 2.4.1.-)	*Lysobacter enzymogenes*		A0A1J1EEU3	--	28065880	Acetyl glucosaminyl transferase activity	Not Available

Target ID	Bacterial Protein Name	Organism Name	Strain	UniProt ID	PDB ID	PubMed ID	Classification as per UniProt GO Molecular Function	Classification of Function as per PDB
561	HmsF protein	*Lysobacter enzymogenes*		A0A1J1EKH2	--	28065880	Hydrolase activity, acting on carbon-nitrogen (but not peptide) bonds	Not Available
562	Motility quorum-sensing regulator MqsR	*Pseudomonas moraviensis R28-S*		V8R8K1	--	24558233	Not Available	Not Available
563	Surface composition regulator	*Salmonella enterica subspp. enterica serovar*	Senftenberg Str. A4-543	G5R5Q1	--	21859443	Not Available	Not Available
564	Polysaccharide deacetylase	*Kangiella koreensis*	Strain DSM 16069 / KCTC 12182 / SW-125	C7RBF0	--	21304661	Hydrolase activity, acting on carbon-nitrogen (but not peptide) bonds	Not Available
565	Uncharacterized protein	*Pseudomonas spp. TCU-HL1*		A0A1B3E8H9	--	27542933	Not Available	Not Available
566	Poly-beta-1, 6-N-acetyl-D-glucosamine biosynthesis protein PgaD	*Acinetobacter spp. ACNIH2*		A0A2L0HWJ0	--	29437920	Not Available	Not Available
567	Uncharacterized protein	*Achromobacter xylosoxidans*	Strain A8	E3HKX9	--	21097610	Not Available	Not Available
568	Uncharacterized protein	*Thioalkalivibrio sulfidiphilus*	Strain HL-EbGR7	B8GTH7	--	21475584	Not Available	Not Available
569	Poly-beta-1, 6-N-acetyl-D-glucosamine biosynthesis protein PgaD	*Variovorax spp. CF313*		J2JIW7	--	23045501	Not Available	Not Available
570	Poly-beta-1, 6-N-acetyl-D-glucosamine synthase (Poly-beta-1, 6-GlcNAc synthase) (EC 2.4.1.-)	*Ralstonia solanacearum*	Strain GMI1000 Pseudomonas solanacearum	Q8XT27	--	11823852	Acetyl glucosaminyl transferase activity	Not Available
571	Poly-beta-1, 6-N-acetyl-D-glucosamine biosynthesis protein PgaD	*Serratia marcescens*		A0A1L6QPC1	--	28536292	Not Available	Not Available
572	Poly-beta-1, 6-N-acetyl-D-glucosamine N-deacetylase PgaB	*Variovorax spp. CF313*		J2SQM8	--	23045501	Hydrolase activity, acting on carbon-nitrogen (but not peptide) bonds	Not Available
573	GGDEF family protein	*Leptospira interrogans serogroup Icterohaemorrhagiae serovar Lai*	Strain 56601	Q8F244	--	12712204	Diguanylate cyclase activity	Not Available
574	Response regulator with GGDEF domain	*Leptospira interrogans serogroup Icterohaemorrhagiae serovar Lai*	Strain 56601	Q8EZC6	--	12712204	Diguanylate cyclase activity	Not Available
575	Poly-beta-1, 6-N-acetyl-D-glucosamine biosynthesis protein PgaD	*Enterobacteriaceae bacterium*		A0A3C0H2G8	--	30148503	Not Available	Not Available
576	Uncharacterized protein	*Enterobacteriaceae bacterium*		A0A3C2BJ06	--	30148503	Not Available	Not Available
577	Poly-beta-1, 6-N-acetyl-D-glucosamine biosynthesis protein PgaD	*Xenorhabdus kozodoii*		A0A2D0LH81	--	28993611	Not Available	Not Available
578	GGDEF family protein	*Leptospira interrogans serogroup Icterohaemorrhagiae serovar Lai*	Strain 56601	Q8F251	--	12712204	Diguanylate cyclase activity	Not Available
579	Poly-beta-1, 6-N-acetyl-D-glucosamine synthase (Poly-beta-1, 6-GlcNAc synthase) (EC 2.4.1.-)	*Xenorhabdus kozodoii*		A0A2D0LIF9	--	28993611	Acetyl glucosaminyl transferase activity	Not Available
580	Toxin-antitoxin system, toxin component	*Enterobacteriaceae bacterium*		A0A3C0H4J1	--	30148503	Not Available	Not Available
581	Poly-beta-1, 6-N-acetyl-D-glucosamine N-deacetylase PgaB	*Enterobacteriaceae bacterium*		A0A3C2BHH0	--	30148503	Hydrolase activity, acting on carbon-nitrogen (but not peptide) bonds	Not Available
582	GGDEF family protein	*Leptospira interrogans serogroup Icterohaemorrhagiae serovar Lai*	Strain 56601	Q8F248	--	12712204	Diguanylate cyclase activity	Not Available
583	GGDEF family protein	*Leptospira interrogans serogroup Icterohaemorrhagiae serovar Lai*	Strain 56601	Q8F245	--	12712204	Diguanylate cyclase activity	Not Available
584	Uncharacterized protein	*Xylella fastidiosa*	Strain Temecula1 / ATCC 700964	Q87EE2	--	12533478	Not Available	Not Available
585	Poly-beta-1, 6-N-acetyl-D-glucosamine biosynthesis protein PgaD	*Enterobacteriaceae bacterium*		A0A3C2BGH1	--	30148503	Not Available	Not Available
586	GGDEF domain receiver component of a two component response regulator	*Leptospira interrogans serogroup Icterohaemorrhagiae serovar Lai*	Strain 56601	Q8F383	--	12712204	Diguanylate cyclase activity	Not Available
587	GGDEF family protein	*Leptospira interrogans serogroup Icterohaemorrhagiae serovar Lai*	Strain 56601	Q8EXH2	--	12712204	Diguanylate cyclase activity	Not Available
588	Surface composition regulator	*Cronobacter turicensis*	Strain DSM 18703 / CCUG 55852 / LMG 23827 / z3032	C9Y2V2	--	21037008	Not Available	Not Available
589	GGDEF family protein	*Leptospira interrogans serogroup Icterohaemorrhagiae serovar Lai*	Strain 56601	Q8F246	--	12712204	Diguanylate cyclase activity	Not Available
590	GGDEF family protein	*Leptospira interrogans serogroup Icterohaemorrhagiae serovar Lai*	Strain 56601	Q8F247	--	12712204	Diguanylate cyclase activity	Not Available
591	Poly-beta-1, 6-N-acetyl-D-glucosamine N-deacetylase PgaB	*Enterobacteriaceae bacterium*		A0A3C0H2V4	--	30148503	Hydrolase activity, acting on carbon-nitrogen (but not peptide) bonds	Not Available
592	Poly-beta-1, 6-N-acetyl-D-glucosamine biosynthesis protein PgaD	*Enterobacteriaceae bacterium*		A0A351NN88	--	30148503	Not Available	Not Available
593	Poly-beta-1, 6-N-acetyl-D-glucosamine N-deacetylase PgaB	*Enterobacteriaceae bacterium*		A0A351NN90	--	30148503	Hydrolase activity, acting on carbon-nitrogen (but not peptide) bonds	Not Available
594	Polysaccharide deacetylase	*Xenorhabdus kozodoii*		A0A2D0LH91	--	28993611	Hydrolase activity, acting on carbon-nitrogen (but not peptide) bonds	Not Available
595	Sensory box / GGDEF family protein	*Bacillus cereus*	Strain ATCC 14579 / DSM 31 / CCUG 7414 / JCM 2152 / NBRC 15305 / NCIMB 9373 / NCTC 2599 / NRRL B-3711	Q819E1	--	12721630	Diguanylate cyclase activity	Not Available
596	Poly-beta-1, 6-N-acetyl-D-glucosamine biosynthesis protein PgaD	*Enterobacter cloacae complex spp. ECNIH11*		A0A2S4SKF2	--	29437920	Not Available	Not Available
597	Poly-beta-1, 6-N-acetyl-D-glucosamine synthase (Poly-beta-1, 6-GlcNAc synthase) (EC 2.4.1.-)	*Enterobacteriaceae bacterium*		A0A351NN89	--	30148503	Acetyl glucosaminyl transferase activity	Not Available
598	Biofilm PGA synthesis deacetylase PgaB	*Paraburkholderia caribensis MBA4*		A0A0P0RAM0	--	24558235	Hydrolase activity, acting on carbon-nitrogen (but not peptide) bonds	Not Available

Target ID	Bacterial Protein Name	Organism Name	Strain	UniProt ID	PDB ID	PubMed ID	Classification as per UniProt GO Molecular Function	Classification of Function as per PDB
599	GGDEF family protein	*Leptospira interrogans serogroup Icterohaemorrhagiae serovar Lai*	Strain 56601	Q8EXH5	--	12712204	Diguanylate cyclase activity	Not Available
600	GGDEF family protein	*Leptospira interrogans serogroup Icterohaemorrhagiae serovar Lai*	Strain 56601	Q8F249	--	12712204	Diguanylate cyclase activity	Not Available
601	Poly-beta-1, 6-N-acetyl-D-glucosamine synthase (Poly-beta-1, 6-GlcNAc synthase) (EC 2.4.1.-)	*Aquitalea magnusonii*		A0A3G9GKV2	--	28293292	Acetyl glucosaminyl transferase activity	Not Available
602	Poly-beta-1, 6-N-acetyl-D-glucosamine biosynthesis protein PgaD	*Xanthomonas vasicola pv. zeae*		A0A7C7F7Z7	--	32040377	Not Available	Not Available
603	Biofilm PGA synthesis deacetylase PgaB	*Aquitalea magnusonii*		A0A3G9GKG4	--	28293292	Hydrolase activity, acting on carbon-nitrogen (but not peptide) bonds	Not Available
604	Biofilm PGA synthesis protein PgaD	*Luteimona cucumeris*		A0A562L7U6	--	26203337	Not Available	Not Available
605	Poly-beta-1, 6-N-acetyl-D-glucosamine N-deacetylase PgaB	*Xanthomonas vasicola pv. zeae*		A0A7C7B1H5	--	32040377	Hydrolase activity, acting on carbon-nitrogen (but not peptide) bonds	Not Available
606	Poly-beta-1, 6-N-acetyl-D-glucosamine N-deacetylase PgaB (EC 3.5.1.-)	*Pseudomonas spp. WS 5503*		A0A7Y0WES9	--	32587583	Hydrolase activity, acting on carbon-nitrogen (but not peptide) bonds	Not Available
607	Poly-beta-1, 6-N-acetyl-D-glucosamine biosynthesis protein PgaD	*Pseudomonas spp. WS 5503*		A0A7Y0WEE3	--	32587583	Not Available	Not Available
608	HmsF protein	*Xanthomonas axonopodis pv. citri*	Strain 306	Q8PLI6	--	12024217	Hydrolase activity, acting on carbon-nitrogen (but not peptide) bonds	Not Available
609	GGDEF domain-containing protein	*Rhodopirellula baltica*	Strain DSM 10527 / NCIMB 13988 / SH1	Q7UXF6	--	12835416	Diguanylate cyclase activity	Not Available
610	Probable response regulatory protein	*Rhodopirellula baltica*	Strain DSM 10527 / NCIMB 13988 / SH1	Q7UF69	--	12835416	Diguanylate cyclase activity	Not Available
611	Uncharacterized protein	*Xanthomonas axonopodis pv. citri*	Strain 306	Q8PLI8	--	12024217	Not Available	Not Available
612	Probable response regulatory protein	*Rhodopirellula baltica*	Strain DSM 10527 / NCIMB 13988 / SH1	Q7UR09	--	12835416	Diguanylate cyclase activity	Not Available
613	Poly-beta-1, 6-N-acetyl-D-glucosamine biosynthesis protein PgaD	*Tatumella spp. OPLPL6*		A0A2G8BYZ7	--	29067021	Not Available	Not Available
614	Poly-beta-1, 6-N-acetyl-D-glucosamine synthase (Poly-beta-1, 6-GlcNAc synthase) (EC 2.4.1.-)	*Enterobacter cloacae complex spp. ECNIH11*		A0A2S4SKE8	--	29437920	Acetyl glucosaminyl transferase activity	Not Available
615	Poly-beta-1, 6-N-acetyl-D-glucosamine N-deacetylase PgaB	*Enterobacter cloacae complex spp. ECNIH11*		A0A2S4SKH4	--	29437920	Hydrolase activity, acting on carbon-nitrogen (but not peptide) bonds	Not Available
616	Conserved hypothetical Membrane protein	*Pseudomonas fluorescens*	Strain SBW25	C3KAG0	--	19432983	Not Available	Not Available
617	Putative lipoprotein	*Pseudomonas fluorescens*	Strain SBW25	C3KAF8	--	19432983	Hydrolase activity, acting on carbon-nitrogen (but not peptide) bonds	Not Available
618	Poly-beta-1, 6-N-acetyl-D-glucosamine synthase (Poly-beta-1, 6-GlcNAc synthase) (EC 2.4.1.-) (Fragment)	*Lentisphaerae bacterium GWF2_44_16*		A0A1G0YRV0	--	27774985	Acetyl glucosaminyl transferase activity	Not Available
619	Surface composition regulator	*Enterobacter cloacae complex spp. ECNIH11*		A0A2S4SUC2	--	29437920	Not Available	Not Available
620	Haemin storage system, HmsF protein	*Yersinia pseudotuberculosis serotype I*	Strain IP32953	Q66B30	--	15358858	Hydrolase activity, acting on carbon-nitrogen (but not peptide) bonds	Not Available
621	Poly-beta-1, 6-N-acetyl-D-glucosamine synthase (Poly-beta-1, 6-GlcNAc synthase) (EC 2.4.1.-)	*Luteibacter yeojuensis*		A0A7X5QS05	--	16957102	Acetyl glucosaminyl transferase activity	Not Available
622	Poly-beta-1, 6-N-acetyl-D-glucosamine N-deacetylase PgaB	*Luteibacter yeojuensis*		A0A7X5QS01	--	16957102	Hydrolase activity, acting on carbon-nitrogen (but not peptide) bonds	Not Available
623	Surface composition regulator	*Serratia plymuthica S13*		S4YI83	--	23929484	Not Available	Not Available
624	Biofilm PGA synthesis lipoprotein PgaB	*Luteimona cucumeris*		A0A562L7K0	--	26203337	Hydrolase activity, acting on carbon-nitrogen (but not peptide) bonds	Not Available
625	PgaB protein	*Escherichia coli*	Strain 55989 / EAEC	B7LFD3	--	19165319	Hydrolase activity, acting on carbon-nitrogen (but not peptide) bonds	Not Available
626	Poly-beta-1, 6-N-acetyl-D-glucosamine biosynthesis protein PgaD	*Luteibacter yeojuensis*		A0A7X5QS23	--	16957102	Not Available	Not Available
627	Poly-beta-1, 6-N-acetyl-D-glucosamine synthase (Poly-beta-1, 6-GlcNAc synthase) (EC 2.4.1.-)	*Escherichia coli O6:K15:H31*	Strain 536 / UPEC	A0A454A2Y6	--	16879640	Acetyl glucosaminyl transferase activity	Not Available
628	Haemin storage system, HmsS protein	*Yersinia pseudotuberculosis serotype I*	Strain IP32953	Q66B28	--	15358858	Not Available	Not Available
629	Biofilm PGA synthesis lipoprotein PgaB (EC 3.-.-.-)	*Klebsiella pneumoniae*	Strain 342	B5Y382	--	18654632	Hydrolase activity, acting on carbon-nitrogen (but not peptide) bonds	Not Available
630	Poly-beta-1, 6-N-acetyl-D-glucosamine synthase (Poly-beta-1, 6-GlcNAc synthase) (EC 2.4.1.-)	*Escherichia coli*	Strain 55989 / EAEC	B7LFD2	--	19165319	Acetyl glucosaminyl transferase activity	Not Available
631	Surface composition regulator	*Klebsiella pneumoniae*	Strain 342	B5XU41	--	18654632	Not Available	Not Available
632	Poly-beta-1, 6-N-acetyl-D-glucosamine N-deacetylase PgaB (EC 3.5.1.-)	*Pseudomonas spp. WS 5027*		A0A7Y1B5A9	--	32587583	Hydrolase activity, acting on carbon-nitrogen (but not peptide) bonds	Not Available
633	Putative biofilm PGA synthesis protein PgaD	*Klebsiella pneumoniae*	Strain 342	B5Y384	--	18654632	Not Available	Not Available
634	Poly-beta-1, 6-N-acetyl-D-glucosamine synthase (Poly-beta-1, 6-GlcNAc synthase) (EC 2.4.1.-)	*Luteimona cucumeris*		A0A562L7D7	--	26203337	Acetyl glucosaminyl transferase activity	Not Available
635	Poly-beta-1, 6-N-acetyl-D-glucosamine synthase (Poly-beta-1, 6-GlcNAc synthase) (EC 2.4.1.-)	*Xanthomonas vasicola pv. zeae*		A0A7C7B0Q2	--	32040377	Acetyl glucosaminyl transferase activity	Not Available
636	Poly-beta-1, 6-N-acetyl-D-glucosamine synthase (Poly-beta-1, 6-GlcNAc synthase) (EC 2.4.1.-)	*Klebsiella pneumoniae*	Strain 342	B5Y383	--	18654632	Acetyl glucosaminyl transferase activity	Not Available
637	Poly-beta-1, 6-N-acetyl-D-glucosamine N-deacetylase PgaB	*Acinetobacter cumulans*		A0A4P8DU29	--	30808586	Hydrolase activity, acting on carbon-nitrogen (but not peptide) bonds	Not Available
638	Poly-beta-1, 6-N-acetyl-D-glucosamine biosynthesis protein PgaD	*Candidatus Muproteobacteria bacterium RBG_19FT_COMBO_61_10*		A0A1F6UP26	--	27774985	Not Available	Not Available
639	Surface composition regulator	*Cronobacter malonaticus*		A0A423XSW2	--	30416493	Not Available	Not Available
640	Poly-beta-1, 6-N-acetyl-D-glucosamine synthase (Poly-beta-1, 6-GlcNAc synthase) (EC 2.4.1.-)	*Acinetobacter baumannii*	Strain AB0057	A0A7U4DG52	--	18931120	Acetyl glucosaminyl transferase activity	Not Available
641	Surface composition regulator	*Cronobacter malonaticus*		V5U106	--	24435860	Not Available	Not Available
642	Uncharacterized protein	*Syntrophobacter spp. DG_60*		A0A0S7XQF1	--	25922666	Not Available	Not Available
643	mRNA interferase MqsR	*Paraburkholderia madseniana*		A0A6N6W8U5	--	32027304	Not Available	Not Available
644	Poly-beta-1, 6-N-acetyl-D-glucosamine synthase (Poly-beta-1, 6-GlcNAc synthase) (EC 2.4.1.-)	*Sulfurimona spp. RIFOXYD2_FULL_34_21*		A0A1G3UF66	--	27774985	Acetyl glucosaminyl transferase activity	Not Available
645	Fimbrial protein	*Yersinia pestis*		A0A384KL23	--	11586360	Not Available	Not Available
646	Poly-beta-1, 6-N-acetyl-D-glucosamine synthase (Poly-beta-1, 6-GlcNAc synthase) (EC 2.4.1.-)	*Citrobacter freundii complex spp. CFNIH4*		A0A2L0TFK3	--	29437920	Acetyl glucosaminyl transferase activity	Not Available

Target ID	Bacterial Protein Name	Organism Name	Strain	UniProt ID	PDB ID	PubMed ID	Classification as per UniProt GO Molecular Function	Classification of Function as per PDB
647	Hypothetical lipoprotein YcdR	*Escherichia coli*	Strain UTI89 / UPEC	Q1RDJ4	--	16585510	Hydrolase activity, acting on carbon-nitrogen (but not peptide) bonds	Not Available
648	Poly-beta-1, 6-N-acetyl-D-glucosamine biosynthesis protein PgaD	*Citrobacter freundii complex spp. CFNIH4*		A0A2L0TFH9	--	29437920	Not Available	Not Available
649	Poly-beta-1, 6-N-acetyl-D-glucosamine N-deacetylase PgaB	*Sulfurimona spp. RIFOXYD2_FULL_34_21*		A0A1G3UF37	--	27774985	Hydrolase activity, acting on carbon-nitrogen (but not peptide) bonds	Not Available
650	Surface composition regulator	*Citrobacter freundii complex spp. CFNIH4*		A0A2L0TH79	--	29437920	Not Available	Not Available
651	Poly-beta-1, 6-N-acetyl-D-glucosamine synthase (Poly-beta-1, 6-GlcNAc synthase) (EC 2.4.1.-)	*Yersinia pestis*		Q0WFJ6	--	11586360	Acetyl glucosaminyl transferase activity	Not Available
652	Uncharacterized protein	*Escherichia coli*	Strain UTI89 / UPEC	Q1RDJ6	--	16585510	Not Available	Not Available
653	Poly-beta-1, 6-N-acetyl-D-glucosamine N-deacetylase	*Citrobacter freundii complex spp. CFNIH4*		A0A2L0TG22	--	29437920	Hydrolase activity, acting on carbon-nitrogen (but not peptide) bonds	Not Available
654	Putative motility quorum-sensing regulator	*Magnetospirillum gryphiswaldense*	Strain DSM 6361 / JCM 21280 / NBRC 15271 / MSR-1	V6F2G1	--	24625872	Not Available	Not Available
655	Poly-beta-1, 6-N-acetyl-D-glucosamine biosynthesis protein PgaD	*Sulfurimona spp. RIFOXYD2_FULL_34_21*		A0A1G3UG59	--	27774985	Not Available	Not Available
656	Hypothetical cytosolic protein	*Syntrophus aciditrophicus*	Strain SB	Q2LYD0	--	17442750	Not Available	Not Available
657	Poly-beta-1, 6-N-acetyl-D-glucosamine synthase (Poly-beta-1, 6-GlcNAc synthase) (EC 2.4.1.-)	*Alcaligenaceae bacterium*		A0A7C6NLD6	--	32123542	Acetyl glucosaminyl transferase activity	Not Available
658	Poly-beta-1, 6-N-acetyl-D-glucosamine biosynthesis protein PgaD	*Alcaligenaceae bacterium*		A0A7C6NL24	--	32123542	Not Available	Not Available
659	Putative membrane protein	*Xanthomonas campestris pv. campestris*	Strain B100	A0A1X7QFS0	--	18304669	Not Available	Not Available
660	Uncharacterized protein	*Rhodanobacter spp. 115*		I4VP81	--	22843592	Not Available	Not Available
661	Poly-beta-1, 6-N-acetyl-D-glucosamine synthase (Poly-beta-1, 6-GlcNAc synthase) (EC 2.4.1.-)	*Pasteurellaceae bacterium*		A0A356E647	--	30148503	Acetyl glucosaminyl transferase activity	Not Available
662	Poly-beta-1, 6-N-acetyl-D-glucosamine biosynthesis protein PgaD	*Alcaligenaceae bacterium*		A0A2D8B7A5	--	29337314	Not Available	Not Available
663	Poly-beta-1, 6-N-acetyl-D-glucosamine N-deacetylase PgaB (Fragment)	*Pasteurellaceae bacterium*		A0A356EBH6	--	30148503	Hydrolase activity, acting on carbon-nitrogen (but not peptide) bonds	Not Available
664	Poly-beta-1, 6-N-acetyl-D-glucosamine synthase (Poly-beta-1, 6-GlcNAc synthase) (EC 2.4.1.-)	*Alcaligenaceae bacterium*		A0A2D8B791	--	29337314	Acetyl glucosaminyl transferase activity	Not Available
665	Poly-beta-1, 6-N-acetyl-D-glucosamine N-deacetylase PgaB (EC 3.5.1.-)	*Alcaligenaceae bacterium*		A0A7C6NPE6	--	32123542	Hydrolase activity, acting on carbon-nitrogen (but not peptide) bonds	Not Available
666	Poly-beta-1, 6-N-acetyl-D-glucosamine synthase (Poly-beta-1, 6-GlcNAc synthase) (EC 2.4.1.-)	*Alcaligenes spp. EGD-AK7*		U1YHE7	--	24407646	Acetyl glucosaminyl transferase activity	Not Available
667	Poly-beta-1, 6-N-acetyl-D-glucosamine synthase (Poly-beta-1, 6-GlcNAc synthase) (EC 2.4.1.-)	*Pseudomonas trivialis*		A0A0H5A7V6	--	26337878	Acetyl glucosaminyl transferase activity	Not Available
668	Outer membrane N-deacetylase	*Pseudomonas trivialis*		A0A0H5ABT7	--	26337878	Hydrolase activity, acting on carbon-nitrogen (but not peptide) bonds	Not Available
669	Motility quorum-sensing regulator MqsR	*Pseudomonas trivialis*		A0A0H5A8B6	--	26337878	Not Available	Not Available
670	Uncharacterized protein	*Sulfurimona denitrificans*	Strain ATCC 33889 / DSM 1251 Thiomicrospira denitrificans Strain ATCC 33889 / DSM 1251	Q30SJ9	--	18065616	Not Available	Not Available
671	Uncharacterized protein	*Alcaligenes spp. EGD-AK7*		U1XZ69	--	24407646	Not Available	Not Available
672	Poly-beta-1, 6-N-acetyl-D-glucosamine synthase (Poly-beta-1, 6-GlcNAc synthase) (EC 2.4.1.-)	*Sulfurimona denitrificans*	Strain ATCC 33889 / DSM 1251 Thiomicrospira denitrificans Strain ATCC 33889 / DSM 1251	Q30SK0	--	18065616	Acetyl glucosaminyl transferase activity	Not Available
673	Polysaccharide deacetylase	*Sulfurimona denitrificans*	Strain ATCC 33889 / DSM 1251 Thiomicrospira denitrificans Strain ATCC 33889 / DSM 1251	Q30SK1	--	18065616	Hydrolase activity, acting on carbon-nitrogen (but not peptide) bonds	Not Available
674	Biofilm PGA synthesis protein PgaD	*Pseudomonas trivialis*		A0A0H5AAQ8	--	26337878	Not Available	Not Available
675	Outer membrane N-deacetylase	*Alcaligenes spp. EGD-AK7*		U1ZPF8	--	24407646	Hydrolase activity, acting on carbon-nitrogen (but not peptide) bonds	Not Available
676	Uncharacterized protein	*Pediococcus damnosus*		A0A0R2HGU2	--	26415554	Not Available	Not Available
677	Biofilm PGA synthesis lipoprotein PgaB	*Xanthomonas oryzae pv. oryzae*	Strain PXO99A	A0A0K0GHF3	--	18452608	Hydrolase activity, acting on carbon-nitrogen (but not peptide) bonds	Not Available
678	Poly-beta-1, 6-N-acetyl-D-glucosamine biosynthesis protein PgaD	*Pseudomonas spp. S04*		A0A6I6SR34	--	25398872	Not Available	Not Available
679	Putative toxin-antitoxin system, toxin component	*Bordetella pertussis STO1-CHOC-0019*		A0A6N3RY39	--	24356839	Not Available	Not Available
680	Poly-beta-1, 6-N-acetyl-D-glucosamine synthase (Poly-beta-1, 6-GlcNAc synthase) (EC 2.4.1.-)	*Pseudomonas spp. S04*		A0A6I6SST5	--	25398872	Acetyl glucosaminyl transferase activity	Not Available
681	Surface composition regulator	*Yersinia pestis bv. Antiqua*	Strain Antiqua	A0A0E1NXP9	--	16740952	DNA binding, DNA-directed DNA polymerase activity	Not Available
682	Poly-beta-1, 6-N-acetyl-D-glucosamine synthase (Poly-beta-1, 6-GlcNAc synthase) (EC 2.4.1.-)	*Methylomona methanica*	Strain MC09	G0A376	--	22123758	Acetyl glucosaminyl transferase activity	Not Available
683	Poly-beta-1, 6-N-acetyl-D-glucosamine N-deacetylase PgaB (EC 3.5.1.-)	*Bordetella pertussis STO1-CHOC-0019*		A0A6N3RXJ4	--	24356839	Hydrolase activity, acting on carbon-nitrogen (but not peptide) bonds	Not Available
684	Uncharacterized protein	*Methylomona methanica*	Strain MC09	G0A375	--	22123758	Not Available	Not Available
685	Poly-beta-1, 6-N-acetyl-D-glucosamine synthase (Poly-beta-1, 6-GlcNAc synthase) (EC 2.4.1.-)	*Lysobacter gummosus*		A0A0S2G4Y6	--	26597042	Acetyl glucosaminyl transferase activity	Not Available
686	Haemin storage system, HmsF protein	*Yersinia pestis bv. Antiqua*	Strain Antiqua	A0A0E1NRK0	--	16740952	Hydrolase activity, acting on carbon-nitrogen (but not peptide) bonds	Not Available
687	GGDEF family protein	*Coxiella burnetii*	Strain RSA 493 / Nine Mile phaSe I	Q83A76	--	12704232; 19047403	Diguanylate cyclase activity	Not Available
688	Poly-beta-1, 6-N-acetyl-D-glucosamine N-deacetylase	*Pseudomonas spp. S04*		A0A6I6SX07	--	25398872	Hydrolase activity, acting on carbon-nitrogen (but not peptide) bonds	Not Available
689	Poly-beta-1, 6-N-acetyl-D-glucosamine synthase (Poly-beta-1, 6-GlcNAc synthase) (EC 2.4.1.-)	*Anaeromyxobacter spp. PSR-1*		A0A0D6QFS3	--	23709511	Acetyl glucosaminyl transferase activity	Not Available
690	Biofilm PGA synthesis lipoprotein PgaB	*Actinobacillus pleuropneumoniae serotype 3*	Strain JL03	B0BTK3	--	18197260	Hydrolase activity, acting on carbon-nitrogen (but not peptide) bonds	Not Available
691	Poly-beta-1, 6-N-acetyl-D-glucosamine synthase (Poly-beta-1, 6-GlcNAc synthase) (EC 2.4.1.-)	*Pseudomonas kairouanensis*		A0A4Z0ANC1	--	30910423	Acetyl glucosaminyl transferase activity	Not Available

Target ID	Bacterial Protein Name	Organism Name	Strain	UniProt ID	PDB ID	PubMed ID	Classification as per UniProt GO Molecular Function	Classification of Function as per PDB
692	Poly-beta-1, 6-N-acetyl-D-glucosamine N-deacetylase PgaB	*Pseudomonas kairouanensis*		A0A4Z0API9	--	30910423	Hydrolase activity, acting on carbon-nitrogen (but not peptide) bonds	Not Available
693	Uncharacterized protein	*Macrococcus goetzii*		A0A395G7Z9	--	29951040	Not Available	Not Available
694	Type II toxin-antitoxin system MqsR family toxin	*Pseudomonas kairouanensis*		A0A4Z0AZZ6	--	30910423	Not Available	Not Available
695	Type II toxin-antitoxin system MqsR family toxin	*Thermoanaerobacter spp.*		A0A7C7E8X0	--	32123542	Not Available	Not Available
696	Uncharacterized protein	*Rhodopila globiformis*	RhodoPseudomonas globiformis	A0A2S6NP98	--	29423563	Not Available	Not Available
697	Poly-beta-1, 6-N-acetyl-D-glucosamine biosynthesis protein PgaD	*Pseudomonas kairouanensis*		A0A4Z0ANQ1	--	30910423	Not Available	Not Available
698	Toxin-antitoxin system, toxin component	*Burkholderiales bacterium RIFOXYC12_FULL_60_6*		A0A1F4P0B8	--	27774985	Not Available	Not Available
699	GGDEF domain-containing protein	*Pseudomonas aeruginosa*	Strain ATCC 15692 / DSM 22644 / CIP 104116 / JCM 14847 / LMG 12228 / 1C / PRS 101 / PAO1	Q9I2P4	--	10984043	Diguanylate cyclase activity	Not Available
700	Surface composition regulator	*Salmonella paratyphi C*	Strain RKS4594	C0PYX0	--	19229335	Not Available	Not Available
701	Poly-beta-1, 6-N-acetyl-D-glucosamine synthase (Poly-beta-1, 6-GlcNAc synthase) (EC 2.4.1.-)	*Macrococcus goetzii*		A0A395G880	--	29951040	Acetyl glucosaminyl transferase activity	Not Available
702	Poly-beta-1, 6-N-acetyl-D-glucosamine synthase (Poly-beta-1, 6-GlcNAc synthase) (EC 2.4.1.-)	*Exiguobacterium chiriqhucha RW-2*		U1LZK0	--	23929485	Acetyl glucosaminyl transferase activity	Not Available
703	Surface composition regulator	*Enterobacter lignolyticus*	Strain SCF1	E3GBM1	--	22180812	Not Available	Not Available
704	Uncharacterized protein	*Acinetobacter baumannii*	Strain AYE	B0V954	--	18350144	Not Available	Not Available
705	NodB homology domain-containing protein	*Acinetobacter baumannii*	Strain AYE	B0V7F4	--	18350144	Hydrolase activity, acting on carbon-nitrogen (but not peptide) bonds	Not Available
706	Poly-beta-1, 6-N-acetyl-D-glucosamine synthase (Poly-beta-1, 6-GlcNAc synthase) (EC 2.4.1.-)	*Halomonas spp. 54_146*		A0A101CZX4	--	26787827	Acetyl glucosaminyl transferase activity	Not Available
707	NodB homology domain-containing protein	*Halomonas spp. 54_146*		A0A101CZW4	--	26787827	Hydrolase activity, acting on carbon-nitrogen (but not peptide) bonds	Not Available
708	Poly-beta-1, 6-N-acetyl-D-glucosamine synthase (Poly-beta-1, 6-GlcNAc synthase) (EC 2.4.1.-)	*Acinetobacter baumannii*	Strain AYE	B0V7F5	--	18350144	Acetyl glucosaminyl transferase activity	Not Available
709	GGDEF domain-containing protein	*Pseudomonas aeruginosa*	Strain ATCC 15692 / DSM 22644 / CIP 104116 / JCM 14847 / LMG 12228 / 1C / PRS 101 / PAO1	Q9HZX6	--	10984043	Diguanylate cyclase activity	Not Available
710	Uncharacterized protein	*Acinetobacter baumannii*	Strain AYE	B0V7F6	--	18350144	Not Available	Not Available
711	Probable two-component response regulator	*Pseudomonas aeruginosa*	Strain ATCC 15692 / DSM 22644 / CIP 104116 / JCM 14847 / LMG 12228 / 1C / PRS 101 / PAO1	Q9HUW7	--	10984043	Diguanylate cyclase activity	Not Available
712	Putative lipoprotein (Haemin storage system) (HmsF)	*Acinetobacter baumannii*	Strain AYE	B0V956	--	18350144	Hydrolase activity, acting on carbon-nitrogen (but not peptide) bonds	Not Available
713	Poly-beta-1, 6-N-acetyl-D-glucosamine N-deacetylase PgaB	*Aeromona salmonicida*		A0A428VR46	--	30502493	Hydrolase activity, acting on carbon-nitrogen (but not peptide) bonds	Not Available
714	Poly-beta-1, 6-N-acetyl-D-glucosamine synthase (Poly-beta-1, 6-GlcNAc synthase) (EC 2.4.1.-)	*Acinetobacter baumannii*	Strain AYE	B0V955	--	18350144	Acetyl glucosaminyl transferase activity	Not Available
715	Uncharacterized protein	*Caldanaerobacter subterraneus subspp. yonseiensis KB-1*		U5CHV1	--	24201201	Not Available	Not Available
716	Poly-beta-1, 6-N-acetyl-D-glucosamine synthase (Poly-beta-1, 6-GlcNAc synthase) (EC 2.4.1.-)	*Acinetobacter ursingii*		A0A3D2SJG2	--	30148503	Acetyl glucosaminyl transferase activity	Not Available
717	Poly-beta-1, 6-N-acetyl-D-glucosamine N-deacetylase PgaB	*Enterobacter hormaechei*		A0A6G3F3N1	--	31477907	Hydrolase activity, acting on carbon-nitrogen (but not peptide) bonds	Not Available
718	Biofilm PGA synthesis auxiliary protein PgaD	*Acinetobacter ursingii*		A0A3G9G0U0	--	28293292	Not Available	Not Available
719	Poly-beta-1, 6-N-acetyl-D-glucosamine N-deacetylase PgaB (Fragment)	*Acinetobacter ursingii*		A0A3D2SPM8	--	30148503	Hydrolase activity, acting on carbon-nitrogen (but not peptide) bonds	Not Available
720	Surface composition regulator	*Enterobacter hormaechei*		A0A2G4ZVG2	--	29088362	Not Available	Not Available
721	Poly-beta-1, 6-N-acetyl-D-glucosamine synthase (Poly-beta-1, 6-GlcNAc synthase) (EC 2.4.1.-)	*Enterobacter hormaechei*		A0A2J0PY96	--	29088362	Acetyl glucosaminyl transferase activity	Not Available
722	Surface composition regulator	*Enterobacter hormaechei*		A0A6G3F999	--	31477907	Not Available	Not Available
723	Poly-beta-1, 6-N-acetyl-D-glucosamine synthase (Poly-beta-1, 6-GlcNAc synthase) (EC 2.4.1.-)	*Achromobacter marplatensis*		J4QR07	--	23105084	Acetyl glucosaminyl transferase activity	Not Available
724	Uncharacterized protein	*Achromobacter marplatensis*		J4J7C4	--	23105084	Not Available	Not Available
725	Poly-beta-1, 6-N-acetyl-D-glucosamine N-deacetylase PgaB	*Methylomona denitrificans*		A0A140E5K7	--	25580993	Hydrolase activity, acting on carbon-nitrogen (but not peptide) bonds	Not Available
726	Poly-beta-1, 6-N-acetyl-D-glucosamine biosynthesis protein PgaD	*Enterobacter hormaechei*		A0A6G3F3X4	--	31477907	Not Available	Not Available
727	Uncharacterized protein	*Achromobacter marplatensis*		J4J6D3	--	23105084	Not Available	Not Available
728	Hemin storage protein	*Achromobacter marplatensis*		J4YMC5	--	23105084	Hydrolase activity, acting on carbon-nitrogen (but not peptide) bonds	Not Available
729	Uncharacterized protein	*Methylomona denitrificans*		A0A140E5K9	--	25580993	Not Available	Not Available
730	Poly-beta-1, 6-N-acetyl-D-glucosamine synthase (Poly-beta-1, 6-GlcNAc synthase) (EC 2.4.1.-)	*Achromobacter marplatensis*		J4Y513	--	23105084	Acetyl glucosaminyl transferase activity	Not Available
731	Biofilm PGA synthesis auxiliary protein PgaD	*Acinetobacter ursingii*		A0A3G9FU89	--	28293292	Not Available	Not Available
732	Poly-beta-1, 6-N-acetyl-D-glucosamine synthase (Poly-beta-1, 6-GlcNAc synthase) (EC 2.4.1.-)	*Methylomona denitrificans*		A0A140E5K8	--	25580993	Acetyl glucosaminyl transferase activity	Not Available
733	Poly-beta-1, 6-N-acetyl-D-glucosamine synthase (Poly-beta-1, 6-GlcNAc synthase) (EC 2.4.1.-)	*Acinetobacter ursingii*		A0A3G9FTN3	--	28293292	Acetyl glucosaminyl transferase activity	Not Available
734	Poly-beta-1, 6-N-acetyl-D-glucosamine N-deacetylase PgaB	*Enterobacter hormaechei*		A0A2J0PYU0	--	29088362	Hydrolase activity, acting on carbon-nitrogen (but not peptide) bonds	Not Available
735	Polysaccharide deacetylase	*Escherichia coli O78:H11*	Strain H10407 / ETEC	E3PJJ0	--	20802035	Hydrolase activity, acting on carbon-nitrogen (but not peptide) bonds	Not Available
736	Surface composition regulator	*Escherichia coli O78:H11*	Strain H10407 / ETEC	E3PJW4	--	20802035	Not Available	Not Available
737	Poly-beta-1, 6-N-acetyl-D-glucosamine synthase (Poly-beta-1, 6-GlcNAc synthase) (EC 2.4.1.-)	*Escherichia coli O78:H11*	Strain H10407 / ETEC	E3PJI9	--	20802035	Acetyl glucosaminyl transferase activity	Not Available
738	Poly-beta-1, 6-N-acetyl-D-glucosamine synthase (Poly-beta-1, 6-GlcNAc synthase) (EC 2.4.1.-)	*Carnobacterium divergens DSM 20623*		A0A0R2HW17	--	26415554	Acetyl glucosaminyl transferase activity	Not Available
739	Uncharacterized protein	*Staphylococcus aureus subspp. aureus 71193*		A0A0E0VNH0	--	22375071	Not Available	Not Available

Target ID	Bacterial Protein Name	Organism Name	Strain	UniProt ID	PDB ID	PubMed ID	Classification as per UniProt GO Molecular Function	Classification of Function as per PDB
740	Poly-beta-1, 6-N-acetyl-D-glucosamine synthase (Poly-beta-1, 6-GlcNAc synthase) (EC 2.4.1.-)	*Candidatus Muproteobacteria bacterium RBG_19FT_COMBO_61_10*		A0A1F6UP22	--	27774985	Acetyl glucosaminyl transferase activity	Not Available
741	Poly-beta-1, 6-N-acetyl-D-glucosamine synthase (Poly-beta-1, 6-GlcNAc synthase) (EC 2.4.1.-)	*Acidobacteria bacterium*		A0A2V7YF32	--	29899444	Acetyl glucosaminyl transferase activity	Not Available
742	Poly-beta-1, 6-N-acetyl-D-glucosamine synthase (Poly-beta-1, 6-GlcNAc synthase) (EC 2.4.1.-)	*Aggregatibacter aphrophilus*	Haemophilus aphrophilus	A0A3M6PFA6	--	30598730	Acetyl glucosaminyl transferase activity	Not Available
743	Poly-beta-1, 6-N-acetyl-D-glucosamine N-deacetylase PgaB	*Gammaproteobacteria bacterium HGW-Gammaproteobacteria-10*		A0A2N1ZS52	--	28350393	Hydrolase activity, acting on carbon-nitrogen (but not peptide) bonds	Not Available
744	Poly-beta-1, 6-N-acetyl-D-glucosamine synthase (Poly-beta-1, 6-GlcNAc synthase) (EC 2.4.1.-)	*Alicyclobacillus acidocaldarius*	Strain Tc-4-1 Bacills acidocaldarius	F8IJL3	--	21914900	Acetyl glucosaminyl transferase activity	Not Available
745	Poly-beta-1, 6-N-acetyl-D-glucosamine synthase (Poly-beta-1, 6-GlcNAc synthase) (EC 2.4.1.-)	*Lautropia spp. SCN 70-15*		A0A1D2TKP6	--	26031303	Acetyl glucosaminyl transferase activity	Not Available
746	mRNA interferase MqsR	*Janthinobacterium spp.*		A0A349L6R0	--	30148503	Not Available	Not Available
747	Poly-beta-1, 6-N-acetyl-D-glucosamine synthase (Poly-beta-1, 6-GlcNAc synthase) (EC 2.4.1.-)	*Gammaproteobacteria bacterium HGW-Gammaproteobacteria-10*		A0A2N1ZS36	--	28350393	Acetyl glucosaminyl transferase activity	Not Available
748	Uncharacterized protein	*Alicyclobacillus acidocaldarius*	Strain Tc-4-1 Bacills acidocaldarius	F8IJL4	--	21914900	Not Available	Not Available
749	Poly-beta-1, 6-N-acetyl-D-glucosamine biosynthesis protein PgaD	*Gammaproteobacteria bacterium HGW-Gammaproteobacteria-10*		A0A2N1ZS44	--	28350393	Not Available	Not Available
750	Biofilm PGA synthesis protein PgaD	*Escherichia coli O139:H28*	Strain E24377A / ETEC	A7ZKC6	--	18676672	Not Available	Not Available
751	Uncharacterized protein	*Pseudomonas putida H8234*		R9VGH1	--	23868128	Not Available	Not Available
752	Motility quorum-sensing regulator MqsR	*Deltaproteobacteria bacterium RIFOXYD12_FULL_56_24*		A0A1F9L5Q3	--	27774985	Not Available	Not Available
753	Poly-beta-1, 6-N-acetyl-D-glucosamine N-deacetylase PgaB	*Lautropia spp. SCN 70-15*		A0A1D2TKR0	--	26031303	Hydrolase activity, acting on carbon-nitrogen (but not peptide) bonds	Not Available
754	Poly-beta-1, 6-N-acetyl-D-glucosamine biosynthesis protein PgaD	*Lautropia spp. SCN 70-15*		A0A1D2TKI8	--	26031303	Not Available	Not Available
755	Poly-beta-1, 6-N-acetyl-D-glucosamine N-deacetylase PgaB	*Aggregatibacter aphrophilus*	Haemophilus aphrophilus	A0A3M6PFB5	--	30598730	Hydrolase activity, acting on carbon-nitrogen (but not peptide) bonds	Not Available
756	Uncharacterized protein	*Deltaproteobacteria bacterium RIFOXYD12_FULL_56_24*		A0A1F9L1K1	--	27774985	Not Available	Not Available
757	Toxin-antitoxin system, toxin component	*Hafnia alvei FB1*		A0A097R795	--	25075225	Not Available	Not Available
758	Poly-beta-1, 6-N-acetyl-D-glucosamine synthase (Poly-beta-1, 6-GlcNAc synthase) (EC 2.4.1.-)	*Staphylococcus aureus subspp. aureus CN1*		T1YDR1	--	23977354	Acetyl glucosaminyl transferase activity	Not Available
759	Polysaccharide deacetylase	*Xenorhabdus ehlersii*		A0A2D0ILC7	--	28993611	Hydrolase activity, acting on carbon-nitrogen (but not peptide) bonds	Not Available
760	Poly-beta-1, 6-N-acetyl-D-glucosamine synthase (Poly-beta-1, 6-GlcNAc synthase) (EC 2.4.1.-)	*Escherichia coli O139:H28*	Strain E24377A / ETEC	A7ZKC7	--	18676672	Acetyl glucosaminyl transferase activity	Not Available
761	Biofilm PGA synthesis lipoprotein pgaB (EC 3.-.-.-)	*Escherichia coli O139:H28*	Strain E24377A / ETEC	A7ZKC8	--	18676672	Hydrolase activity, acting on carbon-nitrogen (but not peptide) bonds	Not Available
762	Hemin storage protein	*Hafnia alvei FB1*		A0A097R0G2	--	25075225	Hydrolase activity, acting on carbon-nitrogen (but not peptide) bonds	Not Available
763	Poly-beta-1, 6-N-acetyl-D-glucosamine synthase (Poly-beta-1, 6-GlcNAc synthase) (EC 2.4.1.-)	*Xenorhabdus ehlersii*		A0A2D0ILF0	--	28993611	Acetyl glucosaminyl transferase activity	Not Available
764	Putative intercellular adhesion protein A (Biofilm formation)	*Vibrio parahaemolyticus serotype O3:K6*	Strain RIMD 2210633	Q87PP2	--	12620739	Not Available	Not Available
765	Surface composition regulator	*Hafnia alvei FB1*		A0A097R276	--	25075225	Not Available	Not Available
766	Uncharacterized protein	*Legionella drancourtii LLAP12*		G9ERV4	--	22047552	Not Available	Not Available
767	Poly-beta-1, 6-N-acetyl-D-glucosamine biosynthesis protein PgaD	*Xenorhabdus ehlersii*		A0A2D0ILC2	--	28993611	Not Available	Not Available
768	Poly-beta-1, 6-N-acetyl-D-glucosamine synthase (Poly-beta-1, 6-GlcNAc synthase) (EC 2.4.1.-)	*Hafnia alvei FB1*		A0A097R0I0	--	25075225	Acetyl glucosaminyl transferase activity	Not Available
769	Surface composition regulator	*Edwardsiella tarda*	Strain FL6-60	A0A0H3DQQ5	--	22003892	Not Available	Not Available
770	Surface composition regulator	*Salmonella paratyphi A*	Strain ATCC 9150 / SARB42	A0A735JW74	--	30286803	Not Available	Not Available
771	Poly-beta-1, 6-N-acetyl-D-glucosamine synthase (Poly-beta-1, 6-GlcNAc synthase) (EC 2.4.1.-)	*Yersinia enterocolitica*	type O:2 Str. YE3094 / 96	A0A7U7IYU5	--	24753568	Acetyl glucosaminyl transferase activity	Not Available
772	Surface composition regulator	*Yersinia enterocolitica*	type O:2 Str. YE3094 / 96	A0A7U7FZ92	--	24753568	DNA binding, DNA-directed DNA polymerase activity	Not Available
773	Uncharacterized protein	*Xanthomonas hortorum pv. carotae*	Str. M081	V7ZEF0	--	21722296	Not Available	Not Available
774	Surface composition regulator	*Salmonella enterica subspp. enterica serovar*	6, 7:c:1, 5	A0A740VH22	--	30286803	Not Available	Not Available
775	Surface composition regulator	*Yersinia enterocolitica*	type O:5, 27 Str. YE149 / 02	A0A7U7IQG9	--	24753568	DNA binding, DNA-directed DNA polymerase activity	Not Available
776	Haemin storage system, HmsS protein	*Yersinia enterocolitica*	type O:5, 27 Str. YE149 / 02	A0A7U7FMU7	--	24753568	Not Available	Not Available
777	IcaA (Fragment)	*Staphylococcus condimenti*		Q83ZI0	--	12957956	Not Available	Not Available
778	Uncharacterized protein	*Yersinia enterocolitica*	type O:2 Str. YE3094 / 96	A0A7U7G2K0	--	24753568	Not Available	Not Available
779	Surface composition regulator	*Salmonella enterica subspp. houtenae serovar*	44:z4, z24:-	A0A737NPA5	--	30286803	Not Available	Not Available
780	Surface composition regulator	*Salmonella enterica subspp. enterica serovar*	Miami	A0A5I2DLX0	--	30286803	Not Available	Not Available
781	Haemin storage system, HmsS protein	*Yersinia enterocolitica*	type O:2 Str. YE3094 / 96	A0A7U7IYG1	--	24753568	Not Available	Not Available
782	Surface composition regulator	*Salmonella enterica subspp. houtenae serovar 48:z4, z32:-*		A0A729FR63	--	30286803	Not Available	Not Available
783	Surface composition regulator	*Salmonella enterica subspp. enterica serovar Typhi*	Str. CT18	A0A718C8V5	--	30286803	Not Available	Not Available
784	Surface composition regulator	*Salmonella enterica subspp. arizonae serovar*	41:z4, z23:-	A0A741N0E9	--	30286803	Not Available	Not Available
785	S-ribosylhomocysteine lyase (EC 4.4.1.21) (AI-2 synthesis protein) (Autoinducer-2 production protein LuxS)	*Aeromona hydrophila*		B1PWE7	--	18930130	Iron ion binding, S-ribosylhomocysteine lyase activity	Not Available
786	PsoR	*Pseudomonas putida*	Arthrobacter siderocapsulatus	A8MN34	--	16585751; 18599835	DNA binding	Not Available
787	IcaA (Fragment)	*Staphylococcus condimenti*		Q83ZH5	--	12957956	Not Available	Not Available
788	Biofilm PGA synthesis lipoprotein pgaB	*Yersinia enterocolitica W22703*		F4N0N6	--	21453472	Hydrolase activity, acting on carbon-nitrogen (but not peptide) bonds	Not Available
789	Uncharacterized protein (Fragment)	*Klebsiella pneumoniae*		Q1G0U9	--	16861646	Not Available	Not Available

Target ID	Bacterial Protein Name	Organism Name	Strain	UniProt ID	PDB ID	PubMed ID	Classification as per UniProt GO Molecular Function	Classification of Function as per PDB
790	Uncharacterized protein	*Klebsiella pneumoniae*		Q1G0V5	--	16861646	Not Available	Not Available
791	Surface composition regulator	*Salmonella enterica subspp. enterica serovar Kouka*		A0A729KY13	--	30286803	Not Available	Not Available
792	LysR_substrate domain-containing protein (Fragment)	*Klebsiella pneumoniae*		Q1G0W0	--	16861646	Not Available	Not Available
793	Uncharacterized protein	*Klebsiella pneumoniae*		Q1G0V8	--	16861646	Not Available	Not Available
794	Surface composition regulator	*Salmonella enterica subspp. houtenae serovar*	1, 40:z4, z32:-	A0A730WRJ9	--	30286803	Not Available	Not Available
795	Uncharacterized protein	*Klebsiella pneumoniae*		Q1G0U0	--	16861646	Not Available	Not Available
796	Aldedh domain-containing protein	*Klebsiella pneumoniae*		Q1G0V4	--	16861646	Oxidoreductase activity, acting on the aldehyde or oxo group of donors, NAD or NADP as acceptor	Not Available
797	Uncharacterized protein (Fragment)	*Klebsiella pneumoniae*		Q1G0T9	--	16861646	Not Available	Not Available
798	Uncharacterized protein (Fragment)	*Klebsiella pneumoniae*		Q1G0U6	--	16861646	Not Available	Not Available
799	Uncharacterized protein	*uncultured Desulfobacterium spp.*		E1YIC6	--	21176053	Not Available	Not Available
800	Poly-beta-1, 6-N-acetyl-D-glucosamine synthase (Poly-beta-1, 6-GlcNAc synthase) (EC 2.4.1.-)	*Acinetobacter baumannii*		C8YYH7	--	19633088	Acetyl glucosaminyl transferase activity	Not Available
801	Autoinducer-2 ABC transporter permease protein	*Klebsiella pneumoniae*		C8BNA6	--	20600864	Transmembrane transporter activity	Not Available
802	Uncharacterized protein	*Klebsiella pneumoniae*		Q1G0U5	--	16861646	Not Available	Not Available
803	Urease accessory protein UreF	*Staphylococcus spp. T-02*		B9A8F9	--	20208338	Nickel cation binding	Not Available
804	Surface composition regulator	*Yersinia enterocolitica W22703*		F4N5Y4	--	21453472	DNA binding, DNA-directed DNA polymerase activity	Not Available
805	Poly-beta-1, 6-N-acetyl-D-glucosamine synthase (Poly-beta-1, 6-GlcNAc synthase) (EC 2.4.1.-) (Fragment)	*Yersinia enterocolitica W22703*		F4MUX8	--	21453472	Acetyl glucosaminyl transferase activity	Not Available
806	Poly-beta-1, 6-N-acetyl-D-glucosamine synthase (Poly-beta-1, 6-GlcNAc synthase) (EC 2.4.1.-)	*Staphylococcus capitis*		Q6YI89	--	12957956	Acetyl glucosaminyl transferase activity	Not Available
807	Surface composition regulator	*Salmonella typhimurium*		A0A701JYH4	--	30286803	Not Available	Not Available
808	Surface composition regulator	*Salmonella enterica subspp. enterica serovar*	Orion	A0A3T2WB67	--	30286803	Not Available	Not Available
809	Surface composition regulator	*Salmonella gallinarum*		A0A3V9NPH3	--	30286803	Not Available	Not Available
810	IcaR (Fragment)	*Staphylococcus capitis*		Q6YI86	--	12957956	DNA binding	Not Available
811	Uncharacterized protein	*Klebsiella pneumoniae*		Q1G0U2	--	16861646	Not Available	Not Available
812	IcaA (Fragment)	*Staphylococcus capitis*		Q83ZH3	--	12957956	Not Available	Not Available
813	PTS EIIC type-3 domain-containing protein (Fragment)	*Klebsiella pneumoniae*		Q1G0U4	--	16861646	Protein-N(PI)-phosphohistidine-sugar phosphoTransferase activity	Not Available
814	Biofilm polysaccharide intercellular adhesin deacetylase (Intercellular adhesion protein B) (Poly-beta-1, 6-N-acetyl-D-glucosamine N-deacetylase) (Fragment)	*Staphylococcus capitis*		Q6YI87	--	12957956	Hydrolase activity, acting on carbon-nitrogen (but not peptide) bonds, in linear amides	Not Available
815	Surface composition regulator	*Salmonella enterica subspp. enterica serovar*	Ohio	A0A3V5VUX0	--	30286803	Not Available	Not Available
816	Surface composition regulator	*Salmonella enterica subspp. houtenae serovar*	43:z4, z23:-	A0A730K0K8	--	30286803	Not Available	Not Available
817	Uncharacterized protein	*Yersinia enterocolitica W22703*		F4MUX9	--	21453472	Not Available	Not Available
818	Surface composition regulator	*Salmonella enterica subspp. enterica serovar Paratyphi B*		A0A3V7VTQ9	--	30286803	Not Available	Not Available
819	Surface composition regulator	*Salmonella enterica subspp. enterica serovar*	Rubislaw Str. ATCC 10717	A0A6W0NR32	--	30286803	Not Available	Not Available
820	Biofilm polysaccharide intercellular adhesin synthesis protein IcaD (Intercellular adhesion protein D) (Poly-beta-1, 6-N-acetyl-D-glucosamine synthesis protein IcaD) (Fragment)	*Staphylococcus capitis*		Q6YI88	--	12957956	Not Available	Not Available
821	S-adenosylmethionine decarboxylase proenzyme (AdoMetDC) (SAMDC) (EC 4.1.1.50) [Cleaved into: S-adenosylmethionine decarboxylase beta chain; S-adenosylmethionine decarboxylase alpha chain]	*Bacillus cereus*		A0A063CLJ3	--	25301660; 30637927	Adenosylmethionine decarboxylase activity	Not Available
822	Ribonuclease J (RNAse J) (EC 3.1.-.-)	*Bacillus cereus*		A0A068NAJ3	--	25301660	5'-3' exoribonuclease activity, endoribonuclease activity, RNA binding, zinc ion binding	Not Available
823	Poly-beta-1, 6-N-acetyl-D-glucosamine synthase (Poly-beta-1, 6-GlcNAc synthase) (EC 2.4.1.-)	*Staphylococcus aureus*		A0A2S6DQT8	--	29479347	Acetyl glucosaminyl transferase activity	Not Available
824	Global transcriptional regulator Spx	*Bacillus cereus*		A0A2A8ED49	--	25301660; 31228823; 30637927; 33353020	Not Available	Not Available
825	Surface composition regulator	*Salmonella hadar*		A0A3V2W5E6	--	30286803; 30902850	Not Available	Not Available
826	Ribonuclease PH (RNAse PH) (EC 2.7.7.56) (tRNA nucleotidyl transferase)	*Bacillus cereus*		A0A080TUN6	--	31228823; 30637927	3'-5'-exoribonuclease activity, tRNA binding, tRNA nucleotidylTransferase activity	Not Available
827	Ribonuclease J (RNAse J) (EC 3.1.-.-)	*Bacillus cereus*		A0A063CPQ3	--	25301660	5'-3' exoribonuclease activity, endoribonuclease activity, RNA binding, zinc ion binding	Not Available
828	Surface composition regulator	*Salmonella enterica subspp. enterica serovar*	Braenderup	A0A3T3G645	--	30286803	Not Available	Not Available
829	Type II toxin-antitoxin system MqsR family toxin	*Yersinia pestis*		A0A3N4BIT9	--	11586360	Not Available	Not Available
830	Surface composition regulator	*Salmonella enterica subspp. enterica serovar*	Javiana	A0A3V7PI12	--	30286803	Not Available	Not Available
831	Surface composition regulator	*Salmonella anatum*		A0A2T8MEV5	--	30286803; 30902850	Not Available	Not Available
832	Surface composition regulator	*Salmonella potsdam*		A0A5I6RXB1	--	30286803	Not Available	Not Available
833	Surface composition regulator	*Salmonella enterica subspp. enterica serovar*	Coeln	A0A5I3IDD6	--	30286803	Not Available	Not Available
834	Surface composition regulator	*Salmonella enterica subspp. enterica serovar*	Napoli	A0A5H6JAD5	--	30286803	Not Available	Not Available
835	Poly-beta-1, 6-N-acetyl-D-glucosamine synthase (Poly-beta-1, 6-GlcNAc synthase) (EC 2.4.1.-)	*Staphylococcus lugdunensis*		A8DMY8	--	17635864	Acetyl glucosaminyl transferase activity	Not Available
836	Surface composition regulator	*Salmonella enterica subspp. enterica serovar*		A0A720D3V5	--	30286803	Not Available	Not Available
837	AHL-dependent Transcriptional regulator	*Edwardsiella tarda*		Q60HM0	--	16233705; 19283379	DNA binding	Not Available
838	Surface composition regulator	*Salmonella enteritidis PT4*	Strain P125109	A0A724LRF7	--	30286803	Not Available	Not Available
839	Surface composition regulator	*Salmonella enterica subspp. enterica serovar*	Havana	A0A3V3TZ71	--	30286803	Not Available	Not Available

Target ID	Bacterial Protein Name	Organism Name	Strain	UniProt ID	PDB ID	PubMed ID	Classification as per UniProt GO Molecular Function	Classification of Function as per PDB
840	Surface composition regulator	*Salmonella enterica subspp. enterica serovar*	Eastbourne	A0A3V5UUL8	--	30286803	Not Available	Not Available
841	Surface composition regulator	*Salmonella enterica subspp. enterica serovar*	Haifa	A0A3V5ZEC4	--	30902850	Not Available	Not Available
842	Surface composition regulator	*Edwardsiella piscicida*		A0A034SUT9	--	24926054	Not Available	Not Available
843	Adenosylhomocysteinae (EC 3.3.1.1) (S-adenosyl-L-homocysteine Hydrolase) (AdoHcyase)	*Pseudomonas aeruginosa*		K7QUT8	--	22942290	Adenosylhomocysteinae activity	Not Available
844	Poly-beta-1, 6-N-acetyl-D-glucosamine N-deacetylase (Poly-beta-1, 6-N-acetyl-D-glucosamine N-deacetylase PgaB) (EC 3.5.1.-)	*Pseudomonas lactis*		A0A219A6N9	--	32587583	Hydrolase activity, acting on carbon-nitrogen (but not peptide) bonds	Not Available
845	Surface composition regulator	*Salmonella enterica subspp. enterica serovar*	Bareilly	A0A4Z8Z843	--	30286803; 30902850	Not Available	Not Available
846	Poly-beta-1, 6-N-acetyl-D-glucosamine N-deacetylase	*Pseudomonas prosekii*		A0A3L8CDD7	--	29867824	Hydrolase activity, acting on carbon-nitrogen (but not peptide) bonds	Not Available
847	Biofilm PGA synthesis lipoprotein PgaB (EC 3.-.-.-) (Poly-beta-1, 6-N-acetyl-D-glucosamine N-deacetylase PgaB) (EC 3.5.1.-)	*Pseudomonas lactis*		I4KF65	--	22792073; 32587583	Hydrolase activity, acting on carbon-nitrogen (but not peptide) bonds	Not Available
848	DNA polymerase III subunit delta (EC 2.7.7.7) (HolA)	*Staphylococcus lugdunensis*		A0A0N7BA97	--	26416910	DNA binding, DNA-directed DNA polymerase activity	Not Available
849	3-hydroxylisobutyryl-CoA Hydrolase (Putative enoyl-CoA hydratase / isomerase family protein)	*Acinetobacter oleivorans*	Strain JCM 16667 / KCTC 23045 / DR1	D3JTG3	--	20629796; 20639327	3-hydroxyisobutyryl-CoA Hydrolase activity, isomerase activity	Not Available
850	Surface composition regulator	*Salmonella enterica subspp. enterica serovar*	Concord	A0A2R4D758	--	30286803	Not Available	Not Available
851	Poly-beta-1, 6-N-acetyl-D-glucosamine biosynthesis protein PgaD	*Pseudomonas prosekii*		A0A3L8CDF8	--	29867824	Not Available	Not Available
852	Poly-beta-1, 6-N-acetyl-D-glucosamine biosynthesis protein PgaD	*Acinetobacter schindleri*		A0A2S1FF05	--	29954893	Not Available	Not Available
853	Poly-beta-1, 6-N-acetyl-D-glucosamine biosynthesis protein PgaD (Putative biofilm PGA synthesis protein PgaD)	*Pseudomonas lactis*		A0A0R3BJY0	--	22792073; 32587583	Not Available	Not Available
854	Poly-beta-1, 6-N-acetyl-D-glucosamine synthase (Poly-beta-1, 6-GlcNAc synthase) (EC 2.4.1.-)	*Acinetobacter cumulans*		A0A3A8G879	--	30808586	Acetyl glucosaminyl transferase activity	Not Available
855	Poly-beta-1, 6-N-acetyl-D-glucosamine synthase (Poly-beta-1, 6-GlcNAc synthase) (EC 2.4.1.-)	*Pseudomonas prosekii*		A0A3L8CCK9	--	29867824	Acetyl glucosaminyl transferase activity	Not Available
856	ComE operon protein 2 (ComEB)	*Staphylococcus lugdunensis*		A0A0N7B1F5	--	26416910	dCMP deaminae activity, zinc ion binding	Not Available
857	Poly-beta-1, 6-N-acetyl-D-glucosamine synthase (Poly-beta-1, 6-GlcNAc synthase) (EC 2.4.1.-)	*Pseudomonas lactis*		I4KC34	--	22792073; 32587583	Acetyl glucosaminyl transferase activity	Not Available
858	Surface composition regulator	*Klebsiella oxytoca*		A0A0G3S4R7	--	29437920	Not Available	Not Available
859	Motility quorum-sensing regulator MqsR (Type II toxin-antitoxin system MqsR family toxin)	*Pseudomonas lactis*		I4KAK6	--	22792073; 32587583	Not Available	Not Available
860	Poly-beta-1, 6-N-acetyl-D-glucosamine synthase (Poly-beta-1, 6-GlcNAc synthase) (EC 2.4.1.-)	*Yersinia pestis*		A0A1U8QLL4	--	12142430; 15368893	Acetyl glucosaminyl transferase activity	Not Available
861	HTH-type Transcriptional regulator LuxR (LuxR regulator)	*Vibrio scophthalmi*		H6URH3	--	23198796	DNA binding	Not Available
862	Acyl-homoserine-lactone synthase (EC 2.3.1.184) (Autoinducer synthesis protein)	*Aeromona hydrophila*		Q27IV3	--	19729404	N-acyl homoserine lactone synthase activity	Not Available
863	Surface composition regulator	*Yersinia pestis*		Q0WDU2	--	11586360	DNA binding, DNA-directed DNA polymerase activity	Not Available
864	Fimbrial protein (Similar to pilin F17d-A) (Type 1 fimbrial protein)	*Yersinia pestis*		Q9ZC32	--	11586360	Not Available	Not Available
865	Poly-beta-1, 6-N-acetyl-D-glucosamine N-deacetylase PgaB	*Acinetobacter wuhouensis*		A0A385C6B4	--	30117799	Hydrolase activity, acting on carbon-nitrogen (but not peptide) bonds	Not Available
866	AhyR (LuxR family Transcriptional regulator) (LuxR-type Transcriptional regulator)	*Aeromona hydrophila*		Q27IV4	--	19729404	DNA binding	Not Available
867	Surface composition regulator	*Salmonella enterica subspp. enterica serovar*	Lattenkamp	A0A5W2LQM8	--	30286803	Not Available	Not Available
868	HmsT (HmsT protein)	*Yersinia pestis*		Q9X5X6	--	9249062; 10438760; 10463178; 11586360	Diguanylate cyclase activity	Not Available
869	Haemin storage system, HmsF protein (HmsF protein) (Poly-beta-1, 6-N-acetyl-D-glucosamine N-deacetylase PgaB) (EC 3.5.1.-)	*Yersinia pestis*		Q9R7V4	--	11586360	Hydrolase activity, acting on carbon-nitrogen (but not peptide) bonds	Not Available
870	Surface composition regulator	*Salmonella enterica subspp. enterica serovar*	Mbandaka	A0A418ZAK8	--	30286803; 31099590	Not Available	Not Available
871	Frimbrial protein (Putative frimbrial protein) (Type 1 fimbrial protein)	*Yersinia pestis*		Q306M1	--	11586360	Not Available	Not Available
872	Haemin storage system, HmsS protein (HmsS) (HmsS protein) (Poly-beta-1, 6-N-acetyl-D-glucosamine biosynthesis protein PgaD)	*Yersinia pestis*		P74989	--	9249062; 11586360	Not Available	Not Available
873	Poly-beta-1, 6-N-acetyl-D-glucosamine biosynthesis protein PgaD	*Acinetobacter wuhouensis*		A0A385C8R0	--	30117799	Not Available	Not Available
874	Poly-beta-1, 6-N-acetyl-D-glucosamine biosynthesis protein PgaD	*Bacillus megaterium*	Strain ATCC 14581 / DSM 32 / JCM 2506 / NBRC 15308 / NCIMB 9376 / NCTC 10342 / NRRL B-14308 / VKM B-512	A0A0B6AKG3	--	25931591	Not Available	Not Available
875	Amyloid fiber anchoring / assembly protein TapA (Biofilm formation protein) (Lipoprotein for biofilm formation)	*Bacillus glycinifermentans*		A0A0T6BTZ5	--	26297378	Not Available	Not Available
876	Surface composition regulator	*Citrobacter pasteurii*		A0A6N6K9P3	--	30087402	Not Available	Not Available
877	Surface composition regulator	*Salmonella enterica I*		A0A3Z0GZU8	--	30286803	Not Available	Not Available
878	Poly-beta-1, 6-N-acetyl-D-glucosamine synthase (Poly-beta-1, 6-GlcNAc synthase) (EC 2.4.1.-)	*Acinetobacter baumannii*		A0A0D8GQW0	--	26170289	Acetyl glucosaminyl transferase activity	Not Available
879	Anti-FlhC(2)FlhD(4) factor YdiV	*Salmonella typhimurium*	Strain LT2 / SGSC1412 / ATCC 700720	Q8ZLB9	--	11260463; 11677609; 11929533	Not Available	Not Available
880	Biofilm synthesis auxiliary protein PgaD (Poly-beta-1, 6-N-acetyl-D-glucosamine auxiliary protein PgaD) (Poly-beta-1, 6-N-acetyl-D-glucosamine biosynthesis protein PgaD)	*Acinetobacter baumannii*		A0A059ZRK6	--	25136007; 25845845; 26170289	Not Available	Not Available
881	Biofilm PGA synthesis lipoprotein pgaB (Poly-beta-1, 6-N-acetyl-D-glucosamine N-deacetylase PgaB)	*Acinetobacter baumannii*		A0A6L8MEI5	--	27917170; 29137647	Hydrolase activity, acting on carbon-nitrogen (but not peptide) bonds	Not Available
882	Type II toxin-antitoxin system MqsR family toxin	*Pseudomonas spp. WS 5010*		A0A7Y0ZGX5	--	32587583	Not Available	Not Available
883	mRNA interferase MqsR	*Gallionellales bacterium RIFCSPHIGHO2_02_FULL_57_16*		A0A1G0EFX2	--	27774985	Not Available	Not Available
884	Poly-beta-1, 6-N-acetyl-D-glucosamine synthase (Poly-beta-1, 6-GlcNAc synthase) (EC 2.4.1.-)	*Pseudomonas spp. WS 5010*		A0A7Y0ZL97	--	32587583	Acetyl glucosaminyl transferase activity	Not Available

Target ID	Bacterial Protein Name	Organism Name	Strain	UniProt ID	PDB ID	PubMed ID	Classification as per UniProt GO Molecular Function	Classification of Function as per PDB
885	Poly-beta-1, 6-N-acetyl-D-glucosamine biosynthesis protein PgaD	*Pseudomonas spp. WS 5010*		A0A7Y0ZL43	--	32587583	Not Available	Not Available
886	Uncharacterized protein	*Burkholderiales bacterium GWF1_66_17*		A0A1F4H8D6	--	27774985	Not Available	Not Available
887	Poly-beta-1, 6-N-acetyl-D-glucosamine biosynthesis protein PgaD	*Rhodocyclaceae bacterium*		A0A7C6VPP3	--	32123542	Not Available	Not Available
888	Outer membrane N-deacetylase	*Achromobacter arsenitoxydans SY8*		H0F5P5	--	22328747	Hydrolase activity, acting on carbon-nitrogen (but not peptide) bonds	Not Available
889	Poly-beta-1, 6-N-acetyl-D-glucosamine biosynthesis protein PgaD	*Pseudomonas spp. GM25*		J2P592	--	23045501	Not Available	Not Available
890	Putative polysaccharide deacetylase	*Klebsiella pneumoniae subspp. pneumoniae*	Strain HS11286	A0A0H3GL72	--	22408243	Hydrolase activity, acting on carbon-nitrogen (but not peptide) bonds	Not Available
891	Uncharacterized protein	*Rhodocyclales bacterium RIFCSPLOWO2_02_FULL_63_24*		A0A1G3HT42	--	27774985	Not Available	Not Available
892	Uncharacterized protein	*Pseudomonas spp. GM25*		J2XBE6	--	23045501	Not Available	Not Available
893	Biofilm PGA synthesis protein PgaD	*Pseudomonas spp.*	Strain M1	W5INN3	--	10388678; 23405299	Not Available	Not Available
894	Uncharacterized protein	*Rhodocyclales bacterium RIFCSPLOWO2_02_FULL_63_24*		A0A1G3HJF3	--	27774985	Not Available	Not Available
895	Poly-beta-1, 6-N-acetyl-D-glucosamine N-deacetylase PgaB (Fragment)	*Candidatus Desulfofervidus auxilii*		A0A7C0Y3M0	--	31911466	Hydrolase activity, acting on carbon-nitrogen (but not peptide) bonds	Not Available
896	Putative hemin storage protein	*Bordetella bronchiseptica 253*		A0A0C6P1R7	--	23051057	Hydrolase activity, acting on carbon-nitrogen (but not peptide) bonds	Not Available
897	Poly-beta-1, 6-N-acetyl-D-glucosamine synthase (Poly-beta-1, 6-GlcNAc synthase) (EC 2.4.1.-)	*Candidatus Desulfofervidus auxilii*		A0A7C0Y6F6	--	31911466	Acetyl glucosaminyl transferase activity	Not Available
898	Surface composition regulator	*Kosakonia radicincitans DSM 16656*		A0A1V0LHN3	--	22965092	Not Available	Not Available
899	Poly-beta-1, 6-N-acetyl-D-glucosamine synthase (Poly-beta-1, 6-GlcNAc synthase) (EC 2.4.1.-)	*Pseudomonas spp. GM25*		J3F4T7	--	23045501	Acetyl glucosaminyl transferase activity	Not Available
900	Uncharacterized protein	*Bordetella bronchiseptica 253*		A0A0C6P5T2	--	23051057	Not Available	Not Available
901	Poly-beta-1, 6-N-acetyl-D-glucosamine synthase (Poly-beta-1, 6-GlcNAc synthase) (EC 2.4.1.-)	*Bordetella bronchiseptica 253*		A0A0C6P4Z6	--	23051057	Acetyl glucosaminyl transferase activity	Not Available
902	Motility quorum-sensing regulator MqsR	*Pseudomonas fluorescens*		A0A166MX06	--	29769716	Not Available	Not Available
903	Poly-beta-1, 6-N-acetyl-D-glucosamine synthase (Poly-beta-1, 6-GlcNAc synthase) (EC 2.4.1.-)	*Stenotrophomonas spp. DDT-1*		A0A149QNS6	--	26888254	Acetyl glucosaminyl transferase activity	Not Available
904	mRNA interferase MqsR	*Betaproteobacteria bacterium HGW-Betaproteobacteria-3*		A0A2N2RGM4	--	28350393	Not Available	Not Available
905	Type II toxin-antitoxin system MqsR family toxin	*Pseudomonas fluorescens*		A0A7Z8NMX6	--	31040301	Not Available	Not Available
906	Poly-beta-1, 6-N-acetyl-D-glucosamine synthase (Poly-beta-1, 6-GlcNAc synthase) (EC 2.4.1.-)	*Pseudomonas fluorescens*		A0A160A1X0	--	29769716	Acetyl glucosaminyl transferase activity	Not Available
907	Hemin storage protein	*Stenotrophomonas spp. DDT-1*		A0A149QNot Available0	--	26888254	Not Available	Not Available
908	Poly-beta-1, 6-N-acetyl-D-glucosamine synthase (Poly-beta-1, 6-GlcNAc synthase) (EC 2.4.1.-)	*Pseudomonas fluorescens*		A0A161Z6J8	--	29769716	Acetyl glucosaminyl transferase activity	Not Available
909	Poly-beta-1, 6-N-acetyl-D-glucosamine biosynthesis protein PgaD	*Pseudomonas fluorescens*		A0A166NCZ6	--	29769716	Not Available	Not Available
910	Type II toxin-antitoxin system MqsR family toxin	*Pseudomonas veronii*		A0A7Y0ZQP0	--	32587583	Not Available	Not Available
911	Motility quorum-sensing regulator MqsR	*Pseudomonas spp. S49*		A0A7Z2M4T1	--	25398872	Not Available	Not Available
912	Poly-beta-1, 6-N-acetyl-D-glucosamine N-deacetylase PgaB (EC 3.5.1.-)	*Pseudomonas veronii*		A0A7Y0ZZU3	--	32587583	Hydrolase activity, acting on carbon-nitrogen (but not peptide) bonds	Not Available
913	Poly-beta-1, 6-N-acetyl-D-glucosamine biosynthesis protein PgaD	*Pseudomonas fluorescens*		A0A0N9WHA4	--	29769716	Not Available	Not Available
914	Uncharacterized protein	*Mycetohabitans rhizoxinica*	Strain DSM 19002 / CIP 109453 / HKI 454 Paraburkholderia rhizoxinica	E5AT95	--	21131495	Not Available	Not Available
915	Poly-beta-1, 6-N-acetyl-D-glucosamine N-deacetylase	*Pseudomonas fluorescens*		A0A160A2W4	--	29769716	Hydrolase activity, acting on carbon-nitrogen (but not peptide) bonds	Not Available
916	Poly-beta-1, 6-N-acetyl-D-glucosamine biosynthesis protein PgaD	*Pseudomonas fluorescens*		A0A160A0I6	--	29769716	Not Available	Not Available
917	Poly-beta-1, 6-N-acetyl-D-glucosamine N-deacetylase PgaB	*Pseudomonas fluorescens*		A0A0N9W7I0	--	29769716	Hydrolase activity, acting on carbon-nitrogen (but not peptide) bonds	Not Available
918	Poly-beta-1, 6-N-acetyl-D-glucosamine N-deacetylase PgaB (EC 3.5.1.-)	*Pseudomonas veronii*		A0A7Y1ABU3	--	32587583	Hydrolase activity, acting on carbon-nitrogen (but not peptide) bonds	Not Available
919	Putative biofilm formation, controlled by quorum sensing, formation of cell aggregates	*Pseudoalteromonas translucida*	Strain TAC 125	Q3IK57	--	16169927	Not Available	Not Available
920	Poly-beta-1, 6-N-acetyl-D-glucosamine biosynthesis protein PgaD	*Pseudomonas fluorescens*		A0A0N9WGY1	--	29769716	Not Available	Not Available
921	Poly-beta-1, 6-N-acetyl-D-glucosamine synthase (Poly-beta-1, 6-GlcNAc synthase) (EC 2.4.1.-)	*Pseudomonas fluorescens*		A0A0N9WHZ0	--	29769716	Acetyl glucosaminyl transferase activity	Not Available
922	Hemin storage protein	*Stenotrophomonas spp. DDT-1*		A0A149QN88	--	26888254	Hydrolase activity, acting on carbon-nitrogen (but not peptide) bonds	Not Available
923	Poly-beta-1, 6-N-acetyl-D-glucosamine N-deacetylase PgaB	*Pseudomonas fluorescens*		A0A0N9VTC1	--	29769716	Hydrolase activity, acting on carbon-nitrogen (but not peptide) bonds	Not Available
924	Type II toxin-antitoxin system MqsR family toxin	*Pseudomonas veronii*		A0A7Y1ABA9	--	32587583	Not Available	Not Available
925	Putative membrane protein	*Xanthomonas citri pv. mangiferaeindicae LMG 941*		H8FLX0	--	22582385	Not Available	Not Available
926	Polysaccharide deacetylase	*Xenorhabdus spp. KK7.4*		A0A2D0K518	--	28993611	Hydrolase activity, acting on carbon-nitrogen (but not peptide) bonds	Not Available
927	Poly-beta-1, 6-N-acetyl-D-glucosamine synthase (Poly-beta-1, 6-GlcNAc synthase) (EC 2.4.1.-)	*Staphylococcus delphini*		A0A2A4GXC5	--	28859126	Acetyl glucosaminyl transferase activity	Not Available
928	Poly-beta-1, 6-N-acetyl-D-glucosamine biosynthesis protein PgaD	*Pseudomonas veronii*		A0A7Y1ABU2	--	32587583	Not Available	Not Available
929	Poly-beta-1, 6-N-acetyl-D-glucosamine synthase (Poly-beta-1, 6-GlcNAc synthase) (EC 2.4.1.-)	*Pseudomonas veronii*		A0A7Y1ABV4	--	32587583	Acetyl glucosaminyl transferase activity	Not Available
930	Poly-beta-1, 6-N-acetyl-D-glucosamine N-deacetylase (EC 3.5.1.-)	*Xanthomonas citri pv. mangiferaeindicae LMG 941*		H8FLX2	--	22582385	Hydrolase activity, acting on carbon-nitrogen (but not peptide) bonds	Not Available
931	Poly-beta-1, 6-N-acetyl-D-glucosamine biosynthesis protein PgaD	*Pseudomonas spp. S49*		A0A7Z2M4F2	--	25398872	Not Available	Not Available
932	Poly-beta-1, 6-N-acetyl-D-glucosamine synthase (Poly-beta-1, 6-GlcNAc synthase) (EC 2.4.1.-)	*Pseudomonas spp. S49*		A0A7Z2M356	--	25398872	Acetyl glucosaminyl transferase activity	Not Available

Target ID	Bacterial Protein Name	Organism Name	Strain	UniProt ID	PDB ID	PubMed ID	Classification as per UniProt GO Molecular Function	Classification of Function as per PDB
933	Poly-beta-1, 6-N-acetyl-D-glucosamine synthase (Poly-beta-1, 6-GlcNAc synthase) (EC 2.4.1.-)	*Staphylococcus delphini*		A0A2A4HFR7	--	28859126	Acetyl glucosaminyl transferase activity	Not Available
934	Poly-beta-1, 6-N-acetyl-D-glucosamine biosynthesis protein PgaD	*Xenorhabdus spp. KK7.4*		A0A2D0K575	--	28993611	Not Available	Not Available
935	Poly-beta-1, 6-N-acetyl-D-glucosamine biosynthesis protein PgaD	*Pseudomonas veronii*		A0A0R2ZU73	--	32587583	Not Available	Not Available
936	Poly-beta-1, 6-N-acetyl-D-glucosamine N-deacetylase	*Pseudomonas spp. S49*		A0A7Z2M3J1	--	25398872	Hydrolase activity, acting on carbon-nitrogen (but not peptide) bonds	Not Available
937	Poly-beta-1, 6-N-acetyl-D-glucosamine synthase (Poly-beta-1, 6-GlcNAc synthase) (EC 2.4.1.-)	*Xanthomonas citri pv. mangiferaeindicae LMG 941*		H8FLX1	--	22582385	Acetyl glucosaminyl transferase activity	Not Available
938	Type II toxin-antitoxin system MqsR family toxin	*Solimona terrae*		A0A6M2BWG 2	--	24421276	Not Available	Not Available
939	Uncharacterized protein	*Achromobacter arsenitoxydans SY8*		H0F5P7	--	22328747	Not Available	Not Available
940	Poly-beta-1, 6-N-acetyl-D-glucosamine synthase (Poly-beta-1, 6-GlcNAc synthase) (EC 2.4.1.-)	*Achromobacter arsenitoxydans SY8*		H0F5P6	--	22328747	Acetyl glucosaminyl transferase activity	Not Available
941	Poly-beta-1, 6-N-acetyl-D-glucosamine N-deacetylase PgaB	*Xanthomonadales bacterium RIFOXYA1_FULL_69_10*		A0A1G4A9M8	--	27774985	Hydrolase activity, acting on carbon-nitrogen (but not peptide) bonds	Not Available
942	Poly-beta-1, 6-N-acetyl-D-glucosamine biosynthesis protein PgaD	*Enterobacteriaceae bacterium ENNIH2*		A0A2I8QSJ0	--	29437920	Not Available	Not Available
943	Poly-beta-1, 6-N-acetyl-D-glucosamine synthase (Poly-beta-1, 6-GlcNAc synthase) (EC 2.4.1.-)	*Xanthomonadales bacterium RIFOXYA1_FULL_69_10*		A0A1G4A9H6	--	27774985	Acetyl glucosaminyl transferase activity	Not Available
944	Poly-beta-1, 6-N-acetyl-D-glucosamine biosynthesis protein PgaD	*Enterobacter spp. EC-NT1*		A0A3D8XP45	--	29665424	Not Available	Not Available
945	Poly-beta-1, 6-N-acetyl-D-glucosamine synthase (Poly-beta-1, 6-GlcNAc synthase) (EC 2.4.1.-)	*Enterobacter spp. EC-NT1*		A0A3D8XNY3	--	29665424	Acetyl glucosaminyl transferase activity	Not Available
946	Poly-beta-1, 6-N-acetyl-D-glucosamine N-deacetylase PgaB	*Enterobacter spp. EC-NT1*		A0A3D8XP54	--	29665424	Hydrolase activity, acting on carbon-nitrogen (but not peptide) bonds	Not Available
947	Poly-beta-1, 6-N-acetyl-D-glucosamine synthase (Poly-beta-1, 6-GlcNAc synthase) (EC 2.4.1.-)	*Pseudomonas lactis*		A0A7Y1LIK1	--	32587583	Acetyl glucosaminyl transferase activity	Not Available
948	Motility quorum-sensing regulator MqsR	*Agrobacterium rubi*		A0A1B9TWB4	--	27547538	Not Available	Not Available
949	Poly-beta-1, 6-N-acetyl-D-glucosamine biosynthesis protein PgaD	*Pseudomonas lactis*		A0A7Y1LIN2	--	32587583	Not Available	Not Available
950	Poly-beta-1, 6-N-acetyl-D-glucosamine N-deacetylase PgaB	*Pseudomonas spp. GM30*		W6VK76	--	23045501	Hydrolase activity, acting on carbon-nitrogen (but not peptide) bonds	Not Available
951	Poly-beta-1, 6-N-acetyl-D-glucosamine biosynthesis protein PgaD	*Pseudomonas spp. GM30*		W6W1H7	--	23045501	Not Available	Not Available
952	Surface composition regulator	*Enterobacter spp. EC-NT1*		A0A3D8XGN6	--	29665424	Not Available	Not Available
953	Biofilm PGA synthesis protein PgaD	*Pseudomonas spp. Os17*		A0A0D6B9Q4	--	25496595	Not Available	Not Available
954	Uncharacterized protein	*Pseudomonas fuscovaginae*		A0A0N0VKM0	--	26422147	Not Available	Not Available
955	Uncharacterized protein	*Deltaproteobacteria bacterium RIFOXYD12_FULL_50_9*		A0A1F9MN67	--	27774985	Not Available	Not Available
956	Outer membrane N-deacetylase	*Pseudomonas spp. Os17*		A0A0D6BA39	--	25496595	Hydrolase activity, acting on carbon-nitrogen (but not peptide) bonds	Not Available
957	Poly-beta-1, 6-N-acetyl-D-glucosamine synthase (Poly-beta-1, 6-GlcNAc synthase) (EC 2.4.1.-)	*Pseudomonas spp. Os17*		A0A0D6B9S5	--	25496595	Acetyl glucosaminyl transferase activity	Not Available
958	Poly-beta-1, 6-N-acetyl-D-glucosamine synthase (Poly-beta-1, 6-GlcNAc synthase) (EC 2.4.1.-)	*Leclercia spp. LSNIH1*		A0A2I8NRS9	--	29437920	Acetyl glucosaminyl transferase activity	Not Available
959	Poly-beta-1, 6-N-acetyl-D-glucosamine synthase (Poly-beta-1, 6-GlcNAc synthase) (EC 2.4.1.-)	*Pseudomonas fuscovaginae*		A0A0M9GJ16	--	26422147	Acetyl glucosaminyl transferase activity	Not Available
960	Poly-beta-1, 6-N-acetyl-D-glucosamine N-deacetylase PgaB	*Pseudomonas fuscovaginae*		A0A0N0VKP0	--	26422147	Hydrolase activity, acting on carbon-nitrogen (but not peptide) bonds	Not Available
961	Poly-beta-1, 6-N-acetyl-D-glucosamine biosynthesis protein PgaD	*Leclercia spp. LSNIH1*		A0A2I8NRR5	--	29437920	Not Available	Not Available
962	Surface composition regulator	*Salmonella newport*	Strain SL254	A0A0H3BM79	--	21602358	Not Available	Not Available
963	Poly-beta-1, 6-N-acetyl-D-glucosamine synthase (Poly-beta-1, 6-GlcNAc synthase) (EC 2.4.1.-)	*Burkholderia spp. KJ006*		I2DW95	--	22843575	Acetyl glucosaminyl transferase activity	Not Available
964	Poly-beta-1, 6-N-acetyl-D-glucosamine biosynthesis protein PgaD	*Pseudomonas spp. GM80*		J3DTA0	--	23045501	Not Available	Not Available
965	Biofilm PGA synthesis deacetylase PgaB	*Burkholderia spp. KJ006*		I2DW94	--	22843575	Hydrolase activity, acting on carbon-nitrogen (but not peptide) bonds	Not Available
966	Uncharacterized protein	*Acidithiobacillus ferrivorans SS3*		G0JSS8	--	21705598	Not Available	Not Available
967	Uncharacterized protein	*Pseudomonas protegens*	Strain DSM 19095 / LMG 27888 / CFBP 6595 / CHA0	A0A2C9EED5	--	24762936	Not Available	Not Available
968	Surface composition regulator	*Salmonella heidelberg*	Strain SL476	A0A6C6ZPJ5	--	21602358	Not Available	Not Available
969	Surface composition regulator	*Salmonella enterica subspp. enterica serovar*	Lubbock	A0A6C8LYN6	--	31527278	Not Available	Not Available
970	Poly-beta-1, 6-N-acetyl-D-glucosamine biosynthesis protein PgaD	*Xanthomonadales bacterium RIFOXYA1_FULL_69_10*		A0A1G4A9L8	--	27774985	Not Available	Not Available
971	Uncharacterized protein	*Pseudomonas protegens*	Strain DSM 19095 / LMG 27888 / CFBP 6595 / CHA0	A0A2C9EGU0	--	24762936	Not Available	Not Available
972	Poly-beta-1, 6-N-acetyl-D-glucosamine N-deacetylase PgaB	*Enterobacteriaceae bacterium ENNIH2*		A0A2I8QUW8	--	29437920	Hydrolase activity, acting on carbon-nitrogen (but not peptide) bonds	Not Available
973	Poly-beta-1, 6-N-acetyl-D-glucosamine N-deacetylase PgaB (EC 3.5.1.-)	*Pseudomonas protegens*	Strain DSM 19095 / LMG 27888 / CFBP 6595 / CHA0	A0A2C9EE93	--	24762936	Hydrolase activity, acting on carbon-nitrogen (but not peptide) bonds	Not Available
974	Poly-beta-1, 6-N-acetyl-D-glucosamine synthase (Poly-beta-1, 6-GlcNAc synthase) (EC 2.4.1.-)	*Enterobacteriaceae bacterium ENNIH2*		A0A2I8QSF7	--	29437920	Acetyl glucosaminyl transferase activity	Not Available
975	Surface composition regulator	*Salmonella enterica subspp. enterica serovar*	Alachua Str. R6-377	G5LTU2	--	21859443	Not Available	Not Available
976	Poly-beta-1, 6-N-acetyl-D-glucosamine synthase (Poly-beta-1, 6-GlcNAc synthase) (EC 2.4.1.-)	*Pseudomonas silesiensis*		A0A191YLQ5	--	29153257	Acetyl glucosaminyl transferase activity	Not Available
977	mRNA interferase MqsR	*Pseudomonas silesiensis*		A0A191YXN6	--	29153257	Not Available	Not Available
978	Poly-beta-1, 6-N-acetyl-D-glucosamine N-deacetylase PgaB	*Ralstonia pickettii OR214*		R0CKR4	--	23792748	Hydrolase activity, acting on carbon-nitrogen (but not peptide) bonds	Not Available
979	Uncharacterized protein	*Arenimona spp. SCN 70-307*		A0A1D2RX64	--	26031303	Not Available	Not Available
980	Polysaccharide deacetylase (EC 3.-.-.-)	*Sulfurimona gotlandica*	Strain DSM 19862 / JCM 16533 / GD1	H1FST3	--	22203982	Hydrolase activity, acting on carbon-nitrogen (but not peptide) bonds	Not Available
981	mRNA interferase MqsR	*Herbaspirillum aquaticum*		A0A225STC8	--	19671717	Not Available	Not Available
982	Uncharacterized protein	*Sulfurimona gotlandica*	Strain DSM 19862 / JCM 16533 / GD1	B6BJF3	--	22203982	Not Available	Not Available

Target ID	Bacterial Protein Name	Organism Name	Strain	UniProt ID	PDB ID	PubMed ID	Classification as per UniProt GO Molecular Function	Classification of Function as per PDB
983	Poly-beta-1, 6-N-acetyl-D-glucosamine synthase (Poly-beta-1, 6-GlcNAc synthase) (EC 2.4.1.-)	*Ralstonia pickettii OR214*		R0CK10	--	23792748	Acetyl glucosaminyl transferase activity	Not Available
984	Poly-beta-1, 6-N-acetyl-D-glucosamine synthase (Poly-beta-1, 6-GlcNAc synthase) (EC 2.4.1.-)	*Sulfurimona gotlandica*	Strain DSM 19862 / JCM 16533 / GD1	B6BJF4	--	22203982	Acetyl glucosaminyl transferase activity	Not Available
985	Poly-beta-1, 6-N-acetyl-D-glucosamine biosynthesis protein PgaD	*Pseudomonas silesiensis*		A0A191YLN0	--	29153257	Not Available	Not Available
986	Surface composition regulator	*Salmonella enterica subspp. enterica serovar*	Montevideo Str. S5-403	G5Q1S1	--	21859443	Not Available	Not Available
987	Uncharacterized protein	*Lactobacillus spp. ASF360*		N1ZVW5	--	24723722	Not Available	Not Available
988	Poly-beta-1, 6-N-acetyl-D-glucosamine N-deacetylase PgaB	*Pseudomonas silesiensis*		A0A191YLE3	--	29153257	Hydrolase activity, acting on carbon-nitrogen (but not peptide) bonds	Not Available
989	Poly-beta-1, 6-N-acetyl-D-glucosamine N-deacetylase PgaB	*Aeromona veronii Hm21*		A0A7Z3YI03	--	24092791	Hydrolase activity, acting on carbon-nitrogen (but not peptide) bonds	Not Available
990	Uncharacterized protein	*Ralstonia pickettii OR214*		R0E6J7	--	23792748	Not Available	Not Available
991	Poly-beta-1, 6-N-acetyl-D-glucosamine synthase (Poly-beta-1, 6-GlcNAc synthase) (EC 2.4.1.-)	*Aeromona veronii Hm21*		A0A7Z3YHZ5	--	24092791	Acetyl glucosaminyl transferase activity	Not Available
992	mRNA interferase MqsR	*Advenella kashmirensis W13003*		V8QRA6	--	24482505	Not Available	Not Available
993	Uncharacterized protein	*Sphingobium spp. MI1205*		A0A126RE24	--	27056230	Not Available	Not Available
994	Poly-beta-1, 6-N-acetyl-D-glucosamine synthase (Poly-beta-1, 6-GlcNAc synthase) (EC 2.4.1.-)	*Pseudomonas protegens*	Strain DSM 19095 / LMG 27888 / CFBP 6595 / CHA0	A0A2C9EE84	--	24762936	Acetyl glucosaminyl transferase activity	Not Available
995	Poly-beta-1, 6-N-acetyl-D-glucosamine synthase (Poly-beta-1, 6-GlcNAc synthase) (EC 2.4.1.-)	*Xenorhabdus spp. KK7.4*		A0A2D0K576	--	28993611	Acetyl glucosaminyl transferase activity	Not Available
996	Putative intercellular adhesion protein A (Biofilm formation)	*Vibrio spp. AND4*		A8T1C7	--	20436956	Not Available	Not Available
997	Poly-beta-1, 6-N-acetyl-D-glucosamine synthase (Poly-beta-1, 6-GlcNAc synthase) (EC 2.4.1.-)	*Pseudomonas veronii*		A0A0R2ZUD4	--	32587583	Acetyl glucosaminyl transferase activity	Not Available
998	Lipoprotein for biofilm formation	*Bacillus stratosphericus LAMA 585*		M5QYB3	--	23640380	Not Available	Not Available
999	GGDEF domain-containing protein	*Streptomyces coelicolor*	Strain ATCC BAA-471 / A3 2 / M145	Q9AD37	--	12000953	Diguanylate cyclase activity, helicase activity	Not Available
1000	Poly-beta-1, 6-N-acetyl-D-glucosamine synthase (Poly-beta-1, 6-GlcNAc synthase) (EC 2.4.1.-)	*Yersinia entomophaga*		A0A3S6EX22	--	27187466	Acetyl glucosaminyl transferase activity	Not Available
1001	Biofilm PGA synthesis lipoprotein PgaB	*Enterobacter cloacae subspp. cloacae*	Strain ATCC 13047 / DSM 30054 / NBRC 13535 / NCTC 10005 / WDCM 00083 / NCDC 279-56	A0A0H3CQE5	--	20207761	Hydrolase activity, acting on carbon-nitrogen (but not peptide) bonds	Not Available
1002	Uncharacterized protein	*Klebsiella pneumoniae*		Q1G0V3	--	16861646	Not Available	Not Available
1003	Surface composition regulator	*Salmonella enterica subspp. enterica serovar*	Javiana	A0A736M1L3	--	30286803	Not Available	Not Available
1004	Surface composition regulator	*Salmonella enterica subspp. salamae serovar*	42:z:1, 5	A0A735PV97	--	30286803	Not Available	Not Available
1005	IcaA (Fragment)	*Staphylococcus cohnii*		Q83ZH4	--	12957956	Not Available	Not Available
1006	Uncharacterized protein (Fragment)	*Klebsiella pneumoniae*		Q1G0U7	--	16861646	Not Available	Not Available
1007	Uncharacterized protein (Fragment)	*Klebsiella pneumoniae*		Q1G0W1	--	16861646	Not Available	Not Available
1008	Uncharacterized protein	*Klebsiella pneumoniae*		Q1G0V7	--	16861646	Not Available	Not Available
1009	Uncharacterized protein	*Klebsiella pneumoniae*		Q1G0V9	--	16861646	Not Available	Not Available
1010	Aldedh domain-containing protein (Fragment)	*Klebsiella pneumoniae*		Q1G0V2	--	16861646	Oxidoreductase activity, acting on the aldehyde or oxo group of donors, NAD or NADP as acceptor	Not Available
1011	Motility quorum-sensing regulator mqsR	*Yersinia enterocolitica W22703*		F4N438	--	21453472	Not Available	Not Available
1012	Uncharacterized protein	*Klebsiella pneumoniae*		Q1G0V6	--	16861646	Not Available	Not Available
1013	Surface composition regulator	*Salmonella enterica subspp. enterica serovar*	Panama	A0A751YZQ8	--	30286803	Not Available	Not Available
1014	Motility quorum-sensing regulator mqsR	*uncultured Desulfobacterium spp.*		E1YKP6	--	21176053	Not Available	Not Available
1015	Uncharacterized protein (Fragment)	*Klebsiella pneumoniae*		Q1G0V1	--	16861646	Not Available	Not Available
1016	Uncharacterized protein (Fragment)	*Klebsiella pneumoniae*		Q1G0U3	--	16861646	Not Available	Not Available
1017	Uncharacterized protein	*Klebsiella pneumoniae*		Q1G0U1	--	16861646	Not Available	Not Available
1018	PTS EIIB type-1 domain-containing protein (Fragment)	*Klebsiella pneumoniae*		Q1G0V0	--	16861646	Protein-N(PI)-phosphohistidine-sugar phosphoTransferase activity	Not Available
1019	Biofilm PGA synthesis protein PgaD	*Pseudomonas moraviensis R28-S*		V8REE0	--	24558233	Not Available	Not Available
1020	Biofilm PGA synthesis protein PgaD, putative	*Pseudomonas fluorescens Q2-87*		J2ERK9	--	22792073	Not Available	Not Available
1021	Surface composition regulator	*Salmonella enterica subspp. enterica serovar*	Javiana	A0A733VDE6	--	30286803	Not Available	Not Available
1022	Poly-beta-1, 6-N-acetyl-D-glucosamine synthase (Poly-beta-1, 6-GlcNAc synthase) (EC 2.4.1.-)	*Pseudomonas fluorescens Q2-87*		J2XWR0	--	22792073	Acetyl glucosaminyl transferase activity	Not Available
1023	Surface composition regulator	*Salmonella enterica subspp. salamae serovar*	30:1, z28:z6	A0A737XXT1	--	30286803	Not Available	Not Available
1024	Biofilm PGA synthesis lipoprotein PgaB (EC 3.-.-.-)	*Pseudomonas fluorescens Q2-87*		J2MEN5	--	22792073	Hydrolase activity, acting on carbon-nitrogen (but not peptide) bonds	Not Available
1025	Surface composition regulator	*Salmonella enterica subspp. salamae serovar*	58:l, z13, z28:z6	A0A734HKS5	--	30286803	Not Available	Not Available
1026	Uncharacterized protein	*Shewanella spp. 33B*		B9W0T4	--	19336243	Not Available	Not Available
1027	Motility quorum-sensing regulator MqsR	*Pseudomonas fluorescens Q2-87*		J2YB16	--	22792073	Not Available	Not Available
1028	Surface composition regulator	*Salmonella enterica subspp. enterica serovar*	Typhi Str. CT18	A0A714V9M2	--	30286803	Not Available	Not Available
1029	UPF0441 protein GKC49_07595	*Enterobacter agglomerans*	Erwinia herbicola Pantoea agglomerans	A0A6I6K1P2	--	32966572	Not Available	Not Available
1030	Surface composition regulator	*Salmonella enterica subspp. salamae*		A0A3U3WQ67	--	30286803	Not Available	Not Available
1031	Biofilm formation regulator BssS (Biofilm formation regulatory protein BssS)	*Enterobacter agglomerans*	Erwinia herbicola Pantoea agglomerans	A0A356RXP9	--	32966572	Not Available	Not Available
1032	Hha toxicity modulator TomB (Toxin overexpression modulator in biofilms)	*Enterobacter agglomerans*	Erwinia herbicola Pantoea agglomerans	A0A1T4JJK3	--	32966572	Not Available	Not Available
1033	Surface composition regulator	*Salmonella enterica subspp. enterica serovar*	Panama	A0A3T2YKC2	--	30286803	Not Available	Not Available
1034	Poly-beta-1, 6-N-acetyl-D-glucosamine synthase (Poly-beta-1, 6-GlcNAc synthase) (EC 2.4.1.-)	*Pusillimona spp.*		A0A2E4R4G5	--	29337314	Acetyl glucosaminyl transferase activity	Not Available
1035	Surface composition regulator	*Salmonella enterica subspp. enterica serovar*	Reading	A0A5H8IBP9	--	30286803	Not Available	Not Available
1036	Poly-beta-1, 6-N-acetyl-D-glucosamine biosynthesis protein PgaD	*Pusillimona spp.*		A0A2E4R6T6	--	29337314	Not Available	Not Available

Target ID	Bacterial Protein Name	Organism Name	Strain	UniProt ID	PDB ID	PubMed ID	Classification as per UniProt GO Molecular Function	Classification of Function as per PDB
1037	Biofilm PGA synthesis auxiliary protein PgaD (Poly-beta-1, 6-N-acetyl-D-glucosamine biosynthesis protein PgaD)	*Pseudomonas synxantha*		A0A0R2YFQ5	--	27231365	Not Available	Not Available
1038	Poly-beta-1, 6-N-acetyl-D-glucosamine synthase (Poly-beta-1, 6-GlcNAc synthase) (EC 2.4.1.-)	*Staphylococcus pseudintermedius*		A0A317YUG5	--	29292005; 30173756	Acetyl glucosaminyl transferase activity	Not Available
1039	Surface composition regulator	*Salmonella enterica subspp. enterica serovar*	Litchfield	A0A3W0FDC6	--	30286803	Not Available	Not Available
1040	Poly-beta-1, 6-N-acetyl-D-glucosamine synthase (Poly-beta-1, 6-GlcNAc synthase) (EC 2.4.1.-)	*Piscirickettsiaceae bacterium*		A0A2A4W8B8	--	29099490	Acetyl glucosaminyl transferase activity	Not Available
1041	Poly-beta-1, 6-N-acetyl-D-glucosamine synthase (Poly-beta-1, 6-GlcNAc synthase) (EC 2.4.1.-)	*Xanthomonas cucurbitae*		A0A2S7DKF0	--	33734871	Acetyl glucosaminyl transferase activity	Not Available
1042	Surface composition regulator	*Salmonella enterica subspp. enterica serovar*	Bovismorbificans	A0A630XF19	--	30286803	Not Available	Not Available
1043	Biofilm PGA synthesis lipoprotein PgaB (Poly-beta-1, 6-N-acetyl-D-glucosamine N-deacetylase)	*Pseudomonas baetica*		A0A2N0DQC4	--	29579234	Hydrolase activity, acting on carbon-nitrogen (but not peptide) bonds	Not Available
1044	Enoyl-CoA hydratase	*Acinetobacter oleivorans*	Strain JCM 16667 / KCTC 23045 / DR1	D3JTG2	--	20629796; 20639327	catalytic activity	Not Available
1045	Motility quorum-sensing regulator / GCU-specific mRNA interferase toxin	*Nitrosospira multiformis*	Strain ATCC 25196 / NCIMB 11849 / C 71	Q2Y6X0	--	18390676	Not Available	Not Available
1046	Outer membrane N-deacetylase (EC 3.5.1.-) (Poly-beta-1, 6-N-acetyl-D-glucosamine N-deacetylase) (Poly-beta-1, 6-N-acetyl-D-glucosamine N-deacetylase PgaB)	*Klebsiella pneumoniae*		A0A2V3JZQ6	--	30373801; 32429555	Hydrolase activity, acting on carbon-nitrogen (but not peptide) bonds	Not Available
1047	Poly-beta-1, 6-N-acetyl-D-glucosamine synthase (Poly-beta-1, 6-GlcNAc synthase) (EC 2.4.1.-)	*Carnobacterium divergens*	Lactobacillus divergens	A0A5F0MNT9	--	30081251	Acetyl glucosaminyl transferase activity	Not Available
1048	Poly-beta-1, 6-N-acetyl-D-glucosamine synthase (Poly-beta-1, 6-GlcNAc synthase) (EC 2.4.1.-)	*Enterobacter hormaechei*		A0A331VAX8	--	30286803	Acetyl glucosaminyl transferase activity	Not Available
1049	Biofilm PGA synthesis deacetylase, xylanase / chitin deacetylase (Hemin storage protein) (Poly-beta-1, 6-N-acetyl-D-glucosamine N-deacetylase PgaB)	*Acinetobacter baumannii*		A0A219CD45	--	30124845	Hydrolase activity, acting on carbon-nitrogen (but not peptide) bonds, Hydrolase activity, acting on glycosyl bonds	Not Available
1050	Poly-beta-1, 6-N-acetyl-D-glucosamine synthase (Poly-beta-1, 6-GlcNAc synthase) (EC 2.4.1.-)	*Acinetobacter baumannii*		A0A505MFJ4	--	29137647	Acetyl glucosaminyl transferase activity	Not Available
1051	Poly-beta-1, 6-N-acetyl-D-glucosamine biosynthesis protein PgaD	*Klebsiella pneumoniae*		A0A0S3FBI0	--	30373801; 32429555	Not Available	Not Available
1052	Uncharacterized protein	*Companilactobacillus farciminis*		A0A0H4LUD2	--	26383668	Not Available	Not Available
1053	Poly-beta-1, 6-N-acetyl-D-glucosamine synthase (Poly-beta-1, 6-GlcNAc synthase) (EC 2.4.1.-)	*Carnobacterium divergens*	Lactobacillus divergens	A0A2R8A3Q4	--	30081251	Acetyl glucosaminyl transferase activity	Not Available
1054	Poly-beta-1, 6-N-acetyl-D-glucosamine biosynthesis protein PgaD	*Acinetobacter baumannii*		A0A0J8V218	--	29437920	Not Available	Not Available
1055	Surface composition regulator	*Salmonella derby*		A0A3W0AU60	--	30286803; 30902850	Not Available	Not Available
1056	Fimbrial protein (Fimbrial-like protein) (MrkF) (Type 1 fimbrial protein) (Type 3 fimbria minor subunit MrkF)	*Klebsiella pneumoniae*		A0A0W8ANM7	--	21901098; 30373801; 29437920; 32429555	Not Available	Not Available
1057	Surface composition regulator	*Salmonella oranienberg*		A0A3V8Z2E2	--	30286803	Not Available	Not Available
1058	Poly-beta-1, 6-N-acetyl-D-glucosamine synthase (Poly-beta-1, 6-GlcNAc synthase) (EC 2.4.1.-)	*Acinetobacter ursingii*		A0A3F3LB10	--	28293292	Acetyl glucosaminyl transferase activity	Not Available
1059	Biofilm PGA synthesis deacetylase PgaB (Poly-beta-1, 6-N-acetyl-D-glucosamine N-deacetylase PgaB)	*Acinetobacter ursingii*		A0A3F3L5D6	--	28293292	Hydrolase activity, acting on carbon-nitrogen (but not peptide) bonds	Not Available
1060	Surface composition regulator	*Salmonella montevideo*		A0A3A3J9M4	--	30286803; 31527278	Not Available	Not Available
1061	Poly-beta-1, 6-N-acetyl-D-glucosamine synthase (Poly-beta-1, 6-GlcNAc synthase) (EC 2.4.1.-)	*Klebsiella pneumoniae*		A0A0W8A3S5	--	25341126; 30373801; 32429555	Acetyl glucosaminyl transferase activity	Not Available
1062	Poly-beta-1, 6-N-acetyl-D-glucosamine synthase (Poly-beta-1, 6-GlcNAc synthase) (EC 2.4.1.-)	*Acinetobacter baumannii*		V5VBU0	--	27917170; 29137647; 30124845	Acetyl glucosaminyl transferase activity	Not Available
1063	Poly-beta-1, 6-N-acetyl-D-glucosamine synthase (Poly-beta-1, 6-GlcNAc synthase) (EC 2.4.1.-)	*Citrobacter pasteurii*		A0A6N6JYD6	--	30087402	Acetyl glucosaminyl transferase activity	Not Available
1064	Poly-beta-1, 6-N-acetyl-D-glucosamine synthase (Poly-beta-1, 6-GlcNAc synthase) (EC 2.4.1.-)	*Acinetobacter baumannii*		A0A059ZRE0	--	25136007; 25845845; 26170289; 27917170; 30124845; 29437920	Acetyl glucosaminyl transferase activity	Not Available
1065	PgaD (Poly-beta-1, 6-N-acetyl-D-glucosamine biosynthesis protein PgaD)	*Acinetobacter baumannii*		C8YYH8	--	19633088	Not Available	Not Available
1066	Biofilm PGA synthesis protein pgaD (Poly-beta-1, 6-N-acetyl-D-glucosamine biosynthesis protein PgaD)	*Acinetobacter baumannii*		A0A0E1JKH0	--	25845845; 27917170	Not Available	Not Available
1067	Biofilm synthesis deacetylase PgaB (Lipoprotein HmsF) (EC 3.5.1.-) (Poly-beta-1, 6-N-acetyl-D-glucosamine N-deacetylase PgaB)	*Acinetobacter baumannii*		V5VFW3	--	30124845; 29437920	Hydrolase activity, acting on carbon-nitrogen (but not peptide) bonds	Not Available
1068	Poly-beta-1, 6-N-acetyl-D-glucosamine synthase (Poly-beta-1, 6-GlcNAc synthase) (EC 2.4.1.-) (Fragment)	*Citrobacter freundii*		V5YMI7	--	28636609; 30286803; 30087402; 32766684	Acetyl glucosaminyl transferase activity	Not Available
1069	Hemin storage system protein (Poly-beta-1, 6-N-acetyl-D-glucosamine biosynthesis protein PgaD)	*Enterobacter hormaechei*		A0A156GTQ3	--	29088362	Not Available	Not Available
1070	DNA invertase Pin homolog (Recombinae family protein) (Resolvase)	*Escherichia coli*		B0ZE07	--	15328093; 18440636; 19717626; 31642700	DNA binding, DNA strand exchange activity	Not Available
1071	InsB (Insertion element protein) (Transposase) (Transposase (Identified by ISEscan HMM))	*Escherichia coli*		B0LHG4	--	18055599; 19717626; 26488485	Not Available	Not Available
1072	Conjugal transfer protein (Killer protein) (TrbM-domain protein)	*Escherichia coli*		B0ZDX4	--	15328093; 18440636; 19717626; 22479389; 31652858	Not Available	Not Available
1073	Poly-beta-1, 6-N-acetyl-D-glucosamine synthase (Poly-beta-1, 6-GlcNAc synthase) (EC 2.4.1.-)	*Escherichia coli*		A0A366YGF8	--	30286803	Acetyl glucosaminyl transferase activity	Not Available
1074	MrkD fimbrial protein (Type 3 fimbria adhesin subunit MrkD)	*Escherichia coli*		B0ZDW1	--	15328093; 18440636	Not Available	Not Available
1075	Uncharacterized protein	*Klebsiella pneumoniae*		Q1G0U8	--	16861646	Not Available	Not Available
1076	Poly-beta-1, 6-N-acetyl-D-glucosamine synthase (Poly-beta-1, 6-GlcNAc synthase) (EC 2.4.1.-)	*Escherichia coli*		A0A7B5TBK4	--	30286803	Acetyl glucosaminyl transferase activity	Not Available

Target ID	Bacterial Protein Name	Organism Name	Strain	UniProt ID	PDB ID	PubMed ID	Classification as per UniProt GO Molecular Function	Classification of Function as per PDB
1077	Fimbriae stability-associated protein (Fimbrial protein) (MrkF) (MrkF fimbrial protein) (MrkF fimbrial protein putative assembly / stability function) (Type 1 fimbrial protein) (Type 3 fimbria minor subunit MrkF)	*Escherichia coli*		B0LHG9	--	15328093; 18055599; 18440636; 19717626; 22479389; 26441869; 30286803; 31652858	Not Available	Not Available
1078	Poly-beta-1, 6-N-acetyl-D-glucosamine synthase (Poly-beta-1, 6-GlcNAc synthase) (EC 2.4.1.-)	*Escherichia coli*		A0A0D6IFI2	--	28686503; 30286803; 31477907; 32021174	Acetyl glucosaminyl transferase activity	Not Available
1079	PGA biosynthesis protein (Poly-beta-1, 6-N-acetyl-D-glucosamine biosynthesis protein PgaD)	*Escherichia coli*		A0A0J2DWH8	--	28686503; 30286803	Not Available	Not Available
1080	Cyanide hydratase (EC 3.1.-.-) (GCU-specific mRNA interferase toxin of the MqsR-MqsA toxin-antitoxin system) (GCU-specific mRNA interferase toxin of the MqsRA toxin-antitoxin system) (Type II toxin-antitoxin system MqsR family toxin) (Type II toxin-antitoxin system toxin MqsR) (EC 3.1.26.-) (mRNA interferase MqsR)	*Escherichia coli*		J7QHJ7	--	23275093; 28686503; 30286803; 29437920; 31642700; 31652858; 31477907; 30947268; 30948755; 32021174; 32739475; 32599678	Hydrolase activity	Not Available
1081	Poly-beta-1, 6-N-acetyl-D-glucosamine biosynthesis protein PgaD	*Chromobacterium haemolyticum*		A0A2T5P0R2	--	29670144	Not Available	Not Available
1082	Poly-beta-1, 6-N-acetyl-D-glucosamine biosynthesis (Poly-beta-1, 6-N-acetyl-D-glucosamine biosynthesis protein PgaD)	*Escherichia coli*		A0A066SWE3	--	30286803; 31477907	Not Available	Not Available
1083	H-HA (Haemolysin expression modulating protein Hha) (Hemolysin activation protein) (Hemolysin expression modulating protein) (Hemolysin expression modulator Hha) (Hha) (Hha expression modulating protein) (Hha expression modulation protein) (Histone-like protein) (Putative hemolysin expression modulating protein)	*Escherichia coli*		B0ZDX1	--	15328093; 18440636; 19717626; 21831988; 22479389; 23836183; 25403661; 30286803; 31652858; 31477907	Not Available	Not Available
1084	Poly-beta-1, 6-N-acetyl-D-glucosamine synthase (Poly-beta-1, 6-GlcNAc synthase) (EC 2.4.1.-)	*Escherichia coli*		A0A1Y2XXH9	--	30286803	Acetyl glucosaminyl transferase activity	Not Available
1085	Surface composition regulator	*Klebsiella pneumoniae*		A0A080SUH1	--	30373801; 29437920; 32429555	Not Available	Not Available
1086	Outer membrane N-deacetylase (Poly-beta-1, 6-N-acetyl-D-glucosamine N-deacetylase PgaB) (EC 3.5.1.-)	*Citrobacter freundii*		A0A0P8KEL7	--	28636609; 30286803	Hydrolase activity, acting on carbon-nitrogen (but not peptide) bonds	Not Available
1087	Poly-beta-1, 6-N-acetyl-D-glucosamine biosynthesis protein PgaD	*Citrobacter freundii*		A0A0P8I821	--	28636609; 30286803	Not Available	Not Available
1088	Fimbria adhesin protein (Fimbrial protein) (MrkD) (MrkD protein) (Type 3 fimbria adhesin subunit MrkD)	*Klebsiella pneumoniae*		G3FT07	--	21901098; 24123820; 25341126; 30373801; 29437920; 32429555	Not Available	Not Available
1089	Hemin storage system protein (Poly-beta-1, 6-N-acetyl-D-glucosamine biosynthesis protein PgaD)	*Klebsiella pneumoniae*		A0A0C7KL91	--	30373801; 32429555	Not Available	Not Available
1090	Surface composition regulator	*Citrobacter freundii*		A0A133LI55	--	30286803; 30087402; 32766684	Not Available	Not Available
1091	Outer membrane N-deacetylase (Poly-beta-1, 6-N-acetyl-D-glucosamine N-deacetylase PgaB) (EC 3.5.1.-)	*Citrobacter freundii*		A0A0D7M2B6	--	30087402; 32766684	Hydrolase activity, acting on carbon-nitrogen (but not peptide) bonds	Not Available
1092	Ferrous iron transporter B (Fimbrial protein) (MrkA) (MrkA protein) (Type 1 fimbrial protein) (Type 3 fimbria major subunit MrkA)	*Klebsiella pneumoniae*		G3FT04	--	21901098; 25341126; 30373801; 29437920; 32429555	Not Available	Not Available
1093	Surface composition regulator	*Salmonella thompson*		A0A403SPK3	--	30286803	Not Available	Not Available
1094	Poly-beta-1, 6-N-acetyl-D-glucosamine biosynthesis protein PgaD	*Acinetobacter ursingii*		A0A3D2SIV6	--	30148503	Not Available	Not Available
1095	Autoinducer 2 import system permease LsrD (Autoinducer-2 ABC transporter permease LsrD) (Autoinducer-2 ABC transporter permease protein)	*Klebsiella pneumoniae*		C8BNA7	--	20600864; 30373801; 32429555	Transmembrane transporter activity	Not Available
1096	Surface composition regulator	*Klebsiella pneumoniae*		W9B3F6	--	25341126	Not Available	Not Available
1097	Surface composition regulator	*Salmonella enterica subspp. enterica serovar*	Kentucky	A0A2T8L9D1	--	30286803; 31715658; 31527278	Not Available	Not Available
1098	Surface composition regulator	*Salmonella enterica subspp. enterica serovar*	4, [5], 12:i:-	A0A315I6K6	--	30286803	Not Available	Not Available
1099	AI-2 transport protein TqsA (AI-2E family transporter) (PerM family permease) (Permease) (Putative transport protein)	*Klebsiella pneumoniae*		C8BNA8	--	20600864; 30373801; 29437920; 32429555	Not Available	Not Available
1100	Surface composition regulator	*Citrobacter freundii*		A0A0A5R3I1	--	28636609	Not Available	Not Available
1101	Surface composition regulator	*Salmonella senftenberg*		A0A3R0HNH3	--	30286803	Not Available	Not Available
1102	Biofilm PGA synthesis auxiliary protein PgaD (Biofilm PGA synthesis protein PgaD) (Hemin storage protein) (Poly-beta-1, 6-N-acetyl-D-glucosamine biosynthesis protein PgaD)	*Citrobacter freundii*		A0A0D7LKL1	--	30286803; 30087402; 32766684	Not Available	Not Available
1103	Outer membrane N-deacetylase (Poly-beta-1, 6-N-acetyl-D-glucosamine N-deacetylase) (EC 3.5.1.-) (Poly-beta-1, 6-N-acetyl-D-glucosamine N-deacetylase PgaB)	*Escherichia coli*		A0A0B0VSG0	--	29437920	Hydrolase activity, acting on carbon-nitrogen (but not peptide) bonds	Not Available
1104	Surface composition regulator	*Escherichia coli*		A0A0K4QSN3	--	30286803	Not Available	Not Available
1105	Biofilm PGA synthesis protein PgaD (Poly-beta-1, 6-N-acetyl-D-glucosamine biosynthesis protein PgaD)	*Escherichia coli*		A0A2X5E7W5	--	30286803	Not Available	Not Available

Target ID	Bacterial Protein Name	Organism Name	Strain	UniProt ID	PDB ID	PubMed ID	Classification as per UniProt GO Molecular Function	Classification of Function as per PDB
1106	Lipoprotein YcdR (Outer membrane N-deacetylase) (Poly-beta-1, 6-N-acetyl-D-glucosamine (PGA) N-deacetylase outer membrane export lipoprotein) (Poly-beta-1, 6-N-acetyl-D-glucosamine N-deacetylase) (Poly-beta-1, 6-N-acetyl-D-glucosamine N-deacetylase PgaB) (EC 3.5.1.-) (Poly-beta-1, 6-N-acetyl-D-glucosamine N-deacetylase outer membrane export lipoprotein)	*Escherichia coli*		J7QZQ0	--	23275093; 26441869; 30286803; 30924410; 31642700; 31652858; 31477907; 30948755; 32739475; 28953937	Hydrolase activity, acting on carbon-nitrogen (but not peptide) bonds	Not Available
1107	Poly-beta-1, 6-N-acetyl-D-glucosamine N-deacetylase PgaB (EC 3.5.1.-)	*Escherichia coli*		A0A061KYR7	--	30286803; 31477907	Hydrolase activity, acting on carbon-nitrogen (but not peptide) bonds	Not Available
1108	Conjugal transfer protein (Hypothetical lipoprotein) (Putative lipoprotein)	*Escherichia coli*		B0ZDX5	--	15328093; 18440636; 19717626; 22479389	Not Available	Not Available
1109	Poly-beta-1, 6-N-acetyl-D-glucosamine N-deacetylase PgaB (EC 3.5.1.-)	*Escherichia coli*		A0A3L1M483	--	30286803	Hydrolase activity, acting on carbon-nitrogen (but not peptide) bonds	Not Available
1110	Lipoprotein YcdR (EC 3.5.1.-) (Poly-beta-1, 6-N-acetyl-D-glucosamine N-deacetylase) (Poly-beta-1, 6-N-acetyl-D-glucosamine N-deacetylase PgaB)	*Escherichia coli*		C3TEU9	--	19439656	Hydrolase activity, acting on carbon-nitrogen (but not peptide) bonds	Not Available
1111	Surface composition regulator	*Escherichia coli*		A0A2X5FFT5	--	30286803	Not Available	Not Available
1112	Putative methyl-accepting chemotaxis protein	*Salmonella typhimurium*	Strain LT2 / SGSC1412 / ATCC 700720	Q8ZPW4	--	11677609	Diguanylate cyclase activity	Not Available
1113	Poly-beta-1, 6-N-acetyl-D-glucosamine synthase (Poly-beta-1, 6-GlcNAc synthase) (EC 2.4.1.-)	*Acinetobacter baumannii*	Strain AB307-0294	A0A5K6CTW7	--	18931120	Acetyl glucosaminyl transferase activity	Not Available
1114	Putative signaling protein	*Clostridium botulinum*	Strain Hall / ATCC 3502 / NCTC 13319 / Type A	A5HXY9	--	17519437	Diguanylate cyclase activity	Not Available
1115	Fimbrial subunit	*Salmonella typhimurium*	Strain LT2 / SGSC1412 / ATCC 700720	Q8ZS05	--	11677609	Not Available	Not Available
1116	Putative signaling protein	*Clostridium botulinum*	Strain Hall / ATCC 3502 / NCTC 13319 / Type A	A5HYM7	--	17519437	Diguanylate cyclase activity	Not Available
1117	Putative diguanylate cyclase / phosphodiesterase domain 1	*Salmonella typhimurium*	Strain LT2 / SGSC1412 / ATCC 700720	H9L4B6	--	11677609	Diguanylate cyclase activity	Not Available
1118	Putative signaling protein	*Clostridium botulinum*	Strain Hall / ATCC 3502 / NCTC 13319 / Type A	A5I1K0	--	17519437	Diguanylate cyclase activity	Not Available
1119	GGDEF family protein	*Deinococcus radiodurans*	Strain ATCC 13939 / DSM 20539 / JCM 16871 / LMG 4051 / NBRC 15346 / NCIMB 9279 / R1 / VKM B-1422	Q9RYX3	--	10567266	Diguanylate cyclase activity, phosphorelay sensor kinase activity	Not Available
1120	Putative fimbrial chaperone protein	*Salmonella typhimurium*	Strain LT2 / SGSC1412 / ATCC 700720	Q8ZJT9	--	11677609	Not Available	Not Available
1121	Putative outer Membrane protein	*Salmonella typhimurium*	Strain LT2 / SGSC1412 / ATCC 700720	Q8ZQ03	--	11677609	Not Available	Not Available
1122	Putative signaling protein	*Clostridium botulinum*	Strain Hall / ATCC 3502 / NCTC 13319 / Type A	A5I786	--	17519437	Diguanylate cyclase activity	Not Available
1123	Putative diguanylate cyclase / phosphodiesterase	*Salmonella typhimurium*	Strain LT2 / SGSC1412 / ATCC 700720	Q8ZMW4	--	11677609	Diguanylate cyclase activity	Not Available
1124	Putative diguanylate cyclase / phosphodiesterase domain 1 containing protein	*Salmonella typhimurium*	Strain LT2 / SGSC1412 / ATCC 700720	Q8ZJW8	--	11677609	Diguanylate cyclase activity	Not Available
1125	Uncharacterized protein	*Acinetobacter baumannii*	Strain AB307-0294	A0A5K6CUI0	--	18931120	Not Available	Not Available
1126	Putative fimbrial subunit	*Salmonella typhimurium*	Strain LT2 / SGSC1412 / ATCC 700720	H9L482	--	11677609	Not Available	Not Available
1127	Fimbrial subunit	*Salmonella typhimurium*	Strain LT2 / SGSC1412 / ATCC 700720	H9L4A8	--	11677609	Not Available	Not Available
1128	Putative signaling protein	*Clostridium botulinum*	Strain Hall / ATCC 3502 / NCTC 13319 / Type A	A5I1D2	--	17519437	Diguanylate cyclase activity	Not Available
1129	Biofilm PGA synthesis protein PgaD	*Acinetobacter baumannii*	Strain AB307-0294	A0A5K6CPI1	--	18931120	Not Available	Not Available
1130	Putative fimbrial subunit	*Salmonella typhimurium*	Strain LT2 / SGSC1412 / ATCC 700720	Q8ZJU2	--	11677609	Not Available	Not Available
1131	Poly-beta-1, 6-N-acetyl-D-glucosamine N-deacetylase (EC 3.5.1.-)	*Acinetobacter baumannii*	Strain AB307-0294	A0A5K6CU40	--	18931120	Hydrolase activity, acting on carbon-nitrogen (but not peptide) bonds	Not Available
1132	GGDEF family protein	*Deinococcus radiodurans*	Strain ATCC 13939 / DSM 20539 / JCM 16871 / LMG 4051 / NBRC 15346 / NCIMB 9279 / R1 / VKM B-1422	Q9RVD9	--	10567266	Diguanylate cyclase activity, phosphorelay sensor kinase activity	Not Available
1133	Fimbrial subunit	*Salmonella typhimurium*	Strain LT2 / SGSC1412 / ATCC 700720	H9L408	--	11677609	Not Available	Not Available
1134	Putative minor fimbrial subunit	*Salmonella typhimurium*	Strain LT2 / SGSC1412 / ATCC 700720	H9L4A9	--	11677609	Not Available	Not Available
1135	Putative fimbrial subunit	*Salmonella typhimurium*	Strain LT2 / SGSC1412 / ATCC 700720	Q8ZRR4	--	11677609	Not Available	Not Available
1136	Putative fimbriae	*Salmonella typhimurium*	Strain LT2 / SGSC1412 / ATCC 700720	Q8ZRR7	--	11677609	Not Available	Not Available
1137	Putative signaling protein	*Clostridium botulinum*	Strain Hall / ATCC 3502 / NCTC 13319 / Type A	A5I3D2	--	17519437	Diguanylate cyclase activity	Not Available
1138	Poly-beta-1, 6-N-acetyl-D-glucosamine synthase (Poly-beta-1, 6-GlcNAc synthase) (EC 2.4.1.-)	*Photobacterium profundum*	Strain SS9	Q6LK51	--	15746425	Acetyl glucosaminyl transferase activity	Not Available
1139	Putative minor fimbrial subunit	*Salmonella typhimurium*	Strain LT2 / SGSC1412 / ATCC 700720	Q8ZRP9	--	11677609	Not Available	Not Available

Target ID	Bacterial Protein Name	Organism Name	Strain	UniProt ID	PDB ID	PubMed ID	Classification as per UniProt GO Molecular Function	Classification of Function as per PDB
1140	GGDEF family protein	*Deinococcus radiodurans*	Strain ATCC 13939 / DSM 20539 / JCM 16871 / LMG 4051 / NBRC 15346 / NCIMB 9279 / R1 / VKM B-1422	Q9RWR1	--	10567266	Diguanylate cyclase activity	Not Available
1141	NodB homology domain-containing protein	*Photobacterium profundum*	Strain SS9	Q6LK50	--	15746425	Hydrolase activity, acting on carbon-nitrogen (but not peptide) bonds	Not Available
1142	Polysaccharide deacetylase	*Halomonas spp. TD01*		F7SRR6	--	22040376	Hydrolase activity, acting on carbon-nitrogen (but not peptide) bonds	Not Available
1143	Diguanylate cyclase with PAS / PAC sensor	*Dictyoglomus turgidum*	Strain DSM 6724 / Z-1310	B8DYG5	--	28066333	Diguanylate cyclase activity	Not Available
1144	Type II toxin-antitoxin system MqsR family toxin	*Desulfuromonadales bacterium*		A0A7C8ARH6	--	32737307	Not Available	Not Available
1145	Uncharacterized protein	*Proteobacteria bacterium SG_bin4*		A0A1W9GZE0	--	28390307	Not Available	Not Available
1146	Diguanylate cyclase	*Dictyoglomus turgidum*	Strain DSM 6724 / Z-1310	B8E0L1	--	28066333	Diguanylate cyclase activity	Not Available
1147	Diguanylate cyclase	*Dictyoglomus turgidum*	Strain DSM 6724 / Z-1310	B8E1P2	--	28066333	Diguanylate cyclase activity	Not Available
1148	Poly-beta-1, 6-N-acetyl-D-glucosamine synthase (Poly-beta-1, 6-GlcNAc synthase) (EC 2.4.1.-)	*Klebsiella pneumoniae subspp. rhinoscleromatis*	SB3432	R4Y4T4	--	21629720	Acetyl glucosaminyl transferase activity	Not Available
1149	Diguanylate cyclase	*Dictyoglomus turgidum*	Strain DSM 6724 / Z-1310	B8DZI2	--	28066333	Diguanylate cyclase activity	Not Available
1150	Diguanylate cyclase	*Dictyoglomus turgidum*	Strain DSM 6724 / Z-1310	B8E392	--	28066333	Diguanylate cyclase activity	Not Available
1151	Poly-beta-1, 6-N-acetyl-D-glucosamine synthase (Poly-beta-1, 6-GlcNAc synthase) (EC 2.4.1.-)	*Romboutsia weinsteinii*		A0A371J6B9	--	28982987	Acetyl glucosaminyl transferase activity	Not Available
1152	Uncharacterized protein	*Rhizobium freirei PRF 81*		N6UY50	--	23270491	Not Available	Not Available
1153	Uncharacterized protein	*Novosphingobium barchaimii LL02*		A0A0J7XQU0	--	25850427	Not Available	Not Available
1154	Poly-beta-1, 6-N-acetyl-D-glucosamine biosynthesis protein PgaD	*Elusimicrobia bacterium RIFOXYA2_FULL_50_26*		A0A1F9WSX0	--	27774985	Not Available	Not Available
1155	Uncharacterized protein	*Pseudomonas spp. GM21*		J3EX79	--	23045501	Not Available	Not Available
1156	Poly-beta-1, 6-N-acetyl-D-glucosamine N-deacetylase PgaB	*Escherichia spp.*		A0A352MKB2	--	30148503	Hydrolase activity, acting on carbon-nitrogen (but not peptide) bonds	Not Available
1157	Poly-beta-1, 6-N-acetyl-D-glucosamine synthase (Poly-beta-1, 6-GlcNAc synthase) (EC 2.4.1.-)	*Elusimicrobia bacterium RIFOXYA2_FULL_50_26*		A0A1F9WSW2	--	27774985	Acetyl glucosaminyl transferase activity	Not Available
1158	Poly-beta-1, 6-N-acetyl-D-glucosamine synthase (Poly-beta-1, 6-GlcNAc synthase) (EC 2.4.1.-)	*Escherichia spp.*		A0A352MKB1	--	30148503	Acetyl glucosaminyl transferase activity	Not Available
1159	Poly-beta-1, 6-N-acetyl-D-glucosamine biosynthesis protein PgaD	*Escherichia spp.*		A0A352MKB0	--	30148503	Not Available	Not Available
1160	Uncharacterized protein	*Geobacteraceae bacterium GWC2_58_44*		A0A1G0MDW5	--	27774985	Not Available	Not Available
1161	Lipoprotein for biofilm formation	*Bacillus atrophaeus*	Strain 1942	A0A0H3E3Y9	--	21464989	Not Available	Not Available
1162	Surface composition regulator	*Escherichia coli O111:H11 str. CVM9455*		K4WSM6	--	23052305	Not Available	Not Available
1163	Poly-beta-1, 6-N-acetyl-D-glucosamine N-deacetylase PgaB	*Pectobacterium polonicum*		A0A4V6ESD3	--	30973319	Hydrolase activity, acting on carbon-nitrogen (but not peptide) bonds	Not Available
1164	Type II toxin-antitoxin system MqsR family toxin	*Escherichia coli*		A0A6M0Q153	--	16943019	Not Available	Not Available
1165	Poly-beta-1, 6-N-acetyl-D-glucosamine N-deacetylase PgaB	*Escherichia coli*		A0A7B6D4F3	--	30286803	Hydrolase activity, acting on carbon-nitrogen (but not peptide) bonds	Not Available
1166	Poly-beta-1, 6-N-acetyl-D-glucosamine biosynthesis protein PgaD	*Escherichia coli*		A0A792HNV3	--	30286803	Not Available	Not Available
1167	Uncharacterized protein	*Desulfuromonadales bacterium GWC2_61_20*		A0A1F9PX82	--	27774985	Not Available	Not Available
1168	Predicted enzyme associated with biofilm formation	*Escherichia coli O1:K1 / APEC*		A0A0H2YXW7	--	17293413	Hydrolase activity, acting on carbon-nitrogen (but not peptide) bonds	Not Available
1169	Predicted inner Membrane protein	*Escherichia coli O1:K1 / APEC*		A0A0H2YXK2	--	17293413	Not Available	Not Available
1170	Hemin storage protein	*Stenotrophomonas maltophilia MF89*		T5K7H5	--	24092793	Hydrolase activity, acting on carbon-nitrogen (but not peptide) bonds	Not Available
1171	Poly-beta-1, 6-N-acetyl-D-glucosamine synthase (Poly-beta-1, 6-GlcNAc synthase) (EC 2.4.1.-)	*Stenotrophomonas maltophilia MF89*		T5KI40	--	24092793	Acetyl glucosaminyl transferase activity	Not Available
1172	Type II toxin-antitoxin system MqsR family toxin	*Herbaspirillum frisingense*		A0A7G6B4A5	--	32414048	Not Available	Not Available
1173	Putative hemin storage protein	*Stenotrophomonas maltophilia*	Strain K279a	B2FMD0	--	18419807	Hydrolase activity, acting on carbon-nitrogen (but not peptide) bonds	Not Available
1174	Poly-beta-1, 6-N-acetyl-D-glucosamine synthase (Poly-beta-1, 6-GlcNAc synthase) (EC 2.4.1.-)	*Aggregatibacter actinomycetemcomitans RhAA1*		H0KC41	--	22328766	Acetyl glucosaminyl transferase activity	Not Available
1175	Putative toxin-antitoxin system, toxin component	*Burkholderia multivorans CF2*		J4RFZ0	--	23105085	Not Available	Not Available
1176	Poly-beta-1, 6-N-acetyl-D-glucosamine biosynthesis protein PgaD	*Pseudomonas spp. WS 5406*		A0A7Y0VDH4	--	32587583	Not Available	Not Available
1177	Poly-beta-1, 6-N-acetyl-D-glucosamine synthase (Poly-beta-1, 6-GlcNAc synthase) (EC 2.4.1.-)	*Pseudomonas spp. WS 5406*		A0A7Y0VCZ0	--	32587583	Acetyl glucosaminyl transferase activity	Not Available
1178	Poly-beta-1, 6-N-acetyl-D-glucosamine synthase (Poly-beta-1, 6-GlcNAc synthase) (EC 2.4.1.-)	*Acidithiobacillales bacterium SM1_46*		A0A0S8L3M3	--	25922666	Acetyl glucosaminyl transferase activity	Not Available
1179	Poly-beta-1, 6-N-acetyl-D-glucosamine N-deacetylase PgaB (EC 3.5.1.-)	*Burkholderia multivorans CF2*		J4RKZ9	--	23105085	Hydrolase activity, acting on carbon-nitrogen (but not peptide) bonds	Not Available
1180	Putative haemin storage system, HmsS transmembrane protein	*Stenotrophomonas maltophilia*	Strain K279a	B2FMD2	--	18419807	Not Available	Not Available
1181	Uncharacterized protein	*Acidithiobacillales bacterium SM1_46*		A0A0S8L209	--	25922666	Not Available	Not Available
1182	Poly-beta-1, 6-N-acetyl-D-glucosamine synthase (Poly-beta-1, 6-GlcNAc synthase) (EC 2.4.1.-)	*Burkholderia multivorans CF2*		J4RKZ2	--	23105085	Acetyl glucosaminyl transferase activity	Not Available
1183	Uncharacterized protein	*Weissella cibaria*		A0A0D1LW17	--	25678547	Not Available	Not Available
1184	Poly-beta-1, 6-N-acetyl-D-glucosamine synthase (Poly-beta-1, 6-GlcNAc synthase) (EC 2.4.1.-)	*Stenotrophomonas maltophilia*	Strain K279a	B2FMD1	--	18419807	Acetyl glucosaminyl transferase activity	Not Available
1185	Motility quorum-sensing regulator / GCU-specific mRNA interferase toxin	*Lysobacter ruishenii*		A0A562LCQ9	--	26203337	Not Available	Not Available
1186	Uncharacterized protein	*Castellaniella defragrans 65Phen*		W8X1U6	--	24952578	Not Available	Not Available
1187	Biofilm PGA synthesis deacetylase PgaB	*Castellaniella defragrans 65Phen*		W8X5T9	--	24952578	Hydrolase activity, acting on carbon-nitrogen (but not peptide) bonds	Not Available
1188	Poly-beta-1, 6-N-acetyl-D-glucosamine biosynthesis protein PgaD	*Pseudomonas nabeulensis*		A0A4Z0ANK2	--	30910423	Not Available	Not Available

Target ID	Bacterial Protein Name	Organism Name	Strain	UniProt ID	PDB ID	PubMed ID	Classification as per UniProt GO Molecular Function	Classification of Function as per PDB
1189	Poly-beta-1, 6-N-acetyl-D-glucosamine synthase (Poly-beta-1, 6-GlcNAc synthase) (EC 2.4.1.-)	*Castellaniella defragrans 65Phen*		W8X1E8	--	24952578	Acetyl glucosaminyl transferase activity	Not Available
1190	Poly-beta-1, 6-N-acetyl-D-glucosamine synthase (Poly-beta-1, 6-GlcNAc synthase) (EC 2.4.1.-)	*Pseudomonas nabeulensis*		A0A4Z0APQ2	--	30910423	Acetyl glucosaminyl transferase activity	Not Available
1191	Uncharacterized protein	*Sulfuricurvum spp. PC08-66*		A0A0C2ZZX5	--	25620962	Not Available	Not Available
1192	Poly-beta-1, 6-N-acetyl-D-glucosamine N-deacetylase PgaB (EC 3.5.1.-)	*Pseudomonas spp. WS 5406*		A0A7Y0VCY5	--	32587583	Hydrolase activity, acting on carbon-nitrogen (but not peptide) bonds	Not Available
1193	Uncharacterized protein	*Furfurilactobacillus rossiae DSM 15814*		A0A0R1RIK6	--	26415554	Not Available	Not Available
1194	Poly-beta-1, 6-N-acetyl-D-glucosamine synthase (Poly-beta-1, 6-GlcNAc synthase) (EC 2.4.1.-)	*Methylotenera versatilis*	Strain 301	D7DJ80	--	21622745	Acetyl glucosaminyl transferase activity	Not Available
1195	GGDEF family protein	*Xanthomonas campestris pv. campestris*	Strain ATCC 33913 / DSM 3586 / NCPPB 528 / LMG 568 / P 25	Q8P4E9	--	12024217	Diguanylate cyclase activity	Not Available
1196	Poly-beta-1, 6-N-acetyl-D-glucosamine synthase (Poly-beta-1, 6-GlcNAc synthase) (EC 2.4.1.-)	*Sulfuricurvum kujiense*	Strain ATCC BAA-921 / DSM 16994 / JCM 11577 / YK-1	E4U030	--	22675602	Acetyl glucosaminyl transferase activity	Not Available
1197	Uncharacterized protein	*Methylotenera versatilis*	Strain 301	D7DK49	--	21622745	Not Available	Not Available
1198	Transcriptional regulator	*Xanthomonas campestris pv. campestris*	Strain ATCC 33913 / DSM 3586 / NCPPB 528 / LMG 568 / P 25	Q8P893	--	12024217	Diguanylate cyclase activity	Not Available
1199	GGDEF domain-containing protein	*Xanthomonas campestris pv. campestris*	Strain ATCC 33913 / DSM 3586 / NCPPB 528 / LMG 568 / P 25	Q8PAG4	--	12024217	Diguanylate cyclase activity	Not Available
1200	Response regulator	*Xanthomonas campestris pv. campestris*	Strain ATCC 33913 / DSM 3586 / NCPPB 528 / LMG 568 / P 25	Q8P782	--	12024217	Diguanylate cyclase activity	Not Available
1201	GGDEF domain-containing protein	*Xanthomonas campestris pv. campestris*	Strain ATCC 33913 / DSM 3586 / NCPPB 528 / LMG 568 / P 25	Q8P5K7	--	12024217	Diguanylate cyclase activity	Not Available
1202	Uncharacterized protein	*Xanthomonas campestris pv. campestris*	Strain ATCC 33913 / DSM 3586 / NCPPB 528 / LMG 568 / P 25	Q8PDU6	--	12024217	Diguanylate cyclase activity	Not Available
1203	Polysaccharide deacetylase	*Methylotenera versatilis*	Strain 301	D7DK51	--	21622745	Hydrolase activity, acting on carbon-nitrogen (but not peptide) bonds	Not Available
1204	GGDEF family protein	*Xanthomonas campestris pv. campestris*	Strain ATCC 33913 / DSM 3586 / NCPPB 528 / LMG 568 / P 25	Q8P9F3	--	12024217	Diguanylate cyclase activity	Not Available
1205	Motility quorum-sensing regulator MqsR	*Hyphomonaceae bacterium*		A0A3D2W0H9	--	30148503	Not Available	Not Available
1206	Poly-beta-1, 6-N-acetyl-D-glucosamine synthase (Poly-beta-1, 6-GlcNAc synthase) (EC 2.4.1.-)	*Stenotrophomonas spp.*		A0A357N2M6	--	30148503	Acetyl glucosaminyl transferase activity	Not Available
1207	Poly-beta-1, 6-N-acetyl-D-glucosamine biosynthesis protein PgaD	*Stenotrophomonas spp.*		A0A357N2K2	--	30148503	Not Available	Not Available
1208	Poly-beta-1, 6-N-acetyl-D-glucosamine N-deacetylase PgaB	*Stenotrophomonas spp.*		A0A356JDF6	--	30148503	Hydrolase activity, acting on carbon-nitrogen (but not peptide) bonds	Not Available
1209	Poly-beta-1, 6-N-acetyl-D-glucosamine N-deacetylase PgaB	*Stenotrophomonas spp.*		A0A3D0GBI1	--	30148503	Hydrolase activity, acting on carbon-nitrogen (but not peptide) bonds	Not Available
1210	Poly-beta-1, 6-N-acetyl-D-glucosamine N-deacetylase PgaB	*Stenotrophomonas spp.*		A0A357N2H1	--	30148503	Hydrolase activity, acting on carbon-nitrogen (but not peptide) bonds	Not Available
1211	Poly-beta-1, 6-N-acetyl-D-glucosamine N-deacetylase PgaB	*Stenotrophomonas spp.*		A0A3D4THG8	--	30148503	Hydrolase activity, acting on carbon-nitrogen (but not peptide) bonds	Not Available
1212	Poly-beta-1, 6-N-acetyl-D-glucosamine synthase (Poly-beta-1, 6-GlcNAc synthase) (EC 2.4.1.-)	*Stenotrophomonas spp.*		A0A3D0GBN0	--	30148503	Acetyl glucosaminyl transferase activity	Not Available
1213	Outer membrane N-deacetylase	*Escherichia coli O83:H1*	Strain NRG 857C / AIEC	A0A0H3EFY1	--	21108814	Hydrolase activity, acting on carbon-nitrogen (but not peptide) bonds	Not Available
1214	Poly-beta-1, 6-N-acetyl-D-glucosamine biosynthesis protein PgaD	*Stenotrophomonas spp.*		A0A3D4TH94	--	30148503	Not Available	Not Available
1215	Surface composition regulator	*Klebsiella pneumoniae 30660 / NJST258_1*		A0A0E1CCY4	--	24639510	Not Available	Not Available
1216	Poly-beta-1, 6-N-acetyl-D-glucosamine N-deacetylase PgaB	*Methylococcaceae bacterium*		A0A351V598	--	30148503	Hydrolase activity, acting on carbon-nitrogen (but not peptide) bonds	Not Available
1217	Biofilm PGA synthesis lipoprotein PgaB	*Acinetobacter spp. HA*		I4ZWX7	--	22933775	Hydrolase activity, acting on carbon-nitrogen (but not peptide) bonds	Not Available
1218	Hemin storage protein	*Stenotrophomonas maltophilia*	Pseudomonas maltophilia Xanthomonas maltophilia	A0A0M0P251	--	26324280	Hydrolase activity, acting on carbon-nitrogen (but not peptide) bonds	Not Available
1219	Poly-beta-1, 6-N-acetyl-D-glucosamine synthase (Poly-beta-1, 6-GlcNAc synthase) (EC 2.4.1.-)	*Stenotrophomonas maltophilia*	Pseudomonas maltophilia Xanthomonas maltophilia	A0A0M0P2A8	--	26324280	Acetyl glucosaminyl transferase activity	Not Available
1220	Poly-beta-1, 6-N-acetyl-D-glucosamine biosynthesis protein PgaD	*Stenotrophomonas maltophilia*	Pseudomonas maltophilia Xanthomonas maltophilia	A0A2J0SNZ8	--	29170656	Not Available	Not Available
1221	Uncharacterized protein	*Acinetobacter spp. HA*		I4ZWY0	--	22933775	Not Available	Not Available
1222	Poly-beta-1, 6-N-acetyl-D-glucosamine synthase (Poly-beta-1, 6-GlcNAc synthase) (EC 2.4.1.-)	*Stenotrophomonas maltophilia*	Pseudomonas maltophilia Xanthomonas maltophilia	A0A2Y9U8M9	--	29170656	Acetyl glucosaminyl transferase activity	Not Available
1223	Poly-beta-1, 6-N-acetyl-D-glucosamine N-deacetylase PgaB	*Stenotrophomonas maltophilia*	Pseudomonas maltophilia Xanthomonas maltophilia	A0A2U4HSV1	--	29170656	Hydrolase activity, acting on carbon-nitrogen (but not peptide) bonds	Not Available
1224	Poly-beta-1, 6-N-acetyl-D-glucosamine synthase (Poly-beta-1, 6-GlcNAc synthase) (EC 2.4.1.-)	*Stenotrophomonas maltophilia*	Pseudomonas maltophilia Xanthomonas maltophilia	A0A2J0UEV7	--	29170656	Acetyl glucosaminyl transferase activity	Not Available
1225	Poly-beta-1, 6-N-acetyl-D-glucosamine synthase (Poly-beta-1, 6-GlcNAc synthase) (EC 2.4.1.-)	*Stenotrophomonas maltophilia*	Pseudomonas maltophilia Xanthomonas maltophilia	A0A2J0TWK6	--	29170656	Acetyl glucosaminyl transferase activity	Not Available

Target ID	Bacterial Protein Name	Organism Name	Strain	UniProt ID	PDB ID	PubMed ID	Classification as per UniProt GO Molecular Function	Classification of Function as per PDB
1226	Poly-beta-1, 6-N-acetyl-D-glucosamine biosynthesis protein PgaD	*Stenotrophomonas maltophilia*	Pseudomonas maltophilia Xanthomonas maltophilia	A0A2J0UEW8	--	29170656	Not Available	Not Available
1227	Poly-beta-1, 6-N-acetyl-D-glucosamine N-deacetylase PgaB	*Stenotrophomonas maltophilia*	Pseudomonas maltophilia Xanthomonas maltophilia	A0A2W6M1R9	--	29170656	Hydrolase activity, acting on carbon-nitrogen (but not peptide) bonds	Not Available
1228	Poly-beta-1, 6-N-acetyl-D-glucosamine N-deacetylase PgaB	*Stenotrophomonas maltophilia*	Pseudomonas maltophilia Xanthomonas maltophilia	A0A2J0SNW7	--	29170656	Hydrolase activity, acting on carbon-nitrogen (but not peptide) bonds	Not Available
1229	Hemin storage protein	*Stenotrophomonas maltophilia*	Pseudomonas maltophilia Xanthomonas maltophilia	A0A0M0P257	--	26324280	Not Available	Not Available
1230	Surface composition regulator	*Escherichia coli O44:H18*	Strain 042 / EAEC	D3GVV9	--	20098708	Not Available	Not Available
1231	Poly-beta-1, 6-N-acetyl-D-glucosamine synthase (Poly-beta-1, 6-GlcNAc synthase) (EC 2.4.1.-)	*Acinetobacter spp. HA*		I4ZWX9	--	22933775	Acetyl glucosaminyl transferase activity	Not Available
1232	Poly-beta-1, 6-N-acetyl-D-glucosamine synthase (Poly-beta-1, 6-GlcNAc synthase) (EC 2.4.1.-)	*Stenotrophomonas maltophilia*	Pseudomonas maltophilia Xanthomonas maltophilia	A0A2J0SHH8	--	29170656	Acetyl glucosaminyl transferase activity	Not Available
1233	Poly-beta-1, 6-N-acetyl-D-glucosamine biosynthesis protein PgaD	*Thauera spp.*		A0A3B9PL39	--	30148503	Not Available	Not Available
1234	Poly-beta-1, 6-N-acetyl-D-glucosamine synthase (Poly-beta-1, 6-GlcNAc synthase) (EC 2.4.1.-)	*Pseudomonas spp. R84*		A0A7Z2KP33	--	25398872	Acetyl glucosaminyl transferase activity	Not Available
1235	Poly-beta-1, 6-N-acetyl-D-glucosamine N-deacetylase PgaB	*Desulfobacteraceae bacterium*		A0A3A4SC85	--	28644444	Hydrolase activity, acting on carbon-nitrogen (but not peptide) bonds	Not Available
1236	Poly-beta-1, 6-N-acetyl-D-glucosamine biosynthesis protein PgaD	*Pseudomonas spp. R84*		A0A7Z2KNW1	--	25398872	Not Available	Not Available
1237	Poly-beta-1, 6-N-acetyl-D-glucosamine synthase (Poly-beta-1, 6-GlcNAc synthase) (EC 2.4.1.-)	*Thauera spp.*		A0A3B9PL11	--	30148503	Acetyl glucosaminyl transferase activity	Not Available
1238	mRNA interferase MqsR	*Paraburkholderia phytofirmans OLGA172*		A0A160FSJ7	--	27063562	Not Available	Not Available
1239	Poly-beta-1, 6-N-acetyl-D-glucosamine N-deacetylase (EC 3.5.1.-)	*Acinetobacter venetianus*		A0A150HS26	--	26902269	Hydrolase activity, acting on carbon-nitrogen (but not peptide) bonds	Not Available
1240	Poly-beta-1, 6-N-acetyl-D-glucosamine N-deacetylase (EC 3.5.1.-)	*Acinetobacter venetianus*		A0A150HP82	--	26902269	Hydrolase activity, acting on carbon-nitrogen (but not peptide) bonds	Not Available
1241	Poly-beta-1, 6-N-acetyl-D-glucosamine biosynthesis protein PgaD	*Desulfobacteraceae bacterium*		A0A3A4RZP0	--	28644444	Not Available	Not Available
1242	Motility quorum-sensing regulator MqsR	*Pseudomonas spp. R84*		A0A7Z2KQT3	--	25398872	Not Available	Not Available
1243	Poly-beta-1, 6-N-acetyl-D-glucosamine N-deacetylase PgaB	*Herbaspirillum spp. YR522*		J3HWP5	--	23045501	Hydrolase activity, acting on carbon-nitrogen (but not peptide) bonds	Not Available
1244	Uncharacterized protein	*Herbaspirillum spp. YR522*		J2W5K5	--	23045501	Not Available	Not Available
1245	Uncharacterized protein	*Acinetobacter venetianus*		A0A150HP16	--	26902269	Not Available	Not Available
1246	Poly-beta-1, 6-N-acetyl-D-glucosamine synthase (Poly-beta-1, 6-GlcNAc synthase) (EC 2.4.1.-)	*Marine gamma proteobacterium HTCC2143*		A0YHE0	--	20601481	Acetyl glucosaminyl transferase activity	Not Available
1247	Poly-beta-1, 6-N-acetyl-D-glucosamine synthase (Poly-beta-1, 6-GlcNAc synthase) (EC 2.4.1.-)	*Herbaspirillum spp. YR522*		J3CKU0	--	23045501	Acetyl glucosaminyl transferase activity	Not Available
1248	Poly-beta-1, 6-N-acetyl-D-glucosamine synthase (Poly-beta-1, 6-GlcNAc synthase) (EC 2.4.1.-)	*Paraburkholderia phytofirmans OLGA172*		A0A160FI12	--	27063562	Acetyl glucosaminyl transferase activity	Not Available
1249	Poly-beta-1, 6-N-acetyl-D-glucosamine N-deacetylase	*Pseudomonas spp. R84*		A0A7Z2KNV3	--	25398872	Hydrolase activity, acting on carbon-nitrogen (but not peptide) bonds	Not Available
1250	Poly-beta-1, 6-N-acetyl-D-glucosamine N-deacetylase PgaB	*Moraxellaceae bacterium*		A0A4Q5VP81	--	30498029	Hydrolase activity, acting on carbon-nitrogen (but not peptide) bonds	Not Available
1251	Poly-beta-1, 6-N-acetyl-D-glucosamine N-deacetylase PgaB (Fragment)	*Moraxellaceae bacterium*		A0A4Q6DWC4	--	30498029	Hydrolase activity, acting on carbon-nitrogen (but not peptide) bonds	Not Available
1252	Poly-beta-1, 6-N-acetyl-D-glucosamine synthase (Poly-beta-1, 6-GlcNAc synthase) (EC 2.4.1.-)	*Moraxellaceae bacterium*		A0A4Q5VP12	--	30498029	Acetyl glucosaminyl transferase activity	Not Available
1253	Poly-beta-1, 6-N-acetyl-D-glucosamine biosynthesis protein PgaD	*Moraxellaceae bacterium*		A0A4V2AC17	--	30498029	Not Available	Not Available
1254	Outer membrane N-deacetylase	*Pseudomonas fluorescens BRIP34879*		L7HN69	--	23661484	Hydrolase activity, acting on carbon-nitrogen (but not peptide) bonds	Not Available
1255	Poly-beta-1, 6-N-acetyl-D-glucosamine synthase (Poly-beta-1, 6-GlcNAc synthase) (EC 2.4.1.-)	*Moraxellaceae bacterium*		A0A4Q5VNZ1	--	30498029	Acetyl glucosaminyl transferase activity	Not Available
1256	Xylanae / chitin deacetylase	*Acinetobacter oleivorans*	Strain JCM 16667 / KCTC 23045 / DR1	D8JGK9	--	20639327	Hydrolase activity, acting on carbon-nitrogen (but not peptide) bonds, Hydrolase activity, acting on glycosyl bonds	Not Available
1257	Biofilm PGA synthesis lipoprotein pgaB	*Acinetobacter oleivorans*	Strain JCM 16667 / KCTC 23045 / DR1	D8JE04	--	20639327	Hydrolase activity, acting on carbon-nitrogen (but not peptide) bonds	Not Available
1258	Poly-beta-1, 6-N-acetyl-D-glucosamine synthase (Poly-beta-1, 6-GlcNAc synthase) (EC 2.4.1.-)	*Acinetobacter oleivorans*	Strain JCM 16667 / KCTC 23045 / DR1	D8JE03	--	20639327	Acetyl glucosaminyl transferase activity	Not Available
1259	Biofilm PGA synthesis protein PgaD, putative	*Pseudomonas fluorescens BRIP34879*		L7HLG7	--	23661484	Not Available	Not Available
1260	Poly-beta-1, 6-N-acetyl-D-glucosamine synthase (Poly-beta-1, 6-GlcNAc synthase) (EC 2.4.1.-)	*Acinetobacter oleivorans*	Strain JCM 16667 / KCTC 23045 / DR1	D8JGL0	--	20639327	Acetyl glucosaminyl transferase activity	Not Available
1261	Poly-beta-1, 6-N-acetyl-D-glucosamine synthase (Poly-beta-1, 6-GlcNAc synthase) (EC 2.4.1.-)	*Pseudomonas fluorescens BRIP34879*		L7HM73	--	23661484	Acetyl glucosaminyl transferase activity	Not Available
1262	Uncharacterized protein	*Acinetobacter oleivorans*	Strain JCM 16667 / KCTC 23045 / DR1	D8JGL1	--	20639327	Not Available	Not Available
1263	Poly-beta-1, 6-N-acetyl-D-glucosamine synthase (Poly-beta-1, 6-GlcNAc synthase) (EC 2.4.1.-)	*Moraxellaceae bacterium*		A0A4Q6EPP1	--	30498029	Acetyl glucosaminyl transferase activity	Not Available
1264	Poly-beta-1, 6-N-acetyl-D-glucosamine biosynthesis protein PgaD	*Moraxellaceae bacterium*		A0A4Q6DYT8	--	30498029	Not Available	Not Available
1265	Poly-beta-1, 6-N-acetyl-D-glucosamine biosynthesis protein PgaD	*Moraxellaceae bacterium*		A0A4Q5VNZ4	--	30498029	Not Available	Not Available
1266	Poly-beta-1, 6-N-acetyl-D-glucosamine synthase (Poly-beta-1, 6-GlcNAc synthase) (EC 2.4.1.-) (Fragment)	*Moraxellaceae bacterium*		A0A4Q6CZ11	--	30498029	Acetyl glucosaminyl transferase activity	Not Available
1267	Polysaccharide deacetylase	*Actinobacillus succinogenes*	Strain ATCC 55618 / DSM 22257 / CCUG 43843 / 130Z	A6VMM9	--	21118570	Hydrolase activity, acting on carbon-nitrogen (but not peptide) bonds	Not Available
1268	Poly-beta-1, 6-N-acetyl-D-glucosamine synthase (Poly-beta-1, 6-GlcNAc synthase) (EC 2.4.1.-)	*Actinobacillus succinogenes*	Strain ATCC 55618 / DSM 22257 / CCUG 43843 / 130Z	A6VMN0	--	21118570	Acetyl glucosaminyl transferase activity	Not Available
1269	Putative transmembrane protein	*Ralstonia solanaarum CFBP2957*		D8P2G5	--	20550686	Not Available	Not Available

Target ID	Bacterial Protein Name	Organism Name	Strain	UniProt ID	PDB ID	PubMed ID	Classification as per UniProt GO Molecular Function	Classification of Function as per PDB
1270	Putative polysaccharide deacetylase associated with biofilm formation putative lipoprotein	*Escherichia coli O7:K1*	Strain IAI39 / ExPEC	A0A0H3MHK2	--	19165319	Hydrolase activity, acting on carbon-nitrogen (but not peptide) bonds	Not Available
1271	Motility quorum-sensing regulator	*Escherichia coli O7:K1*	Strain IAI39 / ExPEC	A0A0H3MKK3	--	19165319	Not Available	Not Available
1272	Toxin-antitoxin system, toxin component	*Burkholderia pyrrocinia*	Pseudomonas pyrrocinia	A0A0G3X296	--	26150017	Not Available	Not Available
1273	MqsR toxin	*Piscirickettsiaceae bacterium NZ-RLO1*		A0A177W0R7	--	27365345	Not Available	Not Available
1274	Motility quorum-sensing regulator mqsR	*Tistrella mobilis*	Strain KA081020-065	I3TW37	--	22458477	Not Available	Not Available
1275	Toxin-antitoxin system, toxin component	*Burkholderia pyrrocinia*	Pseudomonas pyrrocinia	A0A2Z5N2Q2	--	29899517	Not Available	Not Available
1276	Poly-beta-1, 6-N-acetyl-D-glucosamine synthase (Poly-beta-1, 6-GlcNAc synthase) (EC 2.4.1.-)	*Burkholderia pyrrocinia*	Pseudomonas pyrrocinia	A0A0G3X2J4	--	26150017	Acetyl glucosaminyl transferase activity	Not Available
1277	Poly-beta-1, 6-N-acetyl-D-glucosamine N-deacetylase PgaB	*Burkholderia pyrrocinia*	Pseudomonas pyrrocinia	A0A2Z5N2I1	--	29899517	Hydrolase activity, acting on carbon-nitrogen (but not peptide) bonds	Not Available
1278	Poly-beta-1, 6-N-acetyl-D-glucosamine synthase (Poly-beta-1, 6-GlcNAc synthase) (EC 2.4.1.-)	*Burkholderia pyrrocinia*	Pseudomonas pyrrocinia	A0A2Z5N4C5	--	29899517	Acetyl glucosaminyl transferase activity	Not Available
1279	Poly-beta-1, 6-N-acetyl-D-glucosamine synthase (Poly-beta-1, 6-GlcNAc synthase) (EC 2.4.1.-)	*Staphylococcus aureus subspp. aureus*	ST228	A0A7U7F068	--	22720005	Acetyl glucosaminyl transferase activity	Not Available
1280	Poly-beta-1, 6-N-acetyl-D-glucosamine synthase (Poly-beta-1, 6-GlcNAc synthase) (EC 2.4.1.-)	*Yersinia enterocolitica serotype O:8 / biotype 1B*	Strain NCTC 13174 / 8081	A1JS96	--	17173484	Acetyl glucosaminyl transferase activity	Not Available
1281	Uncharacterized protein	*Acinetobacter baylyi*	Strain ATCC 33305 / BD413 / ADP1	Q6FDD7	--	15514110	Not Available	Not Available
1282	Putative lipoprotein (Haemin storage system) (HmsF)	*Acinetobacter baylyi*	Strain ATCC 33305 / BD413 / ADP1	Q6FDD9	--	15514110	Hydrolase activity, acting on carbon-nitrogen (but not peptide) bonds	Not Available
1283	Poly-beta-1, 6-N-acetyl-D-glucosamine biosynthesis protein PgaD (Fragment)	*Acidiferrobacteraceae bacterium*		A0A7V2NLF0	--	31911466	Not Available	Not Available
1284	Poly-beta-1, 6-N-acetyl-D-glucosamine N-deacetylase PgaB	*Pseudomonas fluorescens*		A0A7Z8NJ86	--	31040301	Hydrolase activity, acting on carbon-nitrogen (but not peptide) bonds	Not Available
1285	Poly-beta-1, 6-N-acetyl-D-glucosamine biosynthesis protein PgaD	*Pseudomonas fluorescens*		A0A7Z8NJX9	--	31040301	Not Available	Not Available
1286	Poly-beta-1, 6-N-acetyl-D-glucosamine N-deacetylase PgaB	*Paraburkholderia azotifigens*		A0A5C6VNL0	--	29185955	Hydrolase activity, acting on carbon-nitrogen (but not peptide) bonds	Not Available
1287	Poly-beta-1, 6-N-acetyl-D-glucosamine synthase (Poly-beta-1, 6-GlcNAc synthase) (EC 2.4.1.-)	*Pseudomonas fluorescens*		A0A7Z8NJM0	--	31040301	Acetyl glucosaminyl transferase activity	Not Available
1288	Uncharacterized protein	*Gallionellales bacterium GWA2_59_43*		A0A1G0CW50	--	27774985	Not Available	Not Available
1289	Uncharacterized protein	*Pseudomonas putida*	Strain DOT-T1E	I7BZQ0	--	23815283	Not Available	Not Available
1290	Two-component system response regulator RpfG	*Xanthomonas campestris pv. vesicatoria*	Strain 85-10	Q3BUB5	--	16237009	phosphoric diester Hydrolase activity	Not Available
1291	Uncharacterized protein	*Sphingobium baderi LL03*		T0HNL1	--	24051322	Not Available	Not Available
1292	Poly-beta-1, 6-N-acetyl-D-glucosamine synthase (Poly-beta-1, 6-GlcNAc synthase) (EC 2.4.1.-)	*Pseudogulbenkiania spp.*	Strain NH8B	G2J3U5	--	22038961	Acetyl glucosaminyl transferase activity	Not Available
1293	Diguanylate cyclase	*Chloroflexus aurantiacus*	Strain ATCC 29366 / DSM 635 / J-10-fl	A9WDH0	--	21714912	Diguanylate cyclase activity	Not Available
1294	Diguanylate cyclase	*Chloroflexus aurantiacus*	Strain ATCC 29366 / DSM 635 / J-10-fl	A9WJ93	--	21714912	Diguanylate cyclase activity	Not Available
1295	Diguanylate cyclase	*Chloroflexus aurantiacus*	Strain ATCC 29366 / DSM 635 / J-10-fl	A9WIR5	--	21714912	Diguanylate cyclase activity	Not Available
1296	Polysaccharide deacetylase	*Pseudogulbenkiania spp.*	Strain NH8B	G2J3U6	--	22038961	Hydrolase activity, acting on carbon-nitrogen (but not peptide) bonds	Not Available
1297	Poly-beta-1, 6-N-acetyl-D-glucosamine biosynthesis protein PgaD	*Citrobacter freundii complex spp. CFNIH3*		A0A2I8SAW1	--	29437920	Not Available	Not Available
1298	Poly-beta-1, 6-N-acetyl-D-glucosamine synthase (Poly-beta-1, 6-GlcNAc synthase) (EC 2.4.1.-)	*Desulfotalea psychrophila*	Strain LSv54 / DSM 12343	Q6AMQ9	--	15305914	Acetyl glucosaminyl transferase activity	Not Available
1299	Poly-beta-1, 6-N-acetyl-D-glucosamine synthase (Poly-beta-1, 6-GlcNAc synthase) (EC 2.4.1.-)	*Citrobacter freundii complex spp. CFNIH3*		A0A2I8SBF6	--	29437920	Acetyl glucosaminyl transferase activity	Not Available
1300	Histidinekinase (EC 2.7.13.3)	*Bradyrhizobium diazoefficiens*	Strain JCM 10833 / BCRC 13528 / IAM 13628 / NBRC 14792 / USDA 110	Q89FJ6	--	12597275	Diguanylate cyclase activity, protein histidine kinase activity	Not Available
1301	Polysaccharide deacetylase	*Pseudogulbenkiania spp.*	Strain NH8B	G2IVP1	--	22038961	Hydrolase activity, acting on carbon-nitrogen (but not peptide) bonds	Not Available
1302	Uncharacterized protein	*Actinobacteria bacterium HGW-Actinobacteria-8*		A0A2N3F169	--	28350393	Not Available	Not Available
1303	Uncharacterized protein	*Pseudogulbenkiania spp.*	Strain NH8B	G2J3U4	--	22038961	Not Available	Not Available
1304	mRNA interferase MqsR	*Geobacter spp. OR-1*		A0A0A8WX61	--	23668621	Not Available	Not Available
1305	Surface composition regulator	*Citrobacter freundii complex spp. CFNIH3*		A0A2I8SBG3	--	29437920	Not Available	Not Available
1306	Poly-beta-1, 6-N-acetyl-D-glucosamine N-deacetylase PgaB	*Citrobacter freundii complex spp. CFNIH3*		A0A2I8SBF0	--	29437920	Hydrolase activity, acting on carbon-nitrogen (but not peptide) bonds	Not Available
1307	Diguanylate cyclase	*Chloroflexus aurantiacus*	Strain ATCC 29366 / DSM 635 / J-10-fl	A9WBR3	--	21714912	Diguanylate cyclase activity	Not Available
1308	Diguanylate cyclase	*Chloroflexus aurantiacus*	Strain ATCC 29366 / DSM 635 / J-10-fl	A9WGU1	--	21714912	Diguanylate cyclase activity	Not Available
1309	Diguanylate cyclase	*Chloroflexus aurantiacus*	Strain ATCC 29366 / DSM 635 / J-10-fl	A9WCL4	--	21714912	Diguanylate cyclase activity	Not Available
1310	Diguanylate cyclase	*Chloroflexus aurantiacus*	Strain ATCC 29366 / DSM 635 / J-10-fl	A9WKG1	--	21714912	Diguanylate cyclase activity	Not Available
1311	Diguanylate cyclase	*Chloroflexus aurantiacus*	Strain ATCC 29366 / DSM 635 / J-10-fl	A9WC49	--	21714912	Diguanylate cyclase activity	Not Available
1312	Poly-beta-1, 6-N-acetyl-D-glucosamine synthase (Poly-beta-1, 6-GlcNAc synthase) (EC 2.4.1.-)	*Stenotrophomonas spp. SAU14A_naIMI4_5*		A0A3Q8FC29	--	29765358	Acetyl glucosaminyl transferase activity	Not Available
1313	Poly-beta-1, 6-N-acetyl-D-glucosamine N-deacetylase PgaB	*Stenotrophomonas spp. SAU14A_naIMI4_5*		A0A3Q8FQ27	--	29765358	Hydrolase activity, acting on carbon-nitrogen (but not peptide) bonds	Not Available
1314	Bll7113 protein	*Bradyrhizobium diazoefficiens*	Strain JCM 10833 / BCRC 13528 / IAM 13628 / NBRC 14792 / USDA 110	Q89EH2	--	12597275	Diguanylate cyclase activity	Not Available
1315	Bll1500 protein	*Bradyrhizobium diazoefficiens*	Strain JCM 10833 / BCRC 13528 / IAM 13628 / NBRC 14792 / USDA 110	Q89UB7	--	12597275	Diguanylate cyclase activity	Not Available
1316	Poly-beta-1, 6-N-acetyl-D-glucosamine synthase (Poly-beta-1, 6-GlcNAc synthase) (EC 2.4.1.-)	*Stenotrophomonas spp. SAU14A_naIMI4_5*		A0A3S5GH39	--	29765358	Acetyl glucosaminyl transferase activity	Not Available

Target ID	Bacterial Protein Name	Organism Name	Strain	UniProt ID	PDB ID	PubMed ID	Classification as per UniProt GO Molecular Function	Classification of Function as per PDB
1317	Poly-beta-1, 6-N-acetyl-D-glucosamine synthase (Poly-beta-1, 6-GlcNAc synthase) (EC 2.4.1.-)	*Enterobacter cloacae subspp. cloacae*	Strain ATCC 13047 / DSM 30054 / NBRC 13535 / NCTC 10005 / WDCM 00083 / NCDC 279-56	A0A0H3CRE9	--	20207761	Acetyl glucosaminyl transferase activity	Not Available
1318	GGDEF domain-containing protein	*Streptomyces coelicolor*	Strain ATCC BAA-471 / A3 2 / M145	Q9ADC3	--	12000953	Diguanylate cyclase activity	Not Available
1319	Uncharacterized protein	*Polaromona naphthalenivorans*	Strain CJ2	A1VWM5	--	19453698	Not Available	Not Available
1320	Poly-beta-1, 6-N-acetyl-D-glucosamine synthase (Poly-beta-1, 6-GlcNAc synthase) (EC 2.4.1.-)	*Staphylococcus aureus*	Strain USA300	A0A0H2XJC1	--	16517273	Acetyl glucosaminyl transferase activity	Not Available
1321	Poly-beta-1, 6-N-acetyl-D-glucosamine synthase (Poly-beta-1, 6-GlcNAc synthase) (EC 2.4.1.-)	*Enterobacter ludwigii*		W0BUX8	--	25197457; 26251488	Acetyl glucosaminyl transferase activity	Not Available
1322	Type IV major pilin (Type IV pilus assembly protein PilA)	*Acidovorax citrulli*	Acidovorax avenae subspp. citrulli	C0LEX7	--	19589067	Not Available	Not Available
1323	ABC transporter substrate-binding protein (Hemin Storage System Protein) (Poly-beta-1, 6-N-acetyl-D-glucosamine biosynthesis protein PgaD)	*Enterobacter ludwigii*		G8LE15	--	22675579; 25197457	Not Available	Not Available
1324	Poly-beta-1, 6-N-acetyl-D-glucosamine biosynthesis protein PgaD	*Gammaproteobacteria bacterium*		A0A2E6LPH3	--	29337314	Not Available	Not Available
1325	Protein LpfD	*Salmonella typhimurium*	Strain LT2 / SGSC1412 / ATCC 700720	P43663	--	7721701; 11677609	Not Available	Not Available
1326	Poly-beta-1, 6-N-acetyl-D-glucosamine N-deacetylase (PGA N-deacetylase) (Poly-beta-1, 6-GlcNAc N-deacetylase) (EC 3.5.1.-)	*Escherichia coli O157:H7*		Q8XAR3	--	11206551; 11258796	Hydrolase activity, acting on carbon-nitrogen (but not peptide) bonds	Not Available
1327	Poly-beta-1, 6-N-acetyl-D-glucosamine synthase (PGA synthase) (Poly-beta-1, 6-GlcNAc synthase) (EC 2.4.1.-) (Biofilm PGA synthesis protein PgaC) (N-acetylglucosaminylTransferase PgaC)	*Escherichia coli O157:H7*		Q8XAR5	--	11206551; 11258796	Acetyl glucosaminyl transferase activity	Not Available
1328	Poly-beta-1, 6-N-acetyl-D-glucosamine synthase (PGA synthase) (Poly-beta-1, 6-GlcNAc synthase) (EC 2.4.1.-) (Biofilm PGA synthesis protein PgaC) (N-acetylglucosaminylTransferase PgaC)	*Escherichia coli*	Strain K12	P75905	--	8905232; 9278503; 16738553; 15090514; 15919996; 18359807	Acetyl glucosaminyl transferase activity	Not Available
1329	Biofilm PGA synthesis protein PgaD	*Escherichia coli O157:H7*		P69433	--	11206551; 11258796	Not Available	Not Available
1330	Biofilm PGA synthesis protein PgaD	*Escherichia coli*	Strain K12	P69432	--	8905232; 9278503; 16738553; 15090514; 15919996; 18359807; 19460094	Not Available	Not Available
1331	DNA-binding HTH domain-containing protein	*Eggerthella spp.*	Strain YY7918	F7V097	--	21914883	DNA binding	Not Available
1332	Uncharacterized protein	*Bordetella parapertussis*	Strain Bpp5	K0MGY7	--	23051057	Not Available	Not Available
1333	Sensory box / ggdef domain protein	*Thermodesulfovibrio yellowstonii*	Strain ATCC 51303 / DSM 11347 / YP87	B5YGD8	--	25635016	Diguanylate cyclase activity	Not Available
1334	Signaling protein	*Thermodesulfovibrio yellowstonii*	Strain ATCC 51303 / DSM 11347 / YP87	B5YH87	--	25635016	Diguanylate cyclase activity	Not Available
1335	Haemin storage system, HmsS protein	*Yersinia pestis bv. Antiqua*	Strain Antiqua	A0A0E1NTX1	--	16740952	Not Available	Not Available
1336	Poly-beta-1, 6-N-acetyl-D-glucosamine biosynthesis protein PgaD	*Lysobacter gummosus*		A0A0S2G4X3	--	26597042	Not Available	Not Available
1337	Uncharacterized protein	*Bordetella pertussis STO1-CHOC-0019*		A0A6N3RZF6	--	24356839	Not Available	Not Available
1338	Putative hemin storage protein	*Bordetella pertussis*	Strain ATCC 9797 / DSM 5571 / NCTC 10739 / 18323	A0A0T7CLL3	--	23051057	Hydrolase activity, acting on carbon-nitrogen (but not peptide) bonds	Not Available
1339	Poly-beta-1, 6-N-acetyl-D-glucosamine N-deacetylase PgaB (EC 3.5.1.-)	*Lysobacter gummosus*		A0A0S2G4X4	--	26597042	Hydrolase activity, acting on carbon-nitrogen (but not peptide) bonds	Not Available
1340	Uncharacterized protein	*Bordetella pertussis*	Strain ATCC 9797 / DSM 5571 / NCTC 10739 / 18323	A0A0T7CP04	--	23051057	Not Available	Not Available
1341	Ggdef domain protein	*Thermodesulfovibrio yellowstonii*	Strain ATCC 51303 / DSM 11347 / YP87	B5YJP8	--	25635016	Diguanylate cyclase activity	Not Available
1342	Uncharacterized protein	*Yersinia pestis bv. antiqua*	Strain Antiqua	A0A0E1NW62	--	16740952	Not Available	Not Available
1343	Poly-beta-1, 6-N-acetyl-D-glucosamine synthase (Poly-beta-1, 6-GlcNAc synthase) (EC 2.4.1.-)	*Bordetella pertussis*	Strain ATCC 9797 / DSM 5571 / NCTC 10739 / 18323	A0A0T7CLJ6	--	23051057	Acetyl glucosaminyl transferase activity	Not Available
1344	Surface composition regulator	*Salmonella enterica subspp. enterica serovar*	Adelaide Str. A4-669	A0A6C8GIJ5	--	21859443	Not Available	Not Available
1345	Surface composition regulator	*Salmonella schwarzengrund*	Strain CVM19633	A0A0N1QW65	--	21602358	Not Available	Not Available
1346	Uncharacterized protein	*Anaeromyxobacter spp. PSR-1*		A0A0D6QFE1	--	23709511	Not Available	Not Available
1347	Poly-beta-1, 6-N-acetyl-D-glucosamine N-deacetylase PgaB	*Shigella spp.*		A0A351P2X7	--	30148503	Hydrolase activity, acting on carbon-nitrogen (but not peptide) bonds	Not Available
1348	Poly-beta-1, 6-N-acetyl-D-glucosamine N-deacetylase PgaB	*Klebsiella pneumoniae*		A0A2L0KTV2	--	29437920	Hydrolase activity, acting on carbon-nitrogen (but not peptide) bonds	Not Available
1349	Uncharacterized protein	*Gallionellales bacterium RBG_16_56_9*		A0A1G0CZQ4	--	27774985	Not Available	Not Available
1350	Hemin storage system protein	*Enterobacter spp. DC4*		W7P486	--	24625871	Not Available	Not Available
1351	Uncharacterized protein	*Ligilactobacillus apodemi*	DSM 16634 = JCM 16172	A0A0R1TQZ9	--	26415554	Not Available	Not Available
1352	Poly-beta-1, 6-N-acetyl-D-glucosamine synthase (Poly-beta-1, 6-GlcNAc synthase) (EC 2.4.1.-)	*Enterobacter spp. DC4*		W7P4H8	--	24625871	Acetyl glucosaminyl transferase activity	Not Available
1353	Poly-beta-1, 6-N-acetyl-D-glucosamine synthase (Poly-beta-1, 6-GlcNAc synthase) (EC 2.4.1.-)	*Xanthomonas axonopodis pv. citri*	Strain 306	Q8PLI7	--	12024217	Acetyl glucosaminyl transferase activity	Not Available
1354	Poly-beta-1, 6-N-acetyl-D-glucosamine N-deacetylase (EC 3.5.1.-)	*Enterobacter spp. DC4*		W7NUM0	--	24625871	Hydrolase activity, acting on carbon-nitrogen (but not peptide) bonds	Not Available
1355	mRNA interferase MqsR	*Photorhabdus spp. S8-52*		A0A329WQK4	--	29877789	Not Available	Not Available
1356	Poly-beta-1, 6-N-acetyl-D-glucosamine synthase (Poly-beta-1, 6-GlcNAc synthase) (EC 2.4.1.-)	*Hahella chejuensis*	Strain KCTC 2396	Q2SAS4	--	16352867	Acetyl glucosaminyl transferase activity	Not Available
1357	Predicted xylanase / chitin deacetylase	*Hahella chejuensis*	Strain KCTC 2396	Q2SAS5	--	16352867	Hydrolase activity, acting on carbon-nitrogen (but not peptide) bonds, Hydrolase activity, acting on glycosyl bonds	Not Available

Target ID	Bacterial Protein Name	Organism Name	Strain	UniProt ID	PDB ID	PubMed ID	Classification as per UniProt GO Molecular Function	Classification of Function as per PDB
1358	Poly-beta-1, 6-N-acetyl-D-glucosamine N-deacetylase PgaB	*Nitrospiraceae bacterium*		A0A354A3Q8	--	30148503	Hydrolase activity, acting on carbon-nitrogen (but not peptide) bonds	Not Available
1359	Poly-beta-1, 6-N-acetyl-D-glucosamine biosynthesis protein PgaD	*Nitrospiraceae bacterium*		A0A419J381	--	28644444	Not Available	Not Available
1360	Surface composition regulator	*Salmonella gallinarum*	Strain 287 / 91 / NCTC 13346	B5REF5	--	18583645	Not Available	Not Available
1361	Poly-beta-1, 6-N-acetyl-D-glucosamine synthase (Poly-beta-1, 6-GlcNAc synthase) (EC 2.4.1.-)	*Nitrospiraceae bacterium*		A0A354A3Q7	--	30148503	Acetyl glucosaminyl transferase activity	Not Available
1362	Poly-beta-1, 6-N-acetyl-D-glucosamine N-deacetylase PgaB	*Nitrospiraceae bacterium*		A0A419J3A1	--	28644444	Hydrolase activity, acting on carbon-nitrogen (but not peptide) bonds	Not Available
1363	Poly-beta-1, 6-N-acetyl-D-glucosamine biosynthesis protein PgaD	*Nitrospiraceae bacterium*		A0A354A3Q6	--	30148503	Not Available	Not Available
1364	RNA polymerase sigma factor FliA (RNA polymerase sigma factor for flagellar operon) (Sigma F) (Sigma-28)	*Salmonella typhimurium*	Strain 14028S / SGSC 2262	A0A0F6B2T8	--	19897643	DNA binding, DNA-directed 5'-3' RNA polymerase activity, sigma factor activity	Not Available
1365	Poly-beta-1, 6-N-acetyl-D-glucosamine synthase (Poly-beta-1, 6-GlcNAc synthase) (EC 2.4.1.-)	*Nitrospiraceae bacterium*		A0A419J3A6	--	28644444	Acetyl glucosaminyl transferase activity	Not Available
1366	PgaD protein	*Escherichia coli*	Strain 55989 / EAEC	B7LFD1	--	19165319	Not Available	Not Available
1367	Hypothetical lipoprotein YcdR	*Escherichia coli O6:K15:H31*	Strain 536 / UPEC	A0A454A2Z0	--	16879640	Hydrolase activity, acting on carbon-nitrogen (but not peptide) bonds	Not Available
1368	Poly-beta-1, 6-N-acetyl-D-glucosamine synthase (Poly-beta-1, 6-GlcNAc synthase) (EC 2.4.1.-)	*Thiotrichales bacterium SG8_50*		A0A0S8EMN7	--	25922666	Acetyl glucosaminyl transferase activity	Not Available
1369	Uncharacterized protein (Fragment)	*Rhodospirillales bacterium*	RIFCSPLOWO2_12_FULL_67_15	A0A1G3IPC8	--	27774985	Not Available	Not Available
1370	Uncharacterized protein	*Escherichia coli O6:K15:H31*	Strain 536 / UPEC	A0A454A321	--	16879640	Not Available	Not Available
1371	Poly-beta-1, 6-N-acetyl-D-glucosamine synthase (Poly-beta-1, 6-GlcNAc synthase) (EC 2.4.1.-)	*Lysobacter antibioticus*		A0A0S2FCT6	--	26597042	Acetyl glucosaminyl transferase activity	Not Available
1372	Poly-beta-1, 6-N-acetyl-D-glucosamine synthase (Poly-beta-1, 6-GlcNAc synthase) (EC 2.4.1.-)	*Macrococcus bohemicus*		A0A328A4I7	--	29951040	Acetyl glucosaminyl transferase activity	Not Available
1373	Poly-beta-1, 6-N-acetyl-D-glucosamine synthase (Poly-beta-1, 6-GlcNAc synthase) (EC 2.4.1.-)	*Macrococcus caseolyticus*	Strain JCSC5402	B9EAX5	--	19074389	Acetyl glucosaminyl transferase activity	Not Available
1374	Biofilm PGA synthesis lipoprotein PgaB	*Paraburkholderia spp. BL8N3*		A0A4V2PL11	--	26203337	Hydrolase activity, acting on carbon-nitrogen (but not peptide) bonds	Not Available
1375	Poly-beta-1, 6-N-acetyl-D-glucosamine synthase (Poly-beta-1, 6-GlcNAc synthase) (EC 2.4.1.-)	*Paraburkholderia spp. BL8N3*		A0A4R1IGQ8	--	26203337	Acetyl glucosaminyl transferase activity	Not Available
1376	Surface composition regulator	*Citrobacter amalonaticus Y19*		A0A0F6RIS0	--	23377788	Not Available	Not Available
1377	Toxin-antitoxin system, toxin component	*Burkholderiales bacterium RIFOXYD12_FULL_59_19*		A0A1F4MM23	--	27774985	Not Available	Not Available
1378	Type II toxin-antitoxin system MqsR family toxin	*Agrobacterium salinitolerans*		A0A546WCU3	--	31273398	Not Available	Not Available
1379	Poly-beta-1, 6-N-acetyl-D-glucosamine biosynthesis protein PgaD	*Lysobacter antibioticus*		A0A0S2FD23	--	26597042	Not Available	Not Available
1380	Poly-beta-1, 6-N-acetyl-D-glucosamine N-deacetylase PgaB (EC 3.5.1.-)	*Lysobacter antibioticus*		A0A0S2FCY0	--	26597042	Hydrolase activity, acting on carbon-nitrogen (but not peptide) bonds	Not Available
1381	Polysaccharide deacetylase	*Xenorhabdus spp. KJ12.1*		A0A2D0LA49	--	28993611	Hydrolase activity, acting on carbon-nitrogen (but not peptide) bonds	Not Available
1382	Hemin storage protein	*Stenotrophomonas maltophilia*	Pseudomonas maltophilia Xanthomonas maltophilia	A0A0K2IYF3	--	26324280	Hydrolase activity, acting on carbon-nitrogen (but not peptide) bonds	Not Available
1383	HmsS protein	*Pseudomonas fluorescens WH6*		E2XJK0	--	20920191	Not Available	Not Available
1384	Poly-beta-1, 6-N-acetyl-D-glucosamine biosynthesis protein PgaD	*Salmonella typhimurium*		A0A708ADF7	--	30286803	Not Available	Not Available
1385	Poly-beta-1, 6-N-acetyl-D-glucosamine synthase (Poly-beta-1, 6-GlcNAc synthase) (EC 2.4.1.-)	*Pseudomonas fluorescens WH6*		E2XJJ9	--	20920191	Acetyl glucosaminyl transferase activity	Not Available
1386	Surface composition regulator	*Salmonella enterica subspp. enterica serovar*	Aberdeen	A0A5I4KB90	--	30286803	Not Available	Not Available
1387	Biofilm polysaccharide intercellular adhesin deacetylase (Intercellular adhesion protein B) (Poly-beta-1, 6-N-acetyl-D-glucosamine N-deacetylase)	*Staphylococcus haemolyticus*		B8Y3Y6	--	19144798	Hydrolase activity, acting on carbon-nitrogen (but not peptide) bonds, in linear amides	Not Available
1388	Haemin storage system, HmsS protein	*Yersinia enterocolitica*	type O:9 Str. YE56 / 03	A0A7U7FEY4	--	24753568	Not Available	Not Available
1389	Poly-beta-1, 6-N-acetyl-D-glucosamine synthase (Poly-beta-1, 6-GlcNAc synthase) (EC 2.4.1.-)	*Staphylococcus haemolyticus*		B8Y3Y4	--	19144798	Acetyl glucosaminyl transferase activity	Not Available
1390	Biofilm operon icaADBC HTH-type negative Transcriptional regulator IcaR (Intercellular adhesion protein R)	*Staphylococcus haemolyticus*		B8Y3Y3	--	19144798	DNA binding	Not Available
1391	Uncharacterized protein	*Yersinia enterocolitica*	type O:9 Str. YE56 / 03	A0A7U7FBJ4	--	24753568	Not Available	Not Available
1392	Intercellular adhesion protein C	*Staphylococcus haemolyticus*		B8Y3Y7	--	19144798	Acyltransferase activity, transferring groups other than amino-acyl groups	Not Available
1393	Polysaccharide deacetylase	*Pseudomonas fluorescens WH6*		E2XJJ8	--	20920191	Hydrolase activity, acting on carbon-nitrogen (but not peptide) bonds	Not Available
1394	Surface composition regulator	*Salmonella enterica subspp. houtenae serovar*	48:g, z51:-	A0A727TFY8	--	30286803	Not Available	Not Available
1395	Poly-beta-1, 6-N-acetyl-D-glucosamine synthase (Poly-beta-1, 6-GlcNAc synthase) (EC 2.4.1.-) (Fragment)	*Staphylococcus saprophyticus*		Q5YFS7	--	15472359	Acetyl glucosaminyl transferase activity	Not Available
1396	Biofilm polysaccharide intercellular adhesin synthesis protein IcaD (Intercellular adhesion protein D) (Poly-beta-1, 6-N-acetyl-D-glucosamine synthesis protein IcaD)	*Staphylococcus haemolyticus*		B8Y3Y5	--	19144798	Not Available	Not Available
1397	FucA	*Streptococcus gordonii*		Q6QT50	--	15205427	Alpha-L-fucosidase activity	Not Available
1398	Uncharacterized protein	*Pseudomonas fluorescens WH6*		E2XLZ9	--	20920191	Not Available	Not Available
1399	Motility quorum-sensing regulator MqsR	*Pseudomonas fluorescens Q8r1-96*		I4JYJ9	--	22792073	Not Available	Not Available
1400	Uncharacterized protein	*Acinetobacter oleivorans*	Strain JCM 16667 / KCTC 23045 / DR1	D3JTG6	--	20629796	Not Available	Not Available
1401	Putative biofilm PGA synthesis protein PgaD	*Pseudomonas fluorescens Q8r1-96*		I4L127	--	22792073	Not Available	Not Available
1402	Biofilm PGA synthesis lipoprotein PgaB (EC 3.-.-.-)	*Pseudomonas fluorescens Q8r1-96*		I4KZS7	--	22792073	Hydrolase activity, acting on carbon-nitrogen (but not peptide) bonds	Not Available
1403	Surface composition regulator	*Salmonella enterica subspp. salamae serovar 18:z10:z6*		A0A732CTX5	--	30286803	Not Available	Not Available

Target ID	Bacterial Protein Name	Organism Name	Strain	UniProt ID	PDB ID	PubMed ID	Classification as per UniProt GO Molecular Function	Classification of Function as per PDB
1404	SpsR	*Serratia plymuthica*		C4PDW9	--	21284858	DNA binding	Not Available
1405	Surface composition regulator	*Yersinia enterocolitica*	Type O:9 Str. YE56 / 03	A0A7U7IP11	--	24753568	DNA binding, DNA-directed DNA polymerase activity	Not Available
1406	Haemin storage system, HmsF protein	*Yersinia enterocolitica*	Type O:9 Str. YE56 / 03	A0A7U7FEF6	--	24753568	Hydrolase activity, acting on carbon-nitrogen (but not peptide) bonds	Not Available
1407	Surface composition regulator	*Salmonella typhimurium*		A0A3Z1VB25	--	30286803	Not Available	Not Available
1408	Poly-beta-1, 6-N-acetyl-D-glucosamine synthase (Poly-beta-1, 6-GlcNAc synthase) (EC 2.4.1.-)	*Yersinia enterocolitica*	Type O:9 Str. YE56 / 03	A0A7U7FED0	--	24753568	Acetyl glucosaminyl transferase activity	Not Available
1409	Surface composition regulator	*Salmonella typhimurium*		A0A709Z2J5	--	30286803	Not Available	Not Available
1410	Biofilm polysaccharide intercellular adhesin synthesis protein IcaD (Intercellular adhesion protein D) (Poly-beta-1, 6-N-acetyl-D-glucosamine synthesis protein IcaD)	*Staphylococcus caprae*		Q9AIQ7	--	11159959	Not Available	Not Available
1411	Surface composition regulator	*Salmonella bongori serovar*	44:r:-	A0A702BVU7	--	30286803	Not Available	Not Available
1412	Poly-beta-1, 6-N-acetyl-D-glucosamine N-deacetylase PgaB (EC 3.5.1.-)	*Salmonella typhimurium*		A0A708AH06	--	30286803	Hydrolase activity, acting on carbon-nitrogen (but not peptide) bonds	Not Available
1413	Biofilm operon icaADBC HTH-type negative Transcriptional regulator IcaR (Intercellular adhesion protein R)	*Staphylococcus caprae*		Q9AIQ9	--	11159959	DNA binding	Not Available
1414	IcaA (Fragment)	*Staphylococcus cohnii*		Q83ZI1	--	12957956	Not Available	Not Available
1415	Surface composition regulator	*Salmonella enterica subspp. salamae serovar*	58:a:-	A0A701W280	--	30286803	Not Available	Not Available
1416	Poly-beta-1, 6-N-acetyl-D-glucosamine synthase (Poly-beta-1, 6-GlcNAc synthase) (EC 2.4.1.-)	*Staphylococcus caprae*		Q9AIQ8	--	11159959	Acetyl glucosaminyl transferase activity	Not Available
1417	Outer membrane N-deacetylase	*Pseudomonas moraviensis R28-S*		V8RE08	--	24558233	Hydrolase activity, acting on carbon-nitrogen (but not peptide) bonds	Not Available
1418	Poly-beta-1, 6-N-acetyl-D-glucosamine synthase (Poly-beta-1, 6-GlcNAc synthase) (EC 2.4.1.-)	*Salmonella typhimurium*		A0A708A923	--	30286803	Acetyl glucosaminyl transferase activity	Not Available
1419	Poly-beta-1, 6-N-acetyl-D-glucosamine synthase (Poly-beta-1, 6-GlcNAc synthase) (EC 2.4.1.-)	*Aggregatibacter actinomycetemcomitans*	Actinobacillus actinomycetemcomitans Haemophilus actinomycetemcomitans	Q5VJB2	--	15576769	Acetyl glucosaminyl transferase activity	Not Available
1420	Poly-beta-1, 6-N-acetyl-D-glucosamine synthase (Poly-beta-1, 6-GlcNAc synthase) (EC 2.4.1.-)	*Actinobacillus pleuropneumoniae*	Haemophilus pleuropneumoniae	Q5QFG3	--	15576769	Acetyl glucosaminyl transferase activity	Not Available
1421	Poly-beta-1, 6-N-acetyl-D-glucosamine synthase (Poly-beta-1, 6-GlcNAc synthase) (EC 2.4.1.-)	*Aggregatibacter actinomycetemcomitans*	Actinobacillus actinomycetemcomitans Haemophilus actinomycetemcomitans	A5HJW9	--	17851029	Acetyl glucosaminyl transferase activity	Not Available
1422	Biofilm polysaccharide intercellular adhesin deacetylase (Intercellular adhesion protein B) (Poly-beta-1, 6-N-acetyl-D-glucosamine N-deacetylase)	*Staphylococcus caprae*		Q9AIQ6	--	11159959	Hydrolase activity, acting on carbon-nitrogen (but not peptide) bonds, in linear amides	Not Available
1423	Toxin CdiA	*Neisseria meningitidis*		C0KXJ0	--	19289515	Toxin activity	Not Available
1424	Acyl-homoserine-lactone synthase (EC 2.3.1.184) (Autoinducer synthesis protein)	*Ensifer adhaerens*	Sinorhizobium morelense	K4K1V0	--	23090571	N-acyl homoserine lactone synthase activity	Not Available
1425	Putative membrane protein	*Bordetella bronchiseptica*	Strain ATCC BAA-588 / NCTC 13252 / RB50 AlcaligeneS bronchiSepticuS	A0A0H3LTS4	--	12910271	Not Available	Not Available
1426	Toxin CdiA	*Neisseria meningitidis*		C0KXI6	--	19289515	toxin activity	Not Available
1427	Toxin CdiA	*Neisseria meningitidis*		C0KXI4	--	19289515	toxin activity	Not Available
1428	Uncharacterized protein	*Cupriavidus oxalaticus*		Q84ES5	--	12902278	Not Available	Not Available
1429	Surface composition regulator	*Salmonella enterica subspp. enterica serovar*	Oslo	A0A5I0WQX2	--	30286803	Not Available	Not Available
1430	Acyl-homoserine-lactone synthase (EC 2.3.1.184) (Autoinducer synthesis protein)	*Ensifer adhaerens*	Sinorhizobium morelense	K4KF64	--	23090571	N-acyl homoserine lactone synthase activity	Not Available
1431	Uncharacterized protein	*Cupriavidus necator*	Alcaligenes eutrophus Ralstonia eutropha	Q9ZAL5	--	9887305	Not Available	Not Available
1432	Acyl-homoserine-lactone synthase (EC 2.3.1.184) (Autoinducer synthesis protein)	*Ensifer adhaerens*	Sinorhizobium morelense	K4K584	--	23090571	N-acyl homoserine lactone synthase activity	Not Available
1433	Surface composition regulator	*Salmonella enterica*	Salmonella choleraesuis	A0A750KGM0	--	30286803	Not Available	Not Available
1434	Surface composition regulator	*Salmonella enterica*	Salmonella choleraesuis	A0A753AFU1	--	30286803	Not Available	Not Available
1435	Metal-binding permease	*Streptococcus gordonii*	Str. Challis	Q842E9	--	12700268	zinc ion binding	Not Available
1436	Poly-beta-1, 6-N-acetyl-D-glucosamine synthase (Poly-beta-1, 6-GlcNAc synthase) (EC 2.4.1.-)	*Bordetella bronchiseptica*	Strain ATCC BAA-588 / NCTC 13252 / RB50 Alcaligenes bronchisepticus	A0A0H3LKJ5	--	12910271	Acetyl glucosaminyl transferase activity	Not Available
1437	Surface composition regulator	*Salmonella enterica*	Salmonella choleraesuis	A0A702CJE0	--	30286803	Not Available	Not Available
1438	Surface composition regulator	*Salmonella moscow*		A0A5H7LB63	--	30286803	Not Available	Not Available
1439	Surface composition regulator	*Salmonella typhimurium*		A0A735I5S7	--	30286803	Not Available	Not Available
1440	Surface composition regulator	*Salmonella newport*		A0A3V7UC64	--	30286803	Not Available	Not Available
1441	Surface composition regulator	*Escherichia coli*		C3SU77	--	19439656	Not Available	Not Available
1442	Poly-beta-1, 6-N-acetyl-D-glucosamine synthase (Poly-beta-1, 6-GlcNAc synthase) (EC 2.4.1.-)	*Escherichia coli*		C3TEV2	--	19439656	Acetyl glucosaminyl transferase activity	Not Available
1443	MrkA	*Escherichia coli*		B0LHG5	--	18055599; 19717626	Not Available	Not Available
1444	Surface composition regulator	*Salmonella enterica subspp. enterica serovar*	Corvallis	A0A4U7YCJ9	--	30286803	Not Available	Not Available
1445	Surface composition regulator	*Salmonella enterica subspp. enterica serovar*	Rissen	A0A5H7QG39	--	30286803	Not Available	Not Available
1446	IcaA (Fragment)	*Staphylococcus saprophyticus*		Q83TU9	--	12957956	Not Available	Not Available
1447	Surface composition regulator	*Salmonella typhi*		A0A4U8GBI9	--	30286803	Not Available	Not Available
1448	Acyl-homoserine-lactone synthase (EC 2.3.1.184) (Autoinducer synthesis protein)	*Pseudomonas putida*	Arthrobacter siderocapsulatus	Q8GEM0	--	12450862; 15345437; 16585751	N-acyl homoserine lactone synthase activity	Not Available
1449	Surface composition regulator	*Salmonella enterica*	Salmonella choleraesuis	A0A750MQQ2	--	30286803	Not Available	Not Available
1450	Surface composition regulator	*Salmonella abortus-equi*		A0A738E8L1	--	30286803	Not Available	Not Available
1451	Surface composition regulator	*Salmonella enterica subspp. enterica serovar*	Brandenburg	A0A5I9JE21	--	30286803	Not Available	Not Available
1452	MrkD	*Escherichia coli*		B0LHG8	--	18055599; 19717626	Not Available	Not Available
1453	Surface composition regulator	*Salmonella enterica subspp. enterica serovar*	Paratyphi C	A0A6Y5YMH6	--	30286803	Not Available	Not Available
1454	MrkC	*Escherichia coli*		B0LHG7	--	18055599; 19717626	Fimbrial usher porin activity	Not Available
1455	SLATT_6 domain-containing protein	*Escherichia coli*		B0LHG1	--	18055599; 19717626	Not Available	Not Available

Target ID	Bacterial Protein Name	Organism Name	Strain	UniProt ID	PDB ID	PubMed ID	Classification as per UniProt GO Molecular Function	Classification of Function as per PDB
1456	Surface composition regulator	*Salmonella typhimurium*	Strain SL1344	A0A718QE43	--	30286803	Not Available	Not Available
1457	AGS_C domain-containing protein	*Escherichia coli*		B0LHG2	--	18055599; 19717626	Nucleotidyltransferase activity	Not Available
1458	Poly-beta-1, 6-N-acetyl-D-glucosamine synthase (Poly-beta-1, 6-GlcNAc synthase) (EC 2.4.1.-)	*Staphylococcus capitis*		G4Y1I6	--	23052315	Acetyl glucosaminyl transferase activity	Not Available
1459	Surface composition regulator	*Salmonella enterica subspp. enterica serovar*	Choleraesuis	A0A5I8Q4H1	--	30286803	Not Available	Not Available
1460	AagD (PgaD)	*Aggregatibacter actinomycetemcomitans*	Actinobacillus actinomycetemcomitans Haemophilus actinomycetemcomitans	Q5VJB1	--	15576769; 17851029	Not Available	Not Available
1461	Surface composition regulator	*Salmonella enterica subspp. salamae serovar 48:d:z6*		A0A729JLQ4	--	30286803	Not Available	Not Available
1462	Acyl-homoserine-lactone synthase (EC 2.3.1.184) (Autoinducer synthesis protein)	*Edwardsiella tarda*		Q60HM1	--	16233705; 19283379	N-acyl homoserine lactone synthase activity	Not Available
1463	Surface composition regulator	*Salmonella enterica subspp. enterica serovar*	Alachua	A0A5I4B3X4	--	30286803	Not Available	Not Available
1464	Surface composition regulator	*Salmonella enterica subspp. enterica serovar*	Altona	A0A3V4SJ84	--	30286803	Not Available	Not Available
1465	Surface composition regulator	*Salmonella enterica subspp. enterica serovar*	Agama	A0A3V8VN13	--	30286803	Not Available	Not Available
1466	Surface composition regulator	*Salmonella enterica subspp. salamae serovar*	47:b:1, 5	A0A735M8M7	--	30286803	Not Available	Not Available
1467	Surface composition regulator	*Salmonella enterica subspp. houtenae serovar*	44:z36[z38]:-	A0A737M2X2	--	30286803	Not Available	Not Available
1468	Surface composition regulator	*Salmonella enterica subspp. enterica serovar*	Rough O:-:-	A0A730TM30	--	30286803	Not Available	Not Available
1469	Surface composition regulator	*Salmonella enteritidis PT4*	Strain P125109	A0A725G1U5	--	30286803	Not Available	Not Available
1470	Surface composition regulator	*Salmonella enterica subspp. salamae serovar*	58:d:z6	A0A737SLZ3	--	30286803	Not Available	Not Available
1471	Poly-beta-1, 6-N-acetyl-D-glucosamine synthase (Poly-beta-1, 6-GlcNAc synthase) (EC 2.4.1.-)	*Yersinia pestis*		Q56941	--	9249062	Acetyl glucosaminyl transferase activity	Not Available
1472	Surface composition regulator	*Salmonella enterica*	Salmonella choleraesuis	A0A742XT71	--	30286803	Not Available	Not Available
1473	RNA polymerase sigma factor (Fragment)	*Edwardsiella tarda*		C3U1E8	--	19283379	DNA binding, sigma factor activity	Not Available
1474	HmsF	*Yersinia pestis*		Q56940	--	9249062	Hydrolase activity, acting on carbon-nitrogen (but not peptide) bonds	Not Available
1475	IcaA (Fragment)	*Mammaliicoccus sciuri*	Staphylococcus sciuri	Q83ZI2	--	12957956	Not Available	Not Available
1476	Poly-beta-1, 6-N-acetyl-D-glucosamine synthase (Poly-beta-1, 6-GlcNAc synthase) (EC 2.4.1.-)	*Xenorhabdus nematophila*	Achromobacter nematophilus	B3GN23	--	18515487	Acetyl glucosaminyl transferase activity	Not Available
1477	MacB	*Pseudomonas putida*	Arthrobacter siderocapsulatus	A8MN40	--	16585751; 18599835	ATP binding	Not Available
1478	SuhB (Fragment)	*Pseudomonas putida*	Arthrobacter siderocapsulatus	Q3Y8H9	--	16585751	Not Available	Not Available
1479	PpuR	*Pseudomonas putida*	Arthrobacter siderocapsulatus	Q3Y8H6	--	16585751	DNA binding	Not Available
1480	Surface composition regulator	*Salmonella enterica subspp. enterica serovar*	Ank	A0A5I2X3G4	--	30286803	Not Available	Not Available
1481	OprM	*Pseudomonas putida*	Arthrobacter siderocapsulatus	A8MN35	--	16585751; 18599835	Efflux transmembrane transporter activity	Not Available
1482	Putative polysaccharide deacetylase associated with biofilm formation putative lipoprotein (PgaB)	*Ralstonia syzygii R24*		G3A8J5	--	21931687	Hydrolase activity, acting on carbon-nitrogen (but not peptide) bonds	Not Available
1483	Poly-beta-1, 6-N-acetyl-D-glucosamine biosynthesis protein PgaD	*Shigella spp.*		A0A352GZL1	--	30148503	Not Available	Not Available
1484	Surface composition regulator	*Shigella spp.*		A0A358UM46	--	30148503	Not Available	Not Available
1485	Poly-beta-1, 6-N-acetyl-D-glucosamine biosynthesis protein PgaD	*Klebsiella pneumoniae*		A0A2L0KL40	--	29437920	Not Available	Not Available
1486	HmsS protein	*Klebsiella pneumoniae*		W9BJP9	--	25341126	Not Available	Not Available
1487	Poly-beta-1, 6-N-acetyl-D-glucosamine N-deacetylase	*Anaeromyxobacter spp. PSR-1*		A0A0D6QE29	--	23709511	Hydrolase activity, acting on carbon-nitrogen (but not peptide) bonds	Not Available
1488	Poly-beta-1, 6-N-acetyl-D-glucosamine biosynthesis protein PgaD	*Acinetobacter seifertii*		N8S8L3	--	25563912	Not Available	Not Available
1489	Poly-beta-1, 6-N-acetyl-D-glucosamine N-deacetylase PgaB	*Metakosakonia spp. MRY16-398*		A0A348DF60	--	30524415	Hydrolase activity, acting on carbon-nitrogen (but not peptide) bonds	Not Available
1490	Poly-beta-1, 6-N-acetyl-D-glucosamine biosynthesis protein PgaD	*Metakosakonia spp. MRY16-398*		A0A348DF58	--	30524415	Not Available	Not Available
1491	Poly-beta-1, 6-N-acetyl-D-glucosamine N-deacetylase PgaB	*Gammaproteobacteria bacterium*		A0A7C5JBZ9	--	31911466	Hydrolase activity, acting on carbon-nitrogen (but not peptide) bonds	Not Available
1492	Surface composition regulator	*Metakosakonia spp. MRY16-398*		A0A348DK62	--	30524415	Not Available	Not Available
1493	Poly-beta-1, 6-N-acetyl-D-glucosamine biosynthesis protein PgaD	*Gammaproteobacteria bacterium*		A0A3D1RX59	--	30148503	Not Available	Not Available
1494	Uncharacterized protein	*Gammaproteobacteria bacterium*		A0A3C0I6L8	--	30148503	Not Available	Not Available
1495	Poly-beta-1, 6-N-acetyl-D-glucosamine synthase (Poly-beta-1, 6-GlcNAc synthase) (EC 2.4.1.-)	*Gammaproteobacteria bacterium*		A0A3D1RSI1	--	30148503	Acetyl glucosaminyl transferase activity	Not Available
1496	TonB_C domain-containing protein	*Lysobacter enzymogenes*		A0A1J1EBZ8	--	28065880	Not Available	Not Available
1497	Motility quorum-sensing regulator MqsR	*Deltaproteobacteria bacterium RIFCSPLOWO2_02_FULL_53_8*		A0A1F9IJA9	--	27774985	Not Available	Not Available
1498	Poly-beta-1, 6-N-acetyl-D-glucosamine synthase (Poly-beta-1, 6-GlcNAc synthase) (EC 2.4.1.-)	*Lentisphaerae bacterium GWF2_50_93*		A0A1G0ZQ85	--	27774985	Acetyl glucosaminyl transferase activity	Not Available
1499	Uncharacterized protein	*Deltaproteobacteria bacterium RIFCSPLOWO2_02_FULL_53_8*		A0A1F9IY36	--	27774985	Not Available	Not Available
1500	Biofilm formation regulator HmsP	*Enterobacter agglomerans*	Erwinia herbicola Pantoea agglomerans	A0A8I0LKD8	--	32966572	Not Available	Not Available
1501	Poly-beta-1, 6-N-acetyl-D-glucosamine biosynthesis protein PgaD	*Lentisphaerae bacterium GWF2_50_93*		A0A1G0ZS47	--	27774985	Not Available	Not Available
1502	Poly-beta-1, 6-N-acetyl-D-glucosamine N-deacetylase PgaB	*Lentisphaerae bacterium GWF2_50_93*		A0A1G0ZQG0	--	27774985	Hydrolase activity, acting on carbon-nitrogen (but not peptide) bonds	Not Available
1503	Poly-beta-1, 6-N-acetyl-D-glucosamine synthase (Poly-beta-1, 6-GlcNAc synthase) (EC 2.4.1.-)	*Achromobacter xylosoxidans*	Strain A8	E3HKY0	--	21097610	Acetyl glucosaminyl transferase activity	Not Available
1504	Poly-beta-1, 6-N-acetyl-D-glucosamine synthase (Poly-beta-1, 6-GlcNAc synthase) (EC 2.4.1.-)	*Carnobacterium divergens*	Lactobacillus divergens	A0A5F0MFX7	--	30081251	Acetyl glucosaminyl transferase activity	Not Available
1505	Biofilm PGA synthesis lipoprotein PgaB (EC 3.-.-.-)	*Achromobacter xylosoxidans*	Strain A8	E3HKY1	--	21097610	Hydrolase activity, acting on carbon-nitrogen (but not peptide) bonds	Not Available

Target ID	Bacterial Protein Name	Organism Name	Strain	UniProt ID	PDB ID	PubMed ID	Classification as per UniProt GO Molecular Function	Classification of Function as per PDB
1506	Poly-beta-1, 6-N-acetyl-D-glucosamine synthase (Poly-beta-1, 6-GlcNAc synthase) (EC 2.4.1.-)	*Pseudomonas moraviensis R28-S*		V8RF67	--	24558233	Acetyl glucosaminyl transferase activity	Not Available
1507	Uncharacterized protein	*Caldanaerobacter subterraneus subspp. yonseiensis KB-1*		U5CWT9	--	24201201	Not Available	Not Available
1508	Poly-beta-1, 6-N-acetyl-D-glucosamine synthase (Poly-beta-1, 6-GlcNAc synthase) (EC 2.4.1.-)	*Kangiella koreensis*	Strain DSM 16069 / KCTC 12182 / SW-125	C7RBF1	--	21304661	Acetyl glucosaminyl transferase activity	Not Available
1509	Poly-beta-1, 6-N-acetyl-D-glucosamine synthase (Poly-beta-1, 6-GlcNAc synthase) (EC 2.4.1.-)	*Alicyclobacillus acidocaldarius subspp. acidocaldarius*	Strain ATCC 27009 / DSM 446 / BCRC 14685 / JCM 5260 / KCTC 1825 / NBRC 15652 / NCIMB 11725 / NRRL B-14509 / 104-IA Bacillus acidocaldarius	C8WT25	--	21304673	Acetyl glucosaminyl transferase activity	Not Available
1510	Poly-beta-1, 6-N-acetyl-D-glucosamine synthase (Poly-beta-1, 6-GlcNAc synthase) (EC 2.4.1.-)	*Escherichia coli O157:H7*	Strain EC869	A0A0H3PPW9	--	21421787	Acetyl glucosaminyl transferase activity	Not Available
1511	Poly-beta-1, 6-N-acetyl-D-glucosamine N-deacetylase PgaB	*Stenotrophomonas spp. ESTM1D_MKCIP4_1*		A0A3S7KT60	--	29765358	Hydrolase activity, acting on carbon-nitrogen (but not peptide) bonds	Not Available
1512	Poly-beta-1, 6-N-acetyl-D-glucosamine synthase (Poly-beta-1, 6-GlcNAc synthase) (EC 2.4.1.-)	*Comamonae bacterium*		A0A4Q3R2D4	--	30498029	Acetyl glucosaminyl transferase activity	Not Available
1513	Poly-beta-1, 6-N-acetyl-D-glucosamine biosynthesis protein PgaD	*Comamonae bacterium*		A0A4Q3QYG0	--	30498029	Not Available	Not Available
1514	Lipoprotein for biofilm formation	*Bacillus subtilis XF-1*		M4KUX5	--	23558530	Not Available	Not Available
1515	Poly-beta-1, 6-N-acetyl-D-glucosamine synthase (Poly-beta-1, 6-GlcNAc synthase) (EC 2.4.1.-)	*Pectobacterium atrosepticum*	Strain SCRI 1043 / ATCC BAA-672 Erwinia carotovora subspp. atroseptica	Q6CYQ3	--	15263089	Acetyl glucosaminyl transferase activity	Not Available
1516	Surface composition regulator	*Pectobacterium atrosepticum*	Strain SCRI 1043 / ATCC BAA-672 Erwinia carotovora subspp. atroseptica	Q6D4M7	--	15263089	DNA binding, DNA-directed DNA polymerase activity	Not Available
1517	Putative hemin storage protein	*Pectobacterium atrosepticum*	Strain SCRI 1043 / ATCC BAA-672 Erwinia carotovora subspp. atroseptica	Q6CYQ4	--	15263089	Not Available	Not Available
1518	Poly-beta-1, 6-N-acetyl-D-glucosamine N-deacetylase PgaB	*Comamonae bacterium*		A0A4Q3R554	--	30498029	Hydrolase activity, acting on carbon-nitrogen (but not peptide) bonds	Not Available
1519	Poly-beta-1, 6-N-acetyl-D-glucosamine biosynthesis protein PgaD	*Comamonae bacterium*		A0A4Q3PH24	--	30498029	Not Available	Not Available
1520	Poly-beta-1, 6-N-acetyl-D-glucosamine biosynthesis protein PgaD	*Comamonae bacterium*		A0A4Q3R2C1	--	30498029	Not Available	Not Available
1521	Uncharacterized protein	*Leptospira licerasiae serovar Varillal*	Str. VAR 10	I0XRG0	--	23145189	Not Available	Not Available
1522	Uncharacterized protein	*Stenotrophomonas rhizophila*		A0A4U3GLQ3	--	31040301	Not Available	Not Available
1523	Poly-beta-1, 6-N-acetyl-D-glucosamine synthase (Poly-beta-1, 6-GlcNAc synthase) (EC 2.4.1.-)	*Stenotrophomonas rhizophila*		A0A4U3GSE5	--	31040301	Acetyl glucosaminyl transferase activity	Not Available
1524	Uncharacterized protein	*Pseudomonas brassicacearum*	Strain NFM421	F2KL25	--	21515771	Not Available	Not Available
1525	Poly-beta-1, 6-N-acetyl-D-glucosamine N-deacetylase PgaB	*Stenotrophomonas rhizophila*		A0A4U3GQQ3	--	31040301	Hydrolase activity, acting on carbon-nitrogen (but not peptide) bonds	Not Available
1526	Putative biofilm PNot AvailableG synthesis protein	*Pseudomonas brassicacearum*	Strain NFM421	F2KD34	--	21515771	Not Available	Not Available
1527	Poly-beta-1, 6-N-acetyl-D-glucosamine biosynthesis protein PgaD	*Stenotrophomonas rhizophila*		A0A4V5UEL4	--	31040301	Not Available	Not Available
1528	Uncharacterized protein	*Deltaproteobacteria bacterium RBG_16_58_17*		A0A1F9CH81	--	27774985	Not Available	Not Available
1529	Poly-beta-1, 6-N-acetyl-D-glucosamine synthase (Poly-beta-1, 6-GlcNAc synthase) (EC 2.4.1.-)	*Comamonadaceae bacterium*		A0A4Q3RBI5	--	30498029	Acetyl glucosaminyl transferase activity	Not Available
1530	Poly-beta-1, 6-N-acetyl-D-glucosamine N-deacetylase PgaB (Fragment)	*Comamonadaceae bacterium*		A0A4V2ABY7	--	30498029	Hydrolase activity, acting on carbon-nitrogen (but not peptide) bonds	Not Available
1531	Poly-beta-1, 6-N-acetyl-D-glucosamine biosynthesis protein PgaD	*Proteobacteria bacterium ST_bin11*		A0A1W9K6T1	--	28390307	Not Available	Not Available
1532	Putative hemin storage lipoprotein	*Pectobacterium atrosepticum*	Strain SCRI 1043 / ATCC BAA-672 Erwinia carotovora subspp. atroseptica	Q6CYQ2	--	15263089	Hydrolase activity, acting on carbon-nitrogen (but not peptide) bonds	Not Available
1533	Poly-beta-1, 6-N-acetyl-D-glucosamine N-deacetylase PgaB	*Proteobacteria bacterium ST_bin11*		A0A1W9K6R2	--	28390307	Hydrolase activity, acting on carbon-nitrogen (but not peptide) bonds	Not Available
1534	Poly-beta-1, 6-N-acetyl-D-glucosamine N-deacetylase PgaB	*Acinetobacter spp.*		A0A519JFC4	--	30498029	Hydrolase activity, acting on carbon-nitrogen (but not peptide) bonds	Not Available
1535	Poly-beta-1, 6-N-acetyl-D-glucosamine synthase (Poly-beta-1, 6-GlcNAc synthase) (EC 2.4.1.-)	*Acinetobacter spp.*		A0A519JFC8	--	30498029	Acetyl glucosaminyl transferase activity	Not Available
1536	Poly-beta-1, 6-N-acetyl-D-glucosamine synthase (Poly-beta-1, 6-GlcNAc synthase) (EC 2.4.1.-)	*Klebsiella aerogenes*	Strain ATCC 13048 / DSM 30053 / JCM 1235 / KCTC 2190 / NBRC 13534 / NCIMB 10102 / NCTC 10006 Enterobacter aerogenes	A0A0H3FV53	--	22493190	Acetyl glucosaminyl transferase activity	Not Available
1537	Poly-beta-1, 6-N-acetyl-D-glucosamine biosynthesis protein PgaD	*Acinetobacter spp.*		A0A519JGP9	--	30498029	Not Available	Not Available
1538	Poly-beta-1, 6-N-acetyl-D-glucosamine synthase (Poly-beta-1, 6-GlcNAc synthase) (EC 2.4.1.-)	*Acinetobacter spp.*		A0A519JGN0	--	30498029	Acetyl glucosaminyl transferase activity	Not Available
1539	Hemin storage system protein	*Klebsiella aerogenes*	Strain ATCC 13048 / DSM 30053 / JCM 1235 / KCTC 2190 / NBRC 13534 / NCIMB 10102 / NCTC 10006 Enterobacter aerogenes	A0A0H3FMQ6	--	22493190	Not Available	Not Available
1540	Poly-beta-1, 6-N-acetyl-D-glucosamine synthase (Poly-beta-1, 6-GlcNAc synthase) (EC 2.4.1.-)	*Comamonae bacterium*		A0A4Q3QVP0	--	30498029	Acetyl glucosaminyl transferase activity	Not Available
1541	Poly-beta-1, 6-N-acetyl-D-glucosamine N-deacetylase PgaB	*Acinetobacter spp.*		A0A519JFC5	--	30498029	Hydrolase activity, acting on carbon-nitrogen (but not peptide) bonds	Not Available
1542	Poly-beta-1, 6-N-acetyl-D-glucosamine biosynthesis protein PgaD	*Comamonae bacterium*		A0A4Q3Q3K8	--	30498029	Not Available	Not Available

Target ID	Bacterial Protein Name	Organism Name	Strain	UniProt ID	PDB ID	PubMed ID	Classification as per UniProt GO Molecular Function	Classification of Function as per PDB
1543	Poly-beta-1, 6-N-acetyl-D-glucosamine biosynthesis protein PgaD	*Acinetobacter spp.*		A0A519JFE0	--	30498029	Not Available	Not Available
1544	Poly-beta-1, 6-N-acetyl-D-glucosamine synthase (Poly-beta-1, 6-GlcNAc synthase) (EC 2.4.1.-)	*Proteobacteria bacterium ST_bin11*		A0A1W9K6Q3	--	28390307	Acetyl glucosaminyl transferase activity	Not Available
1545	Poly-beta-1, 6-N-acetyl-D-glucosamine N-deacetylase PgaB (Fragment)	*Comamonae bacterium*		A0A4Q3NPB9	--	30498029	Hydrolase activity, acting on carbon-nitrogen (but not peptide) bonds	Not Available
1546	Poly-beta-1, 6-N-acetyl-D-glucosamine N-deacetylase PgaB (EC 3.5.1.-)	*Pseudomonas spp. BIOMIG1BAC*		A0A6G6ZRV1	--	26992451	Hydrolase activity, acting on carbon-nitrogen (but not peptide) bonds	Not Available
1547	Surface composition regulator	*Klebsiella aerogenes*	Strain ATCC 13048 / DSM 30053 / JCM 1235 / KCTC 2190 / NBRC 13534 / NCIMB 10102 / NCTC 10006 Enterobacter aerogenes	A0A0H3FS42	--	22493190	Not Available	Not Available
1548	Type II toxin-antitoxin system MqsR family toxin	*Comamonae bacterium*		A0A4Q3QZB8	--	30498029	Not Available	Not Available
1549	Poly-beta-1, 6-N-acetyl-D-glucosamine N-deacetylase PgaB	*Acinetobacter spp.*		A0A519JGN1	--	30498029	Hydrolase activity, acting on carbon-nitrogen (but not peptide) bonds	Not Available
1550	Poly-beta-1, 6-N-acetyl-D-glucosamine biosynthesis protein PgaD	*Comamonadaceae bacterium*		A0A4V1UAI0	--	30498029	Not Available	Not Available
1551	Lipoprotein for biofilm formation	*Bacillus spp. WP8*		A0A0B4S988	--	25614565	Not Available	Not Available
1552	Poly-beta-1, 6-N-acetyl-D-glucosamine N-deacetylase PgaB	*Comamonadaceae bacterium*		A0A4Q3RD47	--	30498029	Hydrolase activity, acting on carbon-nitrogen (but not peptide) bonds	Not Available
1553	Poly-beta-1, 6-N-acetyl-D-glucosamine biosynthesis protein PgaD	*Comamonadaceae bacterium*		A0A4Q3PKV1	--	30498029	Not Available	Not Available
1554	Poly-beta-1, 6-N-acetyl-D-glucosamine biosynthesis protein PgaD	*Pseudomonas spp. BIOMIG1BAC*		A0A6G6ZS19	--	26992451	Not Available	Not Available
1555	Poly-beta-1, 6-N-acetyl-D-glucosamine N-deacetylase PgaB (Fragment)	*Comamonadaceae bacterium*		A0A4Q5X007	--	30498029	Hydrolase activity, acting on carbon-nitrogen (but not peptide) bonds	Not Available
1556	Putative biofilm PNot AvailableG synthesis protein	*Pseudomonas brassicacearum*	Strain NFM421	F2KD32	--	21515771	Hydrolase activity, acting on carbon-nitrogen (but not peptide) bonds	Not Available
1557	Poly-beta-1, 6-N-acetyl-D-glucosamine N-deacetylase PgaB	*Pseudomonas viciae*		A0A4P7PAA8	--	30941113	Hydrolase activity, acting on carbon-nitrogen (but not peptide) bonds	Not Available
1558	Poly-beta-1, 6-N-acetyl-D-glucosamine N-deacetylase PgaB	*Acinetobacter pittii*	Acinetobacter genomospp. 3	A0A6H2VLN2	--	30036696	Hydrolase activity, acting on carbon-nitrogen (but not peptide) bonds	Not Available
1559	Polysaccharide deacetylase	*Burkholderia cenocepacia*	Strain ATCC BAA-245 / DSM 16553 / LMG 16656 / NCTC 13227 / J2315 / CF5610 Burkholderia cepacia Strain J2315	B4EHA7	--	18931103	Hydrolase activity, acting on carbon-nitrogen (but not peptide) bonds	Not Available
1560	Poly-beta-1, 6-N-acetyl-D-glucosamine synthase (Poly-beta-1, 6-GlcNAc synthase) (EC 2.4.1.-)	*Burkholderia cenocepacia*	Strain ATCC BAA-245 / DSM 16553 / LMG 16656 / NCTC 13227 / J2315 / CF5610 Burkholderia cepacia Strain J2315	B4EHA6	--	18931103	Acetyl glucosaminyl transferase activity	Not Available
1561	Poly-beta-1, 6-N-acetyl-D-glucosamine biosynthesis protein PgaD	*Acinetobacter pittii*	Acinetobacter genomospp. 3	A0A6H2VP90	--	30036696	Not Available	Not Available
1562	Polysaccharide deacetylase	*Xenorhabdus stockiae*		A0A2D0KWI4	--	28993611	Hydrolase activity, acting on carbon-nitrogen (but not peptide) bonds	Not Available
1563	GGDEF family protein	*Vibrio cholerae serotype O1*	Strain ATCC 39315 / El Tor Inaba N16961	Q9KPY8	--	10952301	Diguanylate cyclase activity	Not Available
1564	GGDEF family protein	*Vibrio cholerae serotype O1*	Strain ATCC 39315 / El Tor Inaba N16961	Q9KN01	--	10952301	Diguanylate cyclase activity	Not Available
1565	GGDEF family protein	*Vibrio cholerae serotype O1*	Strain ATCC 39315 / El Tor Inaba N16961	Q9KS83	--	10952301	Diguanylate cyclase activity	Not Available
1566	Poly-beta-1, 6-N-acetyl-D-glucosamine synthase (Poly-beta-1, 6-GlcNAc synthase) (EC 2.4.1.-)	*Lachnospiraceae bacterium*		A0A3D0DPD1	--	30148503	Acetyl glucosaminyl transferase activity	Not Available
1567	GGDEF family protein	*Vibrio cholerae serotype O1*	Strain ATCC 39315 / El Tor Inaba N16961	Q9KN91	--	10952301	Diguanylate cyclase activity	Not Available
1568	GGDEF family protein	*Vibrio cholerae serotype O1*	Strain ATCC 39315 / El Tor Inaba N16961	Q9KNB6	--	10952301	Diguanylate cyclase activity	Not Available
1569	GGDEF family protein	*Vibrio cholerae serotype O1*	Strain ATCC 39315 / El Tor Inaba N16961	Q9KPS7	--	10952301	Diguanylate cyclase activity	Not Available
1570	Uncharacterized protein	*Cupriavidus necator*	Strain ATCC 17699 / DSM 428 / KCTC 22496 / NCIMB 10442 / H16 / stanier 337 Ralstonia eutropha	Q0KF48	--	16964242	Not Available	Not Available
1571	GGDEF family protein	*Vibrio cholerae serotype O1*	Strain ATCC 39315 / El Tor Inaba N16961	Q9KS92	--	10952301	Diguanylate cyclase activity	Not Available
1572	Surface composition regulator	*Serratia spp. AS13*		A0A7U3Z3G0	--	23450001	Not Available	Not Available
1573	mRNA interferase MqsR	*Betaproteobacteria bacterium RBG_16_58_11*		A0A1F3X5Y1	--	27774985	Not Available	Not Available
1574	Uncharacterized protein	*Azospirillum lipoferum*	Strain 4B	G7Z3P9	--	22216014	Not Available	Not Available
1575	Poly-beta-1, 6-N-acetyl-D-glucosamine N-deacetylase PgaB	*Acinetobacter pittii*	Acinetobacter genomospp. 3	A0A6H2VGR7	--	30036696	Hydrolase activity, acting on carbon-nitrogen (but not peptide) bonds	Not Available
1576	Regulator of the extracellular matrix, regulation of biofilm formation	*Bacillus methanolicus PB1*		I3E0X0	--	22610424	Not Available	Not Available
1577	Poly-beta-1, 6-N-acetyl-D-glucosamine N-deacetylase PgaB	*Chromobacterium haemolyticum*		A0A2T5P0U5	--	29670144	Hydrolase activity, acting on carbon-nitrogen (but not peptide) bonds	Not Available
1578	Lipoprotein for biofilm formation	*Bacillus spizizenii*	Strain ATCC 23059 / NRRL B-14472 / W23 Bacillus Subtilis subspp. spizizenii	E0U410	--	21527469	Not Available	Not Available
1579	Poly-beta-1, 6-N-acetyl-D-glucosamine N-deacetylase PgaB	*Acinetobacter seifertii*		N8SA96	--	25563912	Hydrolase activity, acting on carbon-nitrogen (but not peptide) bonds	Not Available
1580	Uncharacterized protein	*Gammaproteobacteria bacterium HGW-Gammaproteobacteria-14*		A0A2N1YKL6	--	28350393	Not Available	Not Available
1581	Poly-beta-1, 6-N-acetyl-D-glucosamine biosynthesis protein PgaD	*Enterobacter chengduensis*		A0A5C1C3Z6	--	30302649	Not Available	Not Available
1582	Poly-beta-1, 6-N-acetyl-D-glucosamine synthase (Poly-beta-1, 6-GlcNAc synthase) (EC 2.4.1.-)	*Enterobacter chengduensis*		A0A5C1C402	--	30302649	Acetyl glucosaminyl transferase activity	Not Available

Target ID	Bacterial Protein Name	Organism Name	Strain	UniProt ID	PDB ID	PubMed ID	Classification as per UniProt GO Molecular Function	Classification of Function as per PDB
1583	Poly-beta-1, 6-N-acetyl-D-glucosamine synthase (Poly-beta-1, 6-GlcNAc synthase) (EC 2.4.1.-)	*Pseudomonas fluorescens*	Strain Pf0-1	Q3KJY3	--	19432983	Acetyl glucosaminyl transferase activity	Not Available
1584	Hemin storage system, HmsF protein	*Pseudomonas fluorescens*	Strain Pf0-1	Q3KJY4	--	19432983	Hydrolase activity, acting on carbon-nitrogen (but not peptide) bonds	Not Available
1585	Putative hemin storage system	*Pseudomonas fluorescens*	Strain Pf0-1	Q3KJY2	--	19432983	Not Available	Not Available
1586	Type II toxin-antitoxin system MqsR family toxin	*Pseudomonas spp. WS 5019*		A0A7Y1FEC9	--	32587583	Not Available	Not Available
1587	Uncharacterized protein	*Pseudomonas putida ND6*		I3V0P5	--	22933774	Not Available	Not Available
1588	Type II toxin-antitoxin system MqsR family toxin	*Oxalobacteraceae bacterium*		A0A4Q3HUY0	--	30498029	Not Available	Not Available
1589	Poly-beta-1, 6-N-acetyl-D-glucosamine N-deacetylase PgaB (Fragment)	*Oxalobacteraceae bacterium*		A0A4Q3K4V3	--	30498029	Hydrolase activity, acting on carbon-nitrogen (but not peptide) bonds	Not Available
1590	Poly-beta-1, 6-N-acetyl-D-glucosamine N-deacetylase PgaB	*Pelosinus spp. UFO1*		A0A075KI64	--	25189589	Hydrolase activity, acting on carbon-nitrogen (but not peptide) bonds	Not Available
1591	Poly-beta-1, 6-N-acetyl-D-glucosamine N-deacetylase PgaB	*Pelosinus spp. UFO1*		A0A075KDY1	--	25189589	Hydrolase activity, acting on carbon-nitrogen (but not peptide) bonds	Not Available
1592	HmsF protein	*Klebsiella pneumoniae subspp. rhinoscleromatis SB3432*		R4Y4I7	--	21629720	Hydrolase activity, acting on carbon-nitrogen (but not peptide) bonds	Not Available
1593	HmsS protein	*Klebsiella pneumoniae subspp. rhinoscleromatis SB3432*		R4YAK7	--	21629720	Not Available	Not Available
1594	Surface composition regulator	*Salmonella enterica subspp. enterica serovar*	Uganda Str. R8-3404	A0A6C8GYH9	--	21859443	Not Available	Not Available
1595	Poly-beta-1, 6-N-acetyl-D-glucosamine synthase (Poly-beta-1, 6-GlcNAc synthase) (EC 2.4.1.-)	*Pelosinus spp. UFO1*		A0A075KH19	--	25189589	Acetyl glucosaminyl transferase activity	Not Available
1596	Type II toxin-antitoxin system MqsR family toxin	*Lentisphaerae bacterium*		A0A7X8XB07	--	32123542	Not Available	Not Available
1597	Poly-beta-1, 6-N-acetyl-D-glucosamine N-deacetylase PgaB	*Lentisphaerae bacterium GWF2_52_8*		A0A1G0ZGS0	--	27774985	Hydrolase activity, acting on carbon-nitrogen (but not peptide) bonds	Not Available
1598	Outer membrane N-deacetylase	*Pseudomonas aestus*		U6ZQ67	--	24285649	Hydrolase activity, acting on carbon-nitrogen (but not peptide) bonds	Not Available
1599	Poly-beta-1, 6-N-acetyl-D-glucosamine biosynthesis protein PgaD	*Acinetobacter spp. ABNIH27*		A0A2S4T987	--	29437920	Not Available	Not Available
1600	Poly-beta-1, 6-N-acetyl-D-glucosamine synthase (Poly-beta-1, 6-GlcNAc synthase) (EC 2.4.1.-)	*Pseudomonas aestus*		U6ZPI1	--	24285649	Acetyl glucosaminyl transferase activity	Not Available
1601	Biofilm PGA synthesis protein PgaD	*Pseudomonas aestus*		U6ZVB6	--	24285649	Not Available	Not Available
1602	Poly-beta-1, 6-N-acetyl-D-glucosamine biosynthesis protein PgaD	*Lentisphaerae bacterium GWF2_52_8*		A0A1G0ZGL6	--	27774985	Not Available	Not Available
1603	Poly-beta-1, 6-N-acetyl-D-glucosamine N-deacetylase PgaB	*Acinetobacter spp. ABNIH27*		A0A2S4TAA7	--	29437920	Hydrolase activity, acting on carbon-nitrogen (but not peptide) bonds	Not Available
1604	Poly-beta-1, 6-N-acetyl-D-glucosamine N-deacetylase PgaB	*Lentisphaerae bacterium*		A0A7V4HHC5	--	31911466	Hydrolase activity, acting on carbon-nitrogen (but not peptide) bonds	Not Available
1605	Poly-beta-1, 6-N-acetyl-D-glucosamine biosynthesis protein PgaD	*Lentisphaerae bacterium GWF2_49_21*		A0A1G0Y752	--	27774985	Not Available	Not Available
1606	Poly-beta-1, 6-N-acetyl-D-glucosamine biosynthesis protein PgaD	*Lentisphaerae bacterium*		A0A7C5AG76	--	31911466	Not Available	Not Available
1607	Poly-beta-1, 6-N-acetyl-D-glucosamine biosynthesis protein PgaD	*Escherichia spp. ESNIH1*		A0A2S4QVY0	--	29437920	Not Available	Not Available
1608	Poly-beta-1, 6-N-acetyl-D-glucosamine synthase (Poly-beta-1, 6-GlcNAc synthase) (EC 2.4.1.-)	*Lentisphaerae bacterium GWF2_49_21*		A0A1G0Y743	--	27774985	Acetyl glucosaminyl transferase activity	Not Available
1609	Poly-beta-1, 6-N-acetyl-D-glucosamine synthase (Poly-beta-1, 6-GlcNAc synthase) (EC 2.4.1.-)	*Escherichia spp. ESNIH1*		A0A2S4QVY8	--	29437920	Acetyl glucosaminyl transferase activity	Not Available
1610	Uncharacterized protein	*Lentisphaerae bacterium*		A0A7X9AUX7	--	32123542	Not Available	Not Available
1611	Poly-beta-1, 6-N-acetyl-D-glucosamine synthase (Poly-beta-1, 6-GlcNAc synthase) (EC 2.4.1.-)	*Acinetobacter spp. ABNIH27*		A0A2S4T939	--	29437920	Acetyl glucosaminyl transferase activity	Not Available
1612	Poly-beta-1, 6-N-acetyl-D-glucosamine N-deacetylase PgaB	*Acinetobacter spp. ABNIH27*		A0A2S4T902	--	29437920	Hydrolase activity, acting on carbon-nitrogen (but not peptide) bonds	Not Available
1613	Poly-beta-1, 6-N-acetyl-D-glucosamine synthase (Poly-beta-1, 6-GlcNAc synthase) (EC 2.4.1.-)	*Acinetobacter spp. ABNIH27*		A0A2S4TAB3	--	29437920	Acetyl glucosaminyl transferase activity	Not Available
1614	Poly-beta-1, 6-N-acetyl-D-glucosamine N-deacetylase PgaB	*Lentisphaerae bacterium GWF2_49_21*		A0A1G0Y7B1	--	27774985	Hydrolase activity, acting on carbon-nitrogen (but not peptide) bonds	Not Available
1615	Poly-beta-1, 6-N-acetyl-D-glucosamine biosynthesis protein PgaD	*Acinetobacter spp. ABNIH27*		A0A2S4TAC3	--	29437920	Not Available	Not Available
1616	Poly-beta-1, 6-N-acetyl-D-glucosamine biosynthesis protein PgaD	*Lentisphaerae bacterium*		A0A7V4HI93	--	31911466	Not Available	Not Available
1617	Type II toxin-antitoxin system MqsR family toxin	*Lentisphaerae bacterium*		A0A7X7M3S1	--	32123542	Not Available	Not Available
1618	Response regulator	*Xanthomonas campestris pv. campestris*	Strain ATCC 33913 / DSM 3586 / NCPPB 528 / LMG 568 / P 25	Q8P4H8	--	12024217	Diguanylate cyclase activity	Not Available
1619	Biofilm PGA synthesis lipoprotein PgaB	*Aggregatibacter actinomycetemcomitans RhAA1*		H0KC42	--	22328766	Hydrolase activity, acting on carbon-nitrogen (but not peptide) bonds	Not Available
1620	Surface composition regulator	*Cronobacter condimenti 1330*		K8ACP6	--	23166675	Not Available	Not Available
1621	Sensor histidinekinase	*Xanthomonas campestris pv. campestris*	Strain ATCC 33913 / DSM 3586 / NCPPB 528 / LMG 568 / P 25	Q8PAN4	--	12024217	Diguanylate cyclase activity, kinase activity	Not Available
1622	GGDEF domain-containing protein	*Xanthomonas campestris pv. campestris*	Strain ATCC 33913 / DSM 3586 / NCPPB 528 / LMG 568 / P 25	Q8PDD3	--	12024217	Diguanylate cyclase activity	Not Available
1623	Uncharacterized protein	*Sulfuricurvum kujiense*	Strain ATCC BAA-921 / DSM 16994 / JCM 11577 / YK-1	E4U029	--	22675602	Not Available	Not Available
1624	Biofilm formation protein	*Bacillus licheniformis CG-B52*		T5HTA9	--	27174263	Not Available	Not Available
1625	Type II toxin-antitoxin system MqsR family toxin	*Sinorhizobium terangae*		A0A6N7LAM3	--	23425606	Not Available	Not Available
1626	Sensor histidinekinase	*Xanthomonas campestris pv. campestris*	Strain ATCC 33913 / DSM 3586 / NCPPB 528 / LMG 568 / P 25	Q8PB32	--	12024217	Diguanylate cyclase activity, kinase activity	Not Available
1627	GGDEF domain-containing protein	*Xanthomonas campestris pv. campestris*	Strain ATCC 33913 / DSM 3586 / NCPPB 528 / LMG 568 / P 25	Q8PAN6	--	12024217	Diguanylate cyclase activity	Not Available

Target ID	Bacterial Protein Name	Organism Name	Strain	UniProt ID	PDB ID	PubMed ID	Classification as per UniProt GO Molecular Function	Classification of Function as per PDB
1628	GGDEF family protein	*Xanthomonas campestris pv. campestris*	Strain ATCC 33913 / DSM 3586 / NCPPB 528 / LMG 568 / P 25	Q8P9F2	--	12024217	Diguanylate cyclase activity	Not Available
1629	Polysaccharide deacetylase	*Sulfuricurvum kujiense*	Strain ATCC BAA-921 / DSM 16994 / JCM 11577 / YK-1	E4U031	--	22675602	Hydrolase activity, acting on carbon-nitrogen (but not peptide) bonds	Not Available
1630	GGDEF domain-containing protein	*Xanthomonas campestris pv. campestris*	Strain ATCC 33913 / DSM 3586 / NCPPB 528 / LMG 568 / P 25	Q8P502	--	12024217	Diguanylate cyclase activity	Not Available
1631	Uncharacterized protein	*Methylotenera versatilis*	Strain 301	D7DJ79	--	21622745	Not Available	Not Available
1632	Poly-beta-1, 6-N-acetyl-D-glucosamine synthase (Poly-beta-1, 6-GlcNAc synthase) (EC 2.4.1.-)	*Methylotenera versatilis*	Strain 301	D7DK50	--	21622745	Acetyl glucosaminyl transferase activity	Not Available
1633	Polysaccharide deacetylase	*Methylotenera versatilis*	Strain 301	D7DJ81	--	21622745	Hydrolase activity, acting on carbon-nitrogen (but not peptide) bonds	Not Available
1634	GGDEF family protein	*Xanthomonas campestris pv. campestris*	Strain ATCC 33913 / DSM 3586 / NCPPB 528 / LMG 568 / P 25	Q8P528	--	12024217	Diguanylate cyclase activity	Not Available
1635	Uncharacterized protein	*Anaeromyxobacter spp. RBG_16_69_14*		A0A1F3C6Q7	--	27774985	Not Available	Not Available
1636	Poly-beta-1, 6-N-acetyl-D-glucosamine synthase (Poly-beta-1, 6-GlcNAc synthase) (EC 2.4.1.-)	*Pseudomonas brassicacearum*	Strain NFM421	F2KD33	--	21515771	Acetyl glucosaminyl transferase activity	Not Available
1637	Poly-beta-1, 6-N-acetyl-D-glucosamine N-deacetylase	*Escherichia spp. ESNIH1*		A0A2S4QVZ5	--	29437920	Hydrolase activity, acting on carbon-nitrogen (but not peptide) bonds	Not Available
1638	Type II toxin-antitoxin system MqsR family toxin	*Verrucomicrobiales bacterium*		A0A354TEC8	--	30148503	Not Available	Not Available
1639	Uncharacterized protein	*Candidatus Rokubacteria bacterium GWC2_70_16*		A0A1F7LKG1	--	27774985	Not Available	Not Available
1640	Poly-beta-1, 6-N-acetyl-D-glucosamine biosynthesis protein PgaD	*Clostridium spp.*		A0A7X7XZA3	--	32123542	Not Available	Not Available
1641	NodB homology domain-containing protein	*Geoalkalibacter subterraneus*		A0A0B5FGS1	--	25767222	Hydrolase activity, acting on carbon-nitrogen (but not peptide) bonds	Not Available
1642	Poly-beta-1, 6-N-acetyl-D-glucosamine synthase (Poly-beta-1, 6-GlcNAc synthase) (EC 2.4.1.-)	*Klebsiella pneumoniae subspp. pneumoniae DSM 30104 = JCM 1662 = NBRC 14940*		J2XCJ6	--	23012294	Acetyl glucosaminyl transferase activity	Not Available
1643	Poly-beta-1, 6-N-acetyl-D-glucosamine N-deacetylase PgaB (EC 3.5.1.-)	*Lysobacter capsici*		A0A0S2FSL6	--	26597042	Hydrolase activity, acting on carbon-nitrogen (but not peptide) bonds	Not Available
1644	Uncharacterized protein	*Geoalkalibacter subterraneus*		A0A0B5FT76	--	25767222	Not Available	Not Available
1645	Poly-beta-1, 6-N-acetyl-D-glucosamine biosynthesis protein PgaD	*Lysobacter capsici*		A0A0S2FSG4	--	26597042	Not Available	Not Available
1646	Uncharacterized protein	*Romboutsia maritimum*		A0A371IQ26	--	29025937	Not Available	Not Available
1647	Type II toxin-antitoxin system MqsR family toxin	*Dehalococcoidia bacterium*		A0A7X7GXM7	--	32123542	Not Available	Not Available
1648	Poly-beta-1, 6-N-acetyl-D-glucosamine synthase (Poly-beta-1, 6-GlcNAc synthase) (EC 2.4.1.-)	*Lysobacter capsici*		A0A0S2FSU4	--	26597042	Acetyl glucosaminyl transferase activity	Not Available
1649	Poly-beta-1, 6-N-acetyl-D-glucosamine synthase (Poly-beta-1, 6-GlcNAc synthase) (EC 2.4.1.-)	*Geoalkalibacter subterraneus*		A0A0B5FUS7	--	25767222	Acetyl glucosaminyl transferase activity	Not Available
1650	Surface composition regulator	*Serratia inhibens PRI-2C*		A0A1B1KT89	--	22815440	Not Available	Not Available
1651	Uncharacterized protein	*Strain Marseille Minibacterium maSilienSiS*		J3G480	--	23045501	Not Available	Not Available
1652	Hemin storage protein	*Janthinobacterium spp.*	Strain Marseille Minibacterium masiliensis	A6T1E1	--	17722982	Hydrolase activity, acting on carbon-nitrogen (but not peptide) bonds	Not Available
1653	Poly-beta-1, 6-N-acetyl-D-glucosamine synthase (Poly-beta-1, 6-GlcNAc synthase) (EC 2.4.1.-)	*Janthinobacterium spp.*	v	A6T1E0	--	17722982	Acetyl glucosaminyl transferase activity	Not Available
1654	Uncharacterized protein	*Dehalococcoidia bacterium*		A0A7C2WC69	--	32372496	Not Available	Not Available
1655	Poly-beta-1, 6-N-acetyl-D-glucosamine N-deacetylase PgaB	*Proteobacteria bacterium*		A0A6H9I2Q0	--	31478060	Hydrolase activity, acting on carbon-nitrogen (but not peptide) bonds	Not Available
1656	Outer membrane N-deacetylase	*Klebsiella pneumoniae subspp. pneumoniae DSM 30104 = JCM 1662 = NBRC 14940*		J2DQS3	--	23012294	Hydrolase activity, acting on carbon-nitrogen (but not peptide) bonds	Not Available
1657	Poly-beta-1, 6-N-acetyl-D-glucosamine biosynthesis protein PgaD	*Proteobacteria bacterium*		A0A6H9ICA2	--	31478060	Not Available	Not Available
1658	Poly-beta-1, 6-N-acetyl-D-glucosamine synthase (Poly-beta-1, 6-GlcNAc synthase) (EC 2.4.1.-)	*Proteobacteria bacterium*		A0A6H9IFG5	--	31478060	Acetyl glucosaminyl transferase activity	Not Available
1659	Uncharacterized conserved protein	*Janthinobacterium spp.*	Strain Marseille Minibacterium masiliensis	A6T1D9	--	17722982	Not Available	Not Available
1660	Biofilm PGA synthesis lipoprotein PgaB	*Actinobacillus pleuropneumoniae serovar 10*	Str. D13039	E0F777	--	20802045	Hydrolase activity, acting on carbon-nitrogen (but not peptide) bonds	Not Available
1661	Biofilm PGA synthesis auxiliary protein PgaD	*Burkholderia pyrrocinia*	Pseudomonas pyrrocinia	A0A0G3WZX8	--	26150017	Not Available	Not Available
1662	Hemin storage protein	*Burkholderia pyrrocinia*	Pseudomonas pyrrocinia	A0A0G3X357	--	26150017	Hydrolase activity, acting on carbon-nitrogen (but not peptide) bonds	Not Available
1663	Uncharacterized protein	*Tistrella mobilis*	Strain KA081020-065	I3TVX0	--	22458477	Not Available	Not Available
1664	Uncharacterized protein	*Acinetobacter oleivorans*	Strain JCM 16667 / KCTC 23045 / DR1	D8JE02	--	20639327	Not Available	Not Available
1665	Poly-beta-1, 6-N-acetyl-D-glucosamine synthase (Poly-beta-1, 6-GlcNAc synthase) (EC 2.4.1.-)	*Deltaproteobacteria bacterium*		A0A7V3KXN3	--	31911466	Acetyl glucosaminyl transferase activity	Not Available
1666	Poly-beta-1, 6-N-acetyl-D-glucosamine synthase (Poly-beta-1, 6-GlcNAc synthase) (EC 2.4.1.-)	*Shigella flexneri serotype 5b*	Strain 8401	Q0T614	--	16822325	Acetyl glucosaminyl transferase activity	Not Available
1667	Poly-beta-1, 6-N-acetyl-D-glucosamine biosynthesis protein PgaD	*Deltaproteobacteria bacterium*		A0A7X6PVS7	--	32123542	Not Available	Not Available
1668	Poly-beta-1, 6-N-acetyl-D-glucosamine N-deacetylase PgaB	*Deltaproteobacteria bacterium*		A0A7V3NYI2	--	31911466	Hydrolase activity, acting on carbon-nitrogen (but not peptide) bonds	Not Available
1669	Uncharacterized protein	*Shigella flexneri serotype 5b*	Strain 8401	Q0T615	--	16822325	Not Available	Not Available
1670	Uncharacterized protein	*Deltaproteobacteria bacterium*		A0A7V0QJM4	--	31911466	Not Available	Not Available
1671	Type II toxin-antitoxin system MqsR family toxin	*Thermodesulfatator atlanticus*		A0A7V5NYK0	--	31911466	Not Available	Not Available
1672	Outer membrane N-deacetylase	*Pseudomonas fluorescens LMG 5329*		A0A0A1Z5R3	--	23887909	Hydrolase activity, acting on carbon-nitrogen (but not peptide) bonds	Not Available

Target ID	Bacterial Protein Name	Organism Name	Strain	UniProt ID	PDB ID	PubMed ID	Classification as per UniProt GO Molecular Function	Classification of Function as per PDB
1673	Poly-beta-1, 6-N-acetyl-D-glucosamine synthase (Poly-beta-1, 6-GlcNAc synthase) (EC 2.4.1.-)	*Pseudomonas fluorescens LMG 5329*		A0A0A1Z9D1	--	23887909	Acetyl glucosaminyl transferase activity	Not Available
1674	Biofilm PGA synthesis protein PgaD	*Pseudomonas fluorescens LMG 5329*		A0A0A1Z9Y2	--	23887909	Not Available	Not Available
1675	Poly-beta-1, 6-N-acetyl-D-glucosamine N-deacetylase PgaB	*Deltaproteobacteria bacterium*		A0A7V0JQ66	--	31911466	Hydrolase activity, acting on carbon-nitrogen (but not peptide) bonds	Not Available
1676	Poly-beta-1, 6-N-acetyl-D-glucosamine N-deacetylase PgaB (Fragment)	*Deltaproteobacteria bacterium*		A0A7X6PV65	--	32123542	Hydrolase activity, acting on carbon-nitrogen (but not peptide) bonds	Not Available
1677	Surface composition regulator	*Salmonella enterica subspp. enterica serovar*	WandSworth Str. A4-580	G5SH05	--	21859443	Not Available	Not Available
1678	Motility quorum-sensing regulator MqsR	*Pseudomonas fluorescens LMG 5329*		A0A0A1YWG5	--	23887909	Not Available	Not Available
1679	Uncharacterized protein	*Companilactobacillus farciminis*		A0A4V3A398	--	30830251	Not Available	Not Available
1680	Poly-beta-1, 6-N-acetyl-D-glucosamine synthase (Poly-beta-1, 6-GlcNAc synthase) (EC 2.4.1.-)	*Deltaproteobacteria bacterium*		A0A7V0JR38	--	31911466	Acetyl glucosaminyl transferase activity	Not Available
1681	Poly-beta-1, 6-N-acetyl-D-glucosamine synthase (Poly-beta-1, 6-GlcNAc synthase) (EC 2.4.1.-)	*Deltaproteobacteria bacterium*		A0A7C1B4X9	--	31911466	Acetyl glucosaminyl transferase activity	Not Available
1682	Poly-beta-1, 6-N-acetyl-D-glucosamine synthase (Poly-beta-1, 6-GlcNAc synthase) (EC 2.4.1.-)	*Stenotrophomonas maltophilia*	Pseudomonas maltophilia Xanthomonas maltophilia	A0A7Z1RIE6	--	29170656	Acetyl glucosaminyl transferase activity	Not Available
1683	Surface composition regulator	*Salmonella enterica subspp. enterica serovar Rubislaw str. A4-653*		G5QPX3	--	21859443	Not Available	Not Available
1684	Poly-beta-1, 6-N-acetyl-D-glucosamine biosynthesis protein PgaD	*Stenotrophomonas maltophilia*	Pseudomonas maltophilia Xanthomonas maltophilia	A0A7Z1RIY8	--	29170656	Not Available	Not Available
1685	Poly-beta-1, 6-N-acetyl-D-glucosamine biosynthesis protein PgaD	*Deltaproteobacteria bacterium*		A0A7C1BIY6	--	31911466	Not Available	Not Available
1686	Uncharacterized protein	*Polaromona spp.*	Strain JS666 / ATCC BAA-500	Q11Z97	--	18723656	Not Available	Not Available
1687	Poly-beta-1, 6-N-acetyl-D-glucosamine biosynthesis protein PgaD	*Stenotrophomonas maltophilia*	Pseudomonas maltophilia Xanthomonas maltophilia	A0A2Y9U8G9	--	29170656	Not Available	Not Available
1688	Poly-beta-1, 6-N-acetyl-D-glucosamine N-deacetylase PgaB	*Stenotrophomonas maltophilia*	Pseudomonas maltophilia Xanthomonas maltophilia	A0A7Z1RJ06	--	29170656	Hydrolase activity, acting on carbon-nitrogen (but not peptide) bonds	Not Available
1689	Poly-beta-1, 6-N-acetyl-D-glucosamine biosynthesis protein PgaD	*Chromobacterium spp. Panama*		A0A2T5NK43	--	29670144	Not Available	Not Available
1690	Poly-beta-1, 6-N-acetyl-D-glucosamine N-deacetylase PgaB	*Deltaproteobacteria bacterium*		A0A7C1B8G0	--	31911466	Hydrolase activity, acting on carbon-nitrogen (but not peptide) bonds	Not Available
1691	Type II toxin-antitoxin system MqsR family toxin	*Deltaproteobacteria bacterium*		A0A3A5ARM9	--	28644444	Not Available	Not Available
1692	Poly-beta-1, 6-N-acetyl-D-glucosamine synthase (Poly-beta-1, 6-GlcNAc synthase) (EC 2.4.1.-)	*Escherichia coli O55:H7*	Strain CB9615 / EPEC	D3QPZ6	--	20090843	Acetyl glucosaminyl transferase activity	Not Available
1693	Poly-beta-1, 6-N-acetyl-D-glucosamine synthase (Poly-beta-1, 6-GlcNAc synthase) (EC 2.4.1.-)	*Chromobacterium spp. Panama*		A0A2T5NQP9	--	29670144	Acetyl glucosaminyl transferase activity	Not Available
1694	Poly-beta-1, 6-N-acetyl-D-glucosamine N-deacetylase PgaB	*Chromobacterium spp. Panama*		A0A2T5NK52	--	29670144	Hydrolase activity, acting on carbon-nitrogen (but not peptide) bonds	Not Available
1695	Biofilm PGA synthesis protein pgaD	*Escherichia coli O55:H7*	Strain CB9615 / EPEC	D3QPZ5	--	20090843	Not Available	Not Available
1696	Surface composition regulator	*Escherichia coli O55:H7*	Strain CB9615 / EPEC	D3QRZ4	--	20090843	Not Available	Not Available
1697	Bll4347 protein	*Bradyrhizobium diazoefficiens*	Strain JCM 10833 / BCRC 13528 / IAM 13628 / NBRC 14792 / USDA 110	Q89M46	--	12597275	Diguanylate cyclase activity	Not Available
1698	Bll7340 protein	*Bradyrhizobium diazoefficiens*	Strain JCM 10833 / BCRC 13528 / IAM 13628 / NBRC 14792 / USDA 110	Q89DU8	--	12597275	Diguanylate cyclase activity	Not Available
1699	Blr2401 protein	*Bradyrhizobium diazoefficiens*	Strain JCM 10833 / BCRC 13528 / IAM 13628 / NBRC 14792 / USDA 110	Q89SJ9	--	12597275	Diguanylate cyclase activity	Not Available
1700	Bll3041 protein	*Bradyrhizobium diazoefficiens*	Strain JCM 10833 / BCRC 13528 / IAM 13628 / NBRC 14792 / USDA 110	Q89QT3	--	12597275	Diguanylate cyclase activity	Not Available
1701	Surface composition regulator	*Citrobacter rodentium*	Strain ICC168 Citrobacter freundii biotype 4280	D2TRP2	--	19897651	Not Available	Not Available
1702	Poly-beta-1, 6-N-acetyl-D-glucosamine biosynthesis protein PgaD	*Stenotrophomonas spp. SAU14A_naIMI4_5*		A0A3S7KI35	--	29765358	Not Available	Not Available
1703	Blr0624 protein	*Bradyrhizobium diazoefficiens*	Strain JCM 10833 / BCRC 13528 / IAM 13628 / NBRC 14792 / USDA 110	Q89WQ4	--	12597275	Diguanylate cyclase activity	Not Available
1704	Bll1362 protein	*Bradyrhizobium diazoefficiens*	Strain JCM 10833 / BCRC 13528 / IAM 13628 / NBRC 14792 / USDA 110	Q89UP9	--	12597275	Diguanylate cyclase activity	Not Available
1705	Bll2326 protein	*Bradyrhizobium diazoefficiens*	Strain JCM 10833 / BCRC 13528 / IAM 13628 / NBRC 14792 / USDA 110	Q89SS4	--	12597275	Diguanylate cyclase activity	Not Available
1706	Poly-beta-1, 6-N-acetyl-D-glucosamine biosynthesis protein PgaD	*Stenotrophomonas spp. SAU14A_naIMI4_5*		A0A3S7KGX6	--	29765358	Not Available	Not Available
1707	Bll4961 protein	*Bradyrhizobium diazoefficiens*	Strain JCM 10833 / BCRC 13528 / IAM 13628 / NBRC 14792 / USDA 110	Q89KE6	--	12597275	Diguanylate cyclase activity	Not Available
1708	Motility quorum-sensing regulator MqsR	*Gallionellales bacterium RIFCSPLOWO2_02_58_13*		A0A1G0DUP1	--	27774985	Not Available	Not Available
1709	Poly-beta-1, 6-N-acetyl-D-glucosamine biosynthesis protein PgaD	*Xenorhabdus spp. KJ12.1*		A0A2D0LA77	--	28993611	Not Available	Not Available
1710	Uncharacterized protein	*Gallionellales bacterium RIFCSPLOWO2_02_58_13*		A0A1G0DV73	--	27774985	Not Available	Not Available

Target ID	Bacterial Protein Name	Organism Name	Strain	UniProt ID	PDB ID	PubMed ID	Classification as per UniProt GO Molecular Function	Classification of Function as per PDB
1711	Poly-beta-1, 6-N-acetyl-D-glucosamine N-deacetylase PgaB	*Stenotrophomonas maltophilia*	Pseudomonas maltophilia Xanthomonas maltophilia	A0A2J0SHH4	--	29170656	Hydrolase activity, acting on carbon-nitrogen (but not peptide) bonds	Not Available
1712	Poly-beta-1, 6-N-acetyl-D-glucosamine synthase (Poly-beta-1, 6-GlcNAc synthase) (EC 2.4.1.-)	*Xenorhabdus spp. KJ12.1*		A0A2D0LAE0	--	28993611	Acetyl glucosaminyl transferase activity	Not Available
1713	Poly-beta-1, 6-N-acetyl-D-glucosamine biosynthesis protein PgaD	*Stenotrophomonas maltophilia*	Pseudomonas maltophilia Xanthomonas maltophilia	A0A2J0TWJ3	--	29170656	Not Available	Not Available
1714	Poly-beta-1, 6-N-acetyl-D-glucosamine N-deacetylase PgaB	*Stenotrophomonas maltophilia*	Pseudomonas maltophilia Xanthomonas maltophilia	A0A2J0TWJ1	--	29170656	Hydrolase activity, acting on carbon-nitrogen (but not peptide) bonds	Not Available
1715	Poly-beta-1, 6-N-acetyl-D-glucosamine N-deacetylase PgaB	*Stenotrophomonas maltophilia*	Pseudomonas maltophilia Xanthomonas maltophilia	A0A2J0UEW1	--	29170656	Hydrolase activity, acting on carbon-nitrogen (but not peptide) bonds	Not Available
1716	Poly-beta-1, 6-N-acetyl-D-glucosamine synthase (Poly-beta-1, 6-GlcNAc synthase) (EC 2.4.1.-)	*Pseudomonas fluorescens Q8r1-96*		I4L1A4	--	22792073	Acetyl glucosaminyl transferase activity	Not Available
1717	Surface composition regulator	*Salmonella enterica subspp. salamae serovar*	42:r:-	A0A731NDC1	--	30286803	Not Available	Not Available
1718	Acyl-homoserine-lactone synthase (EC 2.3.1.184) (Autoinducer synthesis protein)	*Serratia plymuthica*		C4PDX0	--	21284858	N-acyl homoserine lactone synthase activity	Not Available
1719	SplR	*Serratia plymuthica*		C4PDW8	--	21284858	DNA binding	Not Available
1720	Surface composition regulator	*Salmonella enterica subspp. houtenae serovar*	21:z4, z23:-	A0A752IW31	--	30286803	Not Available	Not Available
1721	IcaA (Fragment)	*Staphylococcus saprophyticus*		Q83ZH7	--	12957956	Not Available	Not Available
1722	Putative transporter protein	*Salmonella typhimurium*	Strain LT2 / SGSC1412 / ATCC 700720	Q8RSN8	--	11260463; 11929533	Not Available	Not Available
1723	Acyl-homoserine-lactone synthase (EC 2.3.1.184) (Autoinducer synthesis protein)	*Serratia plymuthica*		C4PDW7	--	21284858	N-acyl homoserine lactone synthase activity	Not Available
1724	Uncharacterized protein	*Bordetella bronchiseptica*	Strain ATCC BAA-588 / NCTC 13252 / RB50 Alcaligenes bronchisepticus	A0A0H3LPJ8	--	12910271	Not Available	Not Available
1725	Surface composition regulator	*Salmonella enterica subspp. enterica serovar*	Duisburg	A0A5X0EQI5	--	30286803	Not Available	Not Available
1726	Surface composition regulator	*Salmonella typhisuis*		A0A735MWU7	--	30286803	Not Available	Not Available
1727	Poly-beta-1, 6-N-acetyl-D-glucosamine synthase (Poly-beta-1, 6-GlcNAc synthase) (EC 2.4.1.-)	*Staphylococcus lugdunensis*		A8DN18	--	17635864	Acetyl glucosaminyl transferase activity	Not Available
1728	Poly-beta-1, 6-N-acetyl-D-glucosamine synthase (Poly-beta-1, 6-GlcNAc synthase) (EC 2.4.1.-)	*Pseudomonas synxantha BG33R*		I4KXU6	--	22792073	Acetyl glucosaminyl transferase activity	Not Available
1729	Surface composition regulator	*Salmonella enterica subspp. arizonae serovar*	18:z4, z32:-	A0A737EZC4	--	30286803	Not Available	Not Available
1730	Uncharacterized protein	*Yersinia enterocolitica*	Type O:5, 27 Str. YE149 / 02	A0A7U7FJ92	--	24753568	Not Available	Not Available
1731	Surface composition regulator	*Salmonella enterica subspp. enterica serovar*	Decatur	A0A6V9XHS2	--	30286803	Not Available	Not Available
1732	Poly-beta-1, 6-N-acetyl-D-glucosamine synthase (Poly-beta-1, 6-GlcNAc synthase) (EC 2.4.1.-)	*Xanthomonas hortorum pv. carotae*	Str. M081	V7ZDU8	--	21722296	Acetyl glucosaminyl transferase activity	Not Available
1733	Surface composition regulator	*Salmonella enterica subspp. arizonae serovar*	48:z4, z24:-	A0A738XIB5	--	30286803	Not Available	Not Available
1734	Poly-beta-1, 6-N-acetyl-D-glucosamine synthase (Poly-beta-1, 6-GlcNAc synthase) (EC 2.4.1.-)	*Yersinia enterocolitica*	Type O:5, 27 Str. YE149 / 02	A0A7U7IS30	--	24753568	Acetyl glucosaminyl transferase activity	Not Available
1735	Haemin storage system, HmsF protein	*Yersinia enterocolitica*	Type O:5, 27 Str. YE149 / 02	A0A7U7FN73	--	24753568	Hydrolase activity, acting on carbon-nitrogen (but not peptide) bonds	Not Available
1736	AI-2E family transporter	*Derxia gummosa DSM 723*		A0A8B6XBG3	--	16385049; 20559013	Not Available	Not Available
1737	HmsF protein	*Xanthomonas hortorum pv. carotae*	Str. M081	V7ZCS1	--	21722296	Hydrolase activity, acting on carbon-nitrogen (but not peptide) bonds	Not Available
1738	Surface composition regulator	*Salmonella enterica subspp. houtenae serovar*	18:z36, z38:-	A0A729K7U1	--	30286803	Not Available	Not Available
1739	Poly-beta-1, 6-N-acetyl-D-glucosamine synthase (Poly-beta-1, 6-GlcNAc synthase) (EC 2.4.1.-)	*Yersinia enterocolitica*	Type O:3 Str. YE12 / 03	A0A7U7IUF3	--	24753568	Acetyl glucosaminyl transferase activity	Not Available
1740	Uncharacterized protein	*uncultured bacterium*		K1YAI8	--	23019650	Not Available	Not Available
1741	Haemin storage system, HmsF protein	*Yersinia enterocolitica*	Type O:3 Str. YE12 / 03	A0A7U7ITX8	--	24753568	Hydrolase activity, acting on carbon-nitrogen (but not peptide) bonds	Not Available
1742	Biofilm PGA synthesis lipoprotein PgaB (EC 3.-.-.-)	*Pseudomonas synxantha BG33R*		I4KSD4	--	22792073	Hydrolase activity, acting on carbon-nitrogen (but not peptide) bonds	Not Available
1743	Haemin storage system, HmsS protein	*Yersinia enterocolitica*	Type O:3 Str. YE12 / 03	A0A7U7IUU3	--	24753568	Not Available	Not Available
1744	Surface composition regulator	*Yersinia enterocolitica*	Type O:3 Str. YE12 / 03	A0A7U7ITX3	--	24753568	DNA binding, DNA-directed DNA polymerase activity	Not Available
1745	Surface composition regulator	*Salmonella enterica subspp. salamae serovar*	42:b:1, 5	A0A737IX99	--	30286803	Not Available	Not Available
1746	S-ribosylhomocysteine lyase (EC 4.4.1.21) (AI-2 synthesis protein) (Autoinducer-2 production protein LuxS)	*Eikenella corrodens*		Q1HAV9	--	17027872	Iron ion binding, S-ribosylhomocysteine lyase activity	Not Available
1747	Putative biofilm PGA synthesis protein PgaD	*Pseudomonas synxantha BG33R*		I4KVN4	--	22792073	Not Available	Not Available
1748	Motility quorum-sensing regulator	*uncultured bacterium*		K1YWV6	--	23019650	Not Available	Not Available
1749	Surface composition regulator	*Salmonella enterica subspp. enterica serovar*	Saintpaul	A0A1S0ZD14	--	30286803	Not Available	Not Available
1750	Uncharacterized protein	*Yersinia enterocolitica*	Type O:3 Str. YE12 / 03	A0A7U7FV97	--	24753568	Not Available	Not Available
1751	Surface composition regulator	*Salmonella muenchen*		A0A3T3ENA5	--	30286803; 30902850	Not Available	Not Available
1752	Surface composition regulator	*Salmonella enterica subspp. enterica serovar*	Cerro	A0A2T9QB58	--	30286803; 31527278	Not Available	Not Available
1753	Poly-beta-1, 6-N-acetyl-D-glucosamine synthase (Poly-beta-1, 6-GlcNAc synthase) (EC 2.4.1.-)	*Pseudomonas fluorescens*		A0A0N9WUK7	--	29769716	Acetyl glucosaminyl transferase activity	Not Available
1754	Surface composition regulator	*Salmonella enterica subspp. enterica serovar*	Carrau	A0A752RN04	--	30286803	Not Available	Not Available
1755	PpuA (Fragment)	*Pseudomonas putida*	Arthrobacter siderocapsulatus	Q3Y8H5	--	16585751	Not Available	Not Available
1756	PsoB	*Pseudomonas putida*	Arthrobacter siderocapsulatus	A8MN37	--	16585751; 18599835	Catalytic activity, phosphopantetheine binding	Not Available
1757	IcaA (Fragment)	*Staphylococcus epidermidis*		Q83ZH6	--	12957956	Not Available	Not Available

Target ID	Bacterial Protein Name	Organism Name	Strain	UniProt ID	PDB ID	PubMed ID	Classification as per UniProt GO Molecular Function	Classification of Function as per PDB
1758	Surface composition regulator	*Salmonella enterica subspp. enterica serovar*	Abortusovis	A0A738AI74	--	30286803	Not Available	Not Available
1759	NodB homology domain-containing protein	*Escherichia coli*		C3TEU7	--	19439656	Hydrolase activity, acting on carbon-nitrogen (but not peptide) bonds	Not Available
1760	IcaA (Fragment)	*Macrococcus caseolyticus*		Q83ZI3	--	12957956	Not Available	Not Available
1761	Surface composition regulator	*Salmonella enterica subspp. VII serovar*	40:z4, z24:[z39]	A0A731XYY4	--	30286803	Not Available	Not Available
1762	Fimbrial protein FimH (Fragment)	*Escherichia coli*		A0A075Q3Y2	--	25012525	Not Available	Not Available
1763	PsoC	*Pseudomonas putida*	Arthrobacter siderocapsulatus	A8MN38	--	16585751; 18599835	Catalytic activity, phosphopantetheine binding	Not Available
1764	MacA	*Pseudomonas putida*	Arthrobacter siderocapsulatus	A8MN39	--	16585751; 18599835	Transmembrane transporter activity	Not Available
1765	Poly-beta-1, 6-N-acetyl-D-glucosamine synthase (Poly-beta-1, 6-GlcNAc synthase) (EC 2.4.1.-)	*Ralstonia syzygii R24*		G3A8J6	--	21931687	Acetyl glucosaminyl transferase activity	Not Available
1766	Surface composition regulator	*Salmonella enterica subspp. enterica serovar*	Sendai	A0A737RJS4	--	30286803	Not Available	Not Available
1767	HmsS	*Xenorhabdus nematophila*	Achromobacter nematophilus	B3GN24	--	18515487	Not Available	Not Available
1768	Fimbrial protein FimF (Fragment)	*Escherichia coli*		A0A075QDV6	--	25012525	Not Available	Not Available
1769	Uncharacterized protein	*Escherichia coli*		C3TEV7	--	19439656	Not Available	Not Available
1770	Fimbrial protein FimG	*Escherichia coli*		A0A075Q6V7	--	25012525	Not Available	Not Available
1771	HmsF	*Xenorhabdus nematophila*	Achromobacter nematophilus	B3GN22	--	18515487	Hydrolase activity, acting on carbon-nitrogen (but not peptide) bonds	Not Available
1772	Surface composition regulator	*Salmonella enterica subspp. salamae serovar*	42:f, g, t:--	A0A737HCW8	--	30286803	Not Available	Not Available
1773	NodB homology domain-containing protein	*Escherichia coli*		C3TEU8	--	19439656	Hydrolase activity, acting on carbon-nitrogen (but not peptide) bonds	Not Available
1774	Poly-beta-1, 6-N-acetyl-D-glucosamine synthase (Poly-beta-1, 6-GlcNAc synthase) (EC 2.4.1.-)	*Escherichia coli*		A0A6D1ART7	--	16943019	Acetyl glucosaminyl transferase activity	Not Available
1775	Uncharacterized protein	*Escherichia coli*		C3TEV0	--	19439656	Hydrolase activity, acting on carbon-nitrogen (but not peptide) bonds	Not Available
1776	Surface composition regulator	*Escherichia coli*		A0A7A2XRI7	--	30286803	Not Available	Not Available
1777	LuxR-like protein	*Pseudomonas putida*	Arthrobacter siderocapsulatus	A8MN41	--	16585751; 18599835	DNA binding	Not Available
1778	Ribosome maturation factor RimP	*Bacillus cereus*		A0A063CC81	--	25301660	Not Available	Not Available
1779	S-ribosylhomocysteine lyase (EC 4.4.1.21) (AI-2 synthesis protein) (Autoinducer-2 production protein LuxS)	*Bacillus cereus*		A0A063CL35	--	25301660	Iron ion binding, S-ribosylhomocysteine lyase activity	Not Available
1780	Surface composition regulator	*Pectobacterium parmentieri*		A0A0H3I8L4	--	23045508; 30326842	DNA binding, DNA-directed DNA polymerase activity	Not Available
1781	S-ribosylhomocysteine lyase (EC 4.4.1.21) (AI-2 synthesis protein) (Autoinducer-2 production protein LuxS)	*Edwardsiella tarda*	Strain EIB202	C5IWN6	--	19283379; 19865481	Iron ion binding, S-ribosylhomocysteine lyase activity	Not Available
1782	Poly-beta-1, 6-N-acetyl-D-glucosamine synthase (Poly-beta-1, 6-GlcNAc synthase) (EC 2.4.1.-)	*Pectobacterium parmentieri*		A0A0H3ICJ6	--	23045508; 30326842	Acetyl glucosaminyl transferase activity	Not Available
1783	Poly-beta-1, 6-N-acetyl-D-glucosamine N-deacetylase (Poly-beta-1, 6-N-acetyl-D-glucosamine N-deacetylase PgaB) (EC 3.5.1.-)	*Pseudomonas panacis*		A0A5M9I3H1	--	32587583	Hydrolase activity, acting on carbon-nitrogen (but not peptide) bonds	Not Available
1784	Motility quorum-sensing regulator MqsR	*Pseudomonas fluorescens*		A0A0N9WTK9	--	29769716	Not Available	Not Available
1785	Ribosome maturation factor RimM	*Bacillus cereus*		A0A063CCA6	--	25301660	ribosome binding	Not Available
1786	Poly-beta-1, 6-N-acetyl-D-glucosamine synthase (Poly-beta-1, 6-GlcNAc synthase) (EC 2.4.1.-)	*Staphylococcus aureus*		W8TQA2	--	26048971; 32562543; 33052925	Acetyl glucosaminyl transferase activity	Not Available
1787	Poly-beta-1, 6-N-acetyl-D-glucosamine synthase (Poly-beta-1, 6-GlcNAc synthase) (EC 2.4.1.-)	*Trabulsiella odontotermitis*		A0A0L0H1H9	--	26162887	Acetyl glucosaminyl transferase activity	Not Available
1788	Haemin storage system, HmsS protein	*Yersinia enterocolitica serotype O:8 / biotype 1B*	Strain NCTC 13174 / 8081	A1JS92	--	17173484	Not Available	Not Available
1789	Poly-beta-1, 6-N-acetyl-D-glucosamine biosynthesis protein PgaD	*Lentisphaeria bacterium*		A0A3D1KDG4	--	30148503	Not Available	Not Available
1790	Poly-beta-1, 6-N-acetyl-D-glucosamine synthase (Poly-beta-1, 6-GlcNAc synthase) (EC 2.4.1.-)	*Acinetobacter baylyi*	Strain ATCC 33305 / BD413 / ADP1	Q6FDD8	--	15514110	Acetyl glucosaminyl transferase activity	Not Available
1791	Poly-beta-1, 6-N-acetyl-D-glucosamine synthase (Poly-beta-1, 6-GlcNAc synthase) (EC 2.4.1.-)	*Candidatus Muproteobacteria bacterium RBG_16_62_13*		A0A1F6T856	--	27774985	Acetyl glucosaminyl transferase activity	Not Available
1792	Motility quorum-sensing regulator MqsR	*Gallionellales bacterium RIFCSPLOWO2_12_FULL_59_22*		A0A1G0EM28	--	27774985	Not Available	Not Available
1793	Outer membrane N-deacetylase	*Trabulsiella odontotermitis*		A0A0L0H2S7	--	26162887	Hydrolase activity, acting on carbon-nitrogen (but not peptide) bonds	Not Available
1794	Haemin storage system, HmsF protein	*Yersinia enterocolitica serotype O:8 / biotype 1B*	Strain NCTC 13174 / 8081	A1JSA0	--	17173484	Hydrolase activity, acting on carbon-nitrogen (but not peptide) bonds	Not Available
1795	Hemin storage protein	*Trabulsiella odontotermitis*		A0A0L0H272	--	26162887	Not Available	Not Available
1796	Surface composition regulator	*Trabulsiella odontotermitis*		A0A0L0GTT1	--	26162887	Not Available	Not Available
1797	Uncharacterized protein	*Gallionellales bacterium RIFCSPLOWO2_12_FULL_59_22*		A0A1G0EIH8	--	27774985	Not Available	Not Available
1798	Poly-beta-1, 6-N-acetyl-D-glucosamine N-deacetylase PgaB	*Lentisphaeria bacterium*		A0A3526	--	30148503	Not Available	Not Available
1799	Poly-beta-1, 6-N-acetyl-D-glucosamine synthase (Poly-beta-1, 6-GlcNAc synthase) (EC 2.4.1.-)	*Lentisphaeria bacterium*		A0A3525	--	30148503	Not Available	Not Available
1800	Poly-beta-1, 6-N-acetyl-D-glucosamine N-deacetylase PgaB	*Lentisphaeria bacterium*		A0A3D1KEA7	--	30148503	Hydrolase activity, acting on carbon-nitrogen (but not peptide) bonds	Not Available
1801	Poly-beta-1, 6-N-acetyl-D-glucosamine biosynthesis protein PgaD	*Lentisphaeria bacterium*		A0A3524	--	30148503	Not Available	Not Available
1802	Poly-beta-1, 6-N-acetyl-D-glucosamine synthase (Poly-beta-1, 6-GlcNAc synthase) (EC 2.4.1.-)	*Lentisphaeria bacterium*		A0A3D1KDU7	--	30148503	Acetyl glucosaminyl transferase activity	Not Available
1803	Poly-beta-1, 6-N-acetyl-D-glucosamine synthase (Poly-beta-1, 6-GlcNAc synthase) (EC 2.4.1.-)	*Chromobacterium haemolyticum*		A0A2T5P4D0	--	29670144	Acetyl glucosaminyl transferase activity	Not Available
1804	Poly-beta-1, 6-N-acetyl-D-glucosamine synthase (Poly-beta-1, 6-GlcNAc synthase) (EC 2.4.1.-)	*Actinobacillus suis H91-0380*		K0GD63	--	23144422	Acetyl glucosaminyl transferase activity	Not Available
1805	Biofilm PGA synthesis lipoprotein PgaB	*Actinobacillus suis H91-0380*		K0G5L0	--	23144422	Hydrolase activity, acting on carbon-nitrogen (but not peptide) bonds	Not Available
1806	Putative inner membrane protein associated with biofilm formation	*Escherichia coli O7:K1*	Strain IAI39 / ExPEC	A0A0H3MI95	--	19165319	Not Available	Not Available

Target ID	Bacterial Protein Name	Organism Name	Strain	UniProt ID	PDB ID	PubMed ID	Classification as per UniProt GO Molecular Function	Classification of Function as per PDB
1807	Uncharacterized protein	*Cupriavidus taiwanensis*	Strain DSM 17343 / BCRC 17206 / CCUG 44338 / CIP 107171 / LMG 19424 / R1 Ralstonia taiwanensis strain LMG 19424	B2AGD9	--	18490699	Not Available	Not Available
1808	Poly-beta-1, 6-N-acetyl-D-glucosamine synthase (Poly-beta-1, 6-GlcNAc synthase) (EC 2.4.1.-)	*Escherichia coli O7:K1*	Strain IAI39 / ExPEC	A0A0H3MLZ7	--	19165319	Acetyl glucosaminyl transferase activity	Not Available
1809	Surface composition regulator	*Yersinia enterocolitica serotype O:8 / biotype 1B*	Strain NCTC 13174 / 8081	A1JPD4	--	17173484	DNA binding, DNA-directed DNA polymerase activity	Not Available
1810	Poly-beta-1, 6-N-acetyl-D-glucosamine synthase (Poly-beta-1, 6-GlcNAc synthase) (EC 2.4.1.-)	*Ralstonia solanaarum CFBP2957*		D8P2G4	--	20550686	Acetyl glucosaminyl transferase activity	Not Available
1811	Putative polysaccharide deacetylase associated with biofilm formation putative lipoprotein (PgaB)	*Ralstonia solanaarum CFBP2957*		D8P2G3	--	20550686	Hydrolase activity, acting on carbon-nitrogen (but not peptide) bonds	Not Available
1812	Uncharacterized protein	*Azospirillum lipoferum*	Strain 4B	G7Z9K0	--	22216014	Not Available	Not Available
1813	Putative intercellular adhesion protein A (Biofilm formation)	*Vibrio alginolyticus*	Strain ATCC 17749 / DSM 2171 / NBRC 15630 / NCIMB 1903 / NCTC 12160 / XII-53	A0A2I3C1P9	--	25635021	Not Available	Not Available
1814	Poly-beta-1, 6-N-acetyl-D-glucosamine synthase (Poly-beta-1, 6-GlcNAc synthase) (EC 2.4.1.-)	*Firmicutes bacterium*		A0A350X1A8	--	30148503	Acetyl glucosaminyl transferase activity	Not Available
1815	Uncharacterized protein	*Firmicutes bacterium*		A0A3D5Z757	--	30148503	Not Available	Not Available
1816	Poly-beta-1, 6-N-acetyl-D-glucosamine synthase (Poly-beta-1, 6-GlcNAc synthase) (EC 2.4.1.-)	*Xanthomonas oryzae pv. oryzae*	Strain PXO99A	A0A0K0GHM1	--	18452608	Acetyl glucosaminyl transferase activity	Not Available
1817	Uncharacterized protein	*Xanthomonas oryzae pv. oryzae*	Strain PXO99A	A0A0K0GHA3	--	18452608	Not Available	Not Available
1818	Uncharacterized protein	*Firmicutes bacterium*		A0A3B8K032	--	30148503	Not Available	Not Available
1819	ABC transporter substrate-binding protein	*Enterobacter asburiae*		A0A0F3XQA0	--	27284137	Not Available	Not Available
1820	Biofilm PGA synthesis protein PgaD	*Escherichia coli O9:H4*	Strain Hs	A0A7I6GYH4	--	18676672	Not Available	Not Available
1821	Toxin-antitoxin system, toxin component	*Lentilactobacillus kisonensis DSM 19906 = JCM 15041*		A0A0R1NQW4	--	26415554	Not Available	Not Available
1822	Poly-beta-1, 6-N-acetyl-D-glucosamine synthase (Poly-beta-1, 6-GlcNAc synthase) (EC 2.4.1.-)	*Escherichia coli O9:H4*	Strain Hs	A0A7I6H0P8	--	18676672	Acetyl glucosaminyl transferase activity	Not Available
1823	Uncharacterized protein	*Escherichia coli O9:H4*	Strain Hs	A0A7I6H8P9	--	18676672	Not Available	Not Available
1824	Outer membrane N-deacetylase	*Enterobacter asburiae*		A0A143I3T0	--	27284137	Hydrolase activity, acting on carbon-nitrogen (but not peptide) bonds	Not Available
1825	Poly-beta-1, 6-N-acetyl-D-glucosamine biosynthesis protein PgaD	*Firmicutes bacterium*		A0A350X1A9	--	30148503	Not Available	Not Available
1826	Uncharacterized protein	*Xanthomonadaceae bacterium*		A0A3C0HHE3	--	30148503	Not Available	Not Available
1827	Poly-beta-1, 6-N-acetyl-D-glucosamine biosynthesis protein PgaD (Fragment)	*Xanthomonadaceae bacterium*		A0A354YBS0	--	30148503	Not Available	Not Available
1828	Poly-beta-1, 6-N-acetyl-D-glucosamine N-deacetylase PgaB	*Xanthomonadaceae bacterium*		A0A354YE77	--	30148503	Hydrolase activity, acting on carbon-nitrogen (but not peptide) bonds	Not Available
1829	Poly-beta-1, 6-N-acetyl-D-glucosamine synthase (Poly-beta-1, 6-GlcNAc synthase) (EC 2.4.1.-)	*Xanthomonadaceae bacterium*		A0A354YDH4	--	30148503	Acetyl glucosaminyl transferase activity	Not Available
1830	Biofilm PGA synthesis lipoprotein pgaB (EC 3.-.-.-)	*Escherichia coli O9:H4*	Strain Hs	A0A7I6H1T4	--	18676672	Hydrolase activity, acting on carbon-nitrogen (but not peptide) bonds	Not Available
1831	Surface composition regulator	*Cronobacter sakazakii 701*		K8CP83	--	23166675	Not Available	Not Available
1832	Poly-beta-1, 6-N-acetyl-D-glucosamine synthase (Poly-beta-1, 6-GlcNAc synthase) (EC 2.4.1.-)	*Enterobacter asburiae*		A0A143I3I2	--	27284137	Acetyl glucosaminyl transferase activity	Not Available
1833	Uncharacterized protein	*Pusillimona spp.*	Strain T7-7	F4GTJ2	--	21622753	Not Available	Not Available
1834	Motility quorum-sensing regulator MqsR	*Betaproteobacteria bacterium RBG_16_56_24*		A0A1F3WWN2	--	27774985	Not Available	Not Available
1835	Uncharacterized protein	*Rahnella spp.*	Strain Y9602	A0A0H3FP36	--	22461551	Not Available	Not Available
1836	Poly-beta-1, 6-N-acetyl-D-glucosamine synthase (Poly-beta-1, 6-GlcNAc synthase) (EC 2.4.1.-)	*Pusillimona spp.*	Strain T7-7	F4GTJ1	--	21622753	Acetyl glucosaminyl transferase activity	Not Available
1837	Polysaccharide deacetylase	*Pusillimona spp.*	Strain T7-7	F4GTJ0	--	21622753	Hydrolase activity, acting on carbon-nitrogen (but not peptide) bonds	Not Available
1838	Type II toxin-antitoxin system MqsR family toxin	*Firmicutes bacterium*		A0A7C7D9P2	--	32123542	Not Available	Not Available
1839	Poly-beta-1, 6-N-acetyl-D-glucosamine synthase (Poly-beta-1, 6-GlcNAc synthase) (EC 2.4.1.-)	*Xanthomonadaceae bacterium SCN 69-123*		A0A1E4L167	--	26031303	Acetyl glucosaminyl transferase activity	Not Available
1840	Poly-beta-1, 6-N-acetyl-D-glucosamine synthase (Poly-beta-1, 6-GlcNAc synthase) (EC 2.4.1.-) (Fragment)	*Alphaproteobacteria bacterium*		A0A537MT94	--	31110364	Acetyl glucosaminyl transferase activity	Not Available
1841	Surface composition regulator	*Salmonella enterica subspp. indica serovar*	6, 14, 25:z10:1, 2, 7 Str. 1121	V1H6C0	--	24158624	Not Available	Not Available
1842	Poly-beta-1, 6-N-acetyl-D-glucosamine N-deacetylase PgaB	*Chromatiales bacterium*		A0A7C1WNC0	--	31911466	Hydrolase activity, acting on carbon-nitrogen (but not peptide) bonds	Not Available
1843	Poly-beta-1, 6-N-acetyl-D-glucosamine synthase (Poly-beta-1, 6-GlcNAc synthase) (EC 2.4.1.-) (Fragment)	*Chromatiales bacterium*		A0A7C2AD10	--	31911466	Acetyl glucosaminyl transferase activity	Not Available
1844	Response receiver-modulated diguanylate cyclase	*Geobacter sulfurreducens*	Strain ATCC 51573 / DSM 12127 / PCA	Q746Z1	--	14671304	Diguanylate cyclase activity	Not Available
1845	Poly-beta-1, 6-N-acetyl-D-glucosamine synthase (Poly-beta-1, 6-GlcNAc synthase) (EC 2.4.1.-)	*Xanthomonas gardneri ATCC 19865*		F0CCM8	--	21396108	Acetyl glucosaminyl transferase activity	Not Available
1846	Response receiver-modulated diguanylate cyclase	*Geobacter sulfurreducens*	Strain ATCC 51573 / DSM 12127 / PCA	Q74AN9	--	14671304	Diguanylate cyclase activity	Not Available
1847	Sensor diguanylate cyclase, GAF domain-containing	*Geobacter sulfurreducens*	Strain ATCC 51573 / DSM 12127 / PCA	Q74ER5	--	14671304	Diguanylate cyclase activity	Not Available
1848	Response receiver-modulated diguanylate cyclase	*Geobacter sulfurreducens*	Strain ATCC 51573 / DSM 12127 / PCA	Q74CL4	--	14671304	Diguanylate cyclase activity	Not Available
1849	Sensor diguanylate cyclase, GAF domain-containing	*Geobacter sulfurreducens*	Strain ATCC 51573 / DSM 12127 / PCA	Q74C06	--	14671304	Diguanylate cyclase activity	Not Available
1850	Poly-beta-1, 6-N-acetyl-D-glucosamine synthase (Poly-beta-1, 6-GlcNAc synthase) (EC 2.4.1.-)	*Sporolactobacillus laevolacticus DSM 442*		V6J0L8	--	24371202	Acetyl glucosaminyl transferase activity	Not Available
1851	Surface composition regulator	*Salmonella enterica subspp. indica serovar*	6, 14, 25:z10:1, 2, 7 Str. 1121	V1GMT8	--	24158624	Not Available	Not Available
1852	Diguanylate cyclase, HAMP domain-containing	*Geobacter sulfurreducens*	Strain ATCC 51573 / DSM 12127 / PCA	Q747B1	--	14671304	Diguanylate cyclase activity	Not Available
1853	Diguanylate cyclase, CAP_ED domain-containing	*Geobacter sulfurreducens*	Strain ATCC 51573 / DSM 12127 / PCA	Q74F01	--	14671304	Diguanylate cyclase activity	Not Available
1854	Diguanylate cyclase	*Geobacter sulfurreducens*	Strain ATCC 51573 / DSM 12127 / PCA	Q74CW7	--	14671304	Diguanylate cyclase activity	Not Available
1855	Uncharacterized protein	*Xanthomonas gardneri ATCC 19865*		F0CCM7	--	21396108	Not Available	Not Available
1856	Hemin storage system protein	*Enterobacter spp. Ag1*		J1GD16	--	22965099	Not Available	Not Available
1857	Putative transmembrane protein	*Ralstonia solanacearum FQY_4*		M4UJ22	--	23661471	Not Available	Not Available

Target ID	Bacterial Protein Name	Organism Name	Strain	UniProt ID	PDB ID	PubMed ID	Classification as per UniProt GO Molecular Function	Classification of Function as per PDB
1858	Biofilm PGA synthesis deacetylase PgaB (EC 3.-.-.-)	*Ralstonia solanacearum FQY_4*		M4ULZ0	--	23661471	Hydrolase activity, acting on carbon-nitrogen (but not peptide) bonds	Not Available
1859	Toxin-antitoxin system, toxin component	*Candidatus melainabacteria bacterium GWF2_37_15*		A0A1F6PUW9	--	27774985	Not Available	Not Available
1860	Poly-beta-1, 6-N-acetyl-D-glucosamine synthase (Poly-beta-1, 6-GlcNAc synthase) (EC 2.4.1.-)	*Enterobacter spp. Ag1*		J0M9E9	--	22965099	Acetyl glucosaminyl transferase activity	Not Available
1861	Outer membrane N-deacetylase	*Enterobacter spp. Ag1*		J1GD54	--	22965099	Hydrolase activity, acting on carbon-nitrogen (but not peptide) bonds	Not Available
1862	Uncharacterized protein	*Caldanaerobacter subterraneus*		A0A357VJY1	--	30148503	Not Available	Not Available
1863	PleD	*Synechocystis spp.*	Strain PCC 6803 / Kazusa	P73011	--	8905231	Diguanylate cyclase activity	Not Available
1864	Surface composition regulator	*Salmonella typhimurium*	Strain ATCC 68169 / UK-1	A0A3Z6P281	--	21622747	Not Available	Not Available
1865	Sll0779 protein	*Synechocystis spp.*	Strain PCC 6803 / Kazusa	Q55955	--	8905231	Diguanylate cyclase activity	Not Available
1866	Regulatory components of sensory transduction system	*Synechocystis spp.*	Strain PCC 6803 / Kazusa	P72790	--	8905231	Diguanylate cyclase activity	Not Available
1867	Uncharacterized protein	*Cupriavidus spp. GA3-3*		R7XF68	--	23833131	Not Available	Not Available
1868	Slr0302 protein	*Synechocystis spp.*	Strain PCC 6803 / Kazusa	Q55906	--	8905231	Diguanylate cyclase activity	Not Available
1869	Slr0829 protein	*Synechocystis spp.*	Strain PCC 6803 / Kazusa	Q55414	--	8905231	Diguanylate cyclase activity	Not Available
1870	Uncharacterized protein	*Candidatus Rokubacteria bacterium RIFCSPLOWO2_12_FULL_71_19*		A0A1F7PI81	--	27774985	Not Available	Not Available
1871	Poly-beta-1, 6-N-acetyl-D-glucosamine biosynthesis protein PgaD	*Chromatiales bacterium*		A0A7C2B5U1	--	31911466	Not Available	Not Available
1872	Regulatory components of sensory transduction system	*Synechocystis spp.*	Strain PCC 6803 / Kazusa	P73036	--	8905231	Diguanylate cyclase activity	Not Available
1873	Putative bacteriophage related protein	*Lactobacillus acidophilus*	Strain ATCC 700396 / NCK56 / N2 / NCFM	Q5FJC7	--	15671160	Not Available	Not Available
1874	PleD gene product	*Synechocystis spp.*	Strain PCC 6803 / Kazusa	P74658	--	8905231	Diguanylate cyclase activity	Not Available
1875	Poly-beta-1, 6-N-acetyl-D-glucosamine synthase (Poly-beta-1, 6-GlcNAc synthase) (EC 2.4.1.-)	*Ralstonia solanacearum FQY_4*		M4UIL5	--	23661471	Acetyl glucosaminyl transferase activity	Not Available
1876	Uncharacterized protein	*Mammaliicoccus lentus*	Staphylococcus lentus	A0A178PEY6	--	27375578	Not Available	Not Available
1877	Poly-beta-1, 6-N-acetyl-D-glucosamine synthase (Poly-beta-1, 6-GlcNAc synthase) (EC 2.4.1.-)	*Achromobacter spp. AONIH1*		A0A2I8DJJ4	--	29437920	Acetyl glucosaminyl transferase activity	Not Available
1878	Poly-beta-1, 6-N-acetyl-D-glucosamine biosynthesis protein PgaD	*Acinetobacter spp. COS3*		U7GZZ6	--	24356826	Not Available	Not Available
1879	Surface composition regulator	*Yersinia pseudotuberculosis serotype O:1b*	Strain IP 31758	A0A0U1R214	--	17784789	DNA binding, DNA-directed DNA polymerase activity	Not Available
1880	Poly-beta-1, 6-N-acetyl-D-glucosamine N-deacetylase PgaB	*Acinetobacter baumannii*	Strain MDR-ZJ06	A0A654L1R9	--	21788470	Hydrolase activity, acting on carbon-nitrogen (but not peptide) bonds	Not Available
1881	Putative biofilm PGA synthesis protein PgaD	*Yersinia pseudotuberculosis serotype O:1b*	Strain IP 31758	A0A0U1QUY6	--	17784789	Not Available	Not Available
1882	Type II toxin-antitoxin system MqsR family toxin	*Pseudomonas spp. MPC6*		A0A5B7VRQ1	--	31178851; 31089984	Not Available	Not Available
1883	Poly-beta-1, 6-N-acetyl-D-glucosamine synthase (Poly-beta-1, 6-GlcNAc synthase) (EC 2.4.1.-)	*Yersinia pseudotuberculosis serotype O:1b*	Strain IP 31758	A0A0U1QVU7	--	17784789	Acetyl glucosaminyl transferase activity	Not Available
1884	Poly-beta-1, 6-N-acetyl-D-glucosamine N-deacetylase PgaB	*Paraburkholderia phytofirmans OLGA172*		A0A160FHA6	--	27063562	Hydrolase activity, acting on carbon-nitrogen (but not peptide) bonds	Not Available
1885	Poly-beta-1, 6-N-acetyl-D-glucosamine N-deacetylase	*Serratia marcescens*		A0A221DT18	--	28536292	Hydrolase activity, acting on carbon-nitrogen (but not peptide) bonds	Not Available
1886	Uncharacterized protein	*Thioalkalivibrio sulfidiphilus*	Strain HL-EbGR7	B8GT53	--	21475584	Not Available	Not Available
1887	Toxin	*Desulfobacteraceae bacterium*		A0A3N5RWQ1	--	30086797	Not Available	Not Available
1888	Poly-beta-1, 6-N-acetyl-D-glucosamine biosynthesis protein PgaD	*Variovorax spp. SCN 67-85*		A0A1E4IRH5	--	26031303	Not Available	Not Available
1889	Uncharacterized protein	*Acinetobacter venetianus*		A0A150HS68	--	26902269	Not Available	Not Available
1890	GGDEF domain-containing protein	*Aquifex aeolicus*	Strain VF5	O66743	--	9537320	Diguanylate cyclase activity	Not Available
1891	Poly-beta-1, 6-N-acetyl-D-glucosamine N-deacetylase PgaB	*Variovorax spp. SCN 67-85*		A0A1E4IR53	--	26031303	Hydrolase activity, acting on carbon-nitrogen (but not peptide) bonds	Not Available
1892	GGDEF domain-containing protein	*Aquifex aeolicus*	Strain VF5	O66912	--	9537320	Diguanylate cyclase activity	Not Available
1893	Poly-beta-1, 6-N-acetyl-D-glucosamine synthase (Poly-beta-1, 6-GlcNAc synthase) (EC 2.4.1.-)	*Variovorax spp. SCN 67-85*		A0A1E4IR81	--	26031303	Acetyl glucosaminyl transferase activity	Not Available
1894	Poly-beta-1, 6-N-acetyl-D-glucosamine synthase (Poly-beta-1, 6-GlcNAc synthase) (EC 2.4.1.-)	*Variovorax spp. CF313*		J3CAZ9	--	23045501	Acetyl glucosaminyl transferase activity	Not Available
1895	GGDEF domain-containing protein	*Aquifex aeolicus*	Strain VF5	O66623	--	9537320	Diguanylate cyclase activity	Not Available
1896	Hemin storage protein	*Thioalkalivibrio sulfidiphilus*	Strain HL-EbGR7	B8GTH8	--	21475584	Hydrolase activity, acting on carbon-nitrogen (but not peptide) bonds	Not Available
1897	Poly-beta-1, 6-N-acetyl-D-glucosamine N-deacetylase PgaB	*Serratia marcescens*		A0A7D6C0J8	--	21967170	Hydrolase activity, acting on carbon-nitrogen (but not peptide) bonds	Not Available
1898	Putative hemin storage signal peptide protein	*Ralstonia solanacearum*	Strain GMI1000 Pseudomonas solanacearum	Q8XT28	--	11823852	Hydrolase activity, acting on carbon-nitrogen (but not peptide) bonds	Not Available
1899	Poly-beta-1, 6-N-acetyl-D-glucosamine synthase (Poly-beta-1, 6-GlcNAc synthase) (EC 2.4.1.-)	*Desulfobacteraceae bacterium*		A0A3A4RX93	--	28644444	Acetyl glucosaminyl transferase activity	Not Available
1900	Poly-beta-1, 6-N-acetyl-D-glucosamine synthase (Poly-beta-1, 6-GlcNAc synthase) (EC 2.4.1.-)	*Pseudomonas spp. FH1*		W2DS81	--	24286439	Acetyl glucosaminyl transferase activity	Not Available
1901	Uncharacterized protein	*Marine gamma proteobacterium HTCC2143*		A0YHD9	--	20601481	Not Available	Not Available
1902	Uncharacterized protein	*Desulfobacteraceae bacterium*		A0A7C1DMQ6	--	31911466	Not Available	Not Available
1903	Poly-beta-1, 6-N-acetyl-D-glucosamine synthase (Poly-beta-1, 6-GlcNAc synthase) (EC 2.4.1.-)	*Syntrophotalea carbinolica*	Strain DSM 2380 / NBRC 103641 / GraBd1 Pseudomonas solanacearum	Q3A0T2	--	23227809	Acetyl glucosaminyl transferase activity	Not Available
1904	PgaD	*Syntrophotalea carbinolica*	Strain DSM 2380 / NBRC 103641 / GraBd1 Pseudomonas solanacearum	Q3A0T3	--	23227809	Not Available	Not Available

Target ID	Bacterial Protein Name	Organism Name	Strain	UniProt ID	PDB ID	PubMed ID	Classification as per UniProt GO Molecular Function	Classification of Function as per PDB
1905	Uncharacterized protein	*Pseudomonas spp. FH1*		W2D7M0	--	24286439	Not Available	Not Available
1906	Type II toxin-antitoxin system MqsR family toxin	*Xylella fastidiosa subspp. multiplex*		A0A6I2KF74	--	31704683	Not Available	Not Available
1907	Outer membrane N-deacetylase	*Pseudomonas spp. FH1*		W2DUA0	--	24286439	Hydrolase activity, acting on carbon-nitrogen (but not peptide) bonds	Not Available
1908	Uncharacterized protein	*Pseudomonas spp. FH1*		W2DS47	--	24286439	Not Available	Not Available
1909	Uncharacterized protein	*Nitrospirae bacterium RBG_16_64_22*		A0A1G1HY41	--	27774985	Not Available	Not Available
1910	Outer membrane polysaccharide N-deacetylase	*Syntrophotalea carbinolica*	Strain DSM 2380 / NBRC 103641 / GraBd1 Pseudomonas solanacearum	Q3A0T1	--	23227809	Hydrolase activity, acting on carbon-nitrogen (but not peptide) bonds	Not Available
1911	Surface composition regulator	*Edwardsiella anguillarum ET080813*		A0A076LXT7	--	22590641	Not Available	Not Available
1912	Uncharacterized protein	*Spirochaetae bacterium HGW-Spirochaetae-9*		A0A2N1R0F0	--	28350393	Not Available	Not Available
1913	Uncharacterized protein	*Pseudomonas spp. GM24*		J3FKT9	--	23045501	Not Available	Not Available
1914	Uncharacterized protein	*Deltaproteobacteria bacterium RIFOXYD12_FULL_55_16*		A0A1F9MB73	--	27774985	Not Available	Not Available
1915	Probable transmembrane protein	*Ralstonia solanacearum*	Strain GMI1000 Pseudomonas solanacearum	Q8XT26	--	11823852	Not Available	Not Available
1916	Poly-beta-1, 6-N-acetyl-D-glucosamine synthase (Poly-beta-1, 6-GlcNAc synthase) (EC 2.4.1.-)	*Pseudomonadales bacterium RIFCSPLOWO2_12_FULL_59_450*		A0A1G3F5W8	--	27774985	Acetyl glucosaminyl transferase activity	Not Available
1917	Surface composition regulator	*Salmonella enterica subspp. enterica serovar Inverness*	Str. R8-3668	G5NIA2	--	21859443	Not Available	Not Available
1918	Poly-beta-1, 6-N-acetyl-D-glucosamine synthase (Poly-beta-1, 6-GlcNAc synthase) (EC 2.4.1.-)	*Citrobacter freundii complex spp. CFNIH9*		A0A2I8THL0	--	29437920	Acetyl glucosaminyl transferase activity	Not Available
1919	Poly-beta-1, 6-N-acetyl-D-glucosamine N-deacetylase PgaB	*Citrobacter freundii complex spp. CFNIH9*		A0A2I8TH49	--	29437920	Hydrolase activity, acting on carbon-nitrogen (but not peptide) bonds	Not Available
1920	Uncharacterized protein	*Sporolactobacillus laevolacticus DSM 442*		V6IZ98	--	24371202	Not Available	Not Available
1921	Putative xylanase / chitin deacetylase	*Xanthomonas gardneri ATCC 19865*		F0CCM9	--	21396108	Hydrolase activity, acting on carbon-nitrogen (but not peptide) bonds, Hydrolase activity, acting on glycosyl bonds	Not Available
1922	Nucleotide cyclase, HAMP and GGDEF-related domain-containing	*Geobacter sulfurreducens*	Strain ATCC 51573 / DSM 12127 / PCA	Q74BT9	--	14671304	Diguanylate cyclase activity	Not Available
1923	Poly-beta-1, 6-N-acetyl-D-glucosamine biosynthesis protein PgaD	*Citrobacter freundii complex spp. CFNIH9*		A0A2I8THC0	--	29437920	Not Available	Not Available
1924	Surface composition regulator	*Serratia spp. 14-2641*		A0A1C7WFU5	--	27547538	Not Available	Not Available
1925	Poly-beta-1, 6-N-acetyl-D-glucosamine N-deacetylase	*Pseudomonadales bacterium RIFCSPLOWO2_12_FULL_59_450*		A0A1G3F604	--	27774985	Hydrolase activity, acting on carbon-nitrogen (but not peptide) bonds	Not Available
1926	Surface composition regulator	*Citrobacter freundii complex spp. CFNIH9*		A0A2I8TGS9	--	29437920	Not Available	Not Available
1927	Motility quorum-sensing regulator MqsR	*Pseudomonadales bacterium RIFCSPLOWO2_12_FULL_59_450*		A0A1G3FBL9	--	27774985	Not Available	Not Available
1928	Type II toxin-antitoxin system MqsR family toxin	*Mesorhizobium huakuii*		A0A7G6T3G4	--	32686981	Not Available	Not Available
1929	Poly-beta-1, 6-N-acetyl-D-glucosamine biosynthesis protein PgaD	*Pseudomonadales bacterium RIFCSPLOWO2_12_FULL_59_450*		A0A1G3F5W0	--	27774985	Not Available	Not Available
1930	Poly-beta-1, 6-N-acetyl-D-glucosamine biosynthesis protein PgaD	*Acinetobacter baumannii*	Strain MDR-ZJ06	A0A654L1G9	--	21788470	Not Available	Not Available
1931	Uncharacterized protein	*Yersinia pseudotuberculosis serotype O:1b*	Strain IP 31758	A0A0U1R210	--	17784789	Not Available	Not Available
1932	Poly-beta-1, 6-N-acetyl-D-glucosamine synthase (Poly-beta-1, 6-GlcNAc synthase) (EC 2.4.1.-)	*Acinetobacter spp. COS3*		U7H247	--	24356826	Acetyl glucosaminyl transferase activity	Not Available
1933	Poly-beta-1, 6-N-acetyl-D-glucosamine synthase (Poly-beta-1, 6-GlcNAc synthase) (EC 2.4.1.-)	*Lysobacter capsici AZ78*		A0A125U042	--	24762937	Acetyl glucosaminyl transferase activity	Not Available
1934	Poly-beta-1, 6-N-acetyl-D-glucosamine N-deacetylase PgaB	*Acinetobacter baumannii*	Strain MDR-ZJ06	A0A654KXD6	--	21788470	Hydrolase activity, acting on carbon-nitrogen (but not peptide) bonds	Not Available
1935	Poly-beta-1, 6-N-acetyl-D-glucosamine N-deacetylase	*Achromobacter spp. AONIH1*		A0A2I8DLA9	--	29437920	Hydrolase activity, acting on carbon-nitrogen (but not peptide) bonds	Not Available
1936	Biofilm PGA synthesis deacetylase PgaB	*Lysobacter capsici AZ78*		A0A125U043	--	24762937	Hydrolase activity, acting on carbon-nitrogen (but not peptide) bonds	Not Available
1937	Biofilm PGA synthesis lipoprotein PgaB (EC 3.-.-.-)	*Yersinia pseudotuberculosis serotype O:1b*	Strain IP 31758	A0A0U1R215	--	17784789	Hydrolase activity, acting on carbon-nitrogen (but not peptide) bonds	Not Available
1938	Polysaccharide deacetylase	*Acinetobacter spp. COS3*		U7GXQ6	--	24356826	Hydrolase activity, acting on carbon-nitrogen (but not peptide) bonds	Not Available
1939	Poly-beta-1, 6-N-acetyl-D-glucosamine biosynthesis protein PgaD	*Achromobacter spp. AONIH1*		A0A2I8DK81	--	29437920	Not Available	Not Available
1940	Surface composition regulator	*Salmonella enterica subspp. enterica serovar*	Cubana Str. 76814	V7IJA1	--	24459266	Not Available	Not Available
1941	Ferric siderophore transport system, periplasmic binding protein TonB	*Lysobacter capsici AZ78*		A0A125U041	--	24762937	Not Available	Not Available
1942	Surface composition regulator	*Salmonella enterica subspp. enterica serovar*	Virchow Str. SL491	A0A6C8EY32	--	21602358	Not Available	Not Available
1943	Poly-beta-1, 6-N-acetyl-D-glucosamine N-deacetylase PgaB	*Pseudomonas spp. MPC6*		A0A5B7VT08	--	31178851; 31089984	Hydrolase activity, acting on carbon-nitrogen (but not peptide) bonds	Not Available
1944	Uncharacterized protein	*Xylella fastidiosa*	Strain 9a5c	Q9PAL4	--	10910347	Not Available	Not Available
1945	Poly-beta-1, 6-N-acetyl-D-glucosamine biosynthesis protein PgaD	*Pseudomonas spp. MPC6*		A0A5B7VSW4	--	31178851; 31089984	Not Available	Not Available
1946	Type II toxin-antitoxin system MqsR family toxin	*Pseudomonas spp. MPC6*		A0A5B7W3T4	--	31178851; 31089984	Not Available	Not Available
1947	Surface composition regulator	*Yersinia pestis bv. Antiqua*	Strain Nepal516	A0A0H2YFC4	--	16740952	DNA binding, DNA-directed DNA polymerase activity	Not Available

Target ID	Bacterial Protein Name	Organism Name	Strain	UniProt ID	PDB ID	PubMed ID	Classification as per UniProt GO Molecular Function	Classification of Function as per PDB
1948	Lmo2174 protein	*Listeria monocytogenes serovar 1 / 2a*	Strain ATCC BAA-679 / EGD-e	Q8Y595	--	11679669	Diguanylate cyclase activity, phosphorelay sensor kinase activity	Not Available
1949	Biofilm PGA synthesis deacetylase PgaB	*Pseudomonas yamanorum*		A0A145XGS6	--	27231373	Hydrolase activity, acting on carbon-nitrogen (but not peptide) bonds	Not Available
1950	Lmo1911 protein	*Listeria monocytogenes serovar 1 / 2a*	Strain ATCC BAA-679 / EGD-e	Q8Y5Z2	--	11679669	Diguanylate cyclase activity	Not Available
1951	Uncharacterized protein	*Yersinia pestis bv. Antiqua*	Strain Nepal516	A0A0H2YLS5	--	16740952	Not Available	Not Available
1952	Lmo1912 protein	*Listeria monocytogenes serovar 1 / 2a*	Strain ATCC BAA-679 / EGD-e	Q8Y5Z1	--	11679669	Diguanylate cyclase activity, phosphorelay sensor kinase activity	Not Available
1953	Poly-beta-1, 6-N-acetyl-D-glucosamine synthase (Poly-beta-1, 6-GlcNAc synthase) (EC 2.4.1.-)	*Tissierellia bacterium*		A0A7X8I1F8	--	32123542	Acetyl glucosaminyl transferase activity	Not Available
1954	Poly-beta-1, 6-N-acetyl-D-glucosamine synthase (Poly-beta-1, 6-GlcNAc synthase) (EC 2.4.1.-)	*Pseudomonas spp. MPC6*		A0A5B7VSI7	--	31178851; 31089984	Acetyl glucosaminyl transferase activity	Not Available
1955	Poly-beta-1, 6-N-acetyl-D-glucosamine biosynthesis protein PgaD	*Xenorhabdus ishibashii*		A0A2D0KJM4	--	28993611	Not Available	Not Available
1956	Poly-beta-1, 6-N-acetyl-D-glucosamine N-deacetylase PgaB	*Pseudomonas nabeulensis*		A0A4Z0APQ9	--	30910423	Hydrolase activity, acting on carbon-nitrogen (but not peptide) bonds	Not Available
1957	Poly-beta-1, 6-N-acetyl-D-glucosamine synthase (Poly-beta-1, 6-GlcNAc synthase) (EC 2.4.1.-)	*Xenorhabdus ishibashii*		A0A2D0KJN1	--	28993611	Acetyl glucosaminyl transferase activity	Not Available
1958	Poly-beta-1, 6-N-acetyl-D-glucosamine synthase (Poly-beta-1, 6-GlcNAc synthase) (EC 2.4.1.-)	*Acinetobacter baumannii*	Strain MDR-ZJ06	A0A654KXG3	--	21788470	Acetyl glucosaminyl transferase activity	Not Available
1959	Poly-beta-1, 6-N-acetyl-D-glucosamine biosynthesis protein PgaD	*Acinetobacter baumannii*	Strain MDR-ZJ06	A0A654KXA5	--	21788470	Not Available	Not Available
1960	Poly-beta-1, 6-N-acetyl-D-glucosamine synthase (Poly-beta-1, 6-GlcNAc synthase) (EC 2.4.1.-)	*Acinetobacter baumannii*	Strain MDR-ZJ06	A0A654L1A0	--	21788470	Acetyl glucosaminyl transferase activity	Not Available
1961	Polysaccharide deacetylase	*Xenorhabdus ishibashii*		A0A2D0KK07	--	28993611	Hydrolase activity, acting on carbon-nitrogen (but not peptide) bonds	Not Available
1962	Motility quorum-sensing regulator MqsR	*Pseudomonas spp. Os17*		A0A0D6BDD9	--	25496595	Not Available	Not Available
1963	Putative biofilm PGA synthesis protein PgaD	*Pseudomonas fluorescens*	Strain ATCC BAA-477 / NRRL B-23932 / Pf-5	Q4KKC2	--	15980861	Not Available	Not Available
1964	Poly-beta-1, 6-N-acetyl-D-glucosamine synthase (Poly-beta-1, 6-GlcNAc synthase) (EC 2.4.1.-)	*Actinobacillus pleuropneumoniae serotype 5b*	Strain L20	A3N3L7	--	18065534	Acetyl glucosaminyl transferase activity	Not Available
1965	Poly-beta-1, 6-N-acetyl-D-glucosamine biosynthesis protein PgaD	*Pseudomonas fuscovaginae*		A0A0M9GIL7	--	26422147	Not Available	Not Available
1966	Poly-beta-1, 6-N-acetyl-D-glucosamine synthase (Poly-beta-1, 6-GlcNAc synthase) (EC 2.4.1.-)	*Enterobacter hormaechei*		A0A6G3F3P9	--	31477907	Acetyl glucosaminyl transferase activity	Not Available
1967	Uncharacterized protein	*Alphaproteobacteria bacterium HGW-Alphaproteobacteria-3*		A0A2N3BZZ5	--	28350393	Not Available	Not Available
1968	Poly-beta-1, 6-N-acetyl-D-glucosamine biosynthesis protein PgaD (Fragment)	*Acinetobacter ursingii*		A0A3D2SJF3	--	30148503	Not Available	Not Available
1969	Poly-beta-1, 6-N-acetyl-D-glucosamine biosynthesis protein PgaD	*Xanthomonadaceae bacterium SCN 69-123*		A0A1E4L188	--	26031303	Not Available	Not Available
1970	Outer membrane N-deacetylase	*Achromobacter marplatensis*		J4P4T1	--	23105084	Hydrolase activity, acting on carbon-nitrogen (but not peptide) bonds	Not Available
1971	Surface composition regulator	*Serratia fonticola AU-P3*	3	U2LWW9	--	24233592	Not Available	Not Available
1972	Surface composition regulator	*Enterobacteriaceae bacterium*	Strain FGI 57	L0LYH9	--	23469353	Not Available	Not Available
1973	Poly-beta-1, 6-N-acetyl-D-glucosamine biosynthesis protein PgaD	*Escherichia coli*		A0A5P0JEL1	--	31642700	Not Available	Not Available
1974	Uncharacterized protein	*Pluralibacter gergoviae*	Enterobacter gergoviae	A0A1S2B9G4	--	25502672	Not Available	Not Available
1975	Surface composition regulator	*Cronobacter sakazakii*	Strain ATCC BAA-894 Enterobacter sakazakii	A7MF81	--	20221447	Not Available	Not Available
1976	Surface composition regulator	*Escherichia coli*		A0A5D1FAL4	--	31477907	Not Available	Not Available
1977	Poly-beta-1, 6-N-acetyl-D-glucosamine synthase (Poly-beta-1, 6-GlcNAc synthase) (EC 2.4.1.-)	*Escherichia coli*		A0A246NPP4	--	30286803	Acetyl glucosaminyl transferase activity	Not Available
1978	Surface composition regulator	*Pectobacterium polonicum*		A0A4U7KK94	--	30973319	DNA binding, DNA-directed DNA polymerase activity	Not Available
1979	Poly-beta-1, 6-N-acetyl-D-glucosamine synthase (Poly-beta-1, 6-GlcNAc synthase) (EC 2.4.1.-)	*Macrococcus epidermidis*		A0A328A136	--	29951040	Acetyl glucosaminyl transferase activity	Not Available
1980	Surface composition regulator	*Escherichia coli*		A0A6M0PTC6	--	16943019	Not Available	Not Available
1981	Poly-beta-1, 6-N-acetyl-D-glucosamine synthase (Poly-beta-1, 6-GlcNAc synthase) (EC 2.4.1.-)	*Escherichia coli*		A0A6M0PSJ4	--	16943019	Acetyl glucosaminyl transferase activity	Not Available
1982	Lipoprotein (HmsF protein of hmsHFRS Operon)	*Xenorhabdus nematophila*	Strain ATCC 19061 / DSM 3370 / CCUG 14189 / LMG 1036 / NCIMB 9965 / AN6	D3VJ37	--	22125637	Hydrolase activity, acting on carbon-nitrogen (but not peptide) bonds	Not Available
1983	Poly-beta-1, 6-N-acetyl-D-glucosamine synthase (Poly-beta-1, 6-GlcNAc synthase) (EC 2.4.1.-)	*Xenorhabdus nematophila*	Strain ATCC 19061 / DSM 3370 / CCUG 14189 / LMG 1036 / NCIMB 9965 / AN6	D3VJ36	--	22125637	Acetyl glucosaminyl transferase activity	Not Available
1984	Poly-beta-1, 6-N-acetyl-D-glucosamine N-deacetylase PgaB	*Xanthomonadaceae bacterium SCN 69-123*		A0A1E4L199	--	26031303	Hydrolase activity, acting on carbon-nitrogen (but not peptide) bonds	Not Available
1985	Haemin storage system (HmsS protein of hmsHFRS Operon)	*Xenorhabdus nematophila*	Strain ATCC 19061 / DSM 3370 / CCUG 14189 / LMG 1036 / NCIMB 9965 / AN6	D3VJ35	--	22125637	Not Available	Not Available
1986	Poly-beta-1, 6-N-acetyl-D-glucosamine synthase (Poly-beta-1, 6-GlcNAc synthase) (EC 2.4.1.-)	*Escherichia coli*		A0A792HM93	--	30286803	Acetyl glucosaminyl transferase activity	Not Available
1987	Poly-beta-1, 6-N-acetyl-D-glucosamine biosynthesis protein PgaD	*Pectobacterium polonicum*		A0A4U7KCH9	--	30973319	Not Available	Not Available
1988	Poly-beta-1, 6-N-acetyl-D-glucosamine biosynthesis protein PgaD	*Escherichia coli*		A0A5P0J1W1	--	31642700	Not Available	Not Available
1989	mRNA interferase MqsR	*Escherichia coli*		A0A0P7PX62	--	26441869	Not Available	Not Available
1990	Poly-beta-1, 6-N-acetyl-D-glucosamine biosynthesis protein PgaD	*Clostridium spp. Maddingley MBC34-26*		K6T4C8	--	23405323	Not Available	Not Available
1991	Poly-beta-1, 6-N-acetyl-D-glucosamine synthase (Poly-beta-1, 6-GlcNAc synthase) (EC 2.4.1.-)	*Pectobacterium polonicum*		A0A4U7KDL9	--	30973319	Acetyl glucosaminyl transferase activity	Not Available
1992	Poly-beta-1, 6-N-acetyl-D-glucosamine N-deacetylase PgaB (EC 3.5.1.-)	*Escherichia coli*		A0A7B3MEP4	--	30286803	Hydrolase activity, acting on carbon-nitrogen (but not peptide) bonds	Not Available
1993	Poly-beta-1, 6-N-acetyl-D-glucosamine synthase (Poly-beta-1, 6-GlcNAc synthase) (EC 2.4.1.-)	*Escherichia coli*		A0A5P0JF91	--	31642700	Acetyl glucosaminyl transferase activity	Not Available
1994	Poly-beta-1, 6-N-acetyl-D-glucosamine synthase (Poly-beta-1, 6-GlcNAc synthase) (EC 2.4.1.-)	*Clostridium spp. Maddingley MBC34-26*		K6U9Y4	--	23405323	Acetyl glucosaminyl transferase activity	Not Available

Target ID	Bacterial Protein Name	Organism Name	Strain	UniProt ID	PDB ID	PubMed ID	Classification as per UniProt GO Molecular Function	Classification of Function as per PDB
1995	Poly-beta-1, 6-N-acetyl-D-glucosamine N-deacetylase PgaB	*Escherichia coli*		A0A5P0JEP5	--	31642700	Hydrolase activity, acting on carbon-nitrogen (but not peptide) bonds	Not Available
1996	Poly-beta-1, 6-N-acetyl-D-glucosamine N-deacetylase PgaB	*Escherichia coli*		A0A6M0PSS0	--	16943019	Hydrolase activity, acting on carbon-nitrogen (but not peptide) bonds	Not Available
1997	Sll1687 protein	*Synechocystis spp.*	Strain PCC 6803 / Kazusa	P73713	--	8905231	Diguanylate cyclase activity	Not Available
1998	Poly-beta-1, 6-N-acetyl-D-glucosamine synthase (Poly-beta-1, 6-GlcNAc synthase) (EC 2.4.1.-)	*Halomonas spp. HAL1*		G4F1K5	--	22156396	Acetyl glucosaminyl transferase activity	Not Available
1999	Xylanae / chitin deacetylase	*Halomonas spp. HAL1*		G4F1K6	--	22156396	Hydrolase activity, acting on carbon-nitrogen (but not peptide) bonds, Hydrolase activity, acting on glycosyl bonds	Not Available
2000	Diguanylate cyclase (EC 2.7.7.65)	*Shewanella oneidensis*	Strain MR-1	Q8EF08	--	12368813	Diguanylate cyclase activity	Not Available
2001	Diguanylate cyclase (EC 2.7.7.65)	*Shewanella oneidensis*	Strain MR-1	Q8EFD2	--	12368813	Diguanylate cyclase activity	Not Available
2002	Diguanylate cyclase with PAS sensory domain (EC 2.7.7.65)	*Shewanella oneidensis*	Strain MR-1	Q8EJ99	--	12368813	Diguanylate cyclase activity	Not Available
2003	Diguanylate cyclase (EC 2.7.7.65)	*Shewanella oneidensis*	Strain MR-1	Q8EIP1	--	12368813	Diguanylate cyclase activity	Not Available
2004	Surface composition regulator	*Citrobacter spp. CFNIH10*		A0A3Q8DFL3	--	29437920	Not Available	Not Available
2005	Diguanylate cyclase with PAS sensory domain (EC 2.7.7.65)	*Shewanella oneidensis*	Strain MR-1	Q8EF28	--	12368813	Diguanylate cyclase activity, phosphoprotein phosphatase activity	Not Available
2006	Poly-beta-1, 6-N-acetyl-D-glucosamine synthase (Poly-beta-1, 6-GlcNAc synthase) (EC 2.4.1.-)	*Nitrospira bacterium SG8_35_1*		A0A0S8CZY6	--	25922666	Acetyl glucosaminyl transferase activity	Not Available
2007	Type II toxin-antitoxin system MqsR family toxin	*Pseudomonas spp. WS 5078*		A0A7Y1FKF5	--	32587583	Not Available	Not Available
2008	Diguanylate cyclase with HAMP domain (EC 2.7.7.65)	*Shewanella oneidensis*	Strain MR-1	Q8EC11	--	12368813	Diguanylate cyclase activity	Not Available
2009	Poly-beta-1, 6-N-acetyl-D-glucosamine synthase (Poly-beta-1, 6-GlcNAc synthase) (EC 2.4.1.-)	*Enterobacter ludwigii*		G8LE14	--	22675579	Acetyl glucosaminyl transferase activity	Not Available
2010	Diguanylate cyclase (EC 2.7.7.65)	*Shewanella oneidensis*	Strain MR-1	Q8EGF8	--	12368813	Diguanylate cyclase activity	Not Available
2011	Diguanylate cyclase (EC 2.7.7.65)	*Shewanella oneidensis*	Strain MR-1	Q8EIB5	--	12368813	Diguanylate cyclase activity	Not Available
2012	Outer membrane N-deacetylase	*Enterobacter ludwigii*		W0BV21	--	25197457	Hydrolase activity, acting on carbon-nitrogen (but not peptide) bonds	Not Available
2013	Outer membrane N-deacetylase	*Enterobacter ludwigii*		A0A7U4N614	--	26251488	Hydrolase activity, acting on carbon-nitrogen (but not peptide) bonds	Not Available
2014	P pilus subunit FimA	*Shewanella oneidensis*	Strain MR-1	Q8E8S9	--	12368813	Not Available	Not Available
2015	Diguanylate cyclase (EC 2.7.7.65)	*Shewanella oneidensis*	Strain MR-1	Q8EAN9	--	12368813	Diguanylate cyclase activity	Not Available
2016	Diguanylate cyclase (EC 2.7.7.65)	*Shewanella oneidensis*	Strain MR-1	Q8EGP7	--	12368813	Diguanylate cyclase activity	Not Available
2017	7TM intracellular sigNot Availablelling protein with GGDEF domain (EC 2.7.7.65)	*Shewanella oneidensis*	Strain MR-1	Q8EGN0	--	12368813	Diguanylate cyclase activity	Not Available
2018	NodB homology domain-containing protein	*Nitrospira bacterium SG8_35_1*		A0A0S8CYX5	--	25922666	Hydrolase activity, acting on carbon-nitrogen (but not peptide) bonds	Not Available
2019	Diguanylate cyclase with response regulator receiver modulation (EC 2.7.7.65)	*Shewanella oneidensis*	Strain MR-1	Q8EF60	--	12368813	Diguanylate cyclase activity, protein kinase activity	Not Available
2020	Diguanylate cyclase (EC 2.7.7.65)	*Shewanella oneidensis*	Strain MR-1	Q8EGW1	--	12368813	Diguanylate cyclase activity	Not Available
2021	Diguanylate cyclase	*Shewanella oneidensis*	Strain MR-1	Q8EBM2	--	12368813	Diguanylate cyclase activity	Not Available
2022	ABC transporter substrate-binding protein	*Enterobacter ludwigii*		A0A7U4N650	--	26251488	Not Available	Not Available
2023	BifunctioNot Availablel periplasmic substrate binding protein / cytoplasmic diguanylate cyclase	*Shewanella oneidensis*	Strain MR-1	Q8EB35	--	12368813	Diguanylate cyclase activity	Not Available
2024	Diguanylate cyclase (EC 2.7.7.65)	*Shewanella oneidensis*	Strain MR-1	Q8E979	--	12368813	Diguanylate cyclase activity	Not Available
2025	Diguanylate cyclase (EC 2.7.7.65)	*Shewanella oneidensis*	Strain MR-1	Q8E963	--	12368813	Diguanylate cyclase activity	Not Available
2026	Diguanylate cyclase (EC 2.7.7.65)	*Shewanella oneidensis*	Strain MR-1	Q8E936	--	12368813	Diguanylate cyclase activity	Not Available
2027	SigNot Availablelling protein with GGDEF domain	*Shewanella oneidensis*	Strain MR-1	Q8EDJ0	--	12368813	Diguanylate cyclase activity	Not Available
2028	BifunctioNot Availablel periplasmic substrate binding protein / cytoplasmic diguanylate cyclase (EC 2.7.7.65)	*Shewanella oneidensis*	Strain MR-1	Q8EIJ8	--	12368813	Diguanylate cyclase activity	Not Available
2029	Diguanylate cyclase with GAF sensory domain	*Shewanella oneidensis*	Strain MR-1	Q8EBG2	--	12368813	Diguanylate cyclase activity, phosphoprotein phosphatase activity	Not Available
2030	SigNot Availablelling protein with GGDEF domain	*Shewanella oneidensis*	Strain MR-1	Q8EE69	--	12368813	Diguanylate cyclase activity	Not Available
2031	Poly-beta-1, 6-N-acetyl-D-glucosamine N-deacetylase	*Enterobacter ludwigii*		G8LE13	--	22675579	Hydrolase activity, acting on carbon-nitrogen (but not peptide) bonds	Not Available
2032	Putative membrane protein	*Acinetobacter baumannii*	Strain ACICU	A0A7U3XX92	--	18411315	Not Available	Not Available
2033	Poly-beta-1, 6-N-acetyl-D-glucosamine synthase (Poly-beta-1, 6-GlcNAc synthase) (EC 2.4.1.-)	*Acinetobacter baumannii*	Strain ACICU	A0A7U4DDW4	--	18411315	Acetyl glucosaminyl transferase activity	Not Available
2034	Poly-beta-1, 6-N-acetyl-D-glucosamine synthase (Poly-beta-1, 6-GlcNAc synthase) (EC 2.4.1.-)	*Cupriavidus basilensis*		A0A0C4Y8S7	--	25977418	Acetyl glucosaminyl transferase activity	Not Available
2035	Uncharacterized protein	*Acinetobacter baumannii*	Strain ACICU	A0A7U3Y161	--	18411315	Not Available	Not Available
2036	Predicted xylanase / chitin deacetylase	*Acinetobacter baumannii*	Strain ACICU	A0A7U3Y173	--	18411315	Hydrolase activity, acting on carbon-nitrogen (but not peptide) bonds, Hydrolase activity, acting on glycosyl bonds	Not Available
2037	Surface composition regulator	*Cronobacter dublinensis subspp. dublinensis LMG 23823*		A0A0K2P1G4	--	27013041	Not Available	Not Available
2038	Poly-beta-1, 6-N-acetyl-D-glucosamine synthase (Poly-beta-1, 6-GlcNAc synthase) (EC 2.4.1.-)	*Acinetobacter baumannii*	Strain ACICU	A0A7U3XXA6	--	18411315	Acetyl glucosaminyl transferase activity	Not Available
2039	Uncharacterized protein	*Methylocucumis oryzae*		A0A0F3IJ44	--	26547566	Not Available	Not Available
2040	Biofilm PGA synthesis deacetylase PgaB (EC 3.-.-.-)	*Cupriavidus basilensis*		A0A0C4YAV1	--	25977418	Hydrolase activity, acting on carbon-nitrogen (but not peptide) bonds	Not Available
2041	Predicted xylanase / chitin deacetylase	*Acinetobacter baumannii*	Strain ACICU	A0A7U3XXD6	--	18411315	Hydrolase activity, acting on carbon-nitrogen (but not peptide) bonds, Hydrolase activity, acting on glycosyl bonds	Not Available

Target ID	Bacterial Protein Name	Organism Name	Strain	UniProt ID	PDB ID	PubMed ID	Classification as per UniProt GO Molecular Function	Classification of Function as per PDB
2042	Polysaccharide deacetylase	*Methylocucumis oryzae*		A0A0F3IGY7	--	26547566	Hydrolase activity, acting on carbon-nitrogen (but not peptide) bonds	Not Available
2043	Surface composition regulator	*Salmonella dublin*	Strain CT_02021853	A0A6C7A0T4	--	21602358	Not Available	Not Available
2044	Putative xylanase / chitin deacetylase	*Xanthomonas vesicatoria ATCC 35937*		F0BJX6	--	21396108	Hydrolase activity, acting on carbon-nitrogen (but not peptide) bonds, Hydrolase activity, acting on glycosyl bonds	Not Available
2045	Poly-beta-1, 6-N-acetyl-D-glucosamine synthase (Poly-beta-1, 6-GlcNAc synthase) (EC 2.4.1.-)	*Methylocucumis oryzae*		A0A0F3IH37	--	26547566	Acetyl glucosaminyl transferase activity	Not Available
2046	Uncharacterized protein	*Xanthomonas vesicatoria ATCC 35937*		F0BJX8	--	21396108	Not Available	Not Available
2047	Uncharacterized protein	*Methylocucumis oryzae*		A0A0F3IN01	--	26547566	Not Available	Not Available
2048	Uncharacterized protein	*Koribacter versatilis*	Strain Ellin345	Q1IJ03	--	19201974	Not Available	Not Available
2049	Poly-beta-1, 6-N-acetyl-D-glucosamine biosynthesis protein PgaD	*Oxalobacteraceae bacterium*		A0A4Q3IWL6	--	30498029	Not Available	Not Available
2050	Poly-beta-1, 6-N-acetyl-D-glucosamine biosynthesis protein PgaD	*Pelosinus spp. UFO1*		A0A075KH26	--	25189589	Not Available	Not Available
2051	Poly-beta-1, 6-N-acetyl-D-glucosamine synthase (Poly-beta-1, 6-GlcNAc synthase) (EC 2.4.1.-)	*Oxalobacteraceae bacterium*		A0A4Q3IX21	--	30498029	Acetyl glucosaminyl transferase activity	Not Available
2052	Poly-beta-1, 6-N-acetyl-D-glucosamine synthase (Poly-beta-1, 6-GlcNAc synthase) (EC 2.4.1.-)	*Pelosinus spp. UFO1*		A0A075KDU5	--	25189589	Acetyl glucosaminyl transferase activity	Not Available
2053	Uncharacterized protein	*Helicobacteraceae bacterium CG1_02_36_14*		A0A1J4U2J2	--	27112493	Not Available	Not Available
2054	Poly-beta-1, 6-N-acetyl-D-glucosamine synthase (Poly-beta-1, 6-GlcNAc synthase) (EC 2.4.1.-)	*Xanthomonas vesicatoria ATCC 35937*		F0BJX7	--	21396108	Acetyl glucosaminyl transferase activity	Not Available
2055	Poly-beta-1, 6-N-acetyl-D-glucosamine synthase (Poly-beta-1, 6-GlcNAc synthase) (EC 2.4.1.-)	*Escherichia coli O1:K1 / APEC*		A0A0H2YXS0	--	17293413	Acetyl glucosaminyl transferase activity	Not Available
2056	Poly-beta-1, 6-N-acetyl-D-glucosamine synthase (Poly-beta-1, 6-GlcNAc synthase) (EC 2.4.1.-)	*Staphylococcus aureus subspp. aureus M013*		A0A7U4AW02	--	22328755	Acetyl glucosaminyl transferase activity	Not Available
2057	Predicted inner Membrane protein	*Escherichia coli O78:H11*	Strain H10407 / ETEC	E3PJI8	--	20802035	Not Available	Not Available
2058	Putative polysaccharide deacetylase associated with biofilm formation putative lipoprotein	*Escherichia fergusonii*	Strain ATCC 35469 / DSM 13698 / CCUG 18766 / IAM 14443 / JCM 21226 / LMG 7866 / NBRC 102419 / NCTC 12128 / CDC 0568-73	B7LTB0	--	19165319	Hydrolase activity, acting on carbon-nitrogen (but not peptide) bonds	Not Available
2059	Poly-beta-1, 6-N-acetyl-D-glucosamine synthase (Poly-beta-1, 6-GlcNAc synthase) (EC 2.4.1.-)	*Escherichia fergusonii*	Strain ATCC 35469 / DSM 13698 / CCUG 18766 / IAM 14443 / JCM 21226 / LMG 7866 / NBRC 102419 / NCTC 12128 / CDC 0568-73	B7LTB1	--	19165319	Acetyl glucosaminyl transferase activity	Not Available
2060	Uncharacterized protein	*Escherichia coli O78:H11*	Strain H10407 / ETEC	E3PJE2	--	20802035	Not Available	Not Available
2061	Biofilm PGA synthesis protein pgaD	*Escherichia fergusonii*	Strain ATCC 35469 / DSM 13698 / CCUG 18766 / IAM 14443 / JCM 21226 / LMG 7866 / NBRC 102419 / NCTC 12128 / CDC 0568-73	B7LTB2	--	19165319	Not Available	Not Available
2062	Surface composition regulator	*Klebsiella pneumoniae subspp. pneumoniae KpQ3*		A0A7U9CYT9	--	23469341	Not Available	Not Available
2063	Hemin storage system protein	*Klebsiella pneumoniae subspp. pneumoniae KpQ3*		A0A7U9GZW8	--	23469341	Not Available	Not Available
2064	Poly-beta-1, 6-N-acetyl-D-glucosamine synthase (Poly-beta-1, 6-GlcNAc synthase) (EC 2.4.1.-)	*Klebsiella pneumoniae subspp. pneumoniae KpQ3*		A0A7U9D807	--	23469341	Acetyl glucosaminyl transferase activity	Not Available
2065	Outer membrane N-deacetylase	*Klebsiella pneumoniae subspp. pneumoniae KpQ3*		A0A7U9D765	--	23469341	Hydrolase activity, acting on carbon-nitrogen (but not peptide) bonds	Not Available
2066	Putative hemin storage protein	*Bordetella parapertussis*	Strain 12822 / ATCC BAA-587 / NCTC 13253	Q7W830	--	12910271	Hydrolase activity, acting on carbon-nitrogen (but not peptide) bonds	Not Available
2067	Uncharacterized protein	*Desulfovibrio spp. A2*		G2H343	--	22072648	Not Available	Not Available
2068	Poly-beta-1, 6-N-acetyl-D-glucosamine synthase (Poly-beta-1, 6-GlcNAc synthase) (EC 2.4.1.-)	*Pseudomonas spp. GM80*		J2Y7T2	--	23045501	Acetyl glucosaminyl transferase activity	Not Available
2069	Poly-beta-1, 6-N-acetyl-D-glucosamine N-deacetylase PgaB	*Pseudomonas spp. GM102*		J2MA13	--	23045501	Hydrolase activity, acting on carbon-nitrogen (but not peptide) bonds	Not Available
2070	Poly-beta-1, 6-N-acetyl-D-glucosamine synthase (Poly-beta-1, 6-GlcNAc synthase) (EC 2.4.1.-)	*Pseudomonas spp. GM102*		J3DYT0	--	23045501	Acetyl glucosaminyl transferase activity	Not Available
2071	Biofilm PGA synthesis lipoprotein PgaB	*Bibersteinia trehalosi USDA-ARS-USMARC-188*		A0A4V7IAU5	--	24526647	Hydrolase activity, acting on carbon-nitrogen (but not peptide) bonds	Not Available
2072	Poly-beta-1, 6-N-acetyl-D-glucosamine synthase (Poly-beta-1, 6-GlcNAc synthase) (EC 2.4.1.-)	*Bibersteinia trehalosi USDA-ARS-USMARC-188*		A0A4V7IB85	--	24526647	Acetyl glucosaminyl transferase activity	Not Available
2073	Uncharacterized protein	*Bordetella parapertussis*	Strain 12822 / ATCC BAA-587 / NCTC 13253	Q7WB14	--	12910271	Not Available	Not Available
2074	Poly-beta-1, 6-N-acetyl-D-glucosamine N-deacetylase PgaB	*Pseudomonas spp. GM80*		J3IPL5	--	23045501	Hydrolase activity, acting on carbon-nitrogen (but not peptide) bonds	Not Available
2075	Poly-beta-1, 6-N-acetyl-D-glucosamine synthase (Poly-beta-1, 6-GlcNAc synthase) (EC 2.4.1.-)	*Pseudomonas brassicacearum*		W8PKM7	--	24812222	Acetyl glucosaminyl transferase activity	Not Available
2076	Poly-beta-1, 6-N-acetyl-D-glucosamine synthase (Poly-beta-1, 6-GlcNAc synthase) (EC 2.4.1.-)	*Pseudomonas spp. GM18*		J2NNQ5	--	23045501	Acetyl glucosaminyl transferase activity	Not Available
2077	Outer membrane N-deacetylase	*Pseudomonas brassicacearum*		W8PPL1	--	24812222	Hydrolase activity, acting on carbon-nitrogen (but not peptide) bonds	Not Available
2078	Biofilm PGA synthesis protein PgaD	*Pseudomonas brassicacearum*		W8PCX6	--	24812222	Not Available	Not Available
2079	Poly-beta-1, 6-N-acetyl-D-glucosamine biosynthesis protein PgaD	*Pseudomonas spp. GM18*		J3EH54	--	23045501	Not Available	Not Available
2080	Type II toxin-antitoxin system MqsR family toxin	*Geobacter spp.*		A0A351WRQ6	--	30148503	Not Available	Not Available
2081	Poly-beta-1, 6-N-acetyl-D-glucosamine synthase (Poly-beta-1, 6-GlcNAc synthase) (EC 2.4.1.-)	*Bordetella parapertussis*	Strain 12822 / ATCC BAA-587 / NCTC 13253	Q7W831	--	12910271	Acetyl glucosaminyl transferase activity	Not Available
2082	Uncharacterized protein	*Pediococcus damnosus*		A0A2G9VE09	--	29126241	Not Available	Not Available
2083	Uncharacterized protein	*Pseudomonas spp. GM102*		J3DNY8	--	23045501	Not Available	Not Available

Target ID	Bacterial Protein Name	Organism Name	Strain	UniProt ID	PDB ID	PubMed ID	Classification as per UniProt GO Molecular Function	Classification of Function as per PDB
2084	Putative membrane protein	*Bordetella parapertussis*	Strain 12822 / ATCC BAA-587 / NCTC 13253	Q7W832	--	12910271	Not Available	Not Available
2085	Poly-beta-1, 6-N-acetyl-D-glucosamine biosynthesis protein PgaD	*Pseudomonas spp. GM102*		J2VDN2	--	23045501	Not Available	Not Available
2086	Poly-beta-1, 6-N-acetyl-D-glucosamine synthase (Poly-beta-1, 6-GlcNAc synthase) (EC 2.4.1.-)	*Escherichia coli*	Strain UTI89 / UPEC	Q1RDJ5	--	16585510	Acetyl glucosaminyl transferase activity	Not Available
2087	Poly-beta-1, 6-N-acetyl-D-glucosamine synthase (Poly-beta-1, 6-GlcNAc synthase) (EC 2.4.1.-) (Fragment)	*Enterobacteriaceae bacterium*		A0A3C2BGL3	--	30148503	Acetyl glucosaminyl transferase activity	Not Available
2088	Poly-beta-1, 6-N-acetyl-D-glucosamine synthase (Poly-beta-1, 6-GlcNAc synthase) (EC 2.4.1.-)	*Halomonas spp. TD01*		F7SRR7	--	22040376	Acetyl glucosaminyl transferase activity	Not Available
2089	Poly-beta-1, 6-N-acetyl-D-glucosamine N-deacetylase (EC 3.5.1.-)	*Acinetobacter baumannii*	Strain AB307-0294	A0A5K6CQ92	--	18931120	Hydrolase activity, acting on carbon-nitrogen (but not peptide) bonds	Not Available
2090	Putative major fimbrial subunit	*Salmonella typhimurium*	Strain LT2 / SGSC1412 / ATCC 700720	Q8ZJU3	--	11677609	Not Available	Not Available
2091	Two-component response regulator	*Leptospira interrogans serogroup Icterohaemorrhagiae serovar Lai*	Strain 56601	Q8F2R4	--	12712204	Diguanylate cyclase activity	Not Available
2092	Response regulator	*Leptospira interrogans serogroup Icterohaemorrhagiae serovar Lai*	Strain 56601	Q8F629	--	12712204	Diguanylate cyclase activity	Not Available
2093	Poly-beta-1, 6-N-acetyl-D-glucosamine synthase (Poly-beta-1, 6-GlcNAc synthase) (EC 2.4.1.-)	*Acinetobacter baumannii*	Strain AB307-0294	A0A5K6CPH3	--	18931120	Acetyl glucosaminyl transferase activity	Not Available
2094	Putative fimbriae major subunit	*Salmonella typhimurium*	Strain LT2 / SGSC1412 / ATCC 700720	Q8ZRH9	--	11677609	Not Available	Not Available
2095	Putative fimbrial-like protein	*Salmonella typhimurium*	Strain LT2 / SGSC1412 / ATCC 700720	Q7CQ93	--	11677609	Not Available	Not Available
2096	Putative fimbrial-like protein	*Salmonella typhimurium*	Strain LT2 / SGSC1412 / ATCC 700720	Q8ZM89	--	11677609	Not Available	Not Available
2097	Fimbrial subunit	*Salmonella typhimurium*	Strain LT2 / SGSC1412 / ATCC 700720	H9L412	--	11677609	Not Available	Not Available
2098	Surface composition regulator	*Escherichia fergusonii*	Strain ATCC 35469 / DSM 13698 / CCUG 18766 / IAM 14443 / JCM 21226 / LMG 7866 / NBRC 102419 / NCTC 12128 / CDC 0568-73	B7LQC5	--	19165319	Not Available	Not Available
2099	Uncharacterized protein	*Priestia megaterium*	Strain ATCC 12872 / QMB1551 Bacillus megaterium	D5E339	--	21705586	Not Available	Not Available
2100	Poly-beta-1, 6-N-acetyl-D-glucosamine synthase (Poly-beta-1, 6-GlcNAc synthase) (EC 2.4.1.-)	*Escherichia coli O104:H4*	Strain 2011C-3493	A0A0E0Y0U3	--	23133618	Acetyl glucosaminyl transferase activity	Not Available
2101	Putative PGA biosynthesis protein	*Escherichia coli O104:H4*	Strain 2011C-3493	A0A0E0Y2B1	--	23133618	Not Available	Not Available
2102	Poly-beta-1, 6-N-acetyl-D-glucosamine synthase (Poly-beta-1, 6-GlcNAc synthase) (EC 2.4.1.-)	*Priestia megaterium*	Strain ATCC 12872 / QMB1551 Bacillus megaterium	D5E340	--	21705586	Acetyl glucosaminyl transferase activity	Not Available
2103	Poly-beta-1, 6-N-acetyl-D-glucosamine synthase (Poly-beta-1, 6-GlcNAc synthase) (EC 2.4.1.-)	*Klebsiella pneumoniae 30684 / NJST258_2*		W8URY0	--	24639510	Acetyl glucosaminyl transferase activity	Not Available
2104	Secreted biofilm formation protein YqxM	*Bacillus sonorensis L12*		M5P5Z6	--	23538904	Not Available	Not Available
2105	Outer membrane N-deacetylase	*Escherichia coli O104:H4*	Strain 2011C-3493	A0A0E0Y2J4	--	23133618	Hydrolase activity, acting on carbon-nitrogen (but not peptide) bonds	Not Available
2106	Hemin storage protein HmsS	*Klebsiella pneumoniae 30684 / NJST258_2*		W8VIB3	--	24639510	Not Available	Not Available
2107	Surface composition regulator	*Klebsiella pneumoniae 30684 / NJST258_2*		W8UUA8	--	24639510	Not Available	Not Available
2108	Surface composition regulator	*Escherichia coli O104:H4*	Strain 2011C-3493	A0A0E0XXK7	--	23133618	Not Available	Not Available
2109	mRNA interferase MqsR	*Gallionella spp.*		A0A3D2NL57	--	30148503	Not Available	Not Available
2110	HmsF protein	*Klebsiella pneumoniae 30684 / NJST258_2*		W8UPN5	--	24639510	Hydrolase activity, acting on carbon-nitrogen (but not peptide) bonds	Not Available
2111	Poly-beta-1, 6-N-acetyl-D-glucosamine synthase (Poly-beta-1, 6-GlcNAc synthase) (EC 2.4.1.-)	*Syntrophobacter spp. DG_60*		A0A0S7XPJ3	--	25922666	Acetyl glucosaminyl transferase activity	Not Available
2112	Poly-beta-1, 6-N-acetyl-D-glucosamine biosynthesis protein PgaD	*Lentisphaerae bacterium GWF2_44_16*		A0A1G0YRT2	--	27774985	Not Available	Not Available
2113	Poly-beta-1, 6-N-acetyl-D-glucosamine N-deacetylase PgaB	*Acinetobacter baumannii*	Strain AB0057	A0A7U3Y5V9	--	18931120	Hydrolase activity, acting on carbon-nitrogen (but not peptide) bonds	Not Available
2114	Poly-beta-1, 6-N-acetyl-D-glucosamine synthase (Poly-beta-1, 6-GlcNAc synthase) (EC 2.4.1.-)	*Acinetobacter baumannii*	Strain AB0057	A0A7U4DHS5	--	18931120	Acetyl glucosaminyl transferase activity	Not Available
2115	Uncharacterized protein	*Pseudomonas fluorescens*	Strain SBW25	C3K4N7	--	19432983	Not Available	Not Available
2116	Poly-beta-1, 6-N-acetyl-D-glucosamine synthase (Poly-beta-1, 6-GlcNAc synthase) (EC 2.4.1.-)	*Staphylococcus aureus subspp. aureus 71193*		A0A0E0VSR0	--	22375071	Acetyl glucosaminyl transferase activity	Not Available
2117	Poly-beta-1, 6-N-acetyl-D-glucosamine biosynthesis protein PgaD	*Acinetobacter baumannii*	Strain AB0057	A0A7U3Y617	--	18931120	Not Available	Not Available
2118	Poly-beta-1, 6-N-acetyl-D-glucosamine N-deacetylase PgaB	*Acinetobacter baumannii*	Strain AB0057	A0A7U3Y934	--	18931120	Hydrolase activity, acting on carbon-nitrogen (but not peptide) bonds	Not Available
2119	Poly-beta-1, 6-N-acetyl-D-glucosamine synthase (Poly-beta-1, 6-GlcNAc synthase) (EC 2.4.1.-)	*Pseudomonas fluorescens*	Strain SBW25	C3KAF9	--	19432983	Acetyl glucosaminyl transferase activity	Not Available
2120	Poly-beta-1, 6-N-acetyl-D-glucosamine synthase (Poly-beta-1, 6-GlcNAc synthase) (EC 2.4.1.-)	*Paraburkholderia caribensis MBA4*		A0A0P0RAV3	--	24558235	Acetyl glucosaminyl transferase activity	Not Available
2121	Poly-beta-1, 6-N-acetyl-D-glucosamine biosynthesis protein PgaD	*Acinetobacter baumannii*	Strain AB0057	A0A7U3Y8Z6	--	18931120	Not Available	Not Available
2122	Type II toxin-antitoxin system MqsR family toxin	*Geobacter spp.*		A0A3N9VGQ2	--	30086797	Not Available	Not Available
2123	Type II toxin-antitoxin system MqsR family toxin	*Geobacter spp.*		A0A353MF81	--	30148503	Not Available	Not Available
2124	Poly-beta-1, 6-N-acetyl-D-glucosamine synthase (Poly-beta-1, 6-GlcNAc synthase) (EC 2.4.1.-)	*Clostridiaceae bacterium*		A0A7C6H2P8	--	32123542	Acetyl glucosaminyl transferase activity	Not Available
2125	Poly-beta-1, 6-N-acetyl-D-glucosamine N-deacetylase PgaB	*Pseudomonas spp. GM18*		J2W8M4	--	23045501	Hydrolase activity, acting on carbon-nitrogen (but not peptide) bonds	Not Available
2126	HmsF protein	*Klebsiella pneumoniae*		W9BQH1	--	25341126	Hydrolase activity, acting on carbon-nitrogen (but not peptide) bonds	Not Available
2127	Putative membrane protein	*Bordetella parapertussis*	Strain Bpp5	K0MBZ6	--	23051057	Not Available	Not Available

Target ID	Bacterial Protein Name	Organism Name	Strain	UniProt ID	PDB ID	PubMed ID	Classification as per UniProt GO Molecular Function	Classification of Function as per PDB
2128	AI-2 synthesis protein (EC 4.4.1.21) (Autoinducer-2 production protein LuxS) (S-ribosylhomocysteine lyase)	*Capnocytophaga ochracea*	Strain ATCC 27872 / DSM 7271 / JCM 12966 / NCTC 12371 / VPI 2845 Bacteroides ochraceus	C7M4X6	--	21304645	Iron ion binding, S-ribosylhomocysteine lyase activity	Not Available
2129	Putative hemin storage protein	*Bordetella parapertussis*	Strain Bpp5	K0MFJ0	--	23051057	Hydrolase activity, acting on carbon-nitrogen (but not peptide) bonds	Not Available
2130	Poly-beta-1, 6-N-acetyl-D-glucosamine synthase (Poly-beta-1, 6-GlcNAc synthase) (EC 2.4.1.-)	*Bordetella parapertussis*	Strain Bpp5	K0MDE7	--	23051057	Acetyl glucosaminyl transferase activity	Not Available
2131	Poly-beta-1, 6-N-acetyl-D-glucosamine synthase (Poly-beta-1, 6-GlcNAc synthase) (EC 2.4.1.-)	*Acinetobacter calcoaceticus*	Strain PHEA-2	F0KG89	--	21441526	Acetyl glucosaminyl transferase activity	Not Available
2132	Uncharacterized protein	*Ralstonia solanacearum*	Strain UW551	A3RPC6	--	16404955	Not Available	Not Available
2133	Uncharacterized protein	*Gordonibacter pamelaeae*		A0A369M219	--	29761785	Not Available	Not Available
2134	Polysaccharide deacetylase	*Methylomona methanica*	Strain MC09	G0A379	--	22123758	Hydrolase activity, acting on carbon-nitrogen (but not peptide) bonds	Not Available
2135	HmsF protein	*Ralstonia solanacearum*	Strain UW551	A3RPC4	--	16404955	Hydrolase activity, acting on carbon-nitrogen (but not peptide) bonds	Not Available
2136	Poly-beta-1, 6-N-acetyl-D-glucosamine synthase (Poly-beta-1, 6-GlcNAc synthase) (EC 2.4.1.-)	*Acinetobacter calcoaceticus*	Strain PHEA-2	F0KHT9	--	21441526	Acetyl glucosaminyl transferase activity	Not Available
2137	Biofilm PGA synthesis lipoprotein PgaB	*Acinetobacter calcoaceticus*	Strain PHEA-2	F0KG88	--	21441526	Hydrolase activity, acting on carbon-nitrogen (but not peptide) bonds	Not Available
2138	Poly-beta-1, 6-N-acetyl-D-glucosamine synthase (Poly-beta-1, 6-GlcNAc synthase) (EC 2.4.1.-)	*Ralstonia solanacearum*	Strain UW551	A3RPC5	--	16404955	Acetyl glucosaminyl transferase activity	Not Available
2139	Poly-beta-1, 6-N-acetyl-D-glucosamine synthase (Poly-beta-1, 6-GlcNAc synthase) (EC 2.4.1.-)	*Oceanisphaera profunda*		A0A1Y0D3Z0	--	24425742	Acetyl glucosaminyl transferase activity	Not Available
2140	Uncharacterized protein	*Acinetobacter calcoaceticus*	Strain PHEA-2	F0KHT8	--	21441526	Not Available	Not Available
2141	Poly-beta-1, 6-N-acetyl-D-glucosamine synthase (Poly-beta-1, 6-GlcNAc synthase) (EC 2.4.1.-)	*Bordetella pertussis STO1-CHOC-0019*		A0A6N3RYK7	--	24356839	Acetyl glucosaminyl transferase activity	Not Available
2142	Poly-beta-1, 6-N-acetyl-D-glucosamine synthase (Poly-beta-1, 6-GlcNAc synthase) (EC 2.4.1.-)	*Yersinia pestis bv. Antiqua*	Strain Antiqua	A0A0H2Y673	--	16740952	Acetyl glucosaminyl transferase activity	Not Available
2143	Biofilm PGA synthesis lipoprotein PgaB	*Acinetobacter calcoaceticus*	Strain PHEA-2	F0KHU0	--	21441526	Hydrolase activity, acting on carbon-nitrogen (but not peptide) bonds	Not Available
2144	Uncharacterized protein	*Acinetobacter calcoaceticus*	Strain PHEA-2	F0KG90	--	21441526	Not Available	Not Available
2145	Poly-beta-1, 6-N-acetyl-D-glucosamine synthase (Poly-beta-1, 6-GlcNAc synthase) (EC 2.4.1.-)	*Rubrivivax gelatinosus*	Strain NBRC 100245 / IL144	I0HLL7	--	22689232	Acetyl glucosaminyl transferase activity	Not Available
2146	Surface composition regulator	*Yersinia enterocolitica subspp. palearctica serotype O:3*	Strain DSM 13030 / CIP 106945 / Y11	A0A0H3NPC2	--	21296963	DNA binding, DNA-directed DNA polymerase activity	Not Available
2147	Uncharacterized protein	*Pseudomonas spp. GM50*		J3AUJ3	--	23045501	Not Available	Not Available
2148	Poly-beta-1, 6-N-acetyl-D-glucosamine synthase (Poly-beta-1, 6-GlcNAc synthase) (EC 2.4.1.-)	*Yersinia enterocolitica subspp. palearctica serotype O:3*	Strain DSM 13030 / CIP 106945 / Y11	A0A0H3NQX9	--	21296963	Acetyl glucosaminyl transferase activity	Not Available
2149	Type II toxin-antitoxin system MqsR family toxin	*Pseudomonas mandelii*		A0A502IHF4	--	31206918	Not Available	Not Available
2150	Poly-beta-1, 6-N-acetyl-D-glucosamine synthase (Poly-beta-1, 6-GlcNAc synthase) (EC 2.4.1.-)	*Pseudomonas spp. GM50*		J2SHC7	--	23045501	Acetyl glucosaminyl transferase activity	Not Available
2151	Poly-beta-1, 6-N-acetyl-D-glucosamine biosynthesis protein PgaD	*Pseudomonas spp. GM50*		J3GJ92	--	23045501	Not Available	Not Available
2152	Poly-beta-1, 6-N-acetyl-D-glucosamine N-deacetylase PgaB	*Pseudomonas spp. GM50*		J3GJ29	--	23045501	Hydrolase activity, acting on carbon-nitrogen (but not peptide) bonds	Not Available
2153	Uncharacterized protein	*Pseudomonas furukawaii*		L8MFN8	--	22843571	Not Available	Not Available
2154	Uncharacterized protein	*Acidithiobacillales bacterium SM23_46*		A0A0S8GIC3	--	25922666	Not Available	Not Available
2155	Uncharacterized protein	*Methylococcaceae bacterium*		A0A351V4P3	--	30148503	Not Available	Not Available
2156	Poly-beta-1, 6-N-acetyl-D-glucosamine N-deacetylase PgaB (Fragment)	*Methylococcaceae bacterium*		A0A351KVS9	--	30148503	Hydrolase activity, acting on carbon-nitrogen (but not peptide) bonds	Not Available
2157	Poly-beta-1, 6-N-acetyl-D-glucosamine synthase (Poly-beta-1, 6-GlcNAc synthase) (EC 2.4.1.-)	*Acidithiobacillales bacterium SM23_46*		A0A0S8GIQ5	--	25922666	Acetyl glucosaminyl transferase activity	Not Available
2158	Poly-beta-1, 6-N-acetyl-D-glucosamine biosynthesis protein PgaD (Fragment)	*Methylococcaceae bacterium*		A0A351KVS6	--	30148503	Not Available	Not Available
2159	Poly-beta-1, 6-N-acetyl-D-glucosamine N-deacetylase PgaB	*Lautropia spp. SCN 69-89*		A0A1D2SJQ0	--	26031303	Hydrolase activity, acting on carbon-nitrogen (but not peptide) bonds	Not Available
2160	Uncharacterized protein	*Burkholderia multivorans CGD2*		B9BI89	--	23105085	Not Available	Not Available
2161	Poly-beta-1, 6-N-acetyl-D-glucosamine synthase (Poly-beta-1, 6-GlcNAc synthase) (EC 2.4.1.-)	*Mannheimia succiniciproducens*	Strain MBEL55E	Q65TJ1	--	15378067	Acetyl glucosaminyl transferase activity	Not Available
2162	Poly-beta-1, 6-N-acetyl-D-glucosamine synthase (Poly-beta-1, 6-GlcNAc synthase) (EC 2.4.1.-)	*Lautropia spp. SCN 69-89*		A0A1D2SJP8	--	26031303	Acetyl glucosaminyl transferase activity	Not Available
2163	Outer membrane N-deacetylase	*Yersinia enterocolitica subspp. palearctica serotype O:3*	Strain YE-P4	A0A7H4ZKR3	--	23846271	Hydrolase activity, acting on carbon-nitrogen (but not peptide) bonds	Not Available
2164	Outer membrane N-deacetylase	*Pectobacterium peruviense*		A0A0J5XC35	--	26251497	Hydrolase activity, acting on carbon-nitrogen (but not peptide) bonds	Not Available
2165	Poly-beta-1, 6-N-acetyl-D-glucosamine synthase (Poly-beta-1, 6-GlcNAc synthase) (EC 2.4.1.-)	*Priestia megaterium*	Bacillus megaterium	A0A5B8PY98	--	31228823	Acetyl glucosaminyl transferase activity	Not Available
2166	Surface composition regulator	*Shimwellia blattae*	Strain ATCC 29907 / DSM 4481 / JCM 1650 / NBRC 105725 / CDC 9005-74 Escherichia blattae	I2B566	--	22843577	Not Available	Not Available
2167	Poly-beta-1, 6-N-acetyl-D-glucosamine biosynthesis protein PgaD	*Oceanisphaera profunda*		A0A1Y0D3X8	--	24425742	Not Available	Not Available
2168	Surface composition regulator	*Cronobacter dublinensis 582*		K8AVI0	--	23166675	Not Available	Not Available
2169	Polysaccharide deacetylase domain protein	*Burkholderia multivorans CGD2*		B9BJ72	--	23105085	Hydrolase activity, acting on carbon-nitrogen (but not peptide) bonds	Not Available
2170	Poly-beta-1, 6-N-acetyl-D-glucosamine N-deacetylase PgaB	*Pseudomonas chlororaphis*		A0A0D5Y8P3	--	25679537	Hydrolase activity, acting on carbon-nitrogen (but not peptide) bonds	Not Available
2171	Motility quorum-sensing regulator MqsR	*Pseudomonas chlororaphis*		A0A0D5Y591	--	25679537	Not Available	Not Available
2172	GGDEF domain-containing protein	*Thermotoga maritima*	Strain ATCC 43589 / DSM 3109 / JCM 10099 / NBRC 100826 / MSB8	Q9X0P6	--	10360571	Diguanylate cyclase activity	Not Available
2173	Biofilm PGA synthesis protein PgaD	*Pseudomonas chlororaphis*		A0A0G3GEA7	--	26634018	Not Available	Not Available
2174	Poly-beta-1, 6-N-acetyl-D-glucosamine synthase (Poly-beta-1, 6-GlcNAc synthase) (EC 2.4.1.-)	*Stenotrophomonas spp. YAU14A_MKIMI4_1*		A0A7U6AEA2	--	29765358	Acetyl glucosaminyl transferase activity	Not Available

Target ID	Bacterial Protein Name	Organism Name	Strain	UniProt ID	PDB ID	PubMed ID	Classification as per UniProt GO Molecular Function	Classification of Function as per PDB
2175	Biofilm PGA synthesis protein PgaD	*Pseudomonas chlororaphis*		A0A0D5Y8I0	--	25679537	Not Available	Not Available
2176	Poly-beta-1, 6-N-acetyl-D-glucosamine synthase (Poly-beta-1, 6-GlcNAc synthase) (EC 2.4.1.-)	*Pseudomonas chlororaphis*		A0A0G3G9K3	--	26634018	Acetyl glucosaminyl transferase activity	Not Available
2177	Outer membrane N-deacetylase	*Pseudomonas chlororaphis*		A0A0G3GBF9	--	26634018	Hydrolase activity, acting on carbon-nitrogen (but not peptide) bonds	Not Available
2178	Uncharacterized protein	*Companilactobacillus mindensis DSM 14500*		A0A0R1QRU5	--	26415554	Not Available	Not Available
2179	Motility quorum-sensing regulator MqsR	*candidate division NC10 bacterium*		A0A2U1RPS3	--	29662147	Not Available	Not Available
2180	Outer membrane N-deacetylase	*Pseudomonas fluorescens HK44*		A0A010T0D0	--	21742869	Hydrolase activity, acting on carbon-nitrogen (but not peptide) bonds	Not Available
2181	Poly-beta-1, 6-N-acetyl-D-glucosamine synthase (Poly-beta-1, 6-GlcNAc synthase) (EC 2.4.1.-)	*Tetragenococcus koreensis*		A0A3G5F5R6	--	16014460	Acetyl glucosaminyl transferase activity	Not Available
2182	Poly-beta-1, 6-N-acetyl-D-glucosamine N-deacetylase	*Oceanisphaera profunda*		A0A1Y0D9J9	--	24425742	Hydrolase activity, acting on carbon-nitrogen (but not peptide) bonds	Not Available
2183	Poly-beta-1, 6-N-acetyl-D-glucosamine synthase (Poly-beta-1, 6-GlcNAc synthase) (EC 2.4.1.-)	*Pseudomonas fluorescens HK44*		A0A010RED1	--	21742869	Acetyl glucosaminyl transferase activity	Not Available
2184	Motility quorum-sensing regulator MqsR	*Pseudomonas fluorescens HK44*		A0A010SPS9	--	21742869	Not Available	Not Available
2185	Lipoprotein for biofilm formation	*Bacillus subtilis subspp. subtilis*	Str. SC-8	G4ETW0	--	22207744	Not Available	Not Available
2186	Biofilm PGA synthesis protein PgaD	*Pseudomonas fluorescens HK44*		A0A010SD71	--	21742869	Not Available	Not Available
2187	Poly-beta-1, 6-N-acetyl-D-glucosamine synthase (Poly-beta-1, 6-GlcNAc synthase) (EC 2.4.1.-)	*Actinobacillus pleuropneumoniae serotype 3*	Strain JL03	B0BTK4	--	18197260	Acetyl glucosaminyl transferase activity	Not Available
2188	Poly-beta-1, 6-N-acetyl-D-glucosamine synthase (Poly-beta-1, 6-GlcNAc synthase) (EC 2.4.1.-)	*Pseudomonas spp. WS 5027*		A0A7Y1B5C2	--	32587583	Acetyl glucosaminyl transferase activity	Not Available
2189	Uncharacterized protein	*Aquitalea magnusonii*		A0A3G9GES3	--	28293292	Not Available	Not Available
2190	Surface composition regulator	*Salmonella typhimurium*	Strain 14028S / SGSC 2262	A0A0F6B6X7	--	19897643	Not Available	Not Available
2191	Flagellar Transcriptional regulator FlhC	*Salmonella typhimurium*	Strain 14028S / SGSC 2262	A0A0F6B2Q8	--	19897643	DNA binding, zinc ion binding	Not Available
2192	Flagellar Transcriptional regulator FlhD	*Salmonella typhimurium*	Strain 14028S / SGSC 2262	A0A0F6B2Q9	--	19897643	DNA binding	Not Available
2193	Poly-beta-1, 6-N-acetyl-D-glucosamine biosynthesis protein PgaD	*Pseudomonas spp. WS 5027*		A0A7Y1B5A3	--	32587583	Not Available	Not Available
2194	Poly-beta-1, 6-N-acetyl-D-glucosamine synthase (Poly-beta-1, 6-GlcNAc synthase) (EC 2.4.1.-)	*Thiotrichales bacterium SG8_50*		A0A0S8ELF2	--	25922666	Acetyl glucosaminyl transferase activity	Not Available
2195	Uncharacterized protein	*Thiotrichales bacterium SG8_50*		A0A0S8EME4	--	25922666	Not Available	Not Available
2196	Transcriptional regulatory protein SrrA (Staphylococcal respiratory response protein A)	*Staphylococcus aureus*	Strain Mu50 / ATCC 700699	Q7A2R6	--	11418146	DNA binding	Not Available
2197	Sensor protein SrrB (EC 2.7.13.3) (Staphylococcal respiratory response protein B)	*Staphylococcus aureus*	Strain Mu50 / ATCC 700699	Q99TZ9	--	11418146	ATP binding, phosphorelay sensor kinase activity	Not Available
2198	Transcriptional regulatory protein SrrA (Staphylococcal respiratory response protein A)	*Staphylococcus aureus*	Strain MSSA476	Q6G972	--	15213324	DNA binding	Not Available
2199	Transcriptional regulatory protein SrrA (Staphylococcal respiratory response protein A)	*Staphylococcus aureus*		Q9L524	--	11157922; 15060046	DNA binding	Not Available
2200	Transcriptional regulatory protein SrrA (Staphylococcal respiratory response protein A)	*Staphylococcus aureus*	Strain MW2	Q7A0U4	--	12044378	DNA binding	Not Available
2201	Diguanylate cyclase VdcA (DGC) (EC 2.7.7.65)	*Vibrio cholerae serotype O1*	Strain ATCC 39315 / El Tor Inaba N16961	Q9KKZ4	--	10952301; 18227161	Diguanylate cyclase activity, GTP binding, Metal ion binding	Not Available
2202	signaltransduction protein TRAP (Target of RNAIII-activating protein)	*Staphylococcus aureus*	Strain USA300	Q2FFR1	--	16517273	Not Available	Not Available
2203	signaltransduction protein TRAP (Target of RNAIII-activating protein)	*Staphylococcus aureus*	Strain bovine RF122 / ET3-1	Q2YU04	--	17971880	Not Available	Not Available
2204	signaltransduction protein TRAP (Target of RNAIII-activating protein)	*Staphylococcus aureus*	Strain COL	Q5HEU0	--	15774886	Not Available	Not Available
2205	signaltransduction protein TRAP (Target of RNAIII-activating protein)	*Staphylococcus aureus*	Strain Mu50 / ATCC 700699	Q7A2Q4	--	11418146	Not Available	Not Available
2206	signaltransduction protein TRAP (Target of RNAIII-activating protein)	*Staphylococcus aureus*	Strain N315	Q7A4W3	--	11418146	Not Available	Not Available
2207	signaltransduction protein TRAP (Target of RNAIII-activating protein)	*Staphylococcus aureus*	Strain MRSA252	Q6GFM2	--	15213324	Not Available	Not Available
2208	signaltransduction protein TRAP (Target of RNAIII-activating protein)	*Staphylococcus aureus*	Strain MSSA476	Q6G8A1	--	15213324	Not Available	Not Available
2209	signaltransduction protein TRAP (Target of RNAIII-activating protein)	*Staphylococcus aureus*	Strain MW2	Q8NVW1	--	12044378	Not Available	Not Available
2210	signaltransduction protein TRAP (Target of RNAIII-activating protein)	*Staphylococcus epidermidis*		Q8GQQ1	--	12599079	Not Available	Not Available
2211	signaltransduction protein TRAP (Target of RNAIII-activating protein)	*Staphylococcus epidermidis*	Strain ATCC 35984 / RP62A	Q5HNA3	--	15774886; 16842355	Not Available	Not Available
2212	RNA polymerase sigma-54 factor	*Pseudomonas aeruginosa*	Strain ATCC 15692 / DSM 22644 / CIP 104116 / JCM 14847 / LMG 12228 / 1C / PRS 101 / PAO1	P49988	--	8113171; 10984043; 2152909; 9595661; 12644493; 10792721; 26633362; 29760208	DNA-binding Transcription activator activity, DNA-directed 5'-3' RNA polymerase activity, sigma factor activity, Transcription cis-regulatory region binding	Not Available
2213	Uncharacterized fimbrial-like protein SfmA (Type-1A pilin)	*Escherichia coli*	Strain K12	P0ABW5	--	9278503; 16738553; 20345943	Not Available	Not Available
2214	Protein translocase subunit SecA 2 (EC 7.4.2.8)	*Streptococcus pneumoniae serotype 4*	Strain ATCC BAA-334 / TIGR4	Q97P83	--	11463916; 16861665; 18507531; 20714350; 28456649	ATP binding, protein-exporting ATPase activity	Not Available
2215	Sensor protein SrrB (EC 2.7.13.3) (Staphylococcal respiratory response protein B)	*Staphylococcus aureus*	Strain COL	Q5HFT1	--	15774886; 16859493; 17384184; 24222487	ATP binding, phosphorelay sensor kinase activity	Not Available
2216	Transcriptional regulatory protein LytR (Sensory transduction protein LytR)	*Staphylococcus aureus*	Strain USA300	Q2FK09	--	16517273	DNA binding	Not Available
2217	Transcriptional regulatory protein LytR (Sensory transduction protein LytR)	*Staphylococcus aureus*	Strain bovine RF122 / ET3-1	Q2YV67	--	17971880	DNA binding	Not Available
2218	Transcriptional regulatory protein LytR (Sensory transduction protein LytR)	*Staphylococcus aureus*	Strain COL	Q5HJB5	--	15774886	DNA binding	Not Available
2219	Transcriptional regulatory protein LytR (Sensory transduction protein LytR)	*Staphylococcus aureus*	Strain Mu50 / ATCC 700699	P60609	--	11418146; 27127614	DNA binding	Not Available
2220	Transcriptional regulatory protein LytR (Sensory transduction protein LytR)	*Staphylococcus aureus*	Strain N315	P60610	--	11418146	DNA binding	Not Available

Target ID	Bacterial Protein Name	Organism Name	Strain	UniProt ID	PDB ID	PubMed ID	Classification as per UniProt GO Molecular Function	Classification of Function as per PDB
2221	Transcriptional regulatory protein LytR (Sensory transduction protein LytR)	*Staphylococcus aureus*	Strain MRSA252	Q6GK51	--	15213324	DNA binding	Not Available
2222	Transcriptional regulatory protein LytR (Sensory transduction protein LytR)	*Staphylococcus aureus*	Strain MSSA476	Q6GCL1	--	15213324	DNA binding	Not Available
2223	Transcriptional regulatory protein LytR (Sensory transduction protein LytR)	*Staphylococcus aureus*	Strain MW2	Q8NYH3	--	12044378	DNA binding	Not Available
2224	Sensor histidinekinase / phosphatase LytS (EC 2.7.13.3) (EC 3.1.3.-) (Autolysin sensorkinase)	*Staphylococcus aureus*	Strain USA300	Q2FK10	--	16517273	ATP binding, Hydrolase activity, phosphorelay sensor kinase activity	Not Available
2225	Sensor histidinekinase / phosphatase LytS (EC 2.7.13.3) (EC 3.1.3.-) (Autolysin sensorkinase)	*Staphylococcus aureus*	Strain NCTC 8325 / PS 47	Q53705	--	8550490; 9308171; 19502411; 23733465; 25491472	ATP binding, Hydrolase activity, phosphorelay sensor kinase activity	Not Available
2226	Sensor histidinekinase / phosphatase LytS (EC 2.7.13.3) (EC 3.1.3.-) (Autolysin sensorkinase)	*Staphylococcus aureus*	Strain bovine RF122 / ET3-1	Q2YV68	--	17971880	ATP binding, Hydrolase activity, phosphorelay sensor kinase activity	Not Available
2227	Sensor histidinekinase / phosphatase LytS (EC 2.7.13.3) (EC 3.1.3.-) (Autolysin sensorkinase)	*Staphylococcus aureus*	Strain COL	Q5HJB6	--	15774886	ATP binding, Hydrolase activity, phosphorelay sensor kinase activity	Not Available
2228	Sensor histidinekinase / phosphatase LytS (EC 2.7.13.3) (EC 3.1.3.-) (Autolysin sensorkinase)	*Staphylococcus aureus*	Strain Mu50 / ATCC 700699	P60612	--	11418146; 27127614	ATP binding, Hydrolase activity, phosphorelay sensor kinase activity	Not Available
2229	Sensor histidinekinase / phosphatase LytS (EC 2.7.13.3) (EC 3.1.3.-) (Autolysin sensorkinase)	*Staphylococcus aureus*	Strain N315	P60613	--	11418146	ATP binding, Hydrolase activity, phosphorelay sensor kinase activity	Not Available
2230	Sensor histidinekinase / phosphatase LytS (EC 2.7.13.3) (EC 3.1.3.-) (Autolysin sensorkinase)	*Staphylococcus aureus*	Strain MRSA252	Q6GK52	--	15213324	ATP binding, Hydrolase activity, phosphorelay sensor kinase activity	Not Available
2231	Sensor histidinekinase / phosphatase LytS (EC 2.7.13.3) (EC 3.1.3.-) (Autolysin sensorkinase)	*Staphylococcus aureus*	Strain MSSA476	Q6GCL2	--	15213324	ATP binding, Hydrolase activity, phosphorelay sensor kinase activity	Not Available
2232	Sensor histidinekinase / phosphatase LytS (EC 2.7.13.3) (EC 3.1.3.-) (Autolysin sensorkinase)	*Staphylococcus aureus*	Strain MW2	P60614	--	12044378	ATP binding, Hydrolase activity, phosphorelay sensor kinase activity	Not Available
2233	Major fimbrium subunit FimA type-2 (Fimbrillin) (Fimbrilin) (Major fimbrial subunit protein type II)	*Porphyromonas gingivalis*		Q51822	--	7902712; 1987052; 11748193; 23809984	Structural protein	Not Available
2234	Major fimbrium anchoring subunit FimB	*Porphyromonas gingivalis*	Strain ATCC BAA-308 / W83	Q7MT55	--	12949112; 20530728	Not Available	Not Available
2235	Transcriptionalactivator protein AnoR	*Acinetobacter nosocomialis*		A0A0N8YGA2	--	25975610	DNA binding	Not Available
2236	Major fimbrium tip subunit FimD	*Porphyromonas gingivalis*		O32389	--	8981345; 17526848	Host cell extracellular matrix binding	Not Available
2237	Minor curlin subunit	*Escherichia coli*	Strain K12	P0ABK7	--	8817489; 8905232; 9278503; 16738553; 7854117; 17010156; 24097954	Identical protein binding	Not Available
2238	Fimbrial subunit ElfA	*Escherichia coli*	Strain K12	P75855	--	8905232; 9278503; 16738553; 20345943	Not Available	Not Available
2239	Uncharacterized fimbrial-like protein ElfG	*Escherichia coli*	Strain K12	P75858	--	8905232; 9278503; 16738553; 20345943	Not Available	Not Available
2240	Major fimbrium subunit FimA type-1 (FimA1) (Fimbrillin) (Fimbrilin) (Major fimbrial subunit protein type I)	*Porphyromonas gingivalis*		P0C940	--	2895100; 7902712; 8981345; 1987052; 11748193; 20530728; 23809984	Structural protein	Not Available
2241	Probable diguanylate cyclase DgcC (DGC) (EC 2.7.7.65)	*Escherichia coli*	Strain K12	P0AAP1	--	9278503; 16738553; 3533724; 11260463; 16513732; 17010156; 19332833; 19707751; 26148715	Diguanylate cyclase activity, GTP binding, Metal ion binding	Not Available
2242	Probable diguanylate cyclase DgcE (DGC) (EC 2.7.7.65)	*Escherichia coli*	Strain K12	P38097	--	9097040; 9278503; 16738553; 6094528; 7984428; 18765794; 19332833; 20303158; 23708798; 26148715	Diguanylate cyclase activity, GTP binding, Metal ion binding	Not Available
2243	Probable diguanylate cyclase DgcF (DGC) (EC 2.7.7.65)	*Escherichia coli*	Strain K12	P76147	--	9097039; 9278503; 16738553; 21181144; 26148715	Diguanylate cyclase activity, GTP binding, Metal ion binding	Not Available
2244	Probable diguanylate cyclase DgcI (DGC) (EC 2.7.7.65) (Putative lipoprotein DgcI)	*Escherichia coli*	Strain K12	P75801	--	8905232; 9278503; 16738553; 26148715	Diguanylate cyclase activity, GTP binding, Metal ion binding	Not Available
2245	Probable diguanylate cyclase DgcJ (DGC) (EC 2.7.7.65)	*Escherichia coli*	Strain K12	P76237	--	9097040; 9278503; 16738553; 18765794; 19332833; 21181144; 26148715	Diguanylate cyclase activity, GTP binding, Metal ion binding	Not Available
2246	Diguanylate cyclase DgcM (DGC) (EC 2.7.7.65)	*Escherichia coli*	Strain K12	P77302	--	9097039; 9278503; 16738553; 17010156; 19332833; 23708798; 26148715	Diguanylate cyclase activity, GTP binding, Identical protein binding, Metal ion binding	Not Available

Target ID	Bacterial Protein Name	Organism Name	Strain	UniProt ID	PDB ID	PubMed ID	Classification as per UniProt GO Molecular Function	Classification of Function as per PDB
2247	Diguanylate cyclase DgcN (DGC) (EC 2.7.7.65)	*Escherichia coli*	Strain K12	P46139	--	9205837; 9278503; 16738553; 6357787; 7567469; 15919996; 19332833; 20303158; 21181144; 26148715; 27507823	Diguanylate cyclase activity, GTP binding, Identical protein binding, Metal ion binding	Not Available
2248	Diguanylate cyclase DgcP (EC 2.7.7.65)	*Pseudomonas aeruginosa*	Strain ATCC 15692 / DSM 22644 / CIP 104116 / JCM 14847 / LMG 12228 / 1C / PRS 101 / PAO1	Q9HT84	--	10984043; 25118352; 25809128, 2152909	Diguanylate cyclase activity, GTP binding, Metal ion binding	Not Available
2249	Probable diguanylate cyclase DgcQ (DGC) (EC 2.7.7.65) (Cellulose synthesis regulatory protein)	*Salmonella typhimurium*	Strain LT2 / SGSC1412 / ATCC 700720	Q8ZNT5	--	11677609	Diguanylate cyclase activity, GTP binding, Metal ion binding	Not Available
2250	Putative glycosylTransferase EpsH (EC 2.4.-.-)	*Bacillus subtilis*	Strain 168	P71057	--	8969506; 9384377; 15083514; 15175311; 15661000		Not Available
2251	Putative glycosylTransferase EpsD (EC 2.4.-.-)	*Bacillus subtilis*	Strain 168	P71053	--	8969506; 9384377; 15175311; 15661000		Not Available
2252	Minor fimbrium subunit Mfa1 (53 kDa major outer Membrane protein)	*Porphyromonas gingivalis*		P81363	--	9569663; 8208139; 26001707	Not Available	Not Available
2253	Probable diguanylate cyclase CdgI (DGC) (EC 2.7.7.65)	*Escherichia coli*	Strain K12	P76236	--	9097040; 9278503; 16738553; 17010156; 15919996; 21181144; 26148715	Diguanylate cyclase activity, GTP binding, Metal ion binding	Not Available
2254	TransMembrane protein EpsG	*Bacillus subtilis*	Strain 168	P71056	--	8969506; 9384377; 15175311; 15661000	Not Available	Not Available
2255	Fimbrin-like protein FimI	*Salmonella typhimurium*	Strain LT2 / SGSC1412 / ATCC 700720	P37922	--	11677609	Not Available	Not Available
2256	Fimbrial-like protein FimF	*Salmonella typhimurium*	Strain LT2 / SGSC1412 / ATCC 700720	P37926	--	11677609	Not Available	Not Available
2257	Major fimbrium subunit FimC	*Porphyromonas gingivalis*		O32388	--	8981345; 17526848	Host cell extracellular matrix binding	Not Available
2258	CRISPR-associated endonuclease Cas6 / Csy4 (EC 3.1.-.-) (crRNA endonuclease)	*Escherichia coli*	Strain UTI89 / UPEC	Q1RE32	--	16585510; 21398535	Endonuclease activity, RNA binding	Not Available
2259	Fibronectin-binding protein B	*Staphylococcus aureus*	Strain USA300	A0A0H2XKG3	--	16517273; 15234962; 21569203; 24628034; 27387503; 30622139	Not Available	Not Available
2260	Type-1 fimbrial protein, A chain (Type-1A pilin)	*Salmonella typhimurium*	Strain LT2 / SGSC1412 / ATCC 700720	P37921	--	11677609	Not Available	Not Available
2261	DEAD-box ATP-dependent RNA helicase CshA (EC 3.6.4.13)	*Staphylococcus aureus*	Strain NCTC 8325 / PS 47	Q2FWH5	--	21764917; 22447609; 23229022	ATP binding, ATP hydrolysis activity, RNA binding, RNA helicase activity, RNA strand annealing activity	Not Available
2262	ComX pheromone (Competence pheromone)	*Bacillus subtilis subspp. natto*	Strain BEST195	D4G0R3	--	20398357; 25329997; 25855042; 26276536; 29665236	Not Available	Not Available
2263	Putative glycosylTransferase EpsE (EC 2.4.-.-)	*Bacillus subtilis*	Strain 168	P71054	--	8969506; 9384377; 19383706; 15175311; 15661000		Not Available
2264	Putative pyridoxal phosphate-dependent aminoTransferase EpsN (EC 2.6.1.-)	*Bacillus subtilis*	Strain 168	Q795J3	--	8969506; 9384377; 15661000	Pyridoxal phosphate binding, transaminae activity	Not Available
2265	Type 1 fimbrin D-mannose specific adhesin (Protein FimH)	*Salmonella typhimurium*	Strain LT2 / SGSC1412 / ATCC 700720	P37925	--	11677609	Not Available	Not Available
2266	Sensor histidinekinase ArsS (EC 2.7.13.3)	*Helicobacter pylori*	Strain ATCC 700392 / 26695 Campylobacter pylori	O24972	--	9252185; 10735847; 15109719; 16672598; 22865848; 25997502; 27432830	Phosphorelay sensor kinase activity	Not Available
2267	Major fimbrium subunit FimC	*Porphyromonas gingivalis*	Strain ATCC 33277 / DSM 20709 / CIP 103683 / JCM 12257 / NCTC 11834 / 2561	B2RH57	--	18524787; 17675496; 17526848; 19506009	Host cell extracellular matrix binding	Not Available
2268	Response regulator GacA (Global activator)	*Pseudomonas aeruginosa*	Strain ATCC 15692 / DSM 22644 / CIP 104116 / JCM 14847 / LMG 12228 / 1C / PRS 101 / PAO1	Q51373	--	9159518; 10984043, 2152909	DNA binding	Not Available
2269	Major fimbrium tip subunit FimE	*Porphyromonas gingivalis*	Strain ATCC 33277 / DSM 20709 / CIP 103683 / JCM 12257 / NCTC 11834 / 2561	B2RH59	--	18524787; 17675496; 17526848; 19506009	Host cell extracellular matrix binding	Not Available
2270	Long polar fimbria protein A	*Salmonella typhimurium*	Strain LT2 / SGSC1412 / ATCC 700720	P43660	--	7721701; 11677609	Not Available	Not Available

Target ID	Bacterial Protein Name	Organism Name	Strain	UniProt ID	PDB ID	PubMed ID	Classification as per UniProt GO Molecular Function	Classification of Function as per PDB
2271	Putative pyruvyl Transferase EpsO (EC 2.-.-.-)	*Bacillus subtilis*	Strain 168	P71065	--	8969506; 9384377; 15661000	Transferase activity	Not Available
2272	Response regulator protein FleR	*Pseudomonas aeruginosa*	Strain ATCC 15692 / DSM 22644 / CIP 104116 / JCM 14847 / LMG 12228 / 1C / PRS 101 / PAO1	Q9I4N3	--	10984043; 7591148; 8876537; 30877999, 2152909	ATP binding, Sequence-specific DNA binding	Not Available
2273	Major fimbrium anchoring subunit FimB	*Porphyromonas gingivalis*	Strain ATCC 33277 / DSM 20709 / CIP 103683 / JCM 12257 / NCTC 11834 / 2561	A0PA81	--	17081195; 20530728	Not Available	Not Available
2274	Putative glycosylTransferase EpsF (EC 2.4.-.-)	*Bacillus subtilis*	Strain 168	P71055	--	8969506; 9384377; 15175311; 15661000		Not Available
2275	Major fimbrium subunit FimA type-3 (Fimbrillin) (Fimbrilin) (Major fimbrial subunit protein type III)	*Porphyromonas gingivalis*		Q51826	--	7902712; 11748193	Structural protein	Not Available
2276	Major fimbrium subunit FimA type-2 (Fimbrillin) (Fimbrilin) (Major fimbrial subunit protein type II)	*Porphyromonas gingivalis*		Q51825	--	7902712; 23809984	Structural protein	Not Available
2277	Major fimbrium tip subunit FimD	*Porphyromonas gingivalis*	Strain ATCC 33277 / DSM 20709 / CIP 103683 / JCM 12257 / NCTC 11834 / 2561	B2RH58	--	18524787; 17675496; 17526848; 19506009	Host cell extracellular matrix binding	Not Available
2278	AcylTransferase PE (EC 2.3.1.-) (Proline-glutamic acid-rich protein) (PE protein)	*Mycolicibacterium smegmatis*	Strain ATCC 700084 / mc2155 Mycobacterium smegmatis	Q2M5K2	--	17295914; 18955433; 32554804; 27825305; 27028886	AcylTransferase activity	Not Available
2279	RNA polymerase sigma-54 factor	*Pseudomonas aeruginosa*	Strain UCBPP-PA14	A0A0H2ZGR9	--	17038190; 11717271	DNA binding, DNA-binding Transcription activator activity, DNA-directed 5'-3' RNA polymerase activity, sigma factor activity	Not Available
2280	Major fimbrium subunit FimA type-5 (Fimbrillin) (Fimbrilin) (Major fimbrial subunit protein type V)	*Porphyromonas gingivalis*		Q9S0W8	--	10790120; 11748193	Structural protein	Not Available
2281	Pneumolysin (PLY) (Thiol-activated cytolysin)	*Streptococcus pneumoniae serotype 4*	Strain ATCC BAA-334 / TIGR4	P0C2J9	--	11463916; 28456649	Cholesterol binding, toxin activity	Not Available
2282	Rqc2 homolog RqcH (RqcH) (Fibronectin-binding protein YloA)	*Bacillus subtilis subspp. natto*	Strain BEST195	D4FWG0	--	20398357; 25329997; 28294433	Ribosomal large subunit binding, tRNA binding	Not Available
2283	Transcriptional regulatory protein SrrA (Staphylococcal respiratory response protein A)	*Staphylococcus aureus*	Strain MRSA252	Q6GGK6	--	15213324	DNA binding	Not Available
2284	Serine-aspartate repeat-containing protein C	*Staphylococcus aureus*	Strain Newman	O86487	--	9884231; 17951380; 20090838; 25115812; 28320940	Not Available	Not Available
2285	Global Transcriptional regulator Spx	*Staphylococcus aureus*	Strain NCTC 8325 / PS 47	Q2G1U6	--	16788195; 23629700; 32117138	Not Available	Not Available
2286	Transcription antitermiNot Availabletion protein RfaH	*Escherichia coli O6:K15:H31*	Strain 536 / UPEC	Q0TAL4	--	16879640; 16452414	DNA binding, Transcription antitermination factor activity, DNA binding	Not Available
2287	HTH-type Transcriptional regulator MlrA (MerR-like regulator A)	*Escherichia coli*	Strain K12	P33358	--	11489123; 9278503; 16738553; 20874755; 23708798	DNA binding, DNA-binding Transcription factor activity	Not Available
2288	Uncharacterized fimbrial-like protein SfmF	*Escherichia coli*	Strain K12	P38052	--	9278503; 16738553; 2183007; 7984428; 20345943	Not Available	Not Available
2289	Transcriptional regulatory protein SrrA (Staphylococcal respiratory response protein A)	*Staphylococcus aureus*	Strain COL	Q5HFT0	--	15774886; 16859493; 17384184; 24222487	DNA binding	Not Available
2290	Transcriptional regulatory protein SrrA (Staphylococcal respiratory response protein A)	*Staphylococcus aureus*	Strain N315	Q7A5H6	--	11418146; 15590099	DNA binding	Not Available
2291	Uncharacterized fimbrial-like protein YraH	*Escherichia coli*	Strain K12	P42913	--	9278503; 16738553; 20345943	Not Available	Not Available
2292	Uncharacterized fimbrial-like protein SfmH	*Escherichia coli*	Strain K12	P75715	--	9278503; 16738553; 20345943	mannose binding	Not Available
2293	Accessory Sec system protein translocase subunit SecY2	*Streptococcus pneumoniae serotype 4*	Strain ATCC BAA-334 / TIGR4	Q97P79	--	11463916; 16861665; 18507531; 20714350	Not Available	Not Available
2294	Sensor protein SrrB (EC 2.7.13.3) (Staphylococcal respiratory response protein B)	*Staphylococcus aureus*	Strain MW2	Q8NWF3	--	12044378	ATP binding, phosphorelay sensor kinase activity	Not Available
2295	Sensor protein SrrB (EC 2.7.13.3) (Staphylococcal respiratory response protein B)	*Staphylococcus aureus*	Strain N315	Q7A5H7	--	11418146	ATP binding, phosphorelay sensor kinase activity	Not Available
2296	Sensor protein SrrB (EC 2.7.13.3) (Staphylococcal respiratory response protein B)	*Staphylococcus aureus*	Strain MRSA252	Q6GGK7	--	15213324	ATP binding, phosphorelay sensor kinase activity	Not Available
2297	Major fimbrium anchoring subunit FimB	*Porphyromonas gingivalis*		A0PA72	--	17081195; 20530728	Not Available	Not Available
2298	Minor fimbrium subunit Mfa1 (53 kDa fimbrilin)	*Porphyromonas gingivalis*		P0DOA1	--	26001707	Not Available	Not Available
2299	Sensor protein VraS (EC 2.7.13.3)	*Staphylococcus aureus*	Strain MRSA252	Q6GFH2	--	15213324	ATP binding, phosphorelay sensor kinase activity, protein dimerization activity	Not Available
2300	23S rRNA (cytidine-2'-O)-methylTransferase TlyA (EC 2.1.1.226) (23S rRNA (cytidine1920-2'-O)-methylTransferase) (Hemolysin A)	*Campylobacter jejuni subspp. jejuni serotype O:23 / 36*	Strain 81-176	A0A0H3PEK7	--	18389311; 23971210; 24796671; 29404277; 32134554	MethylTransferase activity, RNA binding	Not Available
2301	Sensor protein SrrB (EC 2.7.13.3) (Staphylococcal respiratory response protein B)	*Staphylococcus aureus*	Strain MSSA476	Q6G973	--	15213324	ATP binding, phosphorelay sensor kinase activity	Not Available

Target ID	Bacterial Protein Name	Organism Name	Strain	UniProt ID	PDB ID	PubMed ID	Classification as per UniProt GO Molecular Function	Classification of Function as per PDB
2302	Probable autotransporter YfaL [Cleaved into: Probable secreted autotransporter protein YfaL; Probable autotransporter YfaL translocator]	*Escherichia coli*	Strain K12	P45508	--	9205837; 9278503; 16738553; 6087316; 2834621; 7567469; 16522795; 22344647	serine-type peptidase activity	Not Available
2303	Uncharacterized fimbrial-like protein YadM	*Escherichia coli*	Strain K12	P37018	--	8202364; 9278503; 16738553; 20345943	Not Available	Not Available
2304	Uncharacterized protein YhcK	*Bacillus subtilis*	Strain 168	P54595	--	8969498; 9384377	Diguanylate cyclase activity, phosphorelay sensor kinase activity	Not Available
2305	Uncharacterized fimbrial-like protein YbgD	*Escherichia coli*	Strain K12	P37909	--	8905232; 9278503; 16738553; 7984428	Not Available	Not Available
2306	Uncharacterized fimbrial-like protein YfcQ	*Escherichia coli*	Strain K12	P76500	--	9278503; 16738553; 20345943	Not Available	Not Available
2307	Autotransporter adhesin UpaG (Type 5 secretion system autotransporter YadA)	*Escherichia coli O6:H1*	Strain CFT073 / ATCC 700928 / UPEC	A0A0H2VCA1	--	12471157; 18424525; 22286983	Not Available	Not Available
2308	Uncharacterized fimbrial-like protein YadL	*Escherichia coli*	Strain K12	P37017	--	8202364; 9278503; 16738553; 20345943	Not Available	Not Available
2309	Uncharacterized fimbrial-like protein YcbV	*Escherichia coli*	Strain K12	P75860	--	8905232; 9278503; 16738553; 20345943	Not Available	Not Available
2310	Sensor protein VraS (EC 2.7.13.3)	*Staphylococcus aureus*	Strain MW2	Q7A0H9	--	12044378	ATP binding, phosphorelay sensor kinase activity, protein dimerization activity	Not Available
2311	Uncharacterized fimbrial-like protein YehD	*Escherichia coli*	Strain K12	P33343	--	9278503; 16738553; 20345943	Not Available	Not Available
2312	Uncharacterized fimbrial-like protein YadN	*Escherichia coli*	Strain K12	P37050	--	8202364; 9278503; 16738553; 20345943	Not Available	Not Available
2313	Uncharacterized fimbrial-like protein YgiL	*Escherichia coli*	Strain K12	P39834	--	9278503; 16738553; 1917833; 7984428	Not Available	Not Available
2314	Probable two-component-system connector protein YcgZ	*Escherichia coli*	Strain K12	P75991	--	9278503; 16738553; 14527658; 18174134; 19172264; 19240136	Not Available	Not Available
2315	Uncharacterized fimbrial-like protein YehA	*Escherichia coli*	Strain K12	P33340	--	9097040; 9278503; 16738553; 20345943	Not Available	Not Available
2316	Protein Veg	*Bacillus subtilis*	Strain 168	P37466	--	7584024; 9384377; 6181373; 6790516; 12761295; 23378512	Not Available	Not Available
2317	Uncharacterized fimbrial-like protein YadK	*Escherichia coli*	Strain K12	P37016	--	8202364; 9278503; 16738553; 20345943	Not Available	Not Available
2318	Probable two-component-system connector protein YmgA	*Escherichia coli*	Strain K12	P75992	--	9278503; 16738553; 18174134; 19172264; 19240136	Not Available	Not Available
2319	Sensor protein VraS (EC 2.7.13.3)	*Staphylococcus aureus*	Strain COL	Q5HEN9	--	15774886	ATP binding, phosphorelay sensor kinase activity, protein dimerization activity	Not Available
2320	Sensor protein VraS (EC 2.7.13.3)	*Staphylococcus aureus*	Strain Mu3 / ATCC 700698	Q9KWK8	--	10708580; 17954695	ATP binding, phosphorelay sensor kinase activity, protein dimerization activity	Not Available
2321	Putative iNot Availablective recombiNot Availabletion-promoting nuclease-like protein YjiP	*Escherichia coli*	Strain K12	P0DP21	--	7610040; 9278503; 16738553; 16385049; 26162088; 26618540; 28096446	Not Available	Not Available
2322	Uncharacterized protein YcgV	*Escherichia coli*	Strain K12	P76017	--	9278503; 16738553; 8905232	Not Available	Not Available
2323	Sensor protein VraS (EC 2.7.13.3)	*Staphylococcus aureus*	Strain MSSA476	Q6G849	--	15213324	ATP binding, phosphorelay sensor kinase activity, protein dimerization activity	Not Available
2324	Uncharacterized fimbrial-like protein YfcP	*Escherichia coli*	Strain K12	P76499	--	9278503; 16738553; 20345943	Not Available	Not Available
2325	Uncharacterized fimbrial-like protein YfcR	*Escherichia coli*	Strain K12	P76501	--	9278503; 16738553; 20345943	Not Available	Not Available
2326	Uncharacterized fimbrial-like protein YcbU	*Escherichia coli*	Strain K12	P75859	--	8905232; 9278503; 16738553; 20345943	Not Available	Not Available
2327	Uncharacterized fimbrial-like protein YfcV	*Escherichia coli*	Strain K12	P77288	--	9205837; 9278503; 16738553; 20345943	Not Available	Not Available

Target ID	Bacterial Protein Name	Organism Name	Strain	UniProt ID	PDB ID	PubMed ID	Classification as per UniProt GO Molecular Function	Classification of Function as per PDB
2328	Uncharacterized fimbrial-like protein YdeR	*Escherichia coli*	Strain K12	P77294	--	9097039; 9278503; 16738553	Not Available	Not Available
2329	Uncharacterized fimbrial-like protein YraK	*Escherichia coli*	Strain K12	P43319	--	9278503; 16738553; 20345943	Not Available	Not Available
2330	Uncharacterized protein YtrP	*Bacillus subtilis*	Strain 168	O34325	--	1699930; 9387221; 9384377	Diguanylate cyclase activity	Not Available
2331	AI-2 transport protein TqsA (Transport of quorum-sensing signalprotein)	*Shigella flexneri*		P0AFS6	--	12384590; 12704152	Not Available	Not Available
2332	Uncharacterized protein YeeJ	*Escherichia coli*	Strain K12	P76347	--	9097040; 9278503; 16397293; 16738553	Not Available	Not Available
2333	Sensor protein VraS (EC 2.7.13.3)	*Staphylococcus aureus*	Strain N315	Q99SZ7	--	11418146; 12864861; 21173175; 25899124	ATP binding, phosphorelay sensor kinase activity, protein dimerization activity	Not Available
2334	Uncharacterized fimbrial-like protein YadC	*Escherichia coli*	Strain K12	P31058	--	8202364; 9278503; 16738553; 8096212; 20345943	Not Available	Not Available
2335	Uncharacterized fimbrial-like protein YdeS	*Escherichia coli*	Strain K12	P77789	--	9097039; 9278503; 16738553	Not Available	Not Available
2336	Protein LpfE	*Salmonella typhimurium*	Strain LT2 / SGSC1412 / ATCC 700720	P43664	--	7721701; 11677609	Not Available	Not Available
2337	Transcriptional regulatory protein QseB	*Escherichia coli*	Strain K12	P52076	--	9278503; 16738553; 11929534; 16352847	DNA-binding Transcription activator activity, phosphorelay response regulator activity, Transcription cis-regulatory region binding	Not Available
2338	Extracellular matrix regulatory protein A (Regulator of extracellular matrix A)	*Bacillus subtilis*	Strain 168	Q7WY72	--	9384377; 19363116; 23396918; 23646920	DNA binding	Not Available
2339	Extracellular matrix regulatory protein B (Regulator of the extracellular matrix B)	*Bacillus subtilis*	Strain 168	P37525	--	2987847; 7584024; 9384377; 19383706; 19363116	Not Available	Not Available
2340	Cyclic di-GMP-binding protein (Cellulose synthase regulatory subunit)	*Salmonella typhimurium*	Strain LT2 / SGSC1412 / ATCC 700720	Q93IN1	--	11260463; 11929533; 11677609	Not Available	Not Available
2341	Quorum-quenching protein AidA (Quorum-quenching enzyme) (QQ enzyme)	*Acinetobacter baumannii*	Strain MDR-ZJ06	G2JHL6	--	21788470; 28328989	Hydrolase activity	Not Available
2342	Toxin CdiA (CdiA-EC93)	*Escherichia coli*		Q3YL96	--	16109881; 19124575; 21085179; 23882017; 25174572; 28351921; 30388452	toxin activity	Not Available
2343	Fosfomycin resistance protein AbaF (Acinetobacter baumannii fosfomycin efflux)	*Acinetobacter baumannii*	Strain ATCC 17978 / CIP 53.77 / LMG 1025 / NCDC KC755 / 5377	P0DPR5	--	17344419; 27650185	Transmembrane transporter activity	Not Available
2344	Transcriptional regulatory protein ArsR	*Helicobacter pylori*	Strain ATCC 700392 / 26695 Campylobacter pylori	O24973	--	9252185; 11751811; 16672598; 22865848; 25997502; 27432830	DNA-binding Transcription activator activity, DNA-binding Transcription repressor activity, phosphorelay response regulator activity, Transcription cis-regulatory region binding	Not Available
2345	Uncharacterized Membrane protein EpsK	*Bacillus subtilis*	Strain 168	P71060	--	8969506; 9384377; 19383706; 15661000	Not Available	Not Available
2346	Poly-beta-1, 6-N-acetyl-D-glucosamine synthase (PNot AvailableG synthase) (Poly-beta-1, 6-GlcNAc synthase) (EC 2.4.1.-) (Biofilm polysaccharide intercellular adhesin synthesis protein IcaA) (Biofilm PIA synthesis protein IcaA) (Intercellular adhesion protein A) (N-acetylglucosaminylTransferase IcaA)	*Staphylococcus aureus*	Strain NCTC 8325 / PS 47	Q9RQP9	--	10496925	Acetyl glucosaminyl transferase activity	Not Available
2347	Poly-beta-1, 6-N-acetyl-D-glucosamine synthase (PNot AvailableG synthase) (Poly-beta-1, 6-GlcNAc synthase) (EC 2.4.1.-) (Biofilm polysaccharide intercellular adhesin synthesis protein IcaA) (Biofilm PIA synthesis protein IcaA) (Intercellular adhesion protein A) (N-acetylglucosaminylTransferase IcaA)	*Staphylococcus aureus*	Strain COL	Q5HCN1	--	15774886	Acetyl glucosaminyl transferase activity	Not Available
2348	Poly-beta-1, 6-N-acetyl-D-glucosamine synthase (PNot AvailableG synthase) (Poly-beta-1, 6-GlcNAc synthase) (EC 2.4.1.-) (Biofilm polysaccharide intercellular adhesin synthesis protein IcaA) (Biofilm PIA synthesis protein IcaA) (Intercellular adhesion protein A) (N-acetylglucosaminylTransferase IcaA)	*Staphylococcus aureus*	Strain Mu50 / ATCC 700699	Q99QX3	--	11418146	Acetyl glucosaminyl transferase activity	Not Available
2349	Poly-beta-1, 6-N-acetyl-D-glucosamine synthase (PNot AvailableG synthase) (Poly-beta-1, 6-GlcNAc synthase) (EC 2.4.1.-) (Biofilm polysaccharide intercellular adhesin synthesis protein IcaA) (Biofilm PIA synthesis protein IcaA) (Intercellular adhesion protein A) (N-acetylglucosaminylTransferase IcaA)	*Staphylococcus aureus*	Strain N315	Q7A351	--	11418146	Acetyl glucosaminyl transferase activity	Not Available
2350	Poly-beta-1, 6-N-acetyl-D-glucosamine synthase (PNot AvailableG synthase) (Poly-beta-1, 6-GlcNAc synthase) (EC 2.4.1.-) (Biofilm polysaccharide intercellular adhesin synthesis protein IcaA) (Biofilm PIA synthesis protein IcaA) (Intercellular adhesion protein A) (N-acetylglucosaminylTransferase IcaA)	*Staphylococcus aureus*	Strain MRSA252	Q6GDD8	--	15213324	Acetyl glucosaminyl transferase activity	Not Available

Target ID	Bacterial Protein Name	Organism Name	Strain	UniProt ID	PDB ID	PubMed ID	Classification as per UniProt GO Molecular Function	Classification of Function as per PDB
2351	Poly-beta-1, 6-N-acetyl-D-glucosamine synthase (PNot AvailableG synthase) (Poly-beta-1, 6-GlcNAc synthase) (EC 2.4.1.-) (Biofilm polysaccharide intercellular adhesin synthesis protein IcaA) (Biofilm PIA synthesis protein IcaA) (Intercellular adhesion protein A) (N-acetylglucosaminylTransferase IcaA)	*Staphylococcus aureus*	Strain MSSA476	Q6G608	--	15213324	Acetyl glucosaminyl transferase activity	Not Available
2352	Poly-beta-1, 6-N-acetyl-D-glucosamine synthase (PNot AvailableG synthase) (Poly-beta-1, 6-GlcNAc synthase) (EC 2.4.1.-) (Biofilm polysaccharide intercellular adhesin synthesis protein IcaA) (Biofilm PIA synthesis protein IcaA) (Intercellular adhesion protein A) (N-acetylglucosaminylTransferase IcaA)	*Staphylococcus aureus*	Strain MW2	Q8NUI7	--	12044378	Acetyl glucosaminyl transferase activity	Not Available
2353	Poly-beta-1, 6-N-acetyl-D-glucosamine synthase (PNot AvailableG synthase) (Poly-beta-1, 6-GlcNAc synthase) (EC 2.4.1.-) (Biofilm polysaccharide intercellular adhesin synthesis protein IcaA) (Biofilm PIA synthesis protein IcaA) (Intercellular adhesion protein A) (N-acetylglucosaminylTransferase IcaA)	*Staphylococcus epidermidis*		Q8GLC5	--	15096544	Acetyl glucosaminyl transferase activity	Not Available
2354	Poly-beta-1, 6-N-acetyl-D-glucosamine synthase (PNot AvailableG synthase) (Poly-beta-1, 6-GlcNAc synthase) (EC 2.4.1.-) (Biofilm polysaccharide intercellular adhesin synthesis protein IcaA) (Biofilm PIA synthesis protein IcaA) (Intercellular adhesion protein A) (N-acetylglucosaminylTransferase IcaA)	*Staphylococcus epidermidis*	Strain ATCC 35984 / RP62A	Q5HKQ0	--	8809760; 15774886; 9660830	Acetyl glucosaminyl transferase activity	Not Available
2355	Poly-beta-1, 6-N-acetyl-D-glucosamine N-deacetylase (PNot AvailableG N-deacetylase) (Poly-beta-1, 6-GlcNAc N-deacetylase) (EC 3.5.1.-) (Biofilm polysaccharide intercellular adhesin deacetylase) (Biofilm PIA deacetylase) (Intercellular adhesion protein B)	*Staphylococcus aureus*	Strain NCTC 8325 / PS 47	Q9RQP7	--	10496925	Hydrolase activity, acting on carbon-nitrogen (but not peptide) bonds, in linear amides	Not Available
2356	Poly-beta-1, 6-N-acetyl-D-glucosamine N-deacetylase (PNot AvailableG N-deacetylase) (Poly-beta-1, 6-GlcNAc N-deacetylase) (EC 3.5.1.-) (Biofilm polysaccharide intercellular adhesin deacetylase) (Biofilm PIA deacetylase) (Intercellular adhesion protein B)	*Staphylococcus epidermidis*		Q6TYB1	--	15096544; 15501828	Hydrolase activity, acting on carbon-nitrogen (but not peptide) bonds, in linear amides	Not Available
2357	Poly-beta-1, 6-N-acetyl-D-glucosamine N-deacetylase (PNot AvailableG N-deacetylase) (Poly-beta-1, 6-GlcNAc N-deacetylase) (EC 3.5.1.-) (Biofilm polysaccharide intercellular adhesin deacetylase) (Biofilm PIA deacetylase) (Intercellular adhesion protein B)	*Staphylococcus epidermidis*	Strain ATCC 35984 / RP62A	Q5HKP8	--	8809760; 15774886	Hydrolase activity, acting on carbon-nitrogen (but not peptide) bonds, in linear amides	Not Available
2358	Probable poly-beta-1, 6-N-acetyl-D-glucosamine export protein (PGA export protein) (Poly-beta-1, 6-GlcNAc export protein) (Biofilm polysaccharide intercellular adhesin export protein) (Biofilm PIA export protein) (Intercellular adhesion protein C)	*Staphylococcus aureus*	Strain NCTC 8325 / PS 47	Q9RQP6	--	10496925	Acyltransferase activity, transferring groups other than amino-acyl groups	Not Available
2359	Probable poly-beta-1, 6-N-acetyl-D-glucosamine export protein (PGA export protein) (Poly-beta-1, 6-GlcNAc export protein) (Biofilm polysaccharide intercellular adhesin export protein) (Biofilm PIA export protein) (Intercellular adhesion protein C)	*Staphylococcus epidermidis*		P69518	--	15096544; 15501828	Acyltransferase activity, transferring groups other than amino-acyl groups	Not Available
2360	Probable poly-beta-1, 6-N-acetyl-D-glucosamine export protein (PGA export protein) (Poly-beta-1, 6-GlcNAc export protein) (Biofilm polysaccharide intercellular adhesin export protein) (Biofilm PIA export protein) (Intercellular adhesion protein C)	*Staphylococcus epidermidis*	Strain ATCC 35984 / RP62A	Q5HKP7	--	8809760; 15774886; 9660830	Acyltransferase activity, transferring groups other than amino-acyl groups	Not Available
2361	Poly-beta-1, 6-N-acetyl-D-glucosamine synthesis protein IcaD (PGA synthesis protein IcaD) (Poly-beta-1, 6-GlcNAc synthesis protein IcaD) (Biofilm polysaccharide intercellular adhesin synthesis protein IcaD) (Biofilm PIA synthesis protein IcaD) (Intercellular adhesion protein D)	*Staphylococcus aureus*	Strain NCTC 8325 / PS 47	Q9RQP8	--	10496925	Not Available	Not Available
2362	Biofilm operon icaADBC HTH-type negative Transcriptional regulator IcaR (Intercellular adhesion protein R)	*Staphylococcus aureus*	Strain NCTC 8325 / PS 47	Q9RQQ0	--	10496925	DNA-binding Transcription factor activity, Transcription cis-regulatory region binding	Not Available
2363	signalpeptidase I W (SPase I) (EC 3.4.21.89) (Leader peptidase I)	*Bacillus subtilis*	Strain 168	P54506	--	8969508; 9384377; 9694797; 9823656; 10049401; 10464223; 10559173; 16430695; 23646920	Serine-type endopeptidase activity	Not Available
2364	Endoglucanae (EC 3.2.1.4) (Carboxymethylcellulase) (CMCase) (Cellulase) (Endo-1, 4-beta-glucanae)	*Salmonella typhimurium*	Strain LT2 / SGSC1412 / ATCC 700720	Q8ZLB7	--	11260463; 11929533; 11677609	Sellulase activity	Not Available
2365	Major fimbrium subunit FimA type-4 (Fimbrillin) (Fimbrilin) (Major fimbrial subunit protein type IV)	*Porphyromonas gingivalis*		Q51827	--	7902712; 7851739; 1987052; 11748193	Structural protein	Not Available
2366	Uncharacterized sugar Transferase EpsL (EC 2.-.-.-)	*Bacillus subtilis*	Strain 168	P71062	--	8969506; 9384377; 15661000	PhosphoTransferase activity, for other substituted phosphate groups	Not Available
2367	Biofilm architecture maintece protein MbaA	*Vibrio cholerae serotype O1*	Strain ATCC 39315 / El Tor Iba N16961	Q9KU26	--	10952301; 12562809; 16237027	Not Available	Not Available
2368	Cellulose synthase catalytic subunit [UDP-forming] (EC 2.4.1.12)	*Salmonella typhimurium*	Strain LT2 / SGSC1412 / ATCC 700720	Q93IN2	--	11260463; 11929533; 11677609	Cellulose synthase (UDP-forming) activity, cyclic-di-GMP binding, hexosylTransferase activity	Not Available
2369	CsgBAC operon Transcriptional regulatory protein	*Escherichia coli*	Strain K12	P52106	--	8817489; 8905232; 9278503; 16738553; 16513732; 17010156; 24212724	DNA-binding Transcription activator activity, DNA-binding Transcription repressor activity, Identical protein binding, Transcription cis-regulatory region binding	Not Available
2370	Surface composition regulator	*Escherichia coli O139:H28*	Strain E24377A / ETEC	A7ZRT0	--	18676672	Not Available	Not Available

Target ID	Bacterial Protein Name	Organism Name	Strain	UniProt ID	PDB ID	PubMed ID	Classification as per UniProt GO Molecular Function	Classification of Function as per PDB
2371	Surface composition regulator	*Escherichia coli O127:H6*	Strain E2348 / 69 / EPEC	B7UIV7	--	18952797	Not Available	Not Available
2372	Surface composition regulator	*Escherichia coli O45:K1*	Strain S88 / ExPEC	B7MAC5	--	19165319	Not Available	Not Available
2373	Surface composition regulator	*Escherichia coli*	Strain 55989 / EAEC	B7LGY3	--	19165319	Not Available	Not Available
2374	Surface composition regulator	*Escherichia coli O157:H7*		P64244	--	11206551; 11258796	Not Available	Not Available
2375	Surface composition regulator	*Escherichia coli O157:H7*	Strain EC4115 / EHEC	B5YR89	--	22135463	Not Available	Not Available
2376	Surface composition regulator	*Escherichia coli O7:K1*	Strain IAI39 / ExPEC	B7NJR2	--	19165319	Not Available	Not Available
2377	Surface composition regulator	*Escherichia coli O81*	Strain ED1a	B7N052	--	19165319	Not Available	Not Available
2378	Surface composition regulator	*Escherichia coli O8*	Strain IAI1	B7LZJ8	--	19165319	Not Available	Not Available
2379	Surface composition regulator	*Escherichia coli*	Strain K12 / MC4100 / BW2952	C4ZQW6	--	19376874	Not Available	Not Available
2380	Surface composition regulator	*Escherichia coli*	Strain K12 / DH10B	B1XG54	--	18245285	Not Available	Not Available
2381	Surface composition regulator	*Escherichia coli O9:H4*	Strain HS	A8A4K4	--	18676672	Not Available	Not Available
2382	Surface composition regulator	*Escherichia coli O6:K15:H31*	Strain 536 / UPEC	Q0TD58	--	16879640	Not Available	Not Available
2383	Surface composition regulator	*Escherichia coli O6:H1*	Strain CFT073 / ATCC 700928 / UPEC	P64243	--	12471157	Not Available	Not Available
2384	Surface composition regulator	*Escherichia coli O17:K52:H18*	Strain UMN026 / ExPEC	B7ND37	--	19165319	Not Available	Not Available
2385	Surface composition regulator	*Escherichia coli*	Strain SE11	B6I419	--	18931093	Not Available	Not Available
2386	Surface composition regulator	*Escherichia coli*	Strain SMS-3-5 / SECEC	B1LF40	--	18708504	Not Available	Not Available
2387	Surface composition regulator	*Escherichia coli*	Strain UTI89 / UPEC	Q1R6T2	--	16585510	Not Available	Not Available
2388	Surface composition regulator	*Salmonella choleraesuis*	Strain SC-B67	Q57JR3	--	15781495	Not Available	Not Available
2389	Surface composition regulator	*Salmonella enteritidis PT4*	Strain P125109	B5QZ33	--	18583645	Not Available	Not Available
2390	Surface composition regulator	*Salmonella paratyphi A*	Strain ATCC 9150 / SARB42	Q5PC89	--	15531882	Not Available	Not Available
2391	Surface composition regulator	*Salmonella paratyphi A*	Strain AKU_12601	B5BG08	--	19159446	Not Available	Not Available
2392	Surface composition regulator	*Salmonella typhi*		P58615	--	11677608; 12644504	Not Available	Not Available
2393	Surface composition regulator	*Salmonella typhimurium*	Strain LT2 / SGSC1412 / ATCC 700720	P58614	--	11677609	Not Available	Not Available
2394	Surface composition regulator	*Shigella dysenteriae serotype 1*	Strain Sd197	Q32BR9	--	16275786	Not Available	Not Available
2395	Surface composition regulator	*Shigella flexneri*		Q7UBJ0	--	12384590; 12704152	Not Available	Not Available
2396	Surface composition regulator	*Shigella sonnei*	Strain SS046	Q3YXJ3	--	16275786	Not Available	Not Available
2397	Cellulose synthase operon protein C	*Salmonella typhimurium*	Strain LT2 / SGSC1412 / ATCC 700720	Q8ZLB8	--	11260463; 11929533; 11677609	Not Available	Not Available
2398	Major fimbrium tip subunit FimE	*Porphyromonas gingivalis*		O32390	--	8981345; 17526848	Host cell extracellular matrix binding	Not Available
2399	Probable diguanylate cyclase DgcQ (DGC) (EC 2.7.7.65) (Cellulose synthesis regulatory protein)	*Escherichia coli*	Strain K12	P76330	--	9097040; 9278503; 16738553; 16585767; 17010156; 19332833; 20303158; 21181144; 26148715	Diguanylate cyclase activity, GTP binding, Metal ion binding	Not Available
2400	Autotransporter adhesin EhaG (Type 5 secretion system autotransporter EhaG)	*Escherichia coli O157:H7*		Q7DJ60	--	11206551; 11258796; 27056233; 22286983	Not Available	Not Available
2401	Uncharacterized glycosylTransferase EpsJ (EC 2.4.-.-)	*Bacillus subtilis*	Strain 168	P71059	--	8969506; 9384377; 15661000		Not Available
2402	Putative pyruvyl Transferase EpsI (EC 2.-.-.-)	*Bacillus subtilis*	Strain 168	P71058	--	8969506; 9384377; 15175311; 15661000	Transferase activity	Not Available
2403	Hemin storage protein	*Lysobacter arseniciresistens ZS79*		A0A0A0F1X8	--	26516404	Hydrolase activity, acting on carbon-nitrogen (but not peptide) bonds	Not Available
2404	Poly-beta-1, 6-N-acetyl-D-glucosamine synthase (Poly-beta-1, 6-GlcNAc synthase) (EC 2.4.1.-)	*Lysobacter arseniciresistens ZS79*		A0A0A0EWS4	--	26516404	Acetyl glucosaminyl transferase activity	Not Available
2405	Type II toxin-antitoxin system MqsR family toxin	*Pseudomonas panacis*		A0A7Y1IL75	--	32587583	Not Available	Not Available
2406	Poly-beta-1, 6-N-acetyl-D-glucosamine N-deacetylase PgaB	*Desulfobulbaceae bacterium*		A0A7V5KUV9	--	31911466	Hydrolase activity, acting on carbon-nitrogen (but not peptide) bonds	Not Available
2407	Poly-beta-1, 6-N-acetyl-D-glucosamine synthase (Poly-beta-1, 6-GlcNAc synthase) (EC 2.4.1.-)	*Desulfobulbaceae bacterium*		A0A7V5V5A8	--	31911466	Acetyl glucosaminyl transferase activity	Not Available
2408	Toxin mRNA interferase YgiU	*Pseudomonas furukawaii*		L8MLK2	--	22843571	Not Available	Not Available
2409	Poly-beta-1, 6-N-acetyl-D-glucosamine synthase (Poly-beta-1, 6-GlcNAc synthase) (EC 2.4.1.-)	*Desulfobulbaceae bacterium*		A0A7V5FPJ1	--	31911466	Acetyl glucosaminyl transferase activity	Not Available
2410	Poly-beta-1, 6-N-acetyl-D-glucosamine biosynthesis protein PgaD	*Desulfobulbaceae bacterium*		A0A7V5FPJ3	--	31911466	Not Available	Not Available
2411	Poly-beta-1, 6-N-acetyl-D-glucosamine N-deacetylase PgaB	*Desulfobulbaceae bacterium*		A0A7V5KV26	--	31911466	Hydrolase activity, acting on carbon-nitrogen (but not peptide) bonds	Not Available
2412	Poly-beta-1, 6-N-acetyl-D-glucosamine synthase (Poly-beta-1, 6-GlcNAc synthase) (EC 2.4.1.-)	*Pseudomonas panacis*		A0A7Y1IRF0	--	32587583	Acetyl glucosaminyl transferase activity	Not Available
2413	Poly-beta-1, 6-N-acetyl-D-glucosamine synthase (Poly-beta-1, 6-GlcNAc synthase) (EC 2.4.1.-)	*Aeromona salmonicida*	Strain A449	A4SIQ6	--	18801193	Acetyl glucosaminyl transferase activity	Not Available
2414	Surface composition regulator	*Cronobacter sakazakii 696*		K8CNU6	--	23166675	Not Available	Not Available
2415	Poly-beta-1, 6-N-acetyl-D-glucosamine biosynthesis protein PgaD	*Pseudomonas panacis*		A0A7Y1IRD1	--	32587583	Not Available	Not Available
2416	Poly-beta-1, 6-N-acetyl-D-glucosamine biosynthesis protein PgaD	*Desulfobulbaceae bacterium*		A0A7V5V4Z0	--	31911466	Not Available	Not Available
2417	Poly-beta-1, 6-N-acetyl-D-glucosamine biosynthesis protein PgaD (Fragment)	*Enterobacter spp.*		A0A3D2B4K2	--	30148503	Not Available	Not Available
2418	Poly-beta-1, 6-N-acetyl-D-glucosamine synthase (Poly-beta-1, 6-GlcNAc synthase) (EC 2.4.1.-)	*Acinetobacter wuhouensis*		A0A385C658	--	30117799	Acetyl glucosaminyl transferase activity	Not Available
2419	Uncharacterized protein	*Photorhabdus temperata subspp. temperata M1021*		T0PA00	--	24029767	Not Available	Not Available
2420	Biofilm PGA synthesis deacetylase PgaB	*Yersinia enterocolitica subspp. palearctica serotype O:3*	Strain DSM 13030 / CIP 106945 / Y11	A0A0H3NVK7	--	21296963	Hydrolase activity, acting on carbon-nitrogen (but not peptide) bonds	Not Available
2421	Biofilm PGA synthesis auxiliary protein PgaD	*Yersinia enterocolitica subspp. palearctica serotype O:3*	Strain DSM 13030 / CIP 106945 / Y11	A0A0H3NSA5	--	21296963	Not Available	Not Available
2422	Uncharacterized protein	*Rhodocyclales bacterium GWA2_65_19*		A0A1G3H3U9	--	27774985	Not Available	Not Available

Target ID	Bacterial Protein Name	Organism Name	Strain	UniProt ID	PDB ID	PubMed ID	Classification as per UniProt GO Molecular Function	Classification of Function as per PDB
2423	Uncharacterized protein	*Syntrophomona wolfei subspp. wolfei*	Strain DSM 2245B / Goettingen	Q0AU88	--	21966920	Not Available	Not Available
2424	Toxin mRNA interferase YgiU	*Yersinia enterocolitica subspp. palearctica serotype O:3*	Strain DSM 13030 / CIP 106945 / Y11	A0A0H3NTG7	--	21296963	Not Available	Not Available
2425	DUF1471 domain-containing protein (Multiple stress resistance protein BhsA) (Outer Membrane protein) (Putative outer Membrane protein)	*Salmonella typhimurium*		A0A0F7J832	--	26089426; 30286803	Not Available	Not Available
2426	Surface composition regulator	*Serratia marcescens*		A0A0F6KTF3	--	28536292	Not Available	Not Available
2427	Surface composition regulator	*Serratia marcescens*		A0A0A5LSU7	--	28536292	Not Available	Not Available
2428	Poly-beta-1, 6-N-acetyl-D-glucosamine biosynthesis protein PgaD	*Serratia marcescens*		A0A2V4G8U1	--	21967170; 30286803	Not Available	Not Available
2429	Poly-beta-1, 6-N-acetyl-D-glucosamine synthase (Poly-beta-1, 6-GlcNAc synthase) (EC 2.4.1.-)	*Pseudomonas yamanorum*		A0A145XGR5	--	27231373	Acetyl glucosaminyl transferase activity	Not Available
2430	Surface composition regulator	*Salmonella enterica subspp. enterica serovar*	Poona	A0A4Z0KU92	--	30286803	Not Available	Not Available
2431	Poly-beta-1, 6-N-acetyl-D-glucosamine synthase (Poly-beta-1, 6-GlcNAc synthase) (EC 2.4.1.-)	*Acinetobacter venetianus*		A0A150HR71	--	26902269	Acetyl glucosaminyl transferase activity	Not Available
2432	Biofilm PGA synthesis auxiliary protein PgaD (Biofilm PGA synthesis protein PgaD) (Poly-beta-1, 6-N-acetyl-D-glucosamine biosynthesis protein PgaD)	*Pseudomonas yamanorum*		A0A145XGQ4	--	27231373	Not Available	Not Available
2433	Toxin CdiA	*Neisseria meningitidis*		Q9JPE3	--	10722605; 19289515; 28505234; 30485280; 10722605	Toxin activity	Not Available
2434	Surface composition regulator	*Salmonella typhimurium*		A0A0J5E264	--	30286803	Not Available	Not Available
2435	Surface composition regulator	*Serratia marcescens*		A0A7D6C0Q8	--	21967170; 30286803	Not Available	Not Available
2436	Poly-beta-1, 6-N-acetyl-D-glucosamine synthase (Poly-beta-1, 6-GlcNAc synthase) (EC 2.4.1.-)	*Serratia marcescens*		A0A7D6BXB5	--	21967170; 30286803	Acetyl glucosaminyl transferase activity	Not Available
2437	Poly-beta-1, 6-N-acetyl-D-glucosamine synthase (Poly-beta-1, 6-GlcNAc synthase) (EC 2.4.1.-)	*Serratia marcescens*		A0A0A5LT03	--	28536292; 28636609	Acetyl glucosaminyl transferase activity	Not Available
2438	Poly-beta-1, 6-N-acetyl-D-glucosamine biosynthesis protein PgaD	*Acinetobacter seifertii*		N8QXR6	--	25563912	Not Available	Not Available
2439	Poly-beta-1, 6-N-acetyl-D-glucosamine synthase (Poly-beta-1, 6-GlcNAc synthase) (EC 2.4.1.-)	*Acinetobacter seifertii*		N8QY22	--	25563912	Acetyl glucosaminyl transferase activity	Not Available
2440	Poly-beta-1, 6-N-acetyl-D-glucosamine N-deacetylase (Poly-beta-1, 6-N-acetyl-D-glucosamine N-deacetylase PgaB)	*Serratia marcescens*		A0A0G3SWE7	--	28636609	Hydrolase activity, acting on carbon-nitrogen (but not peptide) bonds	Not Available
2441	Guanosine-3', 5'-bis(diphosphate) 3'-diphosphatase (EC 3.1.7.2)	*Salmonella enteritidis*		K0A192	--	30286803	Guanosine-3', 5'-bis(diphosphate) 3'-diphosphatase activity, kinase activity	Not Available
2442	Poly-beta-1, 6-N-acetyl-D-glucosamine synthase (Poly-beta-1, 6-GlcNAc synthase) (EC 2.4.1.-)	*Acinetobacter seifertii*		N8S570	--	25563912	Acetyl glucosaminyl transferase activity	Not Available
2443	Surface composition regulator	*Salmonella enterica subspp. enterica serovar*	Heidelberg	A0A265B3W8	--	30286803	Not Available	Not Available
2444	Type II toxin-antitoxin system MqsR family toxin	*Pseudomonas putida*	Arthrobacter siderocapsulatus	A0A550F6I7	--	19827713	Not Available	Not Available
2445	Surface composition regulator	*Salmonella enterica subspp. enterica serovar*	Typhimurium var. 5-	A0A5W1BAQ5	--	30286803	Not Available	Not Available
2446	Surface composition regulator	*Salmonella enterica subspp. arizonae*		A0A3Z4JNS2	--	30286803	Not Available	Not Available
2447	Surface composition regulator	*Salmonella paratyphi A*		A0A3U5EDJ2	--	22190395; 30286803	Not Available	Not Available
2448	Flagellin	*Acidovorax citrulli*	Acidovorax avenae subspp. citrulli	A5HIK5	--	20807236; 21554180	Structural protein	Not Available
2449	Poly-beta-1, 6-N-acetyl-D-glucosamine biosynthesis protein PgaD	*Deltaproteobacteria bacterium HGW-Deltaproteobacteria-4*		A0A2N2FUX1	--	28350393	Not Available	Not Available
2450	Toxin mRNA interferase YgiU	*Yersinia enterocolitica subspp. palearctica serotype O:3*	Strain YE-P4	A0A7H4ZBT2	--	23846271	Not Available	Not Available
2451	Poly-beta-1, 6-N-acetyl-D-glucosamine synthase (Poly-beta-1, 6-GlcNAc synthase) (EC 2.4.1.-)	*Yersinia enterocolitica subspp. palearctica serotype O:3*	Strain YE-P4	A0A7H4ZKR9	--	23846271	Acetyl glucosaminyl transferase activity	Not Available
2452	Type II toxin-antitoxin system MqsR family toxin	*Pseudomonas spp. WS 5532*		A0A7Y0Z345	--	32587583	Not Available	Not Available
2453	Poly-beta-1, 6-N-acetyl-D-glucosamine biosynthesis protein PgaD	*Pseudomonas spp. WS 5532*		A0A7Y0Z6M5	--	32587583	Not Available	Not Available
2454	Poly-beta-1, 6-N-acetyl-D-glucosamine synthase (Poly-beta-1, 6-GlcNAc synthase) (EC 2.4.1.-)	*Acinetobacter spp. ACNIH4*		A0A2S4TAE5	--	29437920	Acetyl glucosaminyl transferase activity	Not Available
2455	Surface composition regulator	*Yersinia enterocolitica subspp. palearctica serotype O:3*	Strain YE-P4	A0A7H4ZIG3	--	23846271	DNA binding, DNA-directed DNA polymerase activity	Not Available
2456	Poly-beta-1, 6-N-acetyl-D-glucosamine synthase (Poly-beta-1, 6-GlcNAc synthase) (EC 2.4.1.-)	*Pseudomonas spp. WS 5532*		A0A7Y0Z6L1	--	32587583	Acetyl glucosaminyl transferase activity	Not Available
2457	Poly-beta-1, 6-N-acetyl-D-glucosamine N-deacetylase PgaB	*Deltaproteobacteria bacterium HGW-Deltaproteobacteria-4*		A0A2N2FUZ1	--	28350393	Hydrolase activity, acting on carbon-nitrogen (but not peptide) bonds	Not Available
2458	Surface composition regulator	*Cronobacter dublinensis 1210*		K8ANJ5	--	23166675	Not Available	Not Available
2459	Poly-beta-1, 6-N-acetyl-D-glucosamine synthase (Poly-beta-1, 6-GlcNAc synthase) (EC 2.4.1.-)	*Deltaproteobacteria bacterium HGW-Deltaproteobacteria-4*		A0A2N2FUX4	--	28350393	Acetyl glucosaminyl transferase activity	Not Available
2460	Poly-beta-1, 6-N-acetyl-D-glucosamine N-deacetylase PgaB (EC 3.5.1.-)	*Pseudomonas spp. WS 5532*		A0A7Y0Z6L3	--	32587583	Hydrolase activity, acting on carbon-nitrogen (but not peptide) bonds	Not Available
2461	Hemin storage system protein	*Yersinia enterocolitica subspp. palearctica serotype O:3*	Strain YE-P4	A0A7H4ZKV9	--	23846271	Not Available	Not Available
2462	mRNA interferase MqsR	*Photorhabdus thracensis*		A0A0F7LS53	--	26415660	Not Available	Not Available
2463	Type II toxin-antitoxin system MqsR family toxin	*Rhizobium spp.*		A0A3D3BHM1	--	30148503	Not Available	Not Available
2464	Secreted biofilm formation protein YqxM	*Bacillus licheniformis*	Strain ATCC 14580 / DSM 13 / JCM 2505 / CCUG 7422 / NBRC 12200 / NCIMB 9375 / NCTC 10341 / NRRL NRS-1264 / Gibson 46	Q65HF1	--	15461803	Not Available	Not Available
2465	Surface composition regulator	*Enterobacteriaceae bacterium ENNIH1*		A0A2I8Q9C8	--	29437920	Not Available	Not Available
2466	Poly-beta-1, 6-N-acetyl-D-glucosamine biosynthesis protein PgaD	*Acinetobacter spp. ACNIH4*		A0A2S4TAG1	--	29437920	Not Available	Not Available

Target ID	Bacterial Protein Name	Organism Name	Strain	UniProt ID	PDB ID	PubMed ID	Classification as per UniProt GO Molecular Function	Classification of Function as per PDB
2467	mRNA interferase MqsR	*Photorhabdus khanii subspp. guanajuatensis*		A0A4R4JWB8	--	30688647	Not Available	Not Available
2468	Poly-beta-1, 6-N-acetyl-D-glucosamine biosynthesis protein PgaD	*Stenotrophomonas spp. ESTM1D_MKCIP4_1*		A0A3Q8FUZ0	--	29765358	Not Available	Not Available
2469	Poly-beta-1, 6-N-acetyl-D-glucosamine synthase (Poly-beta-1, 6-GlcNAc synthase) (EC 2.4.1.-)	*Stenotrophomonas spp. ESTM1D_MKCIP4_1*		A0A3S7KXH2	--	29765358	Acetyl glucosaminyl transferase activity	Not Available
2470	Type II toxin-antitoxin system MqsR family toxin	*Rhizobium radiobacter*	Agrobacterium tumefaciens Agrobacterium radiobacter	A0A546XJ46	--	31273398	Not Available	Not Available
2471	Uncharacterized protein	*Alicyclobacillus acidocaldarius subspp. acidocaldarius*	Strain ATCC 27009 / DSM 446 / BCRC 14685 / JCM 5260 / KCTC 1825 / NBRC 15652 / NCIMB 11725 / NRRL B-14509 / 104-IA Bacillus acidocaldarius	C8WT26	--	21304673	Not Available	Not Available
2472	Biofilm PGA synthesis protein PgaD	*Escherichia coli O157:H7*	Strain EC869	A0A0H3PN65	--	21421787	Not Available	Not Available
2473	Poly-beta-1, 6-N-acetyl-D-glucosamine biosynthesis protein PgaD	*Candidatus Manganitrophus noduliformans*		A0A7X6DPU5	--	32669693	Not Available	Not Available
2474	Poly-beta-1, 6-N-acetyl-D-glucosamine biosynthesis protein PgaD	*Stenotrophomonas spp. ESTM1D_MKCIP4_1*		A0A3S5GHZ3	--	29765358	Not Available	Not Available
2475	Poly-beta-1, 6-N-acetyl-D-glucosamine N-deacetylase PgaB	*Acinetobacter spp. ACNIH2*		A0A2L0HWN8	--	29437920	Hydrolase activity, acting on carbon-nitrogen (but not peptide) bonds	Not Available
2476	Uncharacterized protein	*Desulfovibrio magneticus*	Strain ATCC 700980 / DSM 13731 / RS-1	C4XJ49	--	19675025	Not Available	Not Available
2477	Poly-beta-1, 6-N-acetyl-D-glucosamine synthase (Poly-beta-1, 6-GlcNAc synthase) (EC 2.4.1.-)	*Aeromona sobria*		A0A2N3IZW3	--	29276504	Acetyl glucosaminyl transferase activity	Not Available
2478	Poly-beta-1, 6-N-acetyl-D-glucosamine biosynthesis protein PgaD (Fragment)	*Oceanospirillales bacterium*		A0A7V2SRU5	--	31911466	Not Available	Not Available
2479	Chitin deacetylase	*Aeromona sobria*		A0A2N3IZH4	--	29276504	Hydrolase activity, acting on carbon-nitrogen (but not peptide) bonds	Not Available
2480	Uncharacterized protein	*Candidatus Eisenbacteria bacterium*		A0A538U948	--	31110364	Not Available	Not Available
2481	Poly-beta-1, 6-N-acetyl-D-glucosamine synthase (Poly-beta-1, 6-GlcNAc synthase) (EC 2.4.1.-)	*Stenotrophomonas spp. ESTM1D_MKCIP4_1*		A0A3S5GHV1	--	29765358	Acetyl glucosaminyl transferase activity	Not Available
2482	Surface composition regulator	*Salmonella typhimurium*	Strain 4 / 74	E8XBC6	--	21478351	Not Available	Not Available
2483	Surface composition regulator	*Escherichia coli O157:H7*	Strain EC869	A0A0H3PT67	--	21421787	Not Available	Not Available
2484	Poly-beta-1, 6-N-acetyl-D-glucosamine biosynthesis protein PgaD	*Methylococcaceae bacterium*		A0A351V596	--	30148503	Not Available	Not Available
2485	Biofilm PGA synthesis lipoprotein PgaB	*Pectobacterium parmentieri*		A0A0H3ICP1	--	23045508	Hydrolase activity, acting on carbon-nitrogen (but not peptide) bonds	Not Available
2486	Hemin storage system, HmsS protein	*Pectobacterium parmentieri*		A0A0H3IBQ4	--	23045508	Not Available	Not Available
2487	Poly-beta-1, 6-N-acetyl-D-glucosamine N-deacetylase PgaB	*Pseudomonas spp.*		A0A3D4NT91	--	30148503	Hydrolase activity, acting on carbon-nitrogen (but not peptide) bonds	Not Available
2488	Poly-beta-1, 6-N-acetyl-D-glucosamine biosynthesis protein PgaD	*Xenorhabdus budapestensis*		A0A2D0J1F6	--	28993611	Not Available	Not Available
2489	Polysaccharide deacetylase	*Xenorhabdus budapestensis*		A0A2D0J1D9	--	28993611	Hydrolase activity, acting on carbon-nitrogen (but not peptide) bonds	Not Available
2490	Poly-beta-1, 6-N-acetyl-D-glucosamine synthase (Poly-beta-1, 6-GlcNAc synthase) (EC 2.4.1.-)	*Methylococcaceae bacterium*		A0A351KVS7	--	30148503	Acetyl glucosaminyl transferase activity	Not Available
2491	Surface composition regulator	*Salmonella enteritidis*		A0A0M0QID5	--	30286803	Not Available	Not Available
2492	Surface composition regulator	*Salmonella enterica subspp. enterica serovar*	Agona	A0A2T9HZU6	--	30286803	Not Available	Not Available
2493	Surface composition regulator	*Salmonella infantis*		A0A402RIU0	--	30286803; 31476731	Not Available	Not Available
2494	Poly-beta-1, 6-N-acetyl-D-glucosamine biosynthesis protein PgaD	*Serratia marcescens*		A0A658C2L5	--	28636609	Not Available	Not Available
2495	Surface composition regulator	*Salmonella enterica subspp. enterica serovar*	Indiana	A0A4Y6N1C4	--	30286803	Not Available	Not Available
2496	Surface composition regulator	*Pectobacterium brasiliense*		A0A086EFR3	--	32193231	DNA binding, DNA-directed DNA polymerase activity	Not Available
2497	Poly-beta-1, 6-N-acetyl-D-glucosamine biosynthesis protein PgaD	*Pectobacterium brasiliense*	Pseudomonas maltophilia Xanthomonas maltophilia	A0A2J0SHJ3	--	29170656	Not Available	Not Available
2498	Poly-beta-1, 6-N-acetyl-D-glucosamine biosynthesis protein PgaD	*Stenotrophomonas maltophilia*	Pseudomonas maltophilia Xanthomonas maltophilia	A0A1S2B2J2	--	29170656	Not Available	Not Available
2499	Surface composition regulator	*Salmonella houtenae*		A0A5Y6MAD3	--	30286803	Not Available	Not Available
2500	Surface composition regulator	*Salmonella typhimurium*		A0A0F7JA38	--	26089426	Not Available	Not Available
2501	Poly-beta-1, 6-N-acetyl-D-glucosamine synthase (Poly-beta-1, 6-GlcNAc synthase) (EC 2.4.1.-)	*Burkholderia ambifaria*	Strain ATCC BAA-244 / AMMD Burkholderia cepacia Strain AMMD	Q0B9V6	--	25931592	Acetyl glucosaminyl transferase activity	Not Available
2502	S-ribosylhomocysteine lyase (EC 4.4.1.21) (AI-2 synthesis protein) (Autoinducer-2 production protein LuxS)	*Lacticaseibacillus rhamnosus*	Lactobacillus rhamnosus	A1XC58	--	17098890; 31477907	Iron ion binding, S-ribosylhomocysteine lyase activity	Not Available
