# Supplementary material for: 'Targeting' the search: An upgraded structural and functional repository of antimicrobial peptides for biofilm studies (B-AMP v2.0) with a focus on biofilm protein targets
Source: Front Cell Infect Microbiol. 2022 Oct 18;12:1020391. doi: 10.3389/fcimb.2022.1020391 (PMC9623296; doi:10.3389/fcimb.2022.1020391)
Supplement: Supplementary Table 2 — Distribution of biofilm targets in B-AMP v2.0 based on bacterial class, family, genera or description as per NCBI Taxonomy. [file Table_2.pdf]

Supplementary Table 2: Distribution of biofilm targets in B-AMP v2.0 based on bacterial class, family, genera or description as per NCBI Taxonomy

Serial Number	Bacterial Class, Family, Genus or Description as per NCBI Taxonomy	Count
1	*Pseudomonas*	315
2	*Escherichia*	259
3	*Salmonella*	216
4	*Acinetobacter*	160
5	*Staphylococcus*	123
6	*Stenotrophomonas*	79
7	*Klebsiella*	70
8	*Yersinia*	69
9	*Xanthomonas*	49
10	*Enterobacter*	48
11	*Bacillus*	46
12	*Vibrio*	31
13	*Deltaproteobacteria*	27
14	*Xenorhabdus*	27
15	*Bordetella*	26
16	*Citrobacter*	26
17	*Burkholderia*	25
18	*Shewanella*	25
19	*Candidatus*	23
20	*Gammaproteobacteria*	22
21	*Serratia*	22
22	*Ralstonia*	21
23	*Lysobacter*	18
24	*Pectobacterium*	18
25	*Lentisphaerae*	17
26	*Porphyromona*	17
27	*Achromobacter*	15
28	*Enterobacteriaceae*	15
29	*Streptococcus*	15
30	*Geobacter*	14
31	*Leptospira*	14
32	*Paraburkholderia*	12
33	*Aeromonas*	11
34	*Bradyrhizobium*	11
35	*Porphyromonas*	11
36	*Shigella*	11
37	*Actinobacillus*	10
38	*Chromobacterium*	10
39	*Cronobacter*	10
40	*Cupriavidus*	10
41	*Xanthomonadaceae*	10

42	*Clostridium*	9
43	*Comamonae*	9
44	*Moraxellaceae*	9
45	*Sulfurimona*	9
46	*Aggregatibacter*	8
47	*Chloroflexus*	8
48	*Deinococcus*	8
49	*Edwardsiella*	8
50	*Lautropia*	8
51	*Synechocystis*	8
52	*Gallionellales*	7
53	*Methylococcaceae*	7
54	*Proteobacteria*	7
55	*Rhodopirellula*	7
56	*Burkholderiales*	6
57	*Comamonadaceae*	6
58	*Desulfobulbaceae*	6
59	*Halomona*	6
60	*Lentisphaeria*	6
61	*Macrococcus*	6
62	*Methylomona*	6
63	*Methylotenera*	6
64	*Nitrospiraceae*	6
65	*Photorhabdus*	6
66	*Priestia*	6
67	*Variovorax*	6
68	*Alcaligenaceae*	5
69	*Carnobacterium*	5
70	*Desulfobacteraceae*	5
71	*Dictyoglomus*	5
72	*Firmicutes*	5
73	*Herbaspirillum*	5
74	*Neisseria*	5
75	*Pelosinus*	5
76	*Pusillimona*	5
77	*Thermotoga*	5
78	*Acidithiobacillales*	4
79	*Alicyclobacillus*	4
80	*Anaeromyxobacter*	4
81	*Companilactobacillus*	4
82	*Hafnia*	4
83	*Janthinobacterium*	4
84	*Listeria*	4
85	*Metakosakonia*	4
86	*Methylocucumis*	4
86	*Oxalobacteraceae*	4
87	*Pseudogulbenkiania*	4

88	*Pseudomonadales*	4
89	*Sulfuricurvum*	4
90	*Thiotrichales*	4
91	*Trabulsiella*	4
92	*uncultured*	4
93	*Xylella*	4
94	*Alcaligenes*	3
95	*Aquifex*	3
96	*Aquitalea*	3
97	*Betaproteobacteria*	3
98	*blood*	3
99	*Caldanaerobacter*	3
100	*Castellaniella*	3
101	*Chromatiales*	3
102	*Desulfovibrio*	3
103	*Ensifer*	3
104	*Gamma*	3
105	*Geoalkalibacter*	3
106	*Hyphomonaceae*	3
107	*Kangiella*	3
108	*Lachnospiraceae*	3
109	*Lacticaseibacillus*	3
110	*Lactobacillus*	3
111	*Leclercia*	3
112	*Luteibacter*	3
113	*Luteimona*	3
114	*Mammaliicoccus*	3
115	*Novosphingobium*	3
116	*Oceanisphaera*	3
117	*Piscirickettsiaceae*	3
118	*Rhizobium*	3
119	*Rhodocyclales*	3
120	*Sedimenticola*	3
121	*Streptomyces*	3
122	*Syntrophotalea*	3
123	*Tatumella*	3
124	*Thermodesulfovibrio*	3
125	*Thioalkalivibrio*	3
126	*Xanthomonadales*	3
127	*Acidiferrobacteraceae*	2
128	*Acidovorax*	2
129	*Actinobacteria*	2
130	*Agrobacterium*	2
131	*Alphaproteobacteria*	2
132	*Azospirillum*	2
133	*Bacteroides*	2
134	*Bibersteinia*	2

135	*Collinsella*	2
136	*Dehalococcoidia*	2
137	*Desulfuromonadales*	2
138	*Elusimicrobia*	2
139	*Gordonibacter*	2
140	*Hahella*	2
141	*Helicobacter*	2
142	*Lentilactobacillus*	2
143	*Mannheimia*	2
144	*Marine*	2
145	*Nitrospira*	2
146	*Nitrospirae*	2
147	*Oceanospirillales*	2
148	*Parabacteroides*	2
149	*Pasteurellaceae*	2
150	*Pediococcus*	2
151	*Photobacterium*	2
152	*Planctomycetaceae*	2
153	*Pluralibacter*	2
154	*Polaromona*	2
155	*Rhodocyclaceae*	2
156	*Romboutsia*	2
157	*Rubrivivax*	2
158	*Sphingobium*	2
159	*Sporolactobacillus*	2
160	*Syntrophobacter*	2
161	*Tetragenococcus*	2
162	*Thauera*	2
163	*Tistrella*	2
164	*Acidithiobacillus*	1
165	*Acidobacteria*	1
166	*Advenella*	1
167	*Aerococcus*	1
168	*Arenimona*	1
169	*Bacteroidetes*	1
170	*Brucella*	1
171	*Campylobacter*	1
172	*candidate*	1
173	*Capnocytophaga*	1
174	*Clostridiaceae*	1
175	*Coxiella*	1
176	*Derxia*	1
177	*Desulfitobacterium*	1
178	*Desulfotalea*	1
179	*Desulfuromona*	1
180	*Eggerthella*	1
181	*Eikenella*	1

182	*Exiguobacterium*	1
183	*Furfurilactobacillus*	1
184	*Gallionella*	1
185	*Gemmataceae*	1
186	*Geobacteraceae*	1
187	*Guyparkeria*	1
188	*Halomonas*	1
189	*Helicobacteraceae*	1
190	*Hydrogenophaga*	1
191	*Koribacter*	1
192	*Kosakonia*	1
193	*Lactococcus*	1
194	*Legionella*	1
195	*Levilactobacillus*	1
196	*Ligilactobacillus*	1
197	*Limosilactobacillus*	1
198	*Magnetospirillum*	1
199	*Marinobacter*	1
200	*Mesorhizobium*	1
201	*Methylomicrobium*	1
202	*Methylophaga*	1
203	*Methylosinus*	1
204	*Mycetohabitans*	1
205	*Mycolicibacterium*	1
206	*Nitrosomonadales*	1
207	*Nitrosospira*	1
208	*Pelagibacterium*	1
209	*Phocaeicola*	1
210	*Proteus*	1
211	*Pseudoalteromona*	1
212	*Rahnella*	1
213	*Rhodanobacter*	1
214	*Rhodopila*	1
215	*Rhodospirillales*	1
216	*Schinkia*	1
217	*Serpentinomona*	1
218	*Serpentinomonas*	1
219	*Shimwellia*	1
220	*Sinorhizobium*	1
221	*Solimona*	1
222	*Spirochaetae*	1
218	*Strain*	1
219	*Syntrophomona*	1
220	*Syntrophus*	1
221	*Thermoanaerobacter*	1
222	*Thermodesulfatator*	1
223	*Tissierellia*	1

224	*Treponema*	1
225	*Verrucomicrobiales*	1
226	*Weissella*	1
227	*Zetaproteobacteria*	1
	**Total**	**2502**
