# Supplementary material for: 'Targeting' the search: An upgraded structural and functional repository of antimicrobial peptides for biofilm studies (B-AMP v2.0) with a focus on biofilm protein targets
Source: Front Cell Infect Microbiol. 2022 Oct 18;12:1020391. doi: 10.3389/fcimb.2022.1020391 (PMC9623296; doi:10.3389/fcimb.2022.1020391)
Supplement: Supplementary Table 3 — Distribution of biofilm targets in B-AMP v2.0 based on bacterial species or description as per NCBI taxonomy. [file Table_3.pdf]

Supplementary Table 3: Distribution of biofilm targets in B-AMP v2.0 based on bacterial species or description as per NCBI taxonomy

Serial Number	Bacterial Species	Count
1	*Escherichia coli*	249
2	*Salmonella enterica*	118
3	*Pseudomonas spp.*	99
4	*Staphylococcus aureus*	81
5	*Acinetobacter baumannii*	72
6	*Klebsiella pneumoniae*	64
7	*Salmonella typhimurium*	58
8	*Pseudomonas fluorescens*	51
9	*Pseudomonas aeruginosa*	45
10	*Stenotrophomonas spp.*	41
11	*Yersinia enterocolitica*	38
12	*Stenotrophomonas maltophilia*	31
13	*Acinetobacter spp.*	28
14	*Deltaproteobacteria bacterium*	27
15	*Bacillus subtilis*	26
16	*Vibrio cholerae*	25
17	*Shewanella oneidensis*	24
18	*Gammaproteobacteria bacterium*	22
19	*Xanthomonas campestris*	20
20	*Citrobacter freundii*	19
21	*Yersinia pestis*	19
22	*Pseudomonas putida*	18
23	*Lentisphaerae bacterium*	17
24	*Porphyromonas gingivalis*	17
25	*Enterobacteriaceae bacterium*	15
26	*Acinetobacter oleivorans*	14
27	*Leptospira interrogans*	13
28	*Ralstonia solanacearum*	12
29	*Serratia marcescens*	12
30	*Bradyrhizobium diazoefficiens*	11
31	*Enterobacter spp.*	11
32	*Porphyromonas gingivalis*	11
33	*Bordetella pertussis*	10
34	*Geobacter sulfurreducens*	10
35	*Staphylococcus epidermidis*	10
36	*Xanthomonadaceae bacterium*	10
37	*Acinetobacter ursingii*	9
38	*Bacillus cereus*	9
39	*Burkholderia multivorans*	9
40	*Comamonae bacterium*	9
41	*Enterobacter hormaechei*	9
42	*Moraxellaceae bacterium*	9

43	*Xanthomonas oryzae*	9
44	*Yersinia pseudotuberculosis*	9
45	*Bordetella bronchiseptica*	8
46	*Bordetella parapertussis*	8
47	*Chloroflexus aurantiacus*	8
48	*Deinococcus radiodurans*	8
49	*Lautropia spp.*	8
50	*Pseudomonas veronii*	8
51	*Synechocystis spp.*	8
52	*Burkholderia pyrrocinia*	7
53	*Enterobacter cloacae*	7
54	*Enterobacter ludwigii*	7
55	*Gallionellales bacterium*	7
56	*Methylococcaceae bacterium*	7
57	*Proteobacteria bacterium*	7
58	*Pseudomonas brassicacearum*	7
59	*Pseudomonas chlororaphis*	7
60	*Pseudomonas lactis*	7
61	*Rhodopirellula baltica*	7
62	*Achromobacter marplatensis*	6
63	*Acinetobacter calcoaceticus*	6
64	*Acinetobacter pittii*	6
65	*Acinetobacter seifertii*	6
66	*Actinobacillus pleuropneumoniae*	6
67	*Aggregatibacter actinomycetemcomitans*	6
68	*Burkholderiales bacterium*	6
69	*Clostridium botulinum*	6
70	*Comamonadaceae bacterium*	6
71	*Desulfobulbaceae bacterium*	6
72	*Escherichia spp.*	6
73	*Halomona spp.*	6
74	*Lentisphaeria bacterium*	6
75	*Lysobacter capsici*	6
76	*Methylotenera versatilis*	6
77	*Nitrospiraceae bacterium*	6
78	*Priestia megaterium*	6
79	*Staphylococcus capitis*	6
80	*Variovorax spp.*	6
81	*Xenorhabdus nematophila*	6
82	*Xenorhabdus spp.*	6
83	*Acinetobacter venetianus*	5
84	*Alcaligenaceae bacterium*	5
85	*Burkholderia spp.*	5
86	*Carnobacterium divergens*	5
87	*Desulfobacteraceae bacterium*	5
88	*Dictyoglomus turgidum*	5
89	*Edwardsiella tarda*	5

90	*Firmicutes bacterium*	5
91	*Neisseria meningitidis*	5
92	*Pelosinus spp.*	5
93	*Pseudomonas proteolytica*	5
94	*Pusillimona spp.*	5
95	*Salmonella enteritidis*	5
96	*Salmonella paratyphi*	5
97	*Serratia plymuthica*	5
98	*Shigella flexneri*	5
99	*Staphylococcus haemolyticus*	5
100	*Streptococcus gordonii*	5
101	*Thermotoga maritima*	5
102	*Acidithiobacillales bacterium*	4
103	*Acinetobacter schindleri*	4
104	*Aeromona salmonicida*	4
105	*Alicyclobacillus acidocaldarius*	4
106	*Anaeromyxobacter spp.*	4
107	*Chromobacterium violaceum*	4
108	*Citrobacter pasteurii*	4
109	*Enterobacter agglomerans*	4
110	*Escherichia fergusonii*	4
111	*Geobacter spp.*	4
112	*Hafnia alvei*	4
113	*Janthinobacterium spp.*	4
114	*Klebsiella aerogenes*	4
115	*Listeria monocytogenes*	4
116	*Metakosakonia spp.*	4
117	*Methylocucumis oryzae*	4
118	*Oxalobacteraceae bacterium*	4
119	*Pectobacterium atrosepticum*	4
120	*Pectobacterium parmentieri*	4
121	*Pectobacterium peruviense*	4
122	*Pectobacterium polonicum*	4
123	*Pseudogulbenkiania spp.*	4
124	*Pseudomonadales bacterium*	4
125	*Pseudomonas baetica*	4
126	*Pseudomonas fuscovaginae*	4
127	*Pseudomonas gessardii*	4
128	*Pseudomonas kairouanensis*	4
129	*Pseudomonas moraviensis*	4
130	*Pseudomonas ogarae*	4
131	*Pseudomonas panacis*	4
132	*Pseudomonas protegens*	4
133	*Pseudomonas silesiensis*	4
134	*Pseudomonas synxantha*	4
135	*Pseudomonas trivialis*	4
136	*Pseudomonas viciae*	4

137	*Shigella spp.*	4
138	*Staphylococcus caprae*	4
139	*Staphylococcus lugdunensis*	4
140	*Stenotrophomonas rhizophila*	4
141	*Streptococcus pneumoniae*	4
142	*Thiotrichales bacterium*	4
143	*Trabulsiella odontotermitis*	4
144	*Xylella fastidiosa*	4
145	*Achromobacter arsenitoxydans*	3
146	*Achromobacter spp.*	3
147	*Achromobacter xylosoxidans*	3
148	*Acinetobacter baylyi*	3
149	*Acinetobacter cumulans*	3
150	*Acinetobacter wuhouensis*	3
151	*Aeromona hydrophila*	3
152	*Alcaligenes spp.*	3
153	*Aquifex aeolicus*	3
154	*Aquitalea magnusonii*	3
155	*Betaproteobacteria bacterium*	3
156	*blood disease*	3
157	*Caldanaerobacter subterraneus*	3
158	*Candidatus Competibacteraceae*	3
159	*Candidatus Desulfofervidus*	3
160	*Candidatus Manganitrophus*	3
161	*Candidatus Muproteobacteria*	3
162	*Candidatus thiomargarita*	3
163	*Castellaniella defragrans*	3
164	*Chromatiales bacterium*	3
165	*Chromobacterium haemolyticum*	3
166	*Chromobacterium spp.*	3
167	*Clostridium spp.*	3
168	*Cronobacter dublinensis*	3
169	*Cronobacter sakazakii*	3
170	*Cupriavidus gilardii*	3
171	*Ensifer adhaerens*	3
172	*Enterobacter asburiae*	3
173	*Enterobacter chengduensis*	3
174	*Enterobacter kobei*	3
175	*Gamma proteobacterium*	3
176	*Geoalkalibacter subterraneus*	3
177	*Herbaspirillum spp.*	3
178	*Hyphomonaceae bacterium*	3
179	*Kangiella koreensis*	3
180	*Lachnospiraceae bacterium*	3
181	*Leclercia spp.*	3
182	*Luteibacter yeojuensis*	3
183	*Luteimona cucumeris*	3

184	*Lysobacter antibioticus*	3
185	*Lysobacter enzymogenes*	3
186	*Lysobacter gummosus*	3
187	*Methylomona denitrificans*	3
188	*Methylomona methanica*	3
189	*Oceanisphaera profunda*	3
190	*Paraburkholderia phytofirmans*	3
191	*Photorhabdus spp.*	3
192	*Piscirickettsiaceae bacterium*	3
193	*Pseudomonas aestus*	3
194	*Pseudomonas nabeulensis*	3
195	*Pseudomonas orientalis*	3
196	*Pseudomonas prosekii*	3
197	*Pseudomonas yamanorum*	3
198	*Ralstonia pickettii*	3
199	*Ralstonia solanaarum*	3
200	*Ralstonia syzygii*	3
201	*Rhodocyclales bacterium*	3
202	*Salmonella newport*	3
203	*Sedimenticola spp.*	3
204	*Serratia spp.*	3
205	*Staphylococcus saprophyticus*	3
206	*Stenotrophomonas geniculata*	3
207	*Streptomyces coelicolor*	3
208	*Sulfuricurvum kujiense*	3
209	*Sulfurimona denitrificans*	3
210	*Sulfurimona gotlandica*	3
211	*Sulfurimona spp.*	3
212	*Syntrophotalea carbinolica*	3
213	*Tatumella spp.*	3
214	*Thermodesulfovibrio yellowstonii*	3
215	*Thioalkalivibrio sulfidiphilus*	3
216	*Xanthomonadales bacterium*	3
217	*Xanthomonas axonopodis*	3
218	*Xanthomonas citri*	3
219	*Xanthomonas gardneri*	3
220	*Xanthomonas hortorum*	3
221	*Xanthomonas vasicola*	3
222	*Xanthomonas vesicatoria*	3
223	*Xenorhabdus budapestensis*	3
224	*Xenorhabdus ehlersii*	3
225	*Xenorhabdus ishibashii*	3
226	*Xenorhabdus kozodoii*	3
227	*Xenorhabdus stockiae*	3
228	*Yersinia entomophaga*	3
229	*Acidiferrobacteraceae bacterium*	2
230	*Acidovorax citrulli*	2

231	*Actinobacillus succinogenes*	2
232	*Actinobacillus suis*	2
233	*Actinobacteria bacterium*	2
234	*Aeromona sobria*	2
235	*Aeromona veronii*	2
236	*Aggregatibacter aphrophilus*	2
237	*Alphaproteobacteria bacterium*	2
238	*Azospirillum lipoferum*	2
239	*Bacillus licheniformis*	2
240	*Bibersteinia trehalosi*	2
241	*Burkholderia ambifaria*	2
242	*Burkholderia cenocepacia*	2
243	*Candidatus Accumulibacter*	2
244	*Candidatus Rokubacteria*	2
245	*Collinsella aerofaciens*	2
246	*Companilactobacillus farciminis*	2
247	*Cronobacter malonaticus*	2
248	*Cupriavidus basilensis*	2
249	*Cupriavidus necator*	2
250	*Dehalococcoidia bacterium*	2
251	*Desulfovibrio spp.*	2
252	*Desulfuromonadales bacterium*	2
253	*Elusimicrobia bacterium*	2
254	*Hahella chejuensis*	2
255	*Helicobacter pylori*	2
256	*Klebsiella oxytoca*	2
257	*Lacticaseibacillus fabifermentans*	2
258	*Lysobacter arseniciresistens*	2
259	*Macrococcus caseolyticus*	2
260	*Macrococcus goetzii*	2
261	*Mannheimia succiniciproducens*	2
262	*Marine gamma*	2
263	*Nitrospira bacterium*	2
264	*Nitrospirae bacterium*	2
265	*Novosphingobium nitrogenifigens*	2
266	*Oceanospirillales bacterium*	2
267	*Parabacteroides distasonis*	2
268	*Paraburkholderia azotifigens*	2
269	*Paraburkholderia caribensis*	2
270	*Paraburkholderia hospita*	2
271	*Paraburkholderia spp.*	2
272	*Pasteurellaceae bacterium*	2
273	*Pectobacterium brasiliense*	2
274	*Pediococcus damnosus*	2
275	*Photobacterium profundum*	2
276	*Planctomycetaceae bacterium*	2
277	*Pluralibacter gergoviae*	2

278	*Pseudomonas furukawaii*	2
279	*Rhodocyclaceae bacterium*	2
280	*Salmonella dublin*	2
281	*Salmonella gallinarum*	2
282	*Salmonella typhi*	2
283	*Sporolactobacillus laevolacticus*	2
284	*Staphylococcus cohnii*	2
285	*Staphylococcus condimenti*	2
286	*Staphylococcus delphini*	2
287	*Streptococcus mutans*	2
288	*Syntrophobacter spp.*	2
289	*Thauera spp.*	2
290	*Tistrella mobilis*	2
291	*uncultured bacterium*	2
292	*uncultured Desulfobacterium*	2
293	*Vibrio scophthalmi*	2
294	*Xanthomonas cucurbitae*	2
295	*Acidithiobacillus ferrivorans*	1
296	*Acidobacteria bacterium*	1
297	*Acinetobacter nosocomialis*	1
298	*Advenella kashmirensis*	1
299	*Aerococcus urinaehominis*	1
300	*Agrobacterium rubi*	1
301	*Agrobacterium salinitolerans*	1
302	*Arenimona spp.*	1
303	*Bacillus atrophaeus*	1
304	*Bacillus glycinifermentans*	1
305	*Bacillus megaterium*	1
306	*Bacillus methanolicus*	1
307	*Bacillus pumilus*	1
308	*Bacillus sonorensis*	1
309	*Bacillus spizizenii*	1
310	*Bacillus spp.*	1
311	*Bacillus stratosphericus*	1
312	*Bacteroides ovatus*	1
313	*Bacteroides uniformis*	1
314	*Bacteroidetes bacterium*	1
315	*Brucella melitensis*	1
316	*Campylobacter jejuni*	1
317	*candidate division*	1
318	*Candidatus Eisenbacteria*	1
319	*Candidatus Fermentibacter*	1
320	*Candidatus Lambdaproteobacteria*	1
321	*Candidatus melainabacteria*	1
322	*Capnocytophaga ochracea*	1
323	*Citrobacter amalonaticus*	1
324	*Citrobacter rodentium*	1

325	*Citrobacter spp.*	1
326	*Clostridiaceae bacterium*	1
327	*Companilactobacillus mindensis*	1
328	*Companilactobacillus salsicarnum*	1
329	*Coxiella burnetii*	1
330	*Cronobacter condimenti*	1
331	*Cronobacter turicensis*	1
332	*Cupriavidus oxalaticus*	1
333	*Cupriavidus spp.*	1
334	*Cupriavidus taiwanensis*	1
335	*Derxia gummosa*	1
336	*Desulfitobacterium hafniense*	1
337	*Desulfotalea psychrophila*	1
338	*Desulfovibrio magneticus*	1
339	*Desulfuromona spp.*	1
340	*Edwardsiella anguillarum*	1
341	*Edwardsiella ictaluri*	1
342	*Edwardsiella piscicida*	1
343	*Eggerthella spp.*	1
344	*Eikenella corrodens*	1
345	*Enterobacter lignolyticus*	1
346	*Exiguobacterium chiriqhucha*	1
347	*Furfurilactobacillus rossiae*	1
348	*Gallionella spp.*	1
349	*Gemmataceae bacterium*	1
350	*Geobacteraceae bacterium*	1
351	*Gordonibacter pamelaeae*	1
352	*Gordonibacter urolithinfaciens*	1
353	*Guyparkeria spp.*	1
354	*Halomonas spp.*	1
355	*Helicobacteraceae bacterium*	1
356	*Herbaspirillum aquaticum*	1
357	*Herbaspirillum frisingense*	1
358	*Hydrogenophaga spp.*	1
359	*Koribacter versatilis*	1
360	*Kosakonia radicincitans*	1
361	*Lacticaseibacillus rhamnosus*	1
362	*Lactobacillus acidophilus*	1
363	*Lactobacillus johnsonii*	1
364	*Lactobacillus spp.*	1
365	*Lactococcus lactis*	1
366	*Legionella drancourtii*	1
367	*Lentilactobacillus diolivorans*	1
368	*Lentilactobacillus kisonensis*	1
369	*Leptospira licerasiae*	1
370	*Levilactobacillus koreensis*	1
371	*Ligilactobacillus apodemi*	1

372	*Limosilactobacillus mucosae*	1
373	*Lysobacter ruishenii*	1
374	*Macrococcus bohemicus*	1
375	*Macrococcus epidermidis*	1
376	*Magnetospirillum gryphiswaldense*	1
377	*Mammaliicoccus fleurettii*	1
378	*Mammaliicoccus lentus*	1
379	*Mammaliicoccus sciuri*	1
380	*Marinobacter nauticus*	1
381	*Mesorhizobium huakuii*	1
382	*Methylomicrobium album*	1
383	*Methylophaga spp.*	1
384	*Methylosinus sporium*	1
385	*Mycetohabitans rhizoxinica*	1
386	*Mycolicibacterium smegmatis*	1
387	*Nitrosomonadales bacterium*	1
388	*Nitrosospira multiformis*	1
389	*Novosphingobium barchaimii*	1
390	*Paraburkholderia madseniana*	1
391	*Pelagibacterium spp.*	1
392	*Phocaeicola vulgatus*	1
393	*Photorhabdus khanii*	1
394	*Photorhabdus temperata*	1
395	*Photorhabdus thracensis*	1
396	*Polaromona naphthalenivorans*	1
397	*Polaromona spp.*	1
398	*Proteus mirabilis*	1
399	*Pseudoalteromona translucida*	1
400	*Pseudomonas caspiana*	1
401	*Pseudomonas chengduensis*	1
402	*Pseudomonas mandelii*	1
403	*Rahnella spp.*	1
404	*Rhizobium freirei*	1
405	*Rhizobium radiobacter*	1
406	*Rhizobium spp.*	1
407	*Rhodanobacter spp.*	1
408	*Rhodopila globiformis*	1
409	*Rhodospirillales bacterium*	1
410	*Romboutsia maritimum*	1
411	*Romboutsia weinsteinii*	1
412	*Rubrivivax benzoatilyticus*	1
413	*Rubrivivax gelatinosus*	1
414	*Salmonella abortus-equi*	1
415	*Salmonella agona*	1
416	*Salmonella anatum*	1
417	*Salmonella bongori*	1
418	*Salmonella choleraesuis*	1

419	*Salmonella derby*	1
420	*Salmonella diarizonae*	1
421	*Salmonella hadar*	1
422	*Salmonella heidelberg*	1
423	*Salmonella houtenae*	1
424	*Salmonella infantis*	1
425	*Salmonella montevideo*	1
426	*Salmonella moscow*	1
427	*Salmonella muenchen*	1
428	*Salmonella oranienberg*	1
429	*Salmonella potsdam*	1
430	*Salmonella schwarzengrund*	1
431	*Salmonella senftenberg*	1
432	*Salmonella thompson*	1
433	*Salmonella typhisuis*	1
434	*Salmonella virchow*	1
435	*Schinkia azotoformans*	1
436	*Serpentinomona mccroryi*	1
437	*Serpentinomonas raichei*	1
438	*Serratia fonticola*	1
439	*Serratia inhibens*	1
440	*Shewanella spp.*	1
441	*Shigella dysenteriae*	1
442	*Shigella sonnei*	1
443	*Shimwellia blattae*	1
444	*Sinorhizobium terangae*	1
445	*Solimona terrae*	1
446	*Sphingobium baderi*	1
447	*Sphingobium spp.*	1
448	*Spirochaetae bacterium*	1
449	*Staphylococcus pseudintermedius*	1
450	*Staphylococcus simiae*	1
451	*Staphylococcus simulans*	1
452	*Staphylococcus spp.*	1
453	*Strain Marseille*	1
454	*Streptococcus anginosus*	1
455	*Streptococcus parasanguinis*	1
456	*Streptococcus pyogenes*	1
457	*Streptococcus salivarius*	1
458	*Sulfuricurvum spp.*	1
459	*Syntrophomona wolfei*	1
460	*Syntrophus aciditrophicus*	1
461	*Tetragenococcus halophilus*	1
462	*Tetragenococcus koreensis*	1
463	*Thermoanaerobacter spp.*	1
464	*Thermodesulfatator atlanticus*	1
465	*Tissierellia bacterium*	1

466	*Treponema denticola*	1
467	*Verrucomicrobiales bacterium*	1
468	*Vibrio alginolyticus*	1
469	*Vibrio parahaemolyticus*	1
470	*Vibrio spp.*	1
471	*Vibrio vulnificus*	1
472	*Weissella cibaria*	1
473	*Zetaproteobacteria bacterium*	1
	**Total**	**2502**
