# Supplementary material for: 'Targeting' the search: An upgraded structural and functional repository of antimicrobial peptides for biofilm studies (B-AMP v2.0) with a focus on biofilm protein targets
Source: Front Cell Infect Microbiol. 2022 Oct 18;12:1020391. doi: 10.3389/fcimb.2022.1020391 (PMC9623296; doi:10.3389/fcimb.2022.1020391)
Supplement: Supplementary Table 4 — List of PDB target functions of biofilm targets in B-AMP v2.0 [file Table_4.pdf]

Supplementary Table 4: PDB functions of the biofilm targets in B-AMP v2.0

Serial Number	Classification as per PDB	Number of biofilm targets
1.	Cell adhesion	23
2.	Transferase	10
3.	Hydrolase / Hydrolase inhibitor	9
4.	Signaling protein / inhibitor	9
5.	Structural protein	8
6.	Transcription	6
7.	Immune system / RNA	4
8.	Protein fibril	3
9.	Sugar binding protein/ inhibitor	3
10.	Gene regulation	2
11.	Protein binding	2
12.	Membrane protein	2
13.	RNA binding protein / chaperone	2
14.	DNA binding protein / toxin	2
15.	Transcriptional regulator	2
16.	Transport protein	1
17.	Fimbrial protein	1
18.	Protein transport	1
19.	Metal binding protein	1
20.	Signaling protein activator	1
21.	Peptide binding protein	1
22.	Toxin inhibitor	1
23.	Calcium binding protein	1
24.	Ribosome	1
25.	Chaperone / protein transport	1
26.	Structural genomics / biosynthetic protein	1
