# Supplementary material for: 'Targeting' the search: An upgraded structural and functional repository of antimicrobial peptides for biofilm studies (B-AMP v2.0) with a focus on biofilm protein targets
Source: Front Cell Infect Microbiol. 2022 Oct 18;12:1020391. doi: 10.3389/fcimb.2022.1020391 (PMC9623296; doi:10.3389/fcimb.2022.1020391)
Supplement: Supplementary Table 5 — List of UniProt target functions of biofilm targets in B-AMP v2.0 [file Table_5.pdf]

Supplementary Table 5: List of UniProt functions of biofilm targets in B-AMP v2.0

Serial Number	Classification as per UniProt	Number of biofilm targets
1.	Acetyl glucosaminyl transferase activity	474
2.	Hydrolase activity	442
3.	Acting on carbon-nitrogen (but not peptide) bonds	415
4.	Diguanylate cyclase activity	188
5.	DNA binding	69
6.	ATP binding	34
7.	Phosphorelay sensor kinase activity	33
8.	Metal ion binding	22
9.	DNA-directed DNA polymerase activity	18
10.	GTP binding	15
11.	Identical protein binding	14
12.	Transcription cis-regulatory region binding	10
13.	Acting on glycosyl bonds	10
14.	Structural proteins	9
15.	DNA-binding Transcription activator activity	9
16.	N-acyl homoserine lactone synthase activity	9
17.	S-ribosylhomocysteine lyase activity	9
18.	Iron ion binding	9
19.	RNA binding	8
20.	Protein dimerization activity	7
21.	AcylTransferase activity	7
22.	Endoribonuclease activity	6
23.	Phosphorelay response regulator activity	6
24.	Zinc ion binding	6
25.	Host cell extracellular matrix binding	6
26.	Toxin activity	6
27.	Sigma factor activity	5
28.	Transmembrane transporter activity	5
29.	Glycosyl transferase activity	5
30.	DNA-binding Transcription repressor activity	4
31.	DNA-binding Transcription factor activity	4
32.	Catalytic activity	4
33.	Sequence-specific DNA binding	4
34.	Transferring groups other than amino-acyl groups	4

35.	Phosphopantetheine binding	3
36.	Kinase activity	3
37.	Calcium ion binding	3
38.	tRNA binding	3
39.	Oxidoreductase activity	5
40.	Phosphoprotein phosphatase activity	3
41.	DNA-directed 5'-3' RNA polymerase activity	3
42.	Protein histidine kinase activity	2
43.	Transferase activity	2
44.	Nucleotide binding	2
45.	PhosphoTransferase activity	2
46.	DNA binding function	2
47.	3-oxoacyl-[acyl-carrier-protein] synthase activity	2
48.	Efflux transmembrane transporter activity	2
49.	Exodeoxyribonuclease III activity	2
50.	Protein-N(PI)-phosphohistidine-sugar phosphotransferase activity	2
51.	Cyclic-di-GMP binding	2
52.	Phosphoric diester hydrolase activity	2
53.	Transcription antitermination factor activity	2
54.	Helicase activity	2
55.	Endonuclease activity	2
56.	Mannose binding	2
57.	Hydrolyzing O-glycosyl compounds	2
58.	5'-3' exoribonuclease activity	2
59.	mRNA 5'-UTR binding	2
60.	Ribosome binding	2
61.	Acting on the aldehyde or oxo group of donors	2
62.	HexosylTransferase activity	1
63.	GTPase activity	1
64.	RNA strand annealing activity	1
65.	Oxygen binding	1
66.	Ribosomal large subunit binding	1
67.	RNA helicase activity	1
68.	Isomerase activity	1
69.	Pheromone activity	1
70.	Enzyme binding	1
71.	Serine-type peptidase activity	1
72.	Protein Kinase activator activity	1

73.	ABC-type bacteriocin transporter activity	1
74.	Deacetylase activity	1
75.	Pyrophosphatase activity	1
76.	Polyphosphate kinase activity	1
77.	5'-bis(diphosphate) 3'-diphosphatase activity	1
78.	Toxic substance binding	1
79.	Ligase activity	1
80.	Producing 3'-phosphomonoesters	1
81.	For other substituted phosphate groups	1
82.	Double-stranded DNA 3'-5' exodeoxyribonuclease activity	1
83.	DNA strand exchange activity	1
84.	Protein homodimerization activity	1
85.	Transaminase activity	1
86.	Cellulose synthase (UDP-forming) activity	1
87.	Serine-type endopeptidase activity	1
88.	Cholesterol binding	1
89.	Cis-regulatory region Sequence-specific DNA binding	1
90.	Ribosomal small subunit binding	1
91.	tRNA nucleotidylTransferase activity	1
92.	3-hydroxyisobutyryl-CoA Hydrolase activity	1
93.	Endoribonuclease inhibitor activity	1
94.	ATP hydrolysis activity	1
95.	Fimbrial usher porin activity	1
96.	Phosphate ion binding	1
97.	Transmembrane signaling receptor activity	1
98.	dCMP deaminase activity	1
99.	Carbohydrate binding	1
100.	Exopolyphosphatase activity	1
101.	Bacterial-type RNA polymerase core enzyme binding	1
102.	Cysteine-type peptidase activity	1
103.	Nickel cation binding	1
104.	DNA-(apurinic or apyrimidinic site) endonuclease activity	1
105.	Flavin adenine dinucleotide binding	1
106.	Alpha-L-fucosidase activity	1
107.	Porin activity	1
108.	Adenosylmethionine decarboxylase activity	1
109.	Guanosine-3'	1
110.	Beta-ketoacyl-acyl-carrier-protein synthase III activity	1

111.	O-acetyl-ADP-ribose deacetylase activity	1
112.	Acting on the CH-CH group of donors	1
113.	Alcohol group as acceptor	1
114.	Adenosylhomocysteinase activity	1
115.	Complement component C3b binding	1
116.	Cellulase activity	1
117.	Pyridoxal phosphate binding	1
118.	ADP-dependent short-chain-acyl-CoA hydrolase activity	1
119.	Purine nucleoside binding	1
120.	Translation activator activity	1
121.	Amino acid binding	1
122.	NucleotidylTransferase activity	1
123.	rRNA binding	1
124.	Carbon monoxide binding	1
125.	MethylTransferase activity	1
126.	Protein-containing complex binding	1
127.	Heme binding	1
128.	Autoinducer-2 kinase activity	1
129.	Beta-lactamase activity	1
130.	Protein-exporting ATPase activity	1
131.	3'-5'-exoribonuclease activity	1
132.	Protein kinase activity	1
