# Supplementary material for: 'Targeting' the search: An upgraded structural and functional repository of antimicrobial peptides for biofilm studies (B-AMP v2.0) with a focus on biofilm protein targets
Source: Front Cell Infect Microbiol. 2022 Oct 18;12:1020391. doi: 10.3389/fcimb.2022.1020391 (PMC9623296; doi:10.3389/fcimb.2022.1020391)
Supplement: Supplementary Table 7 — List of bacterial species with PDB structures in B-AMP v2.0. [file Table_7.pdf]

Supplementary File 7: List of bacterial species with targets with PDB structures in B-AMP v2.0

Serial Number	Bacterial species with PDB structures available	Number of PDB structures
1.	*Acinetobacter spp.*	1
2.	*Aggregatibacter actinomycetemcomitans*	1
3.	*Bacillus subtilis*	9
4.	*Bacteroides ovatus*	1
5.	*Bacteroides uniformis*	1
6.	*Bordetella bronchiseptica*	1
7.	*Escherichia coli*	54
8.	*Geobacter sulfurreducens*	1
9.	*Marinobacter nauticus*	1
10.	*Neisseria meningitidis* serogroup B	1
11.	*Parabacteroides distasonis*	2
12.	*Phocaeicola vulgatus*	1
13.	*Porphyromonas gingivalis*	12
14.	*Proteus mirabilis*	1
15.	*Pseudomonas aeruginosa*	67
16.	*Salmonella typhimurium*	12
17.	*Shewanella oneidensis*	1
18.	*Staphylococcus aureus*	13
19.	*Staphylococcus epidermidis*	1
20.	*Streptococcus gordonii*	9
21.	*Streptococcus mutans*	2
22.	*Streptococcus parasanguinis*	1
23.	*Streptococcus pneumoniae* serotype 4	1
24.	*Streptococcus pyogenes* serotype M1	3
25.	*Xanthomonas campestris* pv. *campestris*	1
