# Supplementary material for: 'Targeting' the search: An upgraded structural and functional repository of antimicrobial peptides for biofilm studies (B-AMP v2.0) with a focus on biofilm protein targets
Source: Front Cell Infect Microbiol. 2022 Oct 18;12:1020391. doi: 10.3389/fcimb.2022.1020391 (PMC9623296; doi:10.3389/fcimb.2022.1020391)
Supplement: Supplementary Table 8 — List of P. aeruginosa biofilm targets modeled using RoseTTAFold in B-AMP v2.0. [file Table_8.pdf]

Supplementary Table 8: *Pseudomonas aeruginosa* biofilm protein targets modeled using RoseTTAfold

Serial Number	UniProt Entry	Target IDs	Protein names	Length of protein
1.	Q9HU67	533	50S ribosomal subunit assembly factor BipA, EC 3.6.5.- (GTP-binding protein BipA)	605
2.	K7QUT8	843	Adenosylhomocysteinase, EC 3.3.1.1 (S-adenosyl-L-homocysteine hydrolase, AdoHcyase)	469
3.	Q9HT84	2248	Diguanylate cyclase DgcP, EC 2.7.7.65	671
4.	G3XDB0	528	Exodeoxyribonuclease III, EC 3.1.11.2	259
5.	Q9ZN70	106	Exopolyphosphatase, ExopolyPase, EC 3.6.1.11 (Polyphosphate:ADP phosphotransferase, PolyP:ADP phosphotransferase, EC 2.7.4.1)	506
6.	Q9HTS0	529	Fimbrial domain-containing protein	304
7.	Q9I1Y7	520	Fimbrial subunit CupA1	183
8.	Q9I4X7	531	Fimbrial subunit CupC1	205
9.	Q9HZX6	709	GGDEF domain-containing protein	525
10.	Q9I2P4	699	GGDEF domain-containing protein	401
11.	Q9I4M8	527	GGDEF domain-containing protein	398
12.	Q9HYQ2	526	GGDEF domain-containing protein	389
13.	Q9HZ57	532	GGDEF domain-containing protein	307
14.	Q9I6W3	525	GGDEF domain-containing protein	235
15.	Q9HWU2	530	Probable fimbrial subunit CupB1	189
16.	Q9HUW7	711	Probable two-component response regulator	542
17.	G3XD78	535	Regulatory protein RsaL	80
18.	Q51373	2268	Response regulator GacA (Global activator)	214

19.	Q9I4N3	2272	Response regulator protein FleR	473
20.	A0A0H2ZG R9	2279	RNA polymerase sigma-54 factor	497
21.	P49988	2212	RNA polymerase sigma-54 factor	497
22.	Q9HWA4	107	Two-component response regulator PprB	275
23.	Q9I6K0	534	Uncharacterized protein	323
