# Supplementary material for: 'Targeting' the search: An upgraded structural and functional repository of antimicrobial peptides for biofilm studies (B-AMP v2.0) with a focus on biofilm protein targets
Source: Front Cell Infect Microbiol. 2022 Oct 18;12:1020391. doi: 10.3389/fcimb.2022.1020391 (PMC9623296; doi:10.3389/fcimb.2022.1020391)
Supplement: Supplementary Table 9 — List of S. aureus biofilm targets modeled using RoseTTAFold in B-AMP v2.0. [file Table_9.pdf]

Supplementary Table 9: *Staphylococcus aureus* biofilm protein targets modeled using RoseTTAfold

Serial Number	UniProt Entry	Target IDs	Protein names	Length of protein
1.	Q9RQQ0	2362	Biofilm operon icaADBC HTH-type negative transcriptional regulator IcaR (Intercellular adhesion protein R)	186
2.	Q2FWH5	2261	DEAD-box ATP-dependent RNA helicase CshA, EC 3.6.4.13	506
3.	A0A0H2XKG3	2259	Fibronectin-binding protein B	940
4.	Q2G1U6	2285	Global transcriptional regulator Spx	131
5.	Q9RQP5	404	Glycerol ester hydrolase	108
6.	Q83ZH9	402	IcaA	124
7.	Q9RQP7	2355	Poly-beta-1,6-N-acetyl-D-glucosamine N-deacetylase, PNAG N-deacetylase, Poly-beta-1,6-GlcNAc N-deacetylase, EC 3.5.1.- (Biofilm polysaccharide intercellular adhesin deacetylase, Biofilm PIA deacetylase) (Intercellular adhesion protein B)	290
8.	Q5HCN1	2347	Poly-beta-1,6-N-acetyl-D-glucosamine synthase, PNAG synthase, Poly-beta-1,6-GlcNAc synthase, EC 2.4.1.- (Biofilm polysaccharide intercellular adhesin synthesis protein IcaA, Biofilm PIA synthesis protein IcaA) (Intercellular adhesion protein A) (N-acetylglucosaminyltransferase IcaA)	412
9.	Q6G608	2351	Poly-beta-1,6-N-acetyl-D-glucosamine synthase, PNAG synthase, Poly-beta-1,6-GlcNAc synthase, EC 2.4.1.- (Biofilm polysaccharide intercellular adhesin synthesis protein IcaA, Biofilm PIA synthesis protein IcaA) (Intercellular adhesion protein A) (N-acetylglucosaminyltransferase IcaA)	412

10.	Q6GDD8	2350	Poly-beta-1,6-N-acetyl-D-glucosamine synthase, PNAG synthase, Poly-beta-1,6-GlcNAc synthase, EC 2.4.1.- (Biofilm polysaccharide intercellular adhesin synthesis protein IcaA, Biofilm PIA synthesis protein IcaA) (Intercellular adhesion protein A) (N-acetylglucosaminyltransferase IcaA)	412
11.	Q7A351	2349	Poly-beta-1,6-N-acetyl-D-glucosamine synthase, PNAG synthase, Poly-beta-1,6-GlcNAc synthase, EC 2.4.1.- (Biofilm polysaccharide intercellular adhesin synthesis protein IcaA, Biofilm PIA synthesis protein IcaA) (Intercellular adhesion protein A) (N-acetylglucosaminyltransferase IcaA)	412
12.	Q8NUI7	2352	Poly-beta-1,6-N-acetyl-D-glucosamine synthase, PNAG synthase, Poly-beta-1,6-GlcNAc synthase, EC 2.4.1.- (Biofilm polysaccharide intercellular adhesin synthesis protein IcaA, Biofilm PIA synthesis protein IcaA) (Intercellular adhesion protein A) (N-acetylglucosaminyltransferase IcaA)	412
13.	Q99QX3	2348	Poly-beta-1,6-N-acetyl-D-glucosamine synthase, PNAG synthase, Poly-beta-1,6-GlcNAc synthase, EC 2.4.1.- (Biofilm polysaccharide intercellular adhesin synthesis protein IcaA, Biofilm PIA synthesis protein IcaA) (Intercellular adhesion protein A) (N-acetylglucosaminyltransferase IcaA)	412
14.	Q9RQP9	2346	Poly-beta-1,6-N-acetyl-D-glucosamine synthase, PNAG synthase, Poly-beta-1,6-GlcNAc synthase, EC 2.4.1.- (Biofilm polysaccharide intercellular adhesin synthesis protein IcaA, Biofilm PIA synthesis protein IcaA) (Intercellular adhesion protein A) (N-acetylglucosaminyltransferase IcaA)	412
15.	A0A0H2XJC1	1320	Poly-beta-1,6-N-acetyl-D-glucosamine synthase, Poly-beta-1,6-GlcNAc synthase, EC 2.4.1.-	412

16.	A0A0H3KAW6	462	Poly-beta-1,6-N-acetyl-D-glucosamine synthase, Poly-beta-1,6-GlcNAc synthase, EC 2.4.1.-	412
17.	A0A1D0C161	396	Poly-beta-1,6-N-acetyl-D-glucosamine synthase, Poly-beta-1,6-GlcNAc synthase, EC 2.4.1.-	410
18.	A0A2S6DQT8	823	Poly-beta-1,6-N-acetyl-D-glucosamine synthase, Poly-beta-1,6-GlcNAc synthase, EC 2.4.1.-	412
19.	A0A6A9GS78	403	Poly-beta-1,6-N-acetyl-D-glucosamine synthase, Poly-beta-1,6-GlcNAc synthase, EC 2.4.1.-	412
20.	W8TQA2	1786	Poly-beta-1,6-N-acetyl-D-glucosamine synthase, Poly-beta-1,6-GlcNAc synthase, EC 2.4.1.-	412
21.	Q9RQP8	2361	Poly-beta-1,6-N-acetyl-D-glucosamine synthesis protein IcaD, PGA synthesis protein IcaD, Poly-beta-1,6-GlcNAc synthesis protein IcaD (Biofilm polysaccharide intercellular adhesin synthesis protein IcaD, Biofilm PIA synthesis protein IcaD) (Intercellular adhesion protein D)	101
22.	Q9RQP6	2358	Probable poly-beta-1,6-N-acetyl-D-glucosamine export protein, PGA export protein, Poly-beta-1,6-GlcNAc export protein (Biofilm polysaccharide intercellular adhesin export protein, Biofilm PIA export protein) (Intercellular adhesion protein C)	350
23.	P60612	2228	Sensor histidine kinase/phosphatase LytS, EC 2.7.13.3, EC 3.1.3.- (Autolysin sensor kinase)	584
24.	P60613	2229	Sensor histidine kinase/phosphatase LytS, EC 2.7.13.3, EC 3.1.3.- (Autolysin sensor kinase)	584
25.	P60614	2232	Sensor histidine kinase/phosphatase LytS, EC 2.7.13.3, EC 3.1.3.- (Autolysin sensor kinase)	584

26.	Q2FK10	2224	Sensor histidine kinase/phosphatase LytS, EC 2.7.13.3, EC 3.1.3.- (Autolysin sensor kinase)	584
27.	Q2YV68	2226	Sensor histidine kinase/phosphatase LytS, EC 2.7.13.3, EC 3.1.3.- (Autolysin sensor kinase)	584
28.	Q53705	2225	Sensor histidine kinase/phosphatase LytS, EC 2.7.13.3, EC 3.1.3.- (Autolysin sensor kinase)	584
29.	Q5HJB6	2227	Sensor histidine kinase/phosphatase LytS, EC 2.7.13.3, EC 3.1.3.- (Autolysin sensor kinase)	584
30.	Q6GCL2	2231	Sensor histidine kinase/phosphatase LytS, EC 2.7.13.3, EC 3.1.3.- (Autolysin sensor kinase)	584
31.	Q6GK52	2230	Sensor histidine kinase/phosphatase LytS, EC 2.7.13.3, EC 3.1.3.- (Autolysin sensor kinase)	584
32.	Q5HFT1	2215	Sensor protein SrrB, EC 2.7.13.3 (Staphylococcal respiratory response protein B)	583
33.	Q6G973	2301	Sensor protein SrrB, EC 2.7.13.3 (Staphylococcal respiratory response protein B)	583
34.	Q6GGK7	2296	Sensor protein SrrB, EC 2.7.13.3 (Staphylococcal respiratory response protein B)	583
35.	Q7A5H7	2295	Sensor protein SrrB, EC 2.7.13.3 (Staphylococcal respiratory response protein B)	583
36.	Q8NWF3	2294	Sensor protein SrrB, EC 2.7.13.3 (Staphylococcal respiratory response protein B)	583
37.	Q99TZ9	2197	Sensor protein SrrB, EC 2.7.13.3 (Staphylococcal respiratory response protein B)	583
38.	Q5HEN9	2319	Sensor protein VraS, EC 2.7.13.3	347

39.	Q6G849	2323	Sensor protein VraS, EC 2.7.13.3	347
40.	Q6GFH2	2299	Sensor protein VraS, EC 2.7.13.3	347
41.	Q7A0H9	2310	Sensor protein VraS, EC 2.7.13.3	347
42.	Q99SZ7	2333	Sensor protein VraS, EC 2.7.13.3	347
43.	Q9KWK8	2320	Sensor protein VraS, EC 2.7.13.3	347
44.	O86487	2284	Serine-aspartate repeat-containing protein C	947
45.	Q2FFR1	2202	Signal transduction protein TRAP (Target of RNAIII-activating protein)	167
46.	Q2YU04	2203	Signal transduction protein TRAP (Target of RNAIII-activating protein)	167
47.	Q5HEU0	2204	Signal transduction protein TRAP (Target of RNAIII-activating protein)	167
48.	Q6G8A1	2208	Signal transduction protein TRAP (Target of RNAIII-activating protein)	167
49.	Q6GFM2	2207	Signal transduction protein TRAP (Target of RNAIII-activating protein)	167
50.	Q7A2Q4	2205	Signal transduction protein TRAP (Target of RNAIII-activating protein)	167
51.	Q7A4W3	2206	Signal transduction protein TRAP (Target of RNAIII-activating protein)	167
52.	Q8NVW1	2209	Signal transduction protein TRAP (Target of RNAIII-activating protein)	167
53.	P60609	2219	Transcriptional regulatory protein LytR (Sensory transduction protein LytR)	246
54.	P60610	2220	Transcriptional regulatory protein LytR (Sensory transduction protein LytR)	246
55.	Q2FK09	2216	Transcriptional regulatory protein LytR (Sensory transduction protein LytR)	246
56.	Q2YV67	2217	Transcriptional regulatory protein LytR (Sensory transduction protein LytR)	246

57.	Q5HJB5	2218	Transcriptional regulatory protein LytR (Sensory transduction protein LytR)	246
58.	Q6GCL1	2222	Transcriptional regulatory protein LytR (Sensory transduction protein LytR)	246
59.	Q6GK51	2221	Transcriptional regulatory protein LytR (Sensory transduction protein LytR)	246
60.	Q8NYH3	2223	Transcriptional regulatory protein LytR (Sensory transduction protein LytR)	246
61.	Q5HFT0	2289	Transcriptional regulatory protein SrrA (Staphylococcal respiratory response protein A)	241
62.	Q6G972	2198	Transcriptional regulatory protein SrrA (Staphylococcal respiratory response protein A)	241
63.	Q6GGK6	2283	Transcriptional regulatory protein SrrA (Staphylococcal respiratory response protein A)	241
64.	Q7A0U4	2200	Transcriptional regulatory protein SrrA (Staphylococcal respiratory response protein A)	241
65.	Q7A2R6	2196	Transcriptional regulatory protein SrrA (Staphylococcal respiratory response protein A)	241
66.	Q7A5H6	2290	Transcriptional regulatory protein SrrA (Staphylococcal respiratory response protein A)	241
67.	Q9L524	2199	Transcriptional regulatory protein SrrA (Staphylococcal respiratory response protein A)	241
