# Supplementary material for: 'Targeting' the search: An upgraded structural and functional repository of antimicrobial peptides for biofilm studies (B-AMP v2.0) with a focus on biofilm protein targets
Source: Front Cell Infect Microbiol. 2022 Oct 18;12:1020391. doi: 10.3389/fcimb.2022.1020391 (PMC9623296; doi:10.3389/fcimb.2022.1020391)
Supplement: Supplementary Table 10 — Full list of AMP-sortase C docking models for C. striatum analyzed using MD simulations (preference scores 10, 9, 8, 1 and 0). [file Table_10.pdf]

Supplementary File 10: Full list of AMP-sortase C docking models for *C. striatum* analyzed using MD simulations

Standard

Molecular Dynamics trajectory movie: https://drive.google.com/drive/folders/1mNLQzoKsZHoPzKCD_f8yiQPefJg0EZv4?usp=sharing

**Pep ID**	**0ns snapshot of AMP bound with mutated Class C Sortase protein**	**20ns snapshot of AMP bound with mutated Class C Sortase protein**	**AMP-mutated Class C Sortase interactions at 0ns of the MD simulation**	**AMP-mutated Class C Sortase interactions at 20ns of the MD simulation**
Pep1	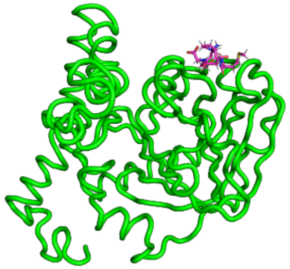	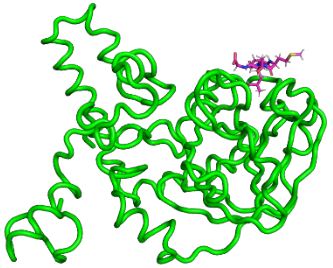	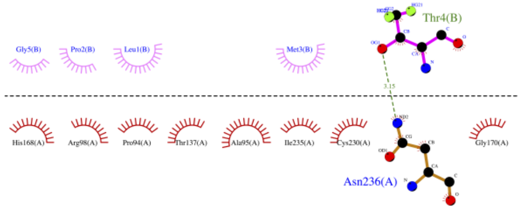	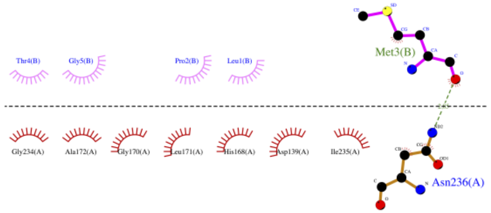

Preference score 10

Molecular Dynamics trajectory movies:
https://drive.google.com/drive/folders/11Wk2zU27oV5Y5mAXLUJ3UgBmtSMkkcdP?usp=sharing

Pep ID	0ns snapshot of AMP bound with mutated Class C Sortase protein	20ns snapshot of AMP bound with mutated Class C Sortase protein	AMP-mutated Class C Sortase interactions at 0ns of the MD simulation	AMP-mutated Class C Sortase interactions at 20ns of the MD simulation
Pep1437	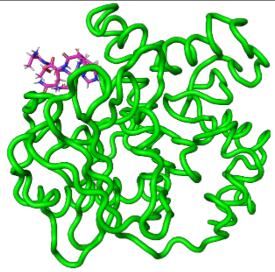	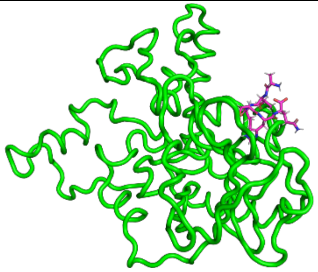	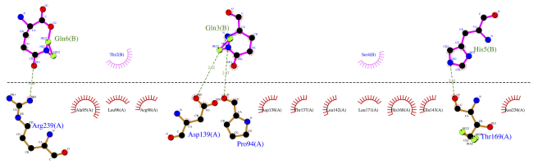	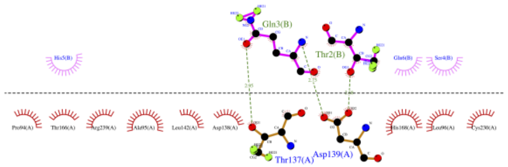
Pep4021	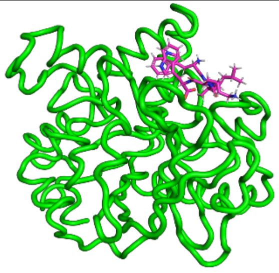	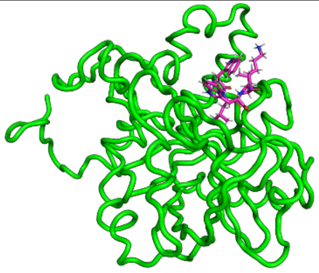	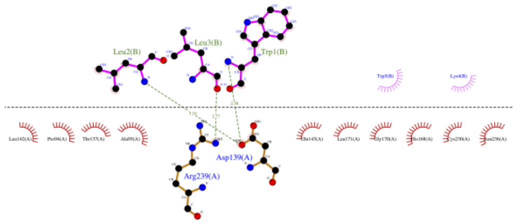	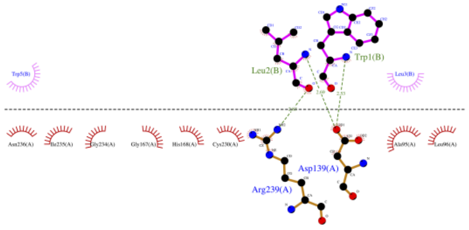
Pep4583	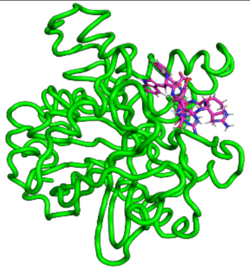	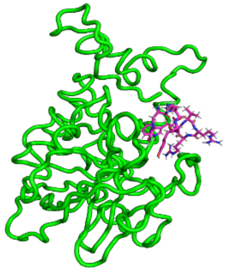	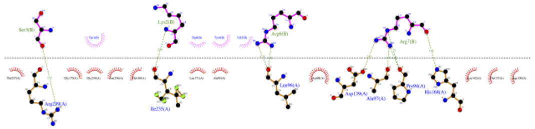	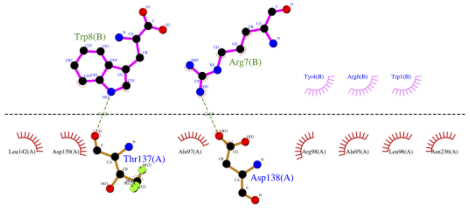

Pep ID	0ns snapshot of AMP bound with mutated Class C Sortase protein	20ns snapshot of AMP bound with mutated Class C Sortase protein	AMP-mutated Class C Sortase interactions at 0ns of the MD simulation	AMP-mutated Class C Sortase interactions at 20ns of the MD simulation
Pep4707	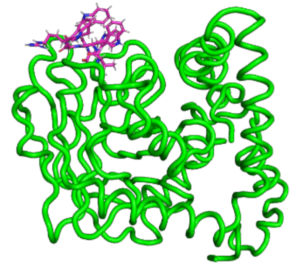	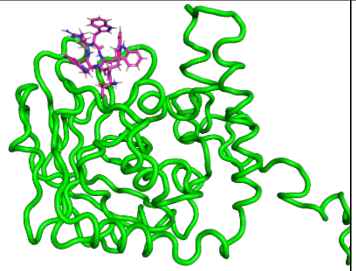	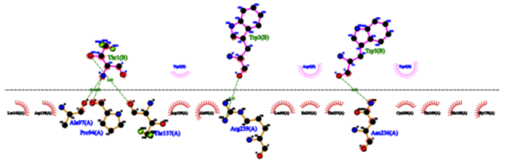	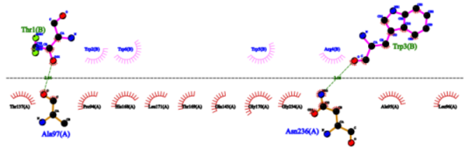
Pep4785	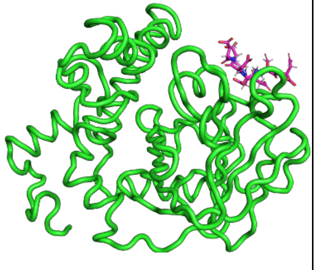	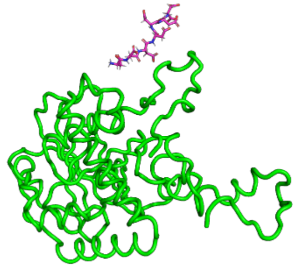	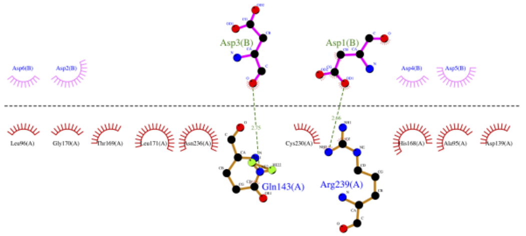	**AMP drifts away from the complex**
Pep5494	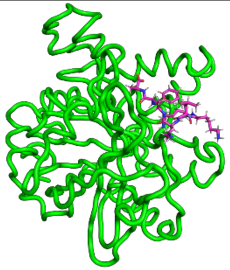	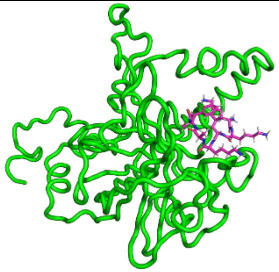	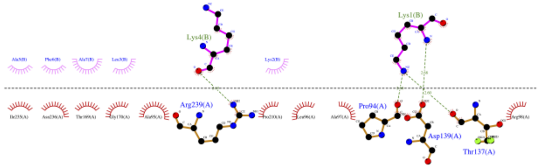	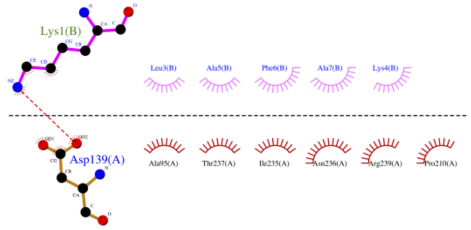
Pep5496	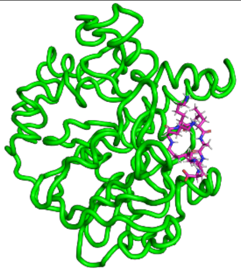	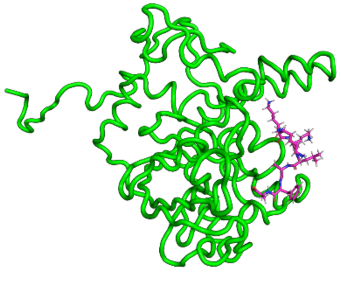	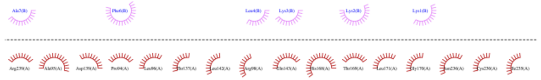	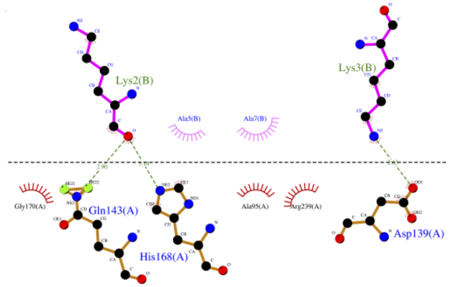

Preference Score 9

Molecular Dynamics trajectory movies: https://drive.google.com/drive/folders/1eZxdvx22BRFIOHNBrk5rkGDaTk49ZWH1?usp=sharing

Pep ID	0ns snapshot of AMP bound with mutated Class C Sortase protein	20ns snapshot of AMP bound with mutated Class C Sortase protein	AMP-mutated Class C Sortase interactions at 0ns of the MD simulation	AMP-mutated Class C Sortase interactions at 20ns of the MD simulation
Pep1218	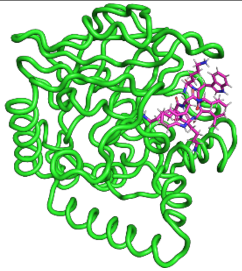	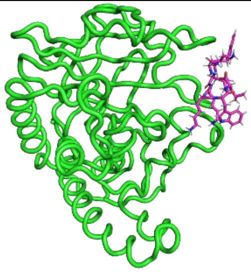	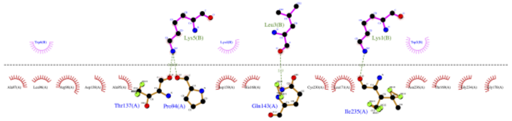	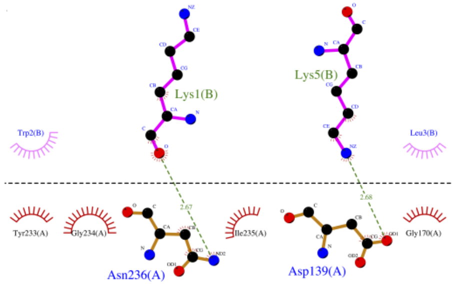
Pep4708	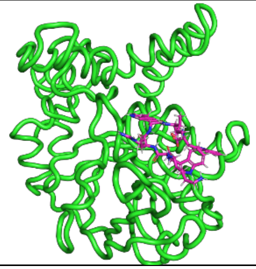	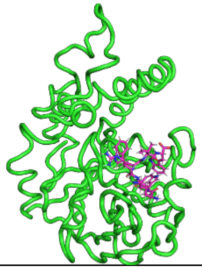	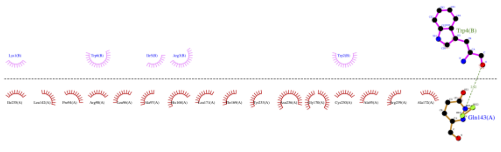	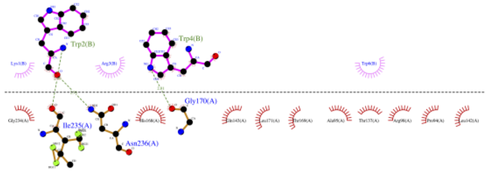
Pep4931	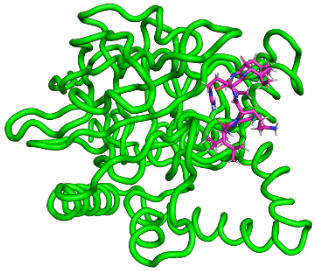	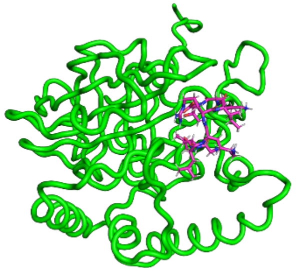	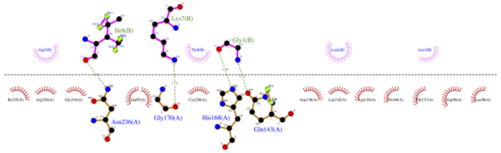	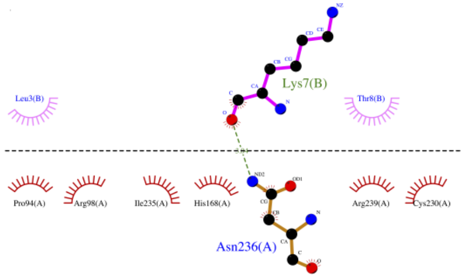

<table>
<tr><th>Pep ID</th><th>0ns snapshot of AMP bound with mutated Class C Sortase protein</th><th>20ns snapshot of AMP bound with mutated Class C Sortase protein</th><th>AMP-mutated Class C Sortase interactions at 0ns of the MD simulation</th><th>AMP-mutated Class C Sortase interactions at 20ns of the MD simulation</th></tr>
<tr><td>Pep4934</td><td>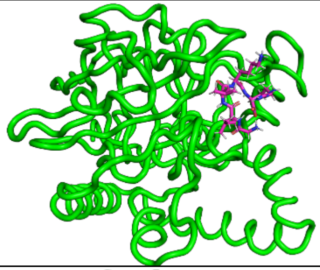</td><td>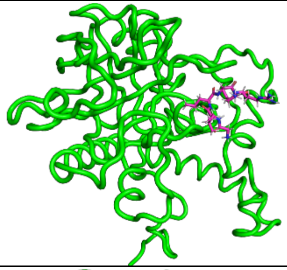</td><td>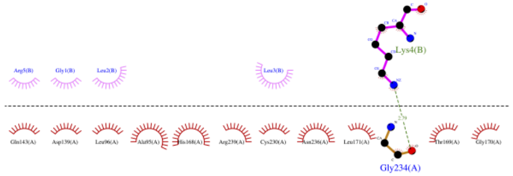
</td><td>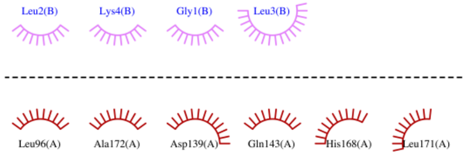
</td></tr>
<tr><td>Pep5482</td><td>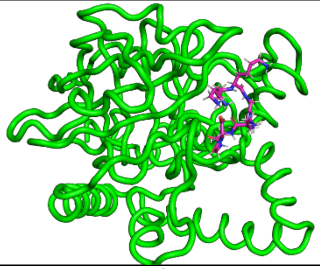</td><td>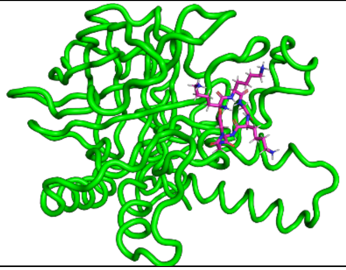</td><td>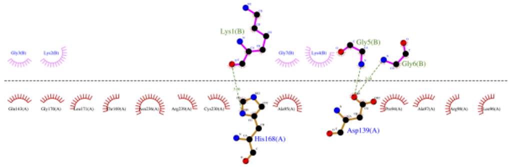
</td><td>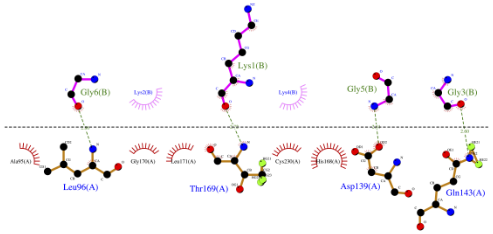
</td></tr>
<tr><td>Pep5508</td><td>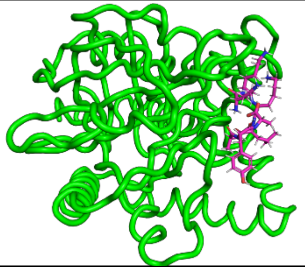</td><td>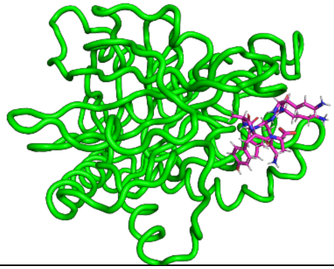</td><td>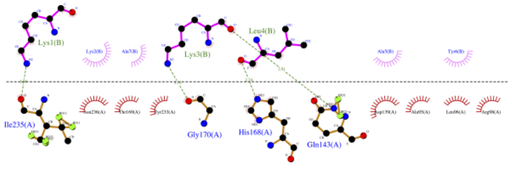
</td><td>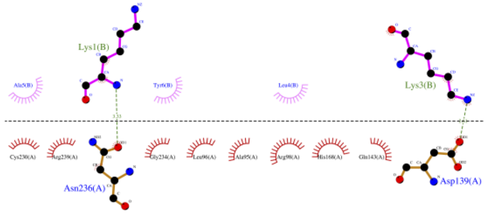
</td></tr>
</table>

Preference score 8

Molecular Dynamics trajectory movies: https://drive.google.com/drive/folders/1uw3buKTkkfMW2E5G_8rweyftcupmQheP?usp=sharing

Pep ID	0ns snapshot of AMP bound with mutated Class C Sortase protein	20ns snapshot of AMP bound with mutated Class C Sortase protein	AMP-mutated Class C Sortase interactions at 0ns of the MD simulation	AMP-mutated Class C Sortase interactions at 20ns of the MD simulation
Pep51	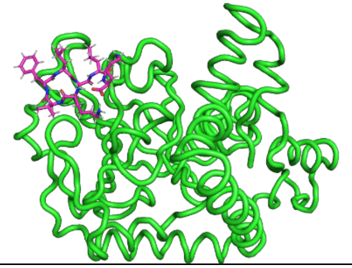	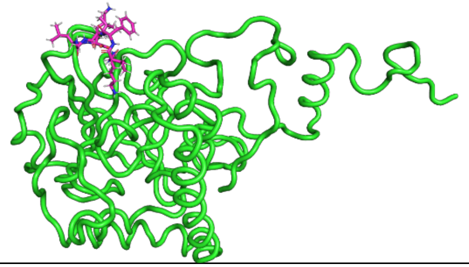	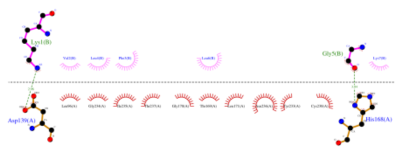	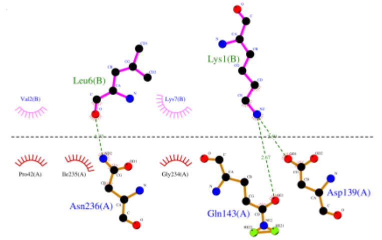
Pep168	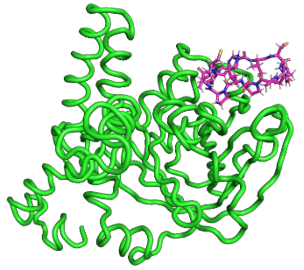	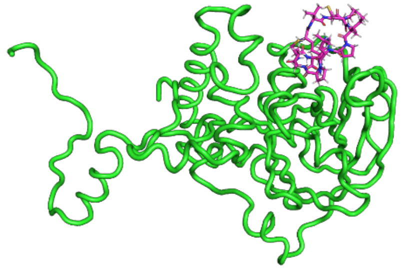	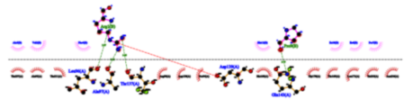	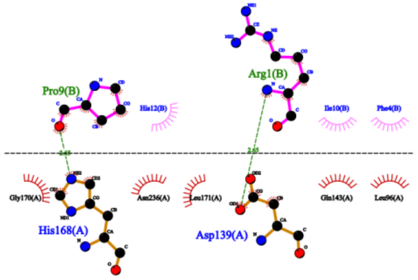
Pep696	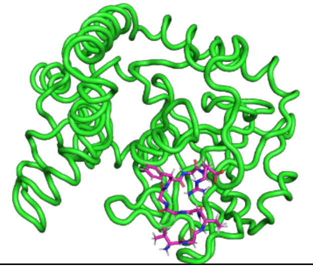	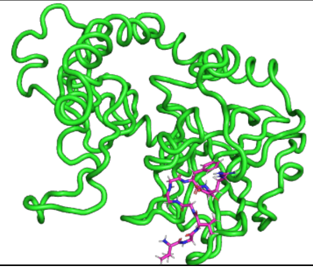	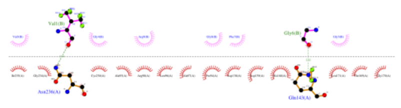	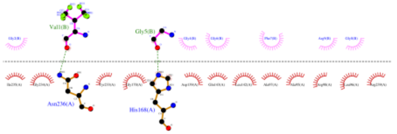

Pep ID	0ns snapshot of AMP bound with mutated Class C Sortase protein	20ns snapshot of AMP bound with mutated Class C Sortase protein	AMP-mutated Class C Sortase interactions at 0ns of the MD simulation	AMP-mutated Class C Sortase interactions at 20ns of the MD simulation
Pep782	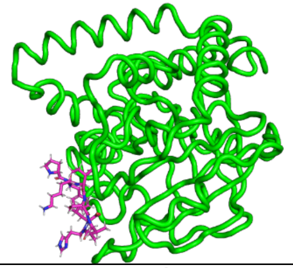	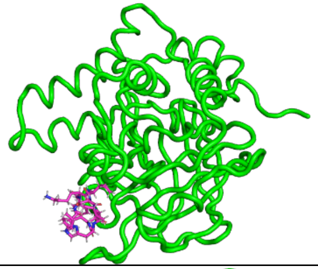	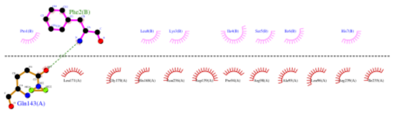	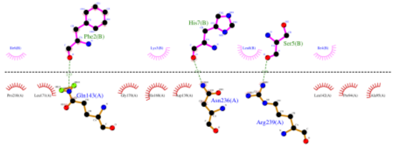
Pep996	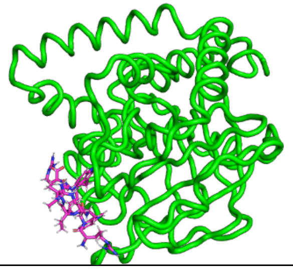	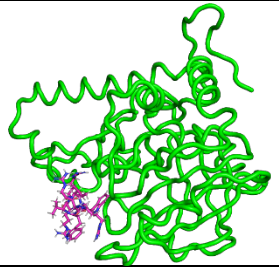	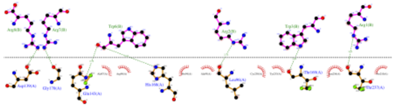	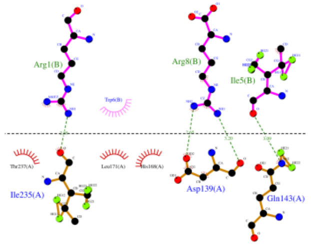
Pep1005	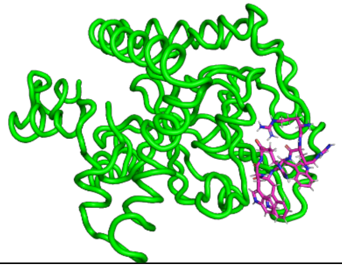	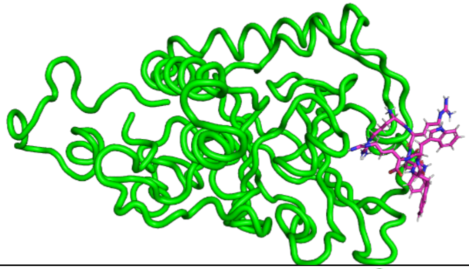	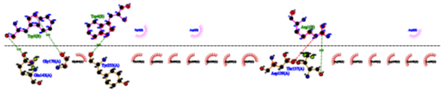	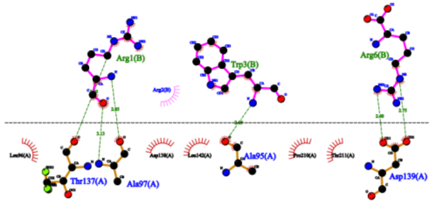
Pep1087	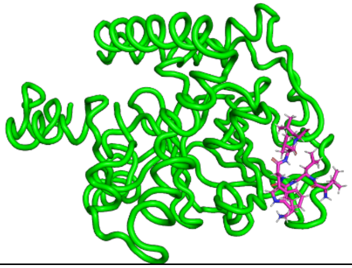	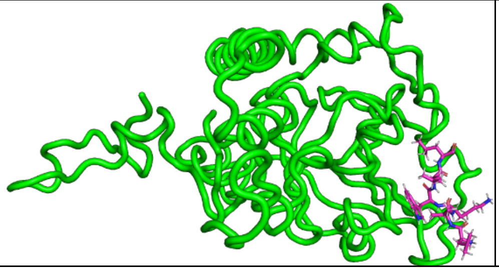	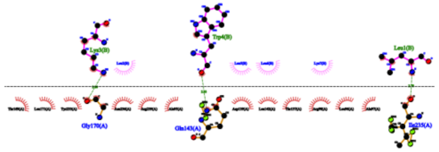	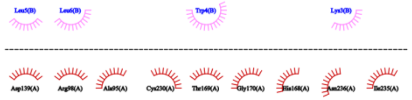

Pep ID	0ns snapshot of AMP bound with mutated Class C Sortase protein	20ns snapshot of AMP bound with mutated Class C Sortase protein	AMP-mutated Class C Sortase interactions at 0ns of the MD simulation	AMP-mutated Class C Sortase interactions at 20ns of the MD simulation
Pep1219	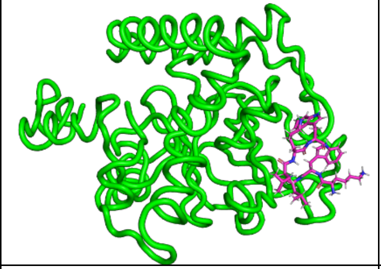	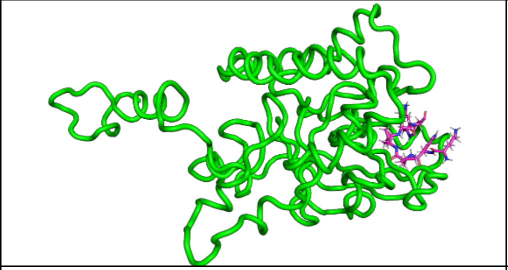	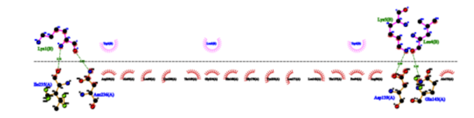	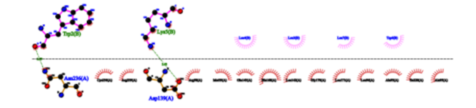
Pep1245	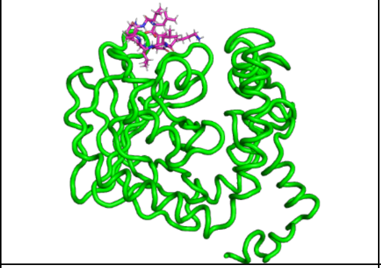	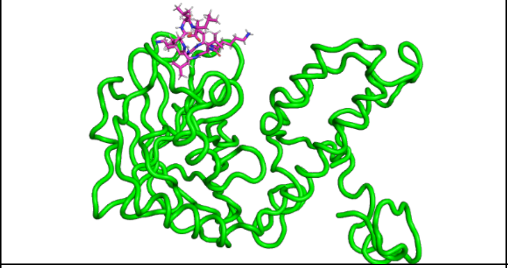	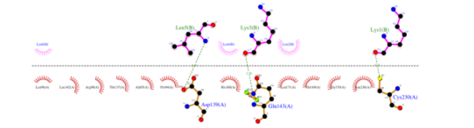	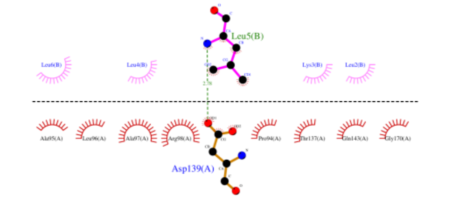
Pep2442	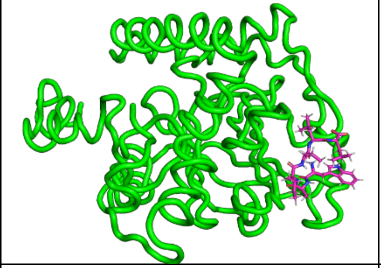	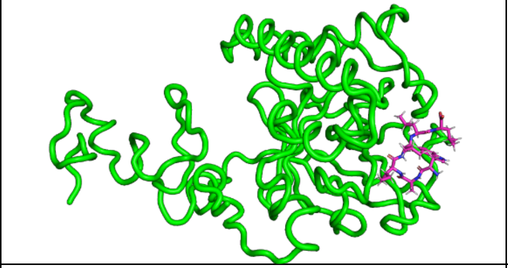	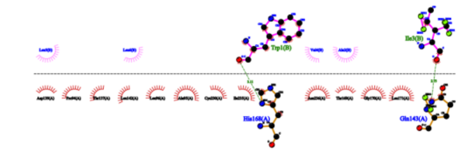	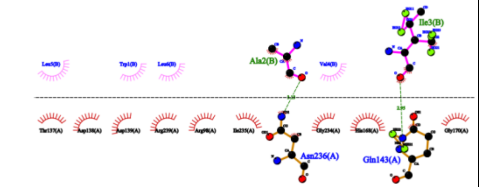
Pep3059	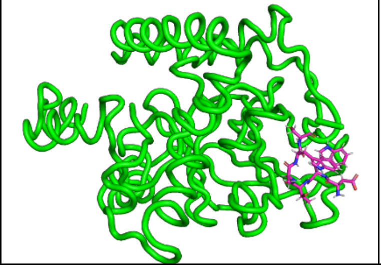	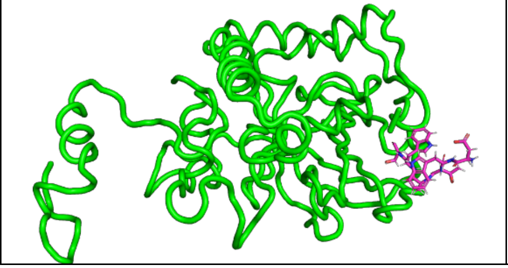	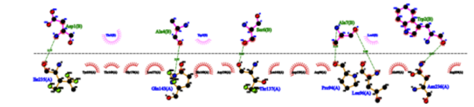	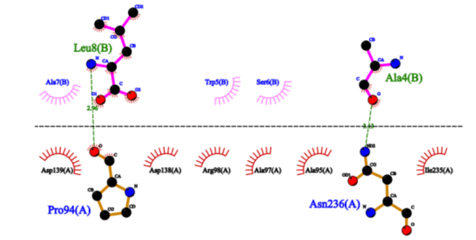

Pep ID	0ns snapshot of AMP bound with mutated Class C Sortase protein	20ns snapshot of AMP bound with mutated Class C Sortase protein	AMP-mutated Class C Sortase interactions at 0ns of the MD simulation	AMP-mutated Class C Sortase interactions at 20ns of the MD simulation
Pep3219	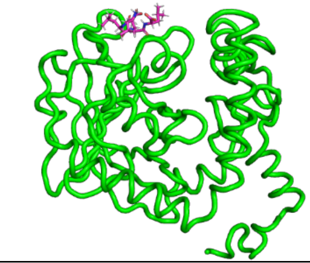	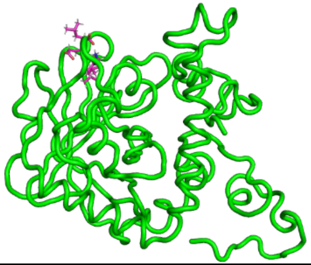	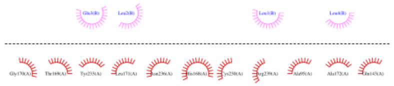	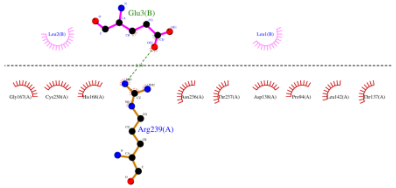
Pep3239	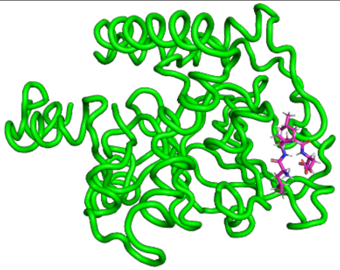	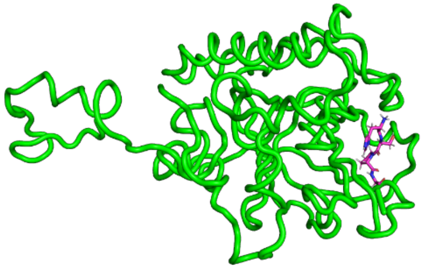	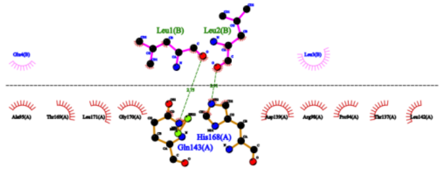	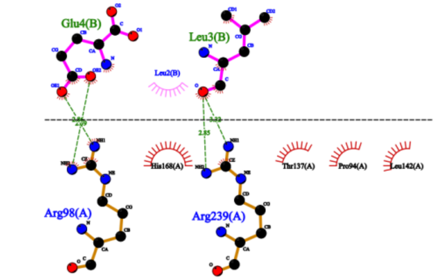
Pep3240	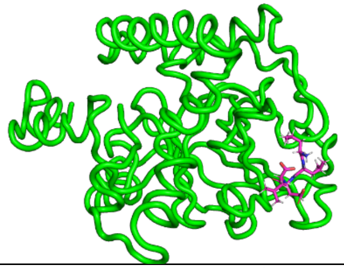	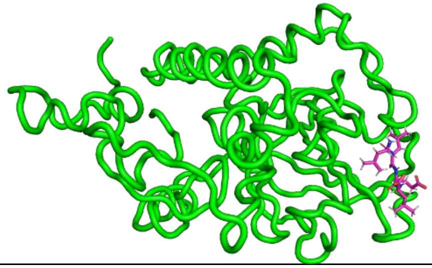	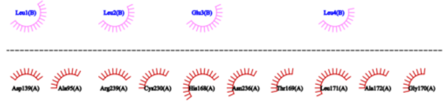	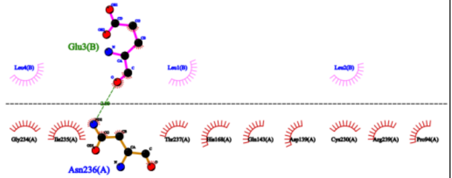
Pep3292	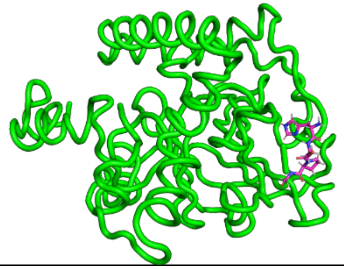	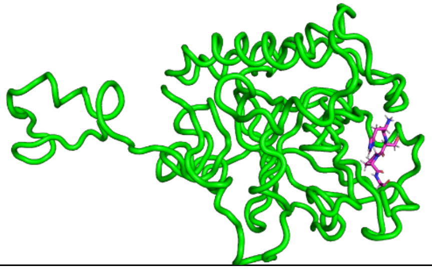	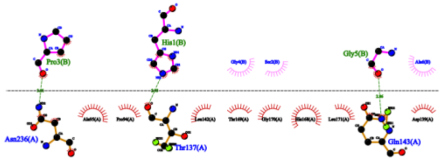	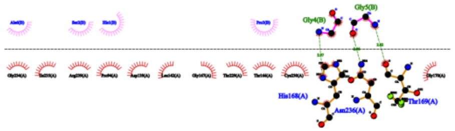

Pep ID	0ns snapshot of AMP bound with mutated Class C Sortase protein	20ns snapshot of AMP bound with mutated Class C Sortase protein	AMP-mutated Class C Sortase interactions at 0ns of the MD simulation	AMP-mutated Class C Sortase interactions at 20ns of the MD simulation
Pep3296	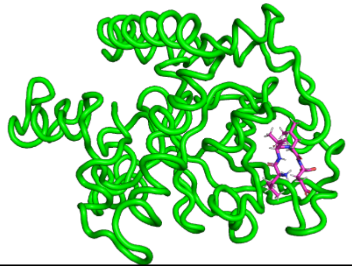	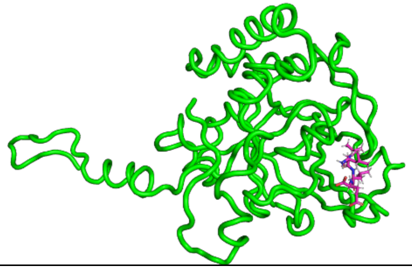	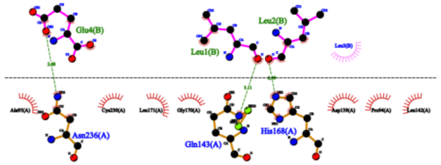	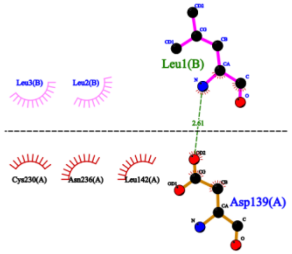
Pep3297	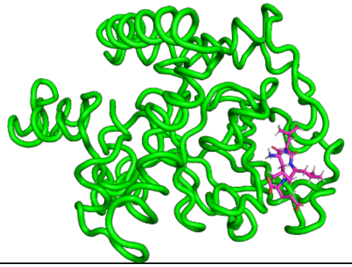	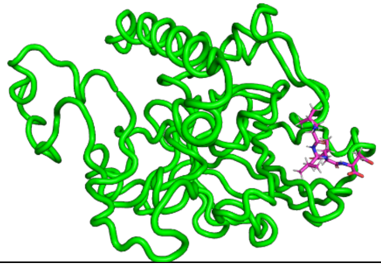	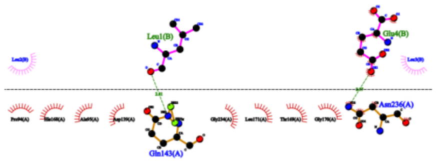	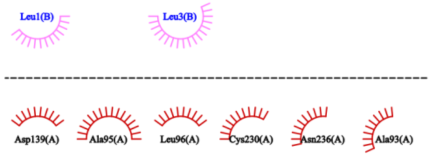
Pep3298	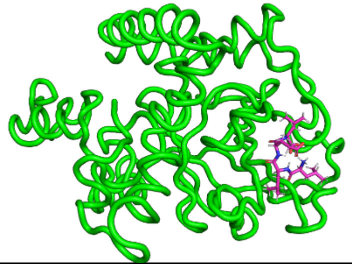	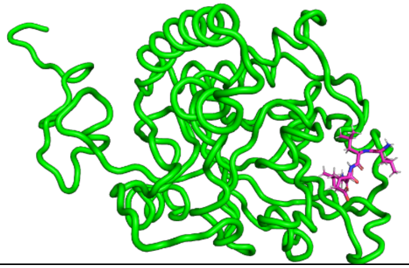	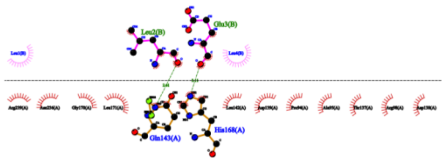	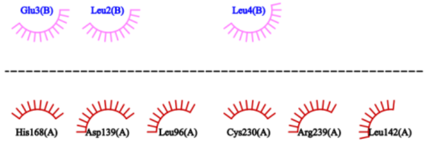
Pep3308	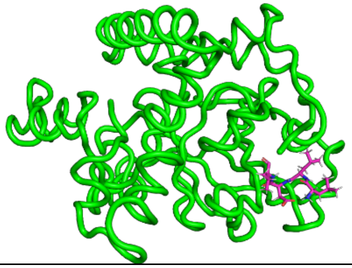	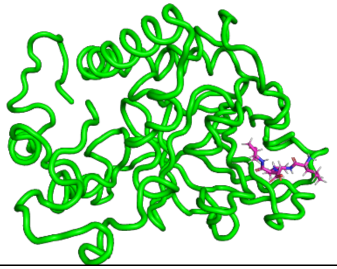	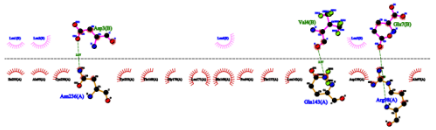	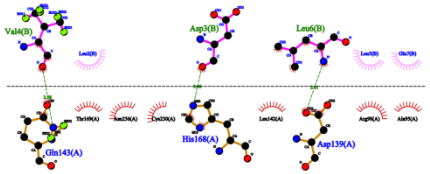

Pep ID	0ns snapshot of AMP bound with mutated Class C Sortase protein	20ns snapshot of AMP bound with mutated Class C Sortase protein	AMP-mutated Class C Sortase interactions at 0ns of the MD simulation	AMP-mutated Class C Sortase interactions at 20ns of the MD simulation
Pep3366	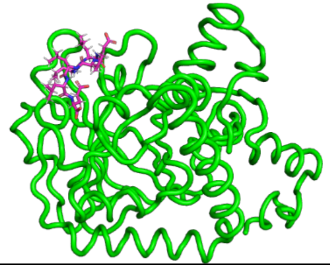	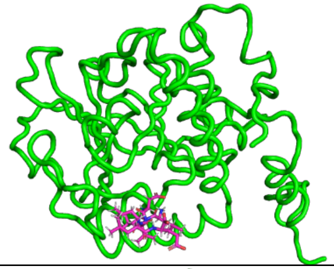	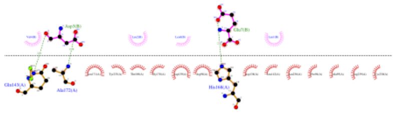	**No interactions**
Pep3461	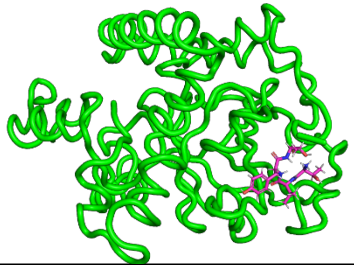	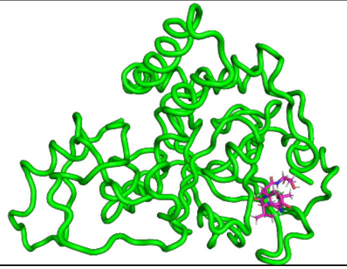	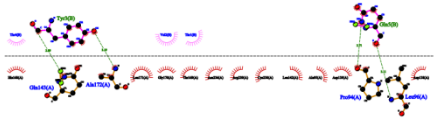	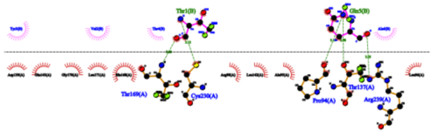
Pep3475	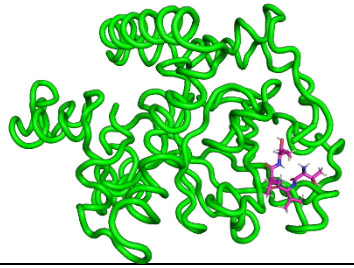	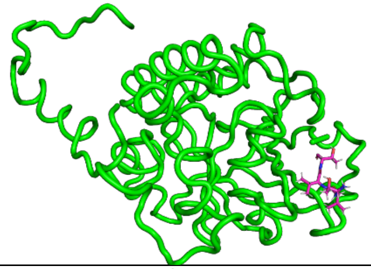	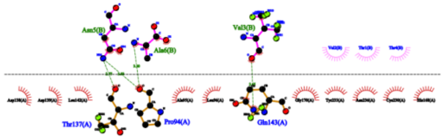	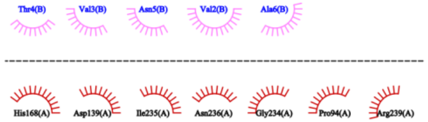
Pep4020	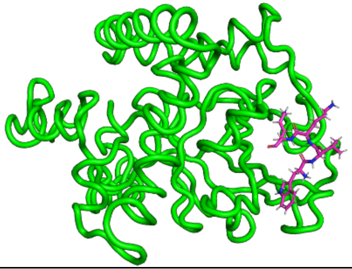	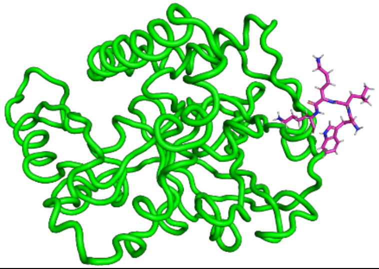	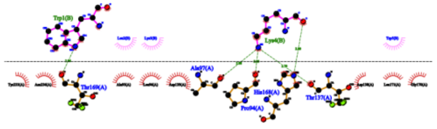	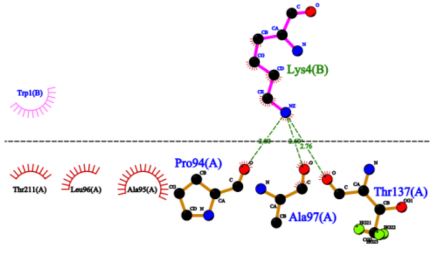

Pep ID	0ns snapshot of AMP bound with mutated Class C Sortase protein	20ns snapshot of AMP bound with mutated Class C Sortase protein	AMP-mutated Class C Sortase interactions at 0ns of the MD simulation	AMP-mutated Class C Sortase interactions at 20ns of the MD simulation
Pep4674	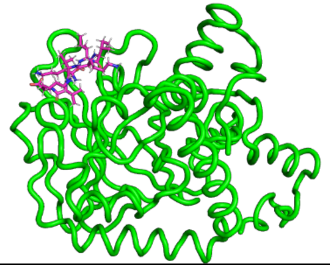	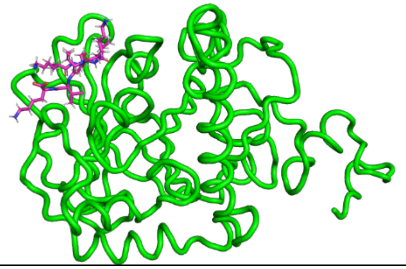	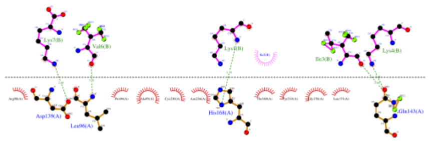	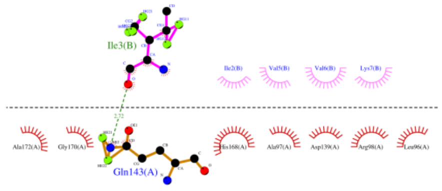
Pep4709	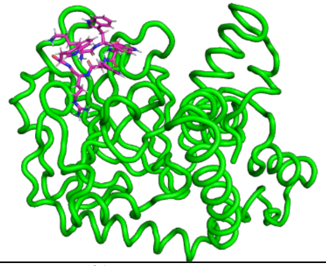	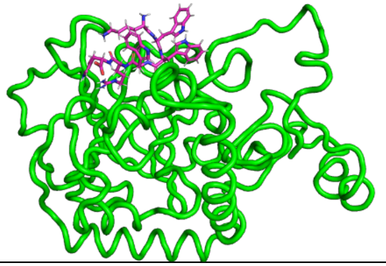	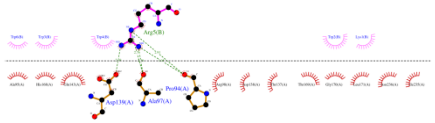	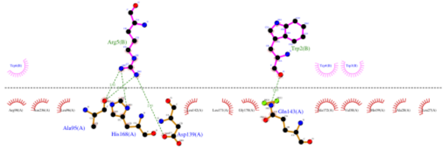
Pep4710	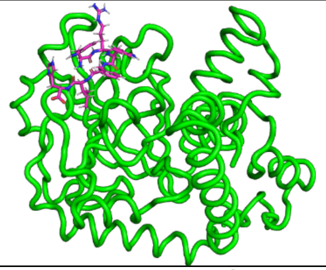	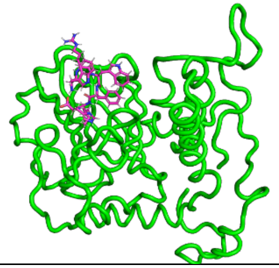	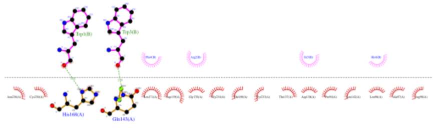	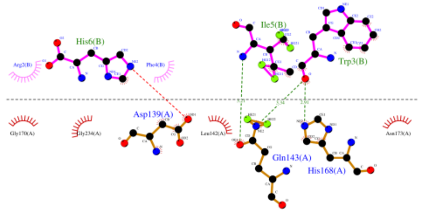
Pep4932	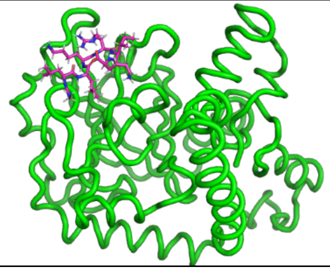	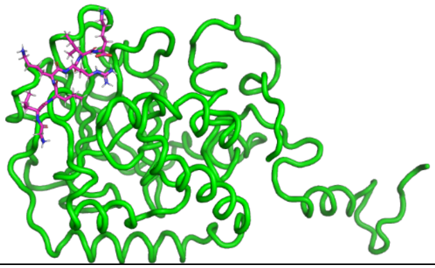	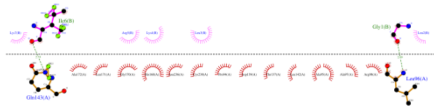	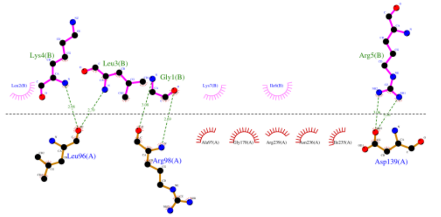

Pep ID	0ns snapshot of AMP bound with mutated Class C Sortase protein	20ns snapshot of AMP bound with mutated Class C Sortase protein	AMP-mutated Class C Sortase interactions at 0ns of the MD simulation	AMP-mutated Class C Sortase interactions at 20ns of the MD simulation
Pep5241	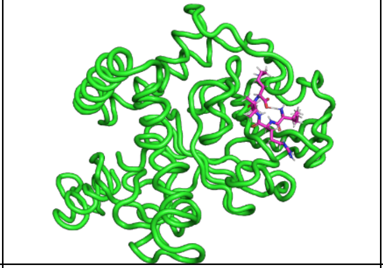	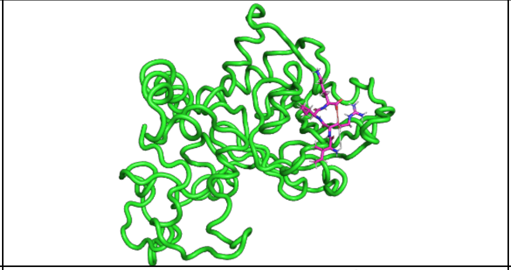	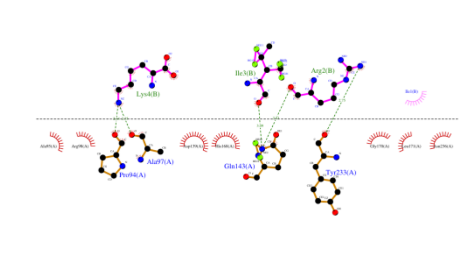	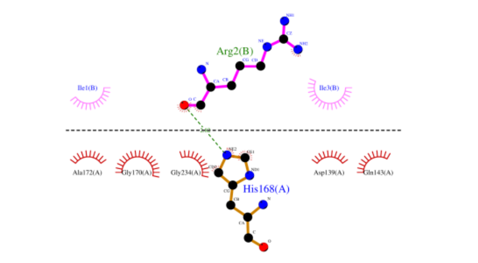
Pep5244	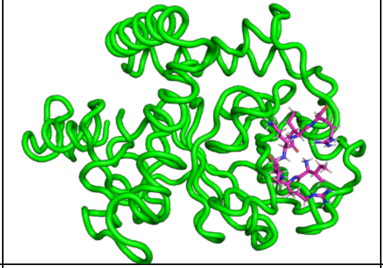	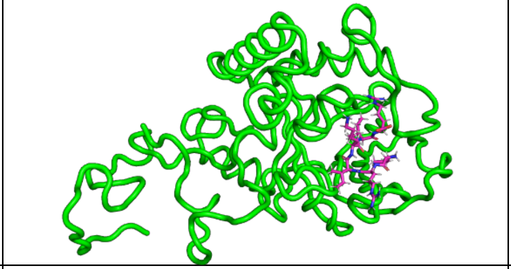	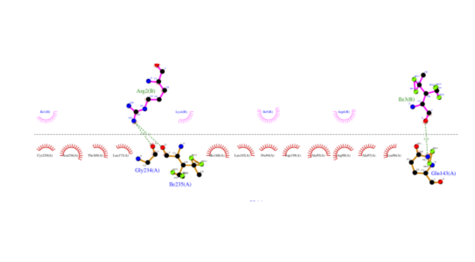	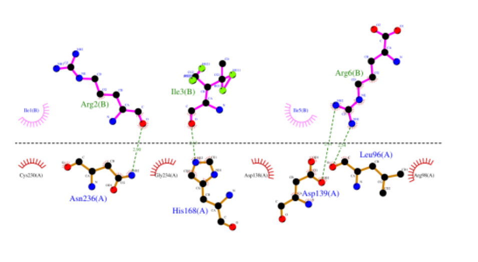
Pep5363	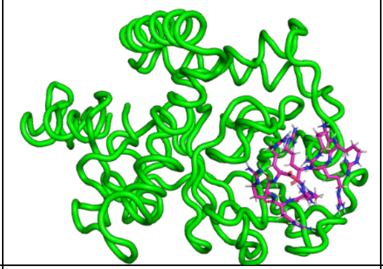	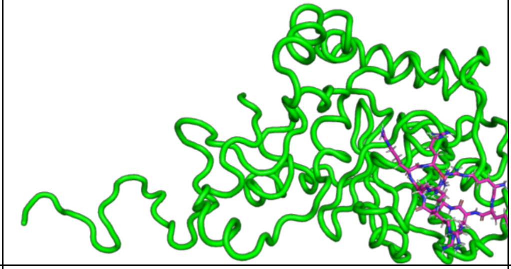	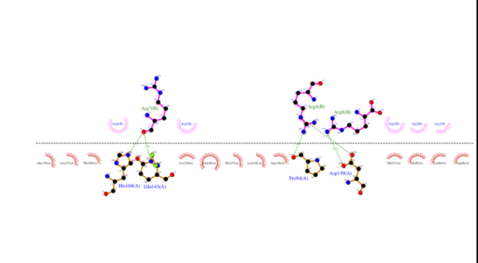	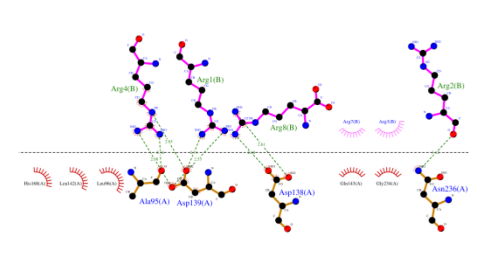
Pep5483	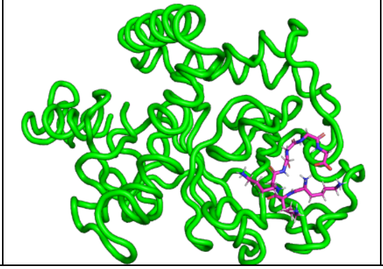	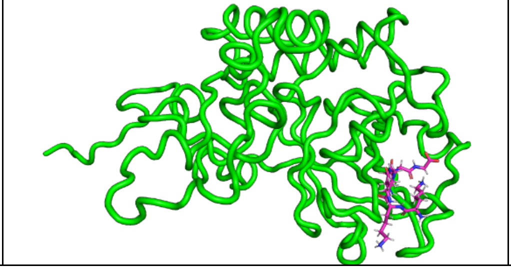	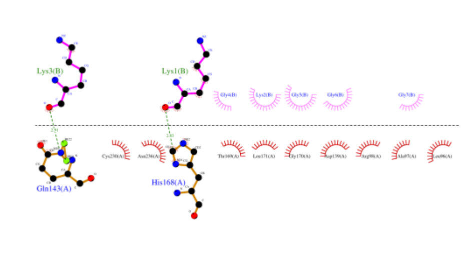	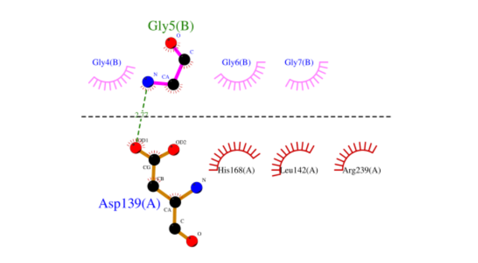

Pep ID	0ns snapshot of AMP bound with mutated Class C Sortase protein	20ns snapshot of AMP bound with mutated Class C Sortase protein	AMP-mutated Class C Sortase interactions at 0ns of the MD simulation	AMP-mutated Class C Sortase interactions at 20ns of the MD simulation
Pep5485	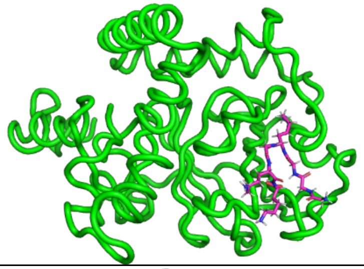	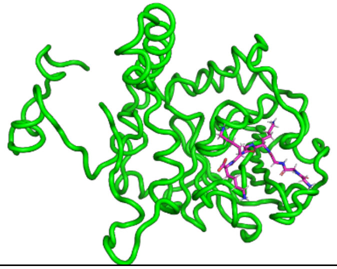	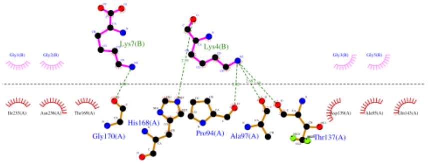	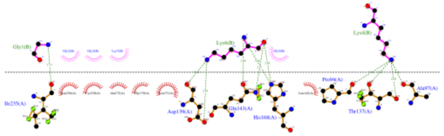
Pep5487	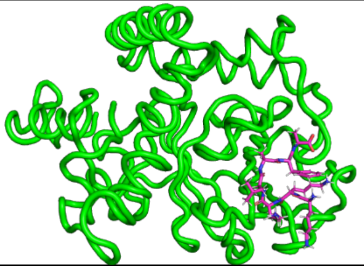	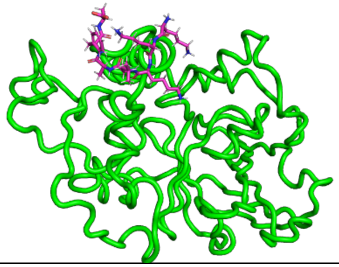	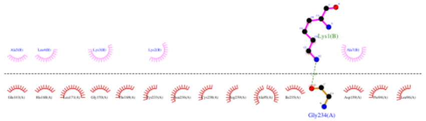	**AMP drifts away from the complex**
Pep5489	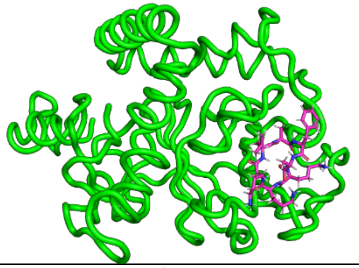	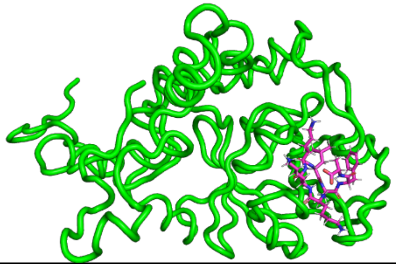	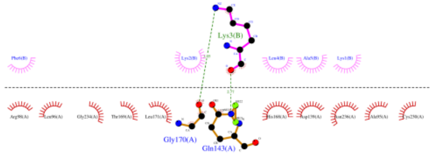	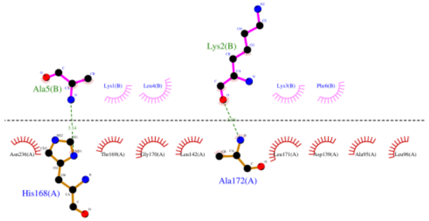
Pep5491	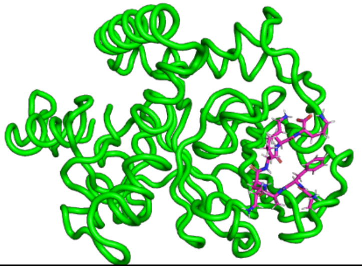	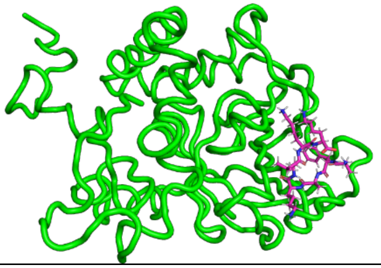	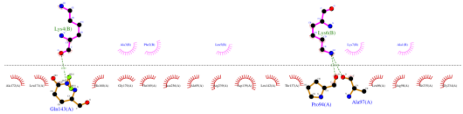	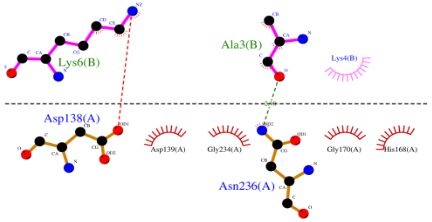

Pep ID	0ns snapshot of AMP bound with mutated Class C Sortase protein	20ns snapshot of AMP bound with mutated Class C Sortase protein	AMP-mutated Class C Sortase interactions at 0ns of the MD simulation	AMP-mutated Class C Sortase interactions at 20ns of the MD simulation
Pep5492	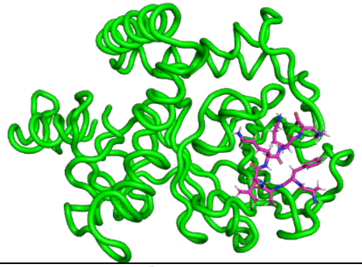	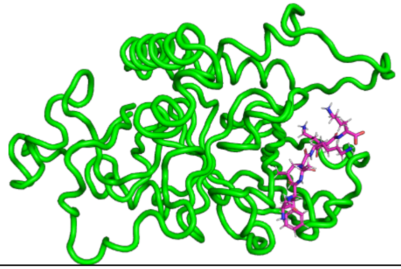	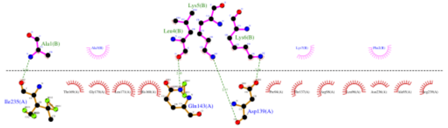	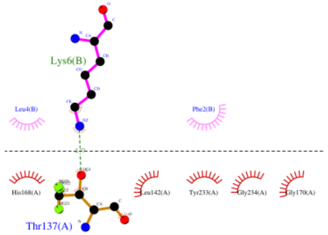
Pep5497	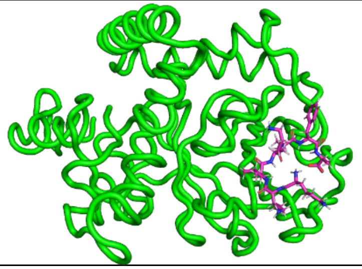	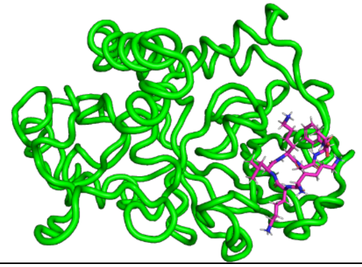	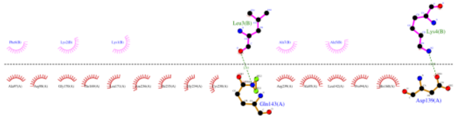	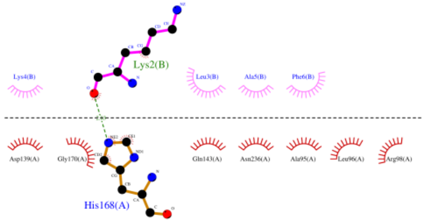
Pep5499	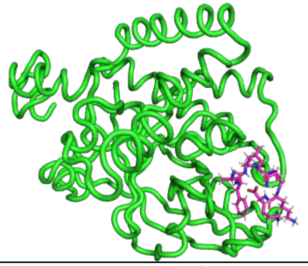	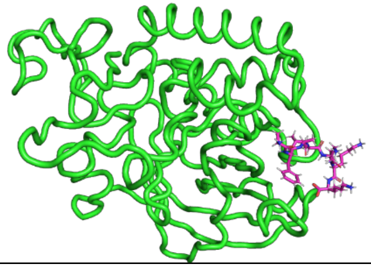	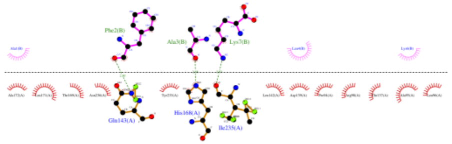	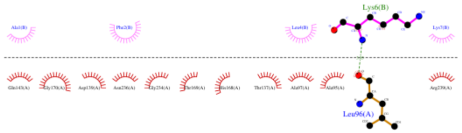
Pep5501	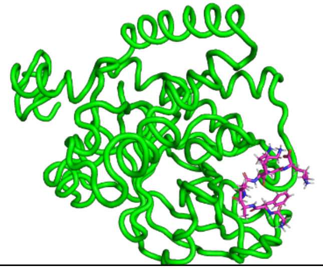	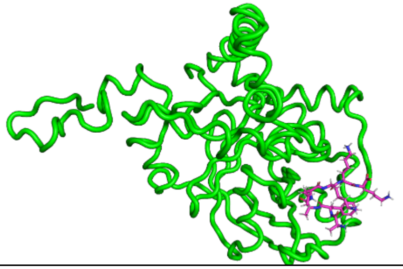	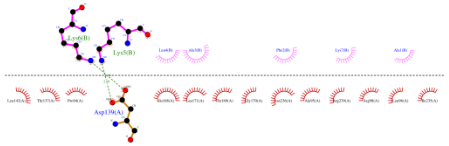	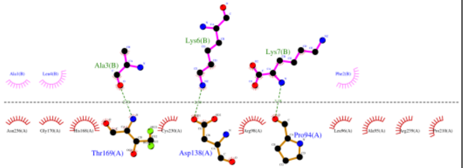

Pep ID	0ns snapshot of AMP bound with mutated Class C Sortase protein	20ns snapshot of AMP bound with mutated Class C Sortase protein	AMP-mutated Class C Sortase interactions at 0ns of the MD simulation	AMP-mutated Class C Sortase interactions at 20ns of the MD simulation
Pep5502	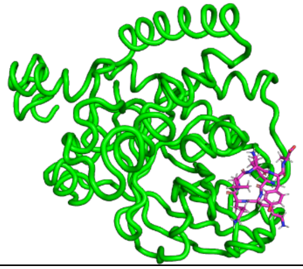	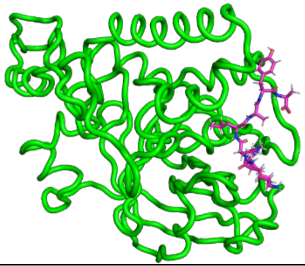	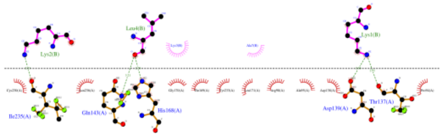	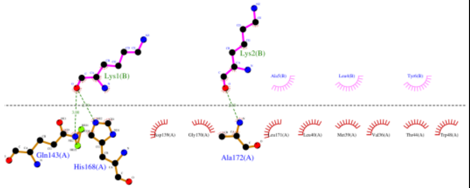
Pep5505	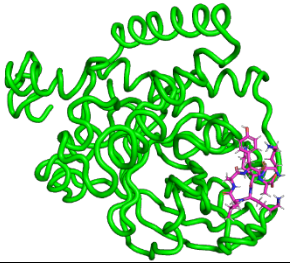	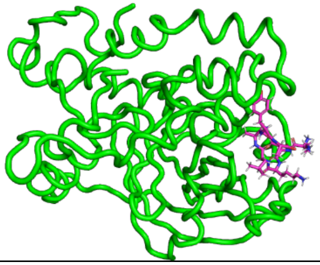	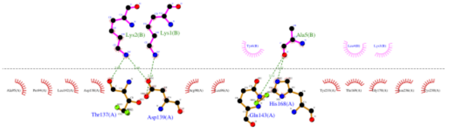	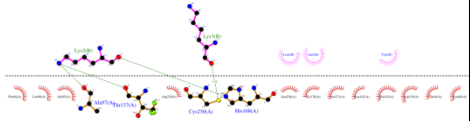
Pep5509	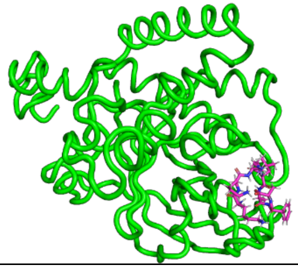	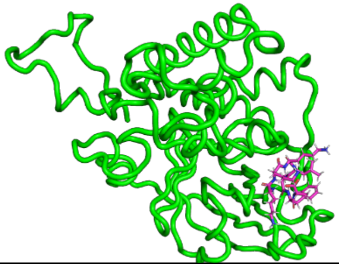	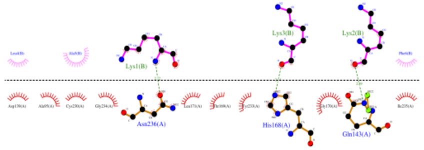	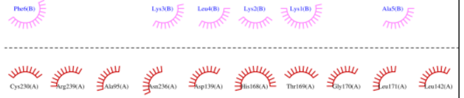

Preference Score 1

Molecular Dynamics trajectory movies: https://drive.google.com/drive/folders/1T24rbeKqzZcbryf5HiSG3wcE_fzgFeGu?usp=sharing

Pep ID	0ns snapshot of AMP bound with mutated Class C Sortase protein	20ns snapshot of AMP bound with mutated Class C Sortase protein	AMP-mutated Class C Sortase interactions at 0ns of the MD simulation	AMP-mutated Class C Sortase interactions at 20ns of the MD simulation
Pep3309	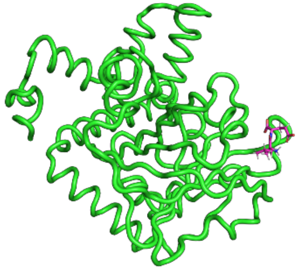	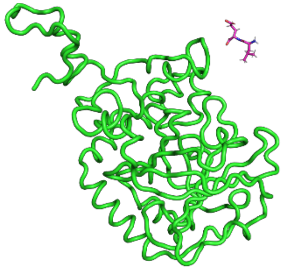	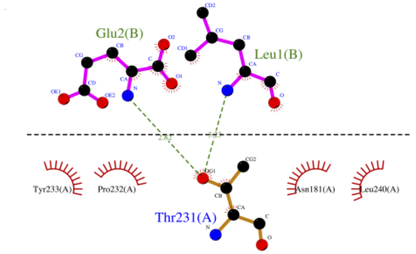	AMP drifts away from the complex
Pep5236	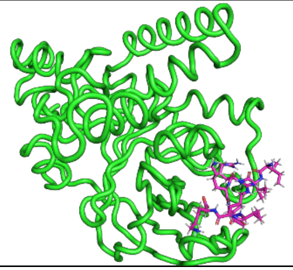	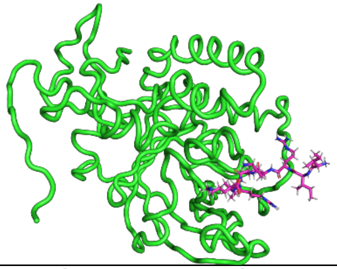	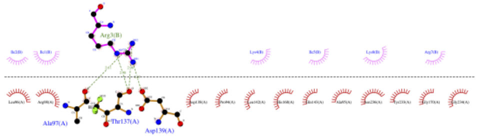	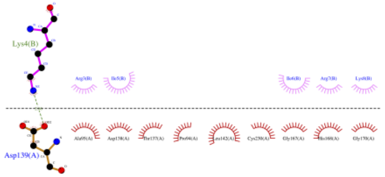
Pep5493	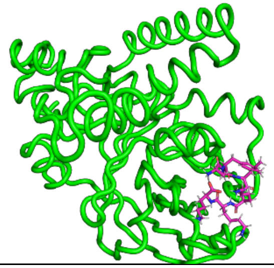	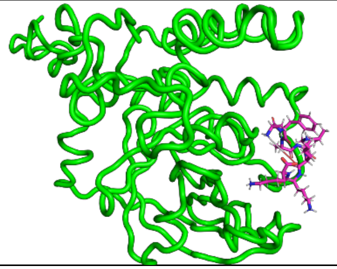	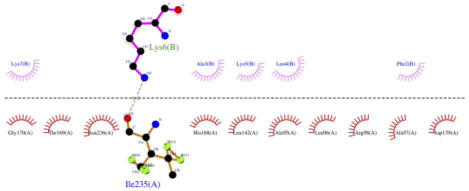	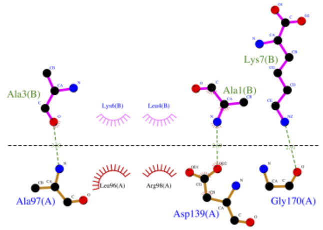

Preference Score 0

Molecular Dynamics trajectory movie: https://drive.google.com/drive/folders/1MRLEgSEre-ixhwyXEjFiBRyxlfFPmj1u?usp=sharing

**Pep ID**	**0ns snapshot of AMP bound with mutated Class C Sortase protein**	**20ns snapshot of AMP bound with mutated Class C Sortase protein**	**AMP-mutated Class C Sortase interactions at 0ns of the MD simulation**	**AMP-mutated Class C Sortase interactions at 20ns of the MD simulation**
Pep993	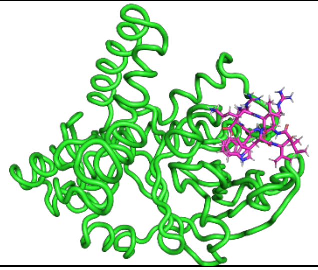	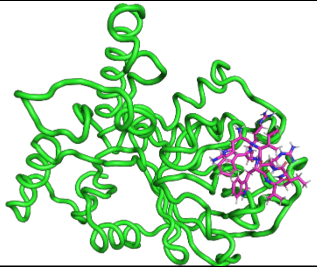	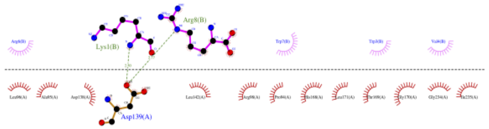	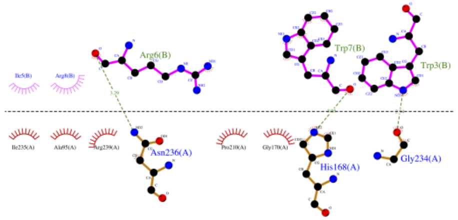
