# Supplementary material for: 'Targeting' the search: An upgraded structural and functional repository of antimicrobial peptides for biofilm studies (B-AMP v2.0) with a focus on biofilm protein targets
Source: Front Cell Infect Microbiol. 2022 Oct 18;12:1020391. doi: 10.3389/fcimb.2022.1020391 (PMC9623296; doi:10.3389/fcimb.2022.1020391)
Supplement: Supplementary File 11 — Filtered list of 2035 AMPs with both anti-Gram positive and anti-Gram negative activity used for in silico molecular docking against Target 37 of P. aeruginosa and Target 1 of S. aureus. [file Table_11.pdf]

S.no.	PepID	DRAMP_ID	Name of the AMP	Activity
1	9	DRAMP00089	Bacteriocin E50-52 (Preclinical)	Antibacterial, Anti-Gram+, Anti-Gram-, Antimicrobial
2	13	DRAMP00107	Bacteriocin L-1077	Antibacterial, Anti-Gram+, Anti-Gram-, Antimicrobial
3	18	DRAMP00136	Enterocin E-760 (Bacteriocin)	Antibacterial, Anti-Gram+, Anti-Gram-, Antimicrobial
4	19	DRAMP00171	Lactocyclicin Q (Bacteriocin)	Antibacterial, Anti-Gram+, Anti-Gram-, Antimicrobial
5	34	DRAMP00336	ChaC7 (Chassatide C7; uncyclotides; Plant defensin)	Antibacterial, Anti-Gram+, Anti-Gram-, Antimicrobial
6	35	DRAMP00337	ChaC8 (Chassatide C8; uncyclotides; Plant defensin)	Antibacterial, Anti-Gram+, Anti-Gram-, Antimicrobial
7	36	DRAMP00338	ChaC11 (Chassatide C11; uncyclotides; Plant defensin)	Antibacterial, Anti-Gram+, Anti-Gram-, Antimicrobial
8	44	DRAMP00431	Defensin-like protein 2 (Cp-thionin II; Cp-thionin-2; Gamma- thionin	Antibacterial, Anti-Gram+, Anti-Gram-, Antimicrobial
9	49	DRAMP00764	Piceain 1 (Plants)	Antibacterial, Antifungal, Anti-Gram+, Anti-Gram-, Antimicrobial
10	50	DRAMP00765	Piceain 2 (Plants)	Antibacterial, Antifungal, Anti-Gram+, Anti-Gram-, Antimicrobial
11	51	DRAMP00766	JCpep7 (Plants)	Antibacterial, Anti-Gram+, Anti-Gram-, Antimicrobial
12	52	DRAMP00774	Hedyotide B1 (hB1; Plants)	Antibacterial, Anti-Gram+, Anti-Gram-, Antimicrobial
13	53	DRAMP00795	Cliotide T1 (cT1; Plant defensin)	Antibacterial, Anticancer, Anti-Gram+, Anti-Gram-, Antimicrobial
14	54	DRAMP00798	Cliotide T4 (cT4; Plant defensin)	Antibacterial, Anticancer, Anti-Gram+, Anti-Gram-, Antimicrobial
15	55	DRAMP00856	Kalata-B1 (Plant defensin)	Antibacterial, Antifungal, Insecticidal, Anti-Gram+, Anti-Gram-, Antimicrobial
16	56	DRAMP00877	Circulin-A (CIRA; Plant defensin)	Antibacterial, Antifungal, Antiviral, Anti-Gram+, Anti-Gram-, Antimicrobial
17	57	DRAMP00878	Circulin-B (CIRB; Plant defensin)	Antibacterial, Antifungal, Antiviral, Anti-Gram+, Anti-Gram-, Antimicrobial
18	58	DRAMP01374	Odorranain-D1 (OdD1; Frogs, amphibians, animals)	Antimicrobial, Antibacterial, Antifungal, Anti-Gram+, Anti-Gram-,
19	59	DRAMP01373	Odorranain-C1 (OdC1; Frogs, amphibians, animals)	Antimicrobial, Antibacterial, Antifungal, Anti-Gram+, Anti-Gram-,
20	60	DRAMP01372	Odorranain-B1 (Frogs, amphibians, animals)	Antimicrobial, Antibacterial, Antifungal, Anti-Gram+, Anti- Gram-,
21	66	DRAMP01018	Cyclopsychotride-A (CPT; Plant defensin)	Antibacterial, Antifungal, Anti-Gram+, Anti-Gram-, Antimicrobial
22	68	DRAMP18193	Cathelicidin-related peptide crotalicidin	Antibacterial, Anti-Gram+, Anti-Gram-, Antimicrobial
23	69	DRAMP01064	Anticancerous peptide 1 (Cr-ACP1; Plants)	Anticancer, Antibacterial, Anti-Gram+, Anti-Gram-, Antimicrobial
24	75	DRAMP01088	Alyteserin-1Ma (toads, amphibians, animals)	Antibacterial, Antifungal, Anti-Gram+, Anti-Gram-, Antimicrobial
25	76	DRAMP01089	Alyteserin-1Mb (toads, amphibians, animals)	Antibacterial, Antifungal, Anti-Gram+, Anti-Gram-, Antimicrobial
26	77	DRAMP01090	Alyteserin-2Ma (toads, amphibians, animals)	Antibacterial, Antifungal, Anti-Gram+, Anti-Gram-, Antimicrobial
27	83	DRAMP02090	Brevinin-1Lb (Frogs, amphibians, animals)	Antibacterial, Anti-Gram+, Anti-Gram-, Antimicrobial
28	87	DRAMP01518	Esculentin-2L (Frogs, amphibians, animals)	Antimicrobial, Antibacterial, Antifungal, Anti-Gram+, Anti- Gram-,
29	90	DRAMP01516	Esculentin-2B (Frogs, amphibians, animals)	Antimicrobial, Antibacterial, Antifungal, Anti-Gram+, Anti-Gram-,
30	91	DRAMP02077	Brevinin-1Pb (Frogs, amphibians, animals)	Antimicrobial, Antibacterial, Antifungal, Anti-Gram+, Anti-Gram-,
31	98	DRAMP01162	Buforin-1 (Buforin I; Fragment of Histone H2A; toads, amphibians,	Antibacterial, Antifungal, Anti-Gram+, Anti-Gram-, Antimicrobial
32	99	DRAMP01163	Buforin-2 (Buforin II; Fragment of Histone H2A; toads, amphibians,	Antibacterial, Antifungal, Anti-Gram+, Anti-Gram-, Antimicrobial
33	100	DRAMP01164	Bombinin (toads, amphibians, animals)	Antibacterial, Anti-Gram+, Anti-Gram-, Antimicrobial
34	102	DRAMP01167	Hylaseptin-P1 (HSP1)	Antibacterial, Anti-Gram+, Anti-Gram-, Antimicrobial
35	103	DRAMP01170	Distinctin 2 (Frogs, amphibians, animals)	Antibacterial, Anti-Gram+, Anti-Gram-, Antimicrobial
36	104	DRAMP01174	Ocellatin-4 (Frogs, amphibians, animals)	Antibacterial, Anti-Gram+, Anti-Gram-, Antimicrobial
37	106	DRAMP01182	Ocellatin-P1 (Pentadactylin; Frogs, amphibians, animals)	Antibacterial, Anti-Gram+, Anti-Gram-, Antimicrobial
38	107	DRAMP01184	SPX(1-22)(truncated peptide of Syphaxin; Frogs, amphibians,	Antibacterial, Anti-Gram+, Anti-Gram-, Antimicrobial
39	108	DRAMP01185	SPX(1-16)(truncated peptide of Syphaxin; Frogs, amphibians,	Antibacterial, Anti-Gram+, Anti-Gram-, Antimicrobial
40	109	DRAMP01188	Chensinin-1ZHa (Frogs, amphibians, animals)	Antibacterial, Antifungal, Anti-Gram+, Anti-Gram-, Antimicrobial
41	110	DRAMP01189	Andersonin-W1 (Frogs, amphibians, animals)	Antibacterial, Antifungal, Anti-Gram+, Anti-Gram-, Antimicrobial
42	111	DRAMP01190	Andersonin-W2 (Frogs, amphibians, animals)	Antibacterial, Antifungal, Anti-Gram+, Anti-Gram-, Antimicrobial
43	112	DRAMP01191	Andersonin-X1 (Frogs, amphibians, animals)	Antibacterial, Antifungal, Anti-Gram+, Anti-Gram-, Antimicrobial
44	113	DRAMP01192	Andersonin-Y1 (Frogs, amphibians, animals)	Antibacterial, Antifungal, Anti-Gram+, Anti-Gram-, Antimicrobial
45	114	DRAMP01194	Andersonin-C1 (Frogs, amphibians, animals)	Antibacterial, Antifungal, Anti-Gram+, Anti-Gram-, Antimicrobial
46	115	DRAMP01195	Andersonin-D1 (Frogs, amphibians, animals)	Antibacterial, Antifungal, Anti-Gram+, Anti-Gram-, Antimicrobial
47	116	DRAMP01199	Hejiangin-A1 (Frogs, amphibians, animals)	Antibacterial, Antifungal, Anti-Gram+, Anti-Gram-, Antimicrobial
48	117	DRAMP01200	Hejiangin-F1 (frog, amphibians, animals)	Antibacterial, Antifungal, Anti-Gram+, Anti-Gram-, Antimicrobial
49	118	DRAMP01201	Schmackerin-C1 (Frogs, amphibians, animals)	Antibacterial, Antifungal, Anti-Gram+, Anti-Gram-, Antimicrobial
50	122	DRAMP01208	Pleurain-A1 (Pleurain A1; Frogs, amphibians, animals)	Antibacterial, Antifungal, Anti-Gram+, Anti-Gram-, Antimicrobial
51	123	DRAMP01209	Pleurain-A2 (Pleurain A2; Frogs, amphibians, animals)	Antibacterial, Antifungal, Anti-Gram+, Anti-Gram-, Antimicrobial
52	124	DRAMP01214	Kassinatuerin-2Ma (Frogs, amphibians, animals)	Antibacterial, Anti-Gram+, Anti-Gram-, Antimicrobial
53	125	DRAMP01218	Kassinatuerin-1 (Frogs, amphibians, animals)	Antibacterial, Antifungal, Anti-Gram+, Anti-Gram-, Antimicrobial

S.no.	PepID	DRAMP_ID	Name of the AMP	Activity
54	127	DRAMP01222	Palustrin-2AJ1 (PL2AJ1; Frogs, amphibians, animals)	Antibacterial, Anti-Gram+, Anti-Gram-, Antimicrobial
55	132	DRAMP01232	Palustrin-2ISc (Frogs, amphibians, animals)	Antibacterial, Anti-Gram+, Anti-Gram-, Antimicrobial
56	136	DRAMP01237	Palustrin-2ISa (Frogs, amphibians, animals)	Antibacterial, Antifungal, Anti-Gram+, Anti-Gram-, Antimicrobial
57	137	DRAMP01238	Palustrin-2SIb (Frogs, amphibians, animals)	Antibacterial, Anti-Gram+, Anti-Gram-, Antimicrobial
58	138	DRAMP01244	Japonicin-1 (Frogs, amphibians, animals)	Antibacterial, Anti-Gram+, Anti-Gram-, Antimicrobial
59	139	DRAMP01245	Japonicin-1CDYa (Frogs, amphibians, animals)	Antibacterial, Anti-Gram+, Anti-Gram-, Antimicrobial
60	140	DRAMP01246	Japonicin-2 (Frogs, amphibians, animals)	Antibacterial, Anti-Gram+, Anti-Gram-, Antimicrobial
61	142	DRAMP01249	Dybowskin-2 (Frogs, amphibians, animals)	Antibacterial, Anti-Gram+, Anti-Gram-, Antimicrobial
62	143	DRAMP01250	Dybowskin-3 (Frogs, amphibians, animals)	Antibacterial, Anti-Gram+, Anti-Gram-, Antimicrobial
63	144	DRAMP01251	Dybowskin-4 (Frogs, amphibians, animals)	Antibacterial, Antifungal, Anti-Gram+, Anti-Gram-, Antimicrobial
64	145	DRAMP01252	Dybowskin-5 (Frogs, amphibians, animals)	Antibacterial, Antifungal, Anti-Gram+, Anti-Gram-, Antimicrobial
65	147	DRAMP01254	Dybowskin-1CDYa (Frogs, amphibians, animals)	Antibacterial, Anti-Gram+, Anti-Gram-, Antimicrobial
66	148	DRAMP01255	Dybowskin-2CDYa (Chensinin-1; Frogs, amphibians, animals)	Antibacterial, Anti-Gram+, Anti-Gram-, Antimicrobial
67	149	DRAMP01257	Dermadistinctin-K (DD K; Frogs, amphibians, animals)	Antibacterial, Antifungal, Antiprotozoal, Anti-Gram+, Anti-Gram-, Antimicrobial
68	150	DRAMP01258	Dermadistinctin-L (DD L; Frogs, amphibians, animals)	Antibacterial, Antifungal, Antiprotozoal, Anti-Gram+, Anti-Gram-, Antimicrobial
69	151	DRAMP01259	Dermadistinctin-M (DD M; Frogs, amphibians, animals)	Antibacterial, Anti-Gram+, Anti-Gram-, Antimicrobial
70	152	DRAMP01260	Dermadistinctin-Q1 (DD Q1; Frogs, amphibians, animals)	Antibacterial, Anti-Gram+, Anti-Gram-, Antimicrobial
71	153	DRAMP01261	Dermadistinctin-Q2 (DD Q2; Frogs, amphibians, animals)	Antibacterial, Anti-Gram+, Anti-Gram-, Antimicrobial
72	155	DRAMP01301	Phylloseptin-1 (PS-1; Frogs, amphibians, animals)	Antibacterial, Antifungal, Antiprotozoal, Anti-Gram+, Anti-Gram-, Antimicrobial
73	156	DRAMP01302	Phylloseptin-2 (PS-2; Frogs, amphibians, animals)	Antibacterial, Antifungal, Anti-Gram+, Anti-Gram-, Antimicrobial
74	157	DRAMP01303	Phylloseptin-3 (PS-3; Frogs, amphibians, animals)	Antibacterial, Antifungal, Anti-Gram+, Anti-Gram-, Antimicrobial
75	158	DRAMP01305	Phylloseptin-7 (PS-7; Frogs, amphibians, animals)	Antibacterial, Anti-Gram+, Anti-Gram-, Antimicrobial
76	159	DRAMP01306	Phylloseptin-7 (PS-7; Frogs, amphibians, animals)	Antibacterial, Anti-Gram+, Anti-Gram-, Antimicrobial
77	161	DRAMP01319	Cathelicidin-AL (Gly-rich; Frogs, amphibians, animals)	Antibacterial, Antifungal, Anti-Gram+, Anti-Gram-, Antimicrobial
78	163	DRAMP01339	Amolopin-2a (Frogs, amphibians, animals)	Antibacterial, Antifungal, Anti-Gram+, Anti-Gram-, Antimicrobial
79	164	DRAMP01341	Amolopin-1b (Frogs, amphibians, animals)	Antibacterial, Antifungal, Anti-Gram+, Anti-Gram-, Antimicrobial
80	165	DRAMP01346	Prepromelittin-related peptide (Frogs, amphibians, animals)	Antibacterial, Antifungal, Anti-Gram+, Anti-Gram-, Antimicrobial
81	166	DRAMP01347	Prepromelittin-related peptide (Frogs, amphibians, animals)	Antibacterial, Antifungal, Anti-Gram+, Anti-Gram-, Antimicrobial
82	167	DRAMP01350	Tigerinin-1 (Frogs, amphibians, animals)	Antibacterial, Antifungal, Anti-Gram+, Anti-Gram-, Antimicrobial
83	168	DRAMP01351	Tigerinin-2 (Frogs, amphibians, animals)	Antibacterial, Antifungal, Anti-Gram+, Anti-Gram-, Antimicrobial
84	169	DRAMP01352	Tigerinin-3 (Frogs, amphibians, animals)	Antibacterial, Antifungal, Anti-Gram+, Anti-Gram-, Antimicrobial
85	170	DRAMP01353	Tigerinin-4 (Frogs, amphibians, animals)	Antibacterial, Antifungal, Anti-Gram+, Anti-Gram-, Antimicrobial
86	172	DRAMP01355	Ranalexin (Frogs, amphibians, animals)	Antibacterial, Antifungal, Anti-Gram+, Anti-Gram-, Antimicrobial
87	173	DRAMP01393	Odorranain-W1 (OdW1; Frogs, amphibians, animals)	Antimicrobial, Antibacterial, Antifungal, Anti-Gram+, Anti-Gram-,
88	174	DRAMP01358	Ranalexin-Vb (Frogs, amphibians, animals)	Antibacterial, Anti-Gram+, Anti-Gram-, Antimicrobial
89	175	DRAMP01359	Ranalexin-1G (Frogs, amphibians, animals)	Antibacterial, Anti-Gram+, Anti-Gram-, Antimicrobial
90	178	DRAMP01362	Frenatin-3 (Frogs, amphibians, animals)	Antibacterial, Anti-Gram+, Anti-Gram-, Antimicrobial
91	179	DRAMP01364	Maculatin-1.1 (Frogs, amphibians, animals)	Antibacterial, Antifungal, Antiviral, Anti-Gram+, Anti-Gram-, Antimicrobial
92	182	DRAMP01370	Oh-defensin (O. hainana defensin; spiders, animals)	Antibacterial, Antifungal, Anti-Gram+, Anti-Gram-, Antimicrobial
93	183	DRAMP01371	Odorranain-NR (Frogs, amphibians, animals)	Antibacterial, Antifungal, Anti-Gram+, Anti-Gram-, Antimicrobial
94	184	DRAMP00931	Antimicrobial peptide 3 (Cn-AMP3; Plant defensin)	Antibacterial, Anti-Gram+, Anti-Gram-, Antimicrobial
95	185	DRAMP00930	Antimicrobial peptide 2 (Cn-AMP2; Plant defensin)	Antibacterial, Anti-Gram+, Anti-Gram-, Antimicrobial
96	186	DRAMP00929	Antimicrobial peptide 1 (Cn-AMP1; Plant defensin)	Antibacterial, Anti-Gram+, Anti-Gram-, Antimicrobial
97	187	DRAMP03542	Neurokinin A (NKA; chicken, animals)	Neuropeptide,Antibacterial, Anti-Gram+, Anti-Gram-, Antimicrobial
98	188	DRAMP04532	Myxinidin (Hagfish, animals)	Antimicrobial, Antibacterial, Antifungal, Anti-Gram+, Anti-Gram-,
99	190	DRAMP02993	Abaecin (Pro-rich; insects, arthropods, invertebrates, animals)	Antibacterial, Anti-Gram+, Anti-Gram-, Antimicrobial
100	191	DRAMP02997	Apidaecin-1B (Apidaecin IB; Insects, animals)	Antibacterial, Anti-Gram+, Anti-Gram-, Antimicrobial
101	193	DRAMP02840	Lactoferricin B (Lfcin B; mammals, animals)	Antibacterial, Anti-Gram+, Anti-Gram-, Antimicrobial
102	198	DRAMP02246	Ranatuerin-1C (Ranatuerin 1C; Frogs, amphibians, animals)	Antifungal, Anti-Gram+, Anti-Gram-,
103	199	DRAMP01394	Odorranain-W2 (Frogs, amphibians, animals)	Antibacterial, Antifungal, Anti-Gram+, Anti-Gram-, Antimicrobial
104	200	DRAMP01395	Odorranain-A-OA1 (Frogs, amphibians, animals)	Antibacterial, Antifungal, Anti-Gram+, Anti-Gram-, Antimicrobial
105	201	DRAMP01396	Odorranain-F-OA1 (Frogs, amphibians, animals)	Antibacterial, Antifungal , Anti-Gram+, Anti-Gram-, Antimicrobial
106	202	DRAMP01397	Odorranain-F-OA2 (Frogs, amphibians, animals)	Antibacterial, Antifungal , Anti-Gram+, Anti-Gram-, Antimicrobial

S.no.	PepID	DRAMP_ID	Name of the AMP	Activity
107	203	DRAMP01398	Odorranain-F-OA3 (Frogs, amphibians, animals)	Antibacterial, Antifungal , Anti-Gram+, Anti-Gram-, Antimicrobial
108	204	DRAMP01399	Odorranain-F-OA4 (Frogs, amphibians, animals)	Antibacterial, Antifungal , Anti-Gram+, Anti-Gram-, Antimicrobial
109	206	DRAMP01401	Odorranain-F-OW1 (Frogs, amphibians, animals)	Antibacterial, Antifungal , Anti-Gram+, Anti-Gram-, Antimicrobial
110	207	DRAMP01402	Odorranain-J-OA1 (Frogs, amphibians, animals)	Antibacterial, Antifungal , Anti-Gram+, Anti-Gram-, Antimicrobial
111	208	DRAMP01403	Odorranain-J-OA2 (Frogs, amphibians, animals)	Antibacterial, Antifungal , Anti-Gram+, Anti-Gram-, Antimicrobial
112	209	DRAMP01409	Nigrocin-OR1 (Frogs, amphibians, animals)	Antibacterial, Antifungal, Anti-Gram+, Anti-Gram-, Antimicrobial
113	210	DRAMP01410	Nigrocin-OR2 (Frogs, amphibians, animals)	Antibacterial, Antifungal, Anti-Gram+, Anti-Gram-, Antimicrobial
114	211	DRAMP01411	Nigrocin-OR3 (Frogs, amphibians, animals)	Antibacterial, Antifungal, Anti-Gram+, Anti-Gram-, Antimicrobial
115	212	DRAMP01412	Nigrocin-2HSa (Frogs, amphibians, animals)	Antibacterial, Anti-Gram+, Anti-Gram-, Antimicrobial
116	213	DRAMP01413	Nigrocin-2HSb (Frogs, amphibians, animals)	Antibacterial, Anti-Gram+, Anti-Gram-, Antimicrobial
117	214	DRAMP01414	Nigrocin-2ISa (Frogs, amphibians, animals)	Antibacterial, Antifungal, Anti-Gram+, Anti-Gram-, Antimicrobial
118	215	DRAMP01415	Nigrocin-2ISb (Frogs, amphibians, animals)	Antibacterial, Antifungal, Anti-Gram+, Anti-Gram-, Antimicrobial
119	216	DRAMP01416	Nigrocin-2ISc (Frogs, amphibians, animals)	Antibacterial, Antifungal, Anti-Gram+, Anti-Gram-, Antimicrobial
120	218	DRAMP01418	Nigrocin-2GRb (Frogs, amphibians, animals)	Antibacterial, Antifungal, Anti-Gram+, Anti-Gram-, Antimicrobial
121	220	DRAMP01420	Nigrocin-OG4 (Frogs, amphibians, animals)	Antibacterial, Antifungal , Anti-Gram+, Anti-Gram-, Antimicrobial
122	221	DRAMP01421	Nigrocin-OG5 (Frogs, amphibians, animals)	Antibacterial, Antifungal , Anti-Gram+, Anti-Gram-, Antimicrobial
123	222	DRAMP01422	Nigrosin-OG21 (Frogs, amphibians, animals)	Antibacterial, Antifungal , Anti-Gram+, Anti-Gram-, Antimicrobial
124	223	DRAMP01423	Nigrosin-OG13 (Frogs, amphibians, animals)	Antibacterial, Antifungal , Anti-Gram+, Anti-Gram-, Antimicrobial
125	224	DRAMP01426	Nigrocin-1-OA1 (Frogs, amphibians, animals)	Antibacterial, Antifungal, Anti-Gram+, Anti-Gram-, Antimicrobial
126	225	DRAMP01427	Nigrocin-1-OA2 (Frogs, amphibians, animals)	Antibacterial, Antifungal, Anti-Gram+, Anti-Gram-, Antimicrobial
127	226	DRAMP01428	Nigrocin-1-OA3 (Frogs, amphibians, animals)	Antibacterial, Antifungal, Anti-Gram+, Anti-Gram-, Antimicrobial
128	227	DRAMP01429	Nigrocin-1-OR1 (Frogs, amphibians, animals)	Antibacterial, Antifungal, Anti-Gram+, Anti-Gram-, Antimicrobial
129	228	DRAMP01430	Nigrocin-1-OR2 (Frogs, amphibians, animals)	Antibacterial, Antifungal, Anti-Gram+, Anti-Gram-, Antimicrobial
130	229	DRAMP01431	Nigrocin-1-OR3 (Frogs, amphibians, animals)	Antibacterial, Antifungal, Anti-Gram+, Anti-Gram-, Antimicrobial
131	230	DRAMP01432	Nigrocin-1-OW2 (Frogs, amphibians, animals)	Antibacterial, Antifungal, Anti-Gram+, Anti-Gram-, Antimicrobial
132	231	DRAMP01433	Nigrocin-1-OW3 (Frogs, amphibians, animals)	Antibacterial, Antifungal, Anti-Gram+, Anti-Gram-, Antimicrobial
133	232	DRAMP01434	Nigrocin-1-OW4 (Frogs, amphibians, animals)	Antibacterial, Antifungal, Anti-Gram+, Anti-Gram-, Antimicrobial
134	233	DRAMP01435	Nigrocin-1-OW5 (Frogs, amphibians, animals)	Antibacterial, Antifungal, Anti-Gram+, Anti-Gram-, Antimicrobial
135	234	DRAMP01436	Nigrocin-1-OW1 (Frogs, amphibians, animals)	Antibacterial, Antifungal, Anti-Gram+, Anti-Gram-, Antimicrobial
136	236	DRAMP01438	Nigrocin-2JDa (Frogs, amphibians, animals)	Antibacterial, Antifungal , Anti-Gram+, Anti-Gram-, Antimicrobial
137	237	DRAMP01439	Nigrocin-2JDb (Odorranain-H2; Frogs, amphibians, animals)	Antibacterial, Antifungal , Anti-Gram+, Anti-Gram-, Antimicrobial
138	238	DRAMP01440	Nigrocin-2LVb (Frogs, amphibians, animals)	Antibacterial, Anti-Gram+, Anti-Gram-, Antimicrobial
139	242	DRAMP01447	Esculentin-2CHa (Frogs, amphibians, animals)	Antibacterial, Antifungal, Anti-Gram+, Anti-Gram-, Antimicrobial
140	243	DRAMP01452	Esculentin-1LTa (Frogs, amphibians, animals)	Antibacterial, Antifungal, Anti-Gram+, Anti-Gram-, Antimicrobial
141	244	DRAMP01453	Esculentin-2LTa (Frogs, amphibians, animals)	Antibacterial, Anti-Gram+, Anti-Gram-, Antimicrobial
142	245	DRAMP01454	Esculentin-2JDa (Frogs, amphibians, animals)	Antibacterial, Anti-Gram+, Anti-Gram-, Antimicrobial
143	246	DRAMP01456	Esculentin-2PLa (Frogs, amphibians, animals)	Antibacterial, Antifungal, Anti-Gram+, Anti-Gram-, Antimicrobial
144	247	DRAMP01457	Esculentin-1V (Frogs, amphibians, animals)	Antibacterial, Anti-Gram+, Anti-Gram-, Antimicrobial
145	248	DRAMP01458	Esculentin-2V (Frogs, amphibians, animals)	Antibacterial, Anti-Gram+, Anti-Gram-, Antimicrobial
146	249	DRAMP01461	Esculentin-1S (Frogs, amphibians, animals)	Antibacterial, Anti-Gram+, Anti-Gram-, Antimicrobial
147	250	DRAMP01462	Esculentin-2S (Frogs, amphibians, animals)	Antibacterial , Anti-Gram+, Anti-Gram-, Antimicrobial
148	251	DRAMP01469	Esculentin-2-Ala (Frogs, amphibians, animals)	Antibacterial, Anti-Gram+, Anti-Gram-, Antimicrobial
149	252	DRAMP01470	Esculentin-2-ALb (Frogs, amphibians, animals)	Antibacterial, Anti-Gram+, Anti-Gram-, Antimicrobial
150	253	DRAMP01471	Esculentin-1PLa (Frogs, amphibians, animals)	Antibacterial, Anti-Gram+, Anti-Gram-, Antimicrobial
151	254	DRAMP01472	Esculentin-1PLb (Frogs, amphibians, animals)	Antibacterial, Anti-Gram+, Anti-Gram-, Antimicrobial
152	256	DRAMP01474	Esculentin-1ARa (Frogs, amphibians, animals)	Antibacterial, Anti-Gram+, Anti-Gram-, Antimicrobial
153	257	DRAMP01475	Esculentin-1ARb (Frogs, amphibians, animals)	Antibacterial, Anti-Gram+, Anti-Gram-, Antimicrobial
154	258	DRAMP01476	Esculentin-2HSa (Frogs, amphibians, animals)	Antibacterial, Anti-Gram+, Anti-Gram-, Antimicrobial
155	259	DRAMP01477	Esculentin-1HSa (Frogs, amphibians, animals)	Antibacterial, Anti-Gram+, Anti-Gram-, Antimicrobial
156	260	DRAMP01479	Esculentin-1CPa (Frogs, amphibians, animals)	Antibacterial, Antifungal, Anti-Gram+, Anti-Gram-, Antimicrobial
157	261	DRAMP01480	Esculentin-2CPa (Frogs, amphibians, animals)	Antibacterial, Antifungal, Anti-Gram+, Anti-Gram-, Antimicrobial
158	262	DRAMP01482	Esculentin-1ISa (Frogs, amphibians, animals)	Antibacterial, Anti-Gram+, Anti-Gram-, Antimicrobial
159	263	DRAMP01483	Esculentin-1ISb (Frogs, amphibians, animals)	Antibacterial, Antifungal, Anti-Gram+, Anti-Gram-, Antimicrobial

S.no.	PepID	DRAMP_ID	Name of the AMP	Activity
160	264	DRAMP01484	Esculentin-2ISa (Frogs, amphibians, animals)	Antibacterial, Antifungal, Anti-Gram+, Anti-Gram-, Antimicrobial
161	265	DRAMP01486	Esculentin-1GRa (Frogs, amphibians, animals)	Antibacterial, Antifungal, Anti-Gram+, Anti-Gram-, Antimicrobial
162	266	DRAMP01490	Esculentin-2A (Frogs, amphibians, animals)	Antibacterial, Anti-Gram+, Anti-Gram-, Antimicrobial
163	267	DRAMP01491	Esculentin-1B (Frogs, amphibians, animals)	Antibacterial, Anti-Gram+, Anti-Gram-, Antimicrobial
164	268	DRAMP01493	Esculentin-1-OA1 (Frogs, amphibians, animals)	Antibacterial, Antifungal, Anti-Gram+, Anti-Gram-, Antimicrobial
165	269	DRAMP01494	Esculentin-1-OA2 (Frogs, amphibians, animals)	Antibacterial, Antifungal, Anti-Gram+, Anti-Gram-, Antimicrobial
166	270	DRAMP01495	Esculentin-1-OA3 (Frogs, amphibians, animals)	Antibacterial, Antifungal, Anti-Gram+, Anti-Gram-, Antimicrobial
167	271	DRAMP01496	Esculentin-1-OA4 (Frogs, amphibians, animals)	Antibacterial, Antifungal, Anti-Gram+, Anti-Gram-, Antimicrobial
168	272	DRAMP01497	Esculentin-1-OA5 (Frogs, amphibians, animals)	Antibacterial, Antifungal, Anti-Gram+, Anti-Gram-, Antimicrobial
169	273	DRAMP01499	Esculentin-1-OR1 (Frogs, amphibians, animals)	Antibacterial, Antifungal, Anti-Gram+, Anti-Gram-, Antimicrobial
170	274	DRAMP01501	Esculentin-1-OR3 (Frogs, amphibians, animals)	Antibacterial, Antifungal, Anti-Gram+, Anti-Gram-, Antimicrobial
171	275	DRAMP01502	Esculentin-1-OR4 (Frogs, amphibians, animals)	Antibacterial, Antifungal, Anti-Gram+, Anti-Gram-, Antimicrobial
172	276	DRAMP01503	Esculentin-1-OR5 (Frogs, amphibians, animals)	Antibacterial, Antifungal, Anti-Gram+, Anti-Gram-, Antimicrobial
173	277	DRAMP01504	Esculentin-2-OA1 (Frogs, amphibians, animals)	Antibacterial, Antifungal, Anti-Gram+, Anti-Gram-, Antimicrobial
174	278	DRAMP01505	Esculentin-2-OA2 (Frogs, amphibians, animals)	Antibacterial, Antifungal, Anti-Gram+, Anti-Gram-, Antimicrobial
175	280	DRAMP01507	Esculentin-2-OR1 (Frogs, amphibians, animals)	Antibacterial, Antifungal, Anti-Gram+, Anti-Gram-, Antimicrobial
176	281	DRAMP01508	Esculentin-2-OR2 (Frogs, amphibians, animals)	Antibacterial, Antifungal, Anti-Gram+, Anti-Gram-, Antimicrobial
177	282	DRAMP01509	Esculentin-2-OR3 (Frogs, amphibians, animals)	Antibacterial, Antifungal, Anti-Gram+, Anti-Gram-, Antimicrobial
178	283	DRAMP01510	Esculentin-2-OR4 (Frogs, amphibians, animals)	Antibacterial, Antifungal, Anti-Gram+, Anti-Gram-, Antimicrobial
179	284	DRAMP01511	Esculentin-2-OR5 (Frogs, amphibians, animals)	Antibacterial, Antifungal, Anti-Gram+, Anti-Gram-, Antimicrobial
180	285	DRAMP01513	Esculentin-1 (Frogs, amphibians, animals)	Antibacterial, Antifungal, Anti-Gram+, Anti-Gram-, Antimicrobial
181	286	DRAMP01520	Rugosin-A (Frogs, amphibians, animals)	Antibacterial, Anti-Gram+, Anti-Gram-, Antimicrobial
182	287	DRAMP01521	Rugosin-B (Frogs, amphibians, animals)	Antibacterial, Anti-Gram+, Anti-Gram-, Antimicrobial
183	288	DRAMP01524	Rugosin-RN1 (Frogs, amphibians, animals)	Antibacterial, Antifungal, Anti-Gram+, Anti-Gram-, Antimicrobial
184	289	DRAMP01525	Rugosin-RN3 (Frogs, amphibians, animals)	Antibacterial, Antifungal, Anti-Gram+, Anti-Gram-, Antimicrobial
185	290	DRAMP01526	Rugosin-RN5 (Frogs, amphibians, animals)	Antibacterial, Antifungal, Anti-Gram+, Anti-Gram-, Antimicrobial
186	291	DRAMP01533	Nigroain-B1 (Frogs, amphibians, animals)	Antibacterial, Anti-Gram+, Anti-Gram-, Antimicrobial
187	292	DRAMP01539	Nigroain-C2 (Frogs, amphibians, animals)	Antibacterial, Antifungal, Anti-Gram+, Anti-Gram-, Antimicrobial
188	295	DRAMP01546	Nigroain-K1 (Frogs, amphibians, animals)	Antibacterial, Antifungal, Anti-Gram+, Anti-Gram-, Antimicrobial
189	297	DRAMP01549	Caerin-1.1 (Frogs, amphibians, animals)	Antibacterial, Antiviral, Anti-Gram+, Anti-Gram-, Antimicrobial
190	298	DRAMP01550	Caerin-1.11 (Frogs, amphibians, animals)	Antibacterial, Anti-Gram+, Anti-Gram-, Antimicrobial
191	299	DRAMP01552	Caerin-1.3 (Frogs, amphibians, animals)	Antibacterial, Anti-Gram+, Anti-Gram-, Antimicrobial
192	300	DRAMP01553	Caerin-1.4 (Frogs, amphibians, animals)	Antibacterial, Anti-Gram+, Anti-Gram-, Antimicrobial
193	301	DRAMP01555	Caerin-1.5 (Frogs, amphibians, animals)	Antibacterial, Anti-Gram+, Anti-Gram-, Antimicrobial
194	302	DRAMP01560	Caerin-1.9 (Frogs, amphibians, animals)	Antibacterial, Antifungal, Antiviral, Anti-Gram+, Anti-Gram-, Antimicrobial
195	304	DRAMP01563	Caerin-2.2 (Frogs, amphibians, animals)	Antibacterial , Anti-Gram+, Anti-Gram-, Antimicrobial
196	309	DRAMP01574	Caerin-4.1 (Frogs, amphibians, animals)	Antibacterial, Antiviral, Anti-Gram+, Anti-Gram-, Antimicrobial
197	310	DRAMP01576	Caerin-4.3 (Frogs, amphibians, animals)	Antibacterial, Anti-Gram+, Anti-Gram-, Antimicrobial
198	311	DRAMP01577	Caerin-1.10 (Frogs, amphibians, animals)	Antibacterial, Anti-Gram+, Anti-Gram-, Antimicrobial
199	316	DRAMP01585	Caerin-1.18 (Frogs, amphibians, animals)	Antibacterial, Anti-Gram+, Anti-Gram-, Antimicrobial
200	317	DRAMP01586	Caerin-1.19 (Frogs, amphibians, animals)	Antibacterial, Anti-Gram+, Anti-Gram-, Antimicrobial
201	321	DRAMP01590	Citropin 1.1 M14 (Frogs, amphibians, animals)	Antibacterial, Anti-Gram+, Anti-Gram-, Antimicrobial
202	322	DRAMP01591	Citropin 1.1 M15 (Frogs, amphibians, animals)	Antibacterial, Anti-Gram+, Anti-Gram-, Antimicrobial
203	329	DRAMP01607	Aurein-1.2 (Frogs, amphibians, animals)	Antibacterial, Anticancer, Anti-Gram+, Anti-Gram-, Antimicrobial
204	335	DRAMP01618	Aurein-3.3 (Frogs, amphibians, animals)	Antibacterial, Anticancer, Anti-Gram+, Anti-Gram-, Antimicrobial
205	337	DRAMP01621	Bombinin-H1 (Frogs, amphibians, animals)	Antibacterial, Anti-Gram+, Anti-Gram-, Antimicrobial
206	338	DRAMP01623	Bombinin-H4 (bombinin H isomers; Frogs, amphibians, animals)	Antibacterial, Anti-Gram+, Anti-Gram-, Antimicrobial
207	339	DRAMP01626	Bombinin-H5 (Frogs, amphibians, animals)	Antibacterial, Anti-Gram+, Anti-Gram-, Antimicrobial
208	340	DRAMP01627	Skin peptide tyrosine-tyrosine (Skin-PYY; SPYY; Frogs, amphibians,	Antibacterial, Antifungal, Anti-Gram+, Anti-Gram-, Antimicrobial
209	341	DRAMP01628	Phylloxin (Frogs, amphibians, animals)	Antibacterial, Anti-Gram+, Anti-Gram-, Antimicrobial
210	343	DRAMP01639	Dermaseptin-1 (DShypo01, DPh-1; Frogs, amphibians, animals)	Antibacterial, Antiprotozoal, Anti-Gram+, Anti-Gram-, Antimicrobial
211	344	DRAMP01643	Dermaseptin-5 (DShypo05, DS 01; Frogs, amphibians, animals)	Antibacterial, Antiprotozoal, Anti-Gram+, Anti-Gram-, Antimicrobial
212	345	DRAMP01646	Adenoregulin (Dermaseptin BII; Dermaseptin B2; Frogs, amphibians,	Antibacterial, Antifungal, Anti-Gram+, Anti-Gram-, Antimicrobial

S.no.	PepID	DRAMP_ID	Name of the AMP	Activity
213	347	DRAMP01648	Dermaseptin-like PBN2 (DRP-PBN2; Plasticin-B1a; Frogs,	Antibacterial, Antifungal, Anti-Gram+, Anti-Gram-, Antimicrobial
214	348	DRAMP01649	Dermaseptin-BI (Dermaseptin B1; Frogs, amphibians, animals)	Antibacterial, Antifungal, Anti-Gram+, Anti-Gram-, Antimicrobial
215	349	DRAMP01650	Dermaseptin-B3 (Dermaseptin BIII; Frogs, amphibians, animals)	Antibacterial, Anti-Gram+, Anti-Gram-, Antimicrobial
216	350	DRAMP01651	Dermaseptin-B4 (Dermaseptin BIV; Frogs, amphibians, animals)	Antibacterial, Anti-Gram+, Anti-Gram-, Antimicrobial
217	352	DRAMP01668	Dermaseptin-1 (DS I; Dermaseptin-S1, DS1; Frogs, amphibians,	Antibacterial, Antifungal, Antiprotozoal, Anti-Gram+, Anti-Gram-, Antimicrobial
218	357	DRAMP01702	Dermaseptin-H5 (Dermaseptin-like peptide 5, DMS5; Frogs,	Antibacterial , Anti-Gram+, Anti-Gram-, Antimicrobial
219	359	DRAMP01730	Temporin-A (Frogs, amphibians, animals)	Antibacterial, Antifungal, Anti-Gram+, Anti-Gram-, Antimicrobial
220	360	DRAMP01731	Temporin-ALd (Frogs, amphibians, animals)	Antibacterial, Anti-Gram+, Anti-Gram-, Antimicrobial
221	361	DRAMP01732	Temporin-ALe (Frogs, amphibians, animals)	Antibacterial, Anti-Gram+, Anti-Gram-, Antimicrobial
222	362	DRAMP01733	Temporin-ALf (Frogs, amphibians, animals)	Antibacterial, Anti-Gram+, Anti-Gram-, Antimicrobial
223	363	DRAMP01734	Temporin-ALg (Frogs, amphibians, animals)	Antibacterial, Anti-Gram+, Anti-Gram-, Antimicrobial
224	364	DRAMP01735	Temporin-ALh (Frogs, amphibians, animals)	Antibacterial, Anti-Gram+, Anti-Gram-, Antimicrobial
225	365	DRAMP01736	Temporin-ALi (Frogs, amphibians, animals)	Antibacterial, Anti-Gram+, Anti-Gram-, Antimicrobial
226	366	DRAMP01737	Temporin-ALj (Frogs, amphibians, animals)	Antibacterial, Anti-Gram+, Anti-Gram-, Antimicrobial
227	367	DRAMP01738	Temporin-ALk (Frogs, amphibians, animals)	Antibacterial, Anti-Gram+, Anti-Gram-, Antimicrobial
228	368	DRAMP01739	Temporin-B (Frogs, amphibians, animals)	Antibacterial, Antifungal, Anti-Gram+, Anti-Gram-, Antimicrobial
229	372	DRAMP01753	Temporin-1CEa (Frogs, amphibians, animals)	Antibacterial, Anti-Gram+, Anti-Gram-, Antimicrobial
230	373	DRAMP01754	Temporin-1CEb (Frogs, amphibians, animals)	Antibacterial, Anti-Gram+, Anti-Gram-, Antimicrobial
231	374	DRAMP01755	Temporin-1TSa (Frogs, amphibians, animals)	Antibacterial, Anti-Gram+, Anti-Gram-, Antimicrobial
232	376	DRAMP01764	Temporin-1TGa (Frogs, amphibians, animals)	Antibacterial, Antifungal, Anti-Gram+, Anti-Gram-, Antimicrobial
233	377	DRAMP01765	Temporin-1TGb (Frogs, amphibians, animals)	Antibacterial, Anti-Gram+, Anti-Gram-, Antimicrobial
234	378	DRAMP01766	Temporin-1TGc (Frogs, amphibians, animals)	Antibacterial, Anti-Gram+, Anti-Gram-, Antimicrobial
235	381	DRAMP01771	Temporin-1Oa (Frogs, amphibians, animals)	Antibacterial, Anti-Gram+, Anti-Gram-, Antimicrobial
236	383	DRAMP01775	Temporin-1Sa (Frogs, amphibians, animals)	Antibacterial, Anti-Gram+, Anti-Gram-, Antimicrobial
237	384	DRAMP01776	Temporin-1Sb (Temporin-SHb; Frogs, amphibians, animals)	Antibacterial, Antifungal, Anti-Gram+, Anti-Gram-, Antimicrobial
238	385	DRAMP01777	Temporin-1Sc (Temporin-SHc; Frogs, amphibians, animals)	Antibacterial, Antifungal, Anti-Gram+, Anti-Gram-, Antimicrobial
239	386	DRAMP01779	Temporin-SHf (Frogs, amphibians, animals)	Antibacterial, Anti-Gram+, Anti-Gram-, Antimicrobial
240	387	DRAMP01780	Temporin-SHa (Temporin-1Sa; Frogs, amphibians, animals)	Antibacterial, Antifungal, Anti-Gram+, Anti-Gram-, Antimicrobial
241	390	DRAMP01784	Temporin-LTc (Frogs, amphibians, animals)	Antibacterial, Antiviral, Anti-Gram+, Anti-Gram-, Antimicrobial
242	391	DRAMP01785	Temporin-CPa (Frogs, amphibians, animals)	Antibacterial, Anti-Gram+, Anti-Gram-, Antimicrobial
243	392	DRAMP01787	Temporin-HN1 (Frogs, amphibians, animals)	Antibacterial, Antifungal, Anti-Gram+, Anti-Gram-, Antimicrobial
244	393	DRAMP01788	Temporin-HN2 (Frogs, amphibians, animals)	Antibacterial, Antifungal, Anti-Gram+, Anti-Gram-, Antimicrobial
245	394	DRAMP01789	Temporin-1Va (Temporin 1Va; Frogs, amphibians, animals)	Antibacterial, Antifungal, Anti-Gram+, Anti-Gram-, Antimicrobial
246	396	DRAMP01791	Temporin-1Vc (Temporin 1Vc; Frogs, amphibians, animals)	Antibacterial, Anti-Gram+, Anti-Gram-, Antimicrobial
247	397	DRAMP01807	Temporin-RN1 (Frogs, amphibians, animals)	Antibacterial, Antifungal, Anti-Gram+, Anti-Gram-, Antimicrobial
248	398	DRAMP01808	Temporin-RN3 (Frogs, amphibians, animals)	Antibacterial, Antifungal, Anti-Gram+, Anti-Gram-, Antimicrobial
249	399	DRAMP01811	Temporin-Ra (Frogs, amphibians, animals)	Antibacterial, Anti-Gram+, Anti-Gram-, Antimicrobial
250	400	DRAMP01812	Temporin-Rb (Frogs, amphibians, animals)	Antibacterial, Anti-Gram+, Anti-Gram-, Antimicrobial
251	402	DRAMP01816	Temporin-1CSb (Frogs, amphibians, animals)	Antibacterial, Anti-Gram+, Anti-Gram-, Antimicrobial
252	403	DRAMP01817	Temporin-1CSc (Frogs, amphibians, animals)	Antibacterial, Anti-Gram+, Anti-Gram-, Antimicrobial
253	404	DRAMP01818	Temporin-1CSd (Temporin-1DRb; Frogs, amphibians, animals)	Antibacterial, Antifungal, Anti-Gram+, Anti-Gram-, Antimicrobial
254	405	DRAMP01392	Odorranain-V1 (OdV1; Frogs, amphibians, animals)	Antimicrobial, Antibacterial, Antifungal, Anti-Gram+, Anti-Gram-,
255	406	DRAMP01391	Odorranain-U1 (OdU1; Frogs, amphibians, animals)	Antimicrobial, Antibacterial, Antifungal, Anti-Gram+, Anti- Gram-,
256	407	DRAMP01832	Temporin-Eca (Frogs, amphibians, animals)	Antibacterial, Anti-Gram+, Anti-Gram-, Antimicrobial
257	408	DRAMP01833	Buforin-EC (Frogs, amphibians, animals)	Antibacterial, Anti-Gram+, Anti-Gram-, Antimicrobial
258	409	DRAMP01834	Cyanophlyctin (Frogs, amphibians, animals)	Antibacterial, Anti-Gram+, Anti-Gram-, Antimicrobial
259	410	DRAMP01840	Ascaphin-1 (Frogs, amphibians, animals)	Antibacterial, Anti-Gram+, Anti-Gram-, Antimicrobial
260	411	DRAMP01842	Ascaphin-3 (Frogs, amphibians, animals)	Antibacterial, Anti-Gram+, Anti-Gram-, Antimicrobial
261	412	DRAMP01844	Ascaphin-5 (Frogs, amphibians, animals)	Antibacterial, Antifungal, Anti-Gram+, Anti-Gram-, Antimicrobial
262	413	DRAMP01846	Ascaphin-7 (Frogs, amphibians, animals)	Antibacterial, Anti-Gram+, Anti-Gram-, Antimicrobial
263	414	DRAMP01847	Ascaphin-8 (Frogs, amphibians, animals)	Antibacterial, Antiviral, Anti-Gram+, Anti-Gram-, Antimicrobial
264	415	DRAMP01849	Jindongenin-1a (JD1a; Frogs, amphibians, animals)	Antibacterial, Antifungal, Anti-Gram+, Anti-Gram-, Antimicrobial
265	416	DRAMP01869	Brevinin-1SPa (Frogs, amphibians, animals)	Antibacterial, Antifungal, Anti-Gram+, Anti-Gram-, Antimicrobial

S.no.	PepID	DRAMP_ID	Name of the AMP	Activity
266	417	DRAMP01870	Brevinin-1SPb (Frogs, amphibians, animals)	Antibacterial, Antifungal, Anti-Gram+, Anti-Gram-, Antimicrobial
267	418	DRAMP01872	Brevinin-1SPd (Frogs, amphibians, animals)	Antibacterial, Antifungal, Anti-Gram+, Anti-Gram-, Antimicrobial
268	419	DRAMP01873	Brevinin-2-related peptide (Frogs, amphibians, animals)	Antibacterial, Antifungal, Anti-Gram+, Anti-Gram-, Antimicrobial
269	420	DRAMP01875	Brevinin-2PRa (Frogs, amphibians, animals)	Antibacterial, Anti-Gram+, Anti-Gram-, Antimicrobial
270	421	DRAMP01876	Brevinin-2PRb (Frogs, amphibians, animals)	Antibacterial, Anti-Gram+, Anti-Gram-, Antimicrobial
271	422	DRAMP01877	Brevinin-2PRd (Frogs, amphibians, animals)	Antibacterial, Anti-Gram+, Anti-Gram-, Antimicrobial
272	423	DRAMP01878	Brevinin-2PRe (Frogs, amphibians, animals)	Antibacterial, Anti-Gram+, Anti-Gram-, Antimicrobial
273	424	DRAMP01879	Brevinin-2LTa (Frogs, amphibians, animals)	Antibacterial, Anti-Gram+, Anti-Gram-, Antimicrobial
274	425	DRAMP01880	Brevinin-2LTb (Frogs, amphibians, animals)	Antibacterial, Anti-Gram+, Anti-Gram-, Antimicrobial
275	426	DRAMP01881	Brevinin-2LTc (Frogs, amphibians, animals)	Antibacterial, Anti-Gram+, Anti-Gram-, Antimicrobial
276	427	DRAMP01885	Brevinin-1TEa (Frogs, amphibians, animals)	Antibacterial, Anti-Gram+, Anti-Gram-, Antimicrobial
277	428	DRAMP01886	Brevinin-2TEa (Frogs, amphibians, animals)	Antibacterial, Anti-Gram+, Anti-Gram-, Antimicrobial
278	429	DRAMP01887	Brevinin-2TEb (Frogs, amphibians, animals)	Antibacterial, Anti-Gram+, Anti-Gram-, Antimicrobial
279	430	DRAMP01888	Brevinin-1CHc (Frogs, amphibians, animals)	Antibacterial, Antifungal, Anti-Gram+, Anti-Gram-, Antimicrobial
280	431	DRAMP01889	Brevinin-1TOa (Frogs, amphibians, animals)	Antibacterial, Antifungal, Anti-Gram+, Anti-Gram-, Antimicrobial
281	432	DRAMP01890	Brevinin-1VLa (Frogs, amphibians, animals)	Antibacterial, Antifungal, Anti-Gram+, Anti-Gram-, Antimicrobial
282	433	DRAMP01891	Brevinin-1VLc (Frogs, amphibians, animals)	Antibacterial, Antifungal, Anti-Gram+, Anti-Gram-, Antimicrobial
283	434	DRAMP01892	Brevinin-1VLd (Frogs, amphibians, animals)	Antibacterial, Antifungal, Anti-Gram+, Anti-Gram-, Antimicrobial
284	435	DRAMP01893	Brevinin-1VLe (Frogs, amphibians, animals)	Antibacterial, Antifungal, Anti-Gram+, Anti-Gram-, Antimicrobial
285	436	DRAMP01896	Brevinin-1CG1 (Frogs, amphibians, animals)	Antibacterial, Antifungal, Anti-Gram+, Anti-Gram-, Antimicrobial
286	437	DRAMP01897	Brevinin-1CG2 (Frogs, amphibians, animals)	Antibacterial, Antifungal, Anti-Gram+, Anti-Gram-, Antimicrobial
287	438	DRAMP01898	Brevinin-1CG3 (Frogs, amphibians, animals)	Antibacterial, Antifungal, Anti-Gram+, Anti-Gram-, Antimicrobial
288	439	DRAMP01899	Brevinin-1CG4 (Frogs, amphibians, animals)	Antibacterial, Antifungal, Anti-Gram+, Anti-Gram-, Antimicrobial
289	440	DRAMP01900	Brevinin-1CG5 (Frogs, amphibians, animals)	Antibacterial, Antifungal, Anti-Gram+, Anti-Gram-, Antimicrobial
290	442	DRAMP01910	Brevinin-2GHb (AMP-2; Frogs, amphibians, animals)	Antibacterial, Anti-Gram+, Anti-Gram-, Antimicrobial
291	443	DRAMP01911	Brevinin-2GHc (AMP-4; Frogs, amphibians, animals)	Antibacterial, Anti-Gram+, Anti-Gram-, Antimicrobial
292	444	DRAMP01913	Brevinin-1GRa (Frogs, amphibians, animals)	Antibacterial, Anti-Gram+, Anti-Gram-, Antimicrobial
293	445	DRAMP01914	Brevinin-2GRa (Frogs, amphibians, animals)	Antibacterial, Antifungal, Anti-Gram+, Anti-Gram-, Antimicrobial
294	446	DRAMP01918	Brevinin-1PLb (Frogs, amphibians, animals)	Antibacterial, Antifungal, Anti-Gram+, Anti-Gram-, Antimicrobial
295	447	DRAMP01919	Brevinin-1PLc (Frogs, amphibians, animals)	Antibacterial, Antifungal, Anti-Gram+, Anti-Gram-, Antimicrobial
296	448	DRAMP01920	Brevinin-1CSa (Frogs, amphibians, animals)	Antibacterial, Anti-Gram+, Anti-Gram-, Antimicrobial
297	450	DRAMP01922	Brevinin-2SKb (Frogs, amphibians, animals)	Antibacterial, Anti-Gram+, Anti-Gram-, Antimicrobial
298	451	DRAMP01933	Brevinin-2Ef (Frogs, amphibians, animals)	Antibacterial, Anti-Gram+, Anti-Gram-, Antimicrobial
299	457	DRAMP01940	Brevinin-1CHa (Frogs, amphibians, animals)	Antibacterial, Antifungal, Anti-Gram+, Anti-Gram-, Antimicrobial
300	458	DRAMP01941	Brevinin-1CHb (Frogs, amphibians, animals)	Antibacterial, Antifungal, Anti-Gram+, Anti-Gram-, Antimicrobial
301	462	DRAMP01949	Brevinin-1HSa (Frogs, amphibians, animals)	Antibacterial, Anti-Gram+, Anti-Gram-, Antimicrobial
302	463	DRAMP01950	Brevinin-1HSb (Brevinin-1JDb; Frogs, amphibians, animals)	Antibacterial, Anti-Gram+, Anti-Gram-, Antimicrobial
303	464	DRAMP01951	Brevinin-1PTa (Frogs, amphibians, animals)	Antibacterial, Anti-Gram+, Anti-Gram-, Antimicrobial
304	465	DRAMP01953	Brevinin-2HSa (Frogs, amphibians, animals)	Antibacterial, Anti-Gram+, Anti-Gram-, Antimicrobial
305	466	DRAMP01955	Brevinin-2PTa (Frogs, amphibians, animals)	Antibacterial, Anti-Gram+, Anti-Gram-, Antimicrobial
306	467	DRAMP01956	Brevinin-2PTb (Frogs, amphibians, animals)	Antibacterial, Anti-Gram+, Anti-Gram-, Antimicrobial
307	468	DRAMP01957	Brevinin-2PTc (Frogs, amphibians, animals)	Antibacterial, Anti-Gram+, Anti-Gram-, Antimicrobial
308	469	DRAMP01959	Brevinin-2PTe (Frogs, amphibians, animals)	Antibacterial, Anti-Gram+, Anti-Gram-, Antimicrobial
309	470	DRAMP01963	Brevinin-1BLa (Frogs, amphibians, animals)	Antibacterial, Antifungal, Anti-Gram+, Anti-Gram-, Antimicrobial
310	471	DRAMP01965	Brevinin-1BLc (Frogs, amphibians, animals)	Antibacterial, Antifungal, Anti-Gram+, Anti-Gram-, Antimicrobial
311	472	DRAMP01968	Brevinin-1Yc (Frogs, amphibians, animals)	Antibacterial, Antifungal, Anti-Gram+, Anti-Gram-, Antimicrobial
312	473	DRAMP01969	Brevinin-1Ja (Frogs, amphibians, animals)	Antibacterial, Anti-Gram+, Anti-Gram-, Antimicrobial
313	474	DRAMP01970	Brevinin-1ZHa (Frogs, amphibians, animals)	Antibacterial, Antifungal, Anti-Gram+, Anti-Gram-, Antimicrobial
314	475	DRAMP01971	Brevinin-1ZHb (Frogs, amphibians, animals)	Antibacterial, Antifungal, Anti-Gram+, Anti-Gram-, Antimicrobial
315	476	DRAMP01974	Brevinin-2ZHa (Frogs, amphibians, animals)	Antibacterial, Antifungal, Anti-Gram+, Anti-Gram-, Antimicrobial
316	477	DRAMP01986	Brevinin-2HS2 (Frogs, amphibians, animals)	Antibacterial, Antifungal, Anti-Gram+, Anti-Gram-, Antimicrobial
317	478	DRAMP01990	Brevinin-1LT1 (Frogs, amphibians, animals)	Antibacterial, Anti-Gram+, Anti-Gram-, Antimicrobial
318	479	DRAMP01994	Brevinin-2ISa (Frogs, amphibians, animals)	Antibacterial, Anti-Gram+, Anti-Gram-, Antimicrobial

S.no.	PepID	DRAMP_ID	Name of the AMP	Activity
319	480	DRAMP01995	Brevinin-2ISb (Frogs, amphibians, animals)	Antibacterial, Antifungal, Anti-Gram+, Anti-Gram-, Antimicrobial
320	481	DRAMP01996	Brevinin-2ISc (Frogs, amphibians, animals)	Antibacterial, Anti-Gram+, Anti-Gram-, Antimicrobial
321	482	DRAMP02001	Brevinin-1HN1 (Frogs, amphibians, animals)	Antibacterial, Antifungal, Anti-Gram+, Anti-Gram-, Antimicrobial
322	483	DRAMP02004	Brevinin-1V (Frogs, amphibians, animals)	Antibacterial, Antifungal, Anti-Gram+, Anti-Gram-, Antimicrobial
323	485	DRAMP02009	Brevinin-1 (Frogs, amphibians, animals)	Antibacterial, Anti-Gram+, Anti-Gram-, Antimicrobial
324	486	DRAMP02010	Brevinin-2 (Frogs, amphibians, animals)	Antibacterial, Anti-Gram+, Anti-Gram-, Antimicrobial
325	487	DRAMP02019	Brevinin-2DYb (Frogs, amphibians, animals)	Antibacterial, Anti-Gram+, Anti-Gram-, Antimicrobial
326	490	DRAMP02023	Brevinin-2DYd (Frogs, amphibians, animals)	Antibacterial, Anti-Gram+, Anti-Gram-, Antimicrobial
327	492	DRAMP02025	Brevinin-2DYe (Brevinin-2CDYa; Frogs, amphibians, animals)	Antibacterial, Anti-Gram+, Anti-Gram-, Antimicrobial
328	493	DRAMP02026	Brevinin-1CDYa (Frogs, amphibians, animals)	Antibacterial, Anti-Gram+, Anti-Gram-, Antimicrobial
329	495	DRAMP02031	Brevinin-1Da (Frogs, amphibians, animals)	Antibacterial, Anti-Gram+, Anti-Gram-, Antimicrobial
330	496	DRAMP02032	Brevinin-1TSa (Frogs, amphibians, animals)	Antibacterial, Antifungal, Anti-Gram+, Anti-Gram-, Antimicrobial
331	497	DRAMP02033	Brevinin-2TSa (Frogs, amphibians, animals)	Antibacterial, Antifungal, Anti-Gram+, Anti-Gram-, Antimicrobial
332	498	DRAMP02034	Brevinin-1AUa (Frogs, amphibians, animals)	Antibacterial, Antifungal, Anti-Gram+, Anti-Gram-, Antimicrobial
333	499	DRAMP02035	Brevinin-1AUb (Frogs, amphibians, animals)	Antibacterial, Antifungal, Anti-Gram+, Anti-Gram-, Antimicrobial
334	500	DRAMP02036	Brevinin-2-RN1 (Frogs, amphibians, animals)	Antibacterial, Antifungal, Anti-Gram+, Anti-Gram-, Antimicrobial
335	501	DRAMP02037	Brevinin-2-RN2 (Frogs, amphibians, animals)	Antibacterial, Antifungal, Anti-Gram+, Anti-Gram-, Antimicrobial
336	502	DRAMP02038	Brevinin-1-OA1 (Frogs, amphibians, animals)	Antibacterial, Antifungal, Anti-Gram+, Anti-Gram-, Antimicrobial
337	504	DRAMP02040	Brevinin-1-OA12 (Frogs, amphibians, animals)	Antibacterial, Antifungal, Anti-Gram+, Anti-Gram-, Antimicrobial
338	505	DRAMP02041	Brevinin-1-OR1 (Frogs, amphibians, animals)	Antibacterial, Antifungal, Anti-Gram+, Anti-Gram-, Antimicrobial
339	506	DRAMP02042	Brevinin-1-OR3 (Frogs, amphibians, animals)	Antibacterial, Antifungal, Anti-Gram+, Anti-Gram-, Antimicrobial
340	507	DRAMP02043	Brevinin-1-OR4 (Frogs, amphibians, animals)	Antibacterial, Antifungal, Anti-Gram+, Anti-Gram-, Antimicrobial
341	508	DRAMP02044	Brevinin-1-OR5 (Frogs, amphibians, animals)	Antibacterial, Antifungal, Anti-Gram+, Anti-Gram-, Antimicrobial
342	509	DRAMP02045	Brevinin-1-OR6 (Frogs, amphibians, animals)	Antibacterial, Antifungal, Anti-Gram+, Anti-Gram-, Antimicrobial
343	510	DRAMP02046	Brevinin-1-OR7 (Frogs, amphibians, animals)	Antibacterial, Antifungal, Anti-Gram+, Anti-Gram-, Antimicrobial
344	511	DRAMP02047	Brevinin-1-OR8 (Frogs, amphibians, animals)	Antibacterial, Antifungal, Anti-Gram+, Anti-Gram-, Antimicrobial
345	512	DRAMP02048	Brevinin-1-OR9 (Frogs, amphibians, animals)	Antibacterial, Antifungal, Anti-Gram+, Anti-Gram-, Antimicrobial
346	513	DRAMP02049	Brevinin-1-OR10 (Frogs, amphibians, animals)	Antibacterial, Antifungal, Anti-Gram+, Anti-Gram-, Antimicrobial
347	514	DRAMP02050	Brevinin-1-OR11 (Frogs, amphibians, animals)	Antibacterial, Antifungal, Anti-Gram+, Anti-Gram-, Antimicrobial
348	515	DRAMP02051	Lividin-1 (Brevinin-1-OR2; Frogs, amphibians, animals)	Antibacterial, Antifungal, Anti-Gram+, Anti-Gram-, Antimicrobial
349	516	DRAMP02052	Lividin-2 (Brevinin-2-OR8; Frogs, amphibians, animals)	Antibacterial, Antifungal, Anti-Gram+, Anti-Gram-, Antimicrobial
350	517	DRAMP02053	Lividin-3 (Brevinin-2-OR1; Frogs, amphibians, animals)	Antibacterial, Antifungal, Anti-Gram+, Anti-Gram-, Antimicrobial
351	518	DRAMP02054	Brevinin-2-OA1 (Frogs, amphibians, animals)	Antibacterial, Antifungal, Anti-Gram+, Anti-Gram-, Antimicrobial
352	519	DRAMP02055	Brevinin-2-OA2 (Brevinin-2E-OG1; Frogs, amphibians, animals)	Antibacterial, Antifungal, Anti-Gram+, Anti-Gram-, Antimicrobial
353	520	DRAMP02056	Brevinin-2-OA3 (Frogs, amphibians, animals)	Antibacterial, Antifungal, Anti-Gram+, Anti-Gram-, Antimicrobial
354	521	DRAMP02057	Brevinin-2-OA4 (Frogs, amphibians, animals)	Antibacterial, Antifungal, Anti-Gram+, Anti-Gram-, Antimicrobial
355	522	DRAMP02058	Brevinin-2-OA5 (Frogs, amphibians, animals)	Antibacterial, Antifungal, Anti-Gram+, Anti-Gram-, Antimicrobial
356	523	DRAMP02059	Brevinin-2-OA6 (Frogs, amphibians, animals)	Antibacterial, Antifungal, Anti-Gram+, Anti-Gram-, Antimicrobial
357	524	DRAMP02060	Brevinin-2-OA7 (Frogs, amphibians, animals)	Antibacterial, Antifungal, Anti-Gram+, Anti-Gram-, Antimicrobial
358	525	DRAMP02061	Brevinin-2-OA8 (Frogs, amphibians, animals)	Antibacterial, Antifungal, Anti-Gram+, Anti-Gram-, Antimicrobial
359	526	DRAMP02062	Brevinin-2-OR2 (Frogs, amphibians, animals)	Antibacterial, Antifungal, Anti-Gram+, Anti-Gram-, Antimicrobial
360	527	DRAMP02063	Brevinin-2-OR3 (Frogs, amphibians, animals)	Antibacterial, Antifungal, Anti-Gram+, Anti-Gram-, Antimicrobial
361	528	DRAMP02064	Brevinin-2-OR4 (Frogs, amphibians, animals)	Antibacterial, Antifungal, Anti-Gram+, Anti-Gram-, Antimicrobial
362	529	DRAMP02065	Brevinin-2-OR5 (Frogs, amphibians, animals)	Antibacterial, Antifungal, Anti-Gram+, Anti-Gram-, Antimicrobial
363	530	DRAMP02066	Brevinin-2-OR6 (Frogs, amphibians, animals)	Antibacterial, Antifungal, Anti-Gram+, Anti-Gram-, Antimicrobial
364	531	DRAMP02067	Brevinin-2-OR7 (Frogs, amphibians, animals)	Antibacterial, Antifungal, Anti-Gram+, Anti-Gram-, Antimicrobial
365	532	DRAMP02068	Brevinin-2-OR9 (Frogs, amphibians, animals)	Antibacterial, Antifungal, Anti-Gram+, Anti-Gram-, Antimicrobial
366	533	DRAMP02069	Brevinin-2-OR10 (Frogs, amphibians, animals)	Antibacterial, Antifungal, Anti-Gram+, Anti-Gram-, Antimicrobial
367	534	DRAMP02070	Brevinin-2-OW1 (Frogs, amphibians, animals)	Antibacterial, Antifungal, Anti-Gram+, Anti-Gram-, Antimicrobial
368	535	DRAMP02071	Brevinin-2-OW2 (Frogs, amphibians, animals)	Antibacterial, Antifungal, Anti-Gram+, Anti-Gram-, Antimicrobial
369	536	DRAMP02072	Brevinin-2-OW3 (Frogs, amphibians, animals)	Antibacterial, Antifungal, Anti-Gram+, Anti-Gram-, Antimicrobial
370	537	DRAMP02073	Brevinin-1JDa (Frogs, amphibians, animals)	Antibacterial, Antifungal, Anti-Gram+, Anti-Gram-, Antimicrobial
371	538	DRAMP02075	Brevinin-1JDc (Frogs, amphibians, animals)	Antibacterial, Anti-Gram+, Anti-Gram-, Antimicrobial

S.no.	PepID	DRAMP_ID	Name of the AMP	Activity
372	543	DRAMP02078	Brevinin-1SY (Frogs, amphibians, animals)	Antibacterial, Anti-Gram+, Anti-Gram-, Antimicrobial
373	544	DRAMP02081	Brevinin-1E (Frogs, amphibians, animals)	Antibacterial, Anti-Gram+, Anti-Gram-, Antimicrobial
374	545	DRAMP02084	Brevinin-2E (Frogs, amphibians, animals)	Antibacterial, Antifungal, Anti-Gram+, Anti-Gram-, Antimicrobial
375	546	DRAMP02101	Brevinin-1RTa (Frogs, amphibians, animals)	Antibacterial, Anti-Gram+, Anti-Gram-, Antimicrobial
376	547	DRAMP02102	Brevinin-1RTb (Frogs, amphibians, animals)	Antibacterial, Anti-Gram+, Anti-Gram-, Antimicrobial
377	548	DRAMP02104	Brevinin-2RTa (Frogs, amphibians, animals)	Antibacterial, Antifungal, Anti-Gram+, Anti-Gram-, Antimicrobial
378	549	DRAMP02105	Brevinin-2RTb (Frogs, amphibians, animals)	Antibacterial, Anti-Gram+, Anti-Gram-, Antimicrobial
379	550	DRAMP02114	Raniseptin-1 (Rsp-1; Frogs, amphibians, animals)	Antibacterial, Anti-Gram+, Anti-Gram-, Antimicrobial
380	551	DRAMP02125	Hylin-a1 (Hy-a1; Frogs, amphibians, animals)	Antibacterial, Antifungal, Anti-Gram+, Anti-Gram-, Antimicrobial
381	554	DRAMP02129	Kasstasin (Frogs, amphibians, animals)	Antibacterial, Anti-Gram+, Anti-Gram-, Antimicrobial
382	555	DRAMP02130	Antimicrobial peptide 1 (XT-1; Frogs, amphibians, animals)	Antibacterial, Antifungal, Anti-Gram+, Anti-Gram-, Antimicrobial
383	556	DRAMP02131	Antimicrobial peptide 2 (XT-2; Frogs, amphibians, animals)	Antibacterial, Anti-Gram+, Anti-Gram-, Antimicrobial
384	557	DRAMP02133	Antimicrobial peptide 4 (XT-4; Frogs, amphibians, animals)	Antibacterial, Antifungal, Anti-Gram+, Anti-Gram-, Antimicrobial
385	558	DRAMP02135	Antimicrobial peptide 6 (XT-6; Frogs, amphibians, animals)	Antibacterial, Antifungal, Anti-Gram+, Anti-Gram-, Antimicrobial
386	559	DRAMP02136	Antimicrobial peptide 7 (XT-7; Frogs, amphibians, animals)	Antibacterial, Antifungal, Anti-Gram+, Anti-Gram-, Antimicrobial
387	561	DRAMP02219	Ranatuerin-2AUa (Frogs, amphibians, animals)	Antimicrobial, Antibacterial, Antifungal, Anti-Gram+, Anti- Gram-,
388	568	DRAMP02228	Ranatuerin-1 (Frogs, amphibians, animals)	Antibacterial, Antifungal, Anti-Gram+, Anti-Gram-, Antimicrobial
389	576	DRAMP02237	Ranatuerin-2Ya (Frogs, amphibians, animals)	Cytolytic,Antibacterial, Anti-Gram+, Anti-Gram-, Antimicrobial
390	577	DRAMP02238	Ranatuerin-2ZHa (Frogs, amphibians, animals)	Antibacterial, Antifungal, Anti-Gram+, Anti-Gram-, Antimicrobial
391	578	DRAMP02239	Ranatuerin-1Ga (Frogs, amphibians, animals)	Antibacterial, Antifungal, Anti-Gram+, Anti-Gram-, Antimicrobial
392	579	DRAMP02241	Ranatuerin-2G (Frogs, amphibians, animals)	Antibacterial, Antifungal, Anti-Gram+, Anti-Gram-, Antimicrobial
393	580	DRAMP01390	Odorranain-T1 (OdT1; Frogs, amphibians, animals)	Antimicrobial, Antibacterial, Antifungal, Anti-Gram+, Anti- Gram-,
394	581	DRAMP01389	Odorranain-S1 (OdS1; Frogs, amphibians, animals)	Antimicrobial, Antibacterial, Antifungal, Anti-Gram+, Anti- Gram-,
395	582	DRAMP02251	Ranatuerin-2CSa (Frogs, amphibians, animals)	Antibacterial, Anti-Gram+, Anti-Gram-, Antimicrobial
396	583	DRAMP02252	Ranatuerin 2SKa (Frogs, amphibians, animals)	Antibacterial, Antifungal, Anti-Gram+, Anti-Gram-, Antimicrobial
397	584	DRAMP01108	Maximin-2 (Toads, amphibians, animals)	Antimicrobial, Antibacterial, Antifungal, Anti-Gram+, Anti-Gram-,
398	586	DRAMP01107	Maximin-1 (Toads, amphibians, animals)	Antimicrobial, Antibacterial, Antifungal, Antiviral, Anticancer, Anti-Gram+, Anti-Gram-,
399	587	DRAMP02268	Xenopsin precursor fragment (XPF; Frogs, amphibians, animals)	Antibacterial, Antifungal, Anti-Gram+, Anti-Gram-, Antimicrobial
400	588	DRAMP02269	Antimicrobial peptide PGQ (PGQ; Frogs, amphibians, animals)	Antibacterial, Antifungal, Anti-Gram+, Anti-Gram-, Antimicrobial
401	589	DRAMP02271	Magainin-2 (Magainin II; chain of Magainins; Frogs, amphibians,	Antibacterial, Antifungal, Antiprotozoal, Anti-Gram+, Anti- Gram-, Antimicrobial
402	590	DRAMP02272	PGLa (chain of PYLa/PGLa A; Frogs, amphibians, animals)	Antibacterial, Antifungal, Anti-Gram+, Anti-Gram-, Antimicrobial
403	591	DRAMP02273	PGLa-H (chain of PYLa/PGLa A; Frogs, amphibians, animals)	Antibacterial, Anti-Gram+, Anti-Gram-, Antimicrobial
404	592	DRAMP02274	Ranacyclin-E (Frogs, amphibians, animals)	Antibacterial, Antifungal, Anti-Gram+, Anti-Gram-, Antimicrobial
405	593	DRAMP02275	Ranacyclin-T (Frogs, amphibians, animals)	Antibacterial, Antifungal, Anti-Gram+, Anti-Gram-, Antimicrobial
406	**596**	DRAMP02278	Ranacyclin-B-RL1 (Frogs, amphibians, animals)	Antibacterial, Antifungal, Anti-Gram+, Anti-Gram-, Antimicrobial
407	602	DRAMP02288	Gaegurin-RN1 (Frogs, amphibians, animals)	Antibacterial, Antifungal, Anti-Gram+, Anti-Gram-, Antimicrobial
408	604	DRAMP02290	Gaegurin-RN5 (Frogs, amphibians, animals)	Antibacterial, Antifungal, Anti-Gram+, Anti-Gram-, Antimicrobial
409	605	DRAMP02291	Gaegurin-1 (Gaegurin 1; GGN1; Frogs, amphibians, animals)	Antibacterial, Antifungal, Anti-Gram+, Anti-Gram-, Antimicrobial
410	606	DRAMP02292	Gaegurin-2 (Gaegurin 2; GGN2; Frogs, amphibians, animals)	Antibacterial, Antifungal, Anti-Gram+, Anti-Gram-, Antimicrobial
411	607	DRAMP02293	Gaegurin-3 (Gaegurin 3; GGN3; Frogs, amphibians, animals)	Antibacterial, Antifungal, Anti-Gram+, Anti-Gram-, Antimicrobial
412	608	DRAMP02294	Gaegurin-4 (Gaegurin 4; GGN4; Frogs, amphibians, animals)	Antibacterial, Antifungal, Antiprotozoal, Anti-Gram+, Anti-Gram-, Antimicrobial
413	609	DRAMP02295	Gaegurin-5 (Gaegurin 5; GGN5; Brevinin-1EMa; Frogs, amphibians,	Antibacterial, Antifungal, Antiprotozoal, Anti-Gram+, Anti-Gram-, Antimicrobial
414	610	DRAMP02296	Gaegurin-6 (Gaegurin 6; GGN6; Frogs, amphibians, animals)	Antibacterial, Antifungal, Anti-Gram+, Anti-Gram-, Antimicrobial
415	616	DRAMP02314	Hepcidin (fish, chordates, animals)	Antibacterial, Antifungal, Anti-Gram+, Anti-Gram-, Antimicrobial
416	617	DRAMP02315	Chrysophsin-1 (fish, chordates, animals)	Antibacterial, Anti-Gram+, Anti-Gram-, Antimicrobial
417	618	DRAMP02316	Chrysophsin-2 (fish, chordates, animals)	Antibacterial, Anti-Gram+, Anti-Gram-, Antimicrobial
418	619	DRAMP02317	Chrysophsin-3 (fish, chordates, animals)	Antibacterial, Anti-Gram+, Anti-Gram-, Antimicrobial
419	620	DRAMP02318	Grammistin Pp1 (Group II grammistin; fish, chordates, animals)	Antibacterial, Anti-Gram+, Anti-Gram-, Antimicrobial
420	621	DRAMP02320	Grammistin PpIIb (Group II grammistin; fish, chordates, animals)	Antibacterial, Anti-Gram+, Anti-Gram-, Antimicrobial
421	622	DRAMP02321	Grammistin Pp3 (Group III grammistin; fish, chordates, animals)	Antibacterial, Anti-Gram+, Anti-Gram-, Antimicrobial
422	623	DRAMP02324	SAMP H1 (fish, chordates, animals)	Antibacterial, Anti-Gram+, Anti-Gram-, Antimicrobial
423	624	DRAMP02330	Piscidin-1 (Pis-1; Piscidin 1; fish, chordates, animals)	Antibacterial, Antifungal, Anti-Gram+, Anti-Gram-, Antimicrobial
424	625	DRAMP02331	Piscidin-2 (Pis-2; fish, chordates, animals)	Antibacterial, Antifungal, Anti-Gram+, Anti-Gram-, Antimicrobial

S.no.	PepID	DRAMP_ID	Name of the AMP	Activity
425	626	DRAMP02336	Oncorhyncin II (Oncorhyncin 2; fish, chordates, animals)	Antibacterial, Anti-Gram+, Anti-Gram-, Antimicrobial
426	627	DRAMP02337	Oncorhyncin III (Oncorhyncin 3; fish, chordates, animals)	Antibacterial, Anti-Gram+, Anti-Gram-, Antimicrobial
427	628	DRAMP02347	NRC-1 (fish, chordates, animals)	Antibacterial, Antifungal, Anti-Gram+, Anti-Gram-, Antimicrobial
428	629	DRAMP02348	NRC-2 (fish, chordates, animals)	Antibacterial, Antifungal, Anti-Gram+, Anti-Gram-, Antimicrobial
429	630	DRAMP02349	NRC-3 (fish, chordates, animals)	Antibacterial, Antifungal, Anti-Gram+, Anti-Gram-, Antimicrobial
430	631	DRAMP02350	Pleurocidin (NRC-4; fish, chordates, animals)	Antibacterial, Antifungal, Anti-Gram+, Anti-Gram-, Antimicrobial
431	632	DRAMP02351	NRC-10 (fish, chordates, animals)	Antibacterial, Antifungal, Anti-Gram+, Anti-Gram-, Antimicrobial
432	633	DRAMP02352	NRC-16 (fish, chordates, animals)	Antibacterial, Antifungal, Anti-Gram+, Anti-Gram-, Antimicrobial
433	634	DRAMP02354	Pleurocidin-like peptide WFY (fish, chordates, animals)	Antibacterial, Antifungal, Anti-Gram+, Anti-Gram-, Antimicrobial
434	635	DRAMP02357	Pleurocidin-like peptide WF3 (NRC-5; fish, chordates, animals)	Antibacterial, Antifungal, Anti-Gram+, Anti-Gram-, Antimicrobial
435	636	DRAMP02358	Pleurocidin-like peptide WF4 (NRC-6; fish, chordates, animals)	Antibacterial, Antifungal, Anti-Gram+, Anti-Gram-, Antimicrobial
436	637	DRAMP02359	Pleurocidin-like peptide YT2 (NRC-7; fish, chordates, animals;	Antibacterial, Antifungal, Anti-Gram+, Anti-Gram-, Antimicrobial
437	638	DRAMP02360	Pleurocidin-like peptide AP1 (NRC-11; fish, chordates, animals;	Antibacterial, Antifungal, Anti-Gram+, Anti-Gram-, Antimicrobial
438	639	DRAMP02361	Pleurocidin-like peptide AP2 (NRC-12; fish, chordates, animals;	Antibacterial, Antifungal, Anti-Gram+, Anti-Gram-, Antimicrobial
439	640	DRAMP02362	Pleurocidin-like peptide AP3 (NRC-13; fish, chordates, animals;	Antibacterial, Antifungal, Anti-Gram+, Anti-Gram-, Antimicrobial
440	641	DRAMP02363	Pleurocidin-like peptide GcSc4C5 (NRC-14; fish, chordates, animals)	Antibacterial, Antifungal, Anti-Gram+, Anti-Gram-, Antimicrobial
441	642	DRAMP02364	Pleurocidin-like peptide GcSc4B7 (NRC-15; fish, chordates, animals;	Antibacterial, Antifungal, Anti-Gram+, Anti-Gram-, Antimicrobial
442	643	DRAMP02365	Pleurocidin-like peptide GC3.8 (NRC-17; fish, chordates, animals;	Antibacterial, Antifungal, Anti-Gram+, Anti-Gram-, Antimicrobial
443	644	DRAMP02366	Pleurocidin-like peptide GC3.2 (NRC-18; fish, chordates, animals;	Antibacterial, Antifungal, Anti-Gram+, Anti-Gram-, Antimicrobial
444	645	DRAMP02367	Pleurocidin-like peptide Hb26 (NRC-19; fish, chordates, animals;	Antibacterial, Antifungal, Anti-Gram+, Anti-Gram-, Antimicrobial
445	646	DRAMP02368	Pleurocidin-like peptide Hb18 (NRC-20; fish, chordates, animals;	Antibacterial, Antifungal, Anti-Gram+, Anti-Gram-, Antimicrobial
446	648	DRAMP02376	Grammistin Gs 1 (Grammistin Gs F; Group I grammistin; soapfish,	Antibacterial, Anti-Gram+, Anti-Gram-, Antimicrobial
447	649	DRAMP02377	Grammistin Gs 2 (Grammistin Gs G; Group I grammistin; soapfish,	Antibacterial, Anti-Gram+, Anti-Gram-, Antimicrobial
448	650	DRAMP02378	Grammistin Gs A (Group III grammistin; soapfish, chordates,	Antibacterial, Anti-Gram+, Anti-Gram-, Antimicrobial
449	651	DRAMP02379	Grammistin Gs B (Group II grammistin; soapfish, chordates, animals)	Antibacterial, Anti-Gram+, Anti-Gram-, Antimicrobial
450	652	DRAMP02380	Grammistin Gs C (Group III grammistin; soapfish, chordates,	Antibacterial, Anti-Gram+, Anti-Gram-, Antimicrobial
451	657	DRAMP02390	Astacidin 2 (crayfish, Arthropods, animals)	Antibacterial, Anti-Gram+, Anti-Gram-, Antimicrobial
452	658	DRAMP02391	Hematopoietic antimicrobial peptide-37 (MgCath37; hagfishes,	Antibacterial, Antifungal, Anti-Gram+, Anti-Gram-, Antimicrobial
453	659	DRAMP02393	HFIAP-1 (HFIAP-2; hagfishes, chordates, animals)	Antibacterial, Antifungal, Anti-Gram+, Anti-Gram-, Antimicrobial
454	660	DRAMP02394	HFIAP-3 (hagfishes, chordates, animals)	Antibacterial, Anti-Gram+, Anti-Gram-, Antimicrobial
455	661	DRAMP02395	Aurelin (jellyfish, chordates, animals)	Antibacterial, Anti-Gram+, Anti-Gram-, Antimicrobial
456	662	DRAMP02397	Big defensin (RPD-1)	Anticancer, Antibacterial, Anti-Gram+, Anti-Gram-, Antimicrobial
457	663	DRAMP02402	Antimicrobial peptide scolopin-1	Antibacterial, Antifungal, Anti-Gram+, Anti-Gram-, Antimicrobial
458	664	DRAMP02403	Antimicrobial peptide scolopin-2	Antibacterial, Antifungal, Anti-Gram+, Anti-Gram-, Antimicrobial
459	665	DRAMP02409	M-theraphotoxin-Gr1a (M-TRTX-Gr1a; GsMTx-4)	Antibacterial, Anti-Gram+, Anti-Gram-, Antimicrobial
460	666	DRAMP02410	Antimicrobial peptide lumbricin-1	Antibacterial, Antifungal, Anti-Gram+, Anti-Gram-, Antimicrobial
461	667	DRAMP02411	Armadillidin (Glyc-rich)	Antibacterial, Anti-Gram+, Anti-Gram-, Antimicrobial
462	668	DRAMP02412	Panusin (Defensin-like peptide 7, PaD7)	Antibacterial, Anti-Gram+, Anti-Gram-, Antimicrobial
463	670	DRAMP02421	Arthropods, animals)	Antibacterial, Anti-Gram+, Anti-Gram-, Antimicrobial
464	671	DRAMP02422	Hlsal-defensin (H. longicornis salivary gland defensin; Ticks,	Antibacterial, Anti-Gram+, Anti-Gram-, Antimicrobial
465	672	DRAMP02423	HlMS-defensin (Ticks, Arthropods, animals)	Antibacterial, Antifungal, Anti-Gram+, Anti-Gram-, Antimicrobial
466	673	DRAMP02425	Ixosin-B (Ticks, Arthropods, animals)	Antibacterial, Antifungal, Anti-Gram+, Anti-Gram-, Antimicrobial
467	678	DRAMP02432	Antimicrobial peptide ISAMP (Ticks, Arthropods, animals)	Antibacterial, Anti-Gram+, Anti-Gram-, Antimicrobial
468	680	DRAMP02434	Antimicrobial peptide lumbricin-PG (Lumbricin-PG)	Antibacterial, Anti-Gram+, Anti-Gram-, Antimicrobial
469	683	DRAMP02445	Antimicrobial protein BL-A60	Antibacterial, Anti-Gram+, Anti-Gram-, Antimicrobial
470	684	DRAMP02446	Antimicrobial protein 1 (Antimicrobial protein AN5-1)	Antibacterial, Anti-Gram+, Anti-Gram-, Antimicrobial
471	687	DRAMP02470	Nosiheptide (NOS; Antibiotic 9671-RP)	Antibacterial, Anti-Gram+, Anti-Gram-, Antimicrobial
472	688	DRAMP02473	Cathelicidin-BF (Cathelicidin-related protein; Snakes, reptiles,	Antibacterial, Antifungal, Anti-Gram+, Anti-Gram-, Antimicrobial
473	689	DRAMP02474	cathelicidin-BF15 (Snakes, reptiles, animals)	Antibacterial, Antifungal, Anti-Gram+, Anti-Gram-, Antimicrobial
474	690	DRAMP02478	L-amino-acid oxidase (Bm-LAO; LAAO; LAO; Snakes, reptiles,	Antibacterial, Antiparasitic, Anti-Gram+, Anti-Gram-, Antimicrobial
475	692	DRAMP02522	L-amino-acid oxidase (LAAO, LAO, Oh-LAAO; Snakes, reptiles,	Antibacterial, Anti-Gram+, Anti-Gram-, Antimicrobial
476	693	DRAMP02573	Penaeidin-3a (Pen-3a; shrimps, Arthropods, animals)	Antibacterial, Antifungal, Anti-Gram+, Anti-Gram-, Antimicrobial
477	694	DRAMP02574	[T8A]-Penaeidin-3a ([T8A]-Pen-3a; shrimps, Arthropods, animals)	Antibacterial, Antifungal, Anti-Gram+, Anti-Gram-, Antimicrobial

S.no.	PepID	DRAMP_ID	Name of the AMP	Activity
478	697	DRAMP02584	Penaeidin-4a (Pen-4a; shrimps, Arthropods, animals)	Antibacterial, Antifungal, Anti-Gram+, Anti-Gram-, Antimicrobial
479	698	DRAMP02586	Penaeidin-2d (Pen-2d; shrimps, Arthropods, animals)	Antibacterial, Antifungal, Anti-Gram+, Anti-Gram-, Antimicrobial
480	700	DRAMP02603	Putative antimicrobial peptide A Northern Europe Heligoland	Antibacterial, Antifungal, Anti-Gram+, Anti-Gram-, Antimicrobial
481	705	DRAMP02740	TBD-1 (Turtle beta-defensin 1; Reptiles, animals)	Antibacterial, Antifungal, Anti-Gram+, Anti-Gram-, Antimicrobial
482	706	DRAMP02768	Pilosulin-1 (Myr b I; ants, insects, animals)	Antibacterial, Antifungal, Anti-Gram+, Anti-Gram-, Antimicrobial
483	709	DRAMP02777	Rhinocerosin (Insects, animals)	Antibacterial, Anti-Gram+, Anti-Gram-, Antimicrobial
484	710	DRAMP02778	Defensin (Insects, animals)	Antibacterial, Anti-Gram+, Anti-Gram-, Antimicrobial
485	711	DRAMP02779	Defensin-A (Defensin A; Insects, animals)	Antibacterial , Anti-Gram+, Anti-Gram-, Antimicrobial
486	712	DRAMP02780	Defensin-B (Defensin B; Insects, animals)	Antibacterial, Anti-Gram+, Anti-Gram-, Antimicrobial
487	713	DRAMP02802	Paneth cell-specific alpha-defensin 1 (DEFA1; horse defensin;	Antibacterial, Antifungal, Anti-Gram+, Anti-Gram-, Antimicrobial
488	714	DRAMP02809	Myticin-B (Myt B; Cys-rich; molluscas, animals)	Antibacterial, Antifungal, Anti-Gram+, Anti-Gram-, Antimicrobial
489	715	DRAMP02811	Defensin MGD-1 (molluscas, animals)	Antibacterial, Anti-Gram+, Anti-Gram-, Antimicrobial
490	717	DRAMP02817	Pyrrhocoricin	Antibacterial, Anti-Gram+, Anti-Gram-, Antimicrobial
491	719	DRAMP01381	Odorranain-K1 (OdK1; Frogs, amphibians, animals)	Antimicrobial, Antibacterial, Antifungal, Anti-Gram+, Anti-Gram-,
492	720	DRAMP01383	Odorranain-M1 (OdM1; Frogs, amphibians, animals)	Antimicrobial, Antibacterial, Antifungal, Anti-Gram+, Anti- Gram-,
493	721	DRAMP02841	Lumbricin I(6-34)	Antibacterial, Antifungal, Anti-Gram+, Anti-Gram-, Antimicrobial
494	722	DRAMP02843	chain a, Structure Of An Indolicidin Peptide Derivative	Antibacterial, Anti-Gram+, Anti-Gram-, Antimicrobial
495	724	DRAMP02845	CP-11 (cathelicidin; mammals, animals)	Antibacterial, Anti-Gram+, Anti-Gram-, Antimicrobial
496	726	DRAMP02851	Cathelicidin-1 (Bactenecin-1, Bac1; Cyclic dodecapeptide; mammals,	Antibacterial, Anti-Gram+, Anti-Gram-, Antimicrobial
497	727	DRAMP02854	Cathelicidin-5 (Antibacterial peptide BMAP-28))	Antibacterial, Antifungal , Anti-Gram+, Anti-Gram-, Antimicrobial
498	728	DRAMP02855	Cathelicidin-6 (Antibacterial peptide BMAP-27)	Antibacterial, Antifungal, Anti-Gram+, Anti-Gram-, Antimicrobial
499	730	DRAMP02859	Bovine Beta-defensin 2 (bBD-2; BNBD-2; BNDB-2; mammals,	Antibacterial, Anti-Gram+, Anti-Gram-, Antimicrobial
500	731	DRAMP02860	Bovine Beta-defensin 3 (bBD-3; BNBD-3; BNDB-3; mammals,	Antibacterial, Anti-Gram+, Anti-Gram-, Antimicrobial
501	732	DRAMP02861	Bovine Beta-defensin 4 (bBD-4; BNBD-4; BNDB-4; mammals,	Antibacterial, Anti-Gram+, Anti-Gram-, Antimicrobial
502	734	DRAMP02863	Bovine Beta-defensin 6 (bBD-6; BNBD-6; BNDB-6; mammals,	Antibacterial, Anti-Gram+, Anti-Gram-, Antimicrobial
503	735	DRAMP02865	Bovine Beta-defensin 8 (bBD-8; BNBD-8; BNDB-8; mammals,	Antibacterial, Anti-Gram+, Anti-Gram-, Antimicrobial
504	736	DRAMP02866	Bovine Beta-defensin 9 (bBD-9; BNBD-9; BNDB-9; mammals,	Antibacterial, Anti-Gram+, Anti-Gram-, Antimicrobial
505	737	DRAMP02867	Bovine Beta-defensin 10 (bBD-10; BNBD-10; BNDB-10; mammals,	Antibacterial, Anti-Gram+, Anti-Gram-, Antimicrobial
506	738	DRAMP02868	Bovine Beta-defensin 11 (bBD-11; BNBD-11; BNDB-11; mammals,	Antibacterial, Anti-Gram+, Anti-Gram-, Antimicrobial
507	739	DRAMP02869	Bovine Beta-defensin 12 (bBD-12; BNBD-12; BNDB-12; mammals,	Antibacterial, Anti-Gram+, Anti-Gram-, Antimicrobial
508	740	DRAMP02870	Bovine Beta-defensin 13 (bBD-13; BNBD-13; BNDB-13; mammals,	Antibacterial, Anti-Gram+, Anti-Gram-, Antimicrobial
509	741	DRAMP02872	Myeloid antimicrobial peptide BMAP-27 (1-18) (mammals, animals)	Antibacterial, Antifungal, Anti-Gram+, Anti-Gram-, Antimicrobial
510	742	DRAMP02873	Myeloid antimicrobial peptide BMAP-28 (1-18) (mammals, animals)	Antibacterial, Antifungal, Anti-Gram+, Anti-Gram-, Antimicrobial
511	744	DRAMP02877	mBMAP28 (mammals, animals)	Antibacterial, Anti-Gram+, Anti-Gram-, Antimicrobial
512	745	DRAMP02878	Tracheal antimicrobial peptide (TAP; mammals, animals)	Antibacterial, Antifungal, Anti-Gram+, Anti-Gram-, Antimicrobial
513	746	DRAMP02903	Bombin H7	Antibacterial, Anti-Gram+, Anti-Gram-, Antimicrobial
514	751	DRAMP02922	Canine beta-defensin (dogs, mammals, animals)	Antibacterial, Antifungal, Anti-Gram+, Anti-Gram-, Antimicrobial
515	752	DRAMP02923	cBD-1 (Canine beta-defensin 1; dogs, mammals, animals)	Antibacterial, Antifungal, Anti-Gram+, Anti-Gram-, Antimicrobial
516	755	DRAMP02925	Cathelicidin (dogs, mammals, animals)	Antibacterial, Antifungal, Anti-Gram+, Anti-Gram-, Antimicrobial
517	756	DRAMP02931	Arasin-likeSp (crabs, Arthropods, animals)	Antibacterial, Anti-Gram+, Anti-Gram-, Antimicrobial
518	758	DRAMP02933	Polyphemusin-1 (PM1; crabs, Arthropods, animals)	Antibacterial, Antifungal, Anti-Gram+, Anti-Gram-, Antimicrobial
519	759	DRAMP02934	PM1-S (linear derivative of PM1)	Antibacterial, Antifungal, Anti-Gram+, Anti-Gram-, Antimicrobial
520	761	DRAMP02951	PtALF6 (Portunus trituberculatus anti-lipopolysaccharide factor	Antibacterial, Antifungal, Anti-Gram+, Anti-Gram-, Antimicrobial
521	762	DRAMP02952	PtALF7 (Portunus trituberculatus anti-lipopolysaccharide factor	Antibacterial, Anti-Gram+, Anti-Gram-, Antimicrobial
522	763	DRAMP02953	Arasin-1 (Pro-rich, Arg-rich; crabs, Arthropods, animals)	Antibacterial, Anti-Gram+, Anti-Gram-, Antimicrobial
523	764	DRAMP02956	Dolabellanin B2	Antibacterial, Antifungal, Anti-Gram+, Anti-Gram-, Antimicrobial
524	765	DRAMP02959	Antibacterial protein PR-39 (pigs, mammals, animals)	Antibacterial, Anti-Gram+, Anti-Gram-, Antimicrobial
525	766	DRAMP02960	Antibacterial peptide PMAP-23 (Myeloid antibacterial peptide 23;	Antibacterial, Anti-Gram+, Anti-Gram-, Antimicrobial
526	767	DRAMP02961	Antibacterial peptide PMAP-37 (Myeloid antibacterial peptide 37;	Antibacterial, Anti-Gram+, Anti-Gram-, Antimicrobial
527	768	DRAMP02962	Antibacterial peptide PMAP-36 (Myeloid antibacterial peptide 36;	Antibacterial, Anti-Gram+, Anti-Gram-, Antimicrobial
528	769	DRAMP02963	PMAP-36(1-20)	Antibacterial, Antifungal, Anti-Gram+, Anti-Gram-, Antimicrobial
529	770	DRAMP02964	PMAP-36(1-34)	Antibacterial, Antifungal, Anti-Gram+, Anti-Gram-, Antimicrobial
530	771	DRAMP02965	PMAP-36(1-35)2	Antibacterial, Antifungal, Anti-Gram+, Anti-Gram-, Antimicrobial

S.no.	PepID	DRAMP_ID	Name of the AMP	Activity
531	772	DRAMP02966	DBI(32-86) (pigs, mammals, animals)	Antibacterial, Anti-Gram+, Anti-Gram-, Antimicrobial
532	773	DRAMP02970	Protegrin-1 (Protegrin 1; PG-1; pigs, mammals, animals)	Antibacterial, Anti-Gram+, Anti-Gram-, Antimicrobial
533	774	DRAMP02975	Tritrpticin (Trp-rich; pigs, mammals, animals)	Antibacterial, Antifungal, Anti-Gram+, Anti-Gram-, Antimicrobial
534	776	DRAMP01376	Odorranain-F1 (OdF1; Frogs, amphibians, animals)	Antimicrobial, Antibacterial, Antifungal, Anti-Gram+, Anti-Gram-,
535	777	DRAMP01377	Odorranain-G1 (OdG1; Frogs, amphibians, animals)	Antimicrobial, Antibacterial, Antifungal, Anti-Gram+, Anti-Gram-,
536	778	DRAMP02995	Hymenoptaecin (Insects, animals)	Antibacterial, Anti-Gram+, Anti-Gram-, Antimicrobial
537	779	DRAMP02996	Apidaecin-2 (Apidaecin II; Insects, animals)	Antibacterial, Anti-Gram+, Anti-Gram-, Antimicrobial
538	780	DRAMP01378	Odorranain-H1 (OdH1; Frogs, amphibians, animals)	Antimicrobial, Antibacterial, Antifungal, Anti-Gram+, Anti- Gram-,
539	781	DRAMP02998	Apidaecin-1A (Apidaecin IA; Insects, animals)	Antibacterial, Anti-Gram+, Anti-Gram-, Antimicrobial
540	782	DRAMP02999	Jellein-1 (Jelleine-I; chain of Major royal jelly protein 1; Insects,	Antibacterial, Antifungal, Anti-Gram+, Anti-Gram-, Antimicrobial
541	783	DRAMP03000	Jellein-2 (Jelleine-II; chain of Major royal jelly protein 1; Insects,	Antibacterial, Antifungal, Anti-Gram+, Anti-Gram-, Antimicrobial
542	784	DRAMP03001	Jellein-3 (Jelleine-III; Insects, animals)	Antibacterial, Antifungal, Anti-Gram+, Anti-Gram-, Antimicrobial
543	785	DRAMP03002	Melittin (Allergen Api m 3; Allergen Api m III; Insects, animals)	Antibacterial, Antifungal, Anti-Gram+, Anti-Gram-, Antimicrobial
544	786	DRAMP03003	Melectin (MEP; Insects, animals)	Antibacterial, Anti-Gram+, Anti-Gram-, Antimicrobial
545	787	DRAMP03007	Osmin (Insects, animals)	Antibacterial, Antifungal, Anti-Gram+, Anti-Gram-, Antimicrobial
546	788	DRAMP03019	Mastoparan PDD-B	Antibacterial, Anti-Gram+, Anti-Gram-, Antimicrobial
547	789	DRAMP03020	Mastoparan PDD-A	Antibacterial, Anti-Gram+, Anti-Gram-, Antimicrobial
548	790	DRAMP03021	Mastoparan PMM	Antibacterial, Anti-Gram+, Anti-Gram-, Antimicrobial
549	791	DRAMP03022	Mastoparan MP	Antibacterial, Anti-Gram+, Anti-Gram-, Antimicrobial
550	792	DRAMP03028	Mastoparan-1 (MP-1; Venom protein MP-1; Insects, animals)	Antibacterial, Anti-Gram+, Anti-Gram-, Antimicrobial
551	793	DRAMP03033	Mastoparan-like peptide 12a (Insects, animals)	Antibacterial, Antifungal, Anti-Gram+, Anti-Gram-, Antimicrobial
552	794	DRAMP03034	Mastoparan-like peptide 12b (Insects, animals)	Antibacterial, Antifungal, Anti-Gram+, Anti-Gram-, Antimicrobial
553	795	DRAMP03035	Mastoparan-like peptide 12c (Insects, animals)	Antibacterial, Antifungal, Anti-Gram+, Anti-Gram-, Antimicrobial
554	796	DRAMP03036	Mastoparan-like peptide 12d (Insects, animals)	Antibacterial, Antifungal, Anti-Gram+, Anti-Gram-, Antimicrobial
555	797	DRAMP03037	Eumenitin (Er-12; Insects, animals)	Antibacterial, Anti-Gram+, Anti-Gram-, Antimicrobial
556	798	DRAMP03038	Eumenitin-R (Insects, animals)	Antibacterial, Antifungal, Anti-Gram+, Anti-Gram-, Antimicrobial
557	799	DRAMP03039	Eumenitin-F (Insects, animals)	Antibacterial, Antifungal, Anti-Gram+, Anti-Gram-, Antimicrobial
558	800	DRAMP03040	Eumenine mastoparan-EF (EMP-EF; Insects, animals)	Antibacterial, Antifungal, Anti-Gram+, Anti-Gram-, Antimicrobial
559	801	DRAMP03041	Eumenine mastoparan-ER (EMP-ER; Insects, animals)	Antibacterial, Antifungal, Anti-Gram+, Anti-Gram-, Antimicrobial
560	802	DRAMP03042	Eumenine mastoparan-AF (EMP-AF; Af-113; Insects, animals)	Antibacterial, Anti-Gram+, Anti-Gram-, Antimicrobial
561	803	DRAMP03043	Agelaia-mastoparan (Agelaia-MP; Insects, animals)	Antibacterial, Anti-Gram+, Anti-Gram-, Antimicrobial
562	804	DRAMP03044	Protonectin (Agelaia-chemotactic peptide, Agelaia-CP; Insects,	Antibacterial, Anti-Gram+, Anti-Gram-, Antimicrobial
563	805	DRAMP03045	Defensin-NV (Insects, animals)	Antibacterial, Antifungal, Anti-Gram+, Anti-Gram-, Antimicrobial
564	807	DRAMP03047	Venom peptide 2-long (OdVP2L; analog of OdVP2; Insects, animals)	Antibacterial, Antifungal, Anti-Gram+, Anti-Gram-, Antimicrobial
565	808	DRAMP03050	Dominulin-A (Insects, animals)	Antibacterial, Anti-Gram+, Anti-Gram-, Antimicrobial
566	809	DRAMP03051	Dominulin-B (Insects, animals)	Antibacterial, Anti-Gram+, Anti-Gram-, Antimicrobial
567	813	DRAMP03055	PP30 (Pro-rich; abaecin-like; Insects, animals)	Antibacterial, Anti-Gram+, Anti-Gram-, Antimicrobial
568	814	DRAMP03056	Decoralin (Insects, animals)	Antibacterial, Antifungal, Anti-Gram+, Anti-Gram-, Antimicrobial
569	815	DRAMP03057	Thanatin (Insects, animals)	Antibacterial, Antifungal, Anti-Gram+, Anti-Gram-, Antimicrobial
570	818	DRAMP03075	Cecropin-D	Antibacterial, Anti-Gram+, Anti-Gram-, Antimicrobial
571	819	DRAMP03089	Drosophila cecropin-A1/A2 (Insects, animals)	Antibacterial, Anti-Gram+, Anti-Gram-, Antimicrobial
572	820	DRAMP03090	Drosophila cecropin B (CecB; Insects, animals)	Antibacterial, Antifungal, Anti-Gram+, Anti-Gram-, Antimicrobial
573	821	DRAMP03095	Andropin (Insects, animals)	Antibacterial, Anti-Gram+, Anti-Gram-, Antimicrobial
574	823	DRAMP18495	Gomesin (Gm; Spiders, arachnids, Chelicerata, arthropods,	Antimicrobial, Antibacterial, Antifungal, Antiparasitic, Antimalarial, Antic, Anti-Gram+, Anti-Gram-,
575	825	DRAMP03104	Sapecin (defensins; Insects, animals)	Antibacterial, Anti-Gram+, Anti-Gram-, Antimicrobial
576	827	DRAMP03116	Ceratotoxin-C (Insects, animals)	Antibacterial, Anti-Gram+, Anti-Gram-, Antimicrobial
577	828	DRAMP03117	Drosophila cecropin-A1 (Insects, animals)	Antibacterial, Anti-Gram+, Anti-Gram-, Antimicrobial
578	830	DRAMP03138	Cecropin-A (Insects, animals)	Antibacterial, Antifungal, Anti-Gram+, Anti-Gram-, Antimicrobial
579	831	DRAMP03140	Anopheles cecropin-A amidated isoform (Insects, animals)	Antibacterial, Antifungal, Anti-Gram+, Anti-Gram-, Antimicrobial
580	832	DRAMP03150	Gambicin (Insects, animals)	Antibacterial, Antifungal, Antiparasitic, Anti-Gram+, Anti- Gram-, Antimicrobial
581	833	DRAMP03153	27 kDa antibacterial protein	Antibacterial, Anti-Gram+, Anti-Gram-, Antimicrobial
582	835	DRAMP03166	P15 (deer beta-defensin; ruminant, animals)	Antibacterial, Antifungal, Anti-Gram+, Anti-Gram-, Antimicrobial
583	837	DRAMP03173	Arenicin-1 (Ar-1; marine polychaeta, animals)	Antibacterial, Antifungal, Cytotoxicity, Anti-Gram+, Anti- Gram-, Antimicrobial

S.no.	PepID	DRAMP_ID	Name of the AMP	Activity
584	839	DRAMP03181	Spinigerin (Insects, animals)	Antibacterial, Antifungal, Anti-Gram+, Anti-Gram-, Antimicrobial
585	840	DRAMP03186	Spheniscin-2 (Sphe-2; penguin avian beta-defensin 103b; birds,	Antibacterial, Antifungal, Anti-Gram+, Anti-Gram-, Antimicrobial
586	841	DRAMP03187	Beta defensin 1(BD-1; mammals, animals)	Antibacterial, Antifungal, Anti-Gram+, Anti-Gram-, Antimicrobial
587	845	DRAMP03198	Alpha-defensin PhD-4 (primates, mammals, animals)	Antibacterial, Antifungal, Anti-Gram+, Anti-Gram-, Antimicrobial
588	846	DRAMP03215	Gomesin (Gm; spiders, Arthropods, animals)	Antibacterial, Antifungal, Anti-Gram+, Anti-Gram-, Antimicrobial
589	847	DRAMP03216	Oxyopinin-4a (Oxt-4a; spiders, Arthropods, animals)	Antibacterial, Anti-Gram+, Anti-Gram-, Antimicrobial
590	848	DRAMP03217	M-oxotoxin-Ot1a (Oxyopinin-1, Oxki1; spiders, Arthropods, animals)	Antibacterial, Insecticidal, Anti-Gram+, Anti-Gram-, Antimicrobial
591	849	DRAMP03222	M-ctenitoxin-Cs1a (M-CNTX-Cs1a; Cupiennin-1a; spiders,	Antibacterial, Insecticidal, Anti-Gram+, Anti-Gram-, Antimicrobial
592	850	DRAMP03225	M-ctenitoxin-Cs1d (M-CNTX-Cs1d; Cupiennin-1d; spiders,	Antibacterial, Anti-Gram+, Anti-Gram-, Antimicrobial
593	851	DRAMP03226	M-zodatoxin-Lt1a (M-ZDTX-Lt1a; Latarcin-1, Ltc-1, Ltc1; spiders,	Antibacterial, Antifungal, Anti-Gram+, Anti-Gram-, Antimicrobial
594	852	DRAMP03227	M-zodatoxin-Lt2a (M-ZDTX-Lt2a; Latarcin-2a, Ltc-2a, Ltc2a; spiders,	Antibacterial, Antifungal, Anti-Gram+, Anti-Gram-, Antimicrobial
595	853	DRAMP03229	M-zodatoxin-Lt3a (M-ZDTX-Lt3a; Latarcin-3a, Ltc-3a; spiders,	Antibacterial, Antifungal, Anti-Gram+, Anti-Gram-, Antimicrobial
596	854	DRAMP03230	M-zodatoxin-Lt3b (M-ZDTX-Lt3b; Latarcin-3b, Ltc-3b; spiders,	Antibacterial, Antifungal, Anti-Gram+, Anti-Gram-, Antimicrobial
597	855	DRAMP03231	M-zodatoxin-Lt4a (M-ZDTX-Lt4a; Latarcin-4a, Ltc-4a; spiders,	Antibacterial, Antifungal, Anti-Gram+, Anti-Gram-, Antimicrobial
598	856	DRAMP03232	M-zodatoxin-Lt4b (M-ZDTX-Lt4b; Latarcin-4b, Ltc-4b; spiders,	Antibacterial, Antifungal, Anti-Gram+, Anti-Gram-, Antimicrobial
599	857	DRAMP03233	M-zodatoxin-Lt5a (M-ZDTX-Lt5a; Latarcin-5, Ltc-5; spiders,	Antibacterial, Antifungal, Anti-Gram+, Anti-Gram-, Antimicrobial
600	858	DRAMP03236	M-zodatoxin-Lt8a (M-ZDTX-Lt8a; Cytoinsectotoxin-1a, CIT- 1a;	Antibacterial, Insecticidal, Anti-Gram+, Anti-Gram-, Antimicrobial
601	859	DRAMP03253	M-lycotoxin-Ls3a (M-LCTX-Ls3a; Lycocitin-1; spiders, Arthropods,	Antibacterial, Antifungal, Anti-Gram+, Anti-Gram-, Antimicrobial
602	860	DRAMP03254	M-lycotoxin-Ls3b (M-LCTX-Ls3b; Lycocitin-2; spiders, Arthropods,	Antibacterial, Antifungal, Anti-Gram+, Anti-Gram-, Antimicrobial
603	861	DRAMP03278	M-lycotoxin-Hc1a (M-LCTX-Hc1a; Lycotoxin I; spiders, Arthropods,	Antibacterial, Antifungal, Anti-Gram+, Anti-Gram-, Antimicrobial
604	862	DRAMP03279	M-lycotoxin-Hc2a (M-LCTX-Hc2a; Lycotoxin-2; Lycotoxin II; spiders,	Antibacterial, Antifungal, Anti-Gram+, Anti-Gram-, Antimicrobial
605	863	DRAMP03280	AcAMP (A. clavatus antimicrobial peptide)	Antibacterial, Antifungal, Antiviral, Anti-Gram+, Anti-Gram-, Antimicrobial
606	864	DRAMP03285	Ostricacin-1 (Beta-defensin 2; Birds, animals)	Antibacterial, Anti-Gram+, Anti-Gram-, Antimicrobial
607	865	DRAMP03286	Ostricacin-2 (Beta-defensin 1; Birds, animals)	Antibacterial, Antifungal, Anti-Gram+, Anti-Gram-, Antimicrobial
608	866	DRAMP03287	Ostricacin-3 (Beta-defensin 7; Birds, animals)	Antibacterial, Anti-Gram+, Anti-Gram-, Antimicrobial
609	867	DRAMP03288	Ostricacin-4 (Beta-defensin 8; Birds, animals)	Antibacterial, Anti-Gram+, Anti-Gram-, Antimicrobial
610	868	DRAMP03311	Stomoxyn (Insects, animals)	Antibacterial, Anti-Gram+, Anti-Gram-, Antimicrobial
611	873	DRAMP03405	mCRAMP-1 (mouse cathelin-related antimicrobial peptide 1;	Antibacterial, Antifungal, Anti-Gram+, Anti-Gram-, Antimicrobial
612	874	DRAMP03406	mCRAMP-2 (mouse cathelin-related antimicrobial peptide 2;	Antibacterial, Antifungal, Anti-Gram+, Anti-Gram-, Antimicrobial
613	875	DRAMP03419	Neutrophil antibiotic peptide NP-1 (RatNP-1; Rodents, mammals,	Antibacterial, Antifungal, Anti-Gram+, Anti-Gram-, Antimicrobial
614	876	DRAMP03422	Neutrophil antibiotic peptide NP-4 (RatNP-4; Rodents, mammals,	Antibacterial, Antifungal, Antiviral, Anti-Gram+, Anti-Gram-, Antimicrobial
615	878	DRAMP03464	Cryptonin (Insects, animals)	Antibacterial, Antifungal, Anti-Gram+, Anti-Gram-, Antimicrobial
616	880	DRAMP03467	Antibacterial napin (Plants)	Antibacterial, Anti-Gram+, Anti-Gram-, Antimicrobial
617	881	DRAMP03471	Recombinant Crassostrea Gigas Defensin (Cg-Def; molluscs,	Antibacterial, Antifungal, Anti-Gram+, Anti-Gram-, Antimicrobial
618	882	DRAMP03472	cgUbiquitin	Antibacterial, Antifungal, Anti-Gram+, Anti-Gram-, Antimicrobial
619	885	DRAMP03486	Manduca Sexta Moricin (MS moricin; Insects, animals)	Antibacterial, Anti-Gram+, Anti-Gram-, Antimicrobial
620	887	DRAMP03507	Cecropin-B (Insects, animals)	Antibacterial, Antifungal, Anti-Gram+, Anti-Gram-, Antimicrobial
621	888	DRAMP03513	G. mellonella moricin-like peptide A (Gm-mlpA; Insects, animals;	Antibacterial, Antifungal, Anti-Gram+, Anti-Gram-, Antimicrobial
622	889	DRAMP03514	G. mellonella moricin-like peptide B (Gm-mlpB; Insects, animals;	Antibacterial, Antifungal, Anti-Gram+, Anti-Gram-, Antimicrobial
623	890	DRAMP03515	Moricin-like peptide C1 (Gm-mlpC1; Insects, animals; Predicted)	Antibacterial, Antifungal, Anti-Gram+, Anti-Gram-, Antimicrobial
624	891	DRAMP03516	Moricin-like peptide C2 (Gm-mlpC2; Insects, animals; Predicted)	Antibacterial, Antifungal, Anti-Gram+, Anti-Gram-, Antimicrobial
625	892	DRAMP03517	Moricin-like peptide C3 (Gm-mlpC3; Insects, animals; Predicted)	Antibacterial, Antifungal, Anti-Gram+, Anti-Gram-, Antimicrobial
626	903	DRAMP03532	Moricin-1 (Insects, animals)	Antibacterial, Anti-Gram+, Anti-Gram-, Antimicrobial
627	908	DRAMP03567	KR-20 (Derived from LL-37)	Antibacterial, Antifungal, Anti-Gram+, Anti-Gram-, Antimicrobial
628	909	DRAMP03568	RK-31 (Derived from LL-37)	Antibacterial, Antifungal, Anti-Gram+, Anti-Gram-, Antimicrobial
629	910	DRAMP03569	KS-30 (Derived from LL-37)	Antibacterial, Antifungal, Anti-Gram+, Anti-Gram-, Antimicrobial
630	911	DRAMP03570	LL-23 (Derived from LL-37)	Antibacterial, Antifungal, Anti-Gram+, Anti-Gram-, Antimicrobial
631	912	DRAMP03571	Antibacterial protein LL-37 (one chain of hCAP-18; Human,	Antibacterial, Anticancer, Anti-Gram+, Anti-Gram-, Antimicrobial
632	915	DRAMP03598	Human beta-defensin 2 (hBD-2; Defensin, beta 2; Beta-defensin 4A;	Antibacterial, Antiviral, Anti-Gram+, Anti-Gram-, Antimicrobial
633	916	DRAMP03599	Human beta-defensin 3 (BD-3, hBD-3; Hbd3; Beta-defensin 103;	Antibacterial, Antifungal, Anti-Gram+, Anti-Gram-, Antimicrobial
634	917	DRAMP03600	Human beta-defensin 4 (hBD-4, BD-4; Beta-defensin 104; Human,	Antibacterial, Anti-Gram+, Anti-Gram-, Antimicrobial
635	918	DRAMP03603	Human beta-defensin 28 (hBD-28; hBD28; Human, mammals,	Antibacterial, Anti-Gram+, Anti-Gram-, Antimicrobial
636	919	DRAMP03638	VpBD (V.philippinarum beta defensin; big defensin)	Antibacterial, Anti-Gram+, Anti-Gram-, Antimicrobial

S.no.	PepID	DRAMP_ID	Name of the AMP	Activity
637	920	DRAMP03642	Chicken heterophil peptides 1 (Antimicrobial peptide CHP1; Birds,	Antibacterial, Antifungal, Anti-Gram+, Anti-Gram-, Antimicrobial
638	921	DRAMP03645	Cathelicidin-2 (CATH-2; Fowlicidin-2; Birds, animals)	Antibacterial, Anti-Gram+, Anti-Gram-, Antimicrobial
639	922	DRAMP03646	Cathelicidin-3 (CATH-3; Fowlicidin-3; Birds, animals)	Antibacterial, Anti-Gram+, Anti-Gram-, Antimicrobial
640	923	DRAMP03647	Cathelicidin-B1 (CATH-B1; cathelicidin; Birds, animals)	Antibacterial, Anti-Gram+, Anti-Gram-, Antimicrobial
641	925	DRAMP03676	GLFcin (Lactoferrin fragment)	Antibacterial, Anti-Gram+, Anti-Gram-, Antimicrobial
642	926	DRAMP03677	GLFcin II (Lactoferrin fragment)	Antibacterial, Anti-Gram+, Anti-Gram-, Antimicrobial
643	927	DRAMP03679	Cathelicidin-2 (Bactenecin-5, Bac5; ChBac5; ruminant, animals)	Antibacterial, Anti-Gram+, Anti-Gram-, Antimicrobial
644	928	DRAMP03682	Vespid chemotactic peptide 5e (VCP 5e; Insects, animals)	Antibacterial, Antifungal, Anti-Gram+, Anti-Gram-, Antimicrobial
645	929	DRAMP03683	Vespid chemotactic peptide 5g (VCP 5g; Insects, animals)	Antibacterial, Antifungal, Anti-Gram+, Anti-Gram-, Antimicrobial
646	930	DRAMP03684	Vespid chemotactic peptide 5f (VCP 5f; Insects, animals)	Antibacterial, Antifungal, Anti-Gram+, Anti-Gram-, Antimicrobial
647	931	DRAMP03687	TsAP-1 (T. serrulatus antimicrobial peptide 1; scorpions, arachnids,	Antibacterial, Anticancer, Anti-Gram+, Anti-Gram-, Antimicrobial
648	933	DRAMP03691	Im-1 (Arthropods, animals)	Antibacterial, Anti-Gram+, Anti-Gram-, Antimicrobial
649	934	DRAMP03693	Bactridin-1 (Bact1; Bactridine 1; Arthropods, animals)	Antibacterial, Anti-Gram+, Anti-Gram-, Antimicrobial
650	935	DRAMP03694	Bactridin-2 (Bact2, Bactridine 2; P-Mice-Antm-beta* NaTx14.8;	Antibacterial, Anti-Gram+, Anti-Gram-, Antimicrobial
651	937	DRAMP03702	Mucroporin (Antimicrobial peptide 36.21; Arthropods, animals)	Antibacterial, Anti-Gram+, Anti-Gram-, Antimicrobial
652	938	DRAMP03706	Antimicrobial peptide 1 (AamAP1; Arthropods, animals)	Antibacterial, Antifungal, Anti-Gram+, Anti-Gram-, Antimicrobial
653	939	DRAMP03707	Antimicrobial peptide 2 (AamAP2; Arthropods, animals)	Antibacterial, Antifungal, Anti-Gram+, Anti-Gram-, Antimicrobial
654	940	DRAMP03714	Amphiphatic peptide 5.13, NDBP-5.13; Arthropods, animals)	Antibacterial, Anti-Gram+, Anti-Gram-, Antimicrobial
655	941	DRAMP03715	Amphiphatic peptide 5.14, NDBP-5.14; Arthropods, animals)	Antibacterial, Anti-Gram+, Anti-Gram-, Antimicrobial
656	942	DRAMP03721	Cytotoxic linear peptide IsCT (IsCT; NDBP-5.2; Arthropods, animals)	Antibacterial, Anti-Gram+, Anti-Gram-, Antimicrobial
657	943	DRAMP03723	Pandinin-1 (Pin1; Arthropods, animals)	Antibacterial, Anti-Gram+, Anti-Gram-, Antimicrobial
658	944	DRAMP03724	Pandinin-2 (Pin2; Arthropods, animals)	Antibacterial, Hemolytic activity, Anti-Gram+, Anti-Gram-, Antimicrobial
659	946	DRAMP03734	Parabutoporin (PP; Non-disulfide-bridged peptide 3.2, NDBP- 3.2;	Antibacterial, Antifungal, Anti-Gram+, Anti-Gram-, Antimicrobial
660	947	DRAMP03735	Opistoporin-1 (OP1; Non-disulfide-bridged peptide 3.5; Opistoporin-	Antibacterial, Antifungal, Anti-Gram+, Anti-Gram-, Antimicrobial
661	948	DRAMP03738	Scorpine (defensins; Arthropods, animals)	Antibacterial, Anti-Gram+, Anti-Gram-, Antimicrobial
662	949	DRAMP02828	BMAP-34 (BMAP 34, bovine cathelicidin, cattle, ruminant, mammals,	Antimicrobial, Antibacterial, Antifungal, Anti-Gram+, Anti-Gram-,
663	951	DRAMP03746	Peptide BmKn2 (Biologically active peptide 4; NDBP-5.1; Arthropods,	Antibacterial, Anti-Gram+, Anti-Gram-, Antimicrobial
664	952	DRAMP03748	Bradykinin-potentiating peptide BmK3 (Bpp BmK3; NDBP-3.3;	Antibacterial, Antifungal, Anti-Gram+, Anti-Gram-, Antimicrobial
665	953	DRAMP03750	Venom antimicrobial peptide-6 (Meucin-13; NDBP-5; Arthropods,	Antibacterial, Antifungal, Anti-Gram+, Anti-Gram-, Antimicrobial
666	954	DRAMP03751	Venom antimicrobial peptide-9 (Meucin-18; NDBP-5; Arthropods,	Antibacterial, Antifungal, Anti-Gram+, Anti-Gram-, Antimicrobial
667	955	DRAMP03752	Peptide BmKb1 (Non-disulfide-bridged peptide 4.2, NDBP-4.2;	Antibacterial, Anti-Gram+, Anti-Gram-, Antimicrobial
668	956	DRAMP03753	Amphipathic peptide CT1 (StCT1; Non-disulfide-bridged peptide 5,	Antibacterial, Anti-Gram+, Anti-Gram-, Antimicrobial
669	957	DRAMP03754	Amphipathic peptide CT2 (StCT2; Non-disulfide-bridged peptide 5,	Antibacterial, Anti-Gram+, Anti-Gram-, Antimicrobial
670	958	DRAMP03774	UyCT1 (Arthropods, animals)	Antibacterial, Anti-Gram+, Anti-Gram-, Antimicrobial
671	960	DRAMP03776	UyCT3 (Arthropods, animals)	Antibacterial, Anti-Gram+, Anti-Gram-, Antimicrobial
672	961	DRAMP03777	UyCT5 (Arthropods, animals)	Antibacterial, Anti-Gram+, Anti-Gram-, Antimicrobial
673	963	DRAMP03814	D16W (GGN4 analogue peptide with single substitution)	Antibacterial, Anti-Gram+, Anti-Gram-, Antimicrobial
674	964	DRAMP03815	D16W-N23 (single amino acid substitution)	Antibacterial, Anti-Gram+, Anti-Gram-, Antimicrobial
675	965	DRAMP03816	D16F-N23 (single amino acid substitution)	Antibacterial, Anti-Gram+, Anti-Gram-, Antimicrobial
676	966	DRAMP03823	Dermaseptin derivative K4-S4-(1-13)	Antibacterial, Anti-Gram+, Anti-Gram-, Antimicrobial
677	967	DRAMP03824	CNBr-cleaved lactoferricin Subfragment 1	Antibacterial, Anti-Gram+, Anti-Gram-, Antimicrobial
678	968	DRAMP03825	CNBr-cleaved lactoferricin Subfragment 2	Antibacterial, Anti-Gram+, Anti-Gram-, Antimicrobial
679	969	DRAMP03826	Ovispirin-1 (OV-1; N-terminal 18 amino acids of SMAP-29)	Antibacterial, Cytotoxicity, Anti-Gram+, Anti-Gram-, Antimicrobial
680	970	DRAMP03827	Novispirin G-10 (mutation of Ovispirin-1)	Antibacterial, Cytotoxicity, Anti-Gram+, Anti-Gram-, Antimicrobial
681	971	DRAMP03828	Novispirin T-7 (mutation of Ovispirin-1)	Antibacterial, Cytotoxicity, Anti-Gram+, Anti-Gram-, Antimicrobial
682	973	DRAMP03830	Palustrin-2ISb + 3aa	Antibacterial, Anti-Gram+, Anti-Gram-, Antimicrobial
683	974	DRAMP03831	Palustrin-2ISb-des-C7	Antibacterial, Antifungal, Anti-Gram+, Anti-Gram-, Antimicrobial
684	975	DRAMP03832	Palustrin-2ISb-des-C7-4D	Antibacterial, Antifungal, Anti-Gram+, Anti-Gram-, Antimicrobial
685	976	DRAMP03833	Palustrin-2ISb-des-C7-12N	Antibacterial, Antifungal, Anti-Gram+, Anti-Gram-, Antimicrobial
686	977	DRAMP03834	Palustrin-2ISb-des-C7-23,29S	Antibacterial, Antifungal, Anti-Gram+, Anti-Gram-, Antimicrobial
687	979	DRAMP03852	G1 (Bac2A variant through single amino acid substitution)	Antibacterial, Antifungal, Anti-Gram+, Anti-Gram-, Antimicrobial
688	980	DRAMP03853	G2 (Bac2A variant through single amino acid substitution)	Antibacterial, Antifungal, Anti-Gram+, Anti-Gram-, Antimicrobial
689	981	DRAMP03854	R2 (Bac2A variant through single amino acid substitution)	Antibacterial, Antifungal, Anti-Gram+, Anti-Gram-, Antimicrobial

S.no.	PepID	DRAMP_ID	Name of the AMP	Activity
690	982	DRAMP03855	R3 (Bac2A variant through single amino acid substitution)	Antibacterial, Antifungal, Anti-Gram+, Anti-Gram-, Antimicrobial
691	983	DRAMP03856	W3 (Bac2A variant through single amino acid substitution)	Antibacterial, Antifungal, Anti-Gram+, Anti-Gram-, Antimicrobial
692	984	DRAMP03857	R5 (Bac2A variant through single amino acid substitution)	Antibacterial, Antifungal, Anti-Gram+, Anti-Gram-, Antimicrobial
693	985	DRAMP03858	K7 (Bac2A variant through single amino acid substitution)	Antibacterial, Antifungal, Anti-Gram+, Anti-Gram-, Antimicrobial
694	986	DRAMP03859	W10 (Bac2A variant through single amino acid substitution)	Antibacterial, Antifungal, Anti-Gram+, Anti-Gram-, Antimicrobial
695	987	DRAMP03860	R11 (Bac2A variant through single amino acid substitution)	Antibacterial, Antifungal, Anti-Gram+, Anti-Gram-, Antimicrobial
696	988	DRAMP03861	G12 (Bac2A variant through single amino acid substitution)	Antibacterial, Antifungal, Anti-Gram+, Anti-Gram-, Antimicrobial
697	989	DRAMP03862	Sub2 (Bac2A variant through two amino acids substitution)	Antibacterial, Antifungal, Anti-Gram+, Anti-Gram-, Antimicrobial
698	990	DRAMP03863	Sub3 (Bac2A variant through three amino acids substitution)	Antibacterial, Antifungal, Anti-Gram+, Anti-Gram-, Antimicrobial
699	991	DRAMP03864	Sub5 (Bac2A variant through five amino acids substitution)	Antibacterial, Antifungal, Anti-Gram+, Anti-Gram-, Antimicrobial
700	992	DRAMP03865	Sub6 (Bac2A variant through six amino acids substitution)	Antibacterial, Antifungal, Anti-Gram+, Anti-Gram-, Antimicrobial
701	993	DRAMP03866	Bac8a (Bac2A variant)	Antibacterial, Antifungal, Anti-Gram+, Anti-Gram-, Antimicrobial
702	994	DRAMP03867	Bac8b (Bac2A variant)	Antibacterial, Antifungal, Anti-Gram+, Anti-Gram-, Antimicrobial
703	995	DRAMP03868	Bac8c (Bac2A variant)	Antibacterial, Antifungal, Anti-Gram+, Anti-Gram-, Antimicrobial
704	996	DRAMP03869	Bac8d (Bac2A variant)	Antibacterial, Antifungal, Anti-Gram+, Anti-Gram-, Antimicrobial
705	997	DRAMP03870	Bac2A (a linear variant of bovine dodecapeptide)	Antibacterial, Antifungal, Anti-Gram+, Anti-Gram-, Antimicrobial
706	998	DRAMP03871	cLf 20-29 (fragment of caprine lalctoferricin, residues 20-29)	Antibacterial, Anti-Gram+, Anti-Gram-, Antimicrobial
707	999	DRAMP03875	bLf 20-29 (fragment of bovine lactoferricin, residues 20-29)	Antibacterial, Anti-Gram+, Anti-Gram-, Antimicrobial
708	1000	DRAMP03876	LFB-RW (derivative of bovine lactoferrin with residues substitution)	Antibacterial, Anti-Gram+, Anti-Gram-, Antimicrobial
709	1001	DRAMP03877	LFB-KW (derivative of bovine lactoferrin with residues substitution)	Antibacterial, Anti-Gram+, Anti-Gram-, Antimicrobial
710	1002	DRAMP03878	LFB-Rwa (derivative of bovine lactoferrin with residues substitution)	Antibacterial, Anti-Gram+, Anti-Gram-, Antimicrobial
711	1003	DRAMP03879	LFB-RF (derivative of bovine lactoferrin with residues substitution)	Antibacterial, Anti-Gram+, Anti-Gram-, Antimicrobial
712	1004	DRAMP03880	LFB-RI (derivative of bovine lactoferrin with residues substitution)	Antibacterial, Anti-Gram+, Anti-Gram-, Antimicrobial
713	1005	DRAMP03881	LFB-6RW (derivative of bovine lactoferrin with residues substitution)	Antibacterial, Anti-Gram+, Anti-Gram-, Antimicrobial
714	1006	DRAMP03882	LFC (fragment of mature caprine lactoferrin, residues 17 to 31)	Antibacterial, Anti-Gram+, Anti-Gram-, Antimicrobial
715	1007	DRAMP03883	LFH W8 (tryptophan-modified human lactoferricin derivative)	Antibacterial, Anti-Gram+, Anti-Gram-, Antimicrobial
716	1008	DRAMP03884	LFC W8 (tryptophan-modified caprine lactoferricin derivative)	Antibacterial, Anti-Gram+, Anti-Gram-, Antimicrobial
717	1009	DRAMP03885	LFP W8 (tryptophan-modified porcine lactoferricin derivative)	Antibacterial, Anti-Gram+, Anti-Gram-, Antimicrobial
718	1010	DRAMP03886	LFB (fragment of bovine lalctoferricin, residues 17 to 31)	Antibacterial, Anti-Gram+, Anti-Gram-, Antimicrobial
719	1011	DRAMP03887	LFB A1 (derivative of LFB, residue substitution with alanine at	Antibacterial, Anti-Gram+, Anti-Gram-, Antimicrobial
720	1012	DRAMP03888	LFB A2 (derivative of LFB, residue substitution with alanine at	Antibacterial, Anti-Gram+, Anti-Gram-, Antimicrobial
721	1013	DRAMP03889	LFB A3 (derivative of LFB, residue substitution with alanine at	Antibacterial, Anti-Gram+, Anti-Gram-, Antimicrobial
722	1014	DRAMP03890	LFB A4 (derivative of LFB, residue substitution with alanine at	Antibacterial, Anti-Gram+, Anti-Gram-, Antimicrobial
723	1015	DRAMP03891	LFB A5 (derivative of LFB, residue substitution with alanine at	Antibacterial, Anti-Gram+, Anti-Gram-, Antimicrobial
724	1016	DRAMP03892	LFB A7 (derivative of LFB, residue substitution with alanine at	Antibacterial, Anti-Gram+, Anti-Gram-, Antimicrobial
725	1017	DRAMP03893	LFB A9 (derivative of LFB, residue substitution with alanine at	Antibacterial, Anti-Gram+, Anti-Gram-, Antimicrobial
726	1018	DRAMP03894	LFB A10 (derivative of LFB, residue substitution with alanine at	Antibacterial, Anti-Gram+, Anti-Gram-, Antimicrobial
727	1019	DRAMP03895	LFB A11 (derivative of LFB, residue substitution with alanine at	Antibacterial, Anti-Gram+, Anti-Gram-, Antimicrobial
728	1020	DRAMP03896	LFB A12 (derivative of LFB, residue substitution with alanine at	Antibacterial, Anti-Gram+, Anti-Gram-, Antimicrobial
729	1021	DRAMP03897	LFB A13 (derivative of LFB, residue substitution with alanine at	Antibacterial, Anti-Gram+, Anti-Gram-, Antimicrobial
730	1022	DRAMP03898	LFB A14 (derivative of LFB, residue substitution with alanine at	Antibacterial, Anti-Gram+, Anti-Gram-, Antimicrobial
731	1025	DRAMP03901	LFM R1 W8 (LFM W8 derivative with residues substitution)	Antibacterial, Anti-Gram+, Anti-Gram-, Antimicrobial
732	1027	DRAMP03903	LFM A1 R9 W8 (LFM W8 derivative with residues substitution)	Antibacterial, Anti-Gram+, Anti-Gram-, Antimicrobial
733	1028	DRAMP03904	LFM A9 R1 W8 (LFM W8 derivative with residues substitution)	Antibacterial, Anti-Gram+, Anti-Gram-, Antimicrobial
734	1029	DRAMP03905	LFM R1,9 W8 (LFM W8 derivative with residues substitution)	Antibacterial, Anti-Gram+, Anti-Gram-, Antimicrobial
735	1032	DRAMP03908	LFM R1 W8 Y13 (LFM W8 derivative with residues substitution)	Antibacterial, Anti-Gram+, Anti-Gram-, Antimicrobial
736	1034	DRAMP03910	LFM A1 R9 W8 Y13 (LFM W8 derivative with residues substitution)	Antibacterial, Anti-Gram+, Anti-Gram-, Antimicrobial
737	1035	DRAMP03911	LFM A9 R1 W8 Y13 (LFM W8 derivative with residues substitution)	Antibacterial, Anti-Gram+, Anti-Gram-, Antimicrobial
738	1036	DRAMP03912	LFM R1,9 W8 Y13 (LFM W8 derivative with residues substitution)	Antibacterial, Anti-Gram+, Anti-Gram-, Antimicrobial
739	1037	DRAMP03920	Cecropin A (1-8)-melittin (1-13)hybrid peptide	Antibacterial, Anti-Gram+, Anti-Gram-, Antimicrobial
740	1038	DRAMP03921	Cecropin A (1-8)-melittin (1-18)hybrid peptide	Antibacterial, Anti-Gram+, Anti-Gram-, Antimicrobial
741	1039	DRAMP03922	Cecropin A (1-8)-melittin (1-12)hybrid peptide	Antibacterial, Anti-Gram+, Anti-Gram-, Antimicrobial
742	1040	DRAMP03923	Cecropin A (1-8)-melittin (1-10)hybrid peptide	Antibacterial, Anti-Gram+, Anti-Gram-, Antimicrobial

S.no.	PepID	DRAMP_ID	Name of the AMP	Activity
743	1041	DRAMP03924	Cecropin A (1-7)-melittin (1-8)hybrid peptide	Antibacterial, Anti-Gram+, Anti-Gram-, Antimicrobial
744	1042	DRAMP03925	Cecropin A (1-7)-melittin (3-10)hybrid peptide	Antibacterial, Anti-Gram+, Anti-Gram-, Antimicrobial
745	1043	DRAMP03927	Cecropin A (1-7)-melittin (2-9)hybrid peptide	Antibacterial, Anti-Gram+, Anti-Gram-, Antimicrobial
746	1044	DRAMP03928	Cecropin A (1-7)-melittin (4-11)hybrid peptide (CAM)	Antibacterial, Anti-Gram+, Anti-Gram-, Antimicrobial
747	1045	DRAMP03929	Cecropin A (1-7)-melittin (5-12)hybrid peptide	Antibacterial, Anti-Gram+, Anti-Gram-, Antimicrobial
748	1046	DRAMP03930	Cecropin A (1-7)-melittin (6-13)hybrid peptide	Antibacterial, Antifungal, Anti-Gram+, Anti-Gram-, Antimicrobial
749	1048	DRAMP03933	I14M (truncated isoform of thanatin, residue 8-21)	Antibacterial, Antifungal, Anti-Gram+, Anti-Gram-, Antimicrobial
750	1050	DRAMP03935	V16M (truncated isoform of thanatin, residue 6-21)	Antibacterial, Antifungal, Anti-Gram+, Anti-Gram-, Antimicrobial
751	1051	DRAMP03936	K18M (truncated isoform of thanatin, residue 4-21)	Antibacterial, Antifungal, Anti-Gram+, Anti-Gram-, Antimicrobial
752	1055	DRAMP03945	Del 1-4 (Ranalexin analog)	Antibacterial, Anti-Gram+, Anti-Gram-, Antimicrobial
753	1056	DRAMP03947	Del 1-2 (Ranalexin analog)	Antibacterial, Anti-Gram+, Anti-Gram-, Antimicrobial
754	1057	DRAMP03948	Del 1 (Ranalexin analog)	Antibacterial, Anti-Gram+, Anti-Gram-, Antimicrobial
755	1058	DRAMP03949	Del 20 (Ranalexin analog)	Antibacterial, Anti-Gram+, Anti-Gram-, Antimicrobial
756	1065	DRAMP03967	P18 (Cecropin A(1-8)-Magainin 2(1-12) hybrid peptide analogue)	Antibacterial, Antitumour, Anti-Gram+, Anti-Gram-, Antimicrobial
757	1066	DRAMP03968	[L9]-P18 (analog of P18)	Antibacterial, Antitumour, Anti-Gram+, Anti-Gram-, Antimicrobial
758	1067	DRAMP03969	[S9]-P18 (analog of P18)	Antibacterial, Antitumour, Anti-Gram+, Anti-Gram-, Antimicrobial
759	1068	DRAMP03970	N-1 (analog of P18)	Antibacterial, Antitumour, Anti-Gram+, Anti-Gram-, Antimicrobial
760	1069	DRAMP03971	N-2 (analog of P18)	Antibacterial, Antitumour, Anti-Gram+, Anti-Gram-, Antimicrobial
761	1070	DRAMP03972	N-3 (analog of P18)	Antibacterial, Antitumour, Anti-Gram+, Anti-Gram-, Antimicrobial
762	1071	DRAMP03973	N-4 (analog of P18)	Antibacterial, Antitumour, Anti-Gram+, Anti-Gram-, Antimicrobial
763	1072	DRAMP03974	N-5 (analog of P18)	Antibacterial, Antitumour, Anti-Gram+, Anti-Gram-, Antimicrobial
764	1073	DRAMP03975	N-3L (analog of P18)	Antibacterial, Antitumour, Anti-Gram+, Anti-Gram-, Antimicrobial
765	1074	DRAMP03976	N-4L (analog of P18)	Antibacterial, Antitumour, Anti-Gram+, Anti-Gram-, Antimicrobial
766	1075	DRAMP03977	N-5L (analog of P18)	Antibacterial, Antitumour, Anti-Gram+, Anti-Gram-, Antimicrobial
767	1076	DRAMP03978	C-1 (analog of P18)	Antibacterial, Antitumour, Anti-Gram+, Anti-Gram-, Antimicrobial
768	1077	DRAMP03979	C-2 (analog of P18)	Antibacterial, Antitumour, Anti-Gram+, Anti-Gram-, Antimicrobial
769	1078	DRAMP03980	C-3 (analog of P18)	Antibacterial, Antitumour, Anti-Gram+, Anti-Gram-, Antimicrobial
770	1079	DRAMP03981	C-4 (analog of P18)	Antibacterial, Antitumour, Anti-Gram+, Anti-Gram-, Antimicrobial
771	1080	DRAMP03982	C-5 (analog of P18)	Antibacterial, Antitumour, Anti-Gram+, Anti-Gram-, Antimicrobial
772	1081	DRAMP03983	C-6 (analog of P18)	Antibacterial, Antitumour, Anti-Gram+, Anti-Gram-, Antimicrobial
773	1082	DRAMP03984	C-7 (analog of P18)	Antibacterial, Antitumour, Anti-Gram+, Anti-Gram-, Antimicrobial
774	1083	DRAMP03985	C-8 (analog of P18)	Antibacterial, Antitumour, Anti-Gram+, Anti-Gram-, Antimicrobial
775	1084	DRAMP03986	C-9 (analog of P18)	Antibacterial, Antitumour, Anti-Gram+, Anti-Gram-, Antimicrobial
776	1085	DRAMP03987	C-10 (analog of P18)	Antibacterial, Antitumour, Anti-Gram+, Anti-Gram-, Antimicrobial
777	1088	DRAMP03990	L4K3W4 (LlKmWn model peptide)	Antibacterial, Anti-Gram+, Anti-Gram-, Antimicrobial
778	1090	DRAMP03992	L5K3W5 (LlKmWn model peptide)	Antibacterial, Anti-Gram+, Anti-Gram-, Antimicrobial
779	1091	DRAMP03993	L5K5W6 (LlKmWn model peptide)	Antibacterial, Anti-Gram+, Anti-Gram-, Antimicrobial
780	1092	DRAMP03994	L6K4W6 (LlKmWn model peptide)	Antibacterial, Anti-Gram+, Anti-Gram-, Antimicrobial
781	1093	DRAMP03995	L7K3W6 (LlKmWn model peptide)	Antibacterial, Anti-Gram+, Anti-Gram-, Antimicrobial
782	1096	DRAMP03999	[A6]-IsCT (Mutant: W6A; IsCT analog)	Antibacterial, Anti-Gram+, Anti-Gram-, Antimicrobial
783	1097	DRAMP04000	[L6]-IsCT (Mutant: W6L; IsCT analog)	Antibacterial, Anti-Gram+, Anti-Gram-, Antimicrobial
784	1098	DRAMP04001	[K7]-IsCT (Mutant: E7K; IsCT analog)	Antibacterial, Anti-Gram+, Anti-Gram-, Antimicrobial
785	1099	DRAMP04002	[L6, K11]-IsCT (IsCT analog through amino acids substitution)	Antibacterial, Anti-Gram+, Anti-Gram-, Antimicrobial
786	1100	DRAMP04003	[K7, P8, K11]-IsCT (IsCT analog through amino acids substitution)	Antibacterial, Anti-Gram+, Anti-Gram-, Antimicrobial
787	1101	DRAMP04004	Gramicidin analogue ([Scr2]-GS)	Antibacterial, Anti-Gram+, Anti-Gram-, Antimicrobial
788	1102	DRAMP04005	Gramicidin analogue ([Ser2,2']-GS)	Antibacterial, Anti-Gram+, Anti-Gram-, Antimicrobial
789	1103	DRAMP04011	Plasticin PD36 KF (analog of PD36)	Antibacterial, Anti-Gram+, Anti-Gram-, Antimicrobial
790	1104	DRAMP04012	Plasticin PD36 K (analog of PD36)	Antibacterial, Anti-Gram+, Anti-Gram-, Antimicrobial
791	1105	DRAMP04013	Plasticin ANC KF (analog of natural peptide ANC)	Antibacterial, Anti-Gram+, Anti-Gram-, Antimicrobial
792	1109	DRAMP04017	LL-23V9 (LL-23 variants)	Antibacterial, Anti-Gram+, Anti-Gram-, Antimicrobial
793	1110	DRAMP04019	Bac014 (Scrambled Variants of Bac2A)	Antibacterial, Antifungal, Anti-Gram+, Anti-Gram-, Antimicrobial
794	1111	DRAMP04020	Bac020 (Scrambled Variants of Bac2A)	Antibacterial, Antifungal, Anti-Gram+, Anti-Gram-, Antimicrobial
795	1112	DRAMP04021	Bac034 (Scrambled Variants of Bac2A)	Antibacterial, Antifungal, Anti-Gram+, Anti-Gram-, Antimicrobial

S.no.	PepID	DRAMP_ID	Name of the AMP	Activity
796	1113	DRAMP04022	F3 (single amino acid substitution of Bac034, which is a scrambled	Antibacterial, Antifungal, Anti-Gram+, Anti-Gram-, Antimicrobial
797	1114	DRAMP04023	W3 (single amino acid substitution of Bac034, which is a scrambled	Antibacterial, Antifungal, Anti-Gram+, Anti-Gram-, Antimicrobial
798	1115	DRAMP04024	W4 (single amino acid substitution of Bac034, which is a scrambled	Antibacterial, Antifungal, Anti-Gram+, Anti-Gram-, Antimicrobial
799	1116	DRAMP04025	R10 (single amino acid substitution of Bac034, which is a scrambled	Antibacterial, Antifungal, Anti-Gram+, Anti-Gram-, Antimicrobial
800	1117	DRAMP04026	K12 (single amino acid substitution of Bac034, which is a scrambled	Antibacterial, Antifungal, Anti-Gram+, Anti-Gram-, Antimicrobial
801	1118	DRAMP04027	opt1 (multiple amino acid substitution of Bac034, which is a	Antibacterial, Antifungal, Anti-Gram+, Anti-Gram-, Antimicrobial
802	1119	DRAMP04028	opt2 (multiple amino acid substitution of Bac034, which is a	Antibacterial, Antifungal, Anti-Gram+, Anti-Gram-, Antimicrobial
803	1120	DRAMP04029	opt3 (multiple amino acid substitution of Bac034, which is a	Antibacterial, Antifungal, Anti-Gram+, Anti-Gram-, Antimicrobial
804	1121	DRAMP04030	opt4 (multiple amino acid substitution of Bac034, which is a	Antibacterial, Antifungal, Anti-Gram+, Anti-Gram-, Antimicrobial
805	1122	DRAMP04031	opt5 (multiple amino acid substitution of Bac034, which is a	Antibacterial, Antifungal, Anti-Gram+, Anti-Gram-, Antimicrobial
806	1123	DRAMP04032	Modified defensin	Antibacterial, Anti-Gram+, Anti-Gram-, Antimicrobial
807	1124	DRAMP04033	Modified defensin	Antibacterial, Anti-Gram+, Anti-Gram-, Antimicrobial
808	1125	DRAMP04034	Modified defensin	Antibacterial, Anti-Gram+, Anti-Gram-, Antimicrobial
809	1126	DRAMP04035	Modified defensin	Antibacterial, Anti-Gram+, Anti-Gram-, Antimicrobial
810	1127	DRAMP04036	Modified defensin	Antibacterial, Anti-Gram+, Anti-Gram-, Antimicrobial
811	1128	DRAMP04048	BacR (cyclic derivative of bactenecin)	Antibacterial, Anti-Gram+, Anti-Gram-, Antimicrobial
812	1129	DRAMP04049	BacP3R (cyclic derivative of bactenecin)	Antibacterial, Anti-Gram+, Anti-Gram-, Antimicrobial
813	1130	DRAMP04050	BacP3R-V (cyclic derivative of bactenecin)	Antibacterial, Anti-Gram+, Anti-Gram-, Antimicrobial
814	1131	DRAMP04051	Bac2I-NH2 (cyclic derivative of bactenecin)	Antibacterial, Anti-Gram+, Anti-Gram-, Antimicrobial
815	1132	DRAMP04052	BacP2R-NH2 (cyclic derivative of bactenecin)	Antibacterial, Anti-Gram+, Anti-Gram-, Antimicrobial
816	1133	DRAMP04053	BacP1 (cyclic derivative of bactenecin)	Antibacterial, Anti-Gram+, Anti-Gram-, Antimicrobial
817	1134	DRAMP04054	BacW (cyclic derivative of bactenecin)	Antibacterial, Anti-Gram+, Anti-Gram-, Antimicrobial
818	1135	DRAMP04055	BacW2R (cyclic derivative of bactenecin)	Antibacterial, Anti-Gram+, Anti-Gram-, Antimicrobial
819	1136	DRAMP04056	Lin Bac2S-NH2 (linear derivative of bactenecin)	Antibacterial, Anti-Gram+, Anti-Gram-, Antimicrobial
820	1137	DRAMP04057	Lin BacS-NH2 (linear derivative of bactenecin)	Antibacterial, Anti-Gram+, Anti-Gram-, Antimicrobial
821	1153	DRAMP04075	Antimicrobial peptide HP (2-20)	Antibacterial, Antifungal, Anti-Gram+, Anti-Gram-, Antimicrobial
822	1154	DRAMP04076	Anal 1 (antimicrobial peptide HP (2-20)analogue)	Antibacterial, Antifungal, Anti-Gram+, Anti-Gram-, Antimicrobial
823	1155	DRAMP04077	Anal 2 (antimicrobial peptide HP (2-20)analogue)	Antibacterial, Antifungal, Anti-Gram+, Anti-Gram-, Antimicrobial
824	1156	DRAMP04078	Anal 3 (antimicrobial peptide HP (2-20)analogue)	Antibacterial, Antifungal, Anti-Gram+, Anti-Gram-, Antimicrobial
825	1157	DRAMP04079	Anal 4 (antimicrobial peptide HP (2-20)analogue)	Antibacterial, Antifungal, Anti-Gram+, Anti-Gram-, Antimicrobial
826	1158	DRAMP04080	Anal 5 (antimicrobial peptide HP (2-20)analogue)	Antibacterial, Antifungal, Anti-Gram+, Anti-Gram-, Antimicrobial
827	1159	DRAMP04081	Anal 6 (antimicrobial peptide HP (2-20)analogue)	Antibacterial, Antifungal, Anti-Gram+, Anti-Gram-, Antimicrobial
828	1160	DRAMP04082	Anal 7 (antimicrobial peptide HP (2-20)analogue)	Antibacterial, Antifungal, Anti-Gram+, Anti-Gram-, Antimicrobial
829	1161	DRAMP04083	D-amino-acid pexiganan (MSI-214)	Antibacterial, Anti-Gram+, Anti-Gram-, Antimicrobial
830	1162	DRAMP04095	Cupiennin-1D (spiders, Arthropods, animals)	Antibacterial, Anti-Gram+, Anti-Gram-, Antimicrobial
831	1163	DRAMP04096	2IQ2	Antibacterial, Antifungal, Anti-Gram+, Anti-Gram-, Antimicrobial
832	1164	DRAMP04097	2IQ3	Antibacterial, Antifungal, Anti-Gram+, Anti-Gram-, Antimicrobial
833	1165	DRAMP04098	3IQ1	Antibacterial, Antifungal, Anti-Gram+, Anti-Gram-, Antimicrobial
834	1166	DRAMP04099	3IQ2	Antibacterial, Antifungal, Anti-Gram+, Anti-Gram-, Antimicrobial
835	1167	DRAMP04100	3IQ3	Antibacterial, Antifungal, Anti-Gram+, Anti-Gram-, Antimicrobial
836	1168	DRAMP04101	3IQ4	Antibacterial, Antifungal, Anti-Gram+, Anti-Gram-, Antimicrobial
837	1180	DRAMP04115	K11 (derivative of CP-P)	Antibacterial, Anti-Gram+, Anti-Gram-, Antimicrobial
838	1186	DRAMP04123	D0-NH2	Antibacterial, Anti-Gram+, Anti-Gram-, Antimicrobial
839	1187	DRAMP04124	D1-NH2	Antibacterial, Anti-Gram+, Anti-Gram-, Antimicrobial
840	1188	DRAMP04125	D2-NH2	Antibacterial, Anti-Gram+, Anti-Gram-, Antimicrobial
841	1189	DRAMP04126	D3-NH2	Antibacterial, Anti-Gram+, Anti-Gram-, Antimicrobial
842	1190	DRAMP04127	D4-NH2	Antibacterial, Anti-Gram+, Anti-Gram-, Antimicrobial
843	1191	DRAMP04128	D5-NH2	Antibacterial, Anti-Gram+, Anti-Gram-, Antimicrobial
844	1192	DRAMP04129	D6-NH2	Antibacterial, Anti-Gram+, Anti-Gram-, Antimicrobial
845	1193	DRAMP04136	LRR-1	Antibacterial, Anti-Gram+, Anti-Gram-, Antimicrobial
846	1194	DRAMP04137	LRR-2	Antibacterial, Anti-Gram+, Anti-Gram-, Antimicrobial
847	1205	DRAMP04159	LR2 (homologue of Pc-CATH1)	Antibacterial, Antifungal, Anti-Gram+, Anti-Gram-, Antimicrobial
848	1206	DRAMP04160	LR3 (homologue of Pc-CATH1)	Antibacterial, Antifungal, Anti-Gram+, Anti-Gram-, Antimicrobial

S.no.	PepID	DRAMP_ID	Name of the AMP	Activity
849	1207	DRAMP04161	LR4 (homologue of Pc-CATH1)	Antibacterial, Antifungal, Anti-Gram+, Anti-Gram-, Antimicrobial
850	1208	DRAMP04162	LR5 (homologue of Pc-CATH1)	Antibacterial, Antifungal, Anti-Gram+, Anti-Gram-, Antimicrobial
851	1209	DRAMP04163	LR6 (homologue of Pc-CATH1)	Antibacterial, Antifungal, Anti-Gram+, Anti-Gram-, Antimicrobial
852	1210	DRAMP04164	LR7 (homologue of Pc-CATH1)	Antibacterial, Antifungal, Anti-Gram+, Anti-Gram-, Antimicrobial
853	1211	DRAMP04165	LR8 (homologue of Pc-CATH1)	Antibacterial, Antifungal, Anti-Gram+, Anti-Gram-, Antimicrobial
854	1212	DRAMP04166	LR9 (homologue of Pc-CATH1)	Antibacterial, Antifungal, Anti-Gram+, Anti-Gram-, Antimicrobial
855	1213	DRAMP04167	LR10 (homologue of Pc-CATH1)	Antifungal, Anti-Gram+, Anti-Gram-, Antimicrobial
856	1214	DRAMP04168	LR11 (homologue of Pc-CATH1)	Antifungal, Anti-Gram+, Anti-Gram-, Antimicrobial
857	1215	DRAMP04169	LR13 (homologue of Pc-CATH1)	Antifungal, Anti-Gram+, Anti-Gram-, Antimicrobial
858	1216	DRAMP04170	LR15 (homologue of Pc-CATH1)	Antifungal, Anti-Gram+, Anti-Gram-, Antimicrobial
859	1217	DRAMP04171	LR16 (homologue of Pc-CATH1)	Antifungal, Anti-Gram+, Anti-Gram-, Antimicrobial
860	1218	DRAMP04174	L2K3W2 (LlKmW2 model peptides)	Antibacterial, Anti-Gram+, Anti-Gram-, Antimicrobial
861	1219	DRAMP04175	L3K2W2 (LlKmW2 model peptides)	Antibacterial, Anti-Gram+, Anti-Gram-, Antimicrobial
862	1220	DRAMP04176	L2K5W2 (LlKmW2 model peptides)	Antibacterial, Anti-Gram+, Anti-Gram-, Antimicrobial
863	1221	DRAMP04177	L3K4W2 (LlKmW2 model peptides)	Antibacterial, Anti-Gram+, Anti-Gram-, Antimicrobial
864	1222	DRAMP04178	L4K3W2 (LlKmW2 model peptides)	Antibacterial, Anti-Gram+, Anti-Gram-, Antimicrobial
865	1223	DRAMP04179	L5K2W2 (LlKmW2 model peptides)	Antibacterial, Anti-Gram+, Anti-Gram-, Antimicrobial
866	1224	DRAMP04180	L3K6W2 (LlKmW2 model peptides)	Antibacterial, Anti-Gram+, Anti-Gram-, Antimicrobial
867	1225	DRAMP04181	L4K5W2 (LlKmW2 model peptides)	Antibacterial, Anti-Gram+, Anti-Gram-, Antimicrobial
868	1226	DRAMP04182	L5K4W2 (LlKmW2 model peptides)	Antibacterial, Anti-Gram+, Anti-Gram-, Antimicrobial
869	1227	DRAMP04183	L6K3W2 (LlKmW2 model peptides)	Antibacterial, Anti-Gram+, Anti-Gram-, Antimicrobial
870	1229	DRAMP04185	DFTamP1-p	Antibacterial, Anti-Gram+, Anti-Gram-, Antimicrobial
871	1230	DRAMP04186	L5K5W1 (L5K5Wn model peptide)	Antibacterial, Anti-Gram+, Anti-Gram-, Antimicrobial
872	1231	DRAMP04187	L5K5W2 (L5K5Wn model peptide)	Antibacterial, Anti-Gram+, Anti-Gram-, Antimicrobial
873	1232	DRAMP04188	L5K5W3 (L5K5Wn model peptide)	Antibacterial, Anti-Gram+, Anti-Gram-, Antimicrobial
874	1233	DRAMP04189	L5K5W4 (L5K5Wn model peptide)	Antibacterial, Anti-Gram+, Anti-Gram-, Antimicrobial
875	1234	DRAMP04190	L5K5W5 (L5K5Wn model peptide)	Antibacterial, Anti-Gram+, Anti-Gram-, Antimicrobial
876	1236	DRAMP04192	L5K5W7 (L5K5Wn model peptide)	Antibacterial, Anti-Gram+, Anti-Gram-, Antimicrobial
877	1237	DRAMP04193	L5K5W8 (L5K5Wn model peptide)	Antibacterial, Anti-Gram+, Anti-Gram-, Antimicrobial
878	1238	DRAMP04194	L5K5W9 (L5K5Wn model peptide)	Antibacterial, Anti-Gram+, Anti-Gram-, Antimicrobial
879	1239	DRAMP04195	L5K5W10 (L5K5Wn model peptide)	Antibacterial, Anti-Gram+, Anti-Gram-, Antimicrobial
880	1240	DRAMP04196	L5K5W11 (L5K5Wn model peptide)	Antibacterial, Anti-Gram+, Anti-Gram-, Antimicrobial
881	1245	DRAMP04240	Synthetic 1	Antibacterial, Anti-Gram+, Anti-Gram-, Antimicrobial
882	1246	DRAMP04241	Synthetic 2	Antibacterial, Anti-Gram+, Anti-Gram-, Antimicrobial
883	1247	DRAMP04242	Synthetic 3	Antibacterial, Anti-Gram+, Anti-Gram-, Antimicrobial
884	1248	DRAMP04243	Synthetic 4	Antibacterial, Anti-Gram+, Anti-Gram-, Antimicrobial
885	1249	DRAMP04244	Synthetic 5	Antibacterial, Anti-Gram+, Anti-Gram-, Antimicrobial
886	1253	DRAMP04359	PDD-A-1 (PDD-A analog)	Antibacterial, Anti-Gram+, Anti-Gram-, Antimicrobial
887	1254	DRAMP04360	PDD-A-2 (PDD-A analog)	Antibacterial, Anti-Gram+, Anti-Gram-, Antimicrobial
888	1255	DRAMP04361	PDD-A-3 (PDD-A analog)	Antibacterial, Anti-Gram+, Anti-Gram-, Antimicrobial
889	1256	DRAMP04362	PDD-A-4 (PDD-A analog)	Antibacterial, Anti-Gram+, Anti-Gram-, Antimicrobial
890	1257	DRAMP04363	PDD-A-5 (PDD-A analog)	Antibacterial, Anti-Gram+, Anti-Gram-, Antimicrobial
891	1258	DRAMP04364	PDD-A-6 (PDD-A analog)	Antibacterial, Anti-Gram+, Anti-Gram-, Antimicrobial
892	1259	DRAMP04365	PDD-A-7 (PDD-A analog)	Antibacterial, Anti-Gram+, Anti-Gram-, Antimicrobial
893	1260	DRAMP04367	PDD-A-9 (PDD-A analog)	Antibacterial, Anti-Gram+, Anti-Gram-, Antimicrobial
894	1261	DRAMP04368	PDD-A-10 (PDD-A analog)	Antibacterial, Anti-Gram+, Anti-Gram-, Antimicrobial
895	1262	DRAMP04369	PDD-A-11 (PDD-A analog)	Antibacterial, Anti-Gram+, Anti-Gram-, Antimicrobial
896	1263	DRAMP04370	PDD-A-12 (PDD-A analog)	Antibacterial, Anti-Gram+, Anti-Gram-, Antimicrobial
897	1264	DRAMP04371	PDD-B-1 (PDD-B analog)	Antibacterial, Anti-Gram+, Anti-Gram-, Antimicrobial
898	1265	DRAMP04372	PDD-B-2 (PDD-B analog)	Antibacterial, Anti-Gram+, Anti-Gram-, Antimicrobial
899	1266	DRAMP04373	PDD-B-3 (PDD-B analog)	Antibacterial, Anti-Gram+, Anti-Gram-, Antimicrobial
900	1267	DRAMP04374	PDD-B-4 (PDD-B analog)	Antibacterial, Anti-Gram+, Anti-Gram-, Antimicrobial
901	1268	DRAMP04376	MP-1 (MP analog)	Antibacterial, Anti-Gram+, Anti-Gram-, Antimicrobial

S.no.	PepID	DRAMP_ID	Name of the AMP	Activity
902	1269	DRAMP04377	MP-2 (MP analog)	Antibacterial, Anti-Gram+, Anti-Gram-, Antimicrobial
903	1270	DRAMP04378	MP-5 (MP analog)	Antibacterial, Anti-Gram+, Anti-Gram-, Antimicrobial
904	1271	DRAMP04379	MP-6 (MP analog)	Antibacterial, Anti-Gram+, Anti-Gram-, Antimicrobial
905	1272	DRAMP04380	PMM-1 (PMM analog)	Antibacterial, Anti-Gram+, Anti-Gram-, Antimicrobial
906	1273	DRAMP04381	PMM-2 (PMM analog)	Antibacterial, Anti-Gram+, Anti-Gram-, Antimicrobial
907	1274	DRAMP04382	PMM-3 (PMM analog)	Antibacterial, Anti-Gram+, Anti-Gram-, Antimicrobial
908	1275	DRAMP04383	PMM-4 (PMM analog)	Antibacterial, Anti-Gram+, Anti-Gram-, Antimicrobial
909	1276	DRAMP04385	PMM-6 (PMM analog)	Antibacterial, Anti-Gram+, Anti-Gram-, Antimicrobial
910	1277	DRAMP04386	PMM-7 (PMM analog)	Antibacterial, Anti-Gram+, Anti-Gram-, Antimicrobial
911	1278	DRAMP04387	PMM-8 (PMM analog)	Antibacterial, Anti-Gram+, Anti-Gram-, Antimicrobial
912	1279	DRAMP04389	PMM-10 (PMM analog)	Antibacterial, Anti-Gram+, Anti-Gram-, Antimicrobial
913	1280	DRAMP04390	PMM-11 (PMM analog)	Antibacterial, Anti-Gram+, Anti-Gram-, Antimicrobial
914	1281	DRAMP04391	PMM-12 (PMM analog)	Antibacterial, Anti-Gram+, Anti-Gram-, Antimicrobial
915	1282	DRAMP04392	PMM-13 (PMM analog)	Antibacterial, Anti-Gram+, Anti-Gram-, Antimicrobial
916	1283	DRAMP04393	PMM-14 (PMM analog)	Antibacterial, Anti-Gram+, Anti-Gram-, Antimicrobial
917	1285	DRAMP04542	Polybia-MP-I (insects, vertebrates, animals)	Antibacterial, Anti-Gram+, Anti-Gram-, Antimicrobial
918	1286	DRAMP04543	Polybia-MP-II (insects, vertebrates, animals)	Antibacterial, Cytotoxicity, Anti-Gram+, Anti-Gram-, Antimicrobial
919	1287	DRAMP04544	Polybia-MP-III (insects, vertebrates, animals)	Antibacterial, Cytotoxicity, Anti-Gram+, Anti-Gram-, Antimicrobial
920	1291	DRAMP04640	PGLa-AN2	Antibacterial, Anti-Gram+, Anti-Gram-, Antimicrobial
921	1292	DRAMP04665	Px-cec1	Antibacterial, Antifungal, Anti-Gram+, Anti-Gram-, Antimicrobial
922	1293	DRAMP04670	PBD1-42	Antibacterial, Anti-Gram+, Anti-Gram-, Antimicrobial
923	1294	DRAMP04671	Myticusin-1	Antibacterial, Antifungal, Anti-Gram+, Anti-Gram-, Antimicrobial
924	1295	DRAMP04676	Brevinin-2HS2A	Antibacterial, Antifungal, Anti-Gram+, Anti-Gram-, Antimicrobial
925	1296	DRAMP04677	Brevinin-2HS2B	Antibacterial, Antifungal, Anti-Gram+, Anti-Gram-, Antimicrobial
926	1346	DRAMP00052	Mutacin-2 (Mutacin II mutacin H-29B; Bacteriocin)	Antibacterial, Anti-Gram+, Anti-Gram-, Antimicrobial
927	1354	DRAMP00064	Enterocin 96 (Bacteriocin)	Antibacterial, Anti-Gram+, Anti-Gram-, Antimicrobial
928	1358	DRAMP00070	Laterosporulin (Bacteriocin)	Antibacterial, Anti-Gram+, Anti-Gram-, Antimicrobial
929	1372	DRAMP00085	Bacteriocin	Antibacterial, Anti-Gram+, Anti-Gram-, Antimicrobial
930	1434	DRAMP00169	Enterocin AS-48 (AS-48; Bacteriocin)	Antibacterial, Anti-Gram+, Anti-Gram-, Antimicrobial
931	1438	DRAMP18338	Thiocillin GE37468 (Bacteriocin)	Antibacterial, Anti-Gram+, Anti-Gram-, Antimicrobial
932	1444	DRAMP00182	Thuricin-S (Bacteriocin)	Antibacterial, Anti-Gram+, Anti-Gram-, Antimicrobial
933	1574	DRAMP00341	Antifungal protein ginkbilobin-1 (Ginkbilobin, GNL; Plants)	Antibacterial, Antifungal, Antiviral, Anti-Gram+, Anti-Gram-, Antimicrobial
934	1609	DRAMP00393	Hedyotide B2 (hB2; Uncyclotides; Plants)	Antifungal, Anti-Gram+, Anti-Gram-, Antimicrobial
935	1612	DRAMP00397	Defensin D1 (Ns-D1; Plant defensin)	Antifungal, Anti-Gram+, Anti-Gram-, Antimicrobial
936	1613	DRAMP00398	Defensin D2 (Ns-D2; Plant defensin)	Antifungal, Anti-Gram+, Anti-Gram-, Antimicrobial
937	1617	DRAMP00402	Defensin D1 (So-D1; Antimicrobial peptide D1; Plant defensin)	Antibacterial, Anti-Gram+, Anti-Gram-, Antimicrobial
938	1618	DRAMP00403	Defensin D2 (So-D2; Antimicrobial peptide D2; Plant defensin)	Antibacterial, Antifungal, Anti-Gram+, Anti-Gram-, Antimicrobial
939	1621	DRAMP00406	Defensin D5 (So-D5; Antimicrobial peptide D5; Plant defensin)	Antibacterial, Antifungal, Anti-Gram+, Anti-Gram-, Antimicrobial
940	1622	DRAMP00407	Defensin D6 (So-D6; Antimicrobial peptide D6; Plant defensin)	Antibacterial, Antifungal, Anti-Gram+, Anti-Gram-, Antimicrobial
941	1624	DRAMP00409	Defensin-like protein (Sesquin; Plant defensin)	Antibacterial, Antifungal, Antiviral, Anti-Gram+, Anti-Gram-, Antimicrobial
942	1661	DRAMP00455	Defensin-like protein 2 (Fabatin-2; Plant defensin)	Antibacterial, Anti-Gram+, Anti-Gram-, Antimicrobial
943	1662	DRAMP00456	Defensin-like protein 1 (Fabatin-1; Plant defensin)	Antibacterial, Anti-Gram+, Anti-Gram-, Antimicrobial
944	1952	DRAMP00746	Flower-specific defensin (NaD1; Plant defensin)	Antifungal, Anti-Gram+, Anti-Gram-, Antimicrobial
945	1969	DRAMP00767	ChaC1 (Chassatide C1; Plant defensin)	Antibacterial, Anticancer, Anti-Gram+, Anti-Gram-, Antimicrobial
946	1970	DRAMP00768	ChaC2 (Chassatide C2; Plant defensin)	Antibacterial, Anticancer, Anti-Gram+, Anti-Gram-, Antimicrobial
947	1971	DRAMP00769	ChaC4 (Chassatide C4; Plant defensin)	Antibacterial, Anticancer, Anti-Gram+, Anti-Gram-, Antimicrobial
948	1972	DRAMP00770	ChaC10 (Chassatide C10; Plant defensin)	Antibacterial, Anticancer, Anti-Gram+, Anti-Gram-, Antimicrobial
949	1976	DRAMP18325	delta-lysin I (Bacteriocin)	Antibacterial, Anti-Gram+, Anti-Gram-, Antimicrobial
950	1977	DRAMP00796	Cliotide T2 (cT2; Plant defensin)	Antibacterial, Anticancer, Anti-Gram+, Anti-Gram-, Antimicrobial
951	1978	DRAMP00797	Cliotide T3 (cT3; Plant defensin)	Antibacterial, Anticancer, Anti-Gram+, Anti-Gram-, Antimicrobial
952	2071	DRAMP00937	Tu-AMP1 (Plant defensin)	Antibacterial, Antifungal, Anti-Gram+, Anti-Gram-, Antimicrobial
953	2072	DRAMP00938	Tu-AMP2 (Plant defensin)	Antibacterial, Antifungal, Anti-Gram+, Anti-Gram-, Antimicrobial
954	2077	DRAMP00957	Pp-AMP1 (P. pubescens AMP1; Plant defensin)	Antibacterial, Antifungal, Anti-Gram+, Anti-Gram-, Antimicrobial

S.no.	PepID	DRAMP_ID	Name of the AMP	Activity
955	2078	DRAMP00958	Pp-AMP2 (P. pubescens AMP2; Plant defensin)	Antibacterial, Antifungal, Anti-Gram+, Anti-Gram-, Antimicrobial
956	2081	DRAMP01380	Odorranain-J1 (OdJ1; Frogs, amphibians, animals)	Antimicrobial, Antibacterial, Antifungal, Anti-Gram+, Anti- Gram-,
957	2090	DRAMP00980	Antimicrobial peptide 1a (WAMP-1a; Plant defensin)	Antibacterial, Antifungal, Anti-Gram+, Anti-Gram-, Antimicrobial
958	2091	DRAMP00981	Antimicrobial peptide 1b (WAMP-1b; Plant defensin)	Antibacterial, Antifungal, Anti-Gram+, Anti-Gram-, Antimicrobial
959	2092	DRAMP00982	Fa-AMP1 (Fagopyrum antimicrobial peptide 1; hevein-type; Plant	Antibacterial, Antifungal, Anti-Gram+, Anti-Gram-, Antimicrobial
960	2093	DRAMP00983	Fa-AMP2 (Fagopyrum antimicrobial peptide 2; hevein-type; Plant	Antibacterial, Antifungal, Anti-Gram+, Anti-Gram-, Antimicrobial
961	2107	DRAMP00997	IB-AMP4 (IBAMP4; Basic peptide AMP4; Plants)	Antibacterial, Antifungal, Anti-Gram+, Anti-Gram-, Antimicrobial
962	2108	DRAMP00998	Antimicrobial peptide MBP-1 (Maize Basic Peptide 1; Plant defensin)	Antibacterial, Antifungal, Anti-Gram+, Anti-Gram-, Antimicrobial
963	2119	DRAMP01010	Lunatusin (Plants)	Antibacterial, Antifungal, Antiviral, Anti-Gram+, Anti-Gram-, Antimicrobial
964	2123	DRAMP01015	VaD1 (Plant defensin)	Antibacterial, Antifungal, Anti-Gram+, Anti-Gram-, Antimicrobial
965	2126	DRAMP01022	Cy-AMP1 (Plant defensin)	Antibacterial, Antifungal, Anti-Gram+, Anti-Gram-, Antimicrobial
966	2127	DRAMP01023	Cy-AMP2 (Plant defensin)	Antibacterial, Antifungal, Anti-Gram+, Anti-Gram-, Antimicrobial
967	2128	DRAMP01024	Cy-AMP3 (Plant defensin)	Antibacterial, Antifungal, Anti-Gram+, Anti-Gram-, Antimicrobial
968	2187	DRAMP01100	Bombinin-like peptide 1 (Contains: Bombinin H; toads, amphibians,	Antibacterial, Anti-Gram+, Anti-Gram-, Antimicrobial
969	2217	DRAMP01140	Uperin-2.2 (toads, amphibians, animals)	Antibacterial, Anti-Gram+, Anti-Gram-, Antimicrobial
970	2250	DRAMP01196	Andersonin-G1 (Frogs, amphibians, animals)	Antibacterial, Antifungal, Anti-Gram+, Anti-Gram-, Antimicrobial
971	2251	DRAMP01197	Andersonin-N1 (Frogs, amphibians, animals)	Antibacterial, Antifungal, Anti-Gram+, Anti-Gram-, Antimicrobial
972	2252	DRAMP01198	Andersonin-Q1 (Frogs, amphibians, animals)	Antibacterial, Antifungal, Anti-Gram+, Anti-Gram-, Antimicrobial
973	2255	DRAMP01207	Galensin (Frogs, amphibians, animals)	Antibacterial, Anti-Gram+, Anti-Gram-, Antimicrobial
974	2258	DRAMP01212	Pleurain-A3 (Pleurain A3; Frogs, amphibians, animals)	Antibacterial, Antifungal, Anti-Gram+, Anti-Gram-, Antimicrobial
975	2259	DRAMP01213	Pleurain-A4 (Pleurain A4; Frogs, amphibians, animals)	Antibacterial, Antifungal, Anti-Gram+, Anti-Gram-, Antimicrobial
976	2264	DRAMP01223	Palustrin-2AJ2 (PL2AJ12; Frogs, amphibians, animals)	Antibacterial, Anti-Gram+, Anti-Gram-, Antimicrobial
977	2265	DRAMP01224	Palustrin-2AR (Palustrin-2ARa; Ranatuerin-2SEa; Frogs,	Antibacterial, Anti-Gram+, Anti-Gram-, Antimicrobial
978	2285	DRAMP18404	Polybia-MPII (mastoparan; insects, arthropods, invertebrates,	Antibacterial, antifungal, Anti-Gram+, Anti-Gram-, Antimicrobial
979	2343	DRAMP01338	Amolopin-1a (Frogs, amphibians, animals)	Antibacterial, Antifungal, Anti-Gram+, Anti-Gram-, Antimicrobial
980	2345	DRAMP01342	Amolopin-2b (Frogs, amphibians, animals)	Antibacterial, Antifungal, Anti-Gram+, Anti-Gram-, Antimicrobial
981	2346	DRAMP01343	Amolopin-1c (Frogs, amphibians, animals)	Antibacterial, Antifungal, Anti-Gram+, Anti-Gram-, Antimicrobial
982	2347	DRAMP01344	Amolopin-2c (Frogs, amphibians, animals)	Antibacterial, Antifungal, Anti-Gram+, Anti-Gram-, Antimicrobial
983	2348	DRAMP01345	Amolopin-1d (Frogs, amphibians, animals)	Antibacterial, Antifungal, Anti-Gram+, Anti-Gram-, Antimicrobial
984	2363	DRAMP01444	Nigrocin-1 (Frogs, amphibians, animals)	Antibacterial, Antifungal, Anti-Gram+, Anti-Gram-, Antimicrobial
985	2364	DRAMP01445	Nigrocin-2 (Nigrocin-2LVa; Frogs, amphibians, animals)	Antibacterial, Antifungal, Anti-Gram+, Anti-Gram-, Antimicrobial
986	2372	DRAMP01463	Esculentin-1SEa (Frogs, amphibians, animals)	Antibacterial, Anti-Gram+, Anti-Gram-, Antimicrobial
987	2373	DRAMP01464	Esculentin-1SEb (Frogs, amphibians, animals)	Antibacterial, Anti-Gram+, Anti-Gram-, Antimicrobial
988	2374	DRAMP01465	Esculentin-1R (Frogs, amphibians, animals)	Antibacterial, Anti-Gram+, Anti-Gram-, Antimicrobial
989	2382	DRAMP01489	Esculentin-1A (Frogs, amphibians, animals)	Antibacterial, Anti-Gram+, Anti-Gram-, Antimicrobial
990	2383	DRAMP01492	Esculentin-IIb (Frogs, amphibians, animals)	Antibacterial, Anti-Gram+, Anti-Gram-, Antimicrobial
991	2388	DRAMP18312	Propionicin PLG-1(Bacteriocin)	Antibacterial, Antifungal, Anti-Gram+, Anti-Gram-, Antimicrobial
992	2392	DRAMP01519	Esculentin-2PRa (Frogs, amphibians, animals)	Antibacterial, Antifungal, Anti-Gram+, Anti-Gram-, Antimicrobial
993	2475	DRAMP01671	Dermaseptin-4 (DS IV; Dermaseptin-S4, DS4; Frogs, amphibians,	Antibacterial, Antifungal, Antiviral, Anti-Gram+, Anti-Gram-, Antimicrobial
994	2503	DRAMP01700	Dermaseptin-H3 (Dermaseptin-like peptide 3, DMS3; Frogs,	Antibacterial, Anti-Gram+, Anti-Gram-, Antimicrobial
995	2518	DRAMP01717	Dermatoxin (Frogs, amphibians, animals)	Antibacterial, Anti-Gram+, Anti-Gram-, Antimicrobial
996	2530	DRAMP02857	Indolicidin (Cathelicidin-4; mammals, animals)	Antibacterial, Anti-Gram+, Anti-Gram-, Antimicrobial
997	2532	DRAMP02819	Anoplin (Insects, arthropods, invertebrates, animals)	Antimicrobial, Antibacterial, Antifungal, Anti-Gram+, Anti-Gram-,
998	2533	DRAMP04395	EP3 (Earthworm,animals)	Antibacterial, Anti-Gram+, Anti-Gram-, Antimicrobial
999	2534	DRAMP04394	EP2 (Earthworm,animals)	Antibacterial, Anti-Gram+, Anti-Gram-, Antimicrobial
1000	2548	DRAMP01772	Temporin-1Ob (Frogs, amphibians, animals)	Antibacterial, Antifungal, Anti-Gram+, Anti-Gram-, Antimicrobial
1001	2560	DRAMP01799	Temporin-1PRa (Temporin 1PRa; Frogs, amphibians, animals)	Antibacterial, Anti-Gram+, Anti-Gram-, Antimicrobial
1002	2561	DRAMP01800	Temporin-1PRb (Temporin 1PRb; Frogs, amphibians, animals)	Antibacterial, Antifungal, Anti-Gram+, Anti-Gram-, Antimicrobial
1003	2562	DRAMP01801	Temporin-1DYa (Frogs, amphibians, animals)	Antibacterial, Anti-Gram+, Anti-Gram-, Antimicrobial
1004	2563	DRAMP01802	Temporin-PTa (Frogs, amphibians, animals)	Antibacterial, Anti-Gram+, Anti-Gram-, Antimicrobial
1005	2565	DRAMP01804	Temporin-CDYb (Brevinin-1CDYb; Frogs, amphibians, animals)	Antibacterial, Anti-Gram+, Anti-Gram-, Antimicrobial
1006	2571	DRAMP01387	Odorranain-P2a (OdP2a; Frogs, amphibians, animals)	Antimicrobial, Antibacterial, Antifungal, Anti-Gram+, Anti- Gram-,
1007	2572	DRAMP01386	Odorranain-P1a (OdP1a; Brevinin-1HS1; Brevinin-1-OA2; Frogs,	Antimicrobial, Antibacterial, Antifungal, Anti-Gram+, Anti- Gram-,

S.no.	PepID	DRAMP_ID	Name of the AMP	Activity
1008	2573	DRAMP01824	Temporin-1Ja (Frogs, amphibians, animals)	Antibacterial, Anti-Gram+, Anti-Gram-, Antimicrobial
1009	2576	DRAMP01126	Maximin-H4 (Toads, amphibians, animals)	Antimicrobial, Antibacterial, Antifungal, Anti-Gram+, Anti- Gram-,
1010	2577	DRAMP01125	Maximin-H3 (Toads, amphibians, animals)	Antimicrobial, Antibacterial, Antifungal, Anti-Gram+, Anti- Gram-,
1011	2621	DRAMP01924	Brevinin-1Ea (Frogs, amphibians, animals)	Antibacterial, Anti-Gram+, Anti-Gram-, Antimicrobial
1012	2622	DRAMP01925	Brevinin-1Eb (Frogs, amphibians, animals)	Antibacterial, Anti-Gram+, Anti-Gram-, Antimicrobial
1013	2623	DRAMP01926	Brevinin-1Ec (Frogs, amphibians, animals)	Antibacterial, Anti-Gram+, Anti-Gram-, Antimicrobial
1014	2624	DRAMP01927	Brevinin-2Ea (Frogs, amphibians, animals)	Antibacterial, Anti-Gram+, Anti-Gram-, Antimicrobial
1015	2625	DRAMP01928	Brevinin-2Eb (Frogs, amphibians, animals)	Antibacterial, Anti-Gram+, Anti-Gram-, Antimicrobial
1016	2626	DRAMP01929	Brevinin-2Ec (Frogs, amphibians, animals)	Antibacterial, Anti-Gram+, Anti-Gram-, Antimicrobial
1017	2628	DRAMP01931	Brevinin-2Ed (Frogs, amphibians, animals)	Antibacterial, Anti-Gram+, Anti-Gram-, Antimicrobial
1018	2629	DRAMP01932	Brevinin-2Ee (Frogs, amphibians, animals)	Antibacterial, Anti-Gram+, Anti-Gram-, Antimicrobial
1019	2631	DRAMP01945	Brevinin-1SE (Frogs, amphibians, animals)	Antibacterial, Anti-Gram+, Anti-Gram-, Antimicrobial
1020	2632	DRAMP01946	Brevinin-20a (Frogs, amphibians, animals)	Antibacterial, Antifungal, Anti-Gram+, Anti-Gram-, Antimicrobial
1021	2633	DRAMP01947	Brevinin-20b (Frogs, amphibians, animals)	Antibacterial, Antifungal, Anti-Gram+, Anti-Gram-, Antimicrobial
1022	2634	DRAMP18294	lactococcin Z(Bacteriocin)	Antibacterial, Anti-Gram+, Anti-Gram-, Antimicrobial
1023	2635	DRAMP01952	Brevinin-1PTb (Frogs, amphibians, animals)	Antibacterial, Anti-Gram+, Anti-Gram-, Antimicrobial
1024	2636	DRAMP01954	Brevinin-2HSb (Frogs, amphibians, animals)	Antibacterial, Anti-Gram+, Anti-Gram-, Antimicrobial
1025	2637	DRAMP01958	Brevinin-2PTd (Frogs, amphibians, animals)	Antibacterial, Anti-Gram+, Anti-Gram-, Antimicrobial
1026	2638	DRAMP01960	Brevinin-1BYa (Frogs, amphibians, animals)	Antibacterial, Antifungal, Anti-Gram+, Anti-Gram-, Antimicrobial
1027	2639	DRAMP01961	Brevinin-1BYb (Frogs, amphibians, animals)	Antibacterial, Antifungal, Anti-Gram+, Anti-Gram-, Antimicrobial
1028	2642	DRAMP01966	Brevinin-1Ya (Frogs, amphibians, animals)	Antibacterial, Antifungal, Anti-Gram+, Anti-Gram-, Antimicrobial
1029	2643	DRAMP01967	Brevinin-1Yb (Frogs, amphibians, animals)	Antibacterial, Antifungal, Anti-Gram+, Anti-Gram-, Antimicrobial
1030	2647	DRAMP01976	Brevinin-2Eg (Frogs, amphibians, animals)	Antibacterial, Anti-Gram+, Anti-Gram-, Antimicrobial
1031	2654	DRAMP18291	Garviecin LG34(Bacteriocin)	Antibacterial, Anti-Gram+, Anti-Gram-, Antimicrobial
1032	2674	DRAMP02012	Brevinin-1T (Brevinin-2T; Frogs, amphibians, animals)	Antibacterial, Anti-Gram+, Anti-Gram-, Antimicrobial
1033	2675	DRAMP02013	Brevinin-1Ta (Frogs, amphibians, animals)	Antibacterial, Anti-Gram+, Anti-Gram-, Antimicrobial
1034	2678	DRAMP02016	Brevinin-1DYa (Frogs, amphibians, animals)	Antibacterial, Anti-Gram+, Anti-Gram-, Antimicrobial
1035	2679	DRAMP02017	Brevinin-2DYa (Frogs, amphibians, animals)	Antibacterial, Anti-Gram+, Anti-Gram-, Antimicrobial
1036	2680	DRAMP02018	Brevinin-1DYb (Brevinin-1CDYb; Frogs, amphibians, animals)	Antibacterial, Anti-Gram+, Anti-Gram-, Antimicrobial
1037	2681	DRAMP02020	Brevinin-1DYc (Frogs, amphibians, animals)	Antibacterial, Anti-Gram+, Anti-Gram-, Antimicrobial
1038	2684	DRAMP18281	Plantaricin KL-1Y (Bacteriocin)	Antibacterial, Anti-Gram+, Anti-Gram-, Antimicrobial
1039	2694	DRAMP02089	Brevinin-1La (Brevinin-1PRd; Frogs, amphibians, animals)	Antibacterial, Anti-Gram+, Anti-Gram-, Antimicrobial
1040	2695	DRAMP01124	Maximin-H2 (Toads, amphibians, animals)	Antimicrobial, Antibacterial, Antifungal, Anti-Gram+, Anti-Gram-,
1041	2696	DRAMP01123	Maximin-H1 (Toads, amphibians, animals)	Antimicrobial, Antibacterial, Antifungal, Anti-Gram+, Anti-Gram-,
1042	2697	DRAMP01111	Maximin-5 (Toads, amphibians, animals)	Antimicrobial, Antibacterial, Antifungal, Antiviral, Anticancer, Anti-Gram+, Anti-Gram-,
1043	2698	DRAMP01110	Maximin-4 (Toads, amphibians, animals)	Antimicrobial, Antibacterial, Antifungal, Antiviral, Anticancer, Anti-Gram+, Anti-Gram-,
1044	2699	DRAMP01109	Maximin-3 (Toads, amphibians, animals)	Antimicrobial, Antibacterial, Antifungal, Antiviral, Anticancer, Anti-Gram+, Anti-Gram-,
1045	2700	DRAMP02100	Brevinin-1Pe (Frogs, amphibians, animals)	Antibacterial, Antifungal, Anti-Gram+, Anti-Gram-, Antimicrobial
1046	2706	DRAMP18272	Enterocin AS-48RJ (Bacteriocin)	Antibacterial, Anti-Gram+, Anti-Gram-, Antimicrobial
1047	2718	DRAMP02132	Antimicrobial peptide 3 (XT-3; Levitide-like peptide; Frogs,	Antibacterial, Anti-Gram+, Anti-Gram-, Antimicrobial
1048	2719	DRAMP02134	Antimicrobial peptide 5 (XT-5; PGLa-like peptide; Frogs, amphibians,	Antibacterial, Antifungal, Anti-Gram+, Anti-Gram-, Antimicrobial
1049	2806	DRAMP02243	Ranatuerin-1T (Brevinin-2T; Frogs, amphibians, animals)	Antibacterial, Anti-Gram+, Anti-Gram-, Antimicrobial
1050	2810	DRAMP02249	Ranatuerin-2SEB (Frogs, amphibians, animals)	Antibacterial, Anti-Gram+, Anti-Gram-, Antimicrobial
1051	2811	DRAMP02250	Ranatuerin-2SEC (Frogs, amphibians, animals)	Antibacterial, Anti-Gram+, Anti-Gram-, Antimicrobial
1052	2813	DRAMP02258	Ranatuerin-IIbYa (Ranatuerin-2bYa; Frogs, amphibians, animals)	Antibacterial, Anti-Gram+, Anti-Gram-, Antimicrobial
1053	2818	DRAMP02284	Pseudin-1 (Pseudin 1; Frogs, amphibians, animals)	Antibacterial, Antifungal, Anti-Gram+, Anti-Gram-, Antimicrobial
1054	2819	DRAMP02285	Pseudin-2 (Pseudin 2; Frogs, amphibians, animals)	Antibacterial, Antifungal, Anti-Gram+, Anti-Gram-, Antimicrobial
1055	2820	DRAMP02286	Pseudin-3 (Pseudin 3; Frogs, amphibians, animals)	Antibacterial, Antifungal, Anti-Gram+, Anti-Gram-, Antimicrobial
1056	2821	DRAMP02287	Pseudin-4 (Pseudin 4; Frogs, amphibians, animals)	Antibacterial, Antifungal, Anti-Gram+, Anti-Gram-, Antimicrobial
1057	2841	DRAMP02332	Piscidin-3 (Pis-3; fish, chordates, animals)	Antibacterial, Antiviral, Anti-Gram+, Anti-Gram-, Antimicrobial
1058	2851	DRAMP01375	Odorranain-E1 (OdE1; Frogs, amphibians, animals)	Antimicrobial, Antibacterial, Antifungal, Anti-Gram+, Anti- Gram-,
1059	2854	DRAMP02353	Pleurocidin-like peptide WFX (fish, chordates, animals; Predicted)	Antibacterial, Antifungal, Anti-Gram+, Anti-Gram-, Antimicrobial
1060	2868	DRAMP02245	Ranatuerin-2Cb (Ranatuerin 2Cb; Frogs, amphibians, animals)	Antimicrobial, Antibacterial, Antifungal, Anti-Gram+, Anti-Gram-,

S.no.	PepID	DRAMP_ID	Name of the AMP	Activity
1061	2869	DRAMP02398	Antimicrobial peptide GP-19 (GP-19)	Antibacterial, Antifungal, Anti-Gram+, Anti-Gram-, Antimicrobial
1062	2876	DRAMP02407	Napin-like polypeptide (Contains: Napin-like polypeptide small chain	Antibacterial, Anti-Gram+, Anti-Gram-, Antimicrobial
1063	2890	DRAMP02437	Papillosin	Antibacterial, Anti-Gram+, Anti-Gram-, Antimicrobial
1064	2891	DRAMP02438	Halocyntin	Antibacterial, Anti-Gram+, Anti-Gram-, Antimicrobial
1065	2896	DRAMP02447	Antimicrobial protein 2 (Antimicrobial protein AN5-2)	Antibacterial, Anti-Gram+, Anti-Gram-, Antimicrobial
1066	2901	DRAMP02453	S. litura moricin (Sl moricin; Insects, animals)	Antibacterial, Anti-Gram+, Anti-Gram-, Antimicrobial
1067	2902	DRAMP02454	Theromacin (Arthropods, animals)	Antibacterial, Anti-Gram+, Anti-Gram-, Antimicrobial
1068	2904	DRAMP02457	L-amino-acid oxidase (Balt-LAAO-I; LAAO; LAO; snakes, reptils,	Antibacterial, Anti-Gram+, Anti-Gram-, Antimicrobial
1069	2909	DRAMP02462	L-amino-acid oxidase (LAAO; LAO; snakes, reptils, animals)	Antibacterial, Antiparasitic, Anti-Gram+, Anti-Gram-, Antimicrobial
1070	2951	DRAMP02511	Crotamine (defensin-like toxin; Snakes, reptiles, animals)	Antibacterial, Antifungal, Cytotoxicity, Anti-Gram+, Anti-Gram-, Antimicrobial
1071	2999	DRAMP18253	Piscicocin CS526(Bacteriocin)	Antibacterial, Anti-Gram+, Anti-Gram-, Antimicrobial
1072	3003	DRAMP02570	Penaeidin-1 (Pen-1; shrimps, Arthropods, animals)	Antibacterial, Antifungal, Anti-Gram+, Anti-Gram-, Antimicrobial
1073	3004	DRAMP02571	Penaeidin-2a (Pen-2a; shrimps, Arthropods, animals)	Antibacterial, Antifungal, Anti-Gram+, Anti-Gram-, Antimicrobial
1074	3006	DRAMP02575	Penaeidin-3b (Pen-3b; shrimps, Arthropods, animals)	Antibacterial, Antifungal, Anti-Gram+, Anti-Gram-, Antimicrobial
1075	3007	DRAMP02576	Penaeidin-3c (Pen-3c; shrimps, Arthropods, animals)	Antibacterial, Antifungal, Anti-Gram+, Anti-Gram-, Antimicrobial
1076	3028	DRAMP02602	Clavaspirin (chordates, animals)	Antibacterial, Anti-Gram+, Anti-Gram-, Antimicrobial
1077	3050	DRAMP18250	Laterosporulin (Bacteriocin)	Antibacterial, Anti-Gram+, Anti-Gram-, Antimicrobial
1078	3052	DRAMP18248	Bifidin I(Bacteriocin)	Antibacterial, Anti-Gram+, Anti-Gram-, Antimicrobial
1079	3053	DRAMP18249	Bac-GM100 (Bacteriocin)	Antibacterial, Antifungal, Anti-Gram+, Anti-Gram-, Antimicrobial
1080	3059	DRAMP18247	Bacthuricin F4(Bacteriocin)	Antibacterial, Anti-Gram+, Anti-Gram-, Antimicrobial
1081	3065	DRAMP02642	Rhesus theta-defensin 1 (RTD-1; primates, mammals, animals)	Antibacterial, Antifungal, Antiviral, Anti-Gram+, Anti-Gram-, Antimicrobial
1082	3066	DRAMP02643	Rhesus theta-defensin 2 (RTD-2; primates, mammals, animals)	Antibacterial, Antifungal, Anti-Gram+, Anti-Gram-, Antimicrobial
1083	3067	DRAMP02644	Rhesus theta-defensin 3 (RTD-3; primates, mammals, animals)	Antibacterial, Antiviral, Anti-Gram+, Anti-Gram-, Antimicrobial
1084	3075	DRAMP02653	Neutrophil defensin 1 (RMAD-1; primates, mammals, animals)	Antibacterial, Antifungal, Anti-Gram+, Anti-Gram-, Antimicrobial
1085	3076	DRAMP02654	Neutrophil defensin 2 (RMAD-2; primates, mammals, animals)	Antibacterial, Antifungal, Anti-Gram+, Anti-Gram-, Antimicrobial
1086	3081	DRAMP02659	Neutrophil defensin 3 (RMAD-3; primates, mammals, animals)	Antibacterial, Antifungal, Anti-Gram+, Anti-Gram-, Antimicrobial
1087	3082	DRAMP02660	Neutrophil defensin 4 (RMAD-4; primates, mammals, animals)	Antibacterial, Antifungal, Anti-Gram+, Anti-Gram-, Antimicrobial
1088	3083	DRAMP02661	Neutrophil defensin 5 (RMAD-5; primates, mammals, animals)	Antibacterial, Antifungal, Anti-Gram+, Anti-Gram-, Antimicrobial
1089	3084	DRAMP02662	Neutrophil defensin 6 (RMAD-6; primates, mammals, animals)	Antibacterial, Antifungal , Anti-Gram+, Anti-Gram-, Antimicrobial
1090	3085	DRAMP02663	Neutrophil defensin 7 (RMAD-7; primates, mammals, animals)	Antibacterial, Antifungal, Antimicrobial, Anti-Gram+, Anti-Gram-,
1091	3119	DRAMP02698	Rhesus macaque oral alpha-defensins (ROADs; primates, mammals,	Antibacterial, Antifungal, Anti-Gram+, Anti-Gram-, Antimicrobial
1092	3128	DRAMP18242	Fengycin B2 (Bacteriocin)	Antibacterial, Antifungal, Anti-Gram+, Anti-Gram-, Antimicrobial
1093	3130	DRAMP18240	Fengycin C(Bacteriocin)	Antibacterial, Antifungal, Anti-Gram+, Anti-Gram-, Antimicrobial
1094	3131	DRAMP18241	Subtilomycin(Bacteriocin)	Antibacterial, Anti-Gram+, Anti-Gram-, Antimicrobial
1095	3132	DRAMP18239	Fengycin A2(Bacteriocin)	Antibacterial, Antifungal, Anti-Gram+, Anti-Gram-, Antimicrobial
1096	3133	DRAMP18238	Fengycin B(Bacteriocin)	Antibacterial, Antifungal, Anti-Gram+, Anti-Gram-, Antimicrobial
1097	3144	DRAMP18237	Fengycin A(Bacteriocin)	Antibacterial, Antifungal, Anti-Gram+, Anti-Gram-, Antimicrobial
1098	3157	DRAMP02739	TEWP (turtle egg-white protein; Reptiles, animals)	Antibacterial, Antiviral, Anti-Gram+, Anti-Gram-, Antimicrobial
1099	3158	DRAMP02741	Pelovaterin (defensin-like AMP; Gly-rich; Reptiles, animals)	Antibacterial, Anti-Gram+, Anti-Gram-, Antimicrobial
1100	3159	DRAMP02742	Defensin-like turtle egg white protein TEWP (TEWP; Reptiles,	Antibacterial, Antiviral, Anti-Gram+, Anti-Gram-, Antimicrobial
1101	3168	DRAMP03743	Androctonin (Arthropods, animals)	Antimicrobial, Antibacterial, Antifungal, Anti-Gram+, Anti- Gram-,
1102	3171	DRAMP02755	Ponericin G3 (ants, insects, animals)	Antibacterial, Antifungal, Anti-Gram+, Anti-Gram-, Antimicrobial
1103	3172	DRAMP02756	Ponericin G4 (ants, insects, animals)	Antibacterial, Antifungal, Anti-Gram+, Anti-Gram-, Antimicrobial
1104	3174	DRAMP02758	Ponericin G6 (ants, insects, animals)	Antibacterial, Antifungal, Anti-Gram+, Anti-Gram-, Antimicrobial
1105	3176	DRAMP02760	Ponericin-L1 (ants, insects, animals)	Antibacterial, Anti-Gram+, Anti-Gram-, Antimicrobial
1106	3177	DRAMP02761	Ponericin-L2 (ants, insects, animals)	Antibacterial, Antiviral, Anti-Gram+, Anti-Gram-, Antimicrobial
1107	3178	DRAMP02762	Ponericin-W1 (ants, insects, animals)	Antibacterial, Antifungal, Insecticidal, Anti-Gram+, Anti-Gram-, Antimicrobial
1108	3180	DRAMP02764	Ponericin-W3 (ants, insects, animals)	Antibacterial, Antifungal, Insecticidal, Anti-Gram+, Anti- Gram-, Antimicrobial
1109	3181	DRAMP02765	Ponericin-W4 (ants, insects, animals)	Antibacterial, Antifungal, Insecticidal, Anti-Gram+, Anti- Gram-, Antimicrobial
1110	3182	DRAMP02766	Ponericin-W5 (ants, insects, animals)	Antibacterial, Antifungal, Insecticidal, Anti-Gram+, Anti-Gram-, Antimicrobial
1111	3183	DRAMP02767	Ponericin-W6 (ants, insects, animals)	Antibacterial, Anti-Gram+, Anti-Gram-, Antimicrobial
1112	3184	DRAMP02770	Pilosulin 3 (ants, insects, animals)	Antibacterial, Anti-Gram+, Anti-Gram-, Antimicrobial
1113	3185	DRAMP02771	Pilosulin 4 (ants, insects, animals)	Antibacterial, Anti-Gram+, Anti-Gram-, Antimicrobial

S.no.	PepID	DRAMP_ID	Name of the AMP	Activity
1114	3189	DRAMP02781	Coleoptericin (Insects, animals)	Antibacterial, Anti-Gram+, Anti-Gram-, Antimicrobial
1115	3191	DRAMP02783	Peptide C (Insects, animals)	Antibacterial, Anti-Gram+, Anti-Gram-, Antimicrobial
1116	3210	DRAMP02803	Mytilin-A (molluscas, animals)	Antibacterial, Anti-Gram+, Anti-Gram-, Antimicrobial
1117	3211	DRAMP02804	Mytilus defensin-B (molluscas, animals)	Antibacterial, Anti-Gram+, Anti-Gram-, Antimicrobial
1118	3219	DRAMP18235	Gageopeptide D(Bacteriocin)	Antifungal, Antibacterial, Anti-Gram+, Anti-Gram-, Antimicrobial
1119	3220	DRAMP02816	Lumbrican	Antibacterial, Antifungal, Anti-Gram+, Anti-Gram-, Antimicrobial
1120	3221	DRAMP02818	Dicynthaurin	Antibacterial, Anti-Gram+, Anti-Gram-, Antimicrobial
1121	3234	DRAMP02832	Reactive oxygen species modulator 1 (ROS modulator 1; mammals,	Antibacterial, Anti-Gram+, Anti-Gram-, Antimicrobial
1122	3239	DRAMP18233	Gageopeptide B(Bacteriocin)	Antifungal, Antibacterial, Anti-Gram+, Anti-Gram-, Antimicrobial
1123	3240	DRAMP18234	Gageopeptide C(Bacteriocin)	Antifungal, Antibacterial, Anti-Gram+, Anti-Gram-, Antimicrobial
1124	3249	DRAMP01356	Ranalexin-1Ca (Ranatuerin 1Ca; Frogs, amphibians, animals)	Antimicrobial, Antibacterial, Antifungal, Anti-Gram+, Anti-Gram-,
1125	3250	DRAMP02864	Bovine Beta-defensin 7 (bBD-7; BNBD-7; BNDB-7; mammals,	Antibacterial, Anti-Gram+, Anti-Gram-, Antimicrobial
1126	3251	DRAMP02871	Beta-defensin 119 (Defensin, beta 119; mammals, animals)	Antibacterial, Anti-Gram+, Anti-Gram-, Antimicrobial
1127	3281	DRAMP02914	Cathelicidin-1 (Bactenecin-1, Bac1; Cyclic dodecapeptide; mammals,	Antibacterial, Anti-Gram+, Anti-Gram-, Antimicrobial
1128	3291	DRAMP02929	Antimicrobial protein 1	Antibacterial, Anti-Gram+, Anti-Gram-, Antimicrobial
1129	3292	DRAMP02930	Antimicrobial protein 2 (crabs, Arthropods, animals)	Antibacterial, Anti-Gram+, Anti-Gram-, Antimicrobial
1130	3294	DRAMP02936	Big defensin (crabs, Arthropods, animals)	Antibacterial, Antifungal, Anti-Gram+, Anti-Gram-, Antimicrobial
1131	3295	DRAMP02937	Tachycitin (crabs, Arthropods, animals)	Antibacterial, Antifungal, Anti-Gram+, Anti-Gram-, Antimicrobial
1132	3296	DRAMP18230	Gageotetrin B (Bacteriocin)	Antifungal, Antibacterial, Anti-Gram+, Anti-Gram-, Antimicrobial
1133	3297	DRAMP18231	Gageotetrin C (Bacteriocin)	Antifungal, Antibacterial, Anti-Gram+, Anti-Gram-, Antimicrobial
1134	3298	DRAMP18232	Gageopeptide A(Bacteriocin)	Antifungal, Antibacterial, Anti-Gram+, Anti-Gram-, Antimicrobial
1135	3301	DRAMP02941	Tachystatin-A1 (crabs, Arthropods, animals)	Antibacterial, Antifungal, Anti-Gram+, Anti-Gram-, Antimicrobial
1136	3302	DRAMP02942	Tachystatin-A2 (crabs, Arthropods, animals)	Antibacterial, Antifungal, Anti-Gram+, Anti-Gram-, Antimicrobial
1137	3305	DRAMP02945	Tachystatin-C (crabs, Arthropods, animals)	Antibacterial, Antifungal, Anti-Gram+, Anti-Gram-, Antimicrobial
1138	3308	DRAMP18228	Gageostatin C (Bacteriocin)	Antibacterial, antifungal, Anti-Gram+, Anti-Gram-, Antimicrobial
1139	3309	DRAMP18229	Gageotetrin A (Bacteriocin)	Antifungal, Antibacterial, Anti-Gram+, Anti-Gram-, Antimicrobial
1140	3312	DRAMP02955	Hedistin (marine annelid, Metazoa)	Antibacterial, Anti-Gram+, Anti-Gram-, Antimicrobial
1141	3316	DRAMP02968	Prophenin-1 (C6, PF-1; Pro-rich; pigs, mammals, animals)	Antibacterial, Anti-Gram+, Anti-Gram-, Antimicrobial
1142	3317	DRAMP02969	Prophenin-2 (C12, PF-2, PR-2; Pro-rich; pigs, mammals, animals)	Antibacterial, Anti-Gram+, Anti-Gram-, Antimicrobial
1143	3318	DRAMP02971	Protegrin-2 (Protegrin 2; PG-2; pigs, mammals, animals)	Antibacterial, Antifungal, Anti-Gram+, Anti-Gram-, Antimicrobial
1144	3319	DRAMP02972	Protegrin-3 (Protegrin 3; PG-3; pigs, mammals, animals)	Antibacterial, Antifungal, Anti-Gram+, Anti-Gram-, Antimicrobial
1145	3322	DRAMP02976	Beta-defensin 1 (BD-1; Defensin, beta 1; pigs, mammals, animals)	Antibacterial, Anti-Gram+, Anti-Gram-, Antimicrobial
1146	3327	DRAMP02982	Reactive oxygen species modulator 1 (ROS modulator 1; pigs,	Antibacterial, Anti-Gram+, Anti-Gram-, Antimicrobial
1147	3329	DRAMP02984	Neutrophil cationic antibacterial polypeptide of 11 kDa (CAP11; pigs,	Antibacterial, Anti-Gram+, Anti-Gram-, Antimicrobial
1148	3330	DRAMP02985	Neutrophil cationic peptide 2 (CP-2; GNCP-2; pigs, mammals,	Antibacterial, Antifungal, Antiviral, Anti-Gram+, Anti-Gram-, Antimicrobial
1149	3331	DRAMP02986	Neutrophil cationic peptide 1 (GPNP; Antiviral defensin; pigs,	Antibacterial, Antifungal, Antiviral, Anti-Gram+, Anti-Gram-, Antimicrobial
1150	3334	DRAMP02989	Lasioglossin LL-I (Insects, animals)	Antibacterial, Anticancer, Anti-Gram+, Anti-Gram-, Antimicrobial
1151	3335	DRAMP02990	Lasioglossin LL-II (Insects, animals)	Antibacterial, Anticancer, Anti-Gram+, Anti-Gram-, Antimicrobial
1152	3336	DRAMP02991	Lasioglossin LL-III (Insects, animals)	Antibacterial, Anticancer, Anti-Gram+, Anti-Gram-, Antimicrobial
1153	3348	DRAMP03023	Mastoparan (Protonectarina-MP; Insects, animals)	Antibacterial, Antifungal, Anti-Gram+, Anti-Gram-, Antimicrobial
1154	3349	DRAMP18227	Gageostatin B (Bacteriocin)	Antibacterial, antifungal, Anti-Gram+, Anti-Gram-, Antimicrobial
1155	3355	DRAMP03049	Eumenine mastoparan-OD (EMP-OD; Venom peptide 1, OdVP1;	Antibacterial, Antifungal, Anti-Gram+, Anti-Gram-, Antimicrobial
1156	3366	DRAMP18226	Gageostatin A (Bacteriocin)	Antibacterial, antifungal, Anti-Gram+, Anti-Gram-, Antimicrobial
1157	3369	DRAMP03073	Sapecin-C (Sapecin C; defensins; Insects, animals)	Antibacterial, Anti-Gram+, Anti-Gram-, Antimicrobial
1158	3378	DRAMP03083	Sapecin-B (defensins; Insects, animals)	Antibacterial, Anti-Gram+, Anti-Gram-, Antimicrobial
1159	3379	DRAMP03084	Ceratotoxin-B (Insects, animals)	Antibacterial, Anti-Gram+, Anti-Gram-, Antimicrobial
1160	3380	DRAMP03085	Ceratotoxin-A (Insects, animals)	Antibacterial, Anti-Gram+, Anti-Gram-, Antimicrobial
1161	3381	DRAMP03086	Ceratotoxin-D (Insects, animals)	Antibacterial, Anti-Gram+, Anti-Gram-, Antimicrobial
1162	3400	DRAMP18223	Sonorensin(Bacteriocin)	Antibacterial, Anti-Gram+, Anti-Gram-, Antimicrobial
1163	3418	DRAMP03134	Defensin-D (AaeDefD; Insects, animals)	Antibacterial, Anti-Gram+, Anti-Gram-, Antimicrobial
1164	3436	DRAMP03157	Halocidin subunit B	Antibacterial, Anti-Gram+, Anti-Gram-, Antimicrobial
1165	3437	DRAMP03158	Halocidin subunit A (invertebrates, animals; Preclinical)	Antibacterial, Anti-Gram+, Anti-Gram-, Antimicrobial
1166	3441	DRAMP03163	R. prolixus defensin A (RprDefA; insect defensin; Insects, animals)	Antibacterial, Insecticidal, Anti-Gram+, Anti-Gram-, Antimicrobial

S.no.	PepID	DRAMP_ID	Name of the AMP	Activity
1167	3442	DRAMP03164	R. prolixus defensin B (RprDefB; insect defensin; Insects, animals)	Antibacterial, Insecticidal, Anti-Gram+, Anti-Gram-, Antimicrobial
1168	3443	DRAMP03165	R. prolixus defensin C (RprDefC; insect defensin; Insects, animals)	Antibacterial, Insecticidal, Anti-Gram+, Anti-Gram-, Antimicrobial
1169	3449	DRAMP03174	Arenicin-2 (Ar-2; marine polychaeta, animals)	Antibacterial, Antifungal, Anti-Gram+, Anti-Gram-, Antimicrobial
1170	3450	DRAMP03175	Perinerin	Antibacterial, Antifungal, Anti-Gram+, Anti-Gram-, Antimicrobial
1171	3454	DRAMP03180	ASABF-alpha (ASABF; nematodes, animals)	Antibacterial, Antifungal, Anti-Gram+, Anti-Gram-, Antimicrobial
1172	3456	DRAMP03183	Naegleriapore A	Antibacterial, Antiprotozoal, Cytotoxicity, Anti-Gram+, Anti-Gram-, Antimicrobial
1173	3457	DRAMP03184	Naegleriapore B	Antibacterial, Antiprotozoal, Cytotoxicity, Anti-Gram+, Anti-Gram-, Antimicrobial
1174	3465	DRAMP03199	PhD1 (PhD-1; Defensin-1; primates, mammals, animals)	Antibacterial, Antifungal, Anti-Gram+, Anti-Gram-, Antimicrobial
1175	3466	DRAMP03200	PhD2 (PhD-2; Defensin-2; primates, mammals, animals)	Antibacterial, Antifungal, Anti-Gram+, Anti-Gram-, Antimicrobial
1176	3467	DRAMP03201	PhD3 (PhD-3; Defensin-3; primates, mammals, animals)	Antibacterial, Antifungal, Anti-Gram+, Anti-Gram-, Antimicrobial
1177	3473	DRAMP03208	BTD-1 (theta-defensin; primates, mammals, animals)	Antibacterial, Antifungal, Anti-Gram+, Anti-Gram-, Antimicrobial
1178	3474	DRAMP03209	BTD-2 (theta-defensin; primates, mammals, animals)	Antibacterial, Antifungal, Anti-Gram+, Anti-Gram-,Antimicrobial
1179	3476	DRAMP03211	BTD-4 (theta-defensin; primates, mammals, animals)	Antibacterial, Antifungal, Anti-Gram+, Anti-Gram-, Antimicrobial
1180	3477	DRAMP03212	BTD-7 (theta-defensin; primates, mammals, animals)	Antibacterial, Antifungal, Anti-Gram+, Anti-Gram-, Antimicrobial
1181	3485	DRAMP03224	M-ctenitoxin-Cs1c (M-CNTX-Cs1c; Cupiennin-1c; spiders,	Antibacterial, Anti-Gram+, Anti-Gram-, Antimicrobial
1182	3489	DRAMP03238	M-zodatoxin-Lt8c (M-ZDTX-Lt8c; Cytoinsectotoxin-1c, CIT-1c;	Antibacterial, Insecticidal, Anti-Gram+, Anti-Gram-, Antimicrobial
1183	3514	DRAMP18213	Gramicidin S(Bacteriocin)	Antibacterial, Antifungal, Anti-Gram+, Anti-Gram-, Antimicrobial
1184	3525	DRAMP03282	Turkey Heterophil Peptide 1 (Antimicrobial peptide THP1; Birds,	Antibacterial, Anti-Gram+, Anti-Gram-, Antimicrobial
1185	3543	DRAMP03304	Hydramacin-1 (Hm-1; annelida, animals)	Antibacterial, Anti-Gram+, Anti-Gram-, Antimicrobial
1186	3554	DRAMP03317	Cathelicidin-related antimicrobial peptide (AMPs)	Antibacterial, Anti-Gram+, Anti-Gram-, Antimicrobial
1187	3565	DRAMP03329	Alpha-defensin cryptdin-1 (Crp1; Rodents, mammals, animals)	Antibacterial, Anti-Gram+, Anti-Gram-, Antimicrobial
1188	3566	DRAMP03330	Alpha-defensin cryptdin-2 (Defensin-related cryptdin-2; Rodents,	Antibacterial, Anti-Gram+, Anti-Gram-, Antimicrobial
1189	3567	DRAMP03331	Alpha-defensin cryptdin-3 (Defensin-related cryptdin-3; Rodents,	Antibacterial, Anti-Gram+, Anti-Gram-, Antimicrobial
1190	3568	DRAMP03332	Alpha-defensin cryptdin-4 (Defensin-related cryptdin4; Rodents,	Antibacterial, Anti-Gram+, Anti-Gram-, Antimicrobial
1191	3569	DRAMP03333	Alpha-defensin cryptdin-5 (Defensin-related cryptdin5; Rodents,	Antibacterial, Anti-Gram+, Anti-Gram-, Antimicrobial
1192	3570	DRAMP03334	Rodents, mammals, animals)	Antibacterial, Anti-Gram+, Anti-Gram-, Antimicrobial
1193	3593	DRAMP03357	Cryptdin related sequence peptide (CRS4C-1a; Rodents, mammals,	Antibacterial, Anti-Gram+, Anti-Gram-, Antimicrobial
1194	3594	DRAMP03358	Cryptdin related sequence peptide (CRS4C-1d; Rodents, mammals,	Antibacterial, Anti-Gram+, Anti-Gram-, Antimicrobial
1195	3595	DRAMP03359	Cryptdin related sequence peptide (CRS4C-2; Rodents, mammals,	Antibacterial, Anti-Gram+, Anti-Gram-, Antimicrobial
1196	3596	DRAMP03360	Cryptdin related sequence peptide (CRS4C-2b; Rodents, mammals,	Antibacterial, Anti-Gram+, Anti-Gram-, Antimicrobial
1197	3598	DRAMP03362	CRS4C-3c (Cryptdin related sequence peptide; Rodents, mammals,	Antimicrobial , Anti-Gram+, Anti-Gram-,
1198	3602	DRAMP03366	Beta-defensin 1 (BD-1; mBD-1; Defensin, beta 1; Rodents,	Antibacterial, Antifungal, Anti-Gram+, Anti-Gram-, Antimicrobial
1199	3605	DRAMP03369	Beta-defensin 4 (BD-4, mBD-4; Defensin, beta 4; Rodents,	Antibacterial, Anti-Gram+, Anti-Gram-, Antimicrobial
1200	3608	DRAMP03373	Beta-defensin 8 (BD-8, mBD-8; Defensin, beta 8; Rodents,	Antibacterial, Anti-Gram+, Anti-Gram-, Antimicrobial
1201	3635	DRAMP03400	WAP four-disulfide core domain protein 12 (Rodents, mammals,	Antibacterial, Anti-Gram+, Anti-Gram-, Antimicrobial
1202	3637	DRAMP03402	WAP four-disulfide core domain protein 15B (Elafin-like protein I;	Antibacterial, Anti-Gram+, Anti-Gram-, Antimicrobial
1203	3639	DRAMP03407	Defr1 (Murine beta-defensin related peptide; Rodents, mammals,	Antibacterial, Anti-Gram+, Anti-Gram-, Antimicrobial
1204	3640	DRAMP03408	Neutrophil defensin 1 (HANP-1; alpha-defensin; Rodents, mammals,	Antibacterial, Antifungal, Anti-Gram+, Anti-Gram-, Antimicrobial
1205	3641	DRAMP03409	Neutrophil defensin 2 (HANP-2; alpha-defensin; Rodents, mammals,	Antibacterial, Antifungal, Anti-Gram+, Anti-Gram-, Antimicrobial
1206	3642	DRAMP03410	Neutrophil defensin 3 (HANP-3; alpha-defensin; Rodents, mammals,	Antibacterial, Antifungal, Anti-Gram+, Anti-Gram-, Antimicrobial
1207	3643	DRAMP03411	Neutrophil defensin 4 (HANP-4; alpha-defensin; Rodents, mammals,	Antibacterial, Antifungal, Anti-Gram+, Anti-Gram-, Antimicrobial
1208	3651	DRAMP03420	Neutrophil antibiotic peptide NP-2 (RatNP-2; Rodents, mammals,	Antibacterial, Antifungal, Anti-Gram+, Anti-Gram-, Antimicrobial
1209	3652	DRAMP03421	Neutrophil antibiotic peptide NP-3 (RatNP-3a, RatNP-3b; Rodents,	Antibacterial, Antifungal, Antiviral, Anti-Gram+, Anti-Gram-, Antimicrobial
1210	3696	DRAMP03470	Defensin-1 (American oyster defensin, AOD; molluscs, animals)	Antibacterial, Anti-Gram+, Anti-Gram-, Antimicrobial
1211	3721	DRAMP03501	La-LTP (LJAFP; Insects, animals)	Antibacterial, Antifungal, Anti-Gram+, Anti-Gram-, Antimicrobial
1212	3728	DRAMP03510	Cecropin-A (Insects, animals)	Antibacterial, Antiviral, Anti-Gram+, Anti-Gram-, Antimicrobial
1213	3729	DRAMP03511	Cecropin-B (Immune protein P9; Insects, animals)	Antibacterial, Anti-Gram+, Anti-Gram-, Antimicrobial
1214	3730	DRAMP03512	Cecropin-D (Cecropin D; Insects, animals)	Antibacterial, Anti-Gram+, Anti-Gram-, Antimicrobial
1215	3737	DRAMP03534	Antibacterial peptide enbocin (Moricin; Insects, animals)	Antibacterial, Anti-Gram+, Anti-Gram-, Antimicrobial
1216	3738	DRAMP03535	Lebocin-1/2 (Pro-rich; Insects, animals)	Antibacterial, Anti-Gram+, Anti-Gram-, Antimicrobial
1217	3739	DRAMP03536	Lebocin-3 (LEB 3; Insects, animals)	Antibacterial, Anti-Gram+, Anti-Gram-, Antimicrobial
1218	3742	DRAMP03554	CCL20(1-67) (Human, mammals, animals)	Antibacterial, Anti-Gram+, Anti-Gram-, Antimicrobial
1219	3743	DRAMP03555	CCL20(2-70) (Human, mammals, animals)	Antibacterial, Anti-Gram+, Anti-Gram-, Antimicrobial

S.no.	PepID	DRAMP_ID	Name of the AMP	Activity
1220	3744	DRAMP03556	C-C motif chemokine 20 (Human, mammals, animals)	Antibacterial, Anti-Gram+, Anti-Gram-, Antimicrobial
1221	3749	DRAMP03564	Human hepcidin-20 (Hepc20; one chain of Hepcidin; Human,	Antibacterial, Antifungal, Anti-Gram+, Anti-Gram-, Antimicrobial
1222	3750	DRAMP03565	Human hepcidin-25 (Hepc25; one chain of Hepcidin; Human,	Antibacterial, Antifungal, Anti-Gram+, Anti-Gram-, Antimicrobial
1223	3751	DRAMP03566	Salvic (Human, mammals, animals)	Antibacterial, Anti-Gram+, Anti-Gram-, Antimicrobial
1224	3762	DRAMP03585	Human TC-1 (Chain of Platelet basic protein; Human, mammals,	Antibacterial, Antifungal, Anti-Gram+, Anti-Gram-, Antimicrobial
1225	3763	DRAMP03586	Human TC-2 (Chain of Platelet basic protein; Human, mammals,	Antibacterial, Antifungal, Anti-Gram+, Anti-Gram-, Antimicrobial
1226	3764	DRAMP03587	DCD-1 (chain of Dermcidin; Human, mammals, animals)	Antibacterial, Antifungal, Proteolytic, Anti-Gram+, Anti- Gram-, Antimicrobial
1227	3765	DRAMP03588	Human MUC7 20-Mer (Human, mammals, animals)	Antibacterial, Antifungal, Anti-Gram+, Anti-Gram-, Antimicrobial
1228	3768	DRAMP03591	Neutrophil defensin 1 (Defensin, alpha 1; HNP-1, HP-1; Human,	Antimicrobial, Antibacterial, Antifungal, Anti-Gram+, Anti- Gram-,
1229	3769	DRAMP03592	Neutrophil defensin 2 (HNP-2, HP-2, HP2; Human, mammals,	Antifungal, Antiviral, Anti-Gram+, Anti-Gram-, Antimicrobial
1230	3770	DRAMP03593	Neutrophil defensin 3 (Defensin, alpha 3; HNP-3, HP-3, HP3;	Antibacterial, Antifungal, Antiviral, Anti-Gram+, Anti-Gram-, Antimicrobial
1231	3771	DRAMP03594	Neutrophil defensin 4 (Defensin, alpha 4; HNP-4, HP-4; Human,	Antibacterial, Antifungal, Antiviral, Anti-Gram+, Anti-Gram-, Antimicrobial
1232	3772	DRAMP03595	Human defensin-5 (HD-5; Defensin, alpha 5; Human, mammals,	Antibacterial, Antifungal, Antiviral, Anti-Gram+, Anti-Gram-, Antimicrobial
1233	3773	DRAMP03596	Human defensin-6 (HD-6; Defensin, alpha 6; Human, mammals,	Antifungal, Antiviral, Anti-Gram+, Anti-Gram-, Antimicrobial
1234	3777	DRAMP18203	Panusin (beta defensins; crustaceans, arthropods, invertebrates,	Antibacterial, Anti-Gram+, Anti-Gram-, Antifungal, Antimicrobial
1235	3789	DRAMP18120	BnPRP1 (Plant defensin)	Antibacterial, Antifungal, Anti-Gram+, Anti-Gram-, Antimicrobial
1236	3799	DRAMP18140	VG16KRKP	Antibacterial, Antifungal, Anti-Gram+, Anti-Gram-, Antimicrobial
1237	3802	DRAMP18194	AAEL000598-PA	Antibacterial, Anti-Gram+, Anti-Gram-, Antimicrobial
1238	3808	DRAMP03641	Longicornsin (defensin-like; Arthropods, invertebrates, animals)	Antibacterial, Antifungal, Anti-Gram+, Anti-Gram-, Antimicrobial
1239	3810	DRAMP03644	Cathelicidin-1 (CATH-1; Fowlicidin-1; Birds, animals)	Antibacterial, Cytolytic, Anti-Gram+, Anti-Gram-, Antimicrobial
1240	3811	DRAMP03648	Gallinacin-1 (Gal-1; Beta-defensin 1; Birds, animals)	Antibacterial, Antifungal, Anti-Gram+, Anti-Gram-, Antimicrobial
1241	3812	DRAMP03649	Gallinacin-1 alpha (Gal-1 alpha; Antimicrobial peptide CHP2; Birds,	Antibacterial, Antifungal, Anti-Gram+, Anti-Gram-, Antimicrobial
1242	3813	DRAMP03650	Gallinacin-2 (Gal-2; Beta-defensin 2; Birds, animals)	Antibacterial, Anti-Gram+, Anti-Gram-, Antimicrobial
1243	3823	DRAMP03661	Gallinacin-13 (Gal-13; Beta-defensin 13; Birds, animals)	Antibacterial, Anti-Gram+, Anti-Gram-, Antimicrobial
1244	3836	DRAMP03674	Cystatin-1 (Cystatin-I)	Antibacterial, Anti-Gram+, Anti-Gram-, Antimicrobial
1245	3839	DRAMP03680	Cathelicidin-3.4 (Bactenecin-3.4, Bac3.4; ChBac3.4; ruminant,	Antibacterial, Anti-Gram+, Anti-Gram-, Antimicrobial
1246	3843	DRAMP03692	Defensin-1 (Cll-dlp; Arthropods, animals)	Antibacterial, Anti-Gram+, Anti-Gram-, Antimicrobial
1247	3871	DRAMP03736	Opistoporin-2 (OP2; Non-disulfide-bridged peptide 3.6, NDBP-3.6;	Antibacterial, Antifungal, Anti-Gram+, Anti-Gram-, Antimicrobial
1248	3872	DRAMP03737	Opistoporin-4 (Non-disulfide-bridged peptides 3.7, NDBP-3.7;	Antibacterial, Antifungal, Anti-Gram+, Anti-Gram-, Antimicrobial
1249	3873	DRAMP03739	Buthinin (Sahara scorpion; Arthropods, animals)	Antibacterial, Anti-Gram+, Anti-Gram-, Antimicrobial
1250	3874	DRAMP03740	Androctonus defensin (4 kDa defensin; Arthropods, animals)	Antibacterial, Anti-Gram+, Anti-Gram-, Antimicrobial
1251	3875	DRAMP03741	Ponericin-W-like 32.1 (Arthropods, animals)	Antibacterial, Antifungal, Insecticidal, Anti-Gram+, Anti-Gram-, Antimicrobial
1252	3876	DRAMP03742	Ponericin-W-like 32.2 (Arthropods, animals)	Antibacterial, Antifungal, Insecticidal, Anti-Gram+, Anti-Gram-, Antimicrobial
1253	3880	DRAMP03755	Potassium channel toxin alpha-KTx 1.1 (ChTX-Lq1; charybdotoxin;	Antibacterial, Antifungal, Antiviral, Anti-Gram+, Anti-Gram-, Antimicrobial
1254	3891	DRAMP03766	Heteroscorpine-1 (HS-1; defensins; Arthropods, animals)	Antibacterial, Anti-Gram+, Anti-Gram-, Antimicrobial
1255	3907	DRAMP03787	Neuropeptide-like protein 31 (NLP-31; nematodes, animals)	Antifungal, Antibacterial, Anti-Gram+, Anti-Gram-, Antimicrobial
1256	3910	DRAMP03790	ABF-2 (nematodes, animals)	Antibacterial, Antifungal, Anti-Gram+, Anti-Gram-, Antimicrobial
1257	3944	DRAMP18190	Pantinin-3 (Non-disulfide-bridged peptide 4.22, NDBP-4.22, Non-	Antibacterial, Antifungal, Anti-Gram+, Anti-Gram-, Antimicrobial
1258	3948	DRAMP03872	hLf 21-30 (fragment of human lalctoferricin, residues 21-30)	Antibacterial, Anti-Gram+, Anti-Gram-, Antimicrobial
1259	3949	DRAMP03873	mLf 20-29 (fragment of murine lalctoferricin, residues 20-29)	Antibacterial, Anti-Gram+, Anti-Gram-, Antimicrobial
1260	3950	DRAMP03874	pLf20-29 (fragment of porcine lactoferricin, residues 20-29)	Antibacterial, Anti-Gram+, Anti-Gram-, Antimicrobial
1261	3961	DRAMP03941	Peptide 3 (Trp- and Arg-rich; derivative of Tritrpticin)	Antibacterial, Antifungal, Anti-Gram+, Anti-Gram-, Antimicrobial
1262	3962	DRAMP03942	Peptide 2 (Trp- and Arg-rich; derivative of Tritrpticin)	Antibacterial, Antifungal, Anti-Gram+, Anti-Gram-, Antimicrobial
1263	3965	DRAMP03946	Del 1-3 (Ranalexin analog)	Antibacterial, Anti-Gram+, Anti-Gram-, Antimicrobial
1264	3970	DRAMP03961	KR-12	Antibacterial, Anti-Gram+, Anti-Gram-, Antimicrobial
1265	3976	DRAMP02092	Brevinin-1Bb (Frogs, amphibians, animals)	Antimicrobial, Antibacterial, Antifungal, Anti-Gram+, Anti-Gram-,
1266	3978	DRAMP18189	Pantinin-2 (Non-disulfide-bridged peptide 4.21, NDBP-4.21, Non-	Antibacterial, Antifungal, Anti-Gram+, Anti-Gram-, Antimicrobial
1267	3982	DRAMP04018	Rp-1	Antibacterial, Antifungal, Anti-Gram+, Anti-Gram-, Antimicrobial
1268	3983	DRAMP04037	Immobilized peptide E07LKK	Antibacterial, Antifungal, Anti-Gram+, Anti-Gram-, Antimicrobial
1269	3984	DRAMP04038	Immobilized peptide E14LKK/H14LKK	Antibacterial, Antifungal, Anti-Gram+, Anti-Gram-, Antimicrobial
1270	3985	DRAMP04039	Immobilized peptige E16KGL/H16KGL	Antibacterial, Antifungal, Anti-Gram+, Anti-Gram-, Antimicrobial
1271	3986	DRAMP04040	Immobilized peptide E17KGG	Antibacterial, Antifungal, Anti-Gram+, Anti-Gram-, Antimicrobial
1272	3987	DRAMP04041	Immobilized peptide E18KGG	Antibacterial, Antifungal, Anti-Gram+, Anti-Gram-, Antimicrobial

S.no.	PepID	DRAMP_ID	Name of the AMP	Activity
1273	3988	DRAMP04042	Immobilized peptide E16LKL	Antibacterial, Antifungal, Anti-Gram+, Anti-Gram-, Antimicrobial
1274	3989	DRAMP04043	Immobilized peptid E10KKL	Antibacterial, Antifungal, Anti-Gram+, Anti-Gram-, Antimicrobial
1275	3990	DRAMP04044	Immobilized peptid E12LLK	Antibacterial, Antifungal, Anti-Gram+, Anti-Gram-, Antimicrobial
1276	3991	DRAMP04045	Immobilized peptid E14KKL	Antibacterial, Antifungal, Anti-Gram+, Anti-Gram-, Antimicrobial
1277	3992	DRAMP04046	Immobilized peptide E23GIG magainin2	Antibacterial, Antifungal, Anti-Gram+, Anti-Gram-, Antimicrobial
1278	3993	DRAMP04047	Immobilized peptide E17HSA magainin 2 deletion	Antibacterial, Antifungal, Anti-Gram+, Anti-Gram-, Antimicrobial
1279	4020	DRAMP04172	LK2W2 (LlKmW2 model peptides)	Antibacterial, Anti-Gram+, Anti-Gram-, Antimicrobial
1280	4021	DRAMP04173	L2KW2 (LlKmW2 model peptides)	Antibacterial, Anti-Gram+, Anti-Gram-, Antimicrobial
1281	4169	DRAMP04366	PDD-A-8 (PDD-A analog)	Antibacterial, Anti-Gram+, Anti-Gram-, Antimicrobial
1282	4170	DRAMP04375	PDD-B-5 (PDD-B analog)	Antibacterial, Anti-Gram+, Anti-Gram-, Antimicrobial
1283	4171	DRAMP04384	PMM-5 (PMM analog)	Antibacterial, Anti-Gram+, Anti-Gram-, Antimicrobial
1284	4172	DRAMP04388	PMM-9 (PMM analog)	Antibacterial, Anti-Gram+, Anti-Gram-, Antimicrobial
1285	4424	DRAMP04658	Hymenochirin-5B	Antimicrobial, Anti-Gram+, Anti-Gram-,
1286	4435	DRAMP18188	Pantinin-1 (Non-disulfide-bridged peptide 4.20, NDBP-4.20, Non-	Antibacterial, Antifungal, Anti-Gram+, Anti-Gram-, Antimicrobial
1287	4443	DRAMP04684	Bacteriocin BAC-IB17	Antibacterial, Anti-Gram+, Anti-Gram-, Antimicrobial
1288	4451	DRAMP04700	Basic phospholipase A2 BnpTX-1 (BnPTx-I, svPLA2;	Antibacterial, Antiparasitic, Anti-Gram+, Anti-Gram-, Antimicrobial
1289	4472	DRAMP18187	Toxin LyeTx 1	Antibacterial, Anti-Gram+, Anti-Gram-, Antimicrobial
1290	4473	DRAMP18128	Antimicrobial peptide HsAp4;	Antimicrobial, Antifungal, Anti-Gram+, Anti-Gram-,
1291	4474	DRAMP18129	Antimicrobial peptide HsAp3;	Antimicrobial, Antifungal, Anti-Gram+, Anti-Gram-,
1292	4475	DRAMP18130	Antimicrobial peptide HsAp2	Antimicrobial, Antifungal, Anti-Gram+, Anti-Gram-,
1293	4476	DRAMP18131	Antimicrobial peptide HsAp1 (HsAp)	Antimicrobial, Antifungal, Anti-Gram+, Anti-Gram-,
1294	4485	DRAMP18185	Jingdongin-1	Antibacterial, Antifungal, Anti-Gram+, Anti-Gram-, Antimicrobial
1295	4531	DRAMP18454	Tepmporin-1Ee (frog, amphibians, animals)	Antibacterial, Anti-Gram+, Anti-Gram-, Antimicrobial
1296	4533	DRAMP18456	Pepcon (peptide consensus sequence, synthetic)	Antibacterial, Anti-Gram+, Anti-Gram-, Antimicrobial
1297	4555	DRAMP18488	Css54 (Css from the species name below; scorpions, arachnids,	Antibacterial, Cytolysis, Hemolysis, Anti-Gram+, Anti-Gram-, Antimicrobial
1298	4564	DRAMP03575	LL-37(17-29) (C-terminal fragment of LL-37, LL; Human, mammals,	Antibacterial, Anticancer, Anti-Gram+, Anti-Gram-, Antimicrobial
1299	4566	DRAMP02094	Brevinin-1Bd (Frogs, amphibians, animals)	Antimicrobial, Antibacterial, Antifungal, Anti-Gram+, Anti- Gram-,
1300	4567	DRAMP02095	Brevinin-1Be (Frogs, amphibians, animals)	Antibacterial, Anti-Gram+, Anti-Gram-, Antimicrobial
1301	4568	DRAMP02096	Brevinin-1Bf (Frogs, amphibians, animals)	Antibacterial, Anti-Gram+, Anti-Gram-, Antimicrobial
1302	4569	DRAMP02097	Brevinin-1Pa (Frogs, amphibians, animals)	Antimicrobial, Antibacterial, Antifungal, Anti-Gram+, Anti-Gram-,
1303	4570	DRAMP02098	Brevinin-1Pc (Frogs, amphibians, animals)	Antimicrobial, Antibacterial, Antifungal, Anti-Gram+, Anti-Gram-,
1304	4571	DRAMP02099	Brevinin-1Pd (Frogs, amphibians, animals)	Antimicrobial, Antibacterial, Antifungal, Anti-Gram+, Anti-Gram-,
1305	4572	DRAMP02255	Ranatuerin-2Lb (Ranatuerin 2Lb; Ranaturin-2PRd; Frogs,	Antimicronial, Antibacterial, Antifungal, Anti-Gram+, Anti- Gram-, Antimicrobial
1306	4573	DRAMP02254	Ranatuerin-2La (Ranatuerin 2La; Ranatuerin-2PRa; Frogs,	Antibacterial, Anti-Gram+, Anti-Gram-, Antimicrobial
1307	4574	DRAMP02256	Ranatuerin-2B (Ranatuerin 2B, Frog, amphibians, animals)	Antimicronial, Antibacterial, Antifungal, Anti-Gram+, Anti- Gram-, Antimicrobial
1308	4575	DRAMP02257	Ranatuerin-2P (Ranatuerin 2P; Frogs, amphibians, animals)	Antimicronial, Antibacterial, Antifungal, Anti-Gram+, Anti- Gram-, Antimicrobial
1309	4577	DRAMP18497	TSG-6 (Ixosin-B peptide derivative)	Antibacterial, Anti-Gram+, Anti-Gram-, Antimicrobial
1310	4578	DRAMP18498	TSG-7 (Ixosin-B peptide derivative)	Antibacterial, Anti-Gram+, Anti-Gram-, Antimicrobial
1311	4579	DRAMP18499	TSG-8 (Ixosin-B peptide derivative)	Antibacterial, Anti-Gram+, Anti-Gram-, Antimicrobial
1312	4580	DRAMP18500	TSG-8-1 (Ixosin-B peptide derivative)	Antibacterial, Anti-Gram+, Anti-Gram-, Antimicrobial
1313	4581	DRAMP18501	TSG-9 (Ixosin-B peptide derivative)	Antibacterial, Anti-Gram+, Anti-Gram-, Antimicrobial
1314	4582	DRAMP18502	TSG-10 (Ixosin-B peptide derivative)	Antibacterial, Anti-Gram+, Anti-Gram-, Antimicrobial
1315	4583	DRAMP18503	TSG-11 (Ixosin-B peptide derivative)	Antibacterial, Anti-Gram+, Anti-Gram-, Antimicrobial
1316	4586	DRAMP18506	OG2 (Palustrin-OG1 peptide derivative)	Antibacterial, Anti-Gram+, Anti-Gram-, Antimicrobial
1317	4587	DRAMP18508	gp41w-FKA (gp41 peptide derivative)	Antibacterial, Anti-Gram+, Anti-Gram-, Antimicrobial
1318	4588	DRAMP18509	Px-cec1 (cecropin1 peptide derivative)	Antimicrobial, Antibacterial, Anitifungal, Anti-Gram+, Anti- Gram-,
1319	4595	DRAMP18533	V13KL (V681 peptide derivative)	Antibacterial, Anti-Gram+, Anti-Gram-, Antimicrobial
1320	4600	DRAMP18538	V681	Antibacterial, Anti-Gram+, Anti-Gram-, Antimicrobial
1321	4601	DRAMP18539	V13LL (V681 peptide derivative)	Antibacterial, Anti-Gram+, Anti-Gram-, Antimicrobial
1322	4602	DRAMP18540	V13AL (V681 peptide derivative)	Antibacterial, Anti-Gram+, Anti-Gram-, Antimicrobial
1323	4603	DRAMP18541	V13G (V681 peptide derivative)	Antibacterial, Anti-Gram+, Anti-Gram-, Antimicrobial
1324	4604	DRAMP18542	V13SL (V681 peptide derivative)	Antibacterial, Anti-Gram+, Anti-Gram-, Antimicrobial
1325	4605	DRAMP18543	V13LD (V681 peptide derivative)	Antibacterial, Anti-Gram+, Anti-Gram-, Antimicrobial

S.no.	PepID	DRAMP_ID	Name of the AMP	Activity
1326	4606	DRAMP18544	V13VD (V681 peptide derivative)	Antibacterial, Anti-Gram+, Anti-Gram-, Antimicrobial
1327	4607	DRAMP18545	V13AD (V681 peptide derivative)	Antibacterial, Anti-Gram+, Anti-Gram-, Antimicrobial
1328	4608	DRAMP18546	V13SD (V681 peptide derivative)	Antibacterial, Anti-Gram+, Anti-Gram-, Antimicrobial
1329	4609	DRAMP18547	V13KD (V681 peptide derivative)	Antibacterial, Anti-Gram+, Anti-Gram-, Antimicrobial
1330	4610	DRAMP18548	S11LL (V681 peptide derivative)	Antibacterial, Anti-Gram+, Anti-Gram-, Antimicrobial
1331	4611	DRAMP18549	S11VL (V681 peptide derivative)	Antibacterial, Anti-Gram+, Anti-Gram-, Antimicrobial
1332	4612	DRAMP18550	S11AL (V681 peptide derivative)	Antibacterial, Anti-Gram+, Anti-Gram-, Antimicrobial
1333	4613	DRAMP18551	S11G (V681 peptide derivative)	Antibacterial, Anti-Gram+, Anti-Gram-, Antimicrobial
1334	4614	DRAMP18552	S11KL (V681 peptide derivative)	Antibacterial, Anti-Gram+, Anti-Gram-, Antimicrobial
1335	4615	DRAMP18553	S11LD (V681 peptide derivative)	Antibacterial, Anti-Gram+, Anti-Gram-, Antimicrobial
1336	4616	DRAMP18554	S11VD (V681 peptide derivative)	Antibacterial, Anti-Gram+, Anti-Gram-, Antimicrobial
1337	4617	DRAMP18555	S11AD (V681 peptide derivative)	Antibacterial, Anti-Gram+, Anti-Gram-, Antimicrobial
1338	4618	DRAMP18556	S11SD (V681 peptide derivative)	Antibacterial, Anti-Gram+, Anti-Gram-, Antimicrobial
1339	4619	DRAMP18557	S11KD (V681 peptide derivative)	Antibacterial, Anti-Gram+, Anti-Gram-, Antimicrobial
1340	4620	DRAMP18558	Kn2-7 (BmKn2 peptide derivative)	Antibacterial, Anti-Gram+, Anti-Gram-, Antimicrobial
1341	4621	DRAMP18559	HFU3	Antibacterial, Anti-Gram+, Anti-Gram-, Antimicrobial
1342	4622	DRAMP18560	HFU4	Antibacterial, Anti-Gram+, Anti-Gram-, Antimicrobial
1343	4623	DRAMP18561	HFU5	Antibacterial, Anti-Gram+, Anti-Gram-, Antimicrobial
1344	4624	DRAMP18562	MAP-04-01 (Ixosin-B peptide derivative)	Antibacterial, Anti-Gram+, Anti-Gram-, Antimicrobial
1345	4625	DRAMP18563	MAP-04-02 (Ixosin-B peptide derivative)	Antibacterial, Anti-Gram+, Anti-Gram-, Antimicrobial
1346	4626	DRAMP18564	MAP-04-03 (Ixosin-B peptide derivative)	Antibacterial, Anti-Gram+, Anti-Gram-, Antimicrobial
1347	4627	DRAMP18565	MAP-04-04 (Ixosin-B peptide derivative)	Antibacterial, Anti-Gram+, Anti-Gram-, Antimicrobial
1348	4628	DRAMP18566	LL-IIIs-1 (lasioglossin III peptide derivative)	Antimicrobial, Antibacterial, Antifungal, Anti-Gram+, Anti-Gram-,
1349	4629	DRAMP18567	LL-IIIs-2 (lasioglossin III peptide derivative)	Antimicrobial, Antibacterial, Antifungal, Anti-Gram+, Anti-Gram-,
1350	4630	DRAMP18568	LL-IIIs-3 (lasioglossin III peptide derivative)	Antimicrobial, Antibacterial, Antifungal, Anti-Gram+, Anti-Gram-,
1351	4631	DRAMP18569	LL-IIIs-4 (lasioglossin III peptide derivative)	Antibacterial, Anti-Gram+, Anti-Gram-, Antimicrobial
1352	4632	DRAMP18570	LL-IIIs-5 cis (lasioglossin III peptide derivative)	Antibacterial, Anti-Gram+, Anti-Gram-, Antimicrobial
1353	4633	DRAMP18571	LL-IIIs-5 trans (lasioglossin III peptide derivative)	Antibacterial, Anti-Gram+, Anti-Gram-, Antimicrobial
1354	4634	DRAMP18572	LL-IIIs-6a (lasioglossin III peptide derivative)	Antibacterial, Anti-Gram+, Anti-Gram-, Antimicrobial
1355	4635	DRAMP18573	LL-IIIs-6b (lasioglossin III peptide derivative)	Antibacterial, Anti-Gram+, Anti-Gram-, Antimicrobial
1356	4636	DRAMP18574	MEP-N (melectin peptide derivative)	Antimicrobial, Antibacterial, Antifungal, Anti-Gram+, Anti-Gram-,
1357	4637	DRAMP18575	MEP-Ns-1 (melectin peptide derivative)	Antimicrobial, Antibacterial, Antifungal, Anti-Gram+, Anti-Gram-,
1358	4638	DRAMP18576	MEP-Ns-2 (melectin peptide derivative)	Antibacterial, Anti-Gram+, Anti-Gram-, Antimicrobial
1359	4639	DRAMP18577	MEP-Ns-3 (melectin peptide derivative)	Antibacterial, Anti-Gram+, Anti-Gram-, Antimicrobial
1360	4640	DRAMP18578	MEP-Ns-4 cis (melectin peptide derivative)	Antibacterial, Anti-Gram+, Anti-Gram-, Antimicrobial
1361	4641	DRAMP18579	MEP-Ns-4 trans (melectin peptide derivative)	Antibacterial, Anti-Gram+, Anti-Gram-, Antimicrobial
1362	4642	DRAMP18580	MEP-Ns-5 (melectin peptide derivative)	Antibacterial, Anti-Gram+, Anti-Gram-, Antimicrobial
1363	4643	DRAMP18581	MEP-Ns-6 (melectin peptide derivative)	Antibacterial, Anti-Gram+, Anti-Gram-, Antimicrobial
1364	4645	DRAMP18583	Tricystine cyclic cystine TP (ccTP, Tachyplesin-1 peptide derivative)	Antimicrobial, Antibacterial, Antifungal, Anti-Gram+, Anti-Gram-,
1365	4646	DRAMP18584	[Arg13]ccTP (ccTP peptide derivative)	Antimicrobial, Antibacterial, Antifungal, Anti-Gram+, Anti-Gram-,
1366	4647	DRAMP18585	[Arg4,8]ccTP (ccTP peptide derivative)	Antimicrobial, Antibacterial, Antifungal, Anti-Gram+, Anti- Gram-,
1367	4648	DRAMP18586	[Arg4,8,13]ccTP (ccTP peptide derivative)	Antimicrobial, Antibacterial, Antifungal, Anti-Gram+, Anti- Gram-,
1368	4649	DRAMP18587	[Arg4,8,13][Lys18]ccTP (ccTP peptide derivative)	Antimicrobial, Antibacterial, Antifungal, Anti-Gram+, Anti- Gram-,
1369	4650	DRAMP18588	RTD	Antimicrobial, Antibacterial, Antifungal, Anti-Gram+, Anti-Gram-,
1370	4651	DRAMP18589	DSE (Ctx-Ha peptide derivative)	Antimicrobial, Antibacterial, Antifungal, Anti-Gram+, Anti-Gram-,
1371	4652	DRAMP18590	DEP (Ctx-Ha peptide derivative)	Antimicrobial, Antibacterial, Antifungal, Anti-Gram+, Anti- Gram-,
1372	4653	DRAMP18591	DEA (Ctx-Ha peptide derivative)	Antimicrobial, Antibacterial, Antifungal, Anti-Gram+, Anti- Gram-,
1373	4654	DRAMP18592	Ctx(Ile21)-Ha (Ctx-Ha peptide derivative)	Antimicrobial, Antibacterial, Antifungal, Anti-Gram+, Anti- Gram-,
1374	4655	DRAMP18593	Ctx(Ile21)-Ha-VD16 (Ctx-Ha peptide derivative)	Antimicrobial, Antibacterial, Antifungal, Anti-Gram+, Anti- Gram-,
1375	4656	DRAMP18594	Ctx(Ile21)-Ha-VD5,16 (Ctx-Ha peptide derivative)	Antimicrobial, Antibacterial, Antifungal, Anti-Gram+, Anti- Gram-,
1376	4658	DRAMP18596	LL-I/1 (Lasioglossin LL-I peptide derivative)	Antibacterial, Anti-Gram+, Anti-Gram-, Antimicrobial
1377	4659	DRAMP18597	LL-I/2 (Lasioglossin LL-I peptide derivative)	Antibacterial, Anti-Gram+, Anti-Gram-, Antimicrobial
1378	4660	DRAMP18598	LL-I/3 (Lasioglossin LL-I peptide derivative)	Antibacterial, Anti-Gram+, Anti-Gram-, Antimicrobial

S.no.	PepID	DRAMP_ID	Name of the AMP	Activity
1379	4661	DRAMP18599	LL-I/4 (Lasioglossin LL-I peptide derivative)	Antibacterial, Anti-Gram+, Anti-Gram-, Antimicrobial
1380	4662	DRAMP18600	LL-II/1 (Lasioglossin LL-II peptide derivative)	Antibacterial, Anti-Gram+, Anti-Gram-, Antimicrobial
1381	4663	DRAMP18601	LL-II/2 (Lasioglossin LL-II peptide derivative)	Antibacterial, Anti-Gram+, Anti-Gram-, Antimicrobial
1382	4664	DRAMP18602	LL-II/3 (Lasioglossin LL-II peptide derivative)	Antibacterial, Anti-Gram+, Anti-Gram-, Antimicrobial
1383	4665	DRAMP18603	LL-II/4 (Lasioglossin LL-II peptide derivative)	Antibacterial, Anti-Gram+, Anti-Gram-, Antimicrobial
1384	4666	DRAMP18604	LL-III/1 (Lasioglossin LL-III peptide derivative)	Antibacterial, Anti-Gram+, Anti-Gram-, Antimicrobial
1385	4667	DRAMP18605	LL-III/2 (Lasioglossin LL-III peptide derivative)	Antibacterial, Anti-Gram+, Anti-Gram-, Antimicrobial
1386	4668	DRAMP18606	LL-III/3 (Lasioglossin LL-III peptide derivative)	Antibacterial, Anti-Gram+, Anti-Gram-, Antimicrobial
1387	4669	DRAMP18607	LL-III/4 (Lasioglossin LL-III peptide derivative)	Antibacterial, Anti-Gram+, Anti-Gram-, Antimicrobial
1388	4670	DRAMP18608	LL-III/5 (Lasioglossin LL-III peptide derivative)	Antibacterial, Anti-Gram+, Anti-Gram-, Antimicrobial
1389	4671	DRAMP18609	LL-III/6 (Lasioglossin LL-III peptide derivative)	Antibacterial, Anti-Gram+, Anti-Gram-, Antimicrobial
1390	4672	DRAMP18610	LL-III/7 (Lasioglossin LL-III peptide derivative)	Antibacterial, Anti-Gram+, Anti-Gram-, Antimicrobial
1391	4675	DRAMP18613	TPG (Tritrpticin peptide derivative)	Antimicrobial, Antibacterial, Antifungal, Anti-Gram+, Anti-Gram-,
1392	4676	DRAMP18627	D4-K9L8W (D-amino acid substitution of K9L8W)	Antibacterial, Anti-Gram+, Anti-Gram-, Antimicrobial
1393	4677	DRAMP18507	SolyC (Plant defensin; tomato, plants)	Antibacterial, Anti-Gram+, Anti-Gram-, Antimicrobial
1394	4681	DRAMP18614	TPA (Tritrpticin peptide derivative)	Antimicrobial, Antibacterial, Antifungal, Anti-Gram+, Anti- Gram-,
1395	4682	DRAMP18615	TWF (Tritrpticin peptide derivative)	Antimicrobial, Antibacterial, Antifungal, Anti-Gram+, Anti- Gram-,
1396	4683	DRAMP18616	[K22,25,27]-SMAP-29 (SMAP-29 peptide derivative)	Antimicrobial, Antibacterial, Antifungal, Anti-Gram+, Anti-Gram-,
1397	4684	DRAMP18617	[A19]-SMAP-29 (SMAP-29 peptide derivative)	Antimicrobial, Antibacterial, Antifungal, Anti-Gram+, Anti-Gram-,
1398	4685	DRAMP18618	SMAP-29(1-17) (SMAP-29 peptide derivative)	Antimicrobial, Antibacterial, Antifungal, Anti-Gram+, Anti- Gram-,
1399	4686	DRAMP18619	[K2,7,13]-SMAP-29(1-17) (SMAP-29 peptide derivative)	Antimicrobial, Antibacterial, Antifungal, Anti-Gram+, Anti- Gram-,
1400	4687	DRAMP18620	Pep-1-K (Pep-1 peptide derivative)	Antibacterial, Anti-Gram+, Anti-Gram-, Antimicrobial
1401	4689	DRAMP18622	Temporin-PEb (Temporin-PE peptide derivative)	Antimicrobial, Antibacterial, Antifungal, Anti-Gram+, Anti- Gram-,
1402	4690	DRAMP18623	[I5,R8] Mastoparan-L ([I5,R8] MP-L;Mastoparan-L peptide derivative)	Antimicrobial, Antibacterial, Antifungal, Anti-Gram+, Anti- Gram-,
1403	4691	DRAMP18624	K9L8W	Antibacterial, Anti-Gram+, Anti-Gram-, Antimicrobial
1404	4692	DRAMP18625	D3-K9L8W-1 (D-amino acid substitution of K9L8W)	Antibacterial, Anti-Gram+, Anti-Gram-, Antimicrobial
1405	4693	DRAMP18626	D3-K9L8W-2 (D-amino acid substitution of K9L8W)	Antibacterial, Anti-Gram+, Anti-Gram-, Antimicrobial
1406	4694	DRAMP18628	D6-K9L8W (D-amino acid substitution of K9L8W)	Antibacterial, Anti-Gram+, Anti-Gram-, Antimicrobial
1407	4695	DRAMP18629	D9-K9L8W-1 (D-amino acid substitution of K9L8W)	Antibacterial, Anti-Gram+, Anti-Gram-, Antimicrobial
1408	4696	DRAMP18630	D9-K9L8W-2 (D-amino acid substitution of K9L8W)	Antibacterial, Anti-Gram+, Anti-Gram-, Antimicrobial
1409	4697	DRAMP18631	H5(61-90) V1 (Histone H5 peptide derivative)	Antibacterial, Anti-Gram+, Anti-Gram-, Antimicrobial
1410	4700	DRAMP18634	H5(61-90) V3 (Histone H5 peptide derivative)	Antibacterial, Anti-Gram+, Anti-Gram-, Antimicrobial
1411	4702	DRAMP18636	NCP-3a (CTX-1 peptide derivative)	Antimicrobial, Antibacterial, Antifungal, Anti-Gram+, Anti-Gram-,
1412	4703	DRAMP18637	NCP-3b (CTX-1 peptide derivative)	Antimicrobial, Antibacterial, Antifungal, Anti-Gram+, Anti-Gram-,
1413	4707	DRAMP18641	KCM11	Antibacterial, Anti-Gram+, Anti-Gram-, Antimicrobial
1414	4708	DRAMP18642	KCM12	Antimicrobial, Antibacterial, Antifungal, Anti-Gram+, Anti-Gram-,
1415	4709	DRAMP18643	KCM21	Antimicrobial, Antibacterial, Antifungal, Anti-Gram+, Anti-Gram-,
1416	4710	DRAMP18644	KRS22	Antibacterial, Anti-Gram+, Anti-Gram-, Antimicrobial
1417	4714	DRAMP18648	[Pro3,DLeu9]TL(3) (Temporin L peptide derivative)	Antimicrobial, Antibacterial, Antifungal, Anti-Gram+, Anti- Gram-,
1418	4724	DRAMP18658	Temporin-PE (Edible frogs, amphibians, animals)	Antimicrobial, Antibacterial, Antifungal, Anticancer, Anti- Gram+, Anti-Gram-,
1419	4725	DRAMP18659	YFGAP-OH(Yellowfin tuna GAPDH-related antimicrobial peptide;	Antibacterial, Anti-Gram+, Anti-Gram-, Antimicrobial
1420	4726	DRAMP18660	YFGAP-NH2(Yellowfin tuna GAPDH-related antimicrobial peptide;	Antimicrobial, Antibacterial, Antifungal, Anti-Gram+, Anti-Gram-,
1421	4727	DRAMP18661	Ctx-Ha (Frogs, amphibians, animals)	Antimicrobial, Antibacterial, Antifungal, Anti-Gram+, Anti-Gram-,
1422	4728	DRAMP18662	Brevinin 21 (Brevinin-1E truncated peptide 21; Frogs, amphibians,	Antimicrobial, Antibacterial, Antifungal, Anti-Gram+, Anti-Gram-,
1423	4729	DRAMP18663	Brevinin 18 (Brevinin-1E truncated peptide 18; Frogs, amphibians,	Antimicrobial, Antibacterial, Antifungal, Anti-Gram+, Anti-Gram-,
1424	4730	DRAMP18664	Brevinin 15 (Brevinin-1E truncated peptide 15; Frogs, amphibians,	Antimicrobial, Antibacterial, Antifungal, Anti-Gram+, Anti- Gram-,
1425	4731	DRAMP18665	Mastoparan-L (MP-L; insects, arthropods, invertebrates, animals)	Antibacterial, Anti-Gram+, Anti-Gram-, Antimicrobial
1426	4732	DRAMP18666	P1 (Pilosulin-1 1-20; Ant, insects, arthropods, invertebrates, animals)	Antimicrobial, Antibacterial, Anitifungal, Anti-Gram+, Anti- Gram-,
1427	4733	DRAMP18667	Pep-1	Antibacterial, Anti-Gram+, Anti-Gram-, Antimicrobial
1428	4737	DRAMP18671	TO17 (TFPI-1 C-terminal peptide)	Antibacterial, Anti-Gram+, Anti-Gram-, Antimicrobial
1429	4738	DRAMP18672	Peptide 7 (Mollusca/molluscs/mollusks, invertebrates, animals)	Antibacterial, Anti-Gram+, Anti-Gram-, Antimicrobial
1430	4739	DRAMP18673	Peptide 3 (Mollusca/molluscs/mollusks, invertebrates, animals)	Antibacterial, Anti-Gram+, Anti-Gram-, Antimicrobial
1431	4740	DRAMP18674	Peptide 2 (Mollusca/molluscs/mollusks, invertebrates, animals)	Antibacterial, Anti-Gram+, Anti-Gram-, Antimicrobial

S.no.	PepID	DRAMP_ID	Name of the AMP	Activity
1432	4741	DRAMP18675	Peptide 4 (Mollusca/molluscs/mollusks, invertebrates, animals)	Antibacterial, Anti-Gram+, Anti-Gram-, Antimicrobial
1433	4742	DRAMP18676	Peptide 5 (Mollusca/molluscs/mollusks, invertebrates, animals)	Antibacterial, Anti-Gram+, Anti-Gram-, Antimicrobial
1434	4743	DRAMP18677	Peptide 6 (Mollusca/molluscs/mollusks, invertebrates, animals)	Antibacterial, Anti-Gram+, Anti-Gram-, Antimicrobial
1435	4744	DRAMP18678	Peptide 8 (Mollusca/molluscs/mollusks, invertebrates, animals)	Antibacterial, Anti-Gram+, Anti-Gram-, Antimicrobial
1436	4745	DRAMP18679	Peptide 9 (Mollusca/molluscs/mollusks, invertebrates, animals)	Antibacterial, Anti-Gram+, Anti-Gram-, Antimicrobial
1437	4759	DRAMP18693	Substance P (Mammals, animals)	Antimicrobial, Antibacterial, Antifungal, Anti-Gram+, Anti-Gram-,
1438	4760	DRAMP18694	substance P antagonist (Mammals, animals)	Antimicrobial, Antibacterial, Antifungal, Anti-Gram+, Anti-Gram-,
1439	4762	DRAMP18696	HLP1 (Lactotransferrin truncated peptide)	Antibacterial, Anti-Gram+, Anti-Gram-, Antimicrobial
1440	4763	DRAMP18697	HLP2 (Lactotransferrin truncated peptide)	Antibacterial, Anti-Gram+, Anti-Gram-, Antimicrobial
1441	4765	DRAMP18699	Pleurain-B1 (Frogs, amphibians, animals)	Antimicrobial, Antibacterial, Antifungal, Anti-Gram+, Anti- Gram-,
1442	4766	DRAMP18700	Pleurain-C1 (Frogs, amphibians, animals)	Antimicrobial, Antibacterial, Antifungal, Anti-Gram+, Anti-Gram-,
1443	4767	DRAMP18701	Pleurain-D4 (Frogs, amphibians, animals)	Antimicrobial, Antibacterial, Antifungal, Anti-Gram+, Anti-Gram-,
1444	4768	DRAMP18702	Pleurain-E1 (Frogs, amphibians, animals)	Antimicrobial, Antibacterial, Antifungal, Anti-Gram+, Anti-Gram-,
1445	4769	DRAMP18703	Pleurain-G1 (Frogs, amphibians, animals)	Antimicrobial, Antibacterial, Antifungal, Anti-Gram+, Anti-Gram-,
1446	4770	DRAMP18704	Pleurain-J1 (Frogs, amphibians, animals)	Antimicrobial, Antibacterial, Antifungal, Anti-Gram+, Anti-Gram-,
1447	4771	DRAMP18705	Pleurain-N1 (Frogs, amphibians, animals)	Antimicrobial, Antibacterial, Antifungal, Anti-Gram+, Anti- Gram-,
1448	4772	DRAMP18706	Pleurain-R1 (Frogs, amphibians, animals)	Antimicrobial, Antibacterial, Antifungal, Anti-Gram+, Anti- Gram-,
1449	4773	DRAMP18707	BACTENECIN 7 (bac 7, Pro-rich; bovine cathelicidin, cattle,	Antibacterial, Anti-Gram+, Anti-Gram-, Antimicrobial
1450	4774	DRAMP18708	Dermaseptin-S4 (DRS-S4, DS4; frog, amphibians, animals)	Antimicrobial, Antibacterial, Antifungal, Anti-Gram+, Anti-Gram-,
1451	4778	DRAMP18712	Styelin A (Tunicate, invertebrates, animals)	Antibacterial, Anti-Gram+, Anti-Gram-, Antimicrobial
1452	4779	DRAMP18713	Styelin B (Tunicate, invertebrates, animals)	Antibacterial, Anti-Gram+, Anti-Gram-, Antimicrobial
1453	4783	DRAMP18717	Charybdotoxin (Yellow scorpions, arachnids, Chelicerata,arthropods,	Antimicrobial, Antibacterial, Antifungal, Anti-Gram+, Anti-Gram-,
1454	4785	DRAMP18719	SAAP fraction 3 (Surfactant-associated anionic peptides; Asp- rich;	Antibacterial, Anti-Gram+, Anti-Gram-, Antimicrobial
1455	4787	DRAMP18721	Hinnavin I (Hin I; insects, arthropods, invertebrates, animals)	Antimicrobial, Antibacterial, Antifungal, Anti-Gram+, Anti-Gram-,
1456	4790	DRAMP18724	Oncorhyncin III (Oncorhyncin-3, histone-derived; fish, animals)	Antibacterial, Anti-Gram+, Anti-Gram-, Antimicrobial
1457	4791	DRAMP03024	Mastoparan B (MP-B; insects, arthropods, invertebrates, animals)	Antimicrobial, Antibacterial, Anti-Gram+, Anti-Gram-, Anti Mammalian cells, Anti-cancer
1458	4818	DRAMP01826	RV-23 (Frogs, amphibians, animals)	Antimicrobial, Antibacterial, Anti-Gram+, Anti-Gram-
1459	4820	DRAMP03025	Mastoparan M	Antimicrobial, Antibacterial, Anti-gram+, Anti-gram-
1460	4825	DRAMP03812	Pardaxin P-4 (Pardaxin P1a; Pardaxin Pa4)	Antimicrobial, Antibacterial, Anti-Gram+, Anti-Gram-, Cytotoxic
1461	4839	DRAMP20771	Acanthaporin (parasite, amoebozoa, protozoa, protists)	Antimicrobial, Antibacterial, Anti-Gram+, Anti-Gram-
1462	4840	DRAMP20772	cPcAMP1/26 (ciliate, Protists)	Antimicrobial, Antibacterial, Anti-Gram+, Anti-Gram-
1463	4842	DRAMP20776	HaA4 (beetles,insects,animals)	Antimicrobial, Antibacterial, Anti-Gram+, Anti-Gram-
1464	4843	DRAMP20777	Cath-BF	Antimicrobial, Antibacterial, Anti-Gram+, Anti-Gram-
1465	4844	DRAMP20778	Temporin-SHf (frogs,amphibians,animals)	Antimicrobial, Antibacterial, Anti-Gram+, Anti-Gram-, Antifungal
1466	4845	DRAMP20779	Halictine 1 (bees,insects,animals)	Antimicrobial, Antibacterial, Anti-Gram+, Anti-Gram-
1467	4846	DRAMP20780	Halictine 2 (bees,insects,animals)	Antimicrobial, Antibacterial, Anti-Gram+, Anti-Gram-
1468	4847	DRAMP20781	Panurgine 1(bees,insects,animals)	Antimicrobial, Antibacterial, Anti-Gram+, Anti-Gram-
1469	4848	DRAMP20782	Pleurain-D1 (Frogs,amphibians,animals)	Antimicrobial, Antibacterial, Anti-Gram+, Anti-Gram-
1470	4849	DRAMP20783	Pleurain-M1 (Frogs,amphibians,animals)	Antimicrobial, Antibacterial, Anti-Gram+, Anti-Gram-
1471	4850	DRAMP20784	Megin 1	Antimicrobial, Antibacterial, Anti-Gram+, Anti-Gram-, Antifungal
1472	4851	DRAMP20785	Megin 2	Antimicrobial, Antibacterial, Anti-Gram+, Anti-Gram-, Antifungal
1473	4852	DRAMP20786	mini-ChBac7.5N alpha	Antimicrobial, Antibacterial, Anti-Gram+, Anti-Gram-, Antifungal
1474	4853	DRAMP20787	mini-ChBac7.5N beta	Antimicrobial, Antibacterial, Anti-Gram+, Anti-Gram-, Antifungal
1475	4856	DRAMP20790	Cecropin B (Insects, arthropods, invertebrates, animals)	Antimicrobial, Antibacterial, Anti-Gram+, Anti-Gram-
1476	4858	DRAMP20792	Maculatin 1.1 (Frog, amphibians, animals)	Antimicrobial, Antibacterial, Anti-Gram+, Anti-Gram-
1477	4863	DRAMP20797	Uperin 3.6 (Toad, amphibians, animals)	Antimicrobial, Antibacterial, Anti-Gram+, Anti-Gram-
1478	4864	DRAMP20798	Lingual antimicrobial peptide (LAP, beta defensin, cattle, ruminant,	Antimicrobial, Antibacterial, Anti-Gram+, Anti-Gram-, Antifungal
1479	4867	DRAMP20801	Mussel Defensin MGD-1 (Mediterranean mussel defensin 1;	Antimicrobial, Antibacterial, Anti-Gram+, Anti-Gram-
1480	4868	DRAMP20802	CPF-AM1 (caerulein precursor fragment-AM1, frogs, amphibians,	Antimicrobial, Antibacterial, Anti-Gram+, Anti-Gram-
1481	4869	DRAMP20803	moronecidin-like peptide	Antimicrobial, Antibacterial, Anti-Gram+, Anti-Gram-, Antifungal
1482	4870	DRAMP20804	Al-hemocidins 2 (Hb-1 truncated peptide)	Antimicrobial, Antibacterial, Anti-Gram+, Anti-Gram-
1483	4871	DRAMP20805	Apo5 APOC164-88	Antimicrobial, Antibacterial, Anti-Gram+, Anti-Gram-
1484	4872	DRAMP20806	Apo6 APOC167-88	Antimicrobial, Antibacterial, Anti-Gram+, Anti-Gram-

S.no.	PepID	DRAMP_ID	Name of the AMP	Activity
1485	4873	DRAMP20807	A1P394-428	Antimicrobial, Antibacterial, Anti-Gram+, Anti-Gram-
1486	4874	DRAMP20808	RI21 (PMAP-36 peptide derivative)	Antimicrobial, Antibacterial, Anti-Gram+, Anti-Gram-, Antifungal
1487	4875	DRAMP20809	RI18 (PMAP-36 peptide derivative)	Antimicrobial, Antibacterial, Anti-Gram+, Anti-Gram-, Antifungal
1488	4876	DRAMP20810	TI15 (PMAP-36 peptide derivative)	Antimicrobial, Antibacterial, Anti-Gram+, Anti-Gram-, Antifungal
1489	4877	DRAMP20811	RI12 (PMAP-36 peptide derivative)	Antimicrobial, Antibacterial, Anti-Gram+, Anti-Gram-, Antifungal
1490	4878	DRAMP20812	K8	Antimicrobial, Antibacterial, Anti-Gram+, Anti-Gram-
1491	4879	DRAMP20813	L1K8	Antimicrobial, Antibacterial, Anti-Gram+, Anti-Gram-
1492	4880	DRAMP20814	S1K8	Antimicrobial, Antibacterial, Anti-Gram+, Anti-Gram-
1493	4881	DRAMP20815	F1K8	Antimicrobial, Antibacterial, Anti-Gram+, Anti-Gram-
1494	4882	DRAMP20816	K1K8	Antimicrobial, Antibacterial, Anti-Gram+, Anti-Gram-
1495	4883	DRAMP20817	RR12	Antimicrobial, Antibacterial, Anti-Gram+, Anti-Gram-
1496	4884	DRAMP20818	RR12Wpolar	Antimicrobial, Antibacterial, Anti-Gram+, Anti-Gram-
1497	4885	DRAMP20819	RR12Whydro	Antimicrobial, Antibacterial, Anti-Gram+, Anti-Gram-
1498	4886	DRAMP20820	FV7	Antimicrobial, Antibacterial, Anti-Gram+, Anti-Gram-
1499	4887	DRAMP20821	FV-LL (FV7 and LL(LL-37,(17-29)) hybrid peptide)	Antimicrobial, Antibacterial, Anti-Gram+, Anti-Gram-
1500	4888	DRAMP20822	FV-MA (FV7 and MA(Magainin 2 (9-21)) hybrid peptide)	Antimicrobial, Antibacterial, Anti-Gram+, Anti-Gram-
1501	4889	DRAMP20823	FV-CE (FV7 and CE(Cecropin A (1	Antimicrobial, Antibacterial, Anti-Gram+, Anti-Gram-
1502	4890	DRAMP20824	AM-CATH36	Antimicrobial, Antibacterial, Anti-Gram+, Anti-Gram-
1503	4892	DRAMP20826	AM-CATH21	Antimicrobial, Antibacterial, Anti-Gram+, Anti-Gram-
1504	4893	DRAMP20827	TB_L1FK	Antimicrobial, Antibacterial, Anti-Gram+, Anti-Gram-
1505	4894	DRAMP20828	TB_KKG6A	Antimicrobial, Antibacterial, Anti-Gram+, Anti-Gram-
1506	4895	DRAMP20831	IsCT1L1	Antimicrobial, Antibacterial, Anti-Gram+, Anti-Gram-
1507	4896	DRAMP20832	Polybia-MP1S-D8N	Antimicrobial, Antibacterial, Anti-Gram+, Anti-Gram-
1508	4897	DRAMP20833	[Pro3,DLeu9]TL(1) (Temporin L peptide derivative)	Antimicrobial, Antibacterial, Anti-Gram+, Anti-Gram-
1509	4898	DRAMP20834	PLS	Antimicrobial, Antibacterial, Anti-Gram+, Anti-Gram-
1510	4899	DRAMP20837	Pb-CATH1 Python bivittatus antimicrobial peptides peptide derivative	Antimicrobial, Antibacterial, Anti-Gram+, Anti-Gram-
1511	4900	DRAMP20838	Pb-CATH4 bivittatus antimicrobial peptides peptide derivative	Antimicrobial, Antibacterial, Anti-Gram+, Anti-Gram-
1512	4901	DRAMP20839	Xylopin	Antimicrobial, Antibacterial, Anti-Gram+, Anti-Gram-
1513	4902	DRAMP20841	C1b	Antimicrobial, Antibacterial, Anti-Gram+, Anti-Gram-
1514	4903	DRAMP20842	C1b(1-11)	Antimicrobial, Antibacterial, Anti-Gram+, Anti-Gram-
1515	4904	DRAMP20843	C1b(1-13)	Antimicrobial, Antibacterial, Anti-Gram+, Anti-Gram-
1516	4905	DRAMP20844	C1b(3-13)	Antimicrobial, Antibacterial, Anti-Gram+, Anti-Gram-
1517	4906	DRAMP20845	C1b(3-11)	Antimicrobial, Antibacterial, Anti-Gram+, Anti-Gram-
1518	4907	DRAMP20846	C1b(3-12)	Antimicrobial, Antibacterial, Anti-Gram+, Anti-Gram-
1519	4908	DRAMP20847	C1b(4-13)	Antimicrobial, Antibacterial, Anti-Gram+, Anti-Gram-
1520	4909	DRAMP20848	[K4]C1b(3-11)	Antimicrobial, Antibacterial, Anti-Gram+, Anti-Gram-
1521	4910	DRAMP20849	[R4]C1b(3-11)	Antimicrobial, Antibacterial, Anti-Gram+, Anti-Gram-
1522	4911	DRAMP20850	[K4,K10]C1b(3-13)	Antimicrobial, Antibacterial, Anti-Gram+, Anti-Gram-
1523	4912	DRAMP20851	[R4,R10]C1b(3-13)	Antimicrobial, Antibacterial, Anti-Gram+, Anti-Gram-
1524	4920	DRAMP20859	TT(1-24)	Antimicrobial, Antibacterial, Anti-Gram+, Anti-Gram-
1525	4921	DRAMP20860	TT(1-35)	Antimicrobial, Antibacterial, Anti-Gram+, Anti-Gram-
1526	4923	DRAMP20862	rtCATH2(5-40)	Antimicrobial, Antibacterial, Anti-Gram+, Anti-Gram-
1527	4924	DRAMP20863	rtCATH2(1-40)	Antimicrobial, Antibacterial, Anti-Gram+, Anti-Gram-
1528	4925	DRAMP20864	SF(18-45)	Antimicrobial, Antibacterial, Anti-Gram+, Anti-Gram-
1529	4926	DRAMP20865	the dimeric RRWQWR motif peptide molecule	Antimicrobial, Antibacterial, Anti-Gram+, Anti-Gram-
1530	4927	DRAMP20866	the tetrameric RRWQWR motif peptide molecule	Antimicrobial, Antibacterial, Anti-Gram+, Anti-Gram-
1531	4928	DRAMP20867	the palindromic RRWQWR motif peptide molecule	Antimicrobial, Antibacterial, Anti-Gram+, Anti-Gram-
1532	4929	DRAMP20868	H4	Antimicrobial, Antibacterial, Anti-Gram+, Anti-Gram-
1533	4930	DRAMP20869	Pal-ano-9 (Pal-anoplin peptide derivative)	Antimicrobial, Antibacterial, Anti-Gram+, Anti-Gram-, Antifungal
1534	4931	DRAMP20870	Pal-ano-8 (Pal-anoplin peptide derivative)	Antimicrobial, Antibacterial, Anti-Gram+, Anti-Gram-, Antifungal
1535	4932	DRAMP20871	Pal-ano-7 (Pal-anoplin peptide derivative)	Antimicrobial, Antibacterial, Anti-Gram+, Anti-Gram-, Antifungal
1536	4933	DRAMP20872	Pal-ano-6 (Pal-anoplin peptide derivative)	Antimicrobial, Antibacterial, Anti-Gram+, Anti-Gram-, Antifungal
1537	4934	DRAMP20873	Pal-ano-5 (Pal-anoplin peptide derivative)	Antimicrobial, Antibacterial, Anti-Gram+, Anti-Gram-, Antifungal

S.no.	PepID	DRAMP_ID	Name of the AMP	Activity
1538	4935	DRAMP20874	Chensinin-1b	Antimicrobial, Antibacterial, Anti-Gram+, Anti-Gram-
1539	4936	DRAMP20875	OA-C1b	Antimicrobial, Antibacterial, Anti-Gram+, Anti-Gram-
1540	4937	DRAMP20876	LA-C1b	Antimicrobial, Antibacterial, Anti-Gram+, Anti-Gram-
1541	4938	DRAMP20877	PA-C1b	Antimicrobial, Antibacterial, Anti-Gram+, Anti-Gram-
1542	4939	DRAMP20878	rVpDef	Antimicrobial, Antibacterial, Anti-Gram+, Anti-Gram-
1543	4940	DRAMP20879	DAN1	Antimicrobial, Antibacterial, Anti-Gram+, Anti-Gram-
1544	4941	DRAMP20880	DAN2	Antimicrobial, Antibacterial, Anti-Gram+, Anti-Gram-, Antifungal
1545	4944	DRAMP20883	Cath-A	Antimicrobial, Antibacterial, Anti-Gram+, Anti-Gram-
1546	4945	DRAMP20884	Cath-B	Antimicrobial, Antibacterial, Anti-Gram+, Anti-Gram-
1547	4948	DRAMP20887	NCP-2 (CTX-1 peptide derivative)	Antimicrobial, Antibacterial, Anti-Gram+, Anti-Gram-, Antifungal
1548	4949	DRAMP20888	NCP-3 (CTX-1 peptide derivative)	Antimicrobial, Antibacterial, Anti-Gram+, Anti-Gram-, Antifungal
1549	4954	DRAMP20893	I16A	Antimicrobial, Antibacterial, Anti-Gram+, Anti-Gram-, Antifungal
1550	4955	DRAMP20894	L19H/I20H	Antimicrobial, Antibacterial, Anti-Gram+, Anti-Gram-, Antifungal
1551	4956	DRAMP20895	F1A/I2A	Antimicrobial, Antibacterial, Anti-Gram+, Anti-Gram-, Antifungal
1552	4961	DRAMP20900	A12I/A15I	Antimicrobial, Antibacterial, Anti-Gram+, Anti-Gram-, Antifungal
1553	4962	DRAMP20901	A12V/A15H	Antimicrobial, Antibacterial, Anti-Gram+, Anti-Gram-, Antifungal
1554	4964	DRAMP20903	dC2	Antimicrobial, Antibacterial, Anti-Gram+, Anti-Gram-, Antifungal
1555	4965	DRAMP20904	R18S/R21H	Antimicrobial, Antibacterial, Anti-Gram+, Anti-Gram-, Antifungal
1556	4967	DRAMP20906	dN2	Antimicrobial, Antibacterial, Anti-Gram+, Anti-Gram-, Antifungal
1557	4968	DRAMP20907	dN4	Antimicrobial, Antibacterial, Anti-Gram+, Anti-Gram-, Antifungal
1558	4971	DRAMP20910	RN7-IN7(designed based on indolicidin and ranalexin)	Antimicrobial, Antibacterial, Anti-Gram+, Anti-Gram-
1559	4973	DRAMP20912	RN7-IN9(designed based on indolicidin and ranalexin)	Antimicrobial, Antibacterial, Anti-Gram+, Anti-Gram-
1560	4974	DRAMP20913	Myxinidin (G1)	Antimicrobial, Antibacterial, Anti-Gram+, Anti-Gram-
1561	4975	DRAMP20914	Myxinidin (I2)	Antimicrobial, Antibacterial, Anti-Gram+, Anti-Gram-
1562	4976	DRAMP20915	Myxinidin (H3)	Antimicrobial, Antibacterial, Anti-Gram+, Anti-Gram-
1563	4977	DRAMP20916	Myxinidin (D4)	Antimicrobial, Antibacterial, Anti-Gram+, Anti-Gram-
1564	4978	DRAMP20917	Myxinidin (I5)	Antimicrobial, Antibacterial, Anti-Gram+, Anti-Gram-
1565	4979	DRAMP20918	Myxinidin (L6)	Antimicrobial, Antibacterial, Anti-Gram+, Anti-Gram-
1566	4980	DRAMP20919	Myxinidin (K7)	Antimicrobial, Antibacterial, Anti-Gram+, Anti-Gram-
1567	4981	DRAMP20920	Myxinidin (Y8)	Antimicrobial, Antibacterial, Anti-Gram+, Anti-Gram-
1568	4982	DRAMP20921	Myxinidin (G9)	Antimicrobial, Antibacterial, Anti-Gram+, Anti-Gram-
1569	4983	DRAMP20922	Myxinidin (K10)	Antimicrobial, Antibacterial, Anti-Gram+, Anti-Gram-
1570	4984	DRAMP20923	Myxinidin (P11)	Antimicrobial, Antibacterial, Anti-Gram+, Anti-Gram-
1571	4985	DRAMP20924	Myxinidin (S12)	Antimicrobial, Antibacterial, Anti-Gram+, Anti-Gram-
1572	4986	DRAMP20925	MH3R	Antimicrobial, Antibacterial, Anti-Gram+, Anti-Gram-
1573	4987	DRAMP20926	IN1(designed based on indolicidin and ranalexin)	Antimicrobial, Antibacterial, Anti-Gram+, Anti-Gram-
1574	4988	DRAMP20927	IN2(designed based on indolicidin and ranalexin)	Antimicrobial, Antibacterial, Anti-Gram+, Anti-Gram-
1575	4989	DRAMP20928	IN3(designed based on indolicidin and ranalexin)	Antimicrobial, Antibacterial, Anti-Gram+, Anti-Gram-
1576	4990	DRAMP20929	RN7-IN6(designed based on indolicidin and ranalexin)	Antimicrobial, Antibacterial, Anti-Gram+, Anti-Gram-
1577	4991	DRAMP20930	BP100-Ala-NH-C16H33	Antimicrobial, Antibacterial, Anti-Gram+, Anti-Gram-
1578	4995	DRAMP20935	Macropin 1(solitary bee, insects, animals)	Antimicrobial, Antibacterial, Anti-Gram+, Anti-Gram-, Antifungal
1579	4996	DRAMP20936	ΔPb-CATH1	Antimicrobial, Antibacterial, Anti-Gram+, Anti-Gram-
1580	4997	DRAMP20937	Pb-CATH3	Antimicrobial, Antibacterial, Anti-Gram+, Anti-Gram-
1581	4998	DRAMP20938	Cbf-14	Antimicrobial, Antibacterial, Anti-Gram+, Anti-Gram-
1582	4999	DRAMP20939	D-Cbf-14	Antimicrobial, Antibacterial, Anti-Gram+, Anti-Gram-
1583	5001	DRAMP20941	[Pro3,DLeu9]TL(8) (Temporin L peptide derivative)	Antimicrobial, Antibacterial, Anti-Gram+, Anti-Gram-, Antifungal
1584	5002	DRAMP20942	[Pro3,DLeu9]TL(9) (Temporin L peptide derivative)	Antimicrobial, Antibacterial, Anti-Gram+, Anti-Gram-, Antifungal
1585	5003	DRAMP20943	[Pro3,DLeu9]TL(10) (Temporin L peptide derivative)	Antimicrobial, Antibacterial, Anti-Gram+, Anti-Gram-, Antifungal
1586	5004	DRAMP20944	[Pro3,DLeu9]TL(11) (Temporin L peptide derivative)	Antimicrobial, Antibacterial, Anti-Gram+, Anti-Gram-, Antifungal
1587	5005	DRAMP20945	Recombinant Cecropin A (1–8)–LL37 (17–30) (C–L)	Antimicrobial, Antibacterial, Anti-Gram+, Anti-Gram-
1588	5014	DRAMP20955	L31-P113	Antimicrobial, Antibacterial, Anti-Gram+, Anti-Gram-, Antifungal
1589	5015	DRAMP20956	AL32-P113	Antimicrobial, Antibacterial, Anti-Gram+, Anti-Gram-, Antifungal
1590	5016	DRAMP20957	StigA6	Antimicrobial, Antibacterial, Anti-Gram+, Anti-Gram-, Antifungal, Antiparasitic, Antiproliferative

S.no.	PepID	DRAMP_ID	Name of the AMP	Activity
1591	5017	DRAMP20958	StigA16	Antimicrobial, Antibacterial, Anti-Gram+, Anti-Gram-, Antifungal, Antiparasitic, Antiproliferative
1592	5021	DRAMP20963	Cp1 alpha s1-casein peptide derivative	Antimicrobial, Antibacterial, Anti-Gram+, Anti-Gram-, low hemolytic and toxic effects
1593	5022	DRAMP20964	Synthesized Cecropin A (1–8)–LL37 (17–30) (C–L)	Antimicrobial, Antibacterial, Anti-Gram+, Anti-Gram-
1594	5023	DRAMP20965	LPcin-YK3 (bovine cathelicidin, cattle, ruminant, mammals, animals)	Antimicrobial, Antibacterial, Anti-Gram+, Anti-Gram-, Antifungal
1595	5024	DRAMP20966	andricin B (Andrias davidianus, Amphibians, Animals)	Antimicrobial, Antibacterial, Anti-Gram+, Anti-Gram-, Antifungal
1596	5025	DRAMP20967	andricin 01 (Andrias davidianus, Amphibians, Animals)	Antimicrobial, Antibacterial, Anti-Gram+, Anti-Gram-
1597	5026	DRAMP20968	Catesbeianin-1 (Ranidae, Anura, Amphibia, Animals)	Antimicrobial, Antibacterial, Anti-Gram+, Anti-Gram-
1598	5027	DRAMP20969	HJH-1 (bovine cathelicidin, cattle, ruminant, mammals, animals)	Antimicrobial, Antibacterial, Anti-Gram+, Anti-Gram-, Antifungal
1599	5028	DRAMP20970	P3 (bovine cathelicidin, cattle, ruminant, mammals, animals)	Antimicrobial, Antibacterial, Anti-Gram+, Anti-Gram-, Antifungal
1600	5029	DRAMP20971	JH-0 (Derived from P3)	Antimicrobial, Antibacterial, Anti-Gram+, Anti-Gram-
1601	5030	DRAMP20972	JH-1 (Derived from P3)	Antimicrobial, Antibacterial, Anti-Gram+, Anti-Gram-
1602	5031	DRAMP20973	JH-2 (Derived from P3)	Antimicrobial, Antibacterial, Anti-Gram+, Anti-Gram-, Antifungal
1603	5032	DRAMP20974	JH-3 (Derived from P3)	Antimicrobial, Antibacterial, Anti-Gram+, Anti-Gram-, Antifungal
1604	5033	DRAMP20975	OH-CM6 (Derived from OH-CATH30)	Antimicrobial, Antibacterial, Anti-Gram+, Anti-Gram-
1605	5034	DRAMP20976	adevonin (Derived from Adenanthera pavonina trypsin inhibitor	Antimicrobial, Antibacterial, Anti-Gram+, Anti-Gram-
1606	5035	DRAMP20977	Anoplin-1 (Derived from Anoplin)	Antimicrobial, Antibacterial, Anti-Gram+, Anti-Gram-
1607	5036	DRAMP20978	Anoplin-2 (Derived from Anoplin)	Antimicrobial, Antibacterial, Anti-Gram+, Anti-Gram-
1608	5037	DRAMP20979	Anoplin-3 (Derived from Anoplin)	Antimicrobial, Antibacterial, Anti-Gram+, Anti-Gram-
1609	5038	DRAMP20980	Anoplin-4 (Derived from Anoplin)	Antimicrobial, Antibacterial, Anti-Gram+, Anti-Gram-
1610	5039	DRAMP20981	CPF-C1 (Frogs, Amphibians, Animals)	Antimicrobial, Antibacterial, Anti-Gram+, Anti-Gram-
1611	5040	DRAMP20982	CPF-1 (Derived from CPF-C1)	Antimicrobial, Antibacterial, Anti-Gram+, Anti-Gram-
1612	5041	DRAMP20983	CPF-2 (Derived from CPF-C1)	Antimicrobial, Antibacterial, Anti-Gram+, Anti-Gram-
1613	5042	DRAMP20984	CPF-3 (Derived from CPF-C1)	Antimicrobial, Antibacterial, Anti-Gram+, Anti-Gram-
1614	5043	DRAMP20985	CPF-4 (Derived from CPF-C1)	Antimicrobial, Antibacterial, Anti-Gram+, Anti-Gram-
1615	5044	DRAMP20986	CPF-5 (Derived from CPF-C1)	Antimicrobial, Antibacterial, Anti-Gram+, Anti-Gram-
1616	5045	DRAMP20987	CPF-6 (Derived from CPF-C1)	Antimicrobial, Antibacterial, Anti-Gram+, Anti-Gram-
1617	5046	DRAMP20988	CPF-7 (Derived from CPF-C1)	Antimicrobial, Antibacterial, Anti-Gram+, Anti-Gram-
1618	5047	DRAMP20989	CPF-8 (Derived from CPF-C1)	Antimicrobial, Antibacterial, Anti-Gram+, Anti-Gram-
1619	5048	DRAMP20990	CPF-9 (Derived from CPF-C1)	Antimicrobial, Antibacterial, Anti-Gram+, Anti-Gram-
1620	5049	DRAMP20991	CPF-10 (Derived from CPF-C1)	Antimicrobial, Antibacterial, Anti-Gram+, Anti-Gram-
1621	5050	DRAMP20992	CPF-11 (Derived from CPF-C1)	Antimicrobial, Antibacterial, Anti-Gram+, Anti-Gram-
1622	5051	DRAMP20993	CPF-12 (Derived from CPF-C1)	Antimicrobial, Antibacterial, Anti-Gram+, Anti-Gram-
1623	5052	DRAMP20994	anoplin analog 4	Antimicrobial, Antibacterial, Anti-Gram+, Anti-Gram-, Antifungal
1624	5053	DRAMP20995	anoplin analog 5	Antimicrobial, Antibacterial, Anti-Gram+, Anti-Gram-, Antifungal
1625	5054	DRAMP20996	anoplin analog 6	Antimicrobial, Antibacterial, Anti-Gram+, Anti-Gram-, Antifungal
1626	5055	DRAMP20997	anoplin analog 7	Antimicrobial, Antibacterial, Anti-Gram+, Anti-Gram-
1627	5056	DRAMP20998	anoplin analog 8	Antimicrobial, Antibacterial, Anti-Gram+, Anti-Gram-
1628	5057	DRAMP20999	anoplin analog 9	Antimicrobial, Antibacterial, Anti-Gram+, Anti-Gram-
1629	5058	DRAMP21000	cGm (Derived from Gm)	Antimicrobial, Antibacterial, Anti-Gram+, Anti-Gram-, Antifungal, Antitumor
1630	5059	DRAMP21001	[Y7W]cGm (Derived from Gm)	Antimicrobial, Antibacterial, Anti-Gram+, Anti-Gram-, Antitumor
1631	5060	DRAMP21002	[Y14W]cGm (Derived from Gm)	Antimicrobial, Antibacterial, Anti-Gram+, Anti-Gram-, Antitumor
1632	5061	DRAMP21003	[K8R]cGm (Derived from Gm)	Antimicrobial, Antibacterial, Anti-Gram+, Anti-Gram-, Antitumor
1633	5062	DRAMP21004	[Y7W, K8R, Y14W]cGm (Derived from Gm)	Antimicrobial, Antibacterial, Anti-Gram+, Anti-Gram-, Antifungal, Antitumor
1634	5063	DRAMP21005	[R4A, R18A]cGm (Derived from Gm)	Antimicrobial, Antibacterial, Anti-Gram+, Anti-Gram-, Antifungal, Antitumor
1635	5064	DRAMP21006	[G1K, K8R]cGm (Derived from Gm)	Antimicrobial, Antibacterial, Anti-Gram+, Anti-Gram-, Antifungal, Antitumor
1636	5065	DRAMP21007	[C/U]cGm (Derived from Gm)	Antimicrobial, Antibacterial, Anti-Gram+, Anti-Gram-, Antifungal, Antitumor
1637	5066	DRAMP21008	[L5W]cGm (Derived from Gm)	Antimicrobial, Antibacterial, Anti-Gram+, Anti-Gram-, Antitumor
1638	5067	DRAMP21009	[D-P L-P]cGm (Derived from Gm)	Antimicrobial, Antibacterial, Anti-Gram+, Anti-Gram-, Antifungal, Antitumor
1639	5068	DRAMP21010	[G1K, L5Y, K8R]cGm (Derived from Gm)	Antimicrobial, Antibacterial, Anti-Gram+, Anti-Gram-, Antifungal, Antitumor
1640	5069	DRAMP21011	[C/U, G1K, L5Y, K8R]cGm (Derived from Gm)	Antimicrobial, Antibacterial, Anti-Gram+, Anti-Gram-, Antifungal, Antitumor
1641	5070	DRAMP21012	NK-2 (Mammals, Animals)	Antimicrobial, Antibacterial, Anti-Gram+, Anti-Gram-, Antifungal, Antitumor
1642	5071	DRAMP21013	NK-pro (Derived from NK-2)	Antimicrobial, Antibacterial, Anti-Gram+, Anti-Gram-, Antifungal, Antitumor
1643	5072	DRAMP21014	NK-dpro (Derived from NK-2)	Antimicrobial, Antibacterial, Anti-Gram+, Anti-Gram-, Antifungal, Antitumor

S.no.	PepID	DRAMP_ID	Name of the AMP	Activity
1644	5073	DRAMP21015	A (A1R) (Derived from AR-23)	Antimicrobial, Antibacterial, Anti-Gram+, Anti-Gram-
1645	5074	DRAMP21016	A (A8R) (Derived from AR-23)	Antimicrobial, Antibacterial, Anti-Gram+, Anti-Gram-
1646	5075	DRAMP21017	A (I17K) (Derived from AR-23)	Antimicrobial, Antibacterial, Anti-Gram+, Anti-Gram-
1647	5076	DRAMP21018	A (I17R) (Derived from AR-23)	Antimicrobial, Antibacterial, Anti-Gram+, Anti-Gram-
1648	5077	DRAMP21019	A (A1R, A8R) (Derived from AR-23)	Antimicrobial, Antibacterial, Anti-Gram+, Anti-Gram-
1649	5078	DRAMP21020	A (A1R, I17K) (Derived from AR-23)	Antimicrobial, Antibacterial, Anti-Gram+, Anti-Gram-
1650	5079	DRAMP21021	A (A8R, I17K) (Derived from AR-23)	Antimicrobial, Antibacterial, Anti-Gram+, Anti-Gram-
1651	5080	DRAMP21022	A (A1R, A8R, I17K) (Derived from AR-23)	Antimicrobial, Antibacterial, Anti-Gram+, Anti-Gram-
1652	5081	DRAMP21023	A (A1R, A8R, I17R) (Derived from AR-23)	Antimicrobial, Antibacterial, Anti-Gram+, Anti-Gram-
1653	5082	DRAMP21024	Stigmurin (Tityus, Scorpionida, Arachnida)	Antimicrobial, Antibacterial, Anti-Gram+, Anti-Gram-, Antifungal
1654	5083	DRAMP21025	StigA25 (Derived from Stigmurin)	Antimicrobial, Antibacterial, Anti-Gram+, Anti-Gram-, Antifungal
1655	5084	DRAMP21026	StigA31 (Derived from Stigmurin)	Antimicrobial, Antibacterial, Anti-Gram+, Anti-Gram-, Antifungal
1656	5085	DRAMP21027	K5, 17-DPS3 (Derived from dermaseptin-PS3 (DPS3))	Antimicrobial, Antibacterial, Anti-Gram+, Anti-Gram-, Antifungal
1657	5086	DRAMP21028	L10, 11-DPS3 (Derived from dermaseptin-PS3 (DPS3))	Antimicrobial, Antibacterial, Anti-Gram+, Anti-Gram-, Antifungal
1658	5087	DRAMP21029	D5R (Derived from HD5)	Antimicrobial, Antibacterial, Anti-Gram+, Anti-Gram-, Antifungal
1659	5088	DRAMP21030	D5r (Derived from HD5)	Antimicrobial, Antibacterial, Anti-Gram+, Anti-Gram-, Antifungal
1660	5089	DRAMP21031	MyD5R (Derived from HD5)	Antimicrobial, Antibacterial, Anti-Gram+, Anti-Gram-, Antifungal
1661	5090	DRAMP21032	MyD5r (Derived from HD5)	Antimicrobial, Antibacterial, Anti-Gram+, Anti-Gram-, Antifungal
1662	5091	DRAMP21033	LaD5R (Derived from HD5)	Antimicrobial, Antibacterial, Anti-Gram+, Anti-Gram-, Antifungal
1663	5092	DRAMP21034	LaD5r (Derived from HD5)	Antimicrobial, Antibacterial, Anti-Gram+, Anti-Gram-, Antifungal
1664	5093	DRAMP21035	AC-UM-14W (De novo synthesis)	Antimicrobial, Antibacterial, Anti-Gram+, Anti-Gram-
1665	5094	DRAMP21036	PapMA (Derived from Papiliocin and Magainin 2)	Antimicrobial, Antibacterial, Anti-Gram+, Anti-Gram-
1666	5095	DRAMP21037	PapMA-k (Derived from Papiliocin and Magainin 2)	Antimicrobial, Antibacterial, Anti-Gram+, Anti-Gram-
1667	5096	DRAMP21038	analog 1 (Derived from Ib-AMP1)	Antimicrobial, Antibacterial, Anti-Gram+, Anti-Gram-
1668	5097	DRAMP21039	analog 2 (Derived from Ib-AMP2)	Antimicrobial, Antibacterial, Anti-Gram+, Anti-Gram-
1669	5098	DRAMP21040	analog 3 (Derived from Ib-AMP2)	Antimicrobial, Antibacterial, Anti-Gram+, Anti-Gram-
1670	5099	DRAMP21041	analog 4 (Derived from Ib-AMP2)	Antimicrobial, Antibacterial, Anti-Gram+, Anti-Gram-
1671	5100	DRAMP21042	A2 (Derived from Indolicidin (IN))	Antimicrobial, Antibacterial, Anti-Gram+, Anti-Gram-
1672	5101	DRAMP21043	A3 (Derived from Indolicidin (IN))	Antimicrobial, Antibacterial, Anti-Gram+, Anti-Gram-
1673	5102	DRAMP21044	A4 (Derived from Indolicidin (IN))	Antimicrobial, Antibacterial, Anti-Gram+, Anti-Gram-
1674	5103	DRAMP21045	A5 (Derived from Indolicidin (IN))	Antimicrobial, Antibacterial, Anti-Gram+, Anti-Gram-
1675	5104	DRAMP21046	A6 (Derived from Indolicidin (IN))	Antimicrobial, Antibacterial, Anti-Gram+, Anti-Gram-
1676	5105	DRAMP21047	A7 (Derived from Indolicidin (IN))	Antimicrobial, Antibacterial, Anti-Gram+, Anti-Gram-
1677	5106	DRAMP21048	peptide 6 (Derived from seq2)	Antimicrobial, Antibacterial, Anti-Gram+, Anti-Gram-
1678	5107	DRAMP21049	peptide 6.2 (Derived from seq2)	Antimicrobial, Antibacterial, Anti-Gram+, Anti-Gram-
1679	5108	DRAMP21050	TP1[K1A] (Derived from TP1)	Antimicrobial, Antibacterial, Anti-Gram+, Anti-Gram-, Antifungal
1680	5109	DRAMP21051	TP1[W2A] (Derived from TP1)	Antimicrobial, Antibacterial, Anti-Gram+, Anti-Gram-, Antifungal
1681	5110	DRAMP21052	TP1[C3A, C16S] (Derived from TP1)	Antimicrobial, Antibacterial, Anti-Gram+, Anti-Gram-, Antifungal
1682	5111	DRAMP21053	TP1[F4A] (Derived from TP1)	Antimicrobial, Antibacterial, Anti-Gram+, Anti-Gram-, Antifungal
1683	5112	DRAMP21054	TP1[R5A] (Derived from TP1)	Antimicrobial, Antibacterial, Anti-Gram+, Anti-Gram-, Antifungal
1684	5113	DRAMP21055	TP1[V6A] (Derived from TP1)	Antimicrobial, Antibacterial, Anti-Gram+, Anti-Gram-, Antifungal
1685	5114	DRAMP21056	TP1[C7A, C12S] (Derived from TP1)	Antimicrobial, Antibacterial, Anti-Gram+, Anti-Gram-, Antifungal
1686	5115	DRAMP21057	TP1[Y8A] (Derived from TP1)	Antimicrobial, Antibacterial, Anti-Gram+, Anti-Gram-, Antifungal
1687	5116	DRAMP21058	TP1[R9A] (Derived from TP1)	Antimicrobial, Antibacterial, Anti-Gram+, Anti-Gram-, Antifungal
1688	5117	DRAMP21059	TP1[G10A] (Derived from TP1)	Antimicrobial, Antibacterial, Anti-Gram+, Anti-Gram-, Antifungal
1689	5118	DRAMP21060	TP1[I11A] (Derived from TP1)	Antimicrobial, Antibacterial, Anti-Gram+, Anti-Gram-, Antifungal
1690	5119	DRAMP21061	TP1[C7S, C12A] (Derived from TP1)	Antimicrobial, Antibacterial, Anti-Gram+, Anti-Gram-, Antifungal
1691	5120	DRAMP21062	TP1[Y13A] (Derived from TP1)	Antimicrobial, Antibacterial, Anti-Gram+, Anti-Gram-, Antifungal
1692	5121	DRAMP21063	TP1[R14A] (Derived from TP1)	Antimicrobial, Antibacterial, Anti-Gram+, Anti-Gram-, Antifungal
1693	5122	DRAMP21064	TP1[R15A] (Derived from TP1)	Antimicrobial, Antibacterial, Anti-Gram+, Anti-Gram-, Antifungal
1694	5123	DRAMP21065	TP1[C3S, C16A] (Derived from TP1)	Antimicrobial, Antibacterial, Anti-Gram+, Anti-Gram-, Antifungal
1695	5124	DRAMP21066	TP1[R17A] (Derived from TP1)	Antimicrobial, Antibacterial, Anti-Gram+, Anti-Gram-, Antifungal
1696	5125	DRAMP21067	TP1[C3A, C16A] (Derived from TP1)	Antimicrobial, Antibacterial, Anti-Gram+, Anti-Gram-, Antifungal

S.no.	PepID	DRAMP_ID	Name of the AMP	Activity
1697	5126	DRAMP21068	TP1[C7A, C12A] (Derived from TP1)	Antimicrobial, Antibacterial, Anti-Gram+, Anti-Gram-, Antifungal
1698	5127	DRAMP21069	TP1[C3A, C7A, C12A, C16A] (Derived from TP1)	Antimicrobial, Antibacterial, Anti-Gram+, Anti-Gram-, Antifungal
1699	5128	DRAMP21070	TP1[V6R, R9A] (Derived from TP1)	Antimicrobial, Antibacterial, Anti-Gram+, Anti-Gram-, Antifungal
1700	5129	DRAMP21071	TP1[K1R] (Derived from TP1)	Antimicrobial, Antibacterial, Anti-Gram+, Anti-Gram-, Antifungal
1701	5130	DRAMP21072	TP1[F4G] (Derived from TP1)	Antimicrobial, Antibacterial, Anti-Gram+, Anti-Gram-, Antifungal
1702	5131	DRAMP21073	TP1[F4S] (Derived from TP1)	Antimicrobial, Antibacterial, Anti-Gram+, Anti-Gram-, Antifungal
1703	5132	DRAMP21074	TP1[Y8G] (Derived from TP1)	Antimicrobial, Antibacterial, Anti-Gram+, Anti-Gram-, Antifungal
1704	5133	DRAMP21075	TP1[I11G] (Derived from TP1)	Antimicrobial, Antibacterial, Anti-Gram+, Anti-Gram-, Antifungal
1705	5134	DRAMP21076	TP1[F4A, Y8A, I11A] (Derived from TP1)	Antimicrobial, Antibacterial, Anti-Gram+, Anti-Gram-, Antifungal
1706	5135	DRAMP21077	TP1[-R5, R17G] (Derived from TP1)	Antimicrobial, Antibacterial, Anti-Gram+, Anti-Gram-, Antifungal
1707	5136	DRAMP21078	TP1[K1A, F4A] (Derived from TP1)	Antimicrobial, Antibacterial, Anti-Gram+, Anti-Gram-, Antifungal
1708	5137	DRAMP21079	TP1[K1A, Y8A] (Derived from TP1)	Antimicrobial, Antibacterial, Anti-Gram+, Anti-Gram-, Antifungal
1709	5138	DRAMP21080	TP1[K1A, I11A] (Derived from TP1)	Antimicrobial, Antibacterial, Anti-Gram+, Anti-Gram-, Antifungal
1710	5139	DRAMP21081	TP1[R9A, R17A] (Derived from TP1)	Antimicrobial, Antibacterial, Anti-Gram+, Anti-Gram-, Antifungal
1711	5140	DRAMP21082	ccTP 3 (Derived from TP2)	Antimicrobial, Antibacterial, Anti-Gram+, Anti-Gram-, Antifungal
1712	5141	DRAMP21083	ccTP 5 (Derived from TP2)	Antimicrobial, Antibacterial, Anti-Gram+, Anti-Gram-, Antifungal
1713	5142	DRAMP21084	ccTP 6 (Derived from TP2)	Antimicrobial, Antibacterial, Anti-Gram+, Anti-Gram-, Antifungal
1714	5143	DRAMP21085	PRW4 (PR) (Derived from PMAP-36)	Antimicrobial, Antibacterial, Anti-Gram+, Anti-Gram-
1715	5144	DRAMP21086	PR-FO (Derived from PRW4)	Antimicrobial, Antibacterial, Anti-Gram+, Anti-Gram-
1716	5145	DRAMP21087	PR-PG (Derived from PRW4)	Antimicrobial, Antibacterial, Anti-Gram+, Anti-Gram-
1717	5146	DRAMP21088	PR-TR (Derived from PRW4)	Antimicrobial, Antibacterial, Anti-Gram+, Anti-Gram-
1718	5147	DRAMP21089	C4 (Derived from PRW4)	Antimicrobial, Antibacterial, Anti-Gram+, Anti-Gram-
1719	5148	DRAMP21090	D4 (Derived from PRW4)	Antimicrobial, Antibacterial, Anti-Gram+, Anti-Gram-
1720	5149	DRAMP21091	I4 (Derived from PRW4)	Antimicrobial, Antibacterial, Anti-Gram+, Anti-Gram-
1721	5150	DRAMP21092	P4 (Derived from PRW4)	Antimicrobial, Antibacterial, Anti-Gram+, Anti-Gram-
1722	5151	DRAMP21093	PRW4-d (Derived from PRW4)	Antimicrobial, Antibacterial, Anti-Gram+, Anti-Gram-
1723	5152	DRAMP21094	PRW4-R (Derived from PRW4)	Antimicrobial, Antibacterial, Anti-Gram+, Anti-Gram-
1724	5153	DRAMP21095	IR1 (Derived from PG-1)	Antimicrobial, Antibacterial, Anti-Gram+, Anti-Gram-
1725	5154	DRAMP21096	IR2 (Derived from PG-1)	Antimicrobial, Antibacterial, Anti-Gram+, Anti-Gram-
1726	5155	DRAMP21097	FR1 (Derived from PG-1)	Antimicrobial, Antibacterial, Anti-Gram+, Anti-Gram-
1727	5156	DRAMP21098	FR2 (Derived from PG-1)	Antimicrobial, Antibacterial, Anti-Gram+, Anti-Gram-
1728	5157	DRAMP21099	WR1 (Derived from PG-1)	Antimicrobial, Antibacterial, Anti-Gram+, Anti-Gram-
1729	5158	DRAMP21100	WR2 (Derived from PG-1)	Antimicrobial, Antibacterial, Anti-Gram+, Anti-Gram-
1730	5159	DRAMP21101	PR1 (Derived from PG-1)	Antimicrobial, Antibacterial, Anti-Gram+, Anti-Gram-
1731	5160	DRAMP21102	PR2 (Derived from PG-1)	Antimicrobial, Antibacterial, Anti-Gram+, Anti-Gram-
1732	5161	DRAMP21165	HYL-11 (Derived from HYL)	Antimicrobial, Antibacterial, Anti-Gram+, Anti-Gram-, Antifungal
1733	5162	DRAMP21166	HYL-12 (Derived from HYL)	Antimicrobial, Antibacterial, Anti-Gram+, Anti-Gram-, Antifungal
1734	5163	DRAMP21164	HYL-10 (Derived from HYL)	Antimicrobial, Antibacterial, Anti-Gram+, Anti-Gram-, Antifungal
1735	5164	DRAMP21158	HYL-4 (Derived from HYL)	Antimicrobial, Antibacterial, Anti-Gram+, Anti-Gram-, Antifungal
1736	5165	DRAMP21159	HYL-5 (Derived from HYL)	Antimicrobial, Antibacterial, Anti-Gram+, Anti-Gram-, Antifungal
1737	5166	DRAMP21160	HYL-6 (Derived from HYL)	Antimicrobial, Antibacterial, Anti-Gram+, Anti-Gram-, Antifungal
1738	5167	DRAMP21161	HYL-7 (Derived from HYL)	Antimicrobial, Antibacterial, Anti-Gram+, Anti-Gram-, Antifungal
1739	5168	DRAMP21162	HYL-8 (Derived from HYL)	Antimicrobial, Antibacterial, Anti-Gram+, Anti-Gram-, Antifungal
1740	5169	DRAMP21163	HYL-9 (Derived from HYL)	Antimicrobial, Antibacterial, Anti-Gram+, Anti-Gram-, Antifungal
1741	5170	DRAMP21157	HYL-3 (Derived from HYL)	Antimicrobial, Antibacterial, Anti-Gram+, Anti-Gram-, Antifungal
1742	5171	DRAMP21156	HYL-2 (Derived from HYL)	Antimicrobial, Antibacterial, Anti-Gram+, Anti-Gram-, Antifungal
1743	5172	DRAMP21155	HYL-1 (Derived from HYL)	Antimicrobial, Antibacterial, Anti-Gram+, Anti-Gram-, Antifungal
1744	5173	DRAMP21154	HYL (Bee, Insecta, Animals)	Antimicrobial, Antibacterial, Anti-Gram+, Anti-Gram-, Antifungal
1745	5174	DRAMP21153	KR-12-a8 (Derived from KR-12)	Antimicrobial, Antibacterial, Anti-Gram+, Anti-Gram-
1746	5175	DRAMP21151	KR-12-a6 (Derived from KR-12)	Antimicrobial, Antibacterial, Anti-Gram+, Anti-Gram-
1747	5176	DRAMP21150	KR-12-a5 (Derived from KR-12)	Antimicrobial, Antibacterial, Anti-Gram+, Anti-Gram-
1748	5177	DRAMP21152	KR-12-a7 (Derived from KR-12)	Antimicrobial, Antibacterial, Anti-Gram+, Anti-Gram-
1749	5178	DRAMP21149	KR-12-a4 (Derived from KR-12)	Antimicrobial, Antibacterial, Anti-Gram+, Anti-Gram-

S.no.	PepID	DRAMP_ID	Name of the AMP	Activity
1750	5179	DRAMP21146	KR-12-a1 (Derived from KR-12)	Antimicrobial, Antibacterial, Anti-Gram+, Anti-Gram-
1751	5180	DRAMP21148	KR-12-a3 (Derived from KR-12)	Antimicrobial, Antibacterial, Anti-Gram+, Anti-Gram-
1752	5181	DRAMP21147	KR-12-a2 (Derived from KR-12)	Antimicrobial, Antibacterial, Anti-Gram+, Anti-Gram-
1753	5182	DRAMP21145	Myxinidin3 (Derived from Myxinidin)	Antimicrobial, Antibacterial, Anti-Gram+, Anti-Gram-
1754	5183	DRAMP21142	AMP2041 (De novo synthesis)	Antimicrobial, Antibacterial, Anti-Gram+, Anti-Gram-
1755	5184	DRAMP21141	AMP126 (De novo synthesis)	Antimicrobial, Antibacterial, Anti-Gram+, Anti-Gram-
1756	5185	DRAMP21144	Myxinidin2 (Derived from Myxinidin)	Antimicrobial, Antibacterial, Anti-Gram+, Anti-Gram-
1757	5186	DRAMP21143	Myxinidin1 (Derived from Myxinidin)	Antimicrobial, Antibacterial, Anti-Gram+, Anti-Gram-
1758	5187	DRAMP21140	AMP72 (De novo synthesis)	Antimicrobial, Antibacterial, Anti-Gram+, Anti-Gram-
1759	5188	DRAMP21139	GNU7 (De novo synthesis)	Antimicrobial, Antibacterial, Anti-Gram+, Anti-Gram-, Antifungal
1760	5189	DRAMP21138	GNU6 (De novo synthesis)	Antimicrobial, Antibacterial, Anti-Gram+, Anti-Gram-, Antifungal
1761	5190	DRAMP21137	GNU5 (De novo synthesis)	Antimicrobial, Antibacterial, Anti-Gram+, Anti-Gram-, Antifungal
1762	5191	DRAMP21135	P7 (Derived from P5)	Antimicrobial, Antibacterial, Anti-Gram+, Anti-Gram-
1763	5192	DRAMP21136	P8 (Derived from P5)	Antimicrobial, Antibacterial, Anti-Gram+, Anti-Gram-
1764	5193	DRAMP21134	P6 (Derived from P5)	Antimicrobial, Antibacterial, Anti-Gram+, Anti-Gram-
1765	5194	DRAMP21133	P5 (Derived from Octa 2)	Antimicrobial, Antibacterial, Anti-Gram+, Anti-Gram-
1766	5195	DRAMP21132	P4 (Derived from P5)	Antimicrobial, Antibacterial, Anti-Gram+, Anti-Gram-
1767	5196	DRAMP21131	P3 (Derived from P5)	Antimicrobial, Antibacterial, Anti-Gram+, Anti-Gram-
1768	5197	DRAMP21130	P2 (Derived from P5)	Antimicrobial, Antibacterial, Anti-Gram+, Anti-Gram-
1769	5198	DRAMP21129	P1 (Derived from P5)	Antimicrobial, Antibacterial, Anti-Gram+, Anti-Gram-
1770	5199	DRAMP21128	T9F (Derived from RI16)	Antimicrobial, Antibacterial, Anti-Gram+, Anti-Gram-
1771	5200	DRAMP21127	T9K (Derived from RI16)	Antimicrobial, Antibacterial, Anti-Gram+, Anti-Gram-
1772	5201	DRAMP21126	T9I (Derived from RI16)	Antimicrobial, Antibacterial, Anti-Gram+, Anti-Gram-
1773	5202	DRAMP21125	T9W (Derived from RI16)	Antimicrobial, Antibacterial, Anti-Gram+, Anti-Gram-
1774	5203	DRAMP21124	RI16 (Derived from PMAP-36)	Antimicrobial, Antibacterial, Anti-Gram+, Anti-Gram-
1775	5204	DRAMP21123	KR-12-a5 (7-(D)L) (Derived from LL-37)	Antimicrobial, Antibacterial, Anti-Gram+, Anti-Gram-
1776	5205	DRAMP21122	KR-12-a5 (6-(D)L) (Derived from LL-37)	Antimicrobial, Antibacterial, Anti-Gram+, Anti-Gram-
1777	5206	DRAMP21121	KR-12-a5 (5-(D)K) (Derived from LL-37)	Antimicrobial, Antibacterial, Anti-Gram+, Anti-Gram-
1778	5207	DRAMP21119	I11R (Derived from tachyplesin I)	Antimicrobial, Antibacterial, Anti-Gram+, Anti-Gram-
1779	5208	DRAMP21120	KR-12-a5 (Derived from LL-37)	Antimicrobial, Antibacterial, Anti-Gram+, Anti-Gram-
1780	5209	DRAMP21118	I11S (Derived from tachyplesin I)	Antimicrobial, Antibacterial, Anti-Gram+, Anti-Gram-
1781	5210	DRAMP21117	Y8R (Derived from tachyplesin I)	Antimicrobial, Antibacterial, Anti-Gram+, Anti-Gram-
1782	5211	DRAMP21116	Y8S (Derived from tachyplesin I)	Antimicrobial, Antibacterial, Anti-Gram+, Anti-Gram-
1783	5212	DRAMP21115	V6R (Derived from tachyplesin I)	Antimicrobial, Antibacterial, Anti-Gram+, Anti-Gram-
1784	5213	DRAMP21114	V6S (Derived from tachyplesin I)	Antimicrobial, Antibacterial, Anti-Gram+, Anti-Gram-
1785	5214	DRAMP21111	ASA (Derived from SLZP)	Antimicrobial, Antibacterial, Anti-Gram+, Anti-Gram-, Antifungal
1786	5215	DRAMP21112	DLSA (Derived from SLZP)	Antimicrobial, Antibacterial, Anti-Gram+, Anti-Gram-, Antifungal
1787	5216	DRAMP21113	PSA (Derived from SLZP)	Antimicrobial, Antibacterial, Anti-Gram+, Anti-Gram-, Antifungal
1788	5217	DRAMP21103	L-RW (De novo synthesis)	Antimicrobial, Antibacterial, Anti-Gram+, Anti-Gram-
1789	5218	DRAMP21110	SLZP (De novo synthesis)	Antimicrobial, Antibacterial, Anti-Gram+, Anti-Gram-, Antifungal
1790	5219	DRAMP21109	FPA-Bombinin-BO (toads, amphibians, animals)	Antimicrobial, Antibacterial, Anti-Gram+, Anti-Gram-, Antifungal
1791	5220	DRAMP21108	Feleucin-K3 (Derived from Feleucin-BO1)	Antimicrobial, Antibacterial, Anti-Gram+, Anti-Gram-, Antifungal
1792	5221	DRAMP21104	Feleucin-2 (toads, amphibians, animals)	Antimicrobial, Antibacterial, Anti-Gram+, Anti-Gram-, Antifungal
1793	5224	DRAMP21107	Feleucin-BO1 (toads, amphibians, animals)	Antimicrobial, Antibacterial, Anti-Gram+, Anti-Gram-, Antifungal
1794	5225	DRAMP21232	Ranatuerin-2PLx (R2PLx; Frogs, Amphibians, Animals)	Antimicrobial, Antibacterial, Anti-Gram+, Anti-Gram-
1795	5229	DRAMP21227	IsCT-P (Derived from IsCT)	Antimicrobial, Antibacterial, Anti-Gram+, Anti-Gram-
1796	5230	DRAMP21228	IsCT-a (Derived from IsCT-P)	Antimicrobial, Antibacterial, Anti-Gram+, Anti-Gram-
1797	5231	DRAMP21225	STPk (Derived from STP)	Antimicrobial, Antibacterial, Anti-Gram+, Anti-Gram-
1798	5232	DRAMP21226	Ink (Derived from IN)	Antimicrobial, Antibacterial, Anti-Gram+, Anti-Gram-
1799	5233	DRAMP21223	IsCT-p (Derived from IsCT-P)	Antimicrobial, Antibacterial, Anti-Gram+, Anti-Gram-
1800	5234	DRAMP21224	TPk (Derived from TP)	Antimicrobial, Antibacterial, Anti-Gram+, Anti-Gram-
1801	5235	DRAMP21222	Control-4D (Derived from IK12-all L)	Antimicrobial, Antibacterial, Anti-Gram+, Anti-Gram-, Antifungal
1802	5236	DRAMP21221	Control-all D (Derived from IK12-all L)	Antimicrobial, Antibacterial, Anti-Gram+, Anti-Gram-, Antifungal

S.no.	PepID	DRAMP_ID	Name of the AMP	Activity
1803	5237	DRAMP21219	IK12-all D (Derived from IK12-all L)	Antimicrobial, Antibacterial, Anti-Gram+, Anti-Gram-, Antifungal
1804	5238	DRAMP21220	Control-all L (Derived from IK12-all L)	Antimicrobial, Antibacterial, Anti-Gram+, Anti-Gram-, Antifungal
1805	5239	DRAMP21218	IK12-all L (De novo synthesis)	Antimicrobial, Antibacterial, Anti-Gram+, Anti-Gram-, Antifungal
1806	5240	DRAMP21217	IK8-2D (Derived from IK8-all L)	Antimicrobial, Antibacterial, Anti-Gram+, Anti-Gram-, Antifungal
1807	5241	DRAMP21215	IK4-all D (Derived from IK8-all L)	Antimicrobial, Antibacterial, Anti-Gram+, Anti-Gram-, Antifungal
1808	5242	DRAMP21216	IK8-4D (Derived from IK8-all L)	Antimicrobial, Antibacterial, Anti-Gram+, Anti-Gram-, Antifungal
1809	5243	DRAMP21213	IK8-all D (Derived from IK8-all L)	Antimicrobial, Antibacterial, Anti-Gram+, Anti-Gram-, Antifungal
1810	5244	DRAMP21214	IK6-all D (Derived from IK8-all L)	Antimicrobial, Antibacterial, Anti-Gram+, Anti-Gram-, Antifungal
1811	5245	DRAMP21212	IK8-all L (De novo synthesis)	Antimicrobial, Antibacterial, Anti-Gram+, Anti-Gram-, Antifungal
1812	5255	DRAMP21202	HPA3NT3-analog (Derived from HPA3NT3)	Antimicrobial, Antibacterial, Anti-Gram+, Anti-Gram-, Antifungal
1813	5256	DRAMP21201	Magainin 2a (M2a; Frogs, Amphibians, Animals)	Antimicrobial, Antibacterial, Anti-Gram+, Anti-Gram-
1814	5257	DRAMP21200	GW-M4 (De novo synthesis)	Antimicrobial, Antibacterial, Anti-Gram+, Anti-Gram-
1815	5258	DRAMP21199	GW-M3 (De novo synthesis)	Antimicrobial, Antibacterial, Anti-Gram+, Anti-Gram-
1816	5259	DRAMP21198	GW-M1 (De novo synthesis)	Antimicrobial, Antibacterial, Anti-Gram+, Anti-Gram-
1817	5260	DRAMP21197	GW-H3 (De novo synthesis)	Antimicrobial, Antibacterial, Anti-Gram+, Anti-Gram-
1818	5261	DRAMP21196	GW-H1 (De novo synthesis)	Antimicrobial, Antibacterial, Anti-Gram+, Anti-Gram-
1819	5262	DRAMP21195	GW-A5 (De novo synthesis)	Antimicrobial, Antibacterial, Anti-Gram+, Anti-Gram-
1820	5263	DRAMP21194	GW-A4 (De novo synthesis)	Antimicrobial, Antibacterial, Anti-Gram+, Anti-Gram-
1821	5264	DRAMP21193	GW-A2 (De novo synthesis)	Antimicrobial, Antibacterial, Anti-Gram+, Anti-Gram-
1822	5265	DRAMP21192	GW-A1 (De novo synthesis)	Antimicrobial, Antibacterial, Anti-Gram+, Anti-Gram-
1823	5266	DRAMP21191	GW-Q6 (De novo synthesis)	Antimicrobial, Antibacterial, Anti-Gram+, Anti-Gram-
1824	5267	DRAMP21190	GW-Q5 (De novo synthesis)	Antimicrobial, Antibacterial, Anti-Gram+, Anti-Gram-
1825	5268	DRAMP21189	GW-Q4 (De novo synthesis)	Antimicrobial, Antibacterial, Anti-Gram+, Anti-Gram-
1826	5269	DRAMP21188	GW-Q3 (De novo synthesis)	Antimicrobial, Antibacterial, Anti-Gram+, Anti-Gram-
1827	5270	DRAMP21187	WRL4 (Derived from leucocin A)	Antimicrobial, Antibacterial, Anti-Gram+, Anti-Gram-, Antifungal
1828	5271	DRAMP21186	WRL3 (Derived from leucocin A)	Antimicrobial, Antibacterial, Anti-Gram+, Anti-Gram-, Antifungal
1829	5272	DRAMP21185	WRL2 (Derived from leucocin A)	Antimicrobial, Antibacterial, Anti-Gram+, Anti-Gram-, Antifungal
1830	5273	DRAMP21184	WR7 (Derived from leucocin A)	Antimicrobial, Antibacterial, Anti-Gram+, Anti-Gram-, Antifungal
1831	5274	DRAMP21183	WR5 (Derived from leucocin A)	Antimicrobial, Antibacterial, Anti-Gram+, Anti-Gram-, Antifungal
1832	5275	DRAMP21182	WR3 (Derived from leucocin A)	Antimicrobial, Antibacterial, Anti-Gram+, Anti-Gram-, Antifungal
1833	5276	DRAMP21181	WR1 (Derived from leucocin A)	Antimicrobial, Antibacterial, Anti-Gram+, Anti-Gram-, Antifungal
1834	5277	DRAMP21180	WG18 (Derived from leucocin A)	Antimicrobial, Antibacterial, Anti-Gram+, Anti-Gram-, Antifungal
1835	5278	DRAMP21179	HYL-26 (Derived from HYL)	Antimicrobial, Antibacterial, Anti-Gram+, Anti-Gram-, Antifungal
1836	5279	DRAMP21178	HYL-25 (Derived from HYL)	Antimicrobial, Antibacterial, Anti-Gram+, Anti-Gram-, Antifungal
1837	5280	DRAMP21177	HYL-24 (Derived from HYL)	Antimicrobial, Antibacterial, Anti-Gram+, Anti-Gram-, Antifungal
1838	5281	DRAMP21176	HYL-23 (Derived from HYL)	Antimicrobial, Antibacterial, Anti-Gram+, Anti-Gram-, Antifungal
1839	5282	DRAMP21175	HYL-22 (Derived from HYL)	Antimicrobial, Antibacterial, Anti-Gram+, Anti-Gram-, Antifungal
1840	5283	DRAMP21174	HYL-21 (Derived from HYL)	Antimicrobial, Antibacterial, Anti-Gram+, Anti-Gram-, Antifungal
1841	5284	DRAMP21173	HYL-20 (Derived from HYL)	Antimicrobial, Antibacterial, Anti-Gram+, Anti-Gram-, Antifungal
1842	5285	DRAMP21172	HYL-19 (Derived from HYL)	Antimicrobial, Antibacterial, Anti-Gram+, Anti-Gram-, Antifungal
1843	5286	DRAMP21168	HYL-15 (Derived from HYL)	Antimicrobial, Antibacterial, Anti-Gram+, Anti-Gram-, Antifungal
1844	5287	DRAMP21169	HYL-16 (Derived from HYL)	Antimicrobial, Antibacterial, Anti-Gram+, Anti-Gram-, Antifungal
1845	5288	DRAMP21170	HYL-17 (Derived from HYL)	Antimicrobial, Antibacterial, Anti-Gram+, Anti-Gram-, Antifungal
1846	5289	DRAMP21171	HYL-18 (Derived from HYL)	Antimicrobial, Antibacterial, Anti-Gram+, Anti-Gram-, Antifungal
1847	5290	DRAMP21243	pardaxin-6 (GE-6) (Derived from pardaxin)	Antimicrobial, Antibacterial, Anti-Gram+, Anti-Gram-
1848	5291	DRAMP21242	Epinecidin-8 (Derived from Epinecidin)	Antimicrobial, Antibacterial, Anti-Gram+, Anti-Gram-
1849	5292	DRAMP21241	Epinecidin-1 (Derived from Epinecidin)	Antimicrobial, Antibacterial, Anti-Gram+, Anti-Gram-
1850	5293	DRAMP21240	FK13-a7 (Derived from FK13)	Antimicrobial, Antibacterial, Anti-Gram+, Anti-Gram-
1851	5294	DRAMP21239	FK13-a6 (Derived from FK13)	Antimicrobial, Antibacterial, Anti-Gram+, Anti-Gram-
1852	5295	DRAMP21238	FK13-a5 (Derived from FK13)	Antimicrobial, Antibacterial, Anti-Gram+, Anti-Gram-
1853	5296	DRAMP21237	FK13-a4 (Derived from FK13)	Antimicrobial, Antibacterial, Anti-Gram+, Anti-Gram-
1854	5297	DRAMP21236	FK13-a3 (Derived from FK13)	Antimicrobial, Antibacterial, Anti-Gram+, Anti-Gram-
1855	5298	DRAMP21235	FK13-a2 (Derived from FK13)	Antimicrobial, Antibacterial, Anti-Gram+, Anti-Gram-

S.no.	PepID	DRAMP_ID	Name of the AMP	Activity
1856	5299	DRAMP21167	HYL-13 (Derived from HYL)	Antimicrobial, Antibacterial, Anti-Gram+, Anti-Gram-, Antifungal
1857	5300	DRAMP21234	FK13-a1 (Derived from FK13)	Antimicrobial, Antibacterial, Anti-Gram+, Anti-Gram-
1858	5301	DRAMP21233	R2PLx-22 (Derived from R2PLx)	Antimicrobial, Antibacterial, Anti-Gram+, Anti-Gram-
1859	5302	DRAMP21244	TsAP-S1 (Derived from TsAP-1)	Antimicrobial, Antibacterial, Anti-Gram+, Anti-Gram-, Antifungal
1860	5303	DRAMP21245	TsAP-S2 (Derived from TsAP-2)	Antimicrobial, Antibacterial, Anti-Gram+, Anti-Gram-, Antifungal
1861	5304	DRAMP21246	pEM-2 (Derived from the venom of the snake Bothrops asper)	Antimicrobial, Antibacterial, Anti-Gram+, Anti-Gram-
1862	5305	DRAMP21247	PV (Derived from pEM-2 and MP-VT1)	Antimicrobial, Antibacterial, Anti-Gram+, Anti-Gram-
1863	5306	DRAMP21248	BVP (Derived from pEM-2 and MP-VT1 and MP-B)	Antimicrobial, Antibacterial, Anti-Gram+, Anti-Gram-
1864	5307	DRAMP21249	PVP (Derived from MP-B and MP-VT1)	Antimicrobial, Antibacterial, Anti-Gram+, Anti-Gram-
1865	5308	DRAMP21250	PV3 (Derived from pEM-2 and MP-VT1)	Antimicrobial, Antibacterial, Anti-Gram+, Anti-Gram-
1866	5311	DRAMP21253	AaeAP1a (Derived from AaeAP1)	Antimicrobial, Antibacterial, Anti-Gram+, Anti-Gram-, Antifungal
1867	5312	DRAMP21254	AaeAP2a (Derived from AaeAP2)	Antimicrobial, Antibacterial, Anti-Gram+, Anti-Gram-, Antifungal
1868	5313	DRAMP21255	WL1 (Derived from CP-1)	Antimicrobial, Antibacterial, Anti-Gram+, Anti-Gram-
1869	5314	DRAMP21256	WL2 (Derived from CP-1)	Antimicrobial, Antibacterial, Anti-Gram+, Anti-Gram-
1870	5315	DRAMP21257	WL3 (Derived from CP-1)	Antimicrobial, Antibacterial, Anti-Gram+, Anti-Gram-
1871	5316	DRAMP21258	Cecropin P1 (CP-1) (nematodes, animals)	Antimicrobial, Antibacterial, Anti-Gram+, Anti-Gram-
1872	5317	DRAMP21259	Scolopendin 1 (Centipedes, Arthropoda, Animals)	Antimicrobial, Antibacterial, Anti-Gram+, Anti-Gram-, Antifungal
1873	5318	DRAMP21260	KL0A10 (De novo synthesis)	Antimicrobial, Antibacterial, Anti-Gram+, Anti-Gram-
1874	5319	DRAMP21261	KL4A6 (De novo synthesis)	Antimicrobial, Antibacterial, Anti-Gram+, Anti-Gram-
1875	5320	DRAMP21262	KL6A4 (De novo synthesis)	Antimicrobial, Antibacterial, Anti-Gram+, Anti-Gram-
1876	5321	DRAMP21263	KL10A0 (De novo synthesis)	Antimicrobial, Antibacterial, Anti-Gram+, Anti-Gram-
1877	5322	DRAMP21264	LK (De novo synthesis)	Antimicrobial, Antibacterial, Anti-Gram+, Anti-Gram-
1878	5323	DRAMP21265	LK-L1A (Derived from LK)	Antimicrobial, Antibacterial, Anti-Gram+, Anti-Gram-
1879	5324	DRAMP21266	LK-L4A (Derived from LK)	Antimicrobial, Antibacterial, Anti-Gram+, Anti-Gram-
1880	5325	DRAMP21267	LK-L5A (Derived from LK)	Antimicrobial, Antibacterial, Anti-Gram+, Anti-Gram-
1881	5326	DRAMP21268	LK-L7A (Derived from LK)	Antimicrobial, Antibacterial, Anti-Gram+, Anti-Gram-
1882	5327	DRAMP21269	LK-L8A (Derived from LK)	Antimicrobial, Antibacterial, Anti-Gram+, Anti-Gram-
1883	5328	DRAMP21270	LK-L11A (Derived from LK)	Antimicrobial, Antibacterial, Anti-Gram+, Anti-Gram-
1884	5329	DRAMP21271	LK-L12A (Derived from LK)	Antimicrobial, Antibacterial, Anti-Gram+, Anti-Gram-
1885	5330	DRAMP21272	LK-L14A (Derived from LK)	Antimicrobial, Antibacterial, Anti-Gram+, Anti-Gram-
1886	5331	DRAMP21273	LK-L8G (Derived from LK)	Antimicrobial, Antibacterial, Anti-Gram+, Anti-Gram-
1887	5332	DRAMP21274	LK-L8S (Derived from LK)	Antimicrobial, Antibacterial, Anti-Gram+, Anti-Gram-
1888	5333	DRAMP21275	LK-L8P (Derived from LK)	Antimicrobial, Antibacterial, Anti-Gram+, Anti-Gram-
1889	5334	DRAMP21276	LK-L8N (Derived from LK)	Antimicrobial, Antibacterial, Anti-Gram+, Anti-Gram-
1890	5335	DRAMP21277	LK-L8Q (Derived from LK)	Antimicrobial, Antibacterial, Anti-Gram+, Anti-Gram-
1891	5336	DRAMP21278	LK-L8D (Derived from LK)	Antimicrobial, Antibacterial, Anti-Gram+, Anti-Gram-
1892	5337	DRAMP21279	LK-L8E (Derived from LK)	Antimicrobial, Antibacterial, Anti-Gram+, Anti-Gram-
1893	5338	DRAMP21280	LK-L8K (Derived from LK)	Antimicrobial, Antibacterial, Anti-Gram+, Anti-Gram-
1894	5339	DRAMP21281	LK-L8H (Derived from LK)	Antimicrobial, Antibacterial, Anti-Gram+, Anti-Gram-
1895	5340	DRAMP21282	Lt-F1A (Derived from Lt)	Antimicrobial, Antibacterial, Anti-Gram+, Anti-Gram-
1896	5341	DRAMP21283	Lt-I4A (Derived from Lt)	Antimicrobial, Antibacterial, Anti-Gram+, Anti-Gram-
1897	5342	DRAMP21284	Lt-V5A (Derived from Lt)	Antimicrobial, Antibacterial, Anti-Gram+, Anti-Gram-
1898	5343	DRAMP21285	Lt-I8A (Derived from Lt)	Antimicrobial, Antibacterial, Anti-Gram+, Anti-Gram-
1899	5344	DRAMP21286	Lt-F11A (Derived from Lt)	Antimicrobial, Antibacterial, Anti-Gram+, Anti-Gram-
1900	5345	DRAMP21287	Lt-F12A (Derived from Lt)	Antimicrobial, Antibacterial, Anti-Gram+, Anti-Gram-
1901	5346	DRAMP21288	Lt-I4G (Derived from Lt)	Antimicrobial, Antibacterial, Anti-Gram+, Anti-Gram-
1902	5347	DRAMP21289	Lt-I4S (Derived from Lt)	Antimicrobial, Antibacterial, Anti-Gram+, Anti-Gram-
1903	5348	DRAMP21290	Lt-I4N (Derived from Lt)	Antimicrobial, Antibacterial, Anti-Gram+, Anti-Gram-
1904	5349	DRAMP21291	Lt-I4Q (Derived from Lt)	Antimicrobial, Antibacterial, Anti-Gram+, Anti-Gram-
1905	5350	DRAMP21292	Lt-I4H (Derived from Lt)	Antimicrobial, Antibacterial, Anti-Gram+, Anti-Gram-
1906	5351	DRAMP21293	Lt-V5G (Derived from Lt)	Antimicrobial, Antibacterial, Anti-Gram+, Anti-Gram-
1907	5352	DRAMP21294	Lt-V5S (Derived from Lt)	Antimicrobial, Antibacterial, Anti-Gram+, Anti-Gram-
1908	5353	DRAMP21295	Lt-V5N (Derived from Lt)	Antimicrobial, Antibacterial, Anti-Gram+, Anti-Gram-

S.no.	PepID	DRAMP_ID	Name of the AMP	Activity
1909	5354	DRAMP21296	Lt-V5Q (Derived from Lt)	Antimicrobial, Antibacterial, Anti-Gram+, Anti-Gram-
1910	5355	DRAMP21297	Lt-V5H (Derived from Lt)	Antimicrobial, Antibacterial, Anti-Gram+, Anti-Gram-
1911	5356	DRAMP21298	Lt-F11G (Derived from Lt)	Antimicrobial, Antibacterial, Anti-Gram+, Anti-Gram-
1912	5357	DRAMP21299	Lt-F11S (Derived from Lt)	Antimicrobial, Antibacterial, Anti-Gram+, Anti-Gram-
1913	5358	DRAMP21300	Lt-F11N (Derived from Lt)	Antimicrobial, Antibacterial, Anti-Gram+, Anti-Gram-
1914	5359	DRAMP21301	Lt-F11Q (Derived from Lt)	Antimicrobial, Antibacterial, Anti-Gram+, Anti-Gram-
1915	5360	DRAMP21302	Lt-F11H (Derived from Lt)	Antimicrobial, Antibacterial, Anti-Gram+, Anti-Gram-
1916	5361	DRAMP21303	A7-PMAP-23 (Derived from PMAP-23)	Antimicrobial, Antibacterial, Anti-Gram+, Anti-Gram-
1917	5362	DRAMP21304	A21-PMAP-23 (Derived from PMAP-23)	Antimicrobial, Antibacterial, Anti-Gram+, Anti-Gram-
1918	5363	DRAMP21305	R8 (De novo synthesis)	Antimicrobial, Antibacterial, Anti-Gram+, Anti-Gram-
1919	5364	DRAMP21306	TL-1 (Derived from Temporin-1Tl (TL))	Antimicrobial, Antibacterial, Anti-Gram+, Anti-Gram-
1920	5365	DRAMP21307	TL-2 (Derived from Temporin-2Tl (TL))	Antimicrobial, Antibacterial, Anti-Gram+, Anti-Gram-
1921	5366	DRAMP21308	TL-3 (Derived from Temporin-3Tl (TL))	Antimicrobial, Antibacterial, Anti-Gram+, Anti-Gram-
1922	5367	DRAMP21309	TL-4 (Derived from Temporin-4Tl (TL))	Antimicrobial, Antibacterial, Anti-Gram+, Anti-Gram-
1923	5368	DRAMP21311	2W-1 (Derived from PMAP-36)	Antimicrobial, Antibacterial, Anti-Gram+, Anti-Gram-
1924	5369	DRAMP21312	2W-2 (Derived from PMAP-36)	Antimicrobial, Antibacterial, Anti-Gram+, Anti-Gram-
1925	5370	DRAMP21313	2W-3 (Derived from PMAP-36)	Antimicrobial, Antibacterial, Anti-Gram+, Anti-Gram-
1926	5371	DRAMP21314	3W-1 (Derived from PMAP-36)	Antimicrobial, Antibacterial, Anti-Gram+, Anti-Gram-
1927	5372	DRAMP21315	3W-2 (Derived from PMAP-36)	Antimicrobial, Antibacterial, Anti-Gram+, Anti-Gram-
1928	5373	DRAMP21316	3W-3 (Derived from PMAP-36)	Antimicrobial, Antibacterial, Anti-Gram+, Anti-Gram-
1929	5374	DRAMP21317	3W-4 (Derived from PMAP-36)	Antimicrobial, Antibacterial, Anti-Gram+, Anti-Gram-
1930	5375	DRAMP21318	3W-5 (Derived from PMAP-36)	Antimicrobial, Antibacterial, Anti-Gram+, Anti-Gram-
1931	5376	DRAMP21319	3V (Derived from PMAP-36)	Antimicrobial, Antibacterial, Anti-Gram+, Anti-Gram-
1932	5377	DRAMP21320	3L (Derived from PMAP-36)	Antimicrobial, Antibacterial, Anti-Gram+, Anti-Gram-
1933	5378	DRAMP21321	4W (Derived from PMAP-36)	Antimicrobial, Antibacterial, Anti-Gram+, Anti-Gram-
1934	5379	DRAMP21322	RTV (Derived from PMAP-36)	Antimicrobial, Antibacterial, Anti-Gram+, Anti-Gram-
1935	5380	DRAMP21323	RTI (Derived from PMAP-36)	Antimicrobial, Antibacterial, Anti-Gram+, Anti-Gram-
1936	5381	DRAMP21324	RTF (Derived from PMAP-36)	Antimicrobial, Antibacterial, Anti-Gram+, Anti-Gram-
1937	5382	DRAMP21325	RTL (Derived from PMAP-36)	Antimicrobial, Antibacterial, Anti-Gram+, Anti-Gram-
1938	5383	DRAMP21326	RLR (Derived from PMAP-36)	Antimicrobial, Antibacterial, Anti-Gram+, Anti-Gram-
1939	5384	DRAMP21327	RVR (Derived from PMAP-36)	Antimicrobial, Antibacterial, Anti-Gram+, Anti-Gram-
1940	5385	DRAMP21328	RTR (Derived from PMAP-36)	Antimicrobial, Antibacterial, Anti-Gram+, Anti-Gram-
1941	5386	DRAMP21329	RFR (Derived from PMAP-36)	Antimicrobial, Antibacterial, Anti-Gram+, Anti-Gram-
1942	5387	DRAMP21330	KVK (Derived from PMAP-36)	Antimicrobial, Antibacterial, Anti-Gram+, Anti-Gram-
1943	5388	DRAMP21331	KLK (Derived from PMAP-36)	Antimicrobial, Antibacterial, Anti-Gram+, Anti-Gram-
1944	5389	DRAMP21332	KIK (Derived from PMAP-36)	Antimicrobial, Antibacterial, Anti-Gram+, Anti-Gram-
1945	5390	DRAMP21333	RVK (Derived from PMAP-36)	Antimicrobial, Antibacterial, Anti-Gram+, Anti-Gram-
1946	5391	DRAMP21334	Ranatuerin-2Pb (Frogs, amphibians, animals)	Antimicrobial, Antibacterial, Anti-Gram+, Anti-Gram-, Antifungal
1947	5392	DRAMP21335	RPa (Frogs, amphibians, animals)	Antimicrobial, Antibacterial, Anti-Gram+, Anti-Gram-, Antifungal
1948	5393	DRAMP21336	RPb (Frogs, amphibians, animals)	Antimicrobial, Antibacterial, Anti-Gram+, Anti-Gram-, Antifungal
1949	5394	DRAMP21337	BMAP-27 (Bovine, mammals, animals)	Antimicrobial, Antibacterial, Anti-Gram+, Anti-Gram-
1950	5395	DRAMP21338	[Arg]3-VmCT1-NH2 (Derived from VmCT1)	Antimicrobial, Antibacterial, Anti-Gram+, Anti-Gram-, Antifungal
1951	5396	DRAMP21339	[Arg]7-VmCT1-NH2 (Derived from VmCT1)	Antimicrobial, Antibacterial, Anti-Gram+, Anti-Gram-, Antifungal
1952	5397	DRAMP21340	[Arg]11-VmCT1-NH2 (Derived from VmCT1)	Antimicrobial, Antibacterial, Anti-Gram+, Anti-Gram-, Antifungal
1953	5398	DRAMP21341	[Gly]1-VmCT1-NH2 (Derived from VmCT1)	Antimicrobial, Antibacterial, Anti-Gram+, Anti-Gram-, Antifungal
1954	5399	DRAMP21342	[Pro]8-VmCT1-NH2 (Derived from VmCT1)	Antimicrobial, Antibacterial, Anti-Gram+, Anti-Gram-, Antifungal
1955	5400	DRAMP21343	[Leu]9-VmCT1-NH2 (Derived from VmCT1)	Antimicrobial, Antibacterial, Anti-Gram+, Anti-Gram-, Antifungal
1956	5401	DRAMP21344	[Phe]9-VmCT1-NH2 (Derived from VmCT1)	Antimicrobial, Antibacterial, Anti-Gram+, Anti-Gram-, Antifungal
1957	5402	DRAMP21345	[Leu]12-VmCT1-NH2 (Derived from VmCT1)	Antimicrobial, Antibacterial, Anti-Gram+, Anti-Gram-, Antifungal
1958	5403	DRAMP21346	[Tyr]12-VmCT1-NH2 (Derived from VmCT1)	Antimicrobial, Antibacterial, Anti-Gram+, Anti-Gram-, Antifungal
1959	5423	DRAMP21366	[Lys]1-VmCT1-NH2 (Derived from VmCT1)	Antimicrobial, Antibacterial, Anti-Gram+, Anti-Gram-, Antifungal
1960	5425	DRAMP21368	[Lys]1[Lys]12-VmCT1-NH2 (Derived from VmCT1)	Antimicrobial, Antibacterial, Anti-Gram+, Anti-Gram-, Antifungal
1961	5426	DRAMP21369	[Lys]3[Lys]7-VmCT1-NH2 (Derived from VmCT1)	Antimicrobial, Antibacterial, Anti-Gram+, Anti-Gram-, Antifungal

S.no.	PepID	DRAMP_ID	Name of the AMP	Activity
1962	5427	DRAMP21370	[Lys]3[Lys]11-VmCT1-NH2 (Derived from VmCT1)	Antimicrobial, Antibacterial, Anti-Gram+, Anti-Gram-, Antifungal
1963	5428	DRAMP21371	[Lys]7[Lys]11-VmCT1-NH2 (Derived from VmCT1)	Antimicrobial, Antibacterial, Anti-Gram+, Anti-Gram-, Antifungal
1964	5429	DRAMP21372	[Lys]3[Lys]7[Lys]11-VmCT1-NH2 (Derived from VmCT1)	Antimicrobial, Antibacterial, Anti-Gram+, Anti-Gram-, Antifungal
1965	5447	DRAMP21390	K17 (Derived from ATG16)	Antimicrobial, Antibacterial, Anti-Gram+, Anti-Gram-, Antifungal
1966	5448	DRAMP21391	K18 (Derived from ATG16)	Antimicrobial, Antibacterial, Anti-Gram+, Anti-Gram-, Antifungal
1967	5449	DRAMP21392	K22 (Derived from ATG16)	Antimicrobial, Antibacterial, Anti-Gram+, Anti-Gram-, Antifungal
1968	5450	DRAMP21393	K22.2 (Derived from ATG16)	Antimicrobial, Antibacterial, Anti-Gram+, Anti-Gram-, Antifungal
1969	5451	DRAMP21394	K30 (Derived from ATG16)	Antimicrobial, Antibacterial, Anti-Gram+, Anti-Gram-, Antifungal
1970	5452	DRAMP21395	K31 (Derived from ATG16)	Antimicrobial, Antibacterial, Anti-Gram+, Anti-Gram-, Antifungal
1971	5453	DRAMP21396	K33 (Derived from ATG16)	Antimicrobial, Antibacterial, Anti-Gram+, Anti-Gram-, Antifungal
1972	5454	DRAMP21397	K36 (Derived from ATG16)	Antimicrobial, Antibacterial, Anti-Gram+, Anti-Gram-, Antifungal
1973	5455	DRAMP21398	K46 (Derived from ATG16)	Antimicrobial, Antibacterial, Anti-Gram+, Anti-Gram-, Antifungal
1974	5457	DRAMP21400	NBC2253 (De Novo Synthesis)	Antimicrobial, Antibacterial, Anti-Gram+, Anti-Gram-
1975	5458	DRAMP21401	NBC2254 (De Novo Synthesis)	Antimicrobial, Antibacterial, Anti-Gram+, Anti-Gram-
1976	5459	DRAMP21402	B1 (Derived from LL-37 and BMAP-27)	Antimicrobial, Antibacterial, Anti-Gram+, Anti-Gram-
1977	5460	DRAMP21403	peptide 1 (De Novo Synthesis)	Antimicrobial, Antibacterial, Anti-Gram+, Anti-Gram-
1978	5461	DRAMP21404	peptide 2 (De Novo Synthesis)	Antimicrobial, Antibacterial, Anti-Gram+, Anti-Gram-
1979	5462	DRAMP21405	LGL13K (De Novo Synthesis)	Antimicrobial, Antibacterial, Anti-Gram+, Anti-Gram-
1980	5463	DRAMP21406	DGL13K (De Novo Synthesis)	Antimicrobial, Antibacterial, Anti-Gram+, Anti-Gram-
1981	5464	DRAMP21407	Bac4K (Derived from CAMPs)	Antimicrobial, Antibacterial, Anti-Gram+, Anti-Gram-
1982	5465	DRAMP21408	Bac3W (Derived from CAMPs)	Antimicrobial, Antibacterial, Anti-Gram+, Anti-Gram-
1983	5466	DRAMP21409	dBac (Derived from CAMPs)	Antimicrobial, Antibacterial, Anti-Gram+, Anti-Gram-
1984	5467	DRAMP21410	dBac4K (Derived from CAMPs)	Antimicrobial, Antibacterial, Anti-Gram+, Anti-Gram-
1985	5468	DRAMP21411	dBac3W (Derived from CAMPs)	Antimicrobial, Antibacterial, Anti-Gram+, Anti-Gram-
1986	5469	DRAMP21412	dBacK (Derived from CAMPs)	Antimicrobial, Antibacterial, Anti-Gram+, Anti-Gram-
1987	5470	DRAMP21413	dBacK- (cap) (Derived from CAMPs)	Antimicrobial, Antibacterial, Anti-Gram+, Anti-Gram-
1988	5471	DRAMP21414	CecB Q53 (Derived from CecB E53)	Antimicrobial, Antibacterial, Anti-Gram+, Anti-Gram-, Antifungal
1989	5472	DRAMP21415	α4-short (Derived from α4)	Antimicrobial, Antibacterial, Anti-Gram+, Anti-Gram-
1990	5473	DRAMP21416	WV (De Novo Synthesis)	Antimicrobial, Antibacterial, Anti-Gram+, Anti-Gram-
1991	5474	DRAMP21417	WI (De Novo Synthesis)	Antimicrobial, Antibacterial, Anti-Gram+, Anti-Gram-
1992	5475	DRAMP21418	WF (De Novo Synthesis)	Antimicrobial, Antibacterial, Anti-Gram+, Anti-Gram-
1993	5476	DRAMP21419	WW (De Novo Synthesis)	Antimicrobial, Antibacterial, Anti-Gram+, Anti-Gram-
1994	5481	DRAMP21424	B1 (De Novo Synthesis)	Antimicrobial, Antibacterial, Anti-Gram+, Anti-Gram-
1995	5482	DRAMP21425	peptide 2 (Derived from B1)	Antimicrobial, Antibacterial, Anti-Gram+, Anti-Gram-
1996	5483	DRAMP21426	peptide 3 (Derived from B1)	Antimicrobial, Antibacterial, Anti-Gram+, Anti-Gram-
1997	5484	DRAMP21427	peptide 4 (Derived from B1)	Antimicrobial, Antibacterial, Anti-Gram+, Anti-Gram-
1998	5485	DRAMP21428	peptide 5 (Derived from B1)	Antimicrobial, Antibacterial, Anti-Gram+, Anti-Gram-
1999	5486	DRAMP21429	peptide 6 (Derived from B1)	Antimicrobial, Antibacterial, Anti-Gram+, Anti-Gram-
2000	5487	DRAMP21430	peptide 7 (Derived from B1)	Antimicrobial, Antibacterial, Anti-Gram+, Anti-Gram-
2001	5488	DRAMP21431	peptide 8 (Derived from B1)	Antimicrobial, Antibacterial, Anti-Gram+, Anti-Gram-
2002	5489	DRAMP21432	peptide 9 (Derived from B1)	Antimicrobial, Antibacterial, Anti-Gram+, Anti-Gram-
2003	5490	DRAMP21433	peptide 10 (Derived from B1)	Antimicrobial, Antibacterial, Anti-Gram+, Anti-Gram-
2004	5491	DRAMP21434	peptide 11 (Derived from B1)	Antimicrobial, Antibacterial, Anti-Gram+, Anti-Gram-
2005	5494	DRAMP21437	peptide 14 (Derived from B1)	Antimicrobial, Antibacterial, Anti-Gram+, Anti-Gram-
2006	5495	DRAMP21438	peptide 15 (Derived from B1)	Antimicrobial, Antibacterial, Anti-Gram+, Anti-Gram-
2007	5496	DRAMP21439	peptide 16 (Derived from B1)	Antimicrobial, Antibacterial, Anti-Gram+, Anti-Gram-
2008	5497	DRAMP21440	peptide 17 (Derived from B1)	Antimicrobial, Antibacterial, Anti-Gram+, Anti-Gram-
2009	5501	DRAMP21444	peptide 21 (Derived from B1)	Antimicrobial, Antibacterial, Anti-Gram+, Anti-Gram-
2010	5505	DRAMP21448	peptide 25 (Derived from B1)	Antimicrobial, Antibacterial, Anti-Gram+, Anti-Gram-
2011	5508	DRAMP21451	peptide 28 (Derived from B1)	Antimicrobial, Antibacterial, Anti-Gram+, Anti-Gram-
2012	5509	DRAMP21452	peptide 29 (Derived from B1)	Antimicrobial, Antibacterial, Anti-Gram+, Anti-Gram-
2013	5510	DRAMP21453	Hybrid (Derived from Melittin and thanatin)	Antimicrobial, Antibacterial, Anti-Gram+, Anti-Gram-
2014	5511	DRAMP21454	PLP1 (Insects, animals)	Antimicrobial, Antibacterial, Anti-Gram+, Anti-Gram-, Antifungal

S.no.	PepID	DRAMP_ID	Name of the AMP	Activity
2015	5512	DRAMP21455	PLP2 (Insects, animals)	Antimicrobial, Antibacterial, Anti-Gram+, Anti-Gram-, Antifungal
2016	5513	DRAMP21456	PLP3 (Insects, animals)	Antimicrobial, Antibacterial, Anti-Gram+, Anti-Gram-, Antifungal
2017	5514	DRAMP21457	PLP4 (Insects, animals)	Antimicrobial, Antibacterial, Anti-Gram+, Anti-Gram-, Antifungal
2018	5515	DRAMP21458	PLP5 (Insects, animals)	Antimicrobial, Antibacterial, Anti-Gram+, Anti-Gram-, Antifungal
2019	5516	DRAMP21459	PLP6 (Insects, animals)	Antimicrobial, Antibacterial, Anti-Gram+, Anti-Gram-, Antifungal
2020	5517	DRAMP21460	PQ (De Novo Synthesis)	Antimicrobial, Antibacterial, Anti-Gram+, Anti-Gram-
2021	5518	DRAMP21461	PP (De Novo Synthesis)	Antimicrobial, Antibacterial, Anti-Gram+, Anti-Gram-
2022	5519	DRAMP21462	GG (De Novo Synthesis)	Antimicrobial, Antibacterial, Anti-Gram+, Anti-Gram-
2023	5520	DRAMP21463	Qa (De Novo Synthesis)	Antimicrobial, Antibacterial, Anti-Gram+, Anti-Gram-
2024	5521	DRAMP21464	Qna (De Novo Synthesis)	Antimicrobial, Antibacterial, Anti-Gram+, Anti-Gram-
2025	5522	DRAMP21465	P1-LI-1577 (De Novo Synthesis)	Antimicrobial, Antibacterial, Anti-Gram+, Anti-Gram-
2026	5523	DRAMP21466	P2-LI-1298 (De Novo Synthesis)	Antimicrobial, Antibacterial, Anti-Gram+, Anti-Gram-
2027	5524	DRAMP21467	P3-LI-2085 (De Novo Synthesis)	Antimicrobial, Antibacterial, Anti-Gram+, Anti-Gram-
2028	5525	DRAMP21310	RK12 (Derived from PMAP-36)	Antimicrobial, Antibacterial, Anti-Gram+, Anti-Gram-
2029	5528	DRAMP21494	MEP-N	Antimicrobial, Antibacterial, Anti-Gram+, Anti-Gram-, Antifungal
2030	5529	DRAMP21579	Val-nHSLP	Antimicrobial, Antibacterial, Anti-Gram+, Anti-Gram-
2031	5538	DRAMP21616	DRIM	Antimicrobial, Antibacterial, Anti-Gram+, Anti-Gram-
2032	5539	DRAMP21618	WWSP	Antimicrobial, Antibacterial, Anti-Gram+, Anti-Gram-
2033	5540	DRAMP21620	KFGF	Antimicrobial, Antibacterial, Anti-Gram+, Anti-Gram-
2034	5541	DRAMP21622	MAP-1	Antimicrobial, Antibacterial, Anti-Gram+, Anti-Gram-
2035	5543	DRAMP21627	E2EM15W	Antimicrobial, Antibacterial, Anti-Gram+, Anti-Gram-
